# ﻿Manual of North American Agromyzidae (Diptera, Schizophora), with revision of the fauna of the “Delmarva” states

**DOI:** 10.3897/zookeys.1051.64603

**Published:** 2021-07-29

**Authors:** Owen Lonsdale

**Affiliations:** 1 Agriculture & Agri-Food Canada, 960 Carling Avenue, Ottawa, ON, K1A 0C6, Canada Agriculture & Agri-Food Canada Ottawa Canada

**Keywords:** Agromyzidae, manual, Nearctic, new species, recombination, revision, synonymy

## Abstract

Тhe agromyzid (Diptera: Schizophora: Agromyzidae) fauna of America north of Mexico is described in the first part of this publication, including a genus key and discussions on morphology, life history and classification. The second part is a species-level revision of the family in the “Delmarva” states of the United States of America, that is, of the District of Columbia and the surrounding states of Delaware, Maryland, Virginia and West Virginia. The fauna of this region includes 156 species.

This study presents 346 new state and provincial records and 23 new country records, two of which are new continental records (*Agromyzaabiens* Zetterstedt and *A.apfelbecki* Strobl). *Liriomyzaendiviae* Hering is no longer considered to occur in North America. Fifteen species are newly described: *Agromyzaechinalis***sp. nov.**, *Melanagromyzabrunkei***sp. nov.**, *M.eoflacensis***sp. nov.**, *M.glyptos***sp. nov.**, *M.rutella***sp. nov.**, *Ophiomyiacapitolia***sp. nov.**, *O.cuprea***sp. nov.**, *O.galiodes***sp. nov.**, *O.heleios***sp. nov.**, *O.kalia***sp. nov.**, *O.laticolis***sp. nov.**, Cerodontha (Poemyza) ungula**sp. nov.**, *Phytomyzaavicursa***sp. nov.**, *P.catenula***sp. nov.**, and *P.winkleri***sp. nov.** Four new species-level synonyms and one genus-level synonym are provided: *Agromyzamarmorensis* Spencer **syn. nov.** is included as a synonym of *A.aristata* Malloch; *Melanagromyzafastosa* Spencer, **syn. nov.** is included as a junior synonym of *Ophiomyiatiliae* (Couden); *Melanagromyzaverbesinae* Spencer is considered a synonym of *M.vernoniana* Steyskal; *Phytomyzaranunculoides* Spencer, **syn. nov.** is included as a junior synonym of *Phytomyzaloewii* Hendel; the genus *Liomycina* Enderlein, **syn. nov.** is included as a junior synonym of *Phytobia* Lioy. *Ophiomyiaultima* (Spencer), **comb. nov.** is recombined from *Melanagromyza*. *Euhexomyzaalbicula* Spencer, **stat. reinst.**, **comb. nov.** is resurrected from synonymy with *E.winnemanae* (Malloch). New host records are given.

## ﻿Introduction

As an agriculturally and ecologically important group, it is critical to understand the systematics, biology, distribution, and impact of species in the family Agromyzidae, a ubiquitous and diverse acalyptrate family of true flies (Diptera: Schizophora). While comprehensive sources on the North American fauna, such as Spencer (1969, 1987) and Spencer and Steyskal (1986b), already discuss these topics, important studies and discoveries have appeared since the time of those publications and it is necessary to provide updates.

### ﻿Scope of the present study

At higher taxonomic levels, the present work examines those subfamilies and genera occurring in Canada and the continental United States (that is, the Nearctic Region north of Mexico), providing diagnoses and brief discussions on their relationships, classification, life history and diversity. To facilitate generic identification, an identification key is provided, accompanied by illustrations of the highly informative and diagnostic male genitalia, and photographs of live and preserved specimens. Discussions include the results of recent revisionary and phylogenetic studies, including redefinitions of *Liriomyza* Mik (Lonsdale 2011, 2017), *Phytomyza* Fallén (Winkler et al. 2009), *Nemorimyza* Frey (Zlobin 1996) and members of the *Ophiomyia* genus group (Lonsdale 2014), among others.

The economic importance of the family was overviewed by Spencer (1973). An update on many of the most agriculturally significant species, including their hosts and methods of control, is currently being prepared (Lonsdale et al., in manuscript) and need not be discussed here.

At the species level, this mostly collection-based study revises the fauna of the “Delmarva” states (discussed below), and is intended as a regional snapshot of the family. It will contribute to a more accurate understanding of the group when used alongside other comprehensive regional works such Griffiths’ series on the boreal *Phytomyza*, Lonsdale and Scheffer’s (2011) revision of the Nearctic holly leaf miners, Boucher’s Nearctic revisions (2002, 2004, 2008), Frick’s (1956)*Calycomyza* Hendel revision, Lonsdale’s (2011, 2017) *Liriomyza* revisions, Shi and Gaimari’s (2015) revision of the Californian *Melanagromyza* Hendel, the primarily northeastern United States surveys of Eiseman et al. (2018–2021), the catalogues compiled by Frick (1952a, 1959, 1965), and of course, Spencer (1969) and Spencer and Steyskal (1986b). The latter two works revised all Agromyzidae known to occur in Canada and Alaska, and the lower continental United States, respectively, and have served as two of the most useful stand-alone resources for the family but need updating.

More broadly, the author hopes that this manual will generate more interest in this relatively difficult and neglected family in North America and facilitate easier identifications, thereby encouraging collectors to gather more field data, preserve more specimens, and explore parts of North America where agromyzids are largely unknown and undescribed. This is an exciting prospect considering that relatively little is known of the North American Agromyzidae outside of California and the northeast, and even in these regions, many new discoveries continue to accumulate. Furthermore, while the morphology of most species in the family can indeed often be accurately characterised as small, somewhat monotonous in appearance (at least externally) and sometimes difficult to work with, it is hoped that the photographs and illustrations given here portray the hidden charm of these animals, especially when considering the often striking and elaborate male genitalia.

### ﻿The “Delmarva” States

The name “Delmarva”, as used here, is an older collective term referring to the District of Columbia and the surrounding states of Delaware, Maryland, Virginia, and West Virginia (Figs 5, 6). Its modern usage is restricted to the Delmarva Peninsula, flanked by the Delaware and Chesapeake Bays. Defining characteristics of these states (and District), including geography, are discussed in Mathis and Foster (2007) and Murphy et al. (2018). These authors also provide interesting historical context, including background on a series of papers examining the dipteran fauna of the greater Washington, D.C. area that eventually became treatments of individual families in the Delmarva states. The present work is a continuation of this series.

### ﻿Agromyzidae of the Delmarva states

All species of Agromyzidae verified as being present in the Delmarva states are here keyed, described and illustrated. It is likely that the fauna of the region is much larger than represented however, if one extrapolates the known distribution of other northeastern species and their host plants, and considers the likelihood of there being many more species new to science yet to be discovered. Recent publications have illustrated that undescribed taxa are still in abundance in the northeast despite the long history of research there. Future discoveries will also certainly include taxa previously known from more distant localities that represent either recent introductions of invasive species, or previously unknown populations of more widely distributed species. These assumptions are supported by comparison to the more diverse and recently revised fauna of Hungary, initiated by Papp and Černý (2016), although unlike here, that work includes unconfirmed species predicted to be in the country. Hungary is slightly more northern in position compared to the Delmarva states and slightly smaller in size (ca. 93,030 km2, versus 99,420 km2). Since this work excludes species not yet known to occur in the region, reference to the broader Nearctic treatments of Spencer (1969) and Spencer and Steyskal (1986b) is suggested in case an identification is uncertain; reference to host plant catalogues (such as Spencer 1990 and Benavent-Corai et al. 2005) and non-Nearctic taxonomic treatments is also recommended.

In total, this study presents 346 new state and provincial records and 23 new country records, including species previously known only from Europe, Canada, and the western or southern United States.

Fifteen species are newly described: *Agromyzaechinalis* sp. nov., *Melanagromyzabrunkei* sp. nov., *M.eoflacensis* sp. nov., *M.glyptos* sp. nov., *M.rutella* sp. nov., *Ophiomyiacapitolia* sp. nov., *O.cuprea* sp. nov., *O.galiodes* sp. nov., *O.heleios* sp. nov., *O.kalia* sp. nov., *O.laticolis* sp. nov., Cerodontha (Poemyza) ungula sp. nov., *Phytomyzaavicursa* sp. nov., *P.catenula* sp. nov., *P.winkleri* sp. nov.

Four new species-level synonyms and one genus-level synonym are provided: *Agromyzamarmorensis* Spencer syn. nov. is included as a synonym of *A.aristata* Malloch; *Melanagromyzafastosa* Spencer syn. nov. is included as a junior synonym of *Ophiomyiatiliae* (Couden); *Melanagromyzaverbesinae* Spencer is considered a synonym of *M.vernoniana* Steyskal; *Phytomyzaranunculoides* Spencer syn. nov. is included as a junior synonym of *Phytomyzaloewii* Hendel; the genus *Liomycina* Enderlein syn. nov. is included as a junior synonym of *Phytobia* Lioy. *Ophiomyiaultima* (Spencer) comb. nov. is recombined from *Melanagromyza*. *Euhexomyzaalbicula* Spencer stat. reinst., comb. nov. is resurrected from synonymy with *E.winnemanae* (Malloch). New host records are provided.

The total number of known species in the Delmarva states is now 156, representing nearly 18% of the 879 species known from the Nearctic north of Mexico (Eiseman, pers. comm.). The Agromyzinae is better represented, with ~ 25% of known species, with *Agromyza* being especially well-represented at ~ 43% of known species. The Phytomyzinae fauna represents ~ 15% of known species, with *Aulagromyza* Enderlein (27.3%), *Calycomyza* (30.7%), *Nemorimyza* (100%) and *Phytobia* (41.0%) being best represented, and *Liriomyza* (8.9%) and *Phytomyza* (11.3%) being much more weakly represented.

Two genus-level groups occurring the Nearctic are not known from the Delmarva states as of yet. The first is the CerodonthaRondanisubgenusPhytagromyza Hendel, which mines in Poaceae. It is known from two western Nearctic species, C. (Ph.) frankensis Spencer (Fig. 100) and C. (Ph.) pusilla Zlobin, and one Palaearctic and eastern Canadian species, C. (Ph.) flavocingulata (Strobl). These species are keyed in Zlobin (1997). Secondly, species of *Pseudonapomyza* Hendel occur further to the north and west in North America, but discovery of this genus in the Delmarva states would not be surprising. *Pseudonapomyza* presently contains at least 100 species worldwide, almost entirely in the Old World, but there are likely dozens more (Černý 1992, 2008; Zlobin 2002a, 2005a; Černý and Zlobin 2008; Boucher 2004). Boucher (2004) last revised the New World fauna, recognising four species. All of these occur in the Nearctic, with *P.asiatica* Spencer extending into Guadeloupe and Venezuela.

Although the inspiration for this revision was Mathis and Foster’s (2007) revision of the Canacidae (Diptera) of the Delmarva states, and discussions with Wayne Mathis, the motivation came from curating the immense acalyptrate collection at the Museum of Natural History at the Smithsonian (USNM), which contains vast amounts of historical material from these states and district. Strangely, this collection was largely ignored by Spencer and Steyskal (1986b) during their revision of the Agromyzidae of the lower United States, using it mostly to examine type specimens designated by earlier researchers. Spanning more than a century of collection, the USNM is likely the largest repository of Nearctic Agromyzidae, and certainly the most extensive collection from the mid-Atlantic states, populated quite heavily through the efforts of C.H. Curran, K.E. Frick, S.W. Frost, G. Hevel, A.L. Melander, W. Steiner, G. Steyskal, and W.W. Wirth, among others. As the author wished to take advantage of this heavily sampled fauna, and to examine all agromyzid genera in a work of reasonable geographic scope for his first study of the family, a revision of the Delmarva fauna seemed appropriate. The author’s later work on Agromyzidae at the Canadian National Collection of Insects, Arachnids and Nematodes (CNC) provided much additional material complementary to this study, and specimens from that institution also predominate here.

**Table 1.** Total number of species for each genus of Agromyzidae in the Nearctic north of Mexico and the Delmarva states.

Subfamily	Genus	# Nearctic species north of Mexico	# species in Delmarva states
Agromyzinae	*Agromyza*	58	25
	*Euhexomyza*	6	1
	*Japanagromyza*	8	1
	*Melanagromyza*	80	16
	*Ophiomyia*	89	18
Phytomyzinae	*Amauromyza*	23	3
	*Aulagromyza*	11	3
	*Calycomyza*	39	12
	*Cerodontha*	73	17
	*Haplopeodes*	5	1
	*Liriomyza*	157	14
	*Metopomyza*	6	1
	*Nemorimyza*	2	2
	*Phytobia*	16	5
	*Phytoliriomyza*	27	6
	*Phytomyza*	275	31
	*Pseudonapomyza*	4	0
	TOTAL	879	156

## ﻿Natural history

The larvae of all Agromyzidae feed inside the living tissue of plants, with species reared from more than 140 families across the monocots, dicots, horsetails, ferns, and liverworts (Hering 1957, 1966; Spencer 1990; Scheffer et al. 2007). At least one species may also occur on a hornwort, with a parasitized puparium tentatively identified as *Phytomyza* recovered from *Nothocerusaenigmaticus* (Gilme, 2017). The non-vascular thallophytes and mosses are not known to be hosts, nor are the vascular gymnosperms with the exception of unsubstantiated *Phytobia* larval traces in fossil and extant tree trunks (Süss 1979), one record of *Tropicomyiaatomella* (Malloch) in leaves of *Gnetum* (Sasakawa and Pong 1988), and presumed leaf mines of *Liriomyzaschmidti* (Aldrich) on the cycad *Zamia* (Eiseman et al. 2019).

Feeding habits among taxa are diverse, with many species leaf-mining or boring inside stems, roots, twigs, and trunks, but others are known to mine in seeds, seed pods and flower heads, and species across a number of genera induce galls. Herbaceous annual plants are disproportionately affected by Agromyzidae compared to other similarly feeding phytophages such as the leaf-mining Lepidoptera, which dominate on perennials (Hespenheide 1991). Most species for which larval habits have been discovered are those that mine leaves because the mines produced are often readily observed, and the mines of some may be visible from a distance. Especially conspicuous mines can be produced by species such as *Calycomyzaflavinotum* (Frick), for example, where numerous larvae might mine across the entire surface of large *Arctium* leaves. Conversely, those species that remain hidden inside thicker, opaque structures are generally overlooked, and as such, many host relationships for these remain to be discovered. An exhaustive and invaluable compendium of these flies and their hosts was provided by Spencer (1990), with an updated list of host genera later provided by Benavent-Corai et al. (2005). Eiseman (2019) provides complete host species lists for all known leaf- and stem-mining species in North American, as well as records of observed mines of unknown agromyzid species.

Since plant hosts are still unknown for many species, and it is becoming apparent that many species once thought to be faithful to a single species or genus of host are actually more varied in their diet, it is not recommended that identifications be made using host alone. Adult male dissections or molecular sequence data are crucial for confident identification, and additional details of larval host usage, mine shape and frass pattern are also useful for contributing to a diagnosis (Eiseman and Lonsdale 2018). While some agromyzids may truly be restricted to a single plant host, it is likely that most can be generally characterised as oligophagous, being limited to multiple hosts in related families or genera. The apparent monophagy of many species will eventually prove to be an artifact of incomplete observations, with a growing body of data revealing that additional rearing records will almost certainly lead to novel host records for many species (Scheffer et al. 2021; Eiseman et al. 2018–2021). The extent to which this is true, however, can only be accurately characterised after more targeted taxonomic and rearing studies, and should prove to be a rich field of study.

Among the agromyzid genera, many lineages have successfully speciated on specific plant taxa, especially on diverse groups such as grasses (e.g., *Cerodontha*, *Metopomyza* Enderlein, the *Pseudonapomyzaatra* group, the *Agromyzabispinata* group, others) and herbaceous Asteraceae (e.g., at least 1/2 of non-*Cerodontha* phytomyzines). Within these more specialised groups, however, there appear to be periodic but major shifts to entirely different plant families or orders followed by bursts of speciation (Griffiths 1980; Scheffer and Wiegmann 2000). This was supported by Scheffer et al. (2007) at the genus level, who found host use to be quite varied, with at least ten genera occurring on both monocots and dicots, and non-angiosperm feeders derived from angiosperm-feeding stock.

True polyphagy is uncommon in the Agromyzidae, with only an estimated 16 species occurring on a wide diversity of host families (Spencer 1990). As would be expected, many of these occur on a variety of agriculturally significant crops and can sometimes be quite pestiferous and difficult to control, as populations easily accumulate on weeds in and around growing areas or alternate crops. They are also often readily dispersed to other countries through trade, hidden within plant tissue and potting soil. A number of these, especially those in the genus *Liriomyza*, appear to have originated in North and Central America, but other Old World species are similarly problematic.

Larval movement of agromyzid leaf miners within the host is characteristic and can be used to differentiate them from other leaf miners such as the microlepidoptera, as discussed in Eberle (1967) and Dempewolf (2005): the larva lies on its side and sweeps its body dorsally and ventrally to shave off successive rows of cells.

Mine location and shape, and the pattern by which frass is deposited in the mine, is often characteristic for a given species on a particular host during a certain part of the year, which may allow for tentative field identification (Nowakowski 1962; Dempewolf 2005; Lonsdale and Scheffer 2011). This is not true for all species, however, with similar mines of different species sometimes occurring on the same host plant or population of plants, which has been particularly problematic in the early diagnosis of some pest species. An especially thorough discussion on this topic is provided in Eiseman (2019). The main categories of leaf mine pattern are linear/serpentine and blotch, although there are many variations and irregularities, and a single larva may produce different mines over time, sometimes with later blotch mines obscuring the linear original. Leaf mines are usually also associated with specific parts of the leaf, such as the midrib, margin, lateral veins or even the petiole. The usage of specific cell layers also varies between species, but a single larva may not necessarily be restricted to a single layer over successive instars. Upon pupariation, the larva may remain in the mine or slightly removed from it within the leaf, it may first emerge and drop to the ground, or it may pupariate partially to entirely emerged from the mine, anchoring itself with enlarged hind spiracles, strong threads, or a hardened frass deposit. Seed and stem miners typically pupariate within the host where feeding occurs (Spencer 1987). A few species switch from leaf to stem mining, such as the pestiferous *Ophiomyiaphaseoli* (Tryon), or from stem to seed mining. If a species is gall-forming, the shape and location of the gall is characteristic (Dempewolf 2005). *Phytobia* species are found under the bark of trees where they feed on young xylem, tissue that in the past was incorrectly referred to as “cambium”, as discussed in Ylioja et al. (1998); their feeding produces characteristic “pith flecks” that are also called “medullary spots”.

Eggs are introduced into host tissue by female oviposition, with the modified terminal segments of the female abdomen specialised for creating a hole in the host and guiding the egg into the appropriate position among the cell layers. The female may produce similar additional punctures in the host to release sap on which both sexes feed (Ferrar 1987; Aukema et al. 1996). These feeding punctures may be abundant and cover the entire leaf surface, with some species such as *Phytomyzahorticola* (Goureau) producing up to 150 punctures per leaf, despite leaving only a few eggs behind (Hill 1986). In *Agromyzafrontella* (Rondani), pheromones left by the female around the egg insertion point, possibly a product of the digestive tract, reduces competition as an oviposition-deterrent (McNeil and Quiring 1983). The larva emerges from the egg after a few days, and feeding may last from a single week to ten months, dependant on the species, host and temperature (Spencer 1987; USDA 2008). Some species such as *Phytomyzailicis* Curtis feed well into the winter (Eber 2004). Species with multiple generations per year tend to produce more rapidly developing larvae, and overwintering larvae may arrest growth to resume activity when favourable conditions return (Spencer 1987). Several forms of conspecific competition have been observed for *A.frontella* on the host, including larval cannibalism, larvae pre-empting competitors by mining the leaf first, and adult feeding on a leaf to preclude larval mining (Quiring and McNeil 1984a–c).

Parasitoids are highly effective in controlling agromyzid population numbers, often with high mortality rates that are frequently discouraging to those attempting to rear the flies. Commonly encountered families are Eulophidae, Figitidae, Braconidae, and Pteromalidae, which emerge from the host puparium, but less commonly the larva. Eiseman (2019) has provided an overview of these parasitoids and their relationships for North American taxa. The preservation of these wasps in agricultural settings is essential, as they often suppress pests to levels not requiring additional intervention. In settings where pesticides are used, misapplication may easily affect wasp populations disproportionately, resulting in the collapse of these natural controls followed by pest outbreaks, unintentionally increasing damage, and reducing crop yield and value (Weintraub and Horowitz 1995; Hidrayani et al. 2005).

## ﻿Classification of Agromyzidae

### ﻿Family-level relationships

While it is relatively simple to diagnose the family, the assignment of derived or synapomorphic states is problematic due to the uncertainty involved with establishing outgroups, and there is little consensus on sister group relationships. Minor similarities in wing venation, chaetotaxy and genitalia have historically allied the Agromyzidae to other acalyptrates, but these simple characters are of equivocal polarity and can be easily interpreted as plesiomorphic.

The Agromyzidae is currently treated as a member of the Opomyzoidea (McAlpine 1989), at least as a close relative of the Clusiidae (Griffiths 1972b; Frick 1952a; Spencer 1987, 1990), but it is becoming increasingly evident that this superfamily is a “dumping ground” for unplaced families of more generalised morphology, at least in part. As an example, the monogeneric Acartophthalmidae was treated as sister to Clusiidae in McAlpine’s (1989) Clusioinea on the basis of a generalised habitus and a handful of putative synapomorphies (an angulate extension on the pedicel is the only character of note and this has been independently derived in other Acalyptratae). Acartophthalmidae has recently been supported as Carnoidea using quantitative phylogenetic methods, subsequently removing it from the Opomyzoidea (Buck 2006). Most recently, Winkler et al.’s (2010) molecular study of the Opomyzoidea provided little evidence for any family-level relationships, with the exception of weak support for Agromyzidae + Neurochaetidae, a subset of periscelid genera within an expanded Aulacigastridae, and relatively strong support for Xenasteiidae + Australimyzidae (Carnoidea). There are additional other relationships among the Asteoinea supported to varying degrees by different character subsets (a summary of these is provided in Winkler et al. 2010), but the homoplasious nature of these characters and the degree to which they have been reinterpreted in the literature precludes any confident statements about common ancestry.

In an effort to identify any possible synapomorphic morphological characters shared by the opomyzoid families, the author examined representatives of all extant genera, excluding some asteiid, odiniid, teratomyzid and Afrotropical taxa; additional data on Palaearctic Anthomyzidae were taken from Roháček (2006). Except for the putatively ancestral characters of chaetotaxy and wing venation that characterise most Opomyzoidea, almost no additional external or genitalic characters were found that would serve to support relationships between these families, with two major exceptions.

The first of these is Anthomyzidae + Opomyzidae, the monophyly of which received modest support in Roháček (2006). This contrasts uncertain results regarding these two families in Winkler et al. (2010), however, and there are possible conflicting characters of the female genitalia (Kotrba and Baptista 2002). The second is an association between the Agromyzidae and the predominantly Australian Fergusoninidae, which is supported by a relatively substantial number of female genitalic and larval characters. Fergusoninidae is a similarly phytophagous family of small-bodied species that was at one time even considered to be Agromyzidae (Malloch 1924b). However, placement of this family in the superfamily Nerioidea was well-supported by Lonsdale (2020) in a quantitative analysis of the nerioid and diopsoid families using morphological data sets (external, male genitalic and female genitalic) and numerous outgroup families, including Agromyzidae. Putative similarities to Agromyzidae were supported as superficial and there is much disagreement in the structure of the male genitalia. The molecular analyses of Bayless et al. (2021) provide conflicting placement of Fergusoninidae, again suggesting closer association to Agromyzidae in a “cleft pedicel clade”. In their preferred phylogeny, Agromyzidae + Odiniidae is sister to Fergusoninidae + Carnidae, which still supports the similar female and larval characters of Agromyzidae and Fergusoninidae as convergent.

### ﻿Putative family synapomorphies

In his discussion of the family, McAlpine (1989) listed a number of derived characters to define the Agromyzidae: the larvae feed in living plant tissue with an associated adaptation of the mouthparts, with “mandibles toothed and angularly positioned in relation to hypopharyngeal sclerite and hypopharyngeal and tentoropharyngeal sclerites fused” (Figs 29–31); the anterior larval spiracles lie close together dorsally on the thorax (visible in puparium, Fig. 27); the cells connected to the larval fat bodies contain calcium carbonate crystals; tergite 6 and sternite 5 are enlarged in the male; the male pregenital sclerites (sternites 6–8) are reduced, usually forming a small symmetrical dorsal band; the phallus is “extremely complex” and [at least ancestrally] contains two gonopores; female segment 7 forms a large oviscape with an anterodorsal apodeme (Figs 11–13); the membrane between female segments 7 and 8 is densely covered with anteriorly directed denticles (Fig. 19); female segment 8 is modified into one pair of serrated egg-guides; the female cercus has a group of apical trichoid sensilla (Figs 16–18).

Additional external adult characters added here include a relatively large and subshiny ocellar triangle surrounding the ocellar tubercle (Fig. 78) (secondarily reduced or obscured in several lineages, especially Phytomyzinae), and possibly the presence of only one pair of lateral scutellar setae (absent in some *Cerodontha*). Elaborating on the structure of the internal components of the genitalia (Figs 139–142): the pregonite is absent; the distiphallus is discrete and capsule-like with basal insertion of the ejaculatory duct (often with basal differentiation of the segment to produce a “mesophallus”); the basiphallus is segmented basally to produce a separate cylindrical structure termed the “phallophorus”; the basiphallus is distally composed of one pair of long lateral plates flanking the ejaculatory duct; the epiphallus is a segmented, folding structure beneath the phallophorus.

### ﻿Generic classification

The genera of Agromyzidae have traditionally been divided between two subfamilies, the Agromyzinae and the Phytomyzinae, which follows the same system originally devised by Fallén (1823a, b) for his “agromyzides” and “phytomyzides”. This system has recently been corroborated using morphological (Dempewolf 2001) and molecular (Scheffer et al. 2007) data. The adults of Phytomyzinae are characterised by plesiomorphic wing venation similar to that of other Opomyzoidea: the subcostal vein is either incomplete distally or it reaches the costa as a thin fold that remains separate from R1; R1 itself is sometimes slightly expanded apically, but it is almost always narrow and straight (Fig. 3). Conversely, the subcosta is fused with R1 distally in the Agromyzinae and the apex of R1 is almost always expanded and sinuate (Fig. 4). This venation is relatively consistent within subfamilies, although a number of *Phytobia* (Phytomyzinae) have converged upon the state otherwise typical of the Agromyzinae, and some *Agromyza* Fallén have the derived character appearing very subtly. Regarding larval morphology, the Phytomyzinae (Figs 24, 25, 30, 31) is characterised by a reduced lower arm on the cephalopharyngeal skeleton while the Agromyzinae (Fig. 29) is characterised by two strong arms (Spencer 1987). A variably developed medial longitudinal sclerotised band on the ejaculatory apodeme is here also found throughout the Agromyzinae (Fig. 141), although it only frequently appears in species of the *Ophiomyia* genus group, being indistinct or secondarily lost in many species of *Agromyza* and *Japanagromyza* Sasakawa.

Since inconsistencies in the subfamilies’ defining characters were brought to light by von Tschirnhaus (1971) it has been speculated that the lineages of Agromyzidae cannot be forced into two distinct halves. As a solution, Spencer (1990) provided an alternate system of classification that encompasses four separate genus groups: the *Penetagromyza* group, which is equivalent to Agromyzinae, and the *Napomyza*, *Phytobia* and *Phytoliriomyza* groups, which together are equivalent to the Phytomyzinae. Little to no evidence was provided in support of these groups or the relationships within them, and their use in classification here appears to be limited, but they are nonetheless mentioned below as they relate to generic relationships.

### ﻿Subfamily Agromyzinae

Although there are a number of highly characteristic lineages within Agromyzinae, most of the subfamily has relatively non-descript and uniform colouration, venation and often chaetotaxy. To compound this, species are often described on the basis of slight morphometric changes in the male genitalia and subtle variations in external morphology, making species identification problematic.

Agromyzinae can be divided into three relatively distinct clades. The first is the *Ophiomyia* genus group, an aggregate of morphologically similar and frequently confused genera that includes *Ophiomyia* Braschnikov (Figs 64–83), *Melanagromyza* (Figs 52–63), *Euhexomyza* Lonsdale (Figs 44–48) and the Old World *Tropicomyia* Spencer. Lonsdale (2014) re-characterised this group and its genera, and aside from sharing a generally similar habitus, he found species to have a large, bulging lunule, insertion of the antenna below the midpoint of the head, brown halters (with very few exceptions) and no prescutellar acrostichal setae (also absent in some *Japanagromyza*). Internally, the subepandrial sclerite is divided into two well-sclerotised plates that almost meet at a point medially, the inner lobe of the hypandrium has partially separated into an arched, band-like sclerite, the postgonite is sometimes reduced to absent, modified or fused to other structures, and the mesophallus is shifted to form a small distoventral lobe on the distiphallus (with some exceptions). Species are mostly internal borers, with leaf mining largely restricted to *Tropicomyia*. *Tropicomyia* is a small genus of minute, brown leaf mining species with a disproportionate number of polyphages. While the genus is likely not monophyletic and in need of revision (Lonsdale 2014), most species appear to belong to a core lineage that produces irregular mines in the upper epidermal layer that appear “silvery” (Spencer 1973).

The second clade is made up of the genus *Agromyza* (Figs 37–43), which is often paler and more slender compared to other Agromyzinae, and it is best diagnosed by the presence of a distinct saw-like stridulatory mechanism along the fused lateral margins of the first and second tergites in both sexes (Fig. 38). All species also have one pair of prescutellar acrostichal setae and three or more dorsocentral setae (Fig. 10), although a number of grass-feeding species have only two well-developed pairs.

The last clade is composed solely of *Japanagromyza* (Figs 49–51), which was redefined by Lonsdale (2013) in his revision of the African species. The genus shares a combination of features characteristic of both *Agromyza* and the *Ophiomyia* genus group, leading some authors to treat it as an “intermediate” of the two. Like *Agromyza*, it often has prescutellar acrostichal setae and the mesophallus is basal to the distiphallus (not ventral). *Japanagromyza* and a minority of *Agromyza* also have an inner-distal comb of setae on the hind tibia (von Tschirnhaus 1991). Like the *Ophiomyia* genus group, *Japanagromyza* lacks the stridulatory file on the abdomen, it is quite dark in colour and sometimes iridescent, and it has a reduced number of dorsocentrals. Furthermore, while most species leaf-mine like *Agromyza*, Sasakawa (2010) revealed that the genus presented a broader range of habits otherwise characteristic of the *Ophiomyia* genus group. Based on the relatively high degree of variation in these characters, and because of the unusual diversity in genitalic features seen across *Japanagromyza* species globally, it seems unlikely that the genus is monophyletic (Lonsdale 2013).

### ﻿Subfamily Phytomyzinae

The Phytomyzinae represents a comparatively heterogenous group that varies widely in external and male genitalic morphology, contrasting the often uniform species of Agromyzinae. A genus-level phylogeny of the subfamily was produced by Dempewolf (2001), but portions of his proposed relationships were challenged by Scheffer et al. (2007) and still remain partially unresolved. Despite this, several groups of genera appear to be moderately well-supported.

The first of these is what Spencer (1990) called the “*Napomyza* group”, and it is largely comprised of the diverse genus *Phytomyza* (Figs 131–138), which is characterised by proclinate orbital setulae in combination with a costa that extends to vein R_4+5_, M1 is weak or spectral, and crossvein dm-m is absent or situated basally. There is also slight to strong costalisation of the wing veins. The classification of this genus was recently advanced by Winkler et al. (2009), who provided the most robust quantitative analysis of *Phytomyza* to date. Winkler et al. (2009) maintained *Napomyza* and *Ptochomyza* Hering as subgenera of *Phytomyza* because they are readily diagnosable, they were both supported as monophyletic, and were both recovered near the base of *Phytomyza* sensu lato. Winkler et al. (2009) also re-synonymised *Chromatomyia* Hardy with *Phytomyza*, supporting the findings of a number of historical studies. An argument for retention of *Chromatomyia* was proposed by von Tschirnhaus (2021), but reanalysis of the evidence by Lonsdale and Eiseman (2021) revealed that synonymy of the genus was well-founded and should be maintained for a number of reasons: *Chromatomyia* renders *Phytomyza* paraphyletic, it is unlikely to be monophyletic itself (although proof is not definitive), larval characters in support of the genus are homoplastic, and male genitalic characters supporting the genus are impractical for use, frequently misdiagnosed or misinterpreted, and also possibly homoplastic.

Other genera in the *Napomyza* group are poorly represented or unknown in the New World. These resemble *Phytomyza* in having the costa extend to R_4+5_, and many species also exhibit costalisation of the wing veins, but the orbital setulae are erect to reclinate or absent. The smallest of these genera is *Gymnophytomyza* Hendel, which has two Palaearctic species that were treated by Zlobin (1999); species lack the proepisternal seta and there are records of leaf mining in *Galium*. *Aulagromyza* and *Pseudonapomyza* are differentiated by characters of wing venation and their monophyly is yet to be confidently established. Most *Aulagromyza* are dark-bodied species (Fig. 88), but the *A.populicola* group (Fig. 89) is made up of bright yellow species that feed on Salicaceae and may not be related, as discussed by Zlobin (2007c) and Winkler et al. (2009). *Pseudonapomyza* is divided into two groups, the most abundant and widespread of which is the *P.atra* group (Zlobin 2002a) (Figs 130, 833–839), which has an angulate first flagellomere (with at least one exception), the male mesophallus and distiphallus are distinctive, and larvae feed on Poaceae (Zlobin 2002a). The *P.acanthacearum* group may be paraphyletic (Zlobin 2002a), and it is restricted to Old World tropical regions, where species feed on Acanthaceae, Amaranthaceae, Asteraceae (Černý 2008) and Bignoniaceae (Madire et al. 2010).

The remaining Phytomyzinae were considered by Spencer (1990) to belong to either the “*Phytoliriomyza* group” or the “*Phytobia* group”. There is no strong morphological or molecular support for the *Phytoliriomyza* group (Scheffer et al. 2007), but a subset of six genera with tubercle-like setae on the posterodistal margin of the epandrium and surstylus is possibly monophyletic: *Calycomyza* (Figs 90–98), *Haplopeodes* Steyskal (Fig. 120), *Liriomyza* (Figs 107–117), *Metopomyza* (Figs 118, 119), *Phytoliriomyza* Hendel (Figs 126–129) and *Selachops*. These genera were recovered together in Scheffer et al. (2007), but not in Dempewolf (2001), although results in the latter were discussed as possibly being attributable to the inclusion of homoplastic features. Another likely monophyletic lineage within this group is *Liriomyza*, *Haplopeodes* and some *Phytoliriomyza*, where the number of tubercles on epandrium and surstylus is reduced to one, although these structures are sometimes secondarily lost, modified or duplicated. In *Metopomyza* and the remaining *Phytoliriomyza* there is usually a discrete comb or otherwise ordered arrangement of tubercles, contrasting the dense, scattered tubercles seen in *Calycomyza*, *Selachops* and the unusual *L.quadrisetosa* (Malloch) (Figs 616–620). Only the small Palaearctic *Selachops* is not known in the Nearctic (Zlobin 1984); the host of *S.flavocinctus* Wahlberg was recently revealed to be *Carexacuta* (Cyperaceae) (Kostromina et al. 2016).

Within this group characterised by tubercle-like setae on the external male terminalia, there is gathering consensus that at least some genera are artificial groupings (Dempewolf 2001; Scheffer et al. 2007). As such, there is a strong need for boundaries to be re-evaluated so that genera can be reconstructed as entities that are both monophyletic and easily diagnosable using external characters. For example, if it is supported that *Phytoliriomyza* is indeed paraphyletic, it will have to be divided into at least two groups if other genera such as *Metopomyza* and *Liriomyza* are to be retained. However, while some lineages of *Phytoliriomyza* can be diagnosed externally (such as that with an entirely black first flagellomere and that with an apically brown halter and setose eyes), others are inseparable from species in related genera without male dissection, reducing potential generic utility. Dissection is currently also necessary for confident diagnosis of *Haplopeodes* and most *Liriomyza*, given that characters of colour, chaetotaxy and wing venation are overlapping and frequently convergent. Some progress has been made to provide natural boundaries for *Liriomyza*, such as including *Galiomyza* Spencer as a junior synonym, but consistently useful external diagnostic characters are still lacking and much additional work needs to be done (Lonsdale 2017). Lonsdale (2017) discussed the polyphyly and variant morphology of *Galiomyza* species in detail before synonymizing it, but Papp and Černý (2017) resurrected it soon thereafter, unusually noting that they preferred to retain it in order to avoid reorganising their as yet unpublished chapter on it, which was to appear in print soon after the synonymy appeared; an unclarified statement on the monophyly of *Galiomyza* was also mentioned. It is recommended that the synonymy of *Galiomyza* be retained until scientific evidence is provided to the contrary. The position of the New World genus *Haplopeodes* is uncertain, although some have proposed it to be an offshoot of *Liriomyza* (Spencer 1990) or at least a close relative of it (Steyskal 1980). Determining *Haplopeodes*’ ancestry has proven to be difficult, however, due to an extreme reduction in size and other features that may have been phylogenetically informative, especially those of the male genitalia.

The positions of the other phytomyzine genera have not been confidently resolved and their relationships remain uncertain (Dempewolf 2001; Scheffer et al. 2007). Molecular data weakly supports *Cerodontha* (Figs 99–106) as sister to the rest of the Phytomyzinae, and there is variable support for a lineage encompassing those genera without tubercles on the surstylus and epandrium, including those in the “*Napomyza* group”. Of these genera, *Amauromyza* Hendel (Figs 84–87), *Nemorimyza* (Figs 121–123) and *Phytobia* (Figs 124, 125) belong to what Spencer (1990) called the “*Phytobia* group”. Spencer (1990) considered these genera to be basal in the family because they share a large body size and a supposedly plesiomorphic feeding mode (Nowakowski 1964; Spencer 1990), that is, stem-boring, even though all *Nemorimyza* and some *Amauromyza* are leaf-miners. Both of these historically held assumptions of evolutionary progression were found to be erroneous by Scheffer et al. (2007), who instead supported leaf mining as the ancestral state.

The status of two genera placed by Spencer (1990) in the *Phytoliriomyza* group is uncertain. Genitalic illustrations of *Xeniomyza* Meijere in Spencer (1990: figs 237, 238) strongly suggest that the genus should be synonymized with *Liriomyza*. The type species is known from Amaranthaceae (Spencer 1990), and the two Palaearctic species are characterised by loss of dm-m and costalisation of the wing veins (Zlobin 1979). The monotypic *Pseudoliriomyza* Spencer (host unknown) is known from Tanzania and Indonesia (Spencer 1966d) and has some similarity to *Liriomyza*, but additional specimens are required for further study. The genus is characterised by loss of vein dm-m, the fourth dorsocentral, the surstylus and the epandrial spine, and there are some genitalic similarities to *Amauromyza* in the structure of the hypandrium and the ejaculatory apodeme.

## ﻿Materials and methods

The majority of material examined for this study is deposited in the Smithsonian Institution, National Museum of Natural History, Washington, D.C., USA (**USNM**), and the Canadian National Collection of Insects, Arachnids and Nematodes, Ottawa, Canada (**CNC**). Additional material was examined from, or is deposited in the following institutions:

**BLTJ** Biological Laboratory, Yazu High School, Tottori Pref., Japan;

**BPBM** Bernice P. Bishop Museum, Honolulu, Hawaii, USA;

**CASC** California Academy of Sciences, San Francisco, California, USA;

**CFS** Canadian Forest Service, Edmonton, Alberta, Canada;

**CSCA** California State Collection of Arthropods California, USA;

**CUIC** Cornell University, Ithaca, New York, USA;

**DEBU** University of Guelph Insect Collection, Guelph, Ontario, Canada;

**INHS** Illinois Natural History Survey, Champaign, Illinois, USA;

**MCZ** Museum of Comparative Zoology, Cambridge, Massachusetts, USA;

**MNHN** Museum National D’Histoire Naturelle, Paris, France;

**NHMUK** The Natural History Museum, London, United Kingdom;

**NMW** Naturhistorisches Museum Wien, Vienna, Austria;

**NRS** Naturhistoriska riksmuseet, Stockholm, Sweden;

**OUMNH** Oxford University Museum of Natural History, Oxford, United Kingdom;

**RNH** Rijksmuseum van Natuurlijke Historie, Leiden, Netherlands;

**SMNS** Staatliches Museum für Naturkunde, Stuttgart, Germany;

**UDCC** University of Delaware, Newark, Delaware, USA;

**UZMH** Finnish Museum of Natural History, University of Helsinki, Finland;

**VPIC** Virginia Tech, Blacksburg, Virginia, USA;

**WVDA** West Virginia Department of Agriculture, Charleston, West Virginia, USA;

**ZIL** Russian Academy of Sciences, Zoological Institute, St. Petersburg, Russia;

**ZMHU** Museum für Naturkunde der Humboldt-Universität, Berlin, Germany;

**ZMUC** Zoological Museum, University of Copenhagen, Denmark;

**ZMUN** Zoological Museum, University of Oslo, Norway.

For the following keys and species descriptions, an emphasis was placed on adult description and diagnosis, especially with regards to the highly derived components of the male terminalia, as the majority of material examined was collected as adults with little or no associated host or habitat data. Host genera are provided only, with plant species listed when only a few are known. While external morphology is of course also useful, findings strongly support Nowakowski (1962), who noted that the external characters usually used to separate closely related agromyzid species, such as wing vein ratios, body size, fronto-orbital number, and subtle colour characters, are often of “negligible taxonomic value”. Many of these external characters were here found to vary subtly to widely within species previously thought to be morphologically conserved. This is of particular significance considering the frequent use of these characters in historical identification keys, which often only provide a single character at each couplet.

Adult terminology and definitions are outlined below in the family description. Terminology for external structures largely following Cumming and Wood (2017), except where noted. Genitalic terminology mostly follows Lonsdale (2020), which expands on Cumming and Wood (2017) by discussing structures of the terminalia peculiar to families of Acalyptratae that are not otherwise considered in the earlier work. A few deviations are made here for structures of the hypandrial complex specific to the Agromyzidae, wherein the terminology presented by Steyskal (1969) is provided: specifically, the phallophorus, proepiphallus, and metepiphallus. Gena height is measured vertically at the lowest point of the eye. Dorsocentral setae are numbered starting from the posterior-most seta because setae number often varies anteriorly. Rows of acrostichal setulae are counted at the transverse suture. New host records, and new country/province/state records are indicated by an asterisk.

Most specimens are pinned, and either air-dried or prepared in a critical-point drier. Some voucher material collected by D. Smith or S. Scheffer, excluding most specimens designated as types, was retained in 95% ethanol for potential future study. Abdomens of dissected specimens were initially prepared by dissolving the soft tissue in hot potassium hydroxide, followed by washing in demineralised or deionised water, neutralisation in glacial acetic acid, and further washing, but later dissections used hot lactic acid exclusively. Thorough washing and neutralisation of the potassium hydroxide still leave minute traces of the chemical, which continue to dissolve pigment and weaken sclerotised tissue, and it is not recommended for use. Genitalia were stored in glycerine in microvials pinned with the specimen. Most illustrations were prepared by the author, excluding several figures scanned from the literature, which are indicated in the text and in the acknowledgements. The sizes of illustrated structures are shown in correct proportion to each other, with the epandrium reduced due to space constraints; some structures were not illustrated if they were damaged, could not be found (ejaculatory apodeme), or were uninformative for diagnosis within the genus.

## ﻿Results

### ﻿Agromyzidae – adult diagnosis

**Figures 1–6.**
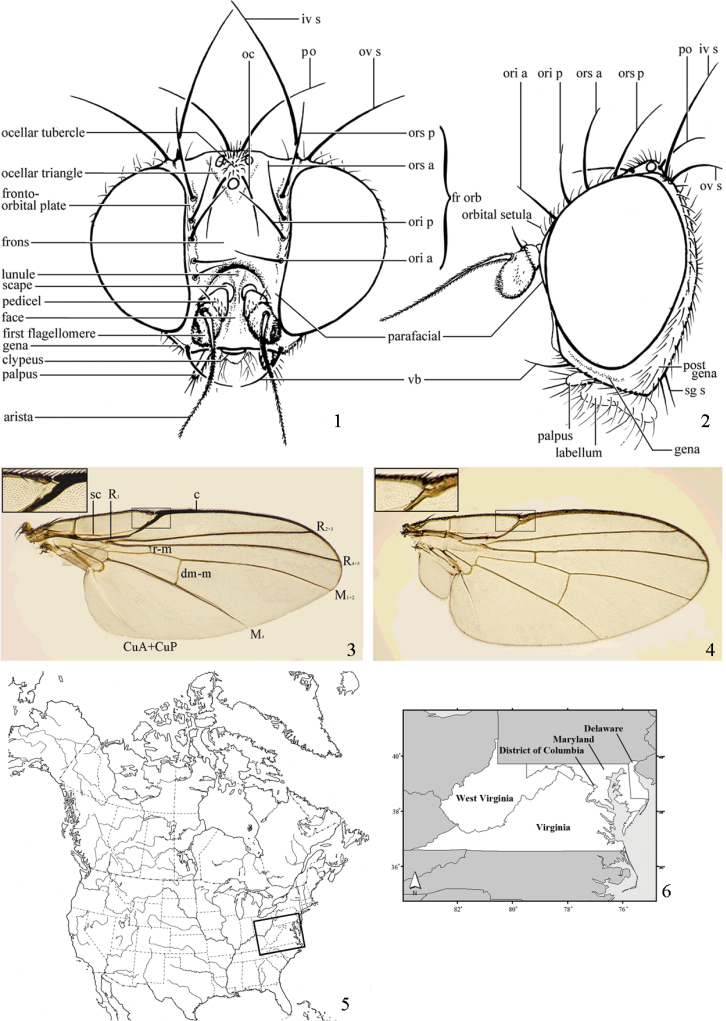
*Agromyzacanadensis* Malloch, female head **1** anterior **2** lateral **3***Phytobiasetosa* (Loew), wing, with detail of junction of R_1_ and costa **4***Melanagromyzavirens* (Loew), wing, with detail of junction of R_1_ and costa **5** political map of the United States and Canada **6** detail of the Delmarva states – Delaware, Maryland, Virginia and West Virginia, and the District of Columbia. Abbreviations: **c**, costal vein; **fr orb**, fronto-orbital setae; **iv s**- inner vertical seta; **oc**, ocellar seta; **ori a**, anterior inferior orbital seta; **ori p**, posterior inferior orbital seta; **ors a**, anterior superior orbital seta; **ors p**, posterior superior orbital seta; **ov s**, outer vertical seta; **po**, postocellar seta; **sc**, subcostal vein; **vb**, vibrissa.

**Figures 7–11.**
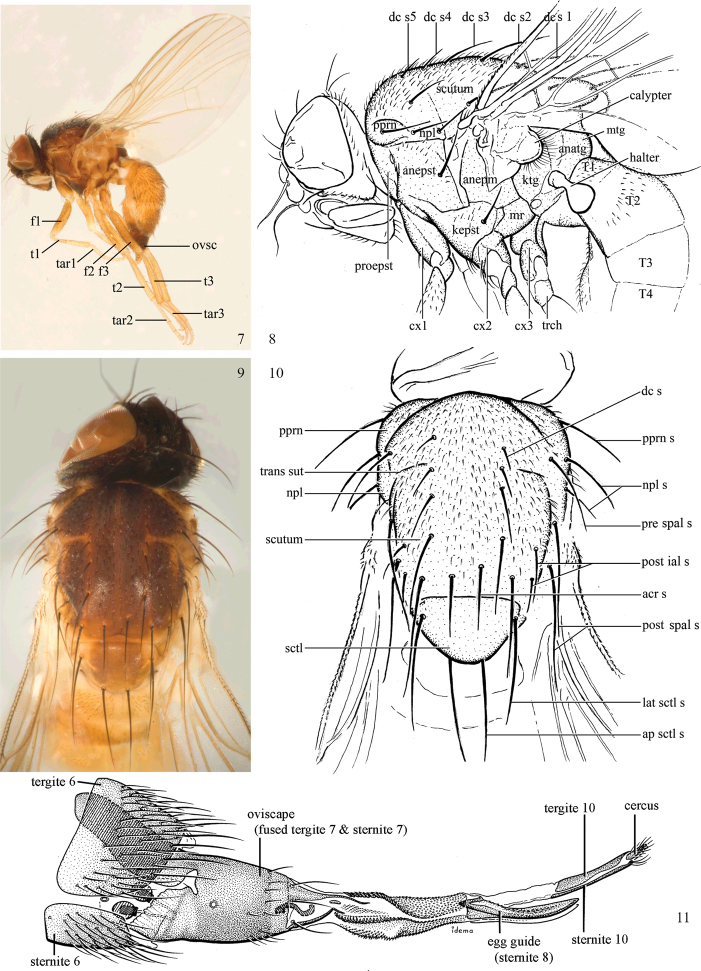
*Agromyzacanadensis* Malloch, female **7** lateral **8** detail of thorax, lateral **9** dorsal **10** detail of thorax, dorsal **11***Agromyzapseudoreptans* Nowakowski, extended female postabdomen, lateral (fig. 20 from Spencer 1987). Abbreviations: **1**, fore; **2**, mid; **3**, hind; **anatg**, anatergite; **anepm**, anepimeron; **anepst**, anepisternum; **ap sctl s**, apical scutellar seta; **cx**, coxa; **dc s**, dorsocentral seta (numbered from back); **f**, femur; **kepst**, katepisternum; **ktg**, katatergite; **lat sctl s**, lateral scutellar seta; **mr**, meron; **mtg**, mediotergite; **npl**, notopleuron; **npl s**, notopleural seta; **ovsc**, oviscape (fused tergite and sternite 7); **pre spal s**, presutural supra-alar seta; **post ial s**, postsutural intra-alar seta; **post spal s**, postsutural supra-alar seta; **pprn**, postpronotum; **pprn s**, postpronotal seta; **proepst**, proepisternum; **sctl**, scutellum; **t**, tibia; **T**, tergite; **tar**, tarsus; **trch**, trochanter.

**Figures 12–23.**
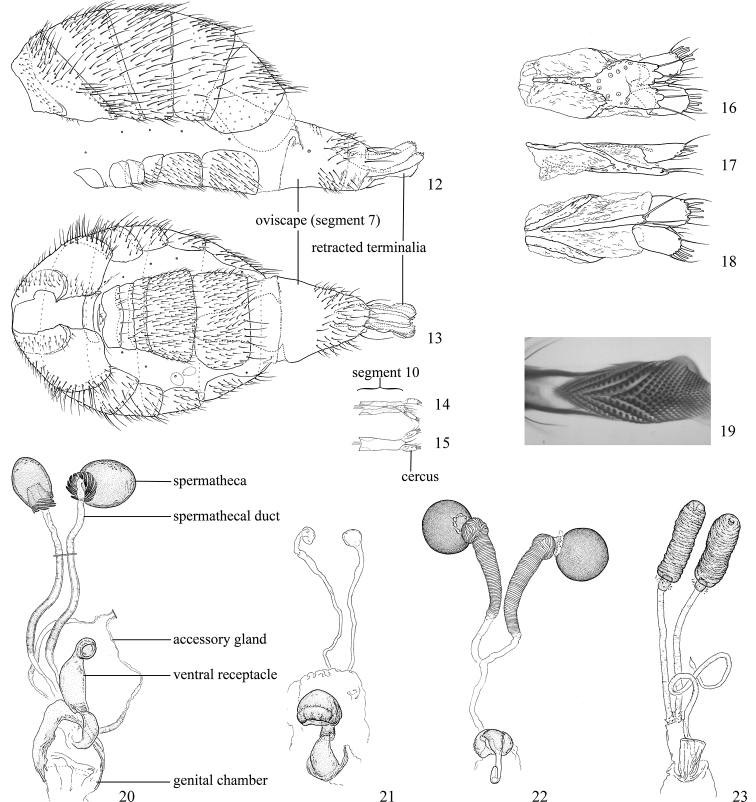
*Melanagromyzametallica* (Thomson), female abdomen (with terminalia retracted) **12** lateral **13** same, ventral **14** same, segment 10 to cercus, ventral **15** same, dorsal **16–18***Phytomyzahorticola* Goureau female, segment 10 to cercus **16** ventral **17** same, lateral **18** same, dorsal **19***Phytomyzamelampyri* Hering female, photo of denticles on membrane anterior to segment 8 **20–23** female internal genitalia **20***Melanagromyzametallica***21***Phytomyzahorticola***22***Liriomyzasativae* Blanchard **23***Agromyza* sp. (Uganda), female internal genitalia.

Adults generally small-bodied, wing length usually 1.5–3.5 mm, but as small as 0.5 mm and as large as 6.5 mm. Postocellar setae diverging. Frons usually with inclinate anterior and reclinate posterior fronto-orbitals. Orbital setulae usually present. Vibrissa present. Arista dorsobasal (Figs 1, 2). Ocellar tubercle surrounded by often large and subshiny ocellar triangle (usually less distinct than in Chloropidae) (Fig. 78), but sometimes reduced or with triangle absent. Lunule distinct, sometimes large. Scutellum with one pair of apical setae and almost always with one pair of well-developed laterobasal setae. Costa with break at end of subcostal vein, which is complete or nearly complete (Figs 3, 4), but sometimes fused to R1 if that vein is apically sinuous; humeral break absent. Vein CuA2 present, enclosing anal cell. Dorsoapical tibial setae absent. Basiphallus segmented into basal phallophorus and usually one pair of distal plates flanking duct. Distiphallus sometimes segmented to produce basal mesophallus. Female with oviscape distinct, dark, and heavily sclerotised (Figs 7, 11–13); membrane between female segments 7 and 8 densely covered with denticles; sternite 8 modified into one pair of serrated egg-guides.

A number of other small-bodied acalyptrate families are similar to Agromyzidae, but differ as follows. Chloropidae can be similarly small-bodied and stout with a large ocellar triangle, but this family lacks the anal cell and oviscape, and has a sharp propleural carina. Anthomyzidae are slender and sometimes pale with a vibrissa and larger ocellar triangle, superficially similar to some genera such as *Cerodontha*, and vein R1 is sinuous apically, similar to Agromyzinae, but in this family, a ctenidial spine is almost always present on the fore femur, the anepisternum is usually bare (with at least one large seta in Agromyzidae), the subcostal vein is clearly fused to R1 apically, and there is no stout oviscape. Odiniidae and Clusiidae have similar chaetotaxy, but preapical tibial setae are usually present, the large oviscape is absent, and Odiniidae have an incomplete subcosta; some Clusiidae do not have these tibial setae, but all species have a projection on the outer- and sometimes inner-distal margin of the pedicel. Other taxa with a large, conspicuous oviscape and complete subcosta, such as Tephritoidea, usually have strongly patterned wings and lack a vibrissa; Fergusoninidae are bright yellow with an anteriorly flattened head with small antennae concealed in deep recesses, a small face, and a very large parafacial and lunule.

### ﻿Agromyzidae – adult description

*General*: Wing length small, usually 1.5–3.5 mm (sometimes as small or as large as 0.5 mm and 6.5 mm). Colour varying from almost entirely black or brown to yellow, but many with some degree of pattern that may be more of less typical of a genus (for example, species of *Liriomyza* and *Calycomyza* are more conservatively patterned than *Phytomyza* and *Phytoliriomyza*); dark species of Agromyzinae sometimes with metallic shine (many *Melanagromyza* and some *Ophiomyia* and *Japanagromyza*); often subshiny to heavily brown or grey pruinose, uncommonly glossy.

*Head*: (Figs 1, 2) Frons with frontal vitta usually soft, but sometimes better sclerotised, sometimes with several setulae bordering lunule (common in *Agromyza* and *Phytobia*); fronto-orbital plate (fused frontal and orbital plates) and variably sized ocellar triangle either indistinct from vitta, more sclerotised, or delimited by difference in texture; ocellar triangle variably sized, sometimes reaching lunule, sometimes reduced and restricted to small, raised ocellar tubercle bearing ocelli. Lunule usually distinct; small soft and sunken, to large heavily sclerotised and bulging. Antenna porrect, first flagellomere rarely angled ventrally; arista subbasal, usually pubescent to bare, rarely with rays more elongate. Eye upright to distinctly oblique; bare to microtrichose. If parafacial continues under eye onto gena, band-like region referred to as “cheek”, and remaining ventral region is referred to as “jowl” (“subgena” in Cumming and Wood 2017). Face shallowly concave, often with broad lateral depressions to receive antennae, sometimes with medial carina that may have a central swelling or “bulb”. Gena usually straight or angled posteriorly, uncommonly deeper or produced anteriorly (many *Ophiomyia* with gena strongly projecting). Clypeus broad and U-shaped, posteromedial margin sometimes emarginate and anterior margin sometimes truncated (*Ophiomyia*). Proboscis normal, uncommonly elongate; palpus normally narrow, small, and cylindrical, rarely enlarged or swollen.

*Chaetotaxy*: In remaining consistent with names occurring in most agromyzid publications, terminology here largely follows that outlined in McAlpine (1981) in the Manual of Nearctic Diptera, with the fronto-orbital setae (usually four pairs, rarely less than three, sometimes five or more) divided into two classes: the inclinate anterior fronto-orbitals are treated as the inferior orbital setae (ori), and the reclinate/slightly inclinate posterior fronto-orbitals are treated as the superior orbital setae (ors); the two sets are most distinct when viewed anteriorly, and are sometimes difficult to differentiate. Pedicel small, margins shallowly rounded, with one dominant dorsomedial seta. Postocellar setae (one pair) divergent, arising from immediately behind ocellar tubercle. Ocellar setae (one pair) divergent and arising from ocellar tubercle at, or behind level of anterior ocellus. Vibrissae (one pair) distinct; multiple vibrissae sometimes present and fused into pointed “fasciculus” or “horn” in male (some *Ophiomyia*). Orbital setulae usually in one or two rows, sometimes in several rows, rarely absent, reclinate to proclinate, sometimes inclinate or exclinate; mostly or entirely arising from orbital plate lateral to fronto-orbital setae. Two pairs of divergent vertical setae arising from orbital plate near posterior margin of frons, divided into inner vertical seta and outer vertical seta.

*Thorax*: Sclerites labelled in Figures 8, 10, typical of adult Schizophora.

Chaetotaxy: (Figs 8, 10) One postpronotal seta. Usually two notopleural setae, uncommonly one. One presutural and two postsutural supra-alar setae (sometimes mistakenly termed the intra-alar); one or two postsutural intra-alar setae (sometimes mistakenly termed the intrapostalar, but these may be distinguished in other families of Diptera as illustrated in Cumming and Wood (2017: figs 41, 42) by being on a discrete swelling in the extreme posterolateral corner of the scutum, the “postalar callus”, which is absent in Agromyzidae; default designation of the posterior-most intra-alar as the post-alar is not justified). Usually two to four dorsocentral setae (fourth seta near or anterior to transverse suture), with additional setae sometimes extending presuturally. One pair of prescutellar acrostichal setae present or absent; acrostichal setulae usually present in 2–10 rows (measured at level of transverse suture), but sometimes absent, becoming sparser and more irregular posteriorly, disappearing at or behind level of posterior dorsocentral. Scutellum with one pair of apical setae and usually one pair of anterolateral setae (absent in two subgenera of *Cerodontha*); scutellum otherwise bare, but Palaearctic *Selachops* with dorsum densely setose. One strong, upcurved proepisternal seta (absent in *Gymnophytomyza*). At least one strong anepisternal and katepisternal seta, but sometimes with additional smaller setae posteromarginally on anepisternum and mediodorsally on katepisternum (mostly Agromyzinae).

*Legs*: Short, slender, fore and mid legs not widely separated. Segments labelled in Figures 7, 8.

*Chaetotaxy*: Posteroventral margin of femora with row of strengthened setae (sometimes indistinct); posterior margin often with scattered setae. Fore tibia sometimes with one posteromedial seta (most *Japanagromyza* and some *Melanagromyza*, *Nemorimyza* and *Phytobia*). Mid tibia sometimes with one or two posteromedial setae, rarely three. Hind tibia sometimes with inner-distal “cleaning comb” (von Tschirnhaus 1991). Tibiae without dorsal preapical setae; at least mid tibia usually with small ventroapical seta.

*Wing*: (Figs 3, 4) Usually clear, rarely whitish or with faint to more distinct clouding, uncommonly patterned (more frequent in Neotropical species). Subcostal break present, humeral break absent; costa extending to R_4+5_ or M_1+2_; subcosta sometimes attaining costa, either as a vein or as a fold if the vein proper ends within the costal cell, but sometimes the subcosta is fused to R1 apically. Crossvein dm-m usually near midpoint of wing, sometimes shifted basally or absent; crossvein r-m present; vein bm-m rarely absent (some *Pseudonapomyza*). M1 and M4 sometimes faint to spectral; CuA+CuP usually faint to spectral, not reaching wing margin. Anal cell (cell cua) present, closed by vein CuA. Calypter (= squama of previous authors) prominent, semi-circular, and fringed with long hairs. Halter white, yellow, black, or bi-coloured.

*Female abdomen*: (Figs 11–23) Tergites and sternites 1–6 well-developed and setose, sternites narrower; tergites 1 and 2 fused. Segment 7 with tergite and sternite completely fused, enclosing spiracles; forming non-retractable, well-sclerotised, and usually elongate and partially shiny oviscape (= ovipositor sheath of Sasakawa and Basalglied of Hendel) that is usually dark brown to black but uncommonly paler; tergite 7 anteromedially with large, hollow, keel-like apodeme that projects into segment 6; remainder of abdomen past segment 7 usually retracted within oviscape, with membranous region past oviscape. Elongate membranous region between segments 7 and 8 (= segment 8 of Sasakawa) with two pairs of sclerotised bands; dorsal and ventral membrane past these bands densely covered with anteriorly directed denticles used to drill into plant tissue. Tergite 8 absent; sternite 8 divided into one pair of short egg guides with shape and arrangement of posteromedial sensilla variable; used for scraping out hole for egg positioning. Segment 9 absent (McAlpine 1981: 44). Segment 10 with tergite and sternite narrow, sometimes fused and often variably modified, terminating in one pair of short, rounded setose cerci that bear stout apical trichoid sensilla. One pair of usually subspherical spermathecae, often with basal invagination at point of insertion to spermathecal duct (reinforced with minute spiralled sclerotisations, excluding smooth apex), which connects spermathecae to elongate, membranous genital chamber. Ventral receptacle recurved with variably shaped apex.

*Male abdomen*: (Figs 139–142, 147–150, 446–451) Tergites and sternites 1–6 well-developed and setose, sternites narrower; tergites 1 and 2 fused; sternite 6 often large and broadly emarginated posteriorly. Male pregenital segments largely reduced to sternite 8, which is narrow, transverse, and often symmetrical and setose; sternites 6 and 7 absent or occasionally represented by lightly sclerotised regions anterior to epandrium ventrolaterally; tergites 7 and 8 absent. Epandrium (= tergite 9) dorsal and dome-like with slightly thickened anterodorsal margin that often produces one pair of raised points laterally to facilitate articulation with hypandrium. Surstylus (one pair) usually present, setose, variably shaped, sometimes with small tubercle-like setae on inner surface; base articulates with, or is variably fused to inner-ventral surface of epandrium or subepandrial sclerite. Cercus (one pair) variably sized, usually setose, sometimes with short spines on inner surface. Subepandrial sclerite (= sternite 10, or ventral epandrial sclerite of some authors) variably shaped, resting between inner surface of epandrium and hypandrial complex; usually plate-like with one pair of well-sclerotised dorsolateral arms (= bacilliform sclerites); sometimes medially divided, often with a pair of setae or spine-like processes, and/or one pair of paler ventral processes that may become thickened and enlarged (Fig. 490) (see Cerodonthasectionbelow for discussion of the structure called “*Langfortsatz*” by previous authors). Hypandrium (= sternite 9) U- or V-shaped with narrow transverse inner-basal lobe (“pregonite” of some authors, including Frick 1952a) bearing two or three apical setae that may be separate or weakly connected to the main body (e.g., *Ophiomyia* genus group); articulates with external components of genitalia via posterobasal “arms”. Pregonite absent (sections of postgonite or hypandrium sometimes interpreted as “pregonite”, such as by Papp and Černý 2015, 2020). Postgonite (one pair) usually long and narrow with one (but sometimes zero or several) seta and often small outer-medial sockets, usually slightly upturned and medially cleft apically; articulated with, surrounded by, or fused to inner lobe of hypandrium; subovate and plate-like in some Agromyzinae excluding *Agromyza*, but sometimes absent or possibly fused to epiphallus. Phallapodeme (= aedeagal apodeme of some authors) long and rod-like with extensive muscular attachment to project phallus from abdomen. Ejaculatory duct originates in ejaculatory apodeme and terminates in the distiphallus (“distal section” of Griffiths 1972a), the last section of the phallus that is sometimes divided into an apical distiphallus proper and a basal mesophallus. The distiphallus appears to be ancestrally bifid in the family, being subtly to conspicuously split in most Phytomyzinae with the paired tubules united or atrophied in a number of lineages (including species of *Xenophytomyza*, *Aulagromyza*, *Haplopeodes*, *Phytomyza*, etc.) or concealed in a basal enclosure (many *Liriomyza*, *Pseudonapomyza* and *Phytomyzaparvicella* (Coquillett)); the paired tubules of the distiphallus are usually less obvious or entirely unified in most Agromyzinae (but see Figs 148, 256, for example), with almost all representatives of the *Ophiomyia* genus group appearing to have a single exit pore (Lonsdale 2014: fig. 38); in some *Phytomyza*, the dorsobasal margin of the distiphallus branches into a sclerotised structure called the “supporting sclerite” (Fig. 752). Basiphallus (“basal section” of Griffiths 1972a) divided into two segments, the basiphallus proper (“basal sclerites or paraphalli” of Griffiths 1972a, “endophallus” of Guglya 2013, “mesophallus” of Papp and Černý 2015), which is one or two plates (rarely more) laterally flanking ejaculatory duct before it enters distiphallus, and the basal phallophorus, a tubular or ring-like basal segment through which the duct enters; phallophorus allows for articulation between phallapodeme and remaining distal components of phallus, is fused to the epiphallus and sometimes the basiphallus, and is sometimes flanked by one pair of sclerites that may be derived from the epiphallus (some *Ophiomyia*) or the phallapodeme (most *Phytomyza*). Hypophallus (= ”medial lobe” of Griffiths 1972a, “trough-like sclerite” of Griffiths 1974 and “wedge-shaped sclerite” of Griffiths 1980) a lobate membranous region ventrally near apex of basiphallus that is variably sclerotised and/or expanded; if present, sclerites may articulate or fuse with basiphallus or (less commonly) disti/mesophallus; sclerotisations may consist of one pair of plates or rods that may meet each other ventrally and/or reach basiphallus dorsally; sclerite sometimes present as single small medial bar that may end in hair-like structures (many *Liriomyza*) and/or widen to form flat, irregular plate (as in some *Calycomyza*). Paraphallus (“paramesophalli” of Griffiths 1972a) (one pair) small, sclerotised, and usually plate or rod-like, usually directed ventrally to ventrodistally; positioned ventrolaterally at base of distiphallus / mesophallus; possibly homologous to transverse sclerite wrapped around the posterobasal surface of distiphallus / mesophallus in *Agromyza*. Epiphallus divided into two parts, a smaller, darker proepiphallus (“epiphallus” of Frick 1952a), which articulates with phallophorus, and a wider, flatter metepiphallus (“aedeagal hood” of Frick 1952a), which articulates/fuses to proepiphallus and postgonite and/or hypandrium; components of epiphallus lie along each other in repose and fold outwards during copulation to extend phallus, but folding mechanism abandoned in most Agromyzinae, with reduced proepiphallus and usually enlarged and elaborated metepiphallus separated by membranous space. Ejaculatory apodeme composed of large, flat sclerotised blade with asymmetrical base, and smaller, clear basal sperm pump that often has transverse sclerotisation (= *pileus ejaculatorius* of Steyskal).

**Notes on male genitalic homology.** Regarding structural homology, a number of points must be clarified. Firstly, as the basalmost component of the phallus meeting the phallapodeme, the phallophorus is clearly homologous with the “basiphallus” of other Schizophora. The term “phallophorus” is here retained, and treated as the basal segment of the basiphallus proper. As the structure is present and often distinct in taxa throughout the family, and it has had wide usage in the literature, the term has utility. It is not entirely certain whether the agromyzid “basiphallus” originates from the same sclerite as the phallophorus, but in assuming this homology, continuity is maintained with the existing literature, and confusion is avoided by eliminating the need to rename structures. The alternative interpretation would be to consider the agromyzid “basiphallus” a novel, secondary sclerotisation of the lateral phallic membrane, but this explanation is not more parsimonious and it does not have the above advantages. Papp and Černý (2015) partially agree with this interpretation, although their “basiphallus” consists solely of the phallophorus; what they call the “mesophallus” is the basiphallus of this study and other works.

Both the phallophorus and folding epiphallus appear to have counterparts in other acalyptrate Diptera and may be phylogenetically informative, but the homology and polarity of these are uncertain. Both structures appear to aid in projecting the phallus from the abdomen for copulation, contrasting the lever and fulcrum method employed by families such as Clusiidae, or the method employed by Tephritoidea, etc., but functional requirements may make these prone to convergence.

Lastly, the position and shape of the hypophallic sclerites in the ventral region distal to the basiphallus varies widely between species, with numerous intermediate forms. Some complex forms have led some authors (e.g., Griffiths 1972a, 1974, 1980) to invent novel terminology for the sclerites present. Noting this variation, and the absence of any sclerotised structures in some taxa, there is the possibility of repeated loss and gain of analogous structures that may be difficult to interpret. Until a more thorough analysis of the structures in this region is made to establish homology, the single term “hypophallus” will be applied to the membranous lobe in this region, including any sclerotised structures found thereupon.

### ﻿Agromyzidae – immature description

*Egg*: Small and clear with shape ovate to reniform; surface texture smooth to reticulate. End bearing small micropyle often broader.

**Figures 24–36.**
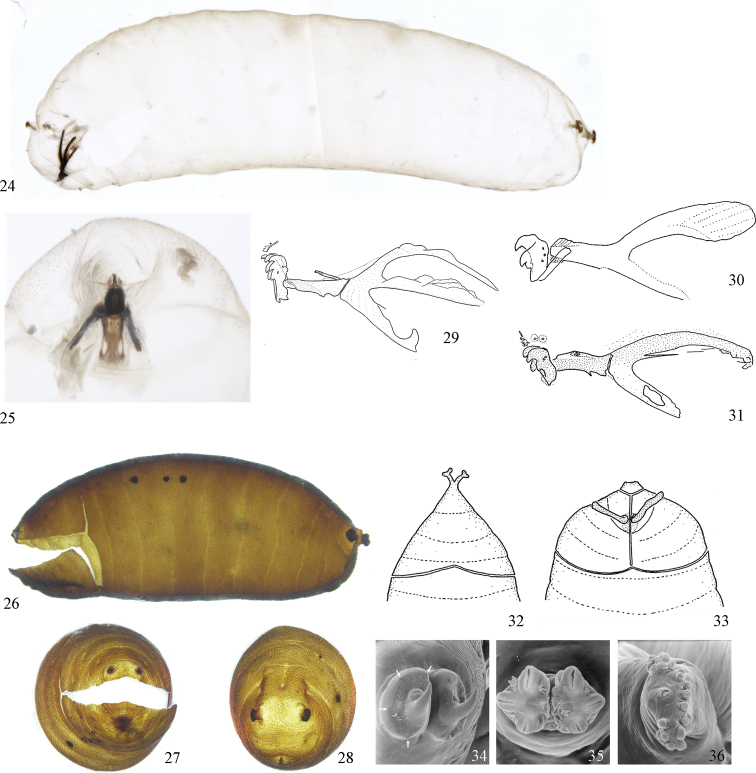
*Phytomyzachelonei* Spencer, larva **24** lateral **25** same, anteroventral **26–28***Phytomyzamisella* Spencer, puparium **26** lateral **27** same, anterior **28** same, posterior **29–31** cephalopharyngeal skeleton, lateral **29***Agromyzaanthracina* Meigen **30***Liriomyzapusilla* (Meigen) **31***Phytomyzahorticola* Goureau **32, 33** puparium, anterior end, dorsal **32***Phytomyzamilii* Kaltenbach **33***Tropicomyiatheae* (Cotes) **34–36** posterior spiracles **34***Agromyzaalbipennis* Meigen **35***Melanagromyzalappae* Loew **36***Phytomyzaranunculi* (Schrank). Figs 29–36 reproduced from Dempewolf (2001).

*Larva*: (Figs 25–31) Typically maggot-like, cylindrical, soft and with ends tapered; *Phytobia* species often longer and narrower (Greene 1914, 1917; Spencer 1987); form varying very little between species with different feeding habits such as leaf-mining and stem-boring (Dempewolf 2005). Mandibles usually asymmetrical; firmly fused at base with movement along the same axis (Spencer 1987); each with as few as one or two accessory hooks, which may be duplicated and variable in shape; often becoming reduced and strengthened in boring species (Dempewolf 2005). Hypopharyngeal sclerite short, linking mandibles to tentopharyngeal sclerite, which has one pair of “arms” (or “cornus”); Agromyzinae with dorsal and sometimes ventral arms of tentopharyngeal sclerite encompassing small “window” that is open posteriorly in dorsal arm (Fig. 29); Phytomyzinae sometimes with window in ventral arm only (Figs 24, 25, 30, 31; Spencer 1987). Anterior and posterior spiracles with at least three or five (but sometimes many more) slit-like openings in clusters of lobes/bulbs that may be narrow and reduced or strongly projecting and expanded, especially in species that use spiracles to anchor puparium to host plant surface (Figs 35, 36); posterior spiracles sometimes with “hook” (Fig. 34); anterior spiracles closely spaced dorsally (Figs 24, 27) (Ferrar 1987; Spencer 1987).

*Puparium*: (Figs 26–36) Surface smooth and shiny; colour usually pale yellow to reddish brown or dark orange (Figs 26–28), but sometimes white or deep black with metallic shine (e.g., *Cerodontha*), with overwintering puparium dark brown to black. Shape slender or flattened to more compact and barrel-shaped; segmentation often clearly evident, minute sculpturing or pits sometimes present. Puparial lid with additional split along the midline in species of *Ophiomyia* genus group (compare Fig. 33 to Fig. 32; Dempewolf 2001).

### ﻿Key to the north American genera of Agromyzidae

**Table:** 

1	Subcostal vein well-developed along entire length and fused to vein R1 before reaching costa (Fig. 4). Vein R1 sinuous for a short distance just before reaching costa, and sometimes thickened (relatively straight in some *Agromyza*, which has a distinct stridulatory file on lateral margin of tergite 2). Vein R_4+5_ ending as close, or closer to wing apex compared to vein M1. Lunule often with medial furrow, well-sclerotised and flat to bulging (recessed in some *Agromyza* and some poorly preserved material)	**Agromyzinae (2)**
–	Subcostal vein either ending freely in cell or continuing as a fold or atrophied vein to the costa (Fig. 3). Vein R1 straight, not deviated before reaching costa, infrequently thickened apically (excluding some *Phytobia*). Lateral margin of tergite 2 never with stridulatory file. Vein M1 usually closer to wing apex compared to vein R_4+5_ (except in *Phytobia* and some *Nemorimyza*). Lunule without medial furrow (except in some *Phytobia*), often soft, and usually flat or recessed (infrequently bulging)	**Phytomyzinae**(**8)**
2	Prescutellar acrostichal seta present (Figs 10, 51); halter usually white, at least apically	3
–	Prescutellar acrostichal seta absent and halter entirely dark (apically white in *Ophiomyiamaculata* Spencer)	4
3	Lateral margin of tergite 2 and fused tergite 1 with straight, thin, and well-sclerotised stridulatory file (Fig. 38). Usually at least three conspicuous dorsocentral setae (Fig. 10). Fore tibia without medial seta. Frons sometimes soft and pale; body never with metallic shine	***Agromyza* Fallén**
–	Lateral margin of tergites 1 and 2 weakly sclerotised and sometimes irregular in outline, never with stridulatory file. Only two well-developed dorsocentral setae (Fig. 51), frons well-sclerotised, rounded and dark, and gena shallow (Figs 49, 50) (*J.rutiliceps* with four dorsocentrals, a projecting frons and gena ¼ height of eye); fore tibia usually with medial posterolateral seta; body sometimes with metallic shine	***Japanagromyza* Sasakawa (in part)**
4	Clypeus with anterior margin truncated anteromedially (Figs 71, 73); sometimes narrowed (Figs 77, 80), often with conspicuous corners anteriorly and arms parallel. Sometimes with one or more of a pronounced facial keel that may have a medial bulb, an anteriorly produced gena, and multiplicated vibrissae forming a “fasciculus” or “horn” (Figs 75–77)	***Ophiomyia* Braschnikov**
–	Clypeus rounded anteromedially; if slightly truncated (some *Melanagromyza*), then with one or more of the following characters: dorsal setulae on eye; one posteromedial seta on fore tibia; more than 1 posteromedial seta on mid tibia; entire thorax and abdomen strongly metallic. Gena sometimes angled anteriorly (*M.buccalis*, Fig. 53), but never forming a point; vibrissa single, simple	5
5	Fore tibia with small to well-developed posteromedial seta (Fig. 121)	6
–	Fore tibia bare posteromedially	7
6	Halter white. Costa extending to M1. Only two developed dorsocentral setae (Fig. 51). Ocellar triangle only reaching level of posterior fronto-orbital seta; frons and lunule smooth with minute pruinosity; orbital plate not raised (Figs 49, 50). Eye bare or with few small, inconspicuous ommatrichia. Epandrium without spine. Basiphallus asymmetrical; often with left and right sides divided (Figs 255, 256). Mesophallus (or if absent, insertion of ejaculatory duct) basal to distiphallus; often forked or membranous. Distiphallus without paired ventrobasal tubules	***Japanagromyza* Sasakawa (in part)**
–	Halter brown. Costa extending to M1 or (uncommonly) R_4+5_. Usually two dorsocentral setae, uncommonly three or four. Ocellar triangle exceeding level of posterior fronto-orbital seta; frons variable, lunule smooth; orbital plate distinct and often raised or bulging (Figs 52–57). Eye often conspicuously with ommatrichia dorsally, particularly in males. Epandrium usually produced into small, but conspicuous spine posteroventrally (Fig. 267). Basiphallus symmetrical; ring- (Fig. 304) or U-shaped (Fig. 256). Mesophallus inserted ventromedially into distiphallus as a cylindrical chamber (Figs 264, 265). Distiphallus with paired ventrobasal tubules, often flanking mesophallus	***Melanagromyza* Hendel (in part)**
7	Thorax (and sometimes head and abdomen) sometimes with green (Fig. 60), blue or coppery metallic sheen. Eye often conspicuously with ommatrichia, particularly in males. Orbital plate usually small, not much visible laterally, but sometimes prominent as below. Mid tibia with 1 or 2 posteromedial setae. Postsutural intra-alar setae unequal in length with anterior seta stronger. Epandrium usually with posteroventral spine (Fig. 267). Phallophorus simple, cylindrical (Figs 264, 265). Basiphallus symmetrical; ring-like or U-shaped. Distiphallus with paired ventrobasal tubules, often flanking mesophallus	***Melanagromyza* Hendel (in part)**
–	Thorax without metallic sheen (Figs 44–48). Eye bare. Orbital plate bulging, forming prominent ring with parafacial. Mid tibia without posteromedial setae. Postsutural intra-alar setae approximately subequal. Epandrium without spine as below (Fig. 250). Phallophorus produced anteroventrally into a large pouch. Basiphallus asymmetrical. Distiphallus never with paired basal tubules	***Euhexomyza* Lonsdale**
8	Lunule conspicuous, at least as high as pedicel, well-sclerotised and usually convex (even if partially concealed by pronounced orbital plates) (Figs 99–105); ranging from subcircular to long and narrow, but if shallower than a semicircle, lunule very large and broad (some *Dizygomyza*). Orbital setulae sometimes only reclinate in part, usually entirely erect to reclinate; vein dm-m always present. Distiphallus often long, robust and bifid (sometimes secondarily fused or more membranous) with apex usually swollen or bell-shaped (Figs 493, 494, 511, 512, 543, 544)	***Cerodontha* Rondani** (**9)**
–	Lunule weakly sclerotised, slightly concave and usually shallow; if appearing higher than pedicel, then either orbital setulae entirely proclinate (*Phytomyza* and some *Phytoliriomyza*) or epandrium with rows of conspicuous tubercle-like setae (some *Phytoliriomyza*). Distiphallus various	**15**
9	First flagellomere pointed (Fig. 106) or angled anterodorsally. Scutellum with lateral scutellar setae absent (i.e., only one pair of setae present apically)	**10**
–	First flagellomere rounded anteriorly. Scutellum with two pairs of setae (some global *Poemyza* rarely with one pair, but lunule very high and narrow)	**11**
10	First flagellomere produced as a thin point anterodorsally (Fig. 106); point uncommonly reduced, some non-Nearctic species with point absent and segment simply angulate. Body with conspicuous yellow patches	***C﻿.* (*Cerodontha*) Rondani**
–	First flagellomere angulate anterodorsally. Body brown to black (Fig. 104)	***C﻿.* (*Xenophytomyza*) Frey**
11	Costa only extending to vein R_4+5_	***C﻿.* (*Phytagromyza*) Hendel**
–	Costa ending at vein M1	**12**
12	Prescutellar acrostichal seta present. Lunule slightly higher than wide, well-sclerotised, smooth, gradually tapering to a point dorsally, and light brown to brown with even covering of very short velvety pubescence (Fig. 99)	***C﻿.* (*Butomomyza*) Nowakowski**
–	Prescutellar acrostichal seta usually absent, but uncommonly weak to well-developed. If lunule higher than wide, then not as above. Lunule uncommonly approaching state as defined above, but if so (e.g., C. (D.) scirpivora Spencer), then lunule smoothly rounded above and male first flagellomere densely haired	**13**
13	Lunule narrow, either higher than wide or appearing higher than wide with sides obscured beneath prominent orbital plates (Figs 101, 102); surface often shiny and minutely textured	***C﻿.* (*Poemyza*) Hendel**
–	Lunule as wide, or wider than high; surface smooth with indistinct to prominent pubescence	**14**
14	Lunule flat or curved, meeting anterior margin of frons, well-sclerotised (Fig. 105). Head usually entirely grey to dark brown. Male first flagellomere often enlarged and sometimes with long, conspicuous hairs; antennal bases often distinctly separated	***C﻿.* (*Dizygomyza*) Hendel**
–	Lunule bulging, usually projecting higher than sunken anterior margin of frons, weakly sclerotised (Fig. 103). Lunule bright yellow with frons sometimes also yellow. Male first flagellomere not larger than those of females and with hairs moderately sized; antennal bases closely approximated	***C﻿.* (*Icteromyza*) Hendel**
15	Vein R_4+5_ as close, or closer to wing apex than vein M_1_ (Figs 689, 690). Lunule often silvery or with shiny pubescence (Fig. 124). Usually large and stout-bodied. Subepandrial sclerite stout, with halves broadly fused, and usually with single large spine (pronounced in *Nemorimyza*) (Figs 670, 684)	**16**
–	Vein M1 closer to wing tip than vein R_4+5_ (Figs 429, 693, 836). Lunule often bare or pubescent, rarely silvery. Usually small and slender-bodied. Subepandrial sclerite various, but u ncommonly as above	**17**
16	Fore tibia sometimes with posteromedial seta (Fig. 121); halter sometimes with dark spot. Surstylus with base elongate, strongly projecting dorsally into epandrium (Figs 662, 663). Basiphallus composed of two wide interlocking plates; apices flanking hypophallus narrow and curved (Figs 665, 666). Distiphallus stout, with abruptly widened base. Common Holarctic species (*N.posticata* (Meigen)) with male abdomen posterior to tergite 3 or 4 yellow	***Nemorimyza* Frey**
–	Fore tibia rarely with posteromedial setae. Halter entirely white. Surstylus small, lobate (Figs 683–685). Male abdomen usually entirely dark, never as above. Basiphallus not as above; narrowed with halves fused basally to form a stalk (Fig. 681). Distiphallus usually narrower than above, at least towards base	***Phytobia* Lioy**
17	Costa extending to vein M1 (Fig. 463)	**18**
–	Costa extending to vein R_4+5_, or slightly past (Fig. 435)	**24**
18	Two distinct dorsocentral setae, with anterior two pairs highly reduced and barely longer than surrounding setulae (few global species with three or four setae developed). Head and notopleuron pale yellow with antenna and most of remaining notum contrastingly dark (some exceptions outside of Nearctic) (Figs 90–98). Epandrium and surstylus each with dense clusters of tubercles (Figs 446–448)	***Calycomyza* Hendel**
–	Three or more pronounced dorsocentral setae; if only two present, colour never as above. Arrangement of tubercles on epandrium usually much reduced (Figs 621, 626), arranged in rows (Fig. 710) or otherwise not as above (but see *Liriomyzaquadrisetosa* – Figs 616–620)	**19**
19	Halter white to entirely black, but never white with apical surface brownish. Distiphallus usually black, dense, and surrounded by minutely spinulose membrane (Figs 424, 426). Gena usually 1/3–1/2 height of eye (Fig. 87), but sometimes shallow (Fig. 85). Ejaculatory apodeme large, bowl-shaped sclerotisation on sperm pump (Fig. 418)	***Amauromyza* Hendel**
–	Halter white, sometimes with apical surface of halter brown. Distiphallus without spinulose membrane. Gena usually < 1/3 eye height, uncommonly higher. Sperm pump uncommonly as above, usually with much smaller, ovate or band-like sclerotisation (Figs 464, 472, 531, 588) that may be vestigial to absent (Fig. 659)	**20**
20	Scutellum yellow centrally and orbital plate brown and slightly to conspicuously pronounced, contrasting with the weakly sclerotised central vitta (also usually brown). Broad, sclerotised ocellar triangle and orbital plates joined to form sharp angles with the outline of an “M”. Surstylus with at least one, and epandrium with two rows of tubercle-like setae (Figs 654–657)	***Metopomyza* Enderlein**
–	If orbital plate dark brown as above, then not pronounced and scutellum entirely brown. If ocellar triangle and orbital plate joined along posterior margin of frons, then broadly joined by an intervening space (i.e., connecting angle broadly rounded or squared). Male genitalia infrequently as above (some *Phytoliriomyza*)	**21**
21	Apical surface of halter light brown (Fig. 127). Orbital setulae proclinate. Eye with minute ommatrichia. Side of frons with light silvery pubescence (Fig. 126). Scutellum entirely brown or yellowish medially	***Phytoliriomyza* Hendel (in part)**
–	Halter entirely white. Orbital setulae upright to reclinate, sometimes absent; if proclinate (some *Phytoliriomyza*) then epandrium with dense row of tubercle-like setae. Eye bare or with few scattered ommatrichia. Side of frons not reflective as above. Scutellum usually distinctly yellow medially	**22**
22	With one of the following characters: frons with slight reflective pruinosity anterolaterally; orbital setulae proclinate; first flagellomere slightly elongate and black; scutellum and large portions of scutum matt grey; epandrium and surstylus with rows of tubercle-like setae (Fig. 705)	***Phytoliriomyza* Hendel (in part)**
–	Frons never with reflective pruinosity. First flagellomere yellow to dark brown, never black. Scutellum usually subshiny to shiny (sometimes pruinose) and with central yellow stripe (sometimes reduced to absent). Epandrium and surstylus usually with one or two tubercle-like setae, but if with more, these never arranged in a discrete row	**23**
23	Vein dm-m often present (Fig. 652). Male abdomen sometimes with anterolateral stridulatory organ in membrane (Fig. 107). Phallus with at least two distinct sclerotised segments (Figs 593, 594). Apical section of ejaculatory duct pigmented and abruptly dilated	***Liriomyza* Mik**
–	Vein dm-m often absent (Fig. 581). Male abdomen never with stridulatory organ as above. Phallus small and membranous with base sclerotised (Figs 579, 580). Apical section of ejaculatory duct clear and of equal width along length	***Haplopeodes* Steyskal**
24	Orbital setulae proclinate (Fig. 133). Phallophorus often flanked by one pair of rod-like sclerites (Figs 803, 804)	***Phytomyza* Fallén *s. l.* (25)**
–	Orbital setulae reclinate or upright (as in Fig. 2). Phallophorus without paired lateral sclerites	**26**
25	Vein dm-m present; usually in line with, or slightly basal to r-m, but sometimes slightly distal to r-m as below. Scutum mostly dark with grey pruinosity (Fig. 131). Distiphallus undivided apically, with apical sclerotised ring or tubule connected to dorsal sclerotisation on shaft	***P.* (*Napomyza)* Westwood**
–	Vein dm-m usually absent; if present, then positioned slightly distal to r-m (Fig. 435). Scutum variably coloured and tomentose (Figs 132–138). Distiphallus usually divided apically; if undivided and with apical ring, then ring continuous with ventral sclerotisation along shaft	**P. (Phytomyza) Fallén**
26	Vein r-m basal to dm-m (Fig. 88); if dm-m absent, then r-m positioned far from vein bm-m and colouration bright yellow (Fig. 89). Vein M sclerotised far basal to vein r-m. First flagellomere rounded apically, sometimes with dorsomedial margin forming a slightly raised angle	***Aulagromyza* Enderlein**
–	Vein dm-m and basal section of M absent, with r-m and bm-m nearly forming a straight transverse line (Fig. 833) [some Old World species with vein r-m distal to vein dm-m, with dm-m enclosing a very small cell between it and bm-m]. Colour always black (Fig. 130). First flagellomere with projecting anterodorsal angle [Old World species outside *P.atra* group with segment rounded]	***Pseudonapomyza* Hendel**

### ﻿Taxonomic treatment

#### Agromyzinae

***Agromyza* Fallén**

Agromyza Fallén, 1810: 21. Type species: reptans Fallén 1823, by subsequent designation [Rondani 1875: 168]. Described without named species. Westwood 1840: 151 [invalid design. of nigripes Meigen 1930 as type]; Rondani 1856: 121 [designation of aeneoventris Fallén as type (now Melanagromyza)], 1875: 168 [designation of reptans as type]; Frick 1952a: 370 [synonymy]; Spencer 1969: 29; Spencer and Steyskal 1986a: 183 [proposed fixation of reptans as type], 1986b: 57; ICZN 1988: 76 [fixation of reptans as type].

Calyptomyza Hardy, 1850: 486. Type species *atra* Hardy, 1850 [= Agromyzalathryi Hendel, 1923], by monotypy (Bland 2000; synonymy).

Adromyza. Misspelling. Meigen 1830: viii.

Domomyza Rondani, 1856: 121 [incorrectly treated as Agromyzidae]. Type species: *cincta* Rondani, 1956 (= CacoxenusindagatorLoew 1858), by monotypy. Sidorenko 1996: 21 [petition to suppress Domomyza – senior synonym of Cacoxenus Loew, 1858 (Drosophilidae)]; ICZN 1997: 195 (suppression of genus name).

Mesonevra Lioy, 1864: 1312. Type species: AgromyzamobilisMeigen 1830, by monotypy. Syn. Frick (1952a).

Cecidomyiaceltis Patton, 1897: 247. Type species: CecidomyiaceltisdesertaPatton 1897: 247, by original designation. Syn. von Tschirnhaus (2016).

Stomacrypolus Enderlein, 1936a: 178 [nomen nudum – no type species designated].

Stomacrypolus Enderlein, 1936b: 42 [attributes to Enderlein 1936a]. Type species: Agromyzaambigua Fallén, 1823, by original designation. Frick 1952a [synonymy not explicit]. Spencer and Martinez 1987: 255 [listed as “error (Enderlein 1936b)”].

**Figures 37–44.**
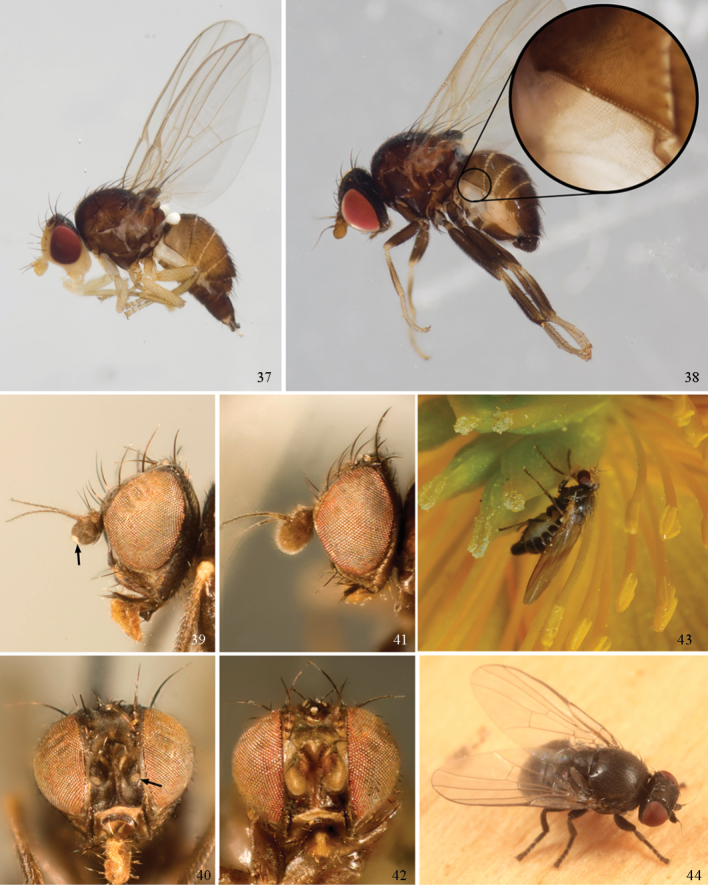
**37***Agromyzaaristata* Malloch, female lateral **38***Agromyzabispinata* Spencer, female lateral with detail of stridulatory file **39, 40***Agromyzakincaidi* Malloch, male head (arrow indicates discrete apical tuft of hairs) **39** lateral **40** same, anterior **41, 42***Agromyzabispinata*, male head **41** lateral **42** same, anterior **43***Agromyza* sp., female (S.A. Marshall – Ontario, Hornings Mills) **44***Euhexomyzaschineri* (Giraud) (T. Murray – Massachusetts, Groton).

Both sexes of *Agromyza* have a dark, straight, well-sclerotised file along the lateral margin of the fused first and second tergites, making it an easily recognised genus. The second tergite itself is also produced posteriorly along the lateral margin, forming a shallow, yet distinct angle (Figs 37, 38). Species in this genus also have one pair of prescutellar acrostichals and three or more dorsocentrals, although several, including *A.bispinata* Spencer, *A.parilis* Spencer and their relatives usually have only two that are immediately apparent. Internally, most species have a transverse sclerite wrapped around the posterobasal surface of the mesophallus (which is often fused to the distiphallus and possibly homologous to the paraphallus), but this is absent in some taxa such as *A.varifrons* Coquillett and *A.vockerothi* Spencer. There is also no basal sclerotisation on the sperm pump (also absent in many *Japanagromyza*), there is a haired membrane extending from the posterior face of the hypandrial lobe (sometimes indistinct), and most of those species with a transverse sclerite on the distiphallus also have a unique phallus (Figs 168, 169): the two sclerites of the basiphallus are approximated basally, divergent distally and the apex is abruptly elbowed inwards; the S-shaped distiphallus (including the fused mesophallus) is usually capsule-like with a ventral subbasal, crack-like fissure for entry of ejaculatory duct. This phallus type is found in numerous species, including those in the *A.nigripes* group, a diverse, predominantly Poaceae-feeding clade (Hendel 1931; Griffiths 1963; Spencer 1990: 353).

For the purposes of species-level identification, the morphology (including shape, colour, and hairiness) of the first flagellomere is quite important, and if these are missing from the specimen it is recommended that the male genitalia be dissected.

### ﻿Key to the Delmarva species of *Agromyza*

**Table:** 

1	Palpus yellow (apex infuscated in *A.apfelbecki*). Face white to light brown, rarely brown. First flagellomere usually light yellow, sometimes with outer surface lightly infuscated	2
–	Palpus brown or darker. Face light to dark brown. First flagellomere orange to dark brown	8
2	At least 6 dorsocentral setae and 5 fronto-orbital setae. Wing length at least 4.0 mm. Epistoma large, distinct	***A.apfelbecki* Strobl**
–	No more than 4 large dorsocentrals and four fronto-orbital setae. Wing length 3.4 mm or less. Epistoma narrow	3
3	Body, excluding most of head and legs, yellow. Thorax shiny. Mid tibia with 2 or 3 posteromedial setae. First flagellomere with apical patch of slightly longer, paler hairs. Wing length 2.7–3.4 mm. Eye 6.1–9.7 × higher than gena. Surstylus indistinct, appearing as broadly rounded ventral margin of epandrium; with tubercle-like setae (Fig. 180). Cercus only with unmodified setae. Distiphallus terminating in a large, dark bulb (Figs 181, 182). Basiphallus consisting of 1 pair of lateral bands	***A.diversa* Johnson**
–	Body, excluding most of head, dark brown. Thorax densely to lightly pruinose. Mid tibia without posteromedial setae. First flagellomere with all hairs similar. Wing length usually 1.7–2.4 mm; 3.4 mm in *A.deserta*. Eye 1.6–3.9 × higher than gena. Surstylus ending in triangular point; without tubercle-like setae. Cercus with tubercle-like setae along inner face. Distiphallus a flat, curved sclerite. Basiphallus consisting of a single band	4
4	Thorax with dense grey pruinosity. Calypter margin and hairs white. Legs light yellow. Parafacial visible laterally, continuing under eye as distinct cheek. Ca. 8 rows of acrostichal setulae. Eye 1.6–2.7 × higher than gena. Length of ultimate section of vein M4 divided by penultimate section: 0.6–0.9. Mesophallus several ×longer than distiphallus (males unknown for *A.pallidiseta*). Ejaculatory apodeme minute and subcylindrical	5
–	Thorax with light dusting of pruinosity. Calypter margin and hairs brown. Legs dark brown with tarsi and knees paler. Parafacial not visible laterally; cheek not strongly delimited. Four rows of acrostichal setulae. Eye 3.5–3.9 × higher than gena. Ultimate and penultimate sections of vein M4 of equal length. Mesophallus shorter than distiphallus. Ejaculatory apodeme large and fan-shaped	7
5	Setae yellow. Ocellar seta 1/2 length of postocellar	***A.pallidiseta* Malloch**
–	Setae dark brown. Ocellar seta as long, or nearly as long as postocellar	6
6	Wing length 2.0–2.4 mm. Legs yellow with mid and hind coxae at least partially brown (Fig. 37). Presutural dorsocentral not much longer than surrounding setulae. Distiphallus straight (Figs 165, 166)	***A.aristata* Malloch**
–	Wing length 3.4 mm. Legs brown with apex of femora and base of tibiae yellow. Presutural dorsocentral distinct. Distiphallus curved	***A.deserta* (Patton)**
7	First flagellomere entirely light yellow, or with dark dorsobasal spot. Mid and hind tibiae dark brown with base yellow, and fore tibia yellow with centre brownish. Male cercus thin along length (Fig. 237)	***A.varifrons* Coquillett**
–	First flagellomere yellow with outer face lightly infuscated on distal 2/3. Tibiae dark brown with only base of fore tibia yellowish. Male cercus broad and V-shaped (Figs 186, 187)	***A.fission* Eiseman & Lonsdale**
8	Five or 6 fronto-orbital setae, with only one ors (some *A.virginiensis* with posterior ori appearing as ors). Fronto-orbital plate and parafacial not projecting. Centre of frons always brownish orange. Distiphallus long, thin, tubular, and bifid	9
–	Four fronto-orbital setae on one or both sides of frons, with two ors, but if 5 fronto-orbitals present (*A.aprilina*), fronto-orbital plate and parafacial projecting. Centre of frons usually dark brown (pale in *A.aprilina*). Distiphallus undivided	**10**
9	At least apical 1/2 of first flagellomere brown. Anterior margin of buccal cavity sharply angulate. Distiphallus strongly curved, forming a semicircle (Figs 147, 148)	***A.ambrosivora* Spencer**
–	First flagellomere entirely orange, with dark spot around base of arista, or with distal 1/2 darker. Anterior margin of buccal cavity straight or slightly rounded, at most with midpoint slightly produced. Distiphallus nearly straight (Fig. 151)	***A.virginiensis* Spencer**
10	First flagellomere black; elongate to slightly enlarged and with anterodorsal angle. Body dark brown to black. Calypter margin and hairs white. Distiphallus relatively short	**11**
–	First flagellomere dark brown or paler; circular to elongate oval, with apex broadly rounded; if flagellomere black and with anterodorsal point (some *A.kincaidi*), then segment not elongate, body paler, calypter margin and hairs usually yellow to brown, and distiphallus large and clavate	**12**
11	Costa extending just past vein R_4+5_. Five fronto-orbital setae. Sides of ocellar triangle straight. Anterodistal margin of epandrium deeply incised before surstylus (Figs 157, 158). Surstylus acutely triangular and directed anteriorly. Distiphallus tapered to apex, which is directed distally (Fig. 160)	***A.aprilina* Malloch**
–	Costa extending to vein M_1+2_. Four fronto-orbital setae (some Canadian material with five). Sides of ocellar triangle slightly concave. Epandrium without deep emargination as above. Surstylus relatively small and lobate (Fig. 143). Distiphallus parallel-sided with apical 1/2 slightly angled dorsally (Fig. 146)	***A.albipennis* Meigen**
12	Four strong dorsocentral setae, presutural seta nearly as long as preceding seta and nearly as long as acrostichal seta. Thorax covered with brownish grey pruinosity, strongly contrasting brown to golden-brown setae. Mid tibia with 1 posteromedial seta. Basiphallus consisting of a single sclerite. Distiphallus and separate mesophallus simple and plate-like	**13**
–	Three to 6 dorsocentral setae, strongly decreasing in height anteriorly (anterior setae often barely larger than surrounding setulae). Thorax usually shiny, but sometimes with light pruinose dusting. Setae usually dark brown, but sometimes brown or (rarely) yellow. Mid tibia with two posteromedial setae. Basiphallus consisting of 2 sclerites. Distiphallus (including fused mesophallus) with transverse dorsobasal band; bulb-like or long and bifid	**14**
13	Calypter hairs brown. Antenna entirely dark brown. Mid and hind tibiae brown with apices yellow (base sometimes also yellow). Distiphallus longer than wide, flat, and curved; short, cylindrical with longer ventral plate (Figs 248, 249)	***A.vockerothi* Spencer**
–	Calypter hairs usually white (only brown in holotype). Base of first flagellomere paler in colour, usually distinctly orange. Mid and hind tibiae yellow, sometimes with hind tibia brownish medially. Distiphallus very short, mesophallus dense and thick apically, and with long, thin, flat “tail” (Figs 191, 192)	***A.isolata* Malloch**
14	First flagellomere usually at least 1.3 × longer than pedicel (larger in males); shape circular (most males) or elongate oval (some males and most females); with elongate pale hairs present and restricted to distal margin in females, but often extending to basal 1/2 or basal 1/3 of segment in males (Figs 41, 42); long-haired section without sharply delimited boundary, and with hairs relatively more sparse than in “tufted” state described below. Only males can be identified further	**15**
–	First flagellomere not much longer than pedicel, with shape ovate; longer hairs, if present, restricted to discrete, sharply delimited apical tuft that may form a minute spot (Figs 39, 40) or encompass the distal 1/3 of the segment	**17**
15	Surstylus with 2 tubercle-like setae of variable size; small and lobate, directed anteriorly and barely visible laterally (Figs 170–173)	***A.bispinata* Spencer**
–	Surstylus with 3 or more tubercle-like setae, rarely 2; subtriangular in outline and distinct laterally	**16**
16	Surstylus with 3–5 tubercle-like setae (rarely 2) (Figs 226–228). Margin of surstylus, seen laterally, bulging at base of tubercles	***A.tacita* Spencer**
–	Surstylus with numerous tubercle-like setae (Figs 232, 233). Margin of surstylus, seen laterally, relatively straight	***A.echinalis* sp. nov.**
17	If present, pale tuft of hairs on first flagellomere encompassing entire distal 1/3 of segment, or nearly as high as segment and teardrop-shaped. Scutum with light pruinosity. Four or 5 dorsocentrals, often with 1 presutural. Distiphallus cup-shaped with central tuft of hairs (strongly enlarged in *A.canadensis*)	**18**
–	If present, pale tuft of apical hairs on first flagellomere circular to ovate in outline, and ranging in size from 1/2 height of first flagellomere to only as wide as base of arista. Scutum usually shiny to subshiny. Number of dorsocentrals variable, but often with only 2 well-developed setae. Distiphallus capsule-like and usually bell-shaped in outline	**19**
18	Three postsutural dorsocentral setae and one sutural to presutural dorsocentral (European specimens with 1 or 2 additional dorsocentrals). Buccal cavity curving onto face. Parafacial entirely visible laterally. Frons orange-brown with fronto-orbital plate dark brown. Distiphallus as long as mesophallus, and with distal 1/2 flared (Figs 141, 142). Mesophallus with dorsal collar	***A.abiens* Zetterstedt**
–	Four postsutural dorsocentral setae and sometimes 1 sutural to presutural dorsocentral (Fig. 10). Margin between face and buccal cavity abruptly angled. Parafacial barely visible laterally (Fig. 8). Sides and centre of frons the same colour. Distiphallus much enlarged, ring-shaped (Figs 177, 178). Mesophallus without collar	***A.canadensis* Malloch**
19	Costa extending to vein R_4+5_. Wing length 3.6–4.3 mm. Distiphallus longer than basiphallus and dark (Figs 196, 197)	***A.kincaidi* Malloch**
–	Costa extending to vein M_1+2_. Wing length < 2.6 mm. Distiphallus shorter than basiphallus and usually pale	**20**
20	Five dorsocentral setae, with presutural dorsocentral as long as preceding setae, and nearly as long as acrostichal seta. Calypter hairs white to light brown in male (female unknown). Surstylus with four spines (Fig. 222). Distiphallus broad, short, and very dark (Figs 223, 224). Dark, sclerotised hypophallus present	***A.soka* Eiseman & Lonsdale**
–	Only 2 or 3 well-developed postsutural dorsocentrals, with third seta (if present) usually much shorter than acrostichal. Calypter hairs sometimes brown in male, usually white in female. Surstylus with > 4 spines. Distiphallus pale and bell-shaped in ventral view. Hypophallus absent	**21**
21	Frons and most of gena orange-brown, or at least distinctly paler than scutum; fronto-orbital plate and posterior region of frons variably dark brown (frons appearing entirely dark in poorly preserved specimens). Lunule light brown to yellow. Frons buckled anteriorly, with anterior region visible in lateral view. Distiphallus bulging subapically	**22**
–	Frons, gena and lunule entirely dark brown, with frons evenly curved to meet face. Sides of distiphallus relatively straight or concave subapically	**23**
22	Fronto-orbital plate always dark brown to level of anterior or posterior ors, and sometimes dark along most of lateral margin. Distiphallus strongly swollen distally, almost circular in shape (viewed ventrally) (Fig. 214)	***A.parvicornis* Loew**
–	Fronto-orbital plate usually brownish orange around base of all fronto-orbital setae, but sometimes as above. Distiphallus only gradually swollen subapically, with distal section only slightly wider than basal section (viewed ventrally) (Fig. 215)	***A.proxima* Spencer**
23	Antenna dark brown with base of first flagellomere sometimes orange. Basiphallus produced distolaterally (i.e., apex split); without membranous lateromedial lobes (Figs 219, 220). Distiphallus narrowest medially	***A.pudica* Spencer**
–	Antenna dark brown, but if first flagellomere paler, then entirely concolourous on outer face. Basiphallus not produced distolaterally; with 1 pair of membranous lateromedial lobes. Distiphallus narrowest apically	**24**
24	Apical tuft of pale hairs present on apex of male first flagellomere. Calypter hairs white to brown. Mesophallus with 1 pair of small, lateral membranous lobes at midpoint. Distiphallus relatively pale, not angled dorsally and with apical section (seen ventrally) irregular in outline (Figs 203, 204)	***A.parca* Spencer**
–	Apical tuft of hairs absent from male first flagellomere. Calypter hairs dark brown. Mesophallus produced laterally into 1 pair of large, triangular, basally sclerotised wings. Distiphallus relatively dark, slightly angled dorsally and with distal section (seen ventrally) evenly tapered (Figs 208, 209)	***A.parilis* Spencer**

#### Species descriptions

Unless otherwise stated, species are characterised in part as follows: body and setae dark brown with ocellar triangle always darker; gena highest posteriorly; notum subshiny; stridulatory organ present; one pair of well-developed acrostichal setae present; acrostichal setulae in eight to ten rows; costa extending to vein M_1+2_; abdomen dark brown.

##### Agromyza abiens

Taxon classificationAnimaliaDipteraAgromyzidae

Zetterstedt

Figs 139–142

Agromyza abiens Zetterstedt, 1848: 2747. Hendel 1931: 147 [as synonym of rufipes Meigen]; Spencer 1963d: 3 [stat. reinst.], 1972: 35, 1976: 88.
Agromyza echii Kaltenbach, 1860: 217. Spencer 1963d [synonymy].

###### Description.

Wing length 3.6–4.1 mm (♂), 4.3 mm (♀). Length of ultimate section of vein M4 divided by penultimate section: 0.6–0.7. Eye height divided by gena height: 3.6–5.3 (up to 10.0 in German material examined). First flagellomere slightly elongate with distal margin covered with tuft of dense, pale hairs. Fronto-orbital plate and parafacial slightly produced, curving under eye as distinct cheek. Ocellar triangle hemispherical in outline. Epistoma relatively large to narrow. Thorax and abdomen with light greyish pruinosity.

***Chaetotaxy***: Three ori, two ors. Four to six dorsocentrals (four in Nearctic specimen), slightly decreasing in size anteriorly (more so presuturally). Mid tibia with two posteromedial setae.

***Colouration***: Body dark brown with halter white and gena and parafacial light brown; lunule, pedicel, scape and frons (between narrow fronto-orbital plates) brownish orange; first flagellomere orange with deeper brown tint past base, sometimes mostly brown; palpus brownish; posterior 1/3–1/2 of frons sometimes darker; ocellar triangle dark brown, distinct, slightly larger than tubercle; notopleuron slightly paler than rest of notum. Calypter margin and hairs white. Examined European material with paler parts of head lighter orange, parafacial and posterior parts of frons darker, thorax reddish, palpus reddish orange and lunule yellow. Wing veins light brown; coxae and femora paler brown with apices of latter yellow; tibiae and tarsi light brownish orange.

***Genitalia***: (Figs 139–142) Surstylus entirely fused to epandrium and with several tubercle-like setae on inner face (concentrated posteriorly). Hypandrium produced at apex. Postgonite broad and flat apically with inner-medial swelling. Phallophorus elongate and narrow, interlocking and partially fused to basiphallus. Left 1/2 of basiphallus short, arising apically from phallophorus; right 1/2 of basiphallus broad basally and with thin curved apical section; apices of both halves abruptly converging. Hypophallus small and membranous with lightly sclerotised medial section. Mesophallus not much longer than wide, rounded and slightly flattened basally, and with several basal, lateral and anteromedial extensions; distiphallus separate, not much longer than wide, cup-like with internal fringe of hairs and basal 1/2 more abruptly narrowed. Ejaculatory apodeme asymmetrical, with distal margin irregular and medial rib offset and annulated.

###### Hosts.

Boraginaceae – *Aegonycho*, *Amsinckia* (?), *Anchusa*, *Asperugo*, *Borago*, *Brunnera*, *Buglossoides*, *Cerinthe*, *Cynoglossum*, *Echium*, *Lappula*, *Lindelofia*, *Lithospermum*, *Lycopsis*, *Myosotis*, *Nonea*, *Omphalodes*, *Onosma*, *Pentaglottis*, *Pulmonaria*, *Podonosma*, *Pulmonaria*, *Solenanthus*, *Symphytum* (Spencer 1976; Benavent-Corai et al. 2005; Ellis 2021).

###### Distribution.

**USA**: VA*. Europe.

###### Type material.

***Syntypes* [*abiens*]: Sweden.** Scania ad Tranas, Esperod, Ostra Torp. [Not examined – one ST labeled as lectotype (Spencer, 1963d)]

***Syntypes* [*echii*]: Germany** [not given]. [Not examined]

###### Material examined.

**England.** Hampstead, 26.vi.1954, em. 2.viii.[19]54, CNC352640 (1♀, CNC). **Germany.** Thuringen: H. Buhr, mine an Cynoglossumofficinale, vii.1964, Hering, CNC352639, No. 2034 (1♂ 1♀ [with puparia], CNC). **Europe.** [illegible], 20.vii.1953, em. 11.viii.[19]53, CNC352638 (1♂, CNC), [illegible] viii.1908 (2♂ 1♀, USNM). **USA. VA**: Strausberg, 21.ix.1980, W.H. Rowe (1♂, USNM).

###### Comments.

The Virginia male recorded here is the first Nearctic instance of this otherwise European species, which feeds on a wide range of Boraginaceae. The irregular, uneven basiphallus, the distinct mesophallus and the cup-like distiphallus with an inner fringe of hairs are characteristic. Externally, this species is relatively large with a white male calypter, it has five fronto-orbitals, a relatively pale head and the apical third of the first flagellomere is densely covered in pale hairs. Morphology is incredibly similar to *Agromyzapseudoreptans* Nowakowski, which also occurs in Europe and North America and may occur in the Delmarva states, but the distiphallus is distinctly longer than wide (not as long as wide) and is similarly constricted dorsoapically (not broadly domed, as illustrated in previous works).

##### Agromyza albipennis

Taxon classificationAnimaliaDipteraAgromyzidae

Meigen

Figs 34 , 143–146

Agromyza albipennis Meigen, 1830: 171. Spencer 1969: 32; Spencer and Steyskal 1986b: 262; Černý et al. 2020: 194.
Agromyza dubitata Malloch, 1913a: 311. Frick 1953: 68 [as synonym reptans Fallén], 1957: 199 [as synonym nigripes Meigen]. Spencer and Steyskal 1986b [syn.].
Agromyza albo-hyalinata Zetterstedt, 1848: 2742. Griffiths 1963 [synonymy].
Agromyza albipennis fennica Griffiths, 1963: 128. Spencer 1976 [synonymy].

###### Description.

Wing length 2.1–2.9 mm (♂), 2.5–3.5 mm (♀). Length of ultimate section of vein M4 divided by penultimate section: 0.6–0.8. Eye height divided by gena height: 3.6–5.6. First flagellomere relatively elongate with dorsoapical corner slightly pointed; velvety with small apical tuft of dense pale hairs (only distinct when viewed anteriorly). Fronto-orbital plate and parafacial slightly pronounced, curving under eye as cheek. Ocellar triangle reaching level of anterior ors, sides concave.

***Chaetotaxy***: Two ors, two or three ori (if three, anterior seta smaller). Four postsutural dorsocentrals, anterior two much shorter. Mid tibia with two posteromedial setae.

***Colouration***: Body black with halter white and wing veins paler and fore knee sometimes very narrowly yellow. Calypter margin and hairs white. Faint greenish shine sometimes evident, mostly on abdomen.

***Genitalia***: (Figs 143–146) Surstylus broad and triangular, entirely fused to epandrium, margin darkly pigmented, setae clustered at apex and inner margin with several medial to subapical tubercle-like setae. Inner lobe of hypandrium with furrows leading to base of setae. Postgonite small, flat, lobe-like. Halves of basiphallus converging to point of fusion at base; apices abruptly converging; right 1/2 with long membranous extension at midpoint. Distiphallus of “*nigripes*-type” (capsule-shaped with subbasal opening for entry of ejaculatory duct, pronounced dorsobasal collar, and medial convolution), with sides parallel medially, one pair of elongate parallel dark ventromedial swellings, and a slightly broadened apical margin with inner surface minutely spinulose; slightly angled dorsally.

###### Hosts.

Poaceae – *Triticumaestivum* (only known North American host), *Agrostis*, *Arrhenatherum*, *Brachypodium*, *Bromus*, *Calamagrostis*, *Dactylis*, *Deschampsia*, *Festuca*, *Glyceria*, *Hordeum*, *Milium*, *Phalaris*, *Phalaroides* (Ellis 2021), *Phleum*, *Poa*, *Secale*, *Setaria*. In Europe, most commonly occurring on *Phalariaarundinacea*, *Hordeum* and *Poa* (Spencer and Steyskal 1986b; Benavent-Corai et al. 2005).

###### Distribution.

**Canada**: AB, BC, MB, NB*, NL, NS*, NT, NU*, ON, QC, SK*, YT*. **USA**: AK, CA, CO, DC*, IA, IL, MA, MD*, MI, NC*, NY, OH, PA*, SC*. Widespread in Palaearctic (Černý et al 2020).

###### Type material.

***Syntype* [*albipennis*]: Austria** [not given] (1♀, NMW). [Not examined].

***Syntype* [*dubitata*]: USA. MA**: Beverly (1♀, USNM; two females reported in Spencer and Steyskal 1986b).

###### Paratypes examined

[***dubitata*]: Canada. QC**: Beaulieu, Ile de Montreal, 14.vii.1906 (1♀, USNM), Ottawa [illegible], Beaulieu, 20.vii.1912, “13” (1♀, USNM), Cottage Beaulieu, Beaulieu, 16.vii.1906, “15” (1♀, USNM), Cottage Beaulieu, Beaulieu, 21.viii.1906, “19” (1♀, USNM), Cottage Beaulieu, Beaulieu, 10.vi.1906, “21 0” (1♀, USNM). **USA. MA**: Beverly, 23.viii.1969 (1♀, USNM).

***Syntypes* [*albo-hyalinata***]: “Ovasa ad Esperod Scaniae… Dania” (? ZIL). [Not examined].

***Holotype* [*albipennisfennica*]: Finland.** Messuby (1♂, UZMH). [Not examined].

###### Additional material examined.

**Canada. AB**: Black Foot Hills, 9.viii.1940, A.R. Brooks, CNC352861 (1♀, CNC), Lethbridge, 27.vi.1923, H.L. Seamans, CNC352860 (1♀, CNC), Banff Natl. Pk., Lk. Louise, 1402 m, 7.vii.1955, R. Coyles, CNC352693 (1♀, CNC), Banff, Jasper Hwy, Sunwapta Pass, 2011 m, 7.vii.1955, R. Coyles, CNC352692 (1♀, CNC), Elkwater, 11.vi.1956, CNC352652 (1♂, CNC), Onefour, 49°6'0"N, 110°24'0"W, 4.vi.1955, J.R. Vockeroth, CNC352653, CNC352654 (2♂, CNC), Elk Island N.P. Wood Bison Trail wetland alongside aspen forest, lots of tall grass, 53.5666°N, 112.8514°W, 722 m, BIOBus, 1.vii.2012, BIOUG06731-D05 (1♂, CNC), Waterton Lakes NP, Highway 6 pulloff east of 2 Flags Lookout montane forest, douglas fir and lodgepole pine (mostly conifer with aspen/birch understory), 49.065°N, 113.7781°W, 1562 m, BIOBus, 11.viii.2012, BIOUG06502-C07 (1♀, CNC), **BC**: Liard Hot Spg. Mi496 Alaska Hwy, 457 m, 9–10.vii.1959, E.E. MacDougall, CNC352698 (1♀, CNC), Moosehorn Lake, 58°10'0"N, 132°7'0"W, 1371 m, 25.vii.1960, R. Pilfrey, CNC352658 (1♂, CNC), Summit L., Mi392 Alaska Hwy, 1402 m, 16.vii.1959, E.E. MacDougall, CNC352699 (1♀, CNC), Terrace, 10.vi.1960, 5.vi.1960, C.H. Mann, CNC352655, CNC352656, CNC352657, CNC352697 (3♂ 1♀, CNC), Vernon, 3.ix.1931, R.D. Bird, CNC352651, 2s37, IV 31 (1♂, CNC), Bobson, 6.v.1952, H.R. Foxlee, CNC352862 (1♀, CNC), Oliver, 20.v.1923, C.B. Garrett, CNC352863 (1♀, CNC), Vernon, 31.vii.1937, H. Leech, CNC352864 (1♀, CNC), Victoria, 20.v.1919, W. Downes, CNC352844 (1♂, CNC), **MB**: Minnedosa, 5mi N, 8.vii.1958, R.L. Hurley, CNC352690 (1♀, CNC), Shilo, 5mi SW, 28.v.1958, R.B. Madge, CNC352663 (1♂, CNC), Western MB, Riding Mountain Nat. Pk. – North Escarpment Trailhead wet meadow/marsh, 50.674°N, 99.65°W, 726 m, J. Straka, J. Crossey, 15.viii.2008, 08BDIP-1973 (1♀, CNC), Aweme, 26.vi.1916, N. Criddle, CNC352842 (2♂, CNC), 28.viii.1917, CNC352843 (1♂, CNC), **NB**: Kouchibouguac N.P., 8.vii.1977, J.F. McAlpine, Code-6020N, CNC352688, CNC352689 (2♀, CNC), **NL**: Gros Morne; Gros Morne Mountain Hiking Trail, 49.5657°N, 57.8324°W, 39 m, J. Crossey, R. Labbee, A. Smith, M. Zhang, 15.vii.2009, 09BBEDI-0938 (1♀, CNC), St. John’s, Agric. Exp. Sta., 12.vii.1967, 15.vii.1967, J.F. McAlpine, CNC353033, CNC353041, CNC353042 (2♂ 1♀, CNC), **NS**: Kentville, 22.viii.1912, from tortricid larva on apple, [illegible], CNC352650 (1♂, CNC), **NT**: Ft. McPherson, 19.vii.1957, R. Hurley, CNC352695 (1♀, CNC), **NU**: [N.W.T.] Muskox L., 64°45'0"N, 108°10'0"W, 2.viii.1953, J.G. Chillcott, CNC352696 (1♀, CNC), **ON**: Almonte, 18.v.1951, J.F. McAlpine, CNC352679 (1♀, CNC), Hailerbury, 12.viii.1947, G.S. Walley, CNC352646 (1♂, CNC), Marmora, 1.vi.1952, 13.v.1952, J.C. Mitchell, J.R. Vockeroth, CNC352674, CNC352675, CNC352676 (3♀, CNC), Maynooth, 25.v.1951, J.F. McAlpine, CNC352677 (1♀, CNC), Minnedosa, 5mi N, 8.vii.1958, R.L. Hurley, CNC352691 (1♀, CNC), Normandale, 42°42'0"N, 80°19'0"W, 27.v.1956, J.R. Vockeroth, CNC352645 (1♂, CNC), 29.v.1956, J.R. Lonsway, CNC352678 (1♀, CNC), Ottawa, 8.vii.1952, G.E. Shewell, CNC352647 (1♂, CNC), Port Hope, 25.v.1925, N.K. Bigelow, CNC352680 (1♀, CNC), Port Severn, 3mi N, 18.v.1959, J.G. Chillcott, CNC352681 (1♀, CNC), Putnam, 26.vi.1925, G.S. Walley, CNC352644 (1♂, CNC), St. Williams, 42°40'0"N, 80°25'0"W, 23.v.1956, J.R. Vockeroth, CNC352649 (1♂, CNC), Turkey Pt., 42°39'0"N, 80°21'0"W, 25.v.1956, J.R. Vockeroth, CNC352683 (1♀, CNC), Brockville, 5.viii.1903, W. Metcalfe, CNC352847 (1♀, CNC), Cottage Beaulieu, 16.vii.1906, 145, CNC352854 (1♀, CNC), Mer Bleue, 10.v.1938, G.E. Shewell, CNC352834 (1♂, CNC), Norway Point, Lake of Bays, 31.vii.1919, J. McDunnough, CNC352851 (1♀, CNC), Orillia, 18.vii.1923, C.H. Curran, CNC352848 (1♀, CNC), 17.viii.1923, CNC352850 (1♀, CNC), Ottawa, 15.vii.1938, A. Brooks, CNC352830 (1♂, CNC), Ottawa, 11.ix.1947, G.E. Shewell, CNC352829 (1♂, CNC), Ottawa, A. Brooks, 5.vii.1938, CNC352852 (1♂, CNC), Ottawa, 27.vii.1912, Beaulieu, CNC352853 (2♂ 2♀, CNC), Pt. Ryerse, 1.vi.1939, G.E. Shewell, CNC352835 (1♂, CNC), St. Johns, Little Montreal River, 9.vii.1937, G.E. Shewell, CNC352855 (1♀, CNC), Simcoe, 8.vi.1939, G.E. Shewell, CNC352849 (1♀, CNC), Simcoe, G.E. Shewell, 14.vi.1939, CNC352831 (1♂, CNC), Simcoe, G.E. Shewell, 22.vi.1939, CNC352832 (1♂, CNC), Simcoe, G.E. Shewell, 29.v.1939, CNC352833 (3♂ 1♀, CNC), Metcalfe, 2mi N, 10.vi.1982, B.E. Cooper, CNC353037 (1♂, CNC), Metcalfe, 25.v.1983, B.E. Cooper, CNC353038 (1♂, CNC), Ottawa, damp second-growth Acer-Betula wood, 15.viii.2003, 20.vii.1974, 29.vi.1991, J.R. Vockeroth, CNC353032, CNC353035, CNC353036 (2♂ 1♀, CNC), Port Severn, 3mi N, black spruce bog, 18.v.1959, J.G. Chillcott, CNC353039, CNC353034 (2♂, CNC), **QC**: Ile de Montreal, 7.vii.1906, Beaulieu, CNC352837 (1♂, CNC), Old Chelsea, 13.vi.1961, J.R. Vockeroth, CNC353040 (1♂, CNC), St. Johns, Little Montreal River, 9.vii.1937, G.E. Shewell, CNC352836 (1♂, CNC), Wakefield, 9.vii.1946, G.E. Shewell, CNC352838 (1♂, CNC), Woburn, 19.vi.1923, C.H. Curran, CNC352859 (1♀, CNC), Aylmer, 16.vii.1959, C.H. Mann, CNC352684 (1♀, CNC), Farnham, 5.vi.1963, J.R. Vockeroth, CNC352642, CNC352682 (1♂ 1♀, CNC), Hull, 20.vi.1956, J.R. Vockeroth, CNC352685 (1♀, CNC), Indian House L., 20.vii.1954, 27.vi.1954, 27.vii.1954, R. Coyle, R. Coyles, W.R. Richards, CNC352648, CNC352686, CNC352687 (1♂ 2♀, CNC), Knowlton, Bolton Pass, 243 m, 5.vi.1963, J.R. Vockeroth, CNC352641 (1♂, CNC), Mt. Orford, 365 m, 5.vi.1963, J.R. Vockeroth, CNC352643 (1♂, CNC), Abbotsford, G. Shewell, 29.v.1936, CNC352839, CNC352858 (1♂ 1♀, CNC), Abbotsford, 10.viii.1937, G.E. Shewell, CNC352840 (1♂, CNC), Abbotsford, G. Shewell, 20.viii.1936, CNC352841 (1♂, CNC), 30.v.1936, CNC352857 (1♀, CNC), Abbotsford, 20.v.1931, J.B. Maltais, CNC352856 (3♂ 3♀, CNC), **SK**: Christopher L., 27.viii.1948, A.R. Brooks, CNC352705, CNC352706 (2♀, CNC), Val Marie, 49°15'0"N, 107°44'0"W, 5.vi.1955, 9.vi.1955, J.R. Vockeroth, CNC352659, CNC352662, CNC352664, CNC352665, CNC352666, CNC352700, CNC352701, CNC352702, CNC352703, CNC352704, CNC352707 (5♂ 6♀, CNC), Grasslands National Park, Visitor’s Center grass cattle pasture, 49.246°N, 107.732°W, 812 m, J. Cossey, N. Jeffery, J. Straka, 14.vii.2008, 08BBDIP-2742 (1♂, CNC), Saskatoon, 12.vii.1940, 22.vii.1939, 25.v.1926, 25.viii.1923, 28.ix.1925, sf. wheat, A.P. Robinson, K.M. King, King, 13288; D679; ‘41, 1328B; D676; ‘41, 1328B; D677, 1328B; D678, 16410- 3N9B, 16423 1229B; D348, 16425 26BS5; D350, 63AN, D663, CNC352845, CNC352846, CNC352865, CNC352866, CNC352867, CNC352868, CNC352869, CNC352870, CNC352871 (2♂ 7♀, CNC), **YT**: Otter Lake, 1219 m, 15.vii.1960, J.E. H. Martin, CNC352694 (1♀, CNC). **USA. AK**: Anchorage, 18.vi.1951, 3.vii.1951, IDEMA Illustration, R.S. Bigelow, CNC352668, CNC352720 (1♂ 1♀, CNC), Fairbanks, 13.vi.1952, J.B. Bartley, CNC352669 (1♂, CNC), Mackenzie Delta, Reindeer Depot, 30.vi.1948, J.R. Vockeroth, CNC352661 (1♂, CNC), Umiat, 13.vii.1959, 24.vii.1959, 5.vii.1959, 6.vii.1959, 7.vii.1959, 7.viii.1959, 9.viii.1959, IDEMA Illustration, J.E.H. Martin, R. Madge, CNC352660, CNC352667, CNC352673, CNC352712, CNC352713, CNC352714, CNC352715, CNC352716, CNC352717, CNC352718, CNC352719 (3♂ 8♀, CNC), **CO**: Walden, 11.viii.1965, G.F. Knowlton (1♂, USNM), Doolittle Ranch, 9800', Mt. Evans, 9.vii.1961, C.H. Mann (1♂, USNM), Doolittle Ranch, Mt. Evans, 2987 m, 3.viii.1961, 9.vii.1961, C.H. Mann, CNC352670, CNC352708 (1♂ 1♀, CNC), Boulder, 5500', 5.vi.1961, B.H. Poole (1♀, USNM), Mt. Evans, 3535 m, 11.vii.1961, C.H. Mann, CNC352711 (1♀, CNC), Mt. Evans, 3566 m, 22.vii.1961, B.H. Poole, CNC352710 (1♀, CNC), Nederland, 2529 m, 5.vii.1961, J.G. Chillcott, CNC352672 (1♂, CNC), Jackson Co., Rabbit Ears Pass, 7.vii.1961, J.G. Chillcott, CNC352671, CNC352709 (1♂ 1♀, CNC), **DC**: Theo Roosevlt Id, 4.vi.1977, W.N. Mathis (1♂, USNM), **MA**: Boston, June (1♂ 1♀, USNM), Concord, 19.vii.1961, marsh, W.W. Wirth (2♂ 3♀, USNM), Forest Hills, 21.ix.1913, A.L. Melander (2♀, USNM), **MD**: Colesville, 4.vii.1976, W.W. Wirth (2♀, USNM), Colesville, 14.vi.1975, Malaise trap, W.W. Wirth (1♀, USNM), **NC**: Macon Co., Highlands, Lake Ravenel, 7.vi.1986, Malaise trap, W.W. Wirth (1♂, USNM), **NY**: Ithaca, 15.viii.1926, A.L. Melander (1♂, USNM), Geneva, 28.v.1914, A.L. Melander (1♂, USNM), Long Island, Cold Spring Harbor, A.L. Melander (1♂ 2♀, USNM), Allegany State park, 28.v-3.vi.1963, mossy woods, W.W. Wirth (1♀, USNM), **PA**: Mineral Spr., 5.ix.1927, A.L. Melander (1♂, USNM), Chester Co., Avondale, Stroud Res. Ctr., 28.ix.2006, K. Styer (1♀, UDCC), **SC**: Black Falls, 7.viii.1953, A.L. Melander (1♀, USNM).

###### Comments.

See comments for *Agromyzaaprilina*.

##### Agromyza ambrosivora

Taxon classificationAnimaliaDipteraAgromyzidae

Spencer

Figs 147–151

Agromyza ambrosivora Spencer, 1969: 35. Spencer and Steyskal 1986b: 64; Scheffer et al. 2007: 770; Scheffer and Lonsdale 2018: 85; Eiseman and Lonsdale 2018: 7.

###### Description.

Wing length 2.3–2.7 mm (♂), 2.7–3.9 mm (♀). Length of ultimate section of vein M4 divided by penultimate section: 0.5–0.7. Eye height divided by gena height: 2.9–3.6. First flagellomere slightly longer than wide with apex broadly rounded; without tuft of pale hairs. Fronto-orbital plate and parafacial slightly projecting. Ocellar triangle broadly rounded and short, not extending much past ocelli. Buccal cavity subquadrate with anteromedial margin emarginate.

***Chaetotaxy***: Four or five thinner ori with anterior seta sometimes shorter; one ors. Four postsutural dorsocentrals, decreasing in length anteriorly. Mid tibia with two posteromedial setae. Female sometimes with strong additional seta between acrostichal seta and posterior dorsocentral.

***Colouration***: Body predominantly brown, halter white. Gena (excluding ventral margin), parafacial and frons (excluding posterolateral corner, posterior margin and ocellar triangle) light brownish orange. Scape and pedicel yellow; first flagellomere orange with distal 1/2–2/3 brownish. Lunule orange with yellowish pilosity. Face brown. Palpus and clypeus dark brown. Tarsi yellow (slightly darker on posterior legs), fore tibia light brown and knees narrowly yellow. Calypter margin and hairs white. Female with frons darker (sometimes entirely brown or with anterior margin lighter), gena darker and fore tibia darker medially.

***Genitalia***: (Figs 147–151) Surstylus triangular, entirely fused to epandrium; with apical setae and medial tubercle-like setae. Hypandrium long and thin with narrow apical point. Postgonite lobate, directed apically. Phallophorus produced apically and fused to base of basiphallus. Halves of basiphallus widely bowed with bases broadly fused; elongate and narrow with margins irregular. Distiphallus very long, thin, dark, bifid, and strongly curved into semicircle at split; base of distiphallus with transverse dorsal band and paired ventral sclerites. Hypophallus large, membranous, and directed apically. Ejaculatory apodeme dark and very reduced in size.

***Variation***: Female from CA differs as follows: wing length 4.1 mm; anterior (of four) ori small; only three dorsocentrals; eye height divided by gena height 2.5; tibiae yellow to base and apex; gena mostly pale.

###### Hosts.

Asteraceae – *Artemisia* (?), *Ambrosiaartemisiifolia*, *A.trifida* and probably *A.douglasiana* (Spencer and Steyskal 1986b; Benavent-Corai et al. 2005); *Helianthusannuus* (Eiseman and Lonsdale 2018).

###### Distribution.

**Canada**: ON. **USA**: CA, CO, MA, MD, NY, PA, VA*.

###### Type material.

***Holotype*: Canada. ON**: Pelee, em. 31.vii from leaf-mines on *Ambrosiaartemisifolia*, leg. 8.vii.1967, CNC352722 (1♂ [with puparium], CNC).

***Paratypes*: Canada. ON**: Pelee, K.A. Spencer, mine *Ambrosiaartemisifolia*, 15.vii.1967, em. 1–10.viii.1967, CNC352723-CNC352744 (10♂ [5 with puparia], 12♀ [8 with puparia], CNC).

###### Additional material examined.

**Canada. ON**: Belleville, P. Harris, vii.1969, em. 22.ix.1969, CNC352721 (1♀ [with puparium], CNC), Ottawa, damp second-growth Acer-Betula woods, 8.viii.1993, J.R. Vockeroth, CNC353060 (1♂, CNC). **USA. CA**: Carbon Canyon, 14.vi.1977, on *Artemesiad*. (1♀, USNM), **CO**: Chaffee Co., Poncha Springs, South Arkansas River, 8.vii.2015, C.S. Eiseman, *Helianthus*, em. 28.vii-1.viii.2015, #CSE1872, CNC654328–654332 (1♂ 4♀, CNC), **MA**: Worcester Co., Sturbridge, Leadmine Rd., 6.vii.2013, em. 20.vii.2013, C.S. Eiseman, ex *Ambrosiaartemisiifolia*, #CSE725, CNC392679, CNC392680 (2♀, CNC), **MD**: Plummers Isl., 30.v.1913, R.C. Shannon (1♀, USNM), College Park, 7.vii.1935, C.T. Greene (1♂, USNM), Pr. William Co., Woodbridge, 26.vii.1968, J.W. Adams (1♂, USNM), Montgomery Co., Colesville, 26.vi.1977, Malaise trap, W.W. Wirth (2♂, USNM), 30.vi.1977 (1♂, USNM), Colesville, W.W. Wirth, 4.vii.1976 (2♀, USNM), 11.vii.1974 (2♂, USNM), 24.vii.1974 (1♂, USNM), 28.vii.1976 (2♂, USNM), 1.viii.1976 (2♂, USNM), 4mi SW of Ashton, 1.ix.1981, Malaise trap, G.F. and J.F. Hevel (1♀, USNM), Bethseda, G. Steyskal, 6.vii.1970 (2♂, USNM), 7.vii.1970 (1♂, USNM), Allegeny Co., Little Orleans, Little Orleans campground, 4.vi.1999, sweeping, C.R. Bartlett (1♂, UDCC), nr. West Mifflin, Coal Valley Rd. #2, 5.vii.1997, sweeping, C.R. Bartlett (1♀, UDCC), **PA**: Chester Co., Pottstown, Warwick County Park, 10.viii.2014, ex. *Ambrosiatrifida*, em. 2.ix.2014, C.S. Eiseman, #CSE1371, CNC384846 (1♀, CNC), **VA**: Great Falls, 21.vi.1931, A.L. Melander (1♂, USNM), Alexandria, “viii-5”, J.M. Aldrich (1♀, USNM), Fairfax Co., Turkey Run Park, nr. mouth of Turkey Run, 38°57.9'N, 7°09.4'W, Malaise trap, 18–30.v.2007, D.R. Smith (1♀, USNM), Turkey Run Park, 0.3 km W mouth Turkey Run, 38°58'N, 77°09.6'W, Malaise trap, D.R. Smith, river 14–17.v.2006 (1♂, USNM), river trap, 17–24.v.2006 (2♀, USNM), Great Falls Park, swamp trail, 38°59.4'N, 77°15.2'W, Malaise trap, trap #2, 18.iv.2.v.2007, D.R. Smith (1♀, USNM).

###### Comments.

*Agromyzaambrosivora* is an easily recognised species with one pronounced ors and four or five thinner ori. The phallus forms a long, dark bifid tubule, which is very similar to that found in *A.virginiensis* (Fig. 151): these two species can be separated externally using the characters mentioned in the key. A third related species, *A.rudbeckiana* Scheffer and Lonsdale, a leaf-miner of *Heliopsis* and *Rudbeckia* (Asteraceae), has a more evenly arched distiphallus that is only split near the apex, and unlike *A.ambrosivora*, there are two ors and three ori (not one and four) (Scheffer and Lonsdale 2018).

##### Agromyza apfelbecki

Taxon classificationAnimaliaDipteraAgromyzidae

Strobl

Figs 152–155

Agromyza abiens var. Apfelbecki Strobl, 1902: 504.
Agromyza andalusiaca Strobl, 1906: 380. Hendel 1920 [synonymy].
Agromyza Apfelbecki . Hendel 1920: 117. Hendel 1931: 107.
Agromyza apfelbecki . Spencer 1966c: 172; Papp 1984: 264; Spencer 1990: 253.

###### Description.

Wing length 4.0–4.1 mm (♂), 4.4–4.8 mm (♀). Length of ultimate section of vein M4 divided by penultimate section: 0.4–0.7. Eye height divided by gena height: 2.1–2.4. Ocellar triangle small and rounded. First flagellomere small and rounded; without apical tuft of pale hairs. Fronto-orbital plate slightly projecting and parafacial strongly projecting, partially to strongly continuing under eye as cheek. Wing slightly pointed apically with apex between M1 and R_4+5_. Thorax with light to dense grey pruinosity.

***Chaetotaxy***: Three to seven ori (two in Spanish female); two ors. Six or seven dorsocentrals, two or three presutural, decreasing in size anteriorly. Mid tibia without posteromedial setae.

***Colouration***: Body mostly dark brown. Head yellow with back of head, occiput, clypeus, mentum and ventral margin of gena dark brown, distal 2/3 of first flagellomere brownish, venter of face light brown and frons slightly darker anteriorly. Knees yellowish orange; tibiae and tarsi paler than femora with tibiae slightly darker medially. Scutum with nearly imperceptible metallic green shine in VA female. Calypter margin and hairs white to dark brown. Halter white, rarely with small lateral spot and ventral margin of knob brown (VA female). Abdomen dark brown with indistinct (VA) or strong coating of grey pruinosity, except on brownish orange male terminalia (sometimes with dark dorsomedial spot on epandrium). European specimens often with fronto-orbital plate, face, apex of palpus, parafacial and ring under eye mostly dark, tibiae often yellowish or more orange on distal 1/3, and epistoma usually larger.

***Genitalia***: (Figs 152–155) Epandrium relatively broad and shallow, with suture between it and surstylus barely evident. Surstylus slightly curved anteriorly and with numerous small, pointed, tubercle-like setae along inner-basal/medial surface. Phallophorus tapered distally and partially fused to flat, pale sclerites of basiphallus, which are weakest and irregular along dorsal margin, with right sclerite partially separated from darker, curved apical section. Hypophallus sac-like and membranous with partial sclerotisation along anteromedial surface. Distiphallus nearly cylindrical with irregularly sclerotised margins not entirely meeting ventrally; twisted past midpoint. Ejaculatory apodeme minute.

###### Hosts.

Asteraceae – *Carduus*, *Cirsium*, *Cynara* (Spencer 1990).

###### Distribution.

**USA**: VA*. France, Italy, Malta, Germany, Spain, Croatia (Papp and Černý 2015). Turkey. Chile (Valparaiso, La Cruz; introduced) and Argentina (Valladares 1998; Çöl et al. 2006).

###### Type material.

***Syntypes* [*Apfelbecki*]: Yugoslavia.** Zadar (♂♀, coll. Strobl). [Not examined]

***Syntypes* [*andalusiaca*]: Spain.** “Southern Spain” [= Algreciras?] (?, coll. Strobl). [Not examined]

###### Material examined.

**France.** Perpignan, 10.x.1959, mine Cynara carduuculus, em. 24.v.1959, K.A. Spencer, CNC352745 (1♂ [with puparium], CNC). **Germany**[?]. “Agrom. Apfelbecki Str., det. Hendel” (1♀, USNM). **Spain.** Barcelona, “prat”, 27.iii.1960, K.A. Spencer (1♀, USNM), CNC352746 (1♀, CNC), En Alcachofe, La Cruz, Valpo, Nac. 5.ix.1959, N. Hichins (5♂ 2♀ 1?, USNM). **USA. VA**: Fairfax Co., Turkey Run Park, 0.3 km W mouth Turkey Run, 38°58'N, 77°09.6'W, Malaise trap, 29.iii-25.iv.2007, D.R. Smith (1♀, USNM).

###### Comments.

While difficult to differentiate from a number of Palaearctic taxa, *Agromyzaapfelbecki* is quite distinct from Nearctic *Agromyza* because it is exceptionally large (wing length at least 4.0 mm), there are numerous dorsocentrals (at least six), the epistoma is large and pronounced, the head is predominantly pale (including the palpus and face) and the halter is sometimes maculated. *Agromyzaapfelbecki* is one of only three *Agromyza* known to feed on Asteraceae, and is distributed primarily around the Mediterranean where it often occurs on globe artichoke (Spencer, 1990).

The above Virginia record is the first known occurrence of this species in the Nearctic, where it was likely introduced accidentally on its host plant. As discussed by Dempewolf (2004), *Agromyzaapfelbecki* can be a major pest on artichoke if populations become large enough, particularly to younger plants; the damage caused by larval feeding can vary significantly between years (Spencer 1973), possibly due to climatic conditions, as frost can severely increase mortality among larvae (Ricchello 1928).

##### Agromyza aprilina

Taxon classificationAnimaliaDipteraAgromyzidae

Malloch

Figs 156–160

Agromyza aprilina Malloch, 1915c: 359. Frison 1927: 192 [lectotype design.]; Frick 1957: 199 [as synonym *subnigripes* Malloch], 1959: 358; Spencer 1969: 36; Spencer and Steyskal 1986b: 262 [stat. reinst.].

###### Description.

Wing length 2.2–2.5 mm (♂), 2.5–3.2 mm (♀). Length of ultimate section of vein M4 divided by penultimate section: 0.6–0.8. Eye height divided by gena height: 2.2–3.4. First flagellomere slightly longer than high with rounded dorsoapical point (pronounced to relatively indistinct), without apical tuft of pale hairs. Fronto-orbital plate and parafacial projecting (more so anteriorly), continuing under eye as cheek. Ocellar triangle longer than wide and subshiny. Costa extending slightly past R_4+5_. Smaller females with vein dm-m incomplete.

***Chaetotaxy***: Two ori; three ors (or vice versa), sometimes appearing as four ori and one ors. One presutural dorsocentral and three or four postsutural dorsocentrals, decreasing in length anteriorly. Mid tibia with two posteromedial setae.

***Colouration***: Body dark brown with halter white and gena and frons (aside from fronto-orbital plate and ocellar triangle) reddish, fore knee yellow and tarsi dirty yellow. Calypter margin and hairs white. Female from “Turkey Run, headquarters” with light green metallic shine on thorax, abdomen and pleuron (faintest).

***Genitalia***: (Figs 156–160) Anterodistal margin of epandrium deeply incised before surstylus. Surstylus triangular, fused to epandrium with suture evident; inner-basal margin with several small tubercle-like setae. Hypandrium slightly produced at apex, inner margin of lobe thickened with several setulae. Postgonite small and lobe-like with thin plate-like base. Phallus relatively small, narrow, and weakly sclerotised. Phallophorus tapering apically, fused to halves of basiphallus, which converge to base. Halves of basiphallus with internal fold apically, with distal portion sharply directed inwards; lateral membrane broad, twisted past midpoint. Distiphallus of "*nigripes*-type” (capsule-shaped with subbasal opening for entry of ejaculatory duct, pronounced dorsobasal collar, and medial convolution); nearly carinate ventromedially, distal section with elongate medial split.

###### Host.

Unknown.

###### Distribution.

**Canada**: AB, BC, MB, NB*, ON, QC, SK, YT*. **USA**: IL, MD*, NC*, NH, VA*.

###### Type material.

***Lectotype*: USA. IL**: Cottonwood Grove, Urbana, 16–20.iv.1915 (1♀, INHS). [Not examined]

###### Paralectotype examined.

**USA. IL**: Urbana, 20.iv.1915, Cottonwood, CNC352757, Type No. 2725 (1♀, CNC).

###### Additional material examined.

**Canada. AB**: Banff, C.B.D. Garrett, 8.vi.1922, CNC352760 (1♀, CNC), **MB**: Ninette, J.F. McAlpine, 21.v.1958, CNC352778 (1♀, CNC), 9.v.1958, CNC352755 (1♂, CNC), R.B. Madge, 9.v.1958, CNC352776 (1♀, CNC), Shilo, 5mi SW, J.F. McAlpine, 28.v.1958, CNC352777 (1♀, CNC), **NB**: Kouchibouguac N.P., Hanley and Cooper, 23.v.1977, Code-5113Q, CNC352754, CNC352774, CNC352775 (1♂,2♀, CNC), W.P. Hanley, 20.v.1977, Code-5098B, CNC352753 (1♂, CNC), **ON**: Bell’s Cor[ner]., J.F. McAlpine, 8.v.1951, CNC352766 (1♀, CNC), Marmora, J.F. McAlpine, 23.iv.1952, CNC352751, CNC352768, CNC352769 (1♂ 2♀, CNC), 24.iv.1952, CNC352752, CNC352767 (1♂,1♀, CNC), 25.iv.1952, CNC352772 (1♀, CNC), 28.iv.1952, CNC352770 (1♀, CNC), 29.iv.1952, CNC352773 (1♀, CNC), 9.v.1952, CNC352771 (1♀, CNC), Maynooth, J.F. McAlpine, 24.v.1951, CNC352764 (1♀, CNC), Metcalfe, B.E. Cooper, 14.v.1983, CNC353020, CNC353022 (2♂, CNC), 25.v.1983, CNC353021, CNC353024 (2♂, CNC), 27.iv.1983, CNC353019, CNC353025, CNC353026 (3♂, CNC), 3.v.1983, CNC353027 (1♂, CNC), 30.iv.1983, CNC353018, CNC353023 (2♂, CNC), 7.v.1994, CNC353028–353031 (4♂, CNC), Ottawa, 28.iv.1955, J.R. Vockeroth, CNC352765 (1♀, CNC), Woodroffe, J.G. Chillcott, 30.iv.1952, CNC352763 (1♀, CNC), **QC**: Abbotsford, G.E. Shewell, 27.iv.1936, CNC352747, CNC352748, CNC352750, CNC352758 (3♂,1♀, CNC), 27.iv.1966, CNC352749 (1♂, CNC), Shewell, 15.v.1936, CNC352759 (1♀, CNC), **SK**: Saskatoon, A.R. Brooks, 28.iv.1949, CNC352756 (1♂, CNC), 9.v.1949, CNC352762 (1♀, CNC), **YT**: Dawson, 14 mi E, P.J. Skitsko, 6.viii.1962, 396 m, CNC352761 (1♀, CNC). **USA. MD**: Montgomery Co., Plummers Island, 38°58'N, 77°10'W, Malaise trap, lower trap, 30.iii-22.iv.2006, D.R. Smith and J.W. Brown (1♀, USNM), Cabin John Bridge, 23.iv.1914, R.C. Shannon (1♂, USNM), Plummers Isl., R.C. Shannon, 8.iv.1914 (4♂ 3♀, USNM; 2♂ 2♀, CNC), 12.iv.1914 (1♂, USNM), 5.iv.1914 (1♂, USNM), 3.iv.1914 (1♂ 1♀, USNM), Plummers Island, Rock Run, 25.iii.1914, R.C. Shannon (1♂, USNM), nr. Plummers Isl., 28.iii.1915, R.C. Shannon (2♂, USNM), **NC**: Smokies, Andrews Bald, 9.vii.1914, A.L. Melander (1♂, USNM), **VA**: Fairfax Co., Turkey Run Park, nr. mouth of Turkey Run, 38°57.9'N, 7°09.4'W, Malaise trap, 29.iii-25.iv.2007, D.R. Smith (4♂ 1♀, USNM), Turkey Run Park, nr. headquarters bldg. 38°57.7'N, 77°08.9'W, Malaise trap, 29.iii-17.iv.2007, D.R. Smith (1♀, USNM), Great Falls Park, swamp trail, 38°59.4'N, 77°15.2'W, Malaise trap, trap #1, 18.iv-2.v.2007, D.R. Smith (1♀, USNM).

###### Comments.

The surstylus and epandrium of *Agromyzaaprilina* are characteristic, readily differentiating it from similar species. In the Delmarva states it can be most easily confused for the relatively common *A.albipennis*, but the ventral margin of the first flagellomere is flatter in this latter species, there are four fronto-orbitals (not five), the parafacial is not as pronounced, the setae are darker and the costa extends to vein M_1+2_. The shape of the basiphallus and distiphallus also differ (see Figs 145, 146). While the first flagellomere can appear less pointed in some material, particularly poorly preserved specimens, it is never entirely rounded as noted in Spencer (1969).

*Agromyzaaprilina* can also be potentially mistaken for *Agromyzanigripes* Meigen, but the latter has darker tarsi, the costa ends at vein M_1+2_, and the male genitalia (based on the dissection of Welsh male in the USNM) are much stouter with slight differences in the shape of the basiphallus and distiphallus. The species *A.ambigua* Fallén (North America, Europe), *A.kincaidi* Malloch (North America; Figs 196, 197) and *A.conjuncta* Spencer (widespread in Europe) can also be mistaken for this species, but the characteristic phallus, surstylus and epandrium of *A.aprilina* allows for definitive identification. Other Nearctic *Agromyza* with a pointed first flagellomere and a shortened costa are restricted to the west coast and have a larger, darker distiphallus (Spencer and Steyskal 1986b).

##### Agromyza aristata

Taxon classificationAnimaliaDipteraAgromyzidae

Malloch

Figs 37 , 161–166

Agromyza aristata Malloch, 1915b: 13. Frick 1957: 199; Spencer 1969: 38; Spencer and Steyskal 1986b: 59; Eiseman and Lonsdale 2018: 8; Eiseman et al. 2021: 5.
Phytagromyza aristata . Frick 1952a: 416.
Agromyza ulmi Frost, 1924: 54. Frick 1952a: 375. Frick 1959 [synonymy].
Agromyza marmorensis Spencer, 1969: 48. Syn. nov.

###### Description

**(Fig. 37).** Wing length 2.0–2.1 mm (♂), 2.3–2.4 mm (♀). Length of ultimate section of vein M4 divided by penultimate section: 0.6–0.9. Eye height divided by gena height: 1.9–2.7. First flagellomere subcircular, without apical tuft of pale hairs. Fronto-orbital plate (more so anteriorly) and parafacial strongly projecting and distinctly continuing under eye as cheek. Ocellar triangle relatively small with corners rounded. Palpus relatively ovate.

***Chaetotaxy***: Two ori; two ors. Ocellar seta thinner than postocellar, and sometimes shorter, but never reaching 1/2 length. Three postsutural dorsocentrals, decreasing in length anteriorly; possibly two more anteriorly that are not much longer than surrounding setulae. Acrostichal seta not much longer than surrounding setulae. Four scattered rows of acrostichal setulae. Mid tibia without posteromedial setae.

***Colouration***: Head predominantly light yellow; first flagellomere yellow; back of head brown above foramen and with grey pruinosity dorsally; ocellar triangle dark brown; posterolateral corner and posterior margin of frons brownish, with spot extending laterally to just behind level of anterior ors; clypeus light brown; face white. Thorax dark brown, covered with grey pruinosity, halter white. Calypter margin and hairs white to yellow. Legs yellow with mid and hind coxae brown, at least in part. Abdomen dark brown; epandrium yellow or with orange to brown tint with dark brown dorsal spot; if epandrium yellow, sternite 8 sometimes also yellow with medial spot.

***Genitalia***: (Figs 161–166) Surstylus fused with epandrium with suture obliterated, shape triangular (nearly equilateral), directed inwards, with several setae along posterior margin. Cercus relatively broad and flat with inner surface covered with pointed tubercle-like setae. Hypandrium setulose, slightly sinuate, and inner lobe with medial desclerotisation. Postgonite short. Phallophorus fused to single sclerite of basiphallus, which originates on left side and is twisted dextrally. Hypophallus membranous with faint left lateral sclerotisation. Mesophallus long, narrow, dark, and flat, lying along ventral margin of membrane; distiphallus short, flat and wrapped ventrally (nearly cylindrical), shortest dorsally. Ejaculatory apodeme minute and finger-like.

###### Hosts.

Cannabaceae – *Celtislaevigata*, *C.occidentalis*, *C.pallida* (leaf mine only); Ulmaceae – *Ulmusalata* (leaf mine only), *U.americana*, *U.rubra* (Eiseman et al. 2021).

###### Distribution.

**Canada**: AB, NB*, ON, QC*. **USA**: IL, IN, IA, KS*, MI*, NY, OH, PA, VA, VT; known from leaf mines in AL, AR, CO, CT, FL, GA, MA, MD, MN, ND, NJ, TN, TX, WI.

###### Type material.

***Holotype* [*aristata*]: USA. IL**: Havana, Gleason’s Sand Dune, 30.iv.1914, C.A. Hart and J.R. Malloch (HT ♀, INHS). [Not examined]

###### Paratypes examined

[***aristata*]: USA. IL**: St. Joseph, Salt Fork, 3.v.1914, Type No. 2726, CNC352790 (1♀, CNC), **PA**: Arendtsville, 27.iv.1923, S.W. Frost (3♂, USNM).

***Syntype* [*ulmi*]: USA. PA**: Arendtsville, 22.iv.1923, S.W. Frost (1♂, USNM; type No. 50033).

***Holotype* [*marmorensis*]: Canada. ON**: Marmora, 7.v.1952, J.R. Vockeroth (HT ♂, CNC).

###### Additional material examined.

**Canada. AB**: Onefour, on wild cherry blossom, 2.vi.1956, O. Peck, CNC353069, CNC353072, CNC352809 (2♂,1♀, CNC), **NB**: Curventon, 19.v.1959, W.W. Moss, C-2, CNC352810 (1♀, CNC), **ON**: Almonte, 18.v.1951, J.F. McAlpine, CNC352788, CNC352803–352806 (1♂,4♀, CNC), Marmora, 16.v.1952, J.C. Mitchell, CNC352786 (1♂, CNC), 30.iv.1952, J.F. McAlpine, CNC352802 (1♀, CNC), Marmora, 28.iv.1952, J.F. McAlpine, CNC352787 (1♂, CNC), Metcalfe, 14.v.1994, B.E. Cooper, CNC353066 (1♂, CNC), 17.v.1994, CNC353067 (1♂, CNC), 7.v.1994, CNC353068 (1♂, CNC), Midland, swampy wood, 26.v.1959, J.G. Chillcott, CNC353071 (1♂, CNC), Nippising, 27.ii.1959, #83, S58-1-1381-01, CNC352779 (1♂, CNC), Ottawa nr. Uplands Airport, 22.v.1990, J.M. Cumming, CNC353064 (1♂, CNC), Ottawa, damp second-growth *Acer*-*Betula* wood, 1.vi.1995, J.R. Vockeroth, CNC353063 (1♂, CNC), 28.v.1995, CNC353070 (1♂, CNC), at sap on Acer stump, 10.v.1991, J.R. Vockeroth, CNC353062 (1♂, CNC), bleeding elm, 22.v.1952, J.F. McAlpine, CNC352791 (1♀, CNC), 28.v.1951, J.F. McAlpine, CNC352794 (1♀, CNC), 5.v.1952, B. Hartley, CNC352783 (1♂, CNC), J.G. Chillcott, CNC352783–352795, CNC352780, CNC352792 (2♂,3♀, CNC), 8.v.1952, J. Chillcott, CNC352782, CNC352785, CNC352796–352800 (2♂,5♀, CNC), Pt. Pelee, 3.vi.1929, G.S. Walley, CNC352801 (1♀, CNC), **QC**: Hemmingford, 10.v.1931, J.B. Maltais, CNC352807 (1♀, CNC), Old Chelsea, 8.v.1938, G.E. Shewell, CNC352808 (1♀, CNC). **USA. IL**: White Heath, 30.iv.1916 (1♀, USNM), Champaign, 28.iv.1953, J.F. McAlpine, CNC352789 (1♂, CNC), **IN**: Lafayette, J.M. Aldrich, 4.v.1916 (1♂, USNM), 11.v.1916 (1♀, USNM), **KS**: Manhattan, C.W. Sabrosky, 27.iv.1934 (1♀, USNM), 17.vi.1934 (1♀, USNM), Lawrence, Nat. Hist. Res., 27.iii.1954, J.G. Chillcott, CNC352781 (1♂, CNC), 28.v.1956, J.G. Chillcott, CNC352811–352818 (8♀, CNC), **MI**: Detroit, 24.v.1935, G. Steyskal (1♂, USNM), E Lansing, C. Sabrosky, 2.vi.1934 (1♀, USNM), 29.iv.1942 (1♀, USNM), **NC**: Scotland Co., Laurinburg, St. Andrews University, 18.iv.2017, T.S. Feldman, *Celtislaevigata*, em. ~17.iv.2018, #CSE4418, CNC1144090 (1♀, CNC), **OK**: Payne Co., Mehan, 36.014339°N, 96.996744°W, 5.iv.2016, em. by 11.iv.2017, M.W. Palmer, ex *Ulmusrubra*, #CSE3448, CNC939912 (1♂, CNC), **VA**: Chain Bridge, 23.iv.1922, J.R. Malloch (1♀, USNM), Fairfax Co., Great Falls Park, quarry, 38°59.1'N, 77°14.8'W, Malaise trap, D.R. Smith, 18–23.iv.2007 (2♀, USNM), 13–24.v.2007 (1♀, USNM), **VT**: USA. Vermont: Chittenden Co., South Burlington, Winooski Gorge, 16.v.2015, C.S. Eiseman, *Ulmusrubra*, em. 24.iii-6.iv.2016, #CSE2264, CNC654497–654499 (3♀, CNC).

###### Comments.

The dissected paratype of *Agromyzaaristata* discussed by Spencer (1969) has been examined and the genitalia are identical to those of the male illustrated here, not as illustrated in Spencer (1969). The unusual illustration in Spencer (1969) appears to belong instead to a non-type male in the USNM from Illinois that has had the phallus broken off near the base of the mesophallus. This mistake in interpreting the male genitalia led Spencer to describe *A.marmorensis* in the same revision, stating that while "Not satisfactorily distinguishable from *A.aristata*” based on external characters, the differences in the genitalia were significant enough to separate them. Since it is now clear that the genitalia of the two species are also identical, *A.marmorensis* is here included as a junior synonym of the senior *A.aristata*.

The new records for NB, QC, MI and KS represent new adult records, but these were previously suspected from leaf mines (Eiseman et al. 2021).

##### Agromyza bispinata

Taxon classificationAnimaliaDipteraAgromyzidae

Spencer

Figs 38 , 41 , 42 , 167–174

Agromyza bispinata Spencer, 1969: 39. Spencer and Steyskal 1986b: 263; Eiseman and Lonsdale 2018: 8.

###### Description

**(Figs 38, 41, 42).** Wing length 2.2–2.4 mm (♂). Female unknown [see discussion below]. Length of ultimate section of vein M4 divided by penultimate section: 0.5–0.7. Eye height divided by gena height: 4.2–6.0. Male first flagellomere large (usually at least 30% longer than pedicel, but not much longer than pedicel in a minority of specimens) and varying in shape from elongate and subovate to (more commonly) circular; at least distal 1/2 (usually distal 2/3) covered with long hairs. Ocellar triangle relatively small with corners slightly rounded. Fronto-orbital plate slightly projecting.

***Chaetotaxy***: Two ori; two ors. Three dorsocentrals, decreasing in length anteriorly with anterior seta not much longer than surrounding setulae. Mid tibia with two posteromedial setae.

***Colouration***: Body primarily dark brown with halter white. Base of first flagellomere and distal margin of pedicel orange; orange region sometimes either extending to basal 1/3 of first flagellomere or strongly reduced (particularly if spines on surstylus reduced). Gena sometimes paler, excluding ventral margin. Calypter white with margin sometimes yellowish or slightly brown, and hairs light brown. Base of fore tibia paler or segment paler overall. Tarsi yellow to orange-brown.

***Genitalia***: (Figs 167–173) Surstylus small, lobate, margin darkly pigmented; with two or three medial setae and two large posterior spines; spines sometimes minute and rounded and distiphallus more elongate with relatively pronounced internally haired medial section; surstylus barely visible when viewed laterally and with basal suture obliterated. Hypandrium relatively narrow and tapered apically with membranous window in lobe. Postgonite relatively small and upcurved. Halves of basiphallus converging to, and overlapping at base; with small mediolateral membranous lobe on each sclerite; twisted medially and broad apically, with converging ventrodistal points. Distiphallus of “*nigripes*-type” (capsule-shaped with subbasal opening for entry of ejaculatory duct, pronounced dorsobasal collar, and medial convolution); relatively narrow and pale with sides parallel, being only slightly wider apically.

***Variation***: Phallus sometimes elongate with pronounced medial section with densely spinulose inner surface and elongate medial membranous projection on right sclerite of mesophallus (Fig. 174); if so, spines on epandrium reduced to absent. Possibly a new species, but “typical” males show intermediate degrees of spine reduction on epandrium that resemble the state seen in these other males.

###### Host.

Poaceae – *Elymushystrix* (Eiseman and Lonsdale 2018).

###### Distribution.

**Canada**: ON, MB*. **USA**: CT*, GA, IA, MD, NC, NH*, NY, PA*, UT, VA, WV*.

###### Type material.

***Holotype*: Canada. ON**: Simcoe, 5.vi.1939, G.S. Steyskal (1♂, CNC).

***Paratypes***: **Canada. ON**: Midland, 20.vii.1955, J.G. Chillcott, CNC352821 (1♂, CNC), Pt. Ryerse, 1.vi.1939, G.E. Shewell, CNC352822, (1♂, CNC), Simcoe, 5.vi.1939, G.E. Shewell, CNC352819 (1♂, CNC).

###### Males examined.

**Canada. MB**: Ninette, 28.vii.1958, “sq. fringe darker spines on surs. larger aed. larger”, J.G. Chillcott, CNC352820 (1♂, CNC). **USA. CT**: Redding, 11.vi.1929, A.L. Melander (1♂, USNM), Rabin Co., 13.vii.1957, W.R. Richards, CNC352824 (1♂, CNC), **IA**: Winneshiek Co., 43°25'55.97"N, 92°0'34.78"W, 16.vii.2015, C.S. Eiseman, *Elymushystrix* em. 7.viii.2015 , #CSE1973, CNC564662 (1♂, CNC), **MD**: Bethseda, 30.v.1980, G.C. Steyskal, swept ex. *Violapapilionaceae* (2♂, USNM), Bethseda, 14.v.1981, G.C. Steyskal (1♂, USNM), 11.viii.1981 (1♂, USNM), 14.viii.1981 (2♂, USNM), 16.viii.1981 (1♂, USNM), 1.viii.1981 (3♂, USNM), 17.v.1969 (1♂, USNM), 7.viii.1981 (2♂, USNM), 13.ix.1981 (1♂, USNM), 27.v.1972 (1♂, USNM), Plummers Isl., 19.vi.1913, R.C. Shannon (1♂, USNM), Montgomery Co., Dickerson, 14.vii1974, G.A. Foster (13♂, USNM), 4mi SW of Ashton, G.F. and J.F. Hevel, 16.viii.1986 (1♂, USNM), 24.vii.1982 (1♂, USNM), 27.viii.1981 (1♂, USNM), Cabin John, 20.vi.1931, A.L. Melander (1♂, USNM), **NC**: Macon Co., Wayah Gap, 3800', 29.vii.1957, J.G. Chillcott (1♂, USNM), Jackson Co., Cherokee, 609 m, 25.vii.1957, CNC352823 (1♂, CNC), **NH**: Bretton Wda, 1.vii.1936, A.L. Melander (1♂, USNM), **PA**: Allegeny Co., Little Orleans, Little Orleans campground, 6.vi.1998, sweeping, C.R. Bartlett (1♂, UDCC), **VA**: Falls Church, Holmes Run, 26.vi.1961, light trap, W.W. Wirth (1♂, USNM), Shenandoah, Big. Meadows, A.L. Melander, 1.vii.1939 (1♂, USNM), 2.vii.1939 (2♂, USNM), 5.vii.1939 (1♂, USNM), Great Falls, 9.vii.1926, A.L. Melander (1♂, USNM), Glencarlyn, 2.vi.1925, J.R. Malloch (1♂, USNM), Fairfax Co., Dead Run, 22.vi.1916, R.C. Shannon (1♂, USNM), Fairfax Co., Turkey Run Park, nr. mouth of Turkey Run, 38°57.9'N, 7°09.4'W, Malaise trap, 18–30.v.2007, D.R. Smith (1♂, USNM), **WV**: Parkersburg, 21.vi.1970, G. Steyskal (1♂, USNM), Morgan Co., nr. Great Cacapon, 1.ix.1984, G.F. and J.F. Hevel (1♂, USNM), Hardy Co., Lost River St. Pk., 1–14.viii.1960, K.V. Krombein (1♂, USNM).

###### Females examined.

**USA. CT**: Colebrook, 28.viii.1941, A.L. Melander (1♀, USNM), **MD**: Coleville, W.W. Wirth, 21.v.1977 (1♀, USNM), 11.v.1977 (1♀, USNM), **NC**: Macon Co., Wayah Bald, 1066 m, 13.vii.1957, W.R. Richards, CNC352828 (1♀, CNC), Macon Co., Wayah Gap, 16.vii.1957, J.G. Chillcott, CNC352827 (1♀, CNC), **NH**: Franconia Notch, 8.vii.1931, J.M. Aldrich (1♀, USNM), **NY**: Ithaca, 31.v.1914, A.L. Melander (3♀, USNM), **TN**: Smokies, Chimneys, 25.vi.1941, A.L. Melander (1♀, USNM), **VA**: Falls Church, 18.vii.1960, W.W. Wirth (1♀, USNM), Shenandoah, Big Meadows, A.L. Melander, 3.vii.1939 (1♀, USNM), 2.vii.1929 (1♀, USNM), Shenandoah, Lewis falls, 3.vii.1939, A.L. Melander (1♀, USNM), Fairfax Co., Dead Run, 28.vii.1915, R.C. Shannon (1♀, USNM), Shenandoah Co., Mt. Jackson, 25.v.1962, J.G. Chillcott, J.R. Vockeroth, CNC352825, CNC352826 (2♀, CNC).

###### Comments.

*Agromyzabispinata* belongs to a complex of species defined by an enlarged and long-haired male first flagellomere, whose species appear to be separable only on the basis of male genitalic morphology. The females of these species, which are currently indistinguishable from one another, differ from the males in having an elongate oval first flagellomere with the long pale hairs restricted to the distal margin; the examined females are listed in the material examined section for this species. The sparse, long hairs on the first flagellomere of these species (Figs 41, 42) should not be confused with the discrete apical tuft of hairs characteristic of many other *Agromyza* species, including *A.canadensis*, *A.kincaidi*, and *A.pudica* (see Figs 39, 40). This complex currently includes A.echinalisin the east Nearctic,A.hockingi Spencer in Canada and the western United States (but expected in the eastern United States), and the widespread *A.tacita* and *A.bispinata*. There is a relatively large amount of genitalic variation within the latter two species, including distiphallus morphology in *A.bispinata* and epandrial spine number in *A.tacita*, which suggests the presence of cryptic taxa. The presence of only two epandrial spines, which is likely the derived state, currently defines *A.bispinata* within this group, but more rigorous analysis of the clade as a whole is required to better delineate species.

##### Agromyza canadensis

Taxon classificationAnimaliaDipteraAgromyzidae

Malloch

Figs 1 , 2 , 7–10 , 175–178

Agromyza canadensis Malloch, 1913: 299. Frick 1952a: 372; Spencer 1969: 39, 1990: 198; Spencer and Steyskal 1986b: 59; von Tschirnhaus 1993: 512 [suspected to be conspecific with A.pseudorufipes Nowakowski].

###### Description

**(Figs 1, 2, 7–10).** Wing length 3.1–3.9 mm (♂), 3.3–4.0 mm (♀). Length of ultimate section of vein M4 divided by penultimate section: 0.5–0.7. Eye height divided by gena height: 4.2–7.3. First flagellomere small and rounded; pale tuft of apical hairs varies from teardrop-shaped to encompassing distal 1/3 of segment. Ocellar triangle relatively small with corners rounded.

***Chaetotaxy***: Three or two ori; two ors. Five dorsocentrals (sometimes small fifth seta indistinct), decreasing in size anteriorly. Mid tibia with two posteromedial setae.

***Colouration***: Setae brown. Body predominantly brown with yellowish tint or yellow with brownish tint, with thorax darker and halter white. First flagellomere dark brown to brown and scape and pedicel yellow to brownish; face and lunule dirty yellow in ON specimens; gena (excluding ventral margin), parafacial (excluding inner margin) and anterior margin of postgena light brown in female; postpronotum and notopleuron yellowish in part; lateral and sometimes apical margins of scutellum and postsutural scutum yellow in females and some males. Pleuron paler brown with venter darker and posterior region yellowish; metanotum yellow with brown mottling and dark brown mediotergite; males sometimes darker. Calypter margin and hairs white. Legs yellow with faint brownish tint; tarsi sometimes paler and femora sometimes slightly darker. Abdomen brownish yellow, paler in female with dorsum sometimes brownish and oviscape brown.

***Genitalia***: (Figs 175–178) Surstylus indistinct, fused to epandrium; inner surface with many small, rounded tubercle-like setae not reaching anterior corner. Hypandrium relatively narrow with apex pointed; inner lobe small, rounded and with large separate medial sclerite closely associated with postgonite. Postgonite large and flat with partial fusion to hypandrial band; with sparse hairs on outer face. Halves of basiphallus flat, wide and poorly sclerotised; apices broad, converging past internal subapical fold. Distiphallus very large, resembling a thick band with anterior and posterior margins strongly curled outwards; basal portion with small, shallow lateral sculpturing. Ejaculatory apodeme well-developed basally, but reduced, thin and pale apically.

###### Hosts.

Boraginaceae – *Cynoglossum*, *Mertensia* (Benavent-Corai et al. 2005).

###### Distribution.

**Canada**: ON, QC*. **USA**: CA, VA*.

###### Type material.

***Holotype*: Canada. ON**: Cottage Beaulieu, Beaulieu, 14.viii.1906 (1♀, USNM).

###### Additional material examined.

**Canada. ON**: Hawkeston, 28.vi.1927, C.H. Curran, CNC353079 (1♂, CNC); Mer Bleu[e], 5mi E Ottawa, 9.vi.1966, D.D. Munroe, CNC353082 (1♂, CNC), Ottawa, Montfort Hosp., 17.vii.1993, J.R. Vockeroth, CNC353074 (1♂, CNC), Ottawa, 18.viii.1924, G.S. Walley, CNC353081 (1♀, CNC), damp second-growth *Acer*-*Betula* woods, 17.viii.2000, J.R. Vockeroth, CNC353075 (1♀, CNC), 30.v.1958, J.R. Vockeroth, CNC353078 (1♂, CNC), 30.vi.1958, J.R. Vockeroth, CNC353080 (1♂, CNC); damp second-growth *Acer*-*Betula* woods, 6.viii.1996, J.R. Vockeroth, CNC353073 (1♂, CNC), **QC**: Burnett, 13.viii.1977, J. O’Hara, CNC353076 (1♀, CNC), Old Chelsea, 11.vi.1959, J.R. Vockeroth, CNC353077 (1♀, CNC). **USA. VA**: nr. Plummers Island, 20.v.1914, R.C. Shannon (1♂, USNM), Giles Co., Ripplemead, White Riverbend Pk., 37°19'N, 80°41'W, 9.v.2006, S.A. Marshall (1♀, DEBU), Fairfax Co., Great Falls Park, quarry, 38°59.1'N, 77°14.8'W, Malaise trap, D.R. Smith, 24.iv-2.v.2007 (4♀, USNM), 3–10.v.2007 (12♀, USNM).

###### Comments.

*Agromyzacanadensis* is highly similar to the Palaearctic *A.pseudorufipes* Nowakowski, especially with regards to the derived structure of the phallus. It is also a borage feeder on *Myosotis*, *Podonosma* and *Trigonotis* (Benavent-Corai et al. 2005). Von Tschirnhaus (1993) suggested that the two species could be conspecific, but additional study is required.

##### Agromyza deserta

Taxon classificationAnimaliaDipteraAgromyzidae

(Patton)

Cecidomyiaceltis deserta Patton, 1897: 247. Spencer 1990: 54.
Agromyza deserta . von Tschirnhaus 2016: 153; Scheffer and Lonsdale 2018: 85.

###### Description

**(von Tschirnhaus 2017: figs 6–9).** Wing length 3.4 mm (♂). Female unknown. Length of ultimate section of vein M4 divided by penultimate section: 0.4. Eye height divided by gena height: 4.5. Ocellar triangle not much larger than tubercle. Orbital plate narrow, inner margin weakly defined. Epistoma present, margin straight, as long as width of first flagellomere. Ventral margin of gena relatively straight, shallow anteriorly and deep posteriorly. Scutum densely matt. Costa extending to M1. Tibiae without medial setae

***Chaetotaxy***: Ocellar and postvertical setae strong. Two ori (three ori on right side), decreasing in length anteriorly; two ors. Minute interfrontals anteromedially on frons. Four dorsocentral setae on left side, five on right, decreasing in length anteriorly. Acrostichal seta strong. Acrostichal setulae in six irregular rows. Two or three anepisternal setae, four or five katepisternals.

***Colouration***: Setae dark brown. Mostly dark brown. Face, parafacial, gena and postgena (to point above midpoint of eye) and mouthparts yellowish white with dark brown line along venter of gena; lunule and antenna pale yellow with arista brown; frons light yellow with brownish/orange tint, with ocellar triangle and posterolateral corner of frons dark brown, faded brownish region around ocellar triangle, with brownish stripe along orbital plate almost to base of posterior ori, becoming indistinct anteriorly. Apices of femora and bases of tibiae narrowly yellow. Halter white with stem faintly brownish anteriorly. Calypter margin and hairs brown.

***Genitalia***: (von Tschirnhaus 2017: figs 1–3, 5) Cercus large, bent on outer margin and straight on inner; inner-distal surface with numerous pointed tubercle-like setae. Surstylus fused to epandrium, indistinct, without tubercle-like setae. Hypandrium subtriangular; apex rounded with narrow, pointed apodeme that may be 2 × as long as wide; inner lobe with basal section narrowing to point of attachment to thicker, angled distal section bearing row of several sockets, one of which has a moderately long seta. Postgonite with minute setulae in irregular double row; shape presumably similar to that of *A.aristata*. Phallophorus narrow, width < 1/3 length. Basiphallus dorsally fused to phallophorus on narrow dorsal/left lateral sclerite, which is apically truncated; right lateral sclerite much narrower, but as long as left sclerite. Paraphallus and hypophallus absent. Ejaculatory duct becoming broader along length of basiphallus, thick along mesophallus. Mesophallus a single band-like ventral sclerite partially fused to distiphallus; nearly as long as basiphallus, strongly curved to form a shallow semicircle. Distiphallus very shallow, band-like, and ventrally curved, obliquely angled. Ejaculatory apodeme unknown.

###### Host.

Cannabaceae – *Celtisoccidentalis* (von Tschirnhaus 2016).

###### Distribution.

**USA**: CT, NY, WV.

###### Type material.

***Holotype*: USA. CT**: Orange, gall on twig of *Celtisoccidentalis*, W.H. Patton. [1 gall, Lost]

###### Other material.

**USA. WV**: Morgan County ca. 6 mi NW Hedgesville, 39°37'N, 78°03'W, ex twig swelling gall *Celtisoccidentalis*, coll. 6-v-1998, em. 15-iii-1999, R. J. Gagné (1♂, USNM). [not examined]

###### Comments.

The adult of this species was thoroughly described in von Tschirnhaus (2016), which also provided a discussion on related species, host usage, and treatment in the literature.

##### Agromyza diversa

Taxon classificationAnimaliaDipteraAgromyzidae

Johnson

Figs 179–182

Agromyza diversa Johnson, 1922: 26. Frick 1953: 58; Spencer 1969: 41; Spencer and Steyskal 1986b: 59; Eiseman and Lonsdale 2018: 9.
Liriomyza diversa . Frick 1952a: 402.

###### Description.

Wing length 2.9–3.3 mm (♂), 2.9–3.8 mm (♀). Length of ultimate section of vein M4 divided by penultimate section: 0.6–0.8. Eye height divided by gena height: 6.1–-9.7. First flagellomere sometimes slightly longer than high; with teardrop-shaped tuft of pale hairs at apex.

***Chaetotaxy***: Two ori; two ors. Three or four dorsocentrals, decreasing in length anteriorly. Mid tibia with two or three posteromedial setae.

***Colouration***: Setae black. Body yellow, except as follows: head mostly dark brownish with yellow tint, with mouthparts light yellow, lunule yellow, fronto-orbital plate (narrow) sometimes yellowish to yellow, gena (excluding ventral margin) and face light brown, and ocellar tubercle sometimes yellowish around base of ocellar and postocellar setae; postpronotum, notopleuron and scutellum pale yellow/whitish; halter white; female with oviscape dark brown and face yellow. Calypter margin and hairs white.

***Genitalia***: (Figs 179–182) Surstylus not distinguishable from epandrium; inner face of surstylus flat and covered with numerous small, rounded tubercle-like setae that do not extend to anterior corner. Postgonite relatively large, flat, and broad. Halves of basiphallus long and narrow, with right sclerite bent at midpoint; apices not bent inwards. Hypophallus convoluted and with scalloped apical fringe and irregularly sclerotised base. Mesophallus subcylindrical, with sides bulging and with transverse dorsal band; closely associated with distiphallus, which is bulging medially, truncated apically and with subapical constriction.

###### Host.

Urticaceae – *Laporteacanadensis*.

###### Distribution.

**Canada**: ON. **USA**: IA, IL, IN, IN, MA, MD*, NC, NY, OH, TN, VA, VT.

###### Type material.

***Holotype*: USA. MA**: Chester, 7.viii.1912, C.W. Johnson (1♀, MCZ).

###### Paratype examined.

**USA. IN**: Lafayette, viii.1912, J.M. Aldrich, Paratype No. 50034 U.S.N.M. / CNC Type No. 16287, CNC352872 (1♀, CNC).

###### Additional material examined.

**Canada**. **ON**: Simcoe, 26.vi.1939, G.E. Shewell, CNC352873 (1♀, CNC). **USA. OH**: Delaware Co., Sunbury, Monkey Hollow Rd., 13–15.ix.2014, ex. *Laporteacanadensis*, em. 16–24.x.2014, C.S. Eiseman, #CSE1432, CNC384847, CNC384848 (1♂ 1♀, CNC), **IA**: Ames, Pammel Woods, W.H. Robinson, 24.vi.1970 (2♂, USNM), 9.viii.1969 (1♂, USNM), 19.vi.1970 (1♂, USNM), Ames, Pammel Woods, 11.vi.1970, R.M. Miller (1♀, USNM), Ames, 1.viii.1969, W.H. Robinson (1♂, USNM), Allamakee Co., 3mi ESE Waterville, 43°11.0'N, 91°14.1'W, 2.viii.1960, J. Laffoon (1♂, USNM), Boone Co., Ledges State Park, 14.vii.1971, R.M. Miller (1♀, USNM), **IL**: University of Illinois Woods, 2.vii.1945, “X 526” (1♂, USNM), 15.vii.1945, “X 527” (1♂, USNM), **MD**: Montgomery Co., Dickerson, 14.vii.1974, G. Foster (1♂, USNM), **OH**: Delaware Co., Sunbury, Monkey Hollow Rd., 13–15.ix.2014, em. 16–24.x.2014, C.S. Eiseman, ex *Laporteacanadensis*, #CSE1432, CNC384847, CNC384848 (1♂ 1♀, CNC), **NC**: Graham Co., Poplar Cove, 2500', 35°21.3'N, 83°56.1'W, 20.vi.1958, J. Laffoon (1♀, USNM), **NY**: Ithaca, 15.vi.1932 (1♀, USNM), **TN**: Davidson Co., Nashville, West Meade Waterfall (36.094655, -86.913252), 21.viii.2017, em. 15–17.ix.2017, C.S. Eiseman, ex *Laporteacanadensis*, #CSE4273, CNC939958–939960 (1♂ 2♀, CNC), **VA**: Fairfax Co., Dead Run, 28.vii.1915, R.C. Shannon (1♀, USNM).

##### Agromyza echinalis sp. nov.

Taxon classificationAnimaliaDipteraAgromyzidae

http://zoobank.org/02F82A6C-4335-4C13-B490-994AE0B6BC66

Figs 232–235

###### Description.

Externally as described for *A.bispinata* except as follows: Wing length 2.5–2.6 mm (♂). Female unknown. Length of ultimate section of vein M4 divided by penultimate section: 0.6. Eye height divided by gena height: 4.2. First flagellomere always enlarged in male, broadly ovate, slightly longer than high and covered with long hairs past base. Additional ori sometimes present on one side of frons. Wing veins yellow. Antenna orange with brownish tint, mostly brownish past base of first flagellomere; frons tinted with orange; fore tibia yellow with centre brownish.

***Genitalia***: (Figs 232–235) Surstylus large and triangular with numerous long spines along inner margin and with one spine at apex; basal suture obliterated, inner surface flat and easily viewed posteriorly. Hypandrium and postgonite as described for *A.bispinata*. Halves of basiphallus with medial membranous process that is longer and truncated on right side. Distiphallus of “*nigripes*-type” (capsule-shaped with subbasal opening for entry of ejaculatory duct, pronounced dorsobasal collar, and medial convolution); ventral curve with dark truncated elbow; ventral surface with medial longitudinal suture; sides shallowly rounded and internally spinulose subapically.

###### Host.

Unknown.

###### Distribution.

**USA**: MD, TN, VA.

###### Etymology.

Gr., referring to the characteristic spiny surstylus.

###### Type material.

***Holotype*: USA. TN**: Clarksville, 13.v.1936, E.W. Howe (1♂, USNM).

***Paratypes*: USA. MD**: nr. Plummers Isl., 6.vi.1914, R.C. Shannon (1♂, CNC), **VA**: Fairfax Co., Dead Run, 19.vi.1915, R.C. Shannon (1♂, USNM).

###### Comments.

The heavily spinulose surstylus of *Agromyzaechinalis* is the primary defining feature that distinguishes it from other species with an enlarged first flagellomere. As in *A.tacita* (Figs 226–228) and *A.hockingi* (Colorado, Texas, Utah; Spencer and Steyskal 1986b: figs 411–413), the surstylus is triangular and clearly visible laterally, but these other two species have a slightly darker and more rounded surstylus with a maximum of five stout spines.

##### Agromyza fission

Taxon classificationAnimaliaDipteraAgromyzidae

Eiseman & Lonsdale

Figs 183–187

Agromyza fission Eiseman & Lonsdale, 2018: 9.

###### Description

**(from Eiseman and Lonsdale 2018).** Wing length 2.3 mm (♂). Female unknown. Length of ultimate section of vein M4 divided by penultimate section: 1.0. Eye height divided by gena height: 3.9. First flagellomere small and rounded, without pale tuft of hairs. Fronto-orbital plate slightly projecting (more so anteriorly). Ocellar triangle small and rounded. Thorax with light pruinosity.

***Chaetotaxy***: Two ori; two ors. Three strong dorsocentrals, anterior seta 2/3 length of second. Mid tibia without posteromedial setae.

***Colouration***: Setae black. Body mostly dark brown. Head yellowish orange with first flagellomere infuscated on distal 2/3, back of head and occiput dark brown, frons dark brown behind level of hind fronto-orbital, face white and clypeus brown to dark brown. Calypter margin and hairs brown. Halter white. Tarsi, base of fore tibia and apex of fore femur yellow.

***Genitalia***: (Figs 183–187) Surstylus small, lobate, setulose, and slightly angled anteriorly; fused to anteroventral margin of epandrium with suture partially evident. Cercus broad and emarginate apically; inner surface covered with numerous small tubercle-like setae. Postgonite small and rounded with shallow inner lobe. Single sclerite of basiphallus fused to phallophorus on left side, twisted dextrally; distal margin forming small transverse sclerite. Mesophallus small and flat. Distiphallus composed of a single dark, flat, curved sclerite (shortest dorsally) nearly forming a complete tube; lateral margins fringed and not meeting ventrally.

###### Host.

Cannabaceae – *Celtisoccidentalis* (Eiseman and Lonsdale 2018).

###### Distribution.

**USA**: IA, MD, OK, WI.

###### Type material.

***Holotype*. IA**: Allamakee Co., Red Oak Prairie (43°14'13.43"N, 91°7'8.58"W), 16.vii.2015, em. 2.viii.2015, C.S. Eiseman, ex *Celtisoccidentalis*, #CSE1925, CNC564711 (1♂, CNC).

***Paratypes*. IA**: same collection as holotype, CNC564712 (1♂, CNC), **MD**: Plummers Isl., 23.v.1914, R.C. Shannon (1♂, USNM), **OK**: Payne Co., Mehan, 36.014339°N, 96.996744°W, 5.iv.2016, em. 20– 22.iv.2017, M.W. Palmer, ex *Celtisoccidentalis*, #CSE3529, CNC939918, CNC939919 (1♂ 1♀, CNC); **WI**: Buffalo Co., Alma, S1287 State Road 88, 17.vii.2015, em. 3.iv.2016, C.S. Eiseman, ex *Celtisoccidentalis*, #CSE2311, CNC634841 (1♂, CNC).

###### Comments.

Although largely similar to *Agromyzavarifrons*, the male cerci are highly derived and diagnostic: they are strongly widened apically and deeply cleft, with small, tubercle-like setae covering the inner surface.

##### Agromyza isolata

Taxon classificationAnimaliaDipteraAgromyzidae

Malloch

Figs 188–192

Agromyza isolata Malloch, 1913: 306. Frick 1952a: 373; Spencer and Steyskal 1986b: 264; Eiseman and Lonsdale 2018: 11.
Agromyza albitarsis Meigen. Misidentification. Frick 1959: 353.
Agromyza populoides Spencer, 1969: 52. Spencer and Steyskal 1986b [synonymy].

###### Description.

Wing length 2.3 mm (♂), 2.5–2.7 mm (♀). Length of ultimate section of vein M4 divided by penultimate section: 0.7. Eye height divided by gena height: 4.8–7.5. First flagellomere small and rounded; without pale tuft of apical hairs. Ocellar triangle only indicated laterally as subshiny regions.

***Chaetotaxy***: Two ori; two ors. Four strong dorsocentrals, one presutural. Mid tibia with one posteromedial seta.

***Colouration***: Setae golden-brown, distinct from scutum. Scutum with brownish grey pruinosity. Calypter margin and hairs white (brown in *isolata* holotype). Body predominantly brown with halter white; base of first flagellomere and most of pedicel distinctly orange, entirely dark in *isolata* holotype; BC, CA, and CO material with first flagellomere dark, with basal margin slightly lighter; tarsi, tibiae (hind tibia brownish medially) and apices of femora yellow. Wing veins light brown.

***Genitalia***: (Figs 188–192) Surstylus broad, not distinct from epandrium and with a number of medial tubercle-like setae along inner-distal margin on posterior 1/2. Hypandrium relatively narrow with long, tapered tip. Postgonite small and dome-like. Phallophorus elongate and produced on left side, fusing with single sclerite of basiphallus. Basiphallus short, shallowly curved and restricted to dorsal surface. Mesophallus curved in cross-section, dark, and with long, bifid, tapering “tail”, with bases either separated or fused; distiphallus short, dark, and cap-like. Ejaculatory apodeme relatively small, blade pale.

###### Host.

Salicaceae – *Populus* spp., *Salix* spp. (Eiseman and Lonsdale 2018).

###### Distribution.

**Canada**: AB, BC*, ON, NS*, QC, SK. **USA**: CA, CO, MN [leaf mine only], PA, VA*, VT, WA.

###### Type material.

***Holotype* [*isolata*]: USA. CA**: Eureka, “22.5”, H.S. Barber (1♀, USNM; type No. 17573).

***Holotype* [*populoides*]: Canada. SK**: Regina, em. 23.viii.1965, [ex leaf mine on *Populusdeltoides* ♀ X *P.balsamifera* ♂ (Spencer, 1969)], ex Gracillarid sp., RRD W65, Ex 2616(10), G.N. Still (1♂ [with puparium], CNC).

###### Paratypes examined

[***populoides*]: Canada. ON**: Grand Bend, 20.vii.1939, G.E. Shewell, CNC352879 (1♂, CNC), Ottawa, em. 28.vii.1967, mine Pop. Bals. 10.vii.1967, CNC352880 (1♀ [with puparium], CNC), **SK**: same data as holotype CNC352875–352878, CNC352881-CNC3528884 (4♂ 4♀ [seven with puparia], CNC).

###### Additional material examined.

**Canada. BC**: Terrace, grass, clover and buttercups, 7.vi.1960, R. Pilfrey, sweeping, CNC353092 (1♂, CNC), Wasa Lk., em. 29.vii.1959, “59-6779-01”, “*Populustrichocarpa*”, “R’rd”, CNC353083, CNC353084 (2♂ [with puparia], CNC), **NS**: S. Harbour Bch., PG962943, 6.vii.1983, J.R. Vockeroth, CNC353091 (1♂, CNC), **ON**: Grand Bend, 20.vii.1939, G.E. Shewell, CNC353089 (1♂, CNC), Ottawa, 15.ix.1997, J.R. Vockeroth, CNC353090 (1♂, CNC), 16.ix.1952, CNC353088 (1♀, CNC). **USA. CO**: Doolittle Ranch, Mt. Evans, 2987 m, 3.viii.1961, C.H. Mann, CNC353085 (1♂, CNC), J.G. Chillcott, CNC353087 (1♀, CNC), Summit L. Flats, Mt. Evans, 3901 m, 24.vii.1961, C.H. Mann, CNC353086 (1♂, CNC), **VA**: Falls Church, reared 25.v.1916, *Populus*, host: underside tentminer, J.J. deGryse (1♂ 2♀, USNM), **VT**: Chittenden Co., South Burlington, Winooski Gorge, 29.vi.2014, ex. *Populustremuloides*, em. 15.vii.2014, C.S. Eiseman, #CSE1176, CNC384839–384841 (3♂, CNC), Chittenden Co., Williston, Mud Pond, 28.viii.2016, C.S. Eiseman, *Populusgrandidentata*, em. 18–19.ix.2016 , #CSE3000, CNC654500–654504 (2♂ 3♀, CNC).

##### Agromyza kincaidi

Taxon classificationAnimaliaDipteraAgromyzidae

Malloch

Figs 39 , 40 , 193–197

Agromyza kincaidi Malloch, 1913: 285. Frick 1952a: 372 [as synonym of *ambigua* Fallén]; Spencer 1969: 45; Spencer and Steyskal 1986b: 264.

###### Description

**(Figs 39, 40).** Wing length 3.6–4.0 mm (♂), 3.9–4.3 mm (♀). Length of ultimate section of vein M4 divided by penultimate section: 0.7–0.8. Eye height divided by gena height: 2.9–3.2. First flagellomere small and rounded with small apical tuft of pale hairs. Ocellar triangle 1/2 length of frons, shape equilateral or more tapered apically. Buccal cavity subquadrate and clypeus broad. Costa only extending to R_4+5_. Thorax with light pruinosity.

***Chaetotaxy***: Two ori; two ors. Four postsutural dorsocentrals, one presutural, decreasing in length anteriorly. Mid tibia with two posteromedial setae.

***Colouration***: Body dark brown with halter white. Gena slightly paler posteriorly. Fore tarsus, apex of fore femur and base of tibiae yellowish. Wing veins light brown. Calypter margin and hairs white. Female fronto-orbital plate slightly paler and less pruinose.

***Genitalia***: (Figs 193–197) Surstylus not strongly differentiated from epandrium, which has deep desclerotised anteroventral emargination; with numerous posterior and medial tubercle-like setae and with rounded inner-distal point bearing several small setae. Hypandrium gently arched, not produced apically; inner lobe largely membranous and with two furrows ending in one setula each. Postgonite small, flat and lobate. Halves of basiphallus folded inwards near apex; right sclerite shorter and fused to enlarged distal margin of phallophorus. Distiphallus of “*nigripes*-type” (capsule-shaped with subbasal opening for entry of ejaculatory duct, pronounced dorsobasal collar, and medial convolution); distal portion dark and extremely elongate with wide ventral furrow (dark medial ridge within furrow); inner surface with pronounced spinulose ridge; dorsal surface covered by dark membranous sheath. Ejaculatory apodeme broad and well-developed with distal margin pale and rib pronounced.

###### Host.

Unknown – likely a grass-feeder, possibly on *Bromuspurgans* (Spencer and Steyskal 1986b).

###### Distribution.

**Canada**: AB, BC, MB, NL, NS, YT. **USA**: AK, CA, CO, IA*, NC*, NY*, TN, UT.

###### Type material.

***Holotype*: USA. AK**: Juneau, 25.vii.1899, Kincaid (1♀, USNM; type No. 15565).

###### Additional material examined.

**Canada. AB**: Banff, Johnston Canyon, 1432 m, 18.vii.1962, K.C. Herrmann, CNC352900, CNC352937 (1♂,1♀, CNC), 30–31.vii.1962, CNC352938 (1♀, CNC), Banff, 19.vii.1922, E. Hearle, CNC353102 (1♂, CNC), Calgary, 20 mi W, Jumping Pd. Cr., 9.viii.1962, K.C. Hermann, CNC352901 (1♂, CNC), Wate[illegible], 15.vii.1923, H.L. Seamans, CNC352903 (1♂, CNC), **BC**: Bowser, 8.vi.1955, R. Coyles, CNC352936 (1♀, CNC), Kleanza Cr., 14 mi E Terrace, 4.vii.1960, C.H. Mann, CNC352942 (1♀, CNC), Mt. Thornhill nr. Terrace, 1066 m, in boggy seepage area, 14.vii.1960, J.G. Chillcott, CNC352933 (1♀, CNC), Pr. Rupert, *Ledum*-*Kalmia* bog, 4.iv.1960, R. Pilfrey, CNC352935 (1♀, CNC), 4.vi.1960, C.H. Mann, CNC352896, CNC352897 (2♂, CNC), Robson, 24.v.1950, H.R. Foxlee, CNC352928 (1♀, CNC), Summit Lake, Mi392 Alaska Hwy., 1280 m, 31.vii.1959, R.E. Leech, CNC352898 (1♂, CNC), Summit Lake, 1280 m, 21.vii.1959, R.E. Leech, CNC352899 (1♂, CNC), Terrace, Airport area, 4.viii.1960, C.H. Mann, CNC352934 (1♀, CNC), Terrace, 11.viii.1960, W.R. Richards, CNC352895 (1♂, CNC), 67 m, 15.viii.1960, B. Heming, CNC352929 (1♀, CNC), 67 m, 8.vi.1960, W.W. Moss, CNC352932 (1♀, CNC), 19.vii.1960, C.H. Mann, CNC352931 (1♀, CNC), 20.vii.1960, W.R. Richards, CNC352885 (1♂, CNC), 31.iv.1960, R.J. Pilfrey, CNC352894 (1♂, CNC), 31.v.1960, C.H. Mann, CNC352930 (1♀, CNC), 31.vi.1960, J.G. Chillcott, CNC353099 (1♂, CNC), **MB**: Churchill, 14.vii.1952, E.F. Pope, CNC352939 (1♀, CNC), Churchill, 58°46'N, 94°10'W, 5.viii.1952, J.G. Chillcott, project#NBP, CNC_Diptera59120 (1 ex, CNC), Deer River, mile 473 m Hudson Bay Ry., 3.viii.1952, J.G. Chillcott, CNC352886 (1♂, CNC), Int. Peace Gardens, Turtle Mtn. For. Res., 7.viii.1958, J.G. Chillcott, CNC352940 (1♀, CNC), **NL**: Bell Is., 4–7.viii.1967, J.F. McAlpine, CNC353108 (1♂, CNC), Lab., Cartwright, 20.vii.1955, E.F. Cashman, CNC352941 (1♀, CNC), St. John’s, Agric. Exp. Sta., 1.viii.1967, J.F. McAlpine, CNC353111–353113, CNC353117 (4♂, CNC), 15.vii.1967, J.F. McAlpine, CNC353126 (1♂, CNC), 16.vii.1967, J.F. McAlpine, CNC353124, CNC353110 (1♂,1♀, CNC), on *Ranunculus*, 18.vii.1967, J.F. McAlpine, CNC353103–353107 (5♂, CNC), *Abiesbalsamea*, 19.vii.1967, J.F. McAlpine, CNC353109 (1♀, CNC), 21.vii.1967, J.F. McAlpine, CNC353131, CNC353135 (2♂, CNC), 24.vii.1967, J.F. McAlpine, CNC353119 (1♀, CNC), 26.vii.1967, J.F. McAlpine, CNC353133 (1♂, CNC), 27.vii.1967, J.F. McAlpine, CNC353128 (1♂, CNC), 29.vii.1967, J.F. McAlpine, CNC353120, CNC353129 (1♂,1♀, CNC), 3.viii.1967, J.F. McAlpine, CNC353114–353116 (3♂, CNC), 30.vii.1967, J.F. McAlpine, CNC353121, CNC353125 (1♂,1♀, CNC), 31.vii.1967, J.F. McAlpine, CNC353130 (1♂, CNC), 9.viii.1967, J.F. McAlpine, CNC353118, CNC353122, CNC353123, CNC353127, CNC353132, CNC353134, CNC353136 (4♂,3♀, CNC), **NS**: Lockeport, Cranberry I., 24.vii.1958, J.R. Vockeroth, CNC352902 (1♂, CNC), **YT**: Ogilvie Mts., North Fork Crossing, 1.vii.1962, P.J. Skitsko, CNC352893 (1♂, CNC), Rampart House, 67°25'N, 140°59'W, 17.vii.1951, J.E.H. Martin, project#NBP, CNC_Diptera109349 (1♀, CNC), Whitehorse, 24.viii.1959, R. Madge, CNC352926, CNC352927 (2♀, CNC). **USA. AK**: Anchorage, 18.vii.1951, R.S. Bigelow, CNC352915 (1♀, CNC), Cold Bay, on tundra, 18.viii.1952, W.R.M. Mason, CNC352892 (1♂, CNC), Curry, 29.vi.1952, W.R. Mason, CNC352914 (1♀, CNC), King Salmon, Naknek, 13.vii.1952, J.B. Hartley, CNC352921, CNC352923, CNC352925 (3♀, CNC), 16.viii.1952, CNC352919 (1♀, CNC), 3.vii.1952, CNC352917 (1♀, CNC), 31.vii.1952, CNC352924 (1♀, CNC), 5.viii.1952, CNC352916 (1♀, CNC), 6.vii.1952, CNC352918, CNC352920, CNC352922 (3♀, CNC), Mile 315 Richard. Hwy., 8.vi.1951, W.R. Mason, CNC352913 (1♀, CNC), Naknek, 18.vii.1952, J.B. Hartley, CNC352910, CNC352911 (2♀, CNC), 8.vii.1952, J.B. Hartley, CNC352907 (1♀, CNC), W.R. Mason, CNC352912, CNC352908, CNC352909 (3♀, CNC), **CO**: Doolittle Ranch, Mt. Evans, 2987 m, 22.vii.1961, J.G. Chillcott, CNC352905 (1♀, CNC), 3.viii.1961, W.R.M. Mason, CNC352888 (1♂, CNC), B.H. Poole, CNC352906 (1♀, CNC), Echo L., Mt. Evans, 3230 m, 25.vii.1961, C.H. Mann, CNC352904 (1♀, CNC), **IA**: Ames, 10.v.1947, A.R. Brooks, CNC352943 (1♀, CNC), **NC**: Gt. Smokies, Clingmans Dome, A.L. Melander, 18.vii.1941 (4♂ 3♀, USNM), 19.vii.1941 (4♂, USNM), 21.vi.1941 (1♂, USNM), Smokies, Andrews Bald, 9.vii.1941, A.L. Melander (2♂ 1♀, USNM), Smokies, Forney Ridge, 26.vi.1941, A.L. Melander (1♂, USNM), Clingman’s Dome, Grt. Sm. Mt. Nat. Park, 6.viii.1957, C.J. Durden, CNC352891 (1♂, CNC), 2011 m, 22.viii.1957, J.G. Chillcott, CNC352887 (1♂, CNC), **NY**: White Face Mt., 4000', 14.vii.1958, A.L. Melander (1♂, USNM), **TN**: Indian Gap to Clingman’s Dome, Gr. Sm. Mt. Nat. Park Tenn., 1584–2011 m, 6.viii.1957, J.G. Chillcott, CNC352889, CNC352890 (2♂, CNC).

###### Comments.

*Agromyzakincaidi* is highly similar in appearance to the European and Nearctic *A.ambigua* Fallén (Spencer and Steyskal 1986b: figs 367–369), which has a highly similar phallus, an anterodorsally pointed first flagellomere (sometimes very strongly pointed in *A.ambigua*), a costa that extends just past R_4+5_ and dark colouration. While never directly compared, differentiation between the two has historically relied most heavily on the colour of the calypter margin and hairs, which are supposedly brownish in *A.kincaidi* and bright white to yellow in *A.ambigua* (Spencer 1969); vein colour does not appear to be yellowish in any *A.ambigua* examined, as noted in Spencer and Steyskal (1986b); examination of specimens further reveals calypter margin/hair colour to range from yellowish to brownish, and is even white in some material identified by Spencer. The limits of these two species should be re-evaluated in the future.

##### Agromyza pallidiseta

Taxon classificationAnimaliaDipteraAgromyzidae

Malloch

Agromyza pallidiseta Malloch, 1924: 192. Frick 1952a: 375; Spencer and Steyskal 1986b: 58.

###### Description.

Wing length 2.3–2.4 mm (♀). Male unknown. Length of ultimate section of vein M4 divided by penultimate section: 0.6. Eye height divided by gena height: 1.6. Head as described for *A.aristata* except parafacial not as strongly projecting and ocellar seta 1/2 length of postocellar seta.

***Chaetotaxy***: As described for *A.aristata*, except there are four dorsocentrals (one presutural), not three.

***Colouration***: As described for *A.aristata* except as follows: setae yellow; first flagellomere yellow with basal 1/3 very pale; posterolateral corner of frons only dark to level of posterior (not anterior) ors.

***Variation***: Florida specimen (head mostly collapsed) with ocellar seta ~ 2/3 length of postocellar and fronto-orbital plate brown to base of anterior fronto-orbital.

###### Host.

Unknown – possibly *Celtisoccidentalis* (Ulmaceae).

###### Distribution.

**Canada**: ON*. **USA**: DC, FL*.

###### Type material.

***Holotype*: USA. DC**: Rock Creek Park, 29.v.1922, J.R. Malloch (1♀, USNM).

###### Additional material examined.

**Canada. ON**: Ottawa, Arboretum, adult on *Celtisoccidentalis*, 27.v.2017, O. Lonsdale, CNC799468–799471 (4♀, CNC). **USA. FL**: Torreya St. Park, 29.iv.1952, O. Peck, on wood mud flat, CNC352944 (1♀, CNC).

###### Comments.

The grey pruinose notum, yellow setae and head of *Agromyzapallidiseta* make it an easily recognised, albeit uncommon, species in eastern North America, known only from the holotype, one Florida female and four Ontario females. The Ontario females were taken as adults on *Celtisoccidentalis*.

The Californian female listed as *Agromyzapallidiseta* in Spencer and Steyskal (deposited in the USNM) is clearly not conspecific with the holotype, as it is 2 × as large (4.6 mm), has a longer ocellar seta and brown setae.

##### Agromyza parca

Taxon classificationAnimaliaDipteraAgromyzidae

Spencer

Figs 198–204

Agromyza parca Spencer in Spencer and Steyskal 1986b: 265; Eiseman and Lonsdale 2018: 11; Eiseman et al. 2021: 6.

###### Description.

Wing length 2.5–2.7 mm (♂), 2.4–2.7 mm (♀). Length of ultimate section of vein M4 divided by penultimate section: 0.6–0.8. Eye height divided by gena height: 3.5–6.7. First flagellomere small and rounded; males with small tuft of apical hairs (as wide as base of arista in specimens with entirely white calypter, and several times width of base of arista in males with darker calypter hairs); females with dark calypter also with wide tuft of hairs on first flagellomere. Ocellar triangle short and equilateral in female, longer in male. Scutum shiny to subshiny.

***Chaetotaxy***: Two ori (sometimes small to well-developed additional anterior ori on one side); two ors. Two well-developed dorsocentrals and one or two much smaller setae anteriorly. Mid tibia with two posteromedial setae.

***Colouration***: Body dark brown with halter white, lunule and gena (excluding ventral margin) paler, tarsi dirty yellow and tibiae sometimes yellow at base. Wing veins light brown. Calypter margin white with hairs brown to white.

***Genitalia***: (Figs 198–204) Surstylus with weak basal suture and numerous small medial and apical tubercle-like setae on inner margin. Inner lobe of hypandrium broad with several furrows leading to empty sockets. Postgonite small and lobate. Both sclerites of basiphallus with thin mediolateral membranous lobes; apices typically incurved. Distiphallus of “*nigripes*-type” (capsule-shaped with subbasal opening for entry of ejaculatory duct, pronounced dorsobasal collar, and medial convolution); distiphallus split ventromedially with dark subbasal swelling and central ridge; with spinulose inner structures and minute anterodorsal scales; seen ventrally, sides nearly parallel or segment narrowest apically (mostly in specimens with white calypter hairs).

###### Host.

Poaceae – *Dichantheliumclandestinum*, *D.scoparium*, *Glyceriacanadensis*, *G.striata* (Eiseman et al. 2021).

###### Distribution.

**USA**: CT*, DC*, IA, MA, MD*, NC, NH*, NJ*, NY*, TN.

###### Type material.

***Holotype*: USA. NC**: Mitchell Co., Roan Mtn., 6200', 13.viii.1957, J.G. Chillcott, CNC352945 (1♂, CNC).

***Paratype*: USA. NC**: Same data as holotype, CNC352946 (1♀, CNC).

###### Additional material examined.

**USA. CT**: Putnam Park, 20.vii.1939, A.L. Melander (1♂, USNM), Redding, 3.vi.1935, A.L. Melander (2♀, USNM), **DC**: 11.vi.1926, J.M. Aldrich (1♂, USNM), **IA**: Allamakee Co., Footbridge Farm, 22.vii.2018, J. van der Linden, Poaceae, em. by 14.viii.2018, #CSE4946, CNC1643675–1643677 (2♂ 1♀, CNC), **MA**: Concord, 17.vii.1961, W.W. Wirth (2♂ 1♀, USNM), Lavale, 9.v.1970, G. Steyskal (1♂, USNM), Hampshire Co., Pelham, Arnold Rd., 2.vii.2013, C.S. Eiseman, ex *Dichantheliumclandestinum* em. 20–22.vii.2013, #CSE724, CNC392666, CNC392667 (1♂,1♀, CNC), em. 20–23.iv.2014, #CSE1089, CNC384728, CNC384729 (2♀, CNC), Plymouth Co., West Bridgewater, Maple St., 15.viii.2013, C.S. Eiseman, ex *Dichantheliumclandestinum* em. 2–5.ix.2013, #CSE866, CNC392683–392687 (2♂ 3♀, CNC), **MD**: Colesville, W.W. Wirth, 4.vi.1977 (1♂, USNM), 1.viii.1976 (1♀, USNM), Bethseda, G. Steyskal, 12.ix.1981 (1♂, USNM), 14.v.1981 (1♀, USNM), Plummers Isl., at light, 3.viii.1915, R.C. Shannon (1♂, USNM), nr. Plummers Isl., 14.v.1915, R.C. Shannon (1♀, USNM), Montgomery Co., Carderock Park, 18.v.1989, M.J. and R. Molineaux (1♀, USNM), **NC**: Chatham Co., Haywood, 5.vi.1986, G.C. Steyskal (1♂, USNM), Durham Co., Durham, 17-Acre Wood Preserve, 36°1'27.82"N, 78°55'29.73"W, 8.v.2017, T.S. Feldman, *Dichanthelium*, em. 4.v.2018, #CSE4482, CNC1135677, CNC1135678 (1♂ 1♀, CNC), Scotland Co., Laurinburg, St. Andrews University, 10.v.2017, T.S. Feldman, *Dichanthelium*, em. 6–14.v.2018, #CSE4502, CNC1144099, CNC1144100 (1♂ 1♀, CNC), Wake Co., Morrisville, Lake Crabtree County Park, 35°50'37.77"N, 78°47'43.02"W, 6.vi.2018, T.S. Feldman, *Dichantheliumscoparium*, em. 25.vi.2018, #CSE4695, CNC1135686 (1♂, CNC), **NH**: White Mts., Stinson Lake, 23.vii.1961, W.W. Wirth (1♀, USNM), **NJ**: Lakehurst, 1.vi.1962, J.E. Puleston (1♂, USNM), **NY**: Bear Mt., 30.v.1941, A.L. Melander (1♀, USNM).

###### Tentatively identified.

**USA. NC**: Scotland Co., Laurinburg, St. Andrews University, 3.v.2017, T.S. Feldman, *Dichanthelium*, em. 6.v.2018, #CSE4503, CNC1144101, CNC1144102 (2♀, CNC)

###### Comments.

*Agromyzaparca* is currently defined by an evenly dark antenna, minute dorsoapical spinules within the distiphallus and small membranous lateral lobes on the basiphallus. There is a slight amount of variation within the species, possibly indicating the presence of a cryptic taxon. Those specimens with an entirely white calypter (i.e., hairs not brown) have the pale tuft of hairs on the first flagellomere that is no wider than the base of the arista in the males and absent in the female, the eye is 3.5–5.9 × higher than the gena (not 5.2–6.7 × higher) and the distiphallus is usually slightly constricted apically (Fig. 198).

##### Agromyza parilis

Taxon classificationAnimaliaDipteraAgromyzidae

Spencer

Figs 205–209

Agromyza parilis Spencer in Spencer and Steyskal 1986b: 266.

###### Description.

Wing length 2.5–2.7 mm (♂), 3.2–3.4 (♀). Length of ultimate section of vein M4 divided by penultimate section: 0.7. Eye height divided by gena height: 2.5–2.8. First flagellomere slightly longer than high and broadly rounded, without apical tuft of pale hairs. Ocellar triangle relatively small and rounded.

***Chaetotaxy***: Two ori; two ors. Two well-developed dorsocentrals and one smaller anterior seta. Mid tibia with two posteromedial setae.

***Colouration***: Body dark brown with halter white, and gena and antenna sometimes slightly paler, with first flagellomere orange on inner-basal 1/3 and tarsi slightly paler. Calypter white with hairs dark brown; hairs sometimes yellowish in females.

***Genitalia***: (Figs 205–209) Surstylus narrow and rounded, entirely fused to epandrium and with numerous medial tubercle-like setae. Hypandrium with broadly rounded subtriangular apex; inner lobe with membranous base and inner-distal emargination. Postgonite small and lobate. Halves of basiphallus interlocking at base and with inner margins dark; outer margin weakly sclerotised, wide, wing-like and pointed; distal section distinct with long processes flanking distiphallus. Hypophallus lobate with pointed medial process. Distiphallus of “*nigripes*-type” (capsule-shaped with subbasal opening for entry of ejaculatory duct, pronounced dorsobasal collar, and medial convolution); distiphallus with dark enlarged sclerite above insertion of ejaculatory duct; distal section dark and elongate with medial suture and subapical texturing; transverse dorsobasal band produced ventrally. Ejaculatory apodeme with narrow rounded blade with numerous striations, long stem, and stout base.

###### Host.

Unknown – likely Poaceae (Spencer and Steyskal 1986b).

###### Distribution.

**USA**: TN, VA*.

###### Type material.

***Holotype*: USA. TN**: Hamilton Co., East Ridge, 9.v.1952, G.S. Walley, CNC352947 (1♂, CNC).

###### Material examined.

**USA. VA**: Fairfax Co., Turkey Run Park, nr. mouth of Turkey Run, 38°57.9'N, 7°09.4'W, Malaise trap, D.R. Smith, 25.v-6.vi.2006 (1♂ 4♀, USNM), 18–30.v.2007 (3♂, USNM), Turkey Run Park, 0.3 km W mouth Turkey Run, 38°58'N, 77°09.6'W, Malaise trap, D.R. Smith, 17–24.v.2006 (4♂ 1♀, USNM), river, 14–17.v.2006 (4♂ 5♀, USNM), Great Falls Park, quarry, 38°59.1'N, 77°14.8'W, Malaise trap, 18–24.v.2007, D.R. Smith (1♂ 3♀, USNM).

###### Comments.

While externally similar to a number of small dark eastern *Agromyza*, the first flagellomere of *A.parilis* is entirely dark on the outer face and there is no apical tuft of pale hairs in either sex. The male genitalia are most distinctive, however, and should be examined for confident identification.

##### Agromyza parvicornis

Taxon classificationAnimaliaDipteraAgromyzidae

Loew

Figs 210–214

Agromyza parvicornis Loew, 1869: 49. Frick 1952a: 373, 1957: 199 [lectotype designation]; Spencer 1969: 51, 1973: 252; Spencer and Steyskal 1986b: 67.

###### Description.

Wing length 2.5–3.2 mm (♂), 2.6–2.9 mm (♀). Length of ultimate section of vein M4 divided by penultimate section: 0.6–0.7. Eye height divided by gena height: 2.1–3.6. First flagellomere small and rounded with minute tuft of pale apical hairs. Fronto-orbital plate projecting and anterior 1/2 of frons soft and buckled.

***Chaetotaxy***: Two ori (sometimes three on one side); two ors. Two dorsocentrals, sometimes with very small third seta present anteriorly. Two posteromedial setae on mid tibia.

***Colouration***: Body mostly dark brown with orange tint, first flagellomere brown with base orange (more extensive on inner surface); frons brownish orange with ocellar triangle, posterior margin and fronto-orbital plate to level of anterior or posterior ors dark brown, sometimes dark laterally along entire margin of eye; lunule yellowish; parafacial beige and gena (excluding ventral margin) light brown; halter white; fore knee and tarsus yellowish. Calypter white with hairs light brown; uncommonly dark brown.

***Genitalia***: (Figs 210–214) Surstylus visible laterally as small lobe; inner surface with more than a dozen tubercle-like setae. Hypandrium and postgonite as described for *A.bispinata*. Halves of basiphallus converging to base where both sclerites are fused to each other and phallophorus; with internal fold past midpoint and apices converging as broad triangular lobes. Distiphallus of “*nigripes*-type” (capsule-shaped with subbasal opening for entry of ejaculatory duct, pronounced dorsobasal collar, and medial convolution); base of distiphallus narrow, distal portion broadly rounded, widest subapically and with internal spinulose processes. Ejaculatory apodeme well-developed with central rib visible near base; blade pale.

###### Hosts.

Poaceae – *Echinochloacrus-galli*, *Panicummiliaceum*, *Zeamays* and probably other cereals (Spencer and Steyskal 1986b).

###### Distribution.

**Canada**: BC, ON, QC (Zhu et al. 2004). **USA**: "Widespread, present in most states” (Frick 1959). Argentina, Cuba, Guadeloupe, Puerto Rico, Dominican Republic, Saint Vincent (Valladares 1998; Martinez and Etienne 2002).

###### Type material.

***Lectotype*: USA. DC [not given**]: “Loew coll.”, parvicornis m. (1♂, MCZ).

###### Material examined.

**Canada**. **BC**: Milner, 12.vii.1953, G.J. Spencer, CNC352949 (1♂, CNC), **ON**: Ottawa, Fletcher Wildlife Gardens, 45°23'7"N, 75°42'11.01"W, 9.vi.2013, O. Lonsdale, CNC1144190 (1♂, CNC), Ottawa, 8.vii.1925, F. Ide, CNC352950 (1♂, CNC), Point Pelee, 8.ix.1954, C.D. Miller, CNC352954, CNC352955 (2♀, CNC), Simcoe, 26.vi.1939, G.E. Shewell, CNC352952 (1♂, CNC), 29.vi.1939, CNC352953 (1♂, CNC), St. Lawrence Is. Nat. Park, Grenadier I. Centre, 26.vi.1975, H.C.W. Walther, Code 1-2406-25, CNC352951 (1♂, CNC), Picton, 10.vii.1970, J.F. McAlpine, CNC353093 (1♂, CNC), **QC**: Ile de Montreal, 17.vi.1966, Beaulieu, CNC352956 (1♀, CNC). **USA. AZ**: Sunnyside Canyon, Huachua Mts., 9.vii.1940, D.E. Hardy (1♂, USNM), **DE**: Wilmington, 31.vii.1951, D.F. Bray (1♂, USNM), Sussex Co., Dewey Beach, 21.xiii.2003, sweeping, K. Bennett (1♂, UDCC), **IN**: Lafayette 13.[?].1915, J.M. Aldrich (1♀, USNM), **MA**: Concord, vii.1960, E.H. Wheeler, reared ex. *Zeamays* (2♂ 3♀, USNM), Franklin Co., Northfield, 276 Old Wendell Rd., 42°38'48.74"N, 72°25'32.15"W, 31.viii.2017, C.S. Eiseman, *Zeamays*, em. 2–23.vi.2018, #CSE4580, CNC1135703–1135712 (5♂ 5♀, CNC), **MD**: Montgomery Co., Colesville, Malaise trap, W.W. Wirth, 7.viii.1975 (1♀, USNM), 14.vi.1973 (1♂, USNM), 20.viii.1975 (1♂, USNM), Bethseda, 6.viii.1967, G. Steyskal (1♂, USNM), Colesville, 24.vii.1974, W.W. Wirth (1♂, USNM), 4mi SW of Ashton, Malaise trap, G.F. and J.F. Hevel, 3.ix.1981 (1♂, USNM), 1.ix.1981 (2♂, USNM), **NY**: L.I. Veg. Res. Fm, Riverhead, at light, 1–7.viii.1938, 30.viii.1938, 7–20.viii.1938, CNC352957, CNC352958, CNC352959, CNC352960, CNC352961, CNC352962, CNC352963, CNC352964, CNC352965, CNC352966, CNC352967, CNC352968 (11♂ 1♀, CNC), **TN**: Nashville, G.G. Ainslie, Webster No. 9467C (1♂ [with puparium], USNM), **VA**: Chain Bridge, 14.v.1924, J.R. Malloch (1♀, USNM).

###### Comments.

*Agromyzaparvicornis* and *A.proxima* are highly similar in appearance and develop in some of the same plant genera, and can only be reliably differentiated on the basis of the relative width of the distiphallus and length of the basiphallus. It is possible that *A.proxima* represents a less common eastern morphological variant of the other, but in the absence of corroborating evidence, these two are maintained here as separate.

##### Agromyza proxima

Taxon classificationAnimaliaDipteraAgromyzidae

Spencer

Fig. 215

Agromyza proxima Spencer, 1969: 52. Spencer and Steyskal 1986b: 67.

###### Description.

Wing length 2.2–2.4 mm (♂), 2.2–2.5 (♀). Length of ultimate section of vein M4 divided by penultimate section: 0.6–0.7. Eye height divided by gena height: 2.8–3.4. First flagellomere small and rounded, sometimes with very small tuft of pale hairs apically. Scutum shiny.

***Chaetotaxy***: Two ori (sometimes three on one side); two ors. Two well-developed dorsocentrals with smaller third seta anteriorly. Hind tibia with two posteromedial setae.

***Colouration***: As described for *A.parvicornis*, except fronto-orbital plate usually brownish orange around base of all fronto-orbital setae.

***Genitalia***: (Fig. 215) Genitalia as described for *A.parvicornis* except as follows: basiphallus short, only slightly longer than distiphallus; distal section of distiphallus narrow, with sides only slightly bulging subapically.

***Variation***: MD male from Anne Arundel Co. with distal 2/5 of first flagellomere covered with pale hairs; genitalia as described above, but surstylus relatively small and spherical; distal 2/3 of first flagellomere dark and pedicel orange distally; frons dark brown. VA female with relatively large ovate distoventral patch of pale hairs on first flagellomere; body entirely dark with pedicel slightly paler, calypter entirely white, notopleuron and postpronotum reddish, and wing veins whitish on basal 1/2. The female is only tentatively allied with the MD male on the basis of similar dark colouration and the increased size of the pale haired patch on the first flagellomere.

###### Hosts.

Poaceae – *Dichanthelium* sp, *Echinochloawalteri*, *Panicumdichotomiflorum* (Scheffer and Lonsdale 2018, Spencer and Steyskal 1986b).

###### Distribution.

**Canada**: MB. **USA**: DE*, FL, MA*, MD*, NY, VA(?).

###### Type material.

***Holotype*: USA. FL**: Sweetwater, Tamiami Canal (1♂, USNM).

***Paratypes*: Canada. MB**: 2 mi N Forrest, Populusbalsamifera stand around slough, 19.vii.1958, J.G. Chillcott, CNC352969 (1♂ [head missing], CNC). **USA. FL**: Sweetwater, 3.viii.1963, ex. *Echinochloawalteri*, leg. 23.vii.1963, K.A. Spencer (1♂ 1♀ [same pin, with puparia], USNM).

###### Additional material examined.

**USA. DE**: Newark, 30.vii.1974 (1♂, UDCC), Stanton, 8.viii.1951, D.F. Bray (1♂, USNM), **MA**: Greenfield, 6.[?].1914, A.L. Melander (1♂, USNM), Wayland, 28.[?], J.J. Pratt, LT (1♂, USNM), **MD**: Bethseda, 12.ix.1981, G.C. Steyskal (1♀, USNM), Colesville, 15.viii.1975, Malaise trap, W.W. Wirth (1♂, USNM), Anne Arundel Co., nr. Edgewater, Smithsonian Envir. Res. Centre, 38°53'N, 76°33'W, 10.vii.1993, G.F. Hevel (1♂, USNM), **VA**: Sherando Lake, 10 mi SW Waynesboro, 25.vi.1970, L.V. Knutson (1♀, USNM).

##### Agromyza pudica

Taxon classificationAnimaliaDipteraAgromyzidae

Spencer

Figs 216–220

Agromyza pudica Spencer in Spencer and Steyskal 1986b: 267; Eiseman and Lonsdale 2018: 12, 2019: 3.

###### Description.

Wing length 2.4–2.6 mm (♂), 2.0–2.6 mm (♀). Length of ultimate section of vein M4 divided by penultimate section: 0.7–0.9. Eye height divided by gena height: 4.8–4.9. First flagellomere small and ovate with small tuft of apical hairs in male. Ocellar triangle small and rounded (smaller in holotype). Scutum shiny.

***Chaetotaxy***: Two ori (sometimes three on one side); two ors. Three dorsocentral setae with anterior seta reduced. Mid tibia with two posteromedial setae.

***Colouration***: Body dark brown with orange tint; first flagellomere dark brown with faint orange tint towards base sometimes evident; lunule paler; parafacial sometimes with orange tint; halter white; tarsi and base of fore tibia yellow. Wing veins light brown. Calypter white with hairs usually brown (white in some females).

***Genitalia***: (Figs 216–220) Genitalia largely as described for *A.parvicornis*, except as follows: basiphallus long and well-sclerotised, halves overlapping at base, weakened point present past midpoint and apex split with dark ventral branch; distiphallus narrow, with broad medial constriction and with elongate ventral sulcus below distomedial ridge; ejaculatory apodeme small with blade pale.

###### Host.

Poaceae – *Dichanthelium* spp. (Eiseman and Lonsdale 2018, 2019).

###### Distribution.

**Canada**: ON*. **USA**: AR, CT*, DC*, GA, MA, MD*, MN, NC, OH, OK, NY*, SC*, VA*.

###### Type material.

***Holotype*: USA. AR**: Garland Co., Hot Springs, 15.v.1979 (1♂, USNM).

###### Paratypes examined.

**USA. GA**: Rabun Co., Rabun Bald, 1280 m, 16.vii.1957, J.G. Chillcott, CNC352972 (1♀, CNC), Rabun Bald, 914 m, 14.vii.1957, J.G. Chillcott, CNC352971 (1♂, CNC), **NC**: Pisgah Forest, Looking Glass Pk., 19.vii.1957, “K.A. Spencer, col.”, W.R. Richards, CNC352970 (1♂, CNC).

###### Additional material examined.

**Canada. ON**: Algonquin Pk., 45°50'0"N, 77°38'0"W, 19.vii.1991, J.R. Vockeroth, CNC353094 (1♂, CNC), Metcalfe, 2mi N, 15.v.1982, B.E. Cooper, CNC353095 (1♂, CNC), 10.vi.1982, CNC353097 (1♂, CNC), 25.v.1982, CNC353096 (1♂, CNC), Ottawa, damp second-growth forest in Acer-Betula wood, 27.vi.1989, J.R. Vockeroth, CNC353098 (1♂, CNC). **USA. CT**: Waterton, 5.vi.1931, A.L. Melander (1♀, USNM), Putnam Park, A.L. Melander, 18.vii.1939 (1♂, USNM), 20.vii.1939 (1♂, USNM), 24.vii.1939 (1♀, USNM), Redding, A.L. Melander, 26.vi.1929 (1♂, USNM), 1.vi.1929 (1♀, USNM), 10.vi.1929 (1♀, USNM), 8.vi.1930 (1♂ 2♀, USNM), 11.vi.1929 (1♀, USNM), 27.vii.1938 (1♀, USNM), **DC**: 11.vi.1926, J.M. Aldrich (2♂, USNM), Chain Bridge, 8.v.1928, J.M. Aldrich (1♂, USNM), Washington, 17.viii.1913, A.L. Melander (2♂, USNM), **MA**: Concord, 17.vi.1961, W.W. Wirth (1♂, USNM), Franklin Co., Northfield, 276 Old Wendell Rd., 4.vii.2016, C.S. Eiseman, Dichantheliumacuminatumssp.fasciculatum, em. 21.vii.2016, #CSE2787, CNC654200–654202 (1♂ 2♀, CNC), **MD**: Bethseda, 30.v.1980, G.C. Steyskal (1♀, USNM), Plummers Isl., 3.vi.1914, R.C. Shannon (1♂, USNM), Montgomery Co., 4mi S of Ashton, 24.vii.1982, G.F. and J.F. Hevel (2♀, USNM), **NC**: Scotland Co., Laurinburg, St. Andrews University, 18.v.2016, T.S. Feldman, *Dichanthelium* em. 13.vi.2016 , #CSE2572, CNC634813–634815 (3♂, CNC), Durham Co., Durham, 17-acre Wood Preserve, 15.v.2016, T.S. Feldman, *Dichantheliumclandestinum*, em. 4.vi.2016, #CSE2542, CNC654303–654306 (4♂, CNC), Scotland Co., Laurinburg, St. Andrews University, 11.v.2016, T.S. Feldman, *Dichantheliumscoparium*, em. 3.vi.2016, #CSE2540, CNC653948, CNC653949 (1♂ 1♀, CNC), **NY**: Lk. George, 26.vii.1929, A.L. Melander (1♀, USNM), Yonkers, 3.vi.1927, A.L. Melander (1♀, USNM), 5.vi.1927 (1♀, USNM), Peekskill, 11.vi.1927, A.L. Melander (1♂, USNM), Bear Mt., 31.v.1941, A.L. Melander (1♂, USNM), **OH**: Hocking Co., South Bloomingville, Deep Woods Farm, 5.viii.2016, C.S. Eiseman, *Dichantheliumclandestinum*, em. 20–22.viii.2016, #CSE2925, CNC654480–654482 (1♂ 2♀, CNC), **SC**: Beaufort Co., Fripp Island, 26.ix.1973, G.C. Steyskal (1♂, USNM), Highland Fall, 6.vii.1941, A.L. Melander (1♀, USNM), Cobleskill, 11.viii.1970, L. Knutson (1♀, USNM), **VA**: Glencarlyn, 23.v.1925, J.R. Malloch (1♂, USNM), Falls Church, Holmes Run, 24.viii.1960, light trap, W.W. Wirth (1♀, USNM), Rosslyn, 25.vi.1913, R.C. Shannon (1♀, USNM), Fairfax Co., Great Falls Park, swamp trail, 38°59.4'N, 77°15.2'W, Malaise trap, trap #1, 3–7.v.2007, D.R. Smith (1♂, USNM).

###### Comments.

*Agromyzapudica* is recorded here in Canada, D.C., and five states for the first time.

##### Agromyza soka

Taxon classificationAnimaliaDipteraAgromyzidae

Eiseman & Lonsdale

Figs 221–224

Agromyza soka Eiseman & Lonsdale, 2018: 13.

###### Description

**(from Eiseman and Lonsdale 2018).** Wing length 2.3–2.5 mm (♂), 2.3–3.0 mm (♀). Length of ultimate section of vein M4 divided by penultimate section: 0.6–0.7. Eye height divided by gena height: 6.9–9.2. Ocellar triangle relatively small and rounded. First flagellomere small and nearly circular or slightly longer than high, with nearly indistinct apical tuft of pale hairs. Notum pruinose.

***Chaetotaxy***: Two or three ori (anterior seta small if present); two ors. Ocellar and postvertical setae subequal to outer vertical seta. Five dorsocentrals, strongly decreasing in length anteriorly. Eight irregular rows of acrostichal setulae. Mid tibia with one (male paratype) or two posteromedial setae.

***Colouration***: Setae dark brown with light brown reflection. Body predominantly dark brown (female darker) with light pruinosity; antenna dirty orange with distal 1/2 of first flagellomere infuscated (more so dorsally) in holotype, entirely brown in paratypes, with antenna entirely dark brown in female; frontal vitta, gena and postgena paler; apices of fore or all femora narrowly yellow; tarsi yellow; fore tibia light brown, fading to yellow at base. Calypter white with hairs brown. Halter white.

***Genitalia***: (Figs 221–224) Surstylus not distinct from epandrium, barely visible laterally, flat on inner surface and with four large spines. Cerci narrow and convergent. Hypandrium broad with thick arch, small apical process, and large inner lobe with two distal setae and several minute basal pits. Postgonite lobate and downturned. Proepiphallus and metepiphallus strongly reduced, flattened. Phallophorus elongate on left side. Halves of basiphallus strongly diverging from, and partially fused to phallophorus; lateromedially with lightly sclerotised membranous lobe; apex folded inwards, with pointed basal process and elongate distal process that is fused to mesophallus. Hypophallus broad, flat, and heavily sclerotised; apically split in dissected NC male. Mesophallus cylindrical, dark, basally rounded, slightly longer than wide, fused to distiphallus. Distiphallus broad, black, ventrally tilted and cup-like with constricted opening enclosing haired inner process. Ejaculatory apodeme well-developed with blade paler, no medial rib evident.

###### Host.

Fabaceae – *Robiniapseudoacacia*, *Wisteriafloribunda*.

###### Distribution.

**USA**: CT, NC, VA.

###### Type material.

***Holotype*: USA. VA**: nr. Plummers Isl., 20.v.1914, R.C. Shannon (1♂, USNM).

***Paratypes*: USA. CT**: Hartford Co., East Hartford, Two Rivers Magnet Middle School, 4.vi.2016, em. 27–29.iv.2017, C.S. Eiseman, ex *Robiniapseudoacacia*, #CSE3574, CNC939943–939946 (3♂ 1♀, CNC), **NC**: Scotland Co., Laurinburg, St. Andrews University, 24.iv.2015, em. 16–18.iii.2016, T.S. Feldman, ex *Robiniapseudoacacia*, #CSE2248, CNC653954, CNC653955 (1♂ 1♀, CNC), 10.iv.2017, *Robiniapseudoacacia*, em. 22.iv.2018, #CSE4423, CNC1135665 (1♂, CNC), 4.iv.2016, em. 18.iv– 3.v.2017, T.S. Feldman, ex *Wisteriafloribunda*, #CSE3518, CNC939744–939747 (1♂ 3♀, CNC).

##### Agromyza tacita

Taxon classificationAnimaliaDipteraAgromyzidae

Spencer

Figs 225–231

Agromyza tacita Spencer, 1969: 59. Spencer and Steyskal 1986b: 66.

###### Description

**(Fig. 225).** Externally as described for *A.bispinata* except as follows: Wing length 2.3–3.3 mm (♂). Length of ultimate section of vein M4 divided by penultimate section: 0.5–0.8. Eye height divided by gena height: 2.7–4.8. Small additional ori present in male with five spines on surstylus. First flagellomere sometimes paler with orange tint or predominantly orange with distal 1/2 infuscated; always enlarged in male. Postpronotum sometimes light brown or with orange tint. Wing veins brown to yellow. Fore tibia of MD male yellow with centre brownish.

***Genitalia***: (Figs 226–231) Surstylus fused to epandrium, easily viewed laterally, with dark anterior lobe and dark pronounced protuberances at base of marginal spines (three to five in number). Hypandrium, postgonite and ejaculatory apodeme as described for *A.bispinata*. Left sclerite of basiphallus overlapping base of right sclerite, which is fused to phallophorus; both halves with lateromedial membranous projection, and with weak connection to incurved distal section. Distiphallus of “*nigripes*-type” (capsule-shaped with subbasal opening for entry of ejaculatory duct, pronounced dorsobasal collar, and medial convolution); ventral curve with truncated sclerotised elbow; distal section with bulging, internally spinulose medial section.

***Variation***: Dissected paratype with two spines on surstylus (as in *A.bispinata*), but surstylus and remaining genitalia otherwise identical to holotype.

***Variation***: *female paratypes*: Wing length 2.9–3.1 mm. Length of ultimate section of vein M4 divided by penultimate section: 0.4. Eye height divided by gena height: 8.7–11.8. First flagellomere smaller, ovate; base always orange, reminder orange to brown. Anterior 1/2 of frons sometimes orangish.

###### Host.

Unknown – likely Poaceae (Spencer and Steyskal 1986b).

###### Distribution.

**Canada**: AB*, MB[?], NB*, ON, QC. **USA**: MD*, MO, NH*, NY*, UT*, VA*.

###### Type material.

***Holotype*: Canada. ON**: Ottawa, 17.vi.1946, G.E. Shewell, CNC352973 (1♂, CNC).

***Paratypes*: Canada**. **MB**: Int. Peace Gardens, Turtle Mtn. For. Res., 7.viii.1958, J.G. Chillcott, CNC Type No. 10355, CNC352975 (1♀, CNC), **ON**: Ottawa, 15.vii.1957, J.E.H. Martin, CNC Type No. 10355, CNC352977 (1♀, CNC), 20.vi.1957, J.G. Chillcott, CNC Type No. 10355, CNC352974 (1♂, CNC), **QC**: Hull, 5.vi.1959, J.R. Vockeroth, CNC Type No. 10355, CNC352976 (1♀, CNC), Old Chelsea, summit of King Mt., 350 m, 11.vi.1959, J.R. Vockeroth, CNC Type No. 10355, CNC352978 (1♀, CNC).

###### Additional material examined.

**Canada. AB**: Elkwater L., 21.vii.1956, O. Peck, CNC353101 (1♂, CNC), **NB**: Kouchibouguac N.P., 11.vii.1977, J.F. McAlpine, Code-6035C, CNC352990–352992 (3♂, CNC), 12.vii.1977, J.F. McAlpine, Code-6040H, CNC352989 (1♂, CNC), 13.vii.1977, G.A. Calderwood, Code-5599I, CNC352993 (1♂, CNC), 30.vi.1977, J.R. Vockeroth, Code-5456V, CNC352986, CNC352987, CNC352981–352985 (7♂, CNC), 6.vii.1977, G.A. Calderwood, Code-54900, CNC352988 (1♂, CNC), 9.vii.1977, J.F. McAlpine, Code 6024R, Code-6024R, CNC352979, CNC352980 (2♂, CNC), **ON**: Ottawa, on leaf of *Philadelphuscoronarius*, 13.vii.1959, J.R. Vockeroth, CNC353100 (1♂, CNC). **USA. MD**: Plummers Isl., 14.vi.1913, R.C. Shannon (1♂, USNM), **NH**: Jefferson Notch, 30.vii.1961, creek margin, W.W. Wirth (1♂, USNM), **NY**: Bear Mt., 31.v.1941, A.L. Melander (1♂, USNM), **UT**: Cache Co., Green Canyon, 23.vii.1964, Malaise trap, W.J. Hanson (1♂, USNM), **VA**: Shenandoah, Big Meadows, 3.vii.1939, A.L. Melander (1♂, USNM), Fairfax Co., Dead Run, 9.vi.1915, R.C. Shannon (1♂, USNM).

###### Comments.

See comments for *Agromyzabispinata*. The female paratypes of *A.tacita* cannot be confidently assigned to this species, which is mostly defined by male genitalic characters, and their placement should be re-evaluated when the boundaries of species in the *A.bispinata* complex can be better delimited.

##### Agromyza varifrons

Taxon classificationAnimaliaDipteraAgromyzidae

Coquillett

Figs 236–242

Agromyza varifrons Coquillett, 1902: 189. Frick 1952a: 374; Spencer 1969: 59; Spencer and Steyskal 1986b: 269; Scheffer and Lonsdale 2018: 85.

###### Description.

Wing length 1.7–2.2 mm (♂), 1.8–2.3 mm (♀). Length of ultimate section of vein M4 divided by penultimate section: 0.9–1.0. Eye height divided by gena height: 3.5–10.0. Arista pubescent. First flagellomere small and rounded, without pale tuft of hairs. Fronto-orbital plate slightly projecting (more so anteriorly). Ocellar triangle small and rounded.

***Chaetotaxy***: Two ori; two ors. Orbital setulae in one row. Ocellar and postvertical setae subequal to fronto-orbitals. Two strong dorsocentral setae, with second seta ~ ¾ length of first seta; much weaker third dorsocentral not much larger than acrostichal seta. Acrostichal seta ~ 2 × length of setulae. Acrostichal setulae in 8 scattered rows, tapering posteriorly to level of first dorsocentral. Mid tibiae without posteromedial setae.

***Colouration***: Setae dark brown. Antenna yellow to orange; first flagellomere darker orange with basal margin paler and with small brown curved spot around base of arista posteriorly and along outer surface. Frons yellow with posterolateral corner and posterior margin dark brown; orbital plate darker, brownish orange behind anterior ors, becoming darker posteriorly, and with narrow brownish line continuing anteriorly along eye margin beside ori; ocellar spot rounded, dark brown, slightly larger than tubercle; remaining posterior 1/2 or less of frontal vitta brownish orange, becoming darker posteriorly, sometimes with this dark region restricted to large circle around ocellar spot. Lunule light yellow. Gena yellow with ventral brownish line. Parafacial yellow. Face whitish yellow. Mouthparts yellow; clypeus with brown tint that is darker laterally. Postgena, back of head and remainder of body mostly dark brown; body subshiny. Apex of fore femur yellowish orange for length equal to width of femur apex; apex of mid femur narrowly paler brown apically; tibiae sometimes paler brown with base and apex yellowish, and mid tibia sometimes with yellow mottling medially; tarsi yellow, becoming more brown tinted on posterior legs; wing veins brown; halter yellow with orange-brown tint on stem; calypter margin and hairs brown.

***Variation***: Posterior 1/2 of frons brownish in holotype and FL specimen. IL male with first flagellomere entirely light yellow, head brighter and scutum shiny.

***Genitalia***: (Figs 236–242) Epandrium as long as high; fused to small, inwardly directed surstylus. Cercus large, well-developed, venter of inner surface covered with tubercle-like setae. Hypandrium with large apical apodeme and narrow basal arms; medially with weakly sclerotised region connected to L-shaped inner lobe bearing row of minute setae. Postgonite in lateral view with short, flat globose body with extremely long tail; inner surface with apically setose shelf. Phallophorus elongate cylindrical, apicodorsal margin irregularly sclerotised to base of basiphallus. Basiphallus with small left basolateral plate partially fused to large dorsal plate that is strongly curved ventrally at base and with medial and apical lobes that wrap along right lateral surface of shaft. Ejaculatory duct widening to mesophallus, which is round, bulbous, and clear with thick triangular sclerotisation ventrally. Distiphallus composed of one pair of upturned “wings” that nearly meet ventrally and are very narrowly connected dorsally; each wing with small, dark, smooth base expanding into densely haired, distally pointed lobes. Ejaculatory apodeme weakly sclerotised, with broad slanted base and short stem grading into thick blade with faint apical striations; sperm pump with slight basal sclerotisation.

###### Hosts.

Cannabaceae – *Celtislaevigata*. Possibly *Ulmus Americana* (Ulmaceae) (Eiseman 2021).

###### Distribution.

**Canada**: ON, QC. **USA**: AK, DC, FL, IA, IL, KS, MS, NY, PA, TX, VA*.

###### Type material.

***Holotype*: USA. DC**: “District of Columbia” (1♀, USNM).

###### Additional material examined.

**USA. FL**: Hialeah, em. 10.v.1963, mine *Celtislaevigata*, 22.ix.1963 (1♂ 1♀ [same pin, with puparia]), Gainesville, Newman’s Lake, 26.iv.1952, O. Peck, CNC352994 (1♂, CNC), Hialeah, 22.ix.1963, mine *Celtislaevigata*, Em. 5.x.1963, [K.A. Spencer], CNC352996 (1♂ 1♀ [with puparia], CNC), **IL**: [illegible], 15.v.1919 (1♀, USNM), **NY**: Suffolk Co., Stony Brook Village, larva coll. 19.vi.1993, S.J. Scheffer, 93-187 (1♂[illustrated], USNM) , eclosed 9.vii.1993 (1♂, USNM), eclosed 8.vii.1993 (1♀, USNM), eclosed 10.vii.1993 (3♂, USNM), eclosed 11.vii.1993 (1♀, USNM), elcosed 17.vii.1993 (1♂, USNM), **TX**: Kerrville, 4.iv.1959, J.F. McAlpine, CNC352995 (1♂, CNC), Welder Wildlife Ref. nr Sinton, 19–23.iii.1965, J.G. Chillcott, CNC352997 (1♀, CNC), **VA**: Fairfax Co., Turkey Run Park, nr. mouth of Turkey Run, 38°57.9'N, 7°09.4'W, Malaise trap, 26.iv-2.v.2007, D.R. Smith (3♀, USNM), Turkey Run Park, 0.3 km W mouth Turkey Run, 38°58'N, 77°09.6'W, Malaise trap, D.R. Smith, 18–30.v.2007 (1♀, USNM), 29.iii-25.iv.2007 (1♂, USNM), Great Falls Park, quarry, 38°59.1'N, 77°14.8'W, Malaise trap, D.R. Smith, 24.iv-2.v.2007 (3♂ 1♀, USNM), 10–17.v.2007 (1♂, USNM), 3–10.v.2007 (1♂, USNM).

###### Comments.

This species is clearly allied to *Agromyzaaristata*, being similar in external and male genitalic morphology. The anterior portion of the head is mostly pale, the cercus is covered with small tubercle-like setae along the anterior surface, and the mesophallus is a ventral band separate from a curved, plate-like distiphallus. The most immediate difference in the genitalia is the densely haired distiphallus and short mesophallus, but the hypophallus has an apical apodeme, the basiphallus is also strongly curved and irregular in outline, the postgonite broadly lobate (lateral view) and the ejaculatory apodeme is well-developed. Externally, the pale portions of *A.aristata* are light yellow, not orange to yellowish, and the legs are extensively yellow.

##### Agromyza virginiensis

Taxon classificationAnimaliaDipteraAgromyzidae

Spencer

Fig. 151

Agromyza virginiensis Spencer, 1977: 237. Spencer and Steyskal 1986b: 64.

###### Description.

Wing length 3.2 mm. Length of ultimate section of vein M4 divided by penultimate section: 0.6. Eye height divided by gena height: 3.4. First flagellomere longer than wide with rounded apex; apical margin possibly with longer, paler hairs. Fronto-orbital plate and parafacial slightly projecting. Ocellar triangle broadly rounded and short, not extending much past ocelli. Buccal cavity subquadrate.

***Chaetotaxy***: Four ori; one ors (not two as reported by previous authors). Four postsutural dorsocentrals, decreasing in length anteriorly. Mid tibia with two posteromedial setae.

***Colouration***: Body predominantly brown with halter white. Gena (excluding ventral margin), parafacial and frons (excluding posterolateral corner, posterior margin and ocellar triangle) light brownish orange, with fronto-orbital plate lightly pigmented posteriorly to base of posterior ori. First flagellomere, scape and pedicel orange, sometimes with dark spot at base of arista. Lunule orange. Face brown. Palpus and clypeus dark brown. Calypter margin and hairs white. Legs dark brown with knees yellow and tibiae and tarsi with orange tint (brighter on tarsi).

***Genitalia***: (Fig. 151) Surstylus triangular with basal suture obliterated; with minute apical setae and medial tubercle-like setae. Hypandrium relatively long and narrow with minute apical point. Postgonite very broad, flat, and lobate. Halves of basiphallus long with ventral margin weakly defined, apices hooked, and right sclerite with broader base. Distiphallus very long, cylindrical, dark and with distal 2/5 bifid and not strongly curved. Ejaculatory apodeme small and thin, without apical blade.

***Variation***: Canadian material differs as follows: wing length 3.0–3.2 mm (♂), 3.4–3.8 mm (♀); eye height divided by gena height 5.1–10.6; first flagellomere with relatively discrete ovate pilose patch; 2 ors and 2–3 ori; first flagellomere brownish on distal and dorsal margins anterior to arista base; posterior 1/3 of frons much darker; fronto-orbital plate with ill-defined brown margin along length; parafacial dark brown; distiphallus only split on distal 1/10.

###### Host.

Unknown – adult collected on *Phyllocarpus* (Fabaceae).

###### Distribution.

**Canada**: ON*. **USA**: VA.

###### Type material.

***Holotype*: USA. VA**: Great Falls, vi.1922, Banks (1♂, MCZ).

###### Additional material examined.

**Canada. ON**: Orleans, Chapel Hill, at flowers of *Phyllocarpus*, 23.vi.1994, J.R. Vockeroth, CNC353052 (1♀, CNC), Ottawa, Damp second-growth *Acer*-*Betula* woods, 12.vii.1994, J.R. Vockeroth, CNC353053 (1♀, CNC), 15.vi.1998, CNC353059 (1♀, CNC), 16.viii.1992, CNC353046 (1♂, CNC), 17.vi.2003, CNC353056 (1♀, CNC), 2.vii.1993, CNC353044 (1♂, CNC), 24.vii.2000, CNC353049 (1♂, CNC), 29.vi.1994, CNC353058 (1♀, CNC), 29.vi.1997, CNC353047 (1♂, CNC), 29.vii.1992, CNC353045 (1♂, CNC), 30.vi.1991, CNC353054 (1♀, CNC), 30.vii.1993, CNC353051 (1♀, CNC), 6.viii.1993, CNC353043, CNC353057 (1♂,1♀, CNC), 7.viii.1993, CNC353050 (1♂, CNC), 7.viii.2000, CNC353055 (1♀, CNC), 9.vii.2000, CNC353048 (1♂, CNC).

###### Comments.

The Canadian material varies slightly from the holotype, being more similar in external appearance to *Agromyzaambrosivora*, although the straight (not strongly curled) distiphallus would seem to preclude this option, and the anterior margin of the buccal cavity is relatively straight (more pointed in *A.ambrosivora*). The phallus of these Canadian specimens further differs from the holotype in that only the apex of the distiphallus is cleft, but these external and phallic differences will be tentatively treated as within-species variation until additional specimens can be measured.

The phallus is similar to that of a Nearctic and European leaf miner on *Urtica* (Urticaceae), *Agromyzareptans* (Spencer and Steyskal 1986b: figs 395, 396). The distiphallus of both is straight and apically split, but that of *A.reptans* is much shorter, paler, and with the apical split represented by one pair of separate, darker cylindrical arms (not the ends of a single, continuous, narrow tube), barely longer than wide and stouter than the base.

##### Agromyza vockerothi

Taxon classificationAnimaliaDipteraAgromyzidae

Spencer

Figs 243–249

Agromyza vockerothi Spencer, 1969: 60. Spencer and Steyskal 1986b: 269; Eiseman and Lonsdale 2018: 14.

###### Description.

Externally as described for *A.isolata* except as follows: Wing length 2.4–2.7 mm (♂), 2.4 mm (♀). Length of ultimate section of vein M4 divided by penultimate section: 0.6–0.8. Eye height divided by gena height: 3.8–6.5. Sometimes three ori on one side of frons. Overall colour slightly darker, with antenna dark brown and calypter margin and hairs always brown; mid and hind tibiae brown with apex and sometimes base yellowish. First flagellomere sometimes appearing slightly angulate and with some longer apical hairs.

***Genitalia***: (Figs 243–249) Surstylus not distinct from epandrium (which has desclerotised distal emargination), and with several setae and tubercle-like setae on inner margin. Hypandrium long and narrow, sides nearly parallel and with tapered apical process. Postgonite small, dome-like with several outer setulae. Phallophorus atrophied on right side, produced and slightly setulose on left side, fused to right sclerite of basiphallus, twisting around shaft; left sclerite of basiphallus narrow, curving around venter at base. Basiphallus broad and fringed basally with dextral twist. Hypophallus composed of weak, long narrow band. Mesophallus short, cylindrical, with longer ventral plate. Distiphallus short, subcylindrical with ventral break, and fringed along distal and ventral margins.

###### Host.

Rosaceae – *Rubus*.

###### Distribution.

**Canada**: AB, BC, ON, NS. **USA**: CT*, DC*, MA, MD*, NC, NH*, NY*, PA*, TN, VA*.

###### Type material.

***Holotype*: Canada. NS**: Shelburne, 10.viii.1958, J.R. Vockeroth, Type No. 10356, CNC352998 (1♂, CNC).

###### Paratypes examined.

**ON**: Maynooth, 22.vi.1953, J.F. McAlpine, CNC353000 (1♂, CNC), Ottawa, 5.vi.1946, G.E. Shewell, CNC353001, CNC353002 (2♂, CNC), Golden Lake, 3.ix.1956, J.R. Vockeroth, CNC353003 (1♀, CNC), **QC**: Old Chelsea, 18.viii.1961, J.R. Vockeroth, CNC352999 (1♂, CNC).

###### Material examined.

**Canada. BC**: 20 km E Pemberton, 14.viii.1991, A. Borkent, CNC353014, CNC353015 (2♂, CNC), Hagensborg, 12.vii.1992, A. Borkent, CNC353016 (1♂, CNC), 42 km NE Hope, 2.viii.1991, A. Borkent, CNC353015 (1♂, CNC), 32 mi SW of Terrace, 9.vii.1960, J.G. Chillcott, CNC353017 (1♂, CNC), **ON**: St. Lawrence Is. Nat. Park, Aubrey Island, 4.ix.1976, W. Reid, Code 4618-R, CNC353006 (1♂, CNC), St. Lawrence Is. Nat. Park, Cedar Island, 31.viii.1976, W. Reid, Code 4586-L, CNC353007 (1♀, CNC), St. Lawrence Is. Nat. Park, McDonald Is., 14.vi.1976, 6.ix.1976, A. Carter, W. Reid, Code 4092-J, Code 4633-G, CNC353005, CNC353008 (1♂ 1♀, CNC), St. Lawrence Is. Nat. Park, Thwartway Is., 24.vii.1976, W. Reid, Code 4198-L, CNC353009 (1♀, CNC). **USA. CT**: Kent Fall, 28.viii.1940, A.L. Melander (1♂, USNM), Redding, 28.v.1939, A.L. Melander (1♂, USNM), **DC**: Washington, “v-17”, J.M. Aldrich (1?, USNM), **MA**: Franklin Co., Northfield, Crag Mountain, 11.x.2013, ex. *Rubus* (blackberry), em. 27.iii.2014, C.S. Eiseman, #CSE1030, CNC384794 (1♂, CNC), **MD**: Glen Echo, 21.viii.1921, J.R. Malloch (1♂, USNM), Colesville, 1.v.1977, W.W. Wirth (1♀, USNM), Montgomery Co., Dickerson, 14.vii.1974, G.A. Foster (1♀, USNM), **NC**: Highlands, Wilson’s Gap, 944 m, 12.v.1957, J.R. Vockeroth, CNC353011 (1♀, CNC), Mitchell Co., Roan Mtn, 1889 m, 13.viii.1957, J.G. Chillcott, CNC353010 (1♀, CNC), Wyah Bald, 13.vi.1957, C.J. Durden, CNC353012 (1♀, CNC), **NH**: White Mts., Stinson Lake, 23.vii.1961, W.W. Wirth (1♂, USNM), **NY**: Allegany St. Pk., 28.v-3.vi.1963, stream margin, W.W. Wirth (1♂, USNM), Orleans Co., Albion, 11.vi.1963, Burma woods, W.W. Wirth (1♂, USNM), Monroe Co., Braddock Bay, 12.vi.1963, near marsh, W.W. Wirth (1♂, USNM), **PA**: Mineral Spr., 5.ix.1927, A.L. Melander (2♂, USNM), Monroe Co., Mt. Pocono, 28.vii.1985, G.F. and J.F. Hevel (1♂, USNM), **TN**: Smokies, Chimneys, A.L. Melander, 25.vi.1941 (1♀, USNM), 20.vi.1941 (1♂, USNM), Indian Gap to Clingman’s Dome, 5200–6600', 6.viii.1957, J.G. Chillcott, CNC353004 (1♂, CNC), **VA**: Luray, 24.vi.1923, A.L. Melander (1♂, USNM), Fairfax Co., Turkey Run Park, nr. mouth of Turkey Run, 38°57.9'N, 7°09.4'W, Malaise trap, river trap, 17–24.v.2006, D.R. Smith (1♀, USNM).

##### Euhexomyza

Taxon classificationAnimaliaDipteraAgromyzidae

Lonsdale

Euhexomyza Lonsdale, 2014: 497. Type species MelanagromyzasimplicoidesHendel 1920, by original designation.

*Euhexomyza* was described by Lonsdale (2014) for a lineage of mostly north-temperate Salicaceae-feeding species where the larva forms an ovate gall in the cortex of a twig. These species were previously treated as part of *Hexomyza* Enderlein, but Lonsdale (2014) found the genus to be a polyphyletic dumping ground for a miscellany of gall-forming species, with the morphologically disparate type species belonging to *Ophiomyia*. He subsequently included the name *Hexomyza* as a junior synonym of *Ophiomyia*.

Species are brown and non-metallic (Fig. 44), they have a rounded clypeus and lack a prescutellar acrostichal. The fronto-orbital plate and parafacial are pronounced, forming a strong ring around the eye, the postsutural intra-alars are subequal (anterior seta never strong), and only the mid tibia sometimes has a lateromedial seta. The male genitalia (at least of the north temperate Salicaceae-feeders) are typified by a distiphallus that has the base broad and rounded and the apex tapered, and the phallophorus has a unique ventral “pouch” emerging from the distal margin (Fig. 250; Lonsdale 2014: fig. 62).

#### Species description

##### Euhexomyza winnemanae

Taxon classificationAnimaliaDipteraAgromyzidae

(Malloch)

Figs 47 , 48

Agromyza winnemanae Malloch, 1913a: 314. Shewell 1953: 465.
Melanagromyza winnemanae . Frick, 1952a: 380, 1959: 367.
Hexomyza winnemanae . Spencer & Steyskal, 1986b: 250.
Euhexomyza winnemanae . Lonsdale, 2014: 503.

###### Description

**(Figs 47, 48).** Wing length 1.9 mm (♂♀). Length of ultimate section of vein M4 divided by penultimate section: 1.2. Eye height divided by gena height: 8.6. Ocellar triangle large, glossy outside of tubercle and sharply pointed anteriorly. First flagellomere broadly rounded. Clypeus broadly rounded anteriorly. Distance between crossveins as long as dm-m. Costa not extending much past R_4+5_. Lunule broad, very shallow. Base of antennae broadly separated. Face flat. Fronto-orbital plate narrow.

***Chaetotaxy***: One strongly incurved ori; three ors; setae decreasing in width and length anteriorly. Ocellar and postocellar setae well-developed. Orbital setulae reclinate. Two well-developed dorsocentrals. Acrostichal setulae in eight rows. Posteromedial tibial setae absent.

***Colouration***: Calypter and wing entirely white with veins brownish (costa darker). Halter brown. Body entirely brown to dark brown with gena and centre of frons paler.

***Genitalia***: Unknown.

###### Host.

Unknown – likely *Salix* (Salicaceae).

###### Distribution.

**Canada**: ON. **USA**: MD. Gall attributed to this species observed in AB (Spencer, 1969).

###### Type material.

***Holotype* [*winnemanae*]: USA. MD**: Plummers Island, 27.vi.1909, W.L. McAtee (1♀, USNM).

***Holotype* [*albicula*]: Canada. ON**: Ottawa, 7.iv.1948, ex. gall on *Salix* sp., G.E. Shewell (1♂, CNC).

***Paratype* [*albicula*]: Canada. ON**: Dryden, 12–13.vi.1960, Kelton and Whitney (1♀, CNC).

###### Comments.

**Figures 45–51.**
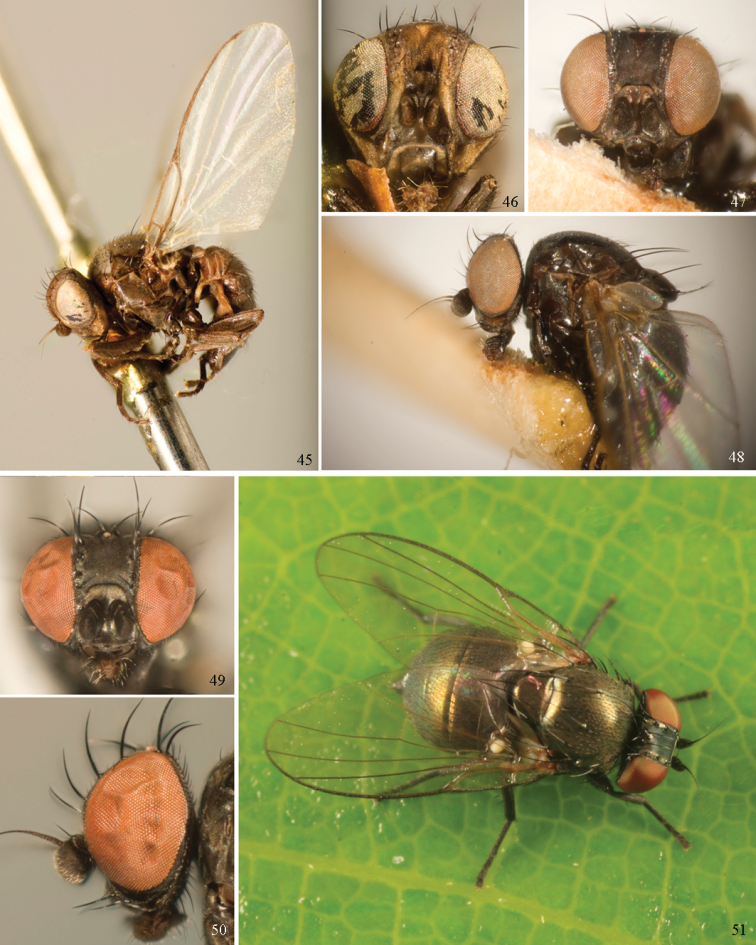
*Euhexomyzaalbicula* Spencer, male paratype **45** lateral **46** head, anterior **47, 48***Euhexomyzawinnemannae* (Malloch), female holotype **47** head, anterior **48** lateral **49–51***Japanagromyzaviridula* (Coquillett) **49** head, anterior **50** same, lateral **51** dorsal (T. Murray – Massachusetts, Plymouth).

*Euhexomyzaalbicula* (Spencer) stat. reinst., comb. nov. (Figs 45, 46, 250) was included as a junior synonym of *H.winnemanae* by Spencer and Steyskal (1986b), but following examination of the types of both species, both are recognised here as distinct, and the former is resurrected from synonymy. Aside from having white wings and a similar body size, the two species are strikingly dissimilar. *Euhexomyzaalbicula* differs as follows: eye height divided by gena height 2.0–2.1; eye relatively small and broadly surrounded by fronto-orbital plate, parafacial and occiput when seen laterally; fronto-orbital plate broad and bulging (more so medially); two ors, two ori (not much longer than ocellar seta); ocellar triangle broad, rounded apically and subshiny; first flagellomere smaller, ovate and with longer hairs; lunule semi-circular and bulging; clypeus broader medially; ocellar and postocellar setae not much longer than ocellar tubercle; scutellum with second small pair of distolateral scutellar setae well-developed and with additional seta on left side in female; veins white, excluding costa, subcosta and base of Rs; calypter margin and hairs brown; body subshiny and light brown to brown (not dark brown).

One additional pair of lateral scutellar setae is also present in the northeastern North American species *Euhexomyzasalicis* (Malloch), which has the costa extending to vein M_1+2_ (not R_4+5_).

##### Japanagromyza

Taxon classificationAnimaliaDipteraAgromyzidae

Sasakawa

Japanagromyza Sasakawa, 1958: 138. Type species: Agromyzaduchesneae Sasakawa, 1954, by original designation. Spencer 1969: 62; Spencer and Steyskal 1986b: 53; Lonsdale 2013: 445.
Geratomyza Spencer in Spencer and Stegmaier 1973: 140. Spencer 1984 [synonymy].

*Japanagromyza* is species-poor in North America north of Florida, but the species *J.viridula* (Coquillett) is relatively abundant from the east coast to Alberta, Kansas and Arizona, and the unusual *J.rutiliceps* (Melander) is known from Montana to California. The genus, which was redefined in Lonsdale (2013), has one pair of acrostichal setae and/or a lateromedial seta on the mid tibia. *Agromyza* also has developed acrostichal setae, but it differs in having a stridulatory file. Those *Japanagromyza* without acrostichals are superficially similar to *Melanagromyza*, but species in the latter genus have a longer ocellar triangle, more pronounced fronto-orbital plates, and often dorsally setose eyes.

The majority of species occurring in the New World can be characterised as follows: colour black (or with iridescence); only two pairs of widely spaced dorsocentrals; knob of halter white (base of knob and stem sometimes variably brown, and occasionally entirely dark, as in *J.brooksi* Spencer from Ontario); fore tibia with lateromedial seta (absent in *J.brooksi* and a number of non-Nearctic species); surstylus often long and narrow; male cercus very large and often bearing large tubercle-like setae (similar structures are found in some *Agromyza*); hypandrium with long, narrow, ventrally curving apodeme apically; phallus long, narrow and clear (morphology much more varied globally), and with ejaculatory apodeme sometimes very thin or atrophied. The unusual *J.rutiliceps* (Melander) from the western United States differs in most of these features, and its placement is uncertain.

##### Japagromyza viridula

Taxon classificationAnimaliaDipteraAgromyzidae

(Coquillett)

Figs 49–51 , 251–256

Agromyza viridula Coquillett, 1902: 189. Frick 1952a: 374, 1959: 359.
Japanagromyza viridula . Spencer, 1966a: 3, 1969: 63; Spencer and Steyskal 1986b: 55; Scheffer et al. 2007: 770; Eiseman and Lonsdale 2018: 16.

###### Description

**(Figs 49–51).** Wing length 2.7–3.2 mm (♂), 3.1–3.2 mm (♀). Length of ultimate section of vein M4 divided by penultimate section: 0.6–0.7. Eye height divided by gena height: 8.0–8.3. Ocellar triangle faintly outlined. Fronto-orbital plate and ocellar tubercle subshiny. Lunule height 1/2 width, silvery pruinose; lunule and face broad and well-sclerotised with face flat. Clypeus broadly rounded. Notum and head with light dusting of pruinosity. Arista virtually bare.

***Chaetotaxy***: All setae well-developed and setulae short and sparse. Two ori; two ors (widely separated). Two postsutural dorsocentrals. Ten rows of acrostichal setulae; acrostichal setula present. Postocellar and ocellar setae well-developed. Fore tibia with one lateral seta; mid tibia with two posteromedial setae.

***Colouration***: Setae black (setulae on ocellar tubercle sometimes golden-brown). Body predominantly dark brown; thorax (less so on pleuron) and abdomen with metallic green reflection, and epandrium faintly to distinctly reddish brown. Wing veins brown. Calypter and halter white.

***Genitalia***: (Figs 251–256) Surstylus fused to epandrium; suture obliterated; very narrow, apex rounded, nearly as high as epandrium; inner surface with patches of numerous tubercle-like setae distally and basally; twisted, inner face visible posteriorly. Hypandrium large, elongate with very long, stout, apical process; inner lobe bare, narrow and transverse. Postgonite broad, ovate, flat, and bare. Phallophorus small, fused to basiphallus. Basiphallus a single plate dorsal to right lateral. Hypophallus slightly shorter than basiphallus, dark, linear. Distiphallus very long, pale, and relatively straight, composed of longer flagellum on left side and shorter flagellum on right side, connected by thin membrane; often longer than illustrated. Mesophallus indistinct. Ejaculatory apodeme narrow, blade reduced with very shallow basolateral process; sperm pump unsclerotised with minutely spinulose ventral surface.

###### Hosts.

Fagaceae – *Quercus* spp., *Castaneamollissima* (Eiseman and Lonsdale 2018).

###### Distribution.

**Canada**: NB*, NS, ON, QC*. **USA**: DC, GA, IN, KS, MA, ME, NC, OK, PA, SC, TN, VA. Puerto Rico.

###### Type material.

***Holotype*: USA. DC**: “June”, “Collection Coquillett” (1♂, USNM; Type No. 6660).

###### Paratypes examined.

**USA. DC**: “DC” (1♀, USNM), **MA**: Beverly, 29.vi.1876, Burgess (1♀, USNM). **Puerto Rico.** “Porto Rico”, Aguadilla, i.1899, A. Busck (1♀, USNM).

###### Additional material examined.

142♂ 97♀ 1?, CNC, USNM. **Canada. NB**: Kouchibouguac N.P., 8.vi.1977, J.R. Vockeroth, Code – 5222V, Code – 52336, CNC358580, CNC358581, CNC358582 (3♀, CNC), 9.vii.1977, J.F. McAlpine, Code – 6023Q, CNC358583 (1♀, CNC), **QC**: Rigaud, 11.vi.1981, J.R. Vockeroth, CNC358579 (1♂, CNC).

###### Comments.

Superficially, *Japanagromyzaviridula* can be mistaken for species of *Melanagromyza* because of its metallic green colouration, but it can be easily separated by the single pair of stout prescutellar acrostichal setae.

There was an error produced in the *Japanagromyza* key in Spencer and Stegmaier (1973) that was perpetuated in Spencer and Steyskal (1986b) and Wiegmann (1991), leaving the only Delmarva species unidentifiable. *Japanagromyzaviridula* was differentiated from U.S. congeners with prescutellar acrostichal setae (*J.inaequalis* (Malloch), *J.aequalis* Spencer and *J.polygonivora* Wiegmann (= *J.polygoni* Spencer) by having the “mesonotum predominantly black”. Upon examination of the types of all of these species deposited in the USNM, however, there appears to be little, if any difference in the colour of the notum. Instead, *J.viridula* can be diagnosed by the arista, which is virtually bare (hairs not as long or longer than width of central filament) by examination of the male genitalia, or sometimes by the setulae on the ocellar tubercle, which are black or golden-brown (always black in the remaining species, which are only found in and south of Florida).

##### Melanagromyza

Taxon classificationAnimaliaDipteraAgromyzidae

Hendel

Melanagromyza Hendel, 1920: 114. Type species: Agromyzaaeneoventris Fallén, 1823, by original designation. Frick 1952a: 375; Spencer 1969: 64; Spencer and Steyskal 1986b: 18; Lonsdale 2014: 495; Shi and Gaimari 2015: 10.
Limnoagromyza Malloch, 1920: 147. Type species: diantherae Malloch, 1920, by original designation. Frick 1952a [synonymy].

Many *Melanagromyza* have a noticeably metallic green, blue, or coppery sheen on the abdomen (not including the black oviscape), which is also often present on the notum, but this feature should not be used as diagnostic in and of itself as a metallic sheen is also found in a handful of other Agromyzinae. This sheen is usually very faint in these other taxa, but some *Japanagromyza* species, including the relatively abundant eastern species *J.viridula*, are strikingly green. Conversely, those *Melanagromyza* with little or no metallic colouration can be easily mistaken for some *Euhexomyza* and *Ophiomyia*, although dorsally setulose (not bare) eyes, a wider fronto-orbital plate and a broadly rounded clypeus will reveal their generic affiliation.

Externally, the Delmarva *Melanagromyza* are best diagnosed by an absence of characters found in other local Agromyzinae: there are no prescutellar acrostichals (present in *Agromyza* and *Japanagromyza*) or a stridulatory file on syntergite 1+2 (*Agromyza*); the clypeus is usually broadly rounded (apically truncated in all *Ophiomyia*), but if the anterior margin is straight, the lateral corners are rounded; the eye is usually setulose dorsally (always bare or very sparsely short-setulose in *Ophiomyia*); the gena is never strongly produced anteriorly, there is never a vibrissal fasciculus, and the facial keel is never prominent if present (many *Ophiomyia*).

The male genitalia are most characteristic of the genus: the basiphallus is short, symmetrical and U-shaped or ring-like; the epandrium sometimes has a small posterodistal spine behind the surstylus (not always visible in lateral view); the ventrobasal surface of the distiphallus has one pair of thin tubules flanking the mesophallus (unconfirmed in some non-Nearctic species).

### ﻿Key to the Delmarva *Melanagromyza*

**Table:** 

1	Calypter margin and hairs dark brown. Eye always bare or with sparse setulae	2
–	Calypter margin and hairs white. Eye almost always setulose dorsomedially	3
2	Fronto-orbital plate and parafacial broad and easily viewed laterally. Gena highest posteriorly. Four ori. Abdomen with light coppery tint. Wing length 2.8–3.6 mm. Basiphallus forming complete ring (Figs 269, 270). Distiphallus broad and stout	***M.burgessi* (Malloch)**
–	Fronto-orbital plate and parafacial narrow, inconspicuous laterally. Gena highest at midpoint. Two ori. Abdomen with green metallic sheen. Wing length 1.9–2.3 mm. Basiphallus U-shaped (Figs 291, 292). Distiphallus thin and gracile	***M.minimoides* Spencer**
3	Gena at its highest near anterior margin of eye (Figs 52, 53). Distiphallus with dark distolateral plate and prominent dorsomedial process	***M.buccalis* Spencer**
–	Gena highest near midpoint of eye. Distiphallus not as above	4
4	Four fronto-orbital setae; anterior 2 pairs (ori) very widely spaced (Fig. 57). Surstylus sometimes with elongate tubercle-like setae (Figs 318, 319). Proepiphallus narrow, straight and V-shaped. Basiphallus open ventrally	***M.virens* group (5)**
–	Usually more than 4 fronto-orbital setae, at least on one side; distance between anterior setae not much larger than distance between any other pair of setae. Tubercle-like setae on surstylus all short. Proepiphallus rounded laterally, upcurved anteriorly and usually with separate halves. Basiphallus sometimes forming a ring	**10**
5	Head (seen laterally) broadest near middle, i.e., without bulging frons. Fronto-orbital plate relatively narrow, slightly wider behind midpoint. Seen laterally, distiphallus gradually tapering apically and with 1 pair of posteromedial bulges/carinae dorsomedially (Fig. 287)	***M.matricarioides* Spencer**
–	Head (seen laterally) usually broadest dorsally, with fronto-orbital plate bulging anterodorsally. Fronto-orbital plate usually swollen medially and narrowed anteriorly. Distiphallus abruptly widened on basal 1/2 and without clear posteromedial bulge	6
6	Wing length 1.8–2.6 mm. Fronto-orbital plate often 1/3 or 1/4 width of frons (Fig. 56). Distiphallus relatively short and squat	7
–	Wing length 2.5–3.5 mm. Fronto-orbital plate swollen to only 1/4 width of frons (Fig. 54), never 1/3. Distiphallus more elongate	8
7	Fronto-orbital plate (seen dorsally) swollen medially, usually at least 1/3 width of frons (sometimes 1/4 width). Male orbital setulae conspicuously long and bushy compared to setulae on eyes (Fig. 57). Wing length 1.8–2.4 mm. Surstylus narrow and with elongate tubercle-like setae (Figs 318, 319). Mesophallus longer, held slightly away from distiphallus. Distiphallus slender apically (Figs 322, 323)	***M.virens* (Loew)**
–	Fronto-orbital plate swollen to only ¼ width of frons, never 1/3. Male orbital setulae slightly longer than ommatrichia, sometimes barely so, relatively sparse. Wing length 1.8–2.6 mm. Surstylus broader and with only short tubercle-like setae at apex (Fig. 297). Mesophallus slender, held close to distiphallus. Distiphallus bulging laterally along distal surface (Figs 299, 300)	***M.splendida* Frick**
8	Notum and abdomen with green shine that may sometimes also be partially coppery. Distal edge of surstylus ~ 1/3 height of epandrium. Basiphallus overlapping base of distiphallus (Figs 326, 327). Large, dark distoventral plate of distiphallus (viewed laterally) continuing past point of insertion of mesophallus	***M.virginiensis* Spencer**
–	Notum with green to bluish tint. Abdomen green (*M.vernoniae* bronze-green in IN and blue in MN). Distal edge of surstylus at least 1/2 height of epandrium. Basiphallus separate from distiphallus, if only slightly. Dark distoventral plate of distiphallus (viewed laterally) never continuing past point of insertion of mesophallus	9
9	Orbital setulae much longer than ommatrichia, relatively bushy. Notum with blue tint that may become greenish posteriorly (entirely green in IN). Distiphallus gradually narrowing apically; distal section straight (seen laterally); basal section smooth; with inner spinulose patch. Phallophorus longer than wide (Figs 306, 307)	***M.vernoniae* Steyskal**
–	Orbital setulae short, as long as ommatrichia. Notum with faint green tint. Abdomen shiny green. Distiphallus only wide near base, with remainder long and curved (seen laterally); basal section strongly convoluted; inner spinulose patch strongly reduced to absent. Phallophorus higher than long (Fig. 312)	***M.vernoniana* Steyskal**
10	Large and stout-bodied; wing length 3.0–5.0 mm. Three or 4 ori, rarely 2. Abdomen often with blue metallic shine, at least posteriorly. Distiphallus stout and broad. No space between apex of basiphallus and base of distiphallus	**11**
–	Smaller and more slender; wing length 2.8–3.4 mm. Two or 3 ori. Abdomen blue, green, or coppery. Distiphallus small and slender. Membranous space between basiphallus and base of distiphallus distinct	**12**
11	Ocellar tubercle with thin strip of setulae extending to base of postocellar. Eye bare. Three dorsocentral setae. First flagellomere as high as gena. Postpronotum with large orange spots anteriorly and laterally. Presutural scutum with faint, narrow pruinose strips medially. Distiphallus broad and rounded with dorsomedial process; internal surface minutely tuberculate; ventrolateral tubules stout and broadly looped; outer surface not sculptured (Figs 275, 276). Basiphallus broad medially, and thin and ill-defined laterally. Metepiphallus without spines	***M.diantherae* (Malloch)**
–	Setulae restricted to anterior 1/2 of ocellar tubercle. Eye setulose. Two or 3 dorsocentral setae. First flagellomere narrower than gena. Postpronotum without orange spots. Scutum without pruinose strips. Distiphallus longer than wide with stout base and long ventromedial plate; with one pair of spinulose internal structures; ventrolateral tubules nearly indistinct laterally; with sculptured lateral surface (Figs 283, 284). Metepiphallus with spines	***M.glyptos* sp. nov.**
12	Distiphallus separated from basiphallus by much more than height of basiphallus (Figs 257, 258)	***M.angelicae* (Frost)**
–	Distiphallus separated from basiphallus by less than height of basiphallus	**13**
13	Wing length 2.8–3.4 mm. Mid tibia with 2 posteromedial setae. Apex of distiphallus sharply upturned (Figs 303, 304). Basiphallus forming complete ring	***M.subvirens* (Malloch)**
–	Wing length 2.1–2.8 mm, sometimes up to 3.1 mm. Mid tibia with 1 or 2 posteromedial setae. Distiphallus not upcurved apically. Basiphallus U-shaped	**14**
14	Surstylus relatively small and angled with posterior corner slightly produced. Ventromedial tubules on distiphallus shallowly to strongly looping below main body of distiphallus (Figs 293, 294). Distiphallus separated from phallophorus by height of basiphallus; sides of distiphallus flared outwards towards apex when viewed ventrally, and gradually narrowed apically when viewed laterally. Distal margin of ejaculatory apodeme pointed	***M.rutella* sp. nov.**
–	Surstylus more robust with distal margin broad and straight with posterior corner shallow. Ventromedial tubules on distiphallus held closely against distiphallus. Distiphallus separated from phallophorus by nearly 2 × height of basiphallus; abruptly narrowed on distal 1/2 (seen laterally); shape gently tapering (viewed ventrally). Distal margin of ejaculatory apodeme rounded	**15**
15	Wing length 2.6–2.7 mm. Ocellar triangle nearly reaching lunule or ending at medial ori. Eye height divided by gena height 5.3. Notum and abdomen faintly greenish. Mid tibia with 1 medial seta. Epandrium deeper dorsally (Fig. 277). Distiphallus (viewed laterally) with strong medial bulge before a strong apical constriction (Fig. 279)	***M.eoflacensis* sp. nov.**
–	Wing length 2.6–3.0 mm. Fronto-orbital plate ending at anterior ors or posterior ori. Eye height divided by gena height 3.8–4.8. Notum more evenly and strongly greenish, and abdomen with very strong green shine. Mid tibia with 1 long and 1 shorter medial seta. Epandrium shallower dorsally (Fig. 261). Distiphallus (viewed laterally) more broadly bulging medially and very gently sloping to apex, not sharply constricted (Fig. 263)	***M.brunkei* sp. nov.**

#### Species descriptions

##### Melanagromyza angelicae

Taxon classificationAnimaliaDipteraAgromyzidae

(Frost)

Figs 257–260

Agromyza angelicae Frost, 1934: 40. Frick 1952: 377, 1959: 362; Steyskal 1981: 38; Spencer and Steyskal 1986b: 24.

###### Description.

Wing length 2.8–3.2 mm (♂), 3.2–3.9 mm (♀). Length of ultimate section of vein M4 divided by penultimate section: 0.6–0.9. Eye height divided by gena height: 3.1–5.6. Clypeus broadly rounded. Ocellar triangle and fronto-orbital plate subshiny, ill-defined, extending slightly past posterior ori. Fronto-orbital plate slightly visible laterally (more pronounced in male). Gena rounded, highest behind midpoint of eye.

***Chaetotaxy***: Four ori with anterior seta strongly reduced to absent, and posterior seta sometimes appearing as third ors; two ors. Orbital setulae erect, in two, sometimes three irregular rows, sometimes with several reclinate, but becoming proportionately more proclinate anteriorly; widely spaced from eye margin. Eye pilose dorsomedially. Two dorsocentral setae. Acrostichal setulae in eight irregular rows. Mid tibia with two posteromedial setae.

***Colouration***: Body, including halter, dark brown in base colour. Gena, parafacial, distal margin of pedicel and inner-distal margin of fronto-orbital plate paler. Notum with faint greenish metallic shine (faded anteriorly). Calypter margin and hairs white. Abdomen metallic green.

***Genitalia***: (Figs 257–260) Epandrium shallow dorsally, with small posterodistal spine. Surstylus ~ 3/5 length distal region of epandrium, with broad distal margin shallowly angled; with several dense, irregular rows of tubercle-like setae on inner surface. Metepiphallus with several small spines on thickened venter and broad spinulose ridge on thickened lateral section. Proepiphallus short and U-shaped. Basiphallus U-shaped with dorsum broad, separated from mesophallus by several times its own height. Distiphallus relatively small with base not exceeding that of mesophallus; ventrolateral tubules small, inconspicuous; swollen basal section longer than flatter plate-like distoventral region, which has sides gradually narrowed.

###### Host.

*Angelicaatropurpurea*, possibly other *Angelica* spp. (Spencer and Steyskal 1986b).

###### Distribution.

**USA**: DE*, MD*, NY, OH.

###### Type material.

***Holotype*: USA. NY**: Ithaca, [reared as a stem borer on *Angelicaatropurpurea*], A.S. Mills (1♂, USNM). [Not examined]

###### Paratypes examined

**. USA. NY**: Same data as holotype, 26.iii.1926 (1♀, USNM), 27.iii.1926 (1♂, USNM), 29.iii.1926 (1♀, USNM), 16.iv.1926 (1♂ 1♀, USNM), 17.iv.1926 (2♂, USNM), 21.iv.1926 (1♀, USNM), [no date] (2♂, USNM).

###### Additional material examined.

**USA. DE**: Wilmington, 9.vi.1974 (2♂, UDCC), **MD**: Cabin John, 20.vi.1931, A.L. Melander (1♂, USNM), Montgomery Co., Bethseda, 28.iv.1968, G. Steyskal (1♂, USNM), 4mi SW of Ashton, 3.vi.1984, G.F. and J.F. Hevel (1♂, USNM), **OH**: 3 mi. E Streetboro, 20.iii.1970, Biol Note No. 17-1, D. Witwer (1♂ [with puparium], USNM), Biol Note No. 17-6 (1♂ [with puparium], USNM), Biol Note No. 17-11 (1♂ [with puparium], USNM), Biol Note No. 17-12 (1♀ [with puparium], USNM), Biol Note No. 17-13 (1♂ [with puparium], USNM).

###### Comments.

*Melanagromyzaangelicae* is only known from the eastern United States. Previous records from the western United States and Europe have been determined to belong to other *Melanagromyza* (Spencer and Steyskal 1986b).

Similar described species with a wide space between the basiphallus and meso/distiphallus include *Melanagromyzahicksi* Steyskal, collected in Ontario and New York and reared fom *Althaearosea*; this species is very robust-bodied, has four or five ori, a gena 1/5–1/4 eye height, a mesophallus that projects further basally past the base of the distiphallus, and a distiphallus that is more strongly tapered apically (Steyskal 1981: fig. 1). *Melanagromyzalomatii* Steyskal is known from Oregon and was reared from *Lomatiumnudicaule*, and is of a similar size with a similarly high gena; this species has four or five ori, the facial carina is slightly pronounced and wide, the notum and abdomen have a faint bluish shine, the calypter margin and hairs are brown, and the distiphallus is relatively short and stout, and while strongly tapered in lateral view, it is broad and truncated in ventral view (Steyskal 1981: fig. 4). *Melanagromyzapanacis* Steyskal is known from Indiana and Ohio, and has been reared from *Panaxquinquefoilus*; it is slightly smaller (2.5–2.8 mm), the phallus is proportionally shorter, and the distiphallus is slightly larger and stouter with slightly less space between its base and the slightly stouter basiphallus.

##### Melanagromyza brunkei sp. nov.

Taxon classificationAnimaliaDipteraAgromyzidae

http://zoobank.org/D7407218-C78D-4AAA-8F0B-FB03A4A84DB3

Figs 261–263

###### Description.

As described for *M.eoflacensis*, except as follows: three ori; wing length 2.6–3.0 mm (♂); fronto-orbital plate ending at anterior ors or posterior ori; eye height divided by gena height 3.8–4.8; head very dark brown, without slightly paler brown regions excluding very narrow inner margin of fronto-orbital plate; notum more evenly greenish and with matching, but brighter abdomen; VA male with additional medial dorsocentral on left side as long as anterior dorsocentral; mid tibia with one long and one shorter seta posteromedially; dorsomedial bulge on distiphallus less prominent, and seen laterally, gradually tapering to apex, not abruptly narrowed; seen ventrally, distiphallus with stouter, more prominent narrow plates flanking distomedial tube (Figs 262, 263).

###### Etymology.

The specific name is a patronym for the collector of the holotype.

###### Host.

Unknown.

###### Distribution.

**USA**: IN, VA.

###### Type material.

***Holotype*: USA. VA**: Giles Co., Ripplemead, Rte 460 bridge, 37°19'43"N, 80°40'48"W, 11–25.v.2008, A. Brunke, debu00304024 (1♂, DEBU).

***Paratype***: **USA. IN**: Vincennes, v-6, swept from wint. wheat (1♂, USNM).

###### Comments.

*Melanagromyzabrunkei* is difficult to differentiate from *M.eoflacensis*, but there are subtle external differences and the genitalia are clearly different when viewed side-by-side, with the profile of the distiphallus being especially diagnostic (see key).

##### Melanagromyza buccalis

Taxon classificationAnimaliaDipteraAgromyzidae

Spencer

**Figures 52–63.**
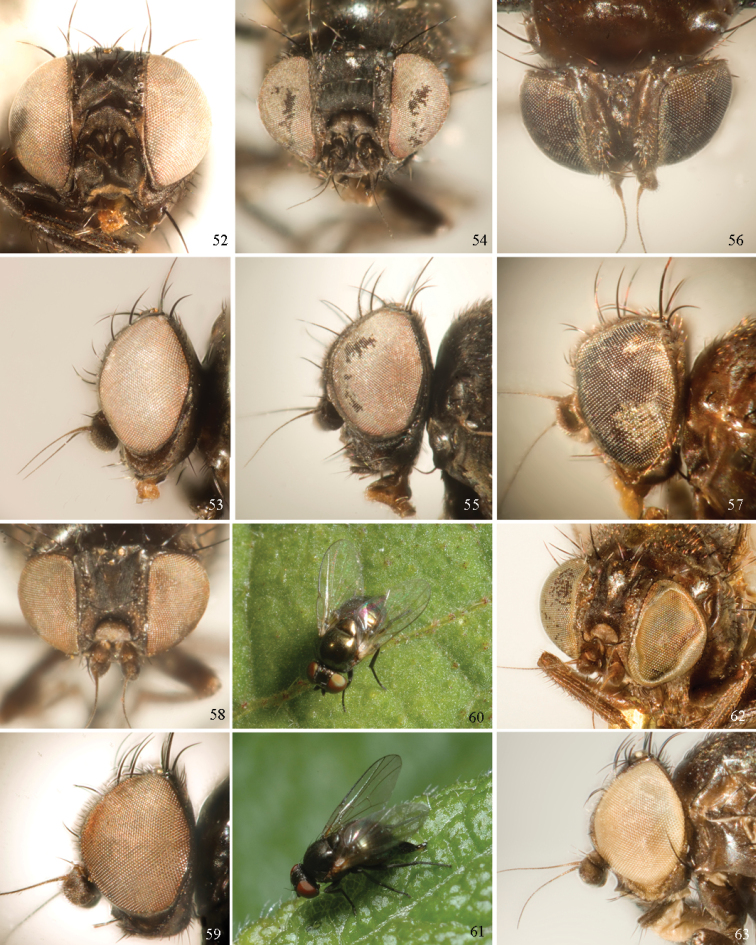
*Melanagromyzabuccalis* Spencer, head **52** anterior **53** lateral **54, 55***M.glyptos* sp. nov., head **54** anterior **55** lateral **56, 57***M.virens* (Loew), head **56** anterior **57** lateral **58, 59***M.virginiensis* Spencer, head **58** anterior **59** lateral **60***Melanagromyza* sp., female (S.A. Marshall – Cuba) **61***Melanagromyza* sp., female (S.A. Marshall – Ontario, Hornings Mills) **62, 63***M.virens* (Loew), lectotype, head **62** anterolateral **63** lateral.

Figs 52 , 53 , 264–268

Melanagromyza buccalis Spencer, 1969: 67. Spencer and Steyskal 1986b: 243; Shi and Gaimari 2015: 10; Eiseman and Lonsdale 2018: 17.

###### Description

**(Figs 52, 53).** Wing length 1.9–2.4 mm (♂), 2.2–2.5 mm (♀). Length of ultimate section of vein M4 divided by penultimate section: 0.6–0.9. Eye height divided by gena height: 3.9–5.6. Gena relatively broad and curved anteriorly with highest point anterior to midpoint of eye. Clypeus slightly truncated to rounded anteromedially. Face with narrow, shallow ridge. Fronto-orbital plate sometimes slightly to moderately swollen medially, narrowed anteriorly and with setae slightly inset (similar to species in *M.virens* group). Ocellar triangle short, nearly equilateral and with anterior point open.

***Chaetotaxy***: Two to four ori, but if only two ori, then ori sometimes widely spaced as in *M.virens* group; two ors. Orbital setulae short, in two to three irregular rows, erect to reclinate with inner row partially inclinate. Eye pilose dorsomedially. Two dorsocentral setae. Acrostichal setulae in eight irregular rows. Mid tibia with two posteromedial setae.

***Colouration***: Body, including halter, dark brown in base colour. Ocellar triangle and fronto-orbital plate sometimes light brown. Scutum brown with sometimes indistinct greenish shine. Calypter margin and hairs white. Abdomen metallic green, or less commonly, coppery-green.

***Genitalia***: (Figs 264–268) Epandrium with small posterodistal spine. Metepiphallus with several clustered spine-like ventral projections and numerous narrow lateral ridges. Proepiphallus V-shaped. Basiphallus U-shaped. Distiphallus separated from phallophorus by slightly less than length of basiphallus; base of distiphallus and mesophallus level; distiphallus narrow, long, with sides parallel, with short, thick dorsomedial lobe, and one pair of internal spinulose patches; distolateral plate forming conspicuous, dark oblique band. Ejaculatory apodeme subovate with stem reduced and margin clear.

###### Host.

Unknown, but Spencer and Steyskal (1986b) speculated Asteraceae, and Shi and Gaimari (2015) reported one instance of rearing from an unknown legume in California; some adults collected around a variety of families including Apiaceae, Salicaceae, Rutaceae, Hydrangeaceae, Rosaceae and Asteraceae (Shi and Gaimari 2015; Eiseman and Lonsdale 2018), and *Baccharis* (Asteraceae) from Maryland.

###### Distribution.

**Canada**: MB*, NB*, ON, QC. **USA**: AZ, CA, CO, DE*, GA*, IA*, IL*, IN*, MA, MD, MO, NC*, NH*, NJ*, NM*, NY, PA*, TN*, VA, WV*.

###### Type material.

***Holotype*: Canada. QC**: Lake Bernard, 7.viii.1938, G.E. Shewell, CNC258290 (1♂, CNC).

***Paratypes*: Canada**. **NS**: Truro, 8.vii.1913, CNC Type No. 10360, CNC358296 (1♂, CNC), **ON**: Mer Bleue, 3.vi.1938, A.R. Brooks, CNC358295 (1♂, CNC), Osgoode, 18.vi.1964, J.R. Vockeroth, CNC358292, CNC358322 (1♂,1♀, CNC), Ottawa, 2mi E, Cyrville Road, 31.v.1965, B.V. Peterson, CNC358321 (1♀, CNC), Ottawa, CEF, on larch, 3.viii.1962, J.F. McAlpine, CNC358302 (1♂, CNC), Ottawa, 20.vi.1954, D. Cobb, CNC358298, CNC358303, CNC358305, CNC358309, CNC358312 (5♂, CNC), 25.viii.1908, J.M. Fletcher, CNC358299 (1♂, CNC), 7.x.1947, G.E. Shewell, CNC358307 (1♂, CNC), 9.vi.1962, J.R. Vockeroth, CNC358300, CNC358311 (2♂, CNC), Simcoe, 15.vi.1939, G.E. Shewell, CNC358301 (1♂, CNC), 19.vi.1939, CNC358319 (1♀, CNC), 20.vi.1939, CNC358310 (1♂, CNC), 22.vi.1939, CNC358308 (1♂, CNC), 23.vi.1939, CNC358320 (1♀, CNC), 4.vi.1939, CNC358306 (1♂, CNC), Spencerville, 14.viii.1939, Hammond, en copulae, CNC358316 (2♂♀, CNC), **QC**: Wakefield, 20.vi.1946, G.S. Walley, CNC358291 (1♂, CNC), Abbotsford, 22.vi.1937, G. Shewell, CNC358293 (1♂, CNC), Hull, 25.ix.1923, C.H. Curran, CNC358294 (1♂, CNC), Knowlton, 1.viii.1929, L.J. Milne, en copulae, CNC358314 (2♂♀, CNC), L’Assumption, 7.viii.1936, Shewell, CNC358304, CNC358313, CNC358315, CNC358318 (2♂ 4♂♀, CNC), Lac Bernard, 7.viii.1938, G.E. Shewell, CNC358317 (1♀, CNC).

###### Additional material examined.

**Canada. MB**: Aweme, 20.viii.1917, N. Criddle, CNC358381 (1♀, CNC), 27.viii.1917, CNC358382 (1♀, CNC), **NB**: Pokeshaw, 47°47'N, 65°14'W, 27–30.vi.2011, S.E. Brooks, Malaise trap, CNC423058 (1♂, CNC), Saint-Jacques NB Botanical Garden, 47°26'20"N, 68°23'39"W, 27.vii.2013, O. Lonsdale, CNC358505 (1♀, CNC), **ON**: Ottawa, 20.vi.1954, D. Cobb, CNC358325 (1♂, CNC), St. Lawrence Is. Nat. Park, Grenadier I. Centre, 14.viii.1975, R.J. McMillan, Malaise trap, Code 2-263D, CNC358379 (1♀, CNC), 21.viii.1975, Code 2-278S, CNC358380 (1♀, CNC), Thornhill, 30.v.1964, J.R. Vockeroth, CNC358326 (1♂, CNC), **QC**: St. Ann-Perade, 6.viii.1930, A.L. Melander (1♂, USNM), Montreal, 8.viii.1950, A.L. Melander (1♂, USNM). **USA. AZ**: Chiricahua Mts., S.W.R.S., 5400', 30.iv.1979, K.N. Barber (2♂, DEBU), **CA**: Victorville, 16.v.1955, W.R.M. Mason, CNC358467 (1♂, CNC), “UpStaAnaRiv”, Cienaga, 28.v.1948, J.L. Sperry (1♀, USNM), Vacaville, 19.iv.1949, A.T. McClay (1♀, USNM), Davis, 14.vi.1953, E.I. Schlinger (1♂, USNM), Toulumne Co., Summit Sonora Pass, 9.viii.1948, sweeping *Salix* eastwoodiae Ckll., Lot No. 108-1, K.E. Frick (3♂, USNM), Summit Sonora Pass, 10.viii.1948, Lot No. 116-1, K.E. Frick (1♀, USNM), San Diego Co., Warmer Spr., 30.viii.1955, E.I. Schlinger (1♂, USNM), Berkeley, 9.vii.1917, J.M. Aldrich (1♂, USNM), 8.vii.1917 (2♂, USNM), Hemet Lake, 13.vi.1961, 500', J.G. Chillcott, CNC358533 (1♂, CNC), **CO**: Boulder, Valmont Butte, 1615 m, 1.vi.1961, J.R. Stainer, CNC358378 (1♀, CNC), Idaho Springs, 5mi SW, 2621 m, 27.v.1961, J.G. Chillcott, CNC358324 (1♂, CNC), Mt. Vernon Cn. nr. Golden, 2194 m, 31.vii.1961, C.H. Mann, CNC358468 (1♂, CNC), **DC**: Washington, “C 1 Aug 1956”, P.H. Arnaud Jr. (1♂, USNM), Washington, “viii.5”, J.M. Aldrich (1♀, USNM), Washington, 17.viii.1915, A.L. Melander (1♂ 1♀, USNM), Chain Bridge, 8.v.1928, J.M. Aldrich (1♀, USNM), **DE**: Dover, 24.vi.1939, A.L. Melander (1♂, USNM), Newark, 31.v.1974 (1♀, UDCC), Newark, 25.viii.1974, D. Buntin (1♀, UDCC), **GA**: Pine Mt., 1mi North, 12.vii.1957, W.R. Richards, CNC358347 (1♀, CNC), Rabun Bald, 9.viii.1957, W.R. Richards, CNC358355–358357 (3♀, CNC), Rabun Co., 13.vii.1957, W.R. Richards, CNC358354 (1♀, CNC), Warwoman Crk., 4.vi.1957, W.R. Richards, CNC358358 (1♀, CNC), **IA**: Ames, 19.vi.1947, A.R. Brooks, CNC358461 (1♀, CNC), 28.vi.1947, CNC358458 (1♂, CNC), **IL**: Champaign, 1.vi.1953, J.F. McAlpine, CNC358460 (1♀, CNC), 22.ix.1956, CNC358459 (1♂, CNC), **IN**: LaFayette, vi-10, J.M. Aldrich (1♂, USNM), vii-10 (1♂, USNM), x-14 (1♀, USNM), **MA**: New Bedford, 30.viii.1896, Mass, A.L. Melander (1♂, USNM), Horse Neck Beach, 8.viii.1896, Hough, A.L. Melander (1♂, USNM), Dennisport, C. Cod, 1.viii.1964, J.R. Vockeroth, CNC358535 (1♀, CNC), Franklin Co., Northfield, 276 Old Wendell Rd., 20.viii.2016, mating on *Erigeronannuus*, #CSE2916, CNC654071, CNC654072 (1♂ 1♀, CNC), **MD**: Plummers Isl., 20.vii.1913, R.C. Shannon (1♀, USNM), Talbot Co., McDaniel (Wades Point), 19–21.ix.1986, Malaise trap in salt marsh with flowering *Baccharis*, W.E. Steiner (2♀, USNM), Fairfax Co., Dead Run, 14.v.1914, R.C. Shannon (1♀, USNM), Montgomery Co., Colesville, W.W. Wirth, 27.vii.1976 (1♀, USNM), 28.vii.1976 (2♀, USNM), Bethseda, C.W. Sabrosky, 24.vii.1961 (1♀, USNM), Bethseda, G.C. Steyskal, 10.vii.1970 (1♂, USNM), 16.vii.1979 (1♂, USNM), 24.viii.1975 (4♂ 1♀, USNM), 4mi SW of Ashton, G.F. and J.F. Hevel, 1.ix.1981, Malaise trap (1♂ 1♀, USNM), 24.vii.1982 (3♀, USNM), 15.viii.1982 (2♀, USNM), 19.v.1985 (1♀, USNM), 29.v.1986 (2♀, USNM), 31.v.1986 (1♀, USNM), 5.vi.1986 (1♀, USNM), P.G. Co, Camp Springs, Malaise trap, G.F. Hevel, 28.viii.1979 (1♀, USNM), 6.ix.1979 (1♀, USNM), 14.ix.1979 (1♀, USNM), 15.ix.1979 (2♀, USNM), **NC**: Highlands, 3800', 21.v.1957, J.R. Vockeroth, CNC358534 (1♂, CNC), Cashiers, 12.vii.1957, W.R. Richards, CNC358351–358353 (3♀, CNC), Clingman’s Dome, 5.viii.1957, W.R. Richards, CNC358350 (1♀, CNC), Highlands, Horse Cove, 30.vii.1957, W.R. Richards, CNC358349 (1♀, CNC), Highlands, Little Bear Pen Mt., 5.viii.1957, W.R. Richards, CNC358348 (1♀, CNC), Highlands, 14.vii.1957, W.R. Richards, CNC358331 (1♂, CNC), 16.viii.1957, CNC358359, CNC358360 (2♀, CNC), 1158 m, 17.viii.1957, J.G. Chillcott, CNC358344 (1♀, CNC), 18.vii.1957, J.G. Chillcott, CNC358339 (1♂, CNC), 1158 m, 21.v.1957, J.R. Vockeroth, CNC358328 (1♂, CNC), 21.vii.1957, W.R. Richards, CNC358327, CNC358329, CNC358330 (3♂, CNC), 1158 m, 21.viii.1957, J.G. Chillcott, CNC358343 (1♀, CNC), W.R. Richards, CNC358361–358366 (6♀, CNC), 22.viii.1957, W.R. Richards, CNC358367–358370 (4♀, CNC), 1158 m, 23.viii.1957, J.G. Chillcott, CNC358338 (1♂, CNC), W.R. Richards, CNC358371 (1♀, CNC), 27.viii.1957, W.R. Richards, CNC358372, CNC358373 (2♀, CNC), 29.vii.1957, W.R. Richards, CNC358332 (1♂, CNC), 1158 m, 3.vi.1957, J.R. Vockeroth, CNC358345, CNC358346 (2♀, CNC), 5.viii.1957, J.G. Chillcott, CNC358333–358337, CNC358340–358342 (5♂ 3♀, CNC), Macon Co., Wayah Gap, 1249 m, 29.vii.1957, J.G. Chillcott, CNC358374 (1♀, CNC), **NH**: Gorham, 15.vii.1958, J.R. Vockeroth, CNC358462 (1♀, CNC), Fabyan, 30.vi.1936, A.L. Melander (1♂, USNM), **NJ**: Hemlock Falls, Aug, A.L. Melander (1♀, USNM), **NM**: Grant Co., ca. 20 mi n. Silver City, 32°57'N, 108°10'W, 7100', meadow, creek, 11.viii.2007, J.D. King, CNC358528 (1♂, CNC), **NY**: Trudeau, 15.vii.1977, S.W.T. Batra (1♂, USNM), Long Island, Cold Spring Harbor, July, A.L. Melander (1♂, USNM), **PA**: Susquehanna Co., Rushboro, 12.v.1964, J.G. Chillcott, CNC358465 (1♂, CNC), Chester Co., Toughkenamon, Stroud Res. Ctr., 39°51'37", 75°46'58", 14.ix.2007, E. Lake (2♂, UDCC), nr. Toughkenamon, Stroud Res. Ctr., N39 51 37.2, W75 46 58.2 (1♀, UDCC), 14.ix.2007, C.R. Bartlett (1♀, UDCC), Oxford, 3.ix.2000, Malaise trap, R.L. Snyder (1♀, UDCC), **TN**: Knoxville, 20.v.1957, J.R. Vockeroth, CNC358375 (1♀, CNC), **VA**: Augusta Co., Reed’s Gap, 792 m, 7.iv.1962, J.R. Vockeroth, CNC358466 (1♂, CNC), Blacksburg, 28.v.1962, J.G. Chillcott, CNC358376 (1♀, CNC), Montgomery Co., Christiansburg, ex flowers Umbelliferae, 21.vi.1962, J.G. Chillcott, CNC358377 (1♀, CNC), Montomery Co., Longshop, 548 m, 29.v.1962, J.R. Vockeroth, CNC358323 (1♂, CNC), Arlington 4-mile Run, 18.v.1977, W.N. Mathis (1♀, USNM), Veitch, 9.v.1912, J.R. Malloch (1♀, USNM), Great Falls, “vi-1”, G.E. Quinter (1♀, USNM), Falls Church, Holmes Run, W.W. Wirth, 27.vii.1960 (1♂, USNM), 6.vi.1961, light trap (1♂, USNM), Fairfax Co., Alexandria, Four Mile Run, 6.ix.1976, W.N. Mathis (1♂, USNM), Page Co., 7mi W of Lunay, 8.vii.1978, G.F. Hevel (2♀, USNM), **WV**: Greenbrier Co., Charmco, 6.ix.1983, G.F. and J.F. Hevel (1♂, USNM), **WV**: Thermopolis, 30.viii.1940, A.L. Melander (1♂, USNM).

###### Comments.

*Melanagromyzabuccalis* is a relatively abundant species most easily recognised by its angled gena, which is highest anterior to the midpoint of the eye. The distiphallus (dark distal outer casing and one pair of dark dorsolateral projections) is also diagnostic. Some specimens, even within collecting events, differ from others in being relatively dark or with a swollen fronto-orbital plate that may be accompanied by widely spaced ori, similar to the state observed in the *M.virens* group.

##### Melanagromyza burgessi

Taxon classificationAnimaliaDipteraAgromyzidae

(Malloch)

Figs 269–271

Agromyza burgessi Malloch, 1913a: 323.
Melanagromyza burgessi . Frick 1952: 378 [as synonym M.lappae (Loew)], 1953: 69, 1959: 363; Spencer and Steyskal 1986b: 244; Shi and Gaimari 2015: 14.
Melanagromyza malefica Spencer, 1981: 46. Spencer and Steyskal 1986b [synonymy].

###### Description.

Wing length 2.7–3.1 mm (♂), 2.9–3.6 mm (♀). Length of ultimate section of vein M4 divided by penultimate section: 0.7. Eye height divided by gena height: 2.2–3.0. Gena strongly angled, highest posteriorly. Fronto-orbital plate and parafacial projecting and conspicuous, obscuring base of antenna (seen laterally) and continuing under eye as cheek; fronto-orbital plate well-developed, setae slightly inset. Clypeus strongly bowed laterally with anterior margin shallowly rounded to slightly truncated medially. Ocellar triangle subshiny and ill-defined.

***Chaetotaxy***: Four or five ori; two ors. Ocellar setulae short and slightly proclinate, with setulae on posterior 1/3 sometimes reclinate. Orbital setulae short, in three rows, reclinate. Eye bare. Two dorsocentral setae. Acrostichal setulae in ten irregular rows. Three very strong anepisternal setae. Mid tibia with two posteromedial setae.

***Colouration***: Body, including halter, dark brown in base colour, sometimes with faint blue or greenish shine evident on notum. Parafacial, antenna and gena sometimes paler. Calypter margin and hairs brown. Abdomen with light coppery, greenish, or bluish shine (only coppery observed in this study).

***Genitalia***: (Figs 269–271) Epandrium with small posterodistal spine. Surstylus rounded with short setae along inner-distal margin and shorter tubercle-like setae in two to three irregular rows on inner surface. Metepiphallus very dark with serrated ventral ridges and two stout coalescing lateral ridges. Proepiphallus thick and globular. Basiphallus forming complete ring. Distiphallus separated from phallophorus by ~ 1.5 × length of basiphallus; distiphallus and mesophallus with bases level; distiphallus with long spinulose internal structures and thin distal projection subequal in length to basal section of distiphallus, which is relatively flat and abruptly ending at midpoint (seen ventrally), with only narrow distoventral plate continuing apically. Ejaculatory apodeme well-developed, typical of *Melanagromyza*, with blade relatively narrow.

###### Host.

Unknown.

###### Distribution.

**USA**: CA, CO, IL, IN, KS, MA, MD*, MI, ND, NY.

###### Type material.

***Holotype* [*burgessi*]: USA. MA**: Beverly, 2.vi.1876, Burgess (1♀, USNM; type No. 15685).

***Paratypes* [*burgessi*]: USA. ND**: Tower City, 5.vi.1906, G.I. Reeves, Webster No. 3122 (2♀, USNM), **CO**: “Colo”, “1563” (1♀, USNM).

***Holotype* [*maelifica*]: USA. CA**: San Diego Co., La Mesa, 23.iii.1962, P.A. Rude (1♂, CAS). [Not examined]

###### Additional material examined.

**USA. IN**: Lafayette, “v-20”, swept from wint. wheat (1♀, USNM), Evansville, 7.v.1914, swept from wint. wheat, J.M. Aldrich (1♀, USNM), **KS**: Manhattan, 4.v.1932, D.A. Wilbur (1♀, USNM), Manhattan, C.W. Sabrosky, 20.iv.1934 (3♂, USNM), 25.iv.1934 (1♀, USNM), **MD**: Montgomery Co., 4mi SW of Ashton, 27.v.1984, G.F. and J.F. Hevel (1♂, USNM), Bethseda, 3.vi.1972, G.C. Steyskal (1♀, USNM), **MI**: St. Joseph, 30.v.1938, C.W. Sabrosky (1♂, USNM).

##### Melanagromyza dianthereae

Taxon classificationAnimaliaDipteraAgromyzidae

(Malloch)

Figs 272–276

Limnoagromyza dianthereae Malloch, 1920: 147.
Melanagromyza dianthereae . Frick 1952a: 378, 1959: 364; Spencer and Steyskal 1986b: 22.

###### Description.

Wing length 3.4–3.9 mm (♂), 4.4–5.0 mm (♀). Length of ultimate section of vein M4 divided by penultimate section: 0.6–0.8. Eye height divided by gena height: 2.8–3.4. Male first flagellomere slightly enlarged, rounded, with longer hairs past base. Fronto-orbital plate and parafacial visible laterally, broadly rounded, and continuing under eye as very narrow cheek; fronto-orbital plate well-developed, setae slightly inset. Ocellar triangle long and narrow with anterior point open. Gena relatively short, slightly angled upwards anteriorly. Clypeus broadly rounded with anterior margin slightly thickened. Scutum with faint, thin longitudinal pruinose stripes medially.

***Chaetotaxy***: Three or four ori; two ors (one in CNC female paratype). Orbital setulae in three irregular rows, reclinate to erect, sometimes proclinate anteriorly. Ocellar setulae erect. Eye bare. Three dorsocentrals, with anterior seta close to second and ~ 3/5 length. Acrostichal setulae in ten irregular rows. Mid tibia with two posteromedial setae.

***Colouration***: Body, including halter, dark brown in base colour. Head nearly black and with greenish tint. Notum with greenish shine; anterolateral margin lighter brown with postpronotum particularly pale and with yellowish spots. Calypter margin and hairs white. Abdomen brown with green shine, sometimes becoming more blue posteriorly or with colour most pronounced medially and on posterior 1/2 of tergites.

***Genitalia***: (Figs 272–276) Epandrium without small posterodistal spine. Surstylus shallow and subtriangular, with long marginal setae and several shorter, stouter setae. Hypandrium broad and stout with lobe thick. Metepiphallus smooth; lateral portion nearly divided into separate sclerite and with two crossing ridges. Proepiphallus minute. Basiphallus U-shaped with ends tapered and dorsum very broad and ill-defined with thick transverse medial ridge. Distiphallus large, bulbous, close to phallophorus, tapered apically; with long, thin dorsomedial process; ventrolateral tubules strongly arched, level with mesophallus; internal surface shallowly textured and outer surface minutely tuberculate dorsally.

###### Hosts.

Acanthaceae – *Justiciaamericana* (formerly treated as *Dianthera*).

###### Distribution.

**USA**: DC*, IL, IN, MD, VA*.

###### Type material.

***Holotype*: USA. IL**: Muncie, 15.viii.1917, Frison and Malloch (1♀, INHS). [Not examined]

###### Paratypes examined.

**USA. IN**: Lafayette, 19.viii.1916, J.M. Aldrich, swept from Dianthera wildcat Cv. (1♂, USNM), swept from *Dianthreaamericana* (1♂, USNM), Lafayette, on Diantheraamericana, 2.vi.1915, J.M. Aldrich, CNC356825, CNC358427 (1♂ 1♀, CNC).

###### Additional material examined.

**USA. DC**: Rock Creek Park, 18.vii.1933, J.M. Aldrich (1♂ 2♀, USNM), Rock Creek Park, 14.viii.1931, on *Diantheraamericana* (1♂, USNM), **IN**: Lafayette, J.M. Aldrich, swept from *Diantheraamericana*, 11.v.1915 (1♂, USNM), 11.vi.1915 (3♂, USNM), 18.vi.1915 (2♂ 2♀, USNM), 21.viii.1916 (1♂, USNM), Lafayette, J.M. Aldrich, 18.viii.1916 (1♂, USNM), 10.vi.1915 (4♂ 5♀, USNM) 11.vi.1915 (1♂, USNM), vi-iv.1915 (1♀, USNM), 21.viii.1916 (1♀, USNM), Lafayette, on *Dianthera* 26.vi.1915 (2♂, USNM), 29.vi.1916 (1♂, USNM), “vii-14” (1♂, USNM), Lafayette, A.L. Melander, “vi-2” (3♂ 1♀, USNM), **MD**: Plummers Isl., 6.vii.1963, G. Steyskal (1♂ 1♀, USNM), Plummers Isl., K.V. Kromberin, 3.viii.1962 (1♂, USNM), 11.vii.1962 (4♂ 1♀, USNM), 9.vi.1963 (7♂ 2♀ 1?, USNM), 5.viii.1962 (1♂, USNM), 7.viii.1962 (1♂ 1♀, USNM), 1.ix.1962 (3♂ 6♀, 2?, USNM), 31.vii.1962 (2♂, USNM), **VA**: Fairfax Co., Potomac River at Scott Run, 7.vi.1955, C.W. Sabrosky (2♀, USNM).

##### Melanagromyza eoflacensis sp. nov.

Taxon classificationAnimaliaDipteraAgromyzidae

http://zoobank.org/C81180E3-9EDA-4D78-8E66-DB8AA0F5A8F3

Figs 277–280

###### Description.

Wing length 2.6–2.7 mm (♂). Length of ultimate section of vein M4 divided by penultimate section: 0.6–0.9. Eye height divided by gena height: 5.3. Clypeus broadly rounded. Ocellar triangle and fronto-orbital plate subshiny to slightly matt with triangle reaching level of mid ori or nearly reaching lunule (tapering anteriorly), and fronto-orbital plate slightly visible laterally. Gena rounded, highest behind midpoint of eye.

***Chaetotaxy***: Three ori (sometimes four on one side); two ors. Orbital setulae in two irregular rows, erect to reclinate. Eye pilose dorsomedially, hairs relatively dense. Two dorsocentral setae. Acrostichal setulae in eight irregular rows. Mid tibia with one posteromedial seta (shorter than width of tibia).

***Colouration***: Body, including halter, dark brown in base colour. Gena, parafacial, distal margin of pedicel and inner-distal margin of fronto-orbital plate paler. Notum faintly greenish (faded anteriorly), and femora sometimes also slightly reflective. Calypter margin and hairs white. Abdomen with bluish (MD-Bethseda), greenish (IN, PA) or coppery shine (MD-Plummers Isl.).

***Genitalia***: (Figs 277–280) Epandrium with small posterodistal spine. Metepiphallus with ventral surface dark and with short serrated ridge; lateral margin strongly ridged and nearly separate. Proepiphallus narrow and V-shaped with medial desclerotisation. Hypandrium stout, subtriangular and with small apical process. Basiphallus short and U-shaped; space between basiphallus and distiphallus separated by slightly less than height of basiphallus (slightly more in Lewiston, PA male, illustrated here). Base of mesophallus slightly exceeding that of distiphallus. Distiphallus with spinulose pad along inner-dorsal margin; with thicker basal section ending in bulge before much narrowed apical 1/2; base of distiphallus in IN male straighter and slightly narrower. Ejaculatory apodeme similar to that of *M.subvirens*, with distal margin darker in holotype.

###### Etymology.

The specific name indicates similarity to *M.osoflacensis* Spencer, while indicating is relative eastern distribution (*eos* – Gr. for east).

###### Host.

Unknown.

###### Distribution.

**USA**: IN, MD, PA, VA.

###### Type material.

***Holotype*: USA. MD**: Montgomery Co., Bethseda, 3.vi.1972, G. Steyskal (1♂, USNM).

***Paratype*: USA. IN**: LaFayette, 10.v.1915, swept from grass, J.M. Aldrich (1♂, USNM), **MD**: Plummers Isl., 20.iv.1921, H.S. Barber (1♂, USNM), **PA**: Lewiston, 7.vi.1940, A.L. Melander (1♂, USNM).

###### Additional material examined.

**USA. VA**: Blacksburg, 2100', 1.vi.1962, J.G. Chillcott (1♂[only wing and genitalia remaining], CNC).

###### Comments.

*Melanagromyzaeoflacensis* can be partially diagnosed by a single posteromedial seta on the mid tibia (not two), but similar species (*M.angelicae*, *M.osoflacensis*, *M.panacis*, *M. subvirens*, *M.brunkei*) may be easily misidentified as this taxon, particularly if one or both of the mid tibiae are damaged. As such, the genitalia should always be examined for verification. The phallus of *M.eoflacensis* is distinct in having a space between the basiphallus and distiphallus that is nearly equal to the height of the basiphallus, the mesophallus only slightly projects past the base of the distiphallus, and seen laterally, the distiphallus has a thick dorsobasal section ending in a pronounced bulge before it abruptly narrows (similar to the state seen in *M.angelicae*).

The terminalia of *Melanagromyzaangelicae* (Figs 257–260) differ in having a much longer distance between the basiphallus and the base of the distiphallus. *Melanagromyzasubvirens* (Figs 301–304) differs in having a ring-like basiphallus and a flared membranous carina anterodorsally, and the wing is slightly larger. The phalli of *M.osoflacensis* and *M.panacis* (Spencer and Steyskal 1986b: figs 35, 36) are more similar, but the former species has very little space between the basiphallus (with posteromedial notch) and the more gradually tapered distiphallus, the fronto-orbital plate is slightly more pronounced and the ocellar triangle is shinier; the latter species has a slightly longer distance between the basiphallus and the base of the distiphallus and it is stouter-bodied but with a similar wing length (2.5–2.8 mm) and the posterior two ori are more closely spaced. The abdomen in these species is also always shiny green, with that of *M.subvirens* sometimes also bluish. Also see comments for *M.brunkei*.

The genitalic illustration of this species was erroneously provided for *Melanagromyzaosoflacensis* Spencer in Shi and Gaimari (2015), provided by the present author, at a period when he was not aware of the identity of this new species.

The Virginia male is only tentatively included as most of the body is missing (only one wing is left on the pin) and the genitalia are preserved on a mini-prep slide on an angle that does not allow for fully confident identification, but it appears to agree with the above description.

##### Melanagromyza glyptos sp. nov.

Taxon classificationAnimaliaDipteraAgromyzidae

http://zoobank.org/ACED6C5F-3696-4795-B295-D1C5D0C399CA

Figs 54 , 55 , 281–284

###### Description

**(Figs 54, 55).** Wing length 3.6–3.9 mm (♂), 3.1–3.4 mm (♀). Length of ultimate section of vein M4 divided by penultimate section: 0.6–0.7. Eye height divided by gena height: 2.4–4.9. Gena angled dorsally on anterior 1/2, highest near midpoint. Fronto-orbital plate and parafacial broadly visible laterally, continuing under eye as distinct cheek; fronto-orbital plate well-developed, setae slightly inset. Clypeus narrowed and slightly truncated medially, sides bowed outwards. Sides of ocellar triangle shallowly concave. Parafacial and ocellar triangle subshiny.

***Chaetotaxy***: Three or four ori, sometimes with slightly larger gap between anterior two setae; two ors. Orbital setulae dense, in several irregular rows, reclinate to erect. Ocellar setulae erect. Eye broadly pilose dorsomedially. Third dorsocentral (variable from slightly longer than setulae to 2/3 length of second dc) sometimes present directly in front of second dorsocentral in male. Acrostichal setulae in eight irregular rows. Mid tibia with two posteromedial setae.

***Colouration***: Body, including halter, dark brown in base colour. Scutum shiny with faint greenish reflection. Calypter margin and hairs white. Abdomen (excluding tergite 1) sometimes coppery, with light blue shine usually evident posteriorly.

***Genitalia***: (Figs 281–284) Epandrium with small posterodistal spine. Hypandrium with apex strongly produced as apodeme. Metepiphallus with serrated ridges, one pair of longer ventral spines, and several overlapping lateral ridges. Proepiphallus V-shaped, narrowly divided medially and with apex upcurved. Basiphallus U-shaped with sides diverging and tips irregular in outline. Distiphallus separated from phallophorus by less than height of basiphallus; high, with spinulose internal structures, apex abruptly truncated, sides subparallel, and with short, thin bifid ventroapical plate; lateral surface of distiphallus with raised plate bearing minute scales and ridges; mesophallus projecting basally from distiphallus and far exceeding basal margin of distiphallus.

*Variation*: Male from Plummers Island differs as follows: two ori; frons with several longitudinal wrinkles on each side of ocellar triangle; wing length 3.5 mm; length of ultimate section of vein M4 divided by penultimate section 0.5; eye height divided by gena height: 4.8; abdomen with light blue shine; lateral margin of basiphallus less sculptured; lateral plate on dorsal lobe of distiphallus less pronounced, without medial scales. Several specimens from Turkey Run with wing length 3.0–3.2 mm (♂), 3.4 mm (♀), abdomen and notum distinctly green with apex of abdomen bluish, and phallus slightly smaller and paler.

###### Host.

Unknown.

###### Distribution.

**USA**: MD, SC, VA.

###### Etymology.

Gr.glyptos for carved, referring to sculptured outer lateral surface of distiphallus.

###### Type material.

***Holotype*: USA. NC**: Buncombe Co., 4 km SW Black Mtn., 21–27.1986, W.E. Steiner, Malaise trap, mixed deciduous and hemlock forest nr. small stream (1♂, USNM).

***Paratypes*: USA. MD**: Montgomery Co., Plummers Island, 38°58'N, 77°10'W, Malaise trap, lower trap, 24.iv-7.v.2006, D.R. Smith and J.W. Brown (1♂ 1♀, USNM; 1♀, CNC), Plummers Isl., 11.vii.1915, R.C. Shannon (1♂, USNM). **VA**: Shenandoah, Lewis Falls, 4.vii.1939, A.L. Melander (1♂, USNM), Fairfax Co., Turkey Run Park, nr. mouth of Turkey Run, 38°57.9'N, 7°09.4'W, Malaise trap, D.R. Smith, 29.iii-25.iv.2005 (1♂ 5♀, USNM; 1♂, CNC), 26.iv-2.v.2007 (12♂ 9♀, USNM), river, 14–17.v.2006 (2♀, USNM), Turkey Run Park, nr. headquarters bldg. 38°57.7'N, 77°08.9'N, Malaise trap, 29.iii-17.iv.2007, D.R. Smith (2♂ 1♀, USNM; 1♂, CNC), Great Falls Park, swamp trail, 38°59.4'N, 77°15.2'W, Malaise trap, 24.iv-2.v.2007, D.R. Smith (1♂, USNM; 1♀, CNC), Shenandoah, N Park Pinnacles, 19.vii.1952, W.W. Wirth (1♂, USNM).

###### Comments.

*Melanagromyzaglyptos* and *M.diantherae* are superficially similar, being large and stout-bodied with a blue metallic shine and more than five fronto-orbital setae. The two species are otherwise quite different in both outward and genitalic morphology, and can be easily diagnosed using the characters listed in the key.

##### Melanagromyza matricarioides

Taxon classificationAnimaliaDipteraAgromyzidae

Spencer

Figs 285–288

Melanagromyza matricarioides Spencer, 1969: 72. Plakidas 1982: 3.

###### Description.

Wing length 2.3 mm (♂). Female unknown (see *variation*). Length of ultimate section of vein M4 divided by penultimate section: 0.7. Eye height divided by gena height: 5.7. Gena highest behind midpoint of eye. Clypeus rounded, relatively thin and with arms slightly converging apically. Fronto-orbital plate and ocellar triangle subshiny. Fronto-orbital plate well-developed, relatively narrow, broadest behind midpoint of eye but not bulging, with swelling very shallow and indistinct; setae inset. Ocellar triangle narrow with anterior point open. Parafacial projecting dorsally.

***Chaetotaxy***: Two broadly separated ori; two ors. Orbital setulae in two irregular rows, becoming sparser posteriorly, erect to slightly proclinate. Ocellar setulae erect. Eye pilose in small spot dorsomedially. Two dorsocentral setae. Acrostichal setulae in eight irregular rows. Mid tibia with two posteromedial setae.

***Colouration***: Body, including halter, dark brown in base colour. Inner margin of fronto-orbital plate (more so anteriorly) and anterior corner of ocellar triangle sometimes light brown. Notum with faint greenish shine posteriorly (blue-green in holotype). Abdomen with green shine (bluer in holotype). Gena, notopleuron, postpronotum, and parafacial sometimes paler. Wing veins pale. Calypter margin and hairs white.

***Genitalia***: (Figs 285–288) Epandrium with small posterodistal spine. Surstylus narrow and downturned with discrete group of elongate, apical tubercle-like setae (not as figured in Spencer 1969). Hypandrium broad, short, and thick with long apical process. Metepiphallus with shallow spine and serrated ridge ventrally, and with narrow plate with several coalescing ridges laterally. Proepiphallus V-shaped and upcurved. Basiphallus U-shaped, arms relatively thick. Distiphallus separated from phallophorus by less than height of basiphallus; bases of distiphallus and mesophallus level; distiphallus with dorsomedial swelling, internal surface smooth or with remnants of spinulose internal structure (holotype), and with thick distoventral plate and dorsal processes comprising slightly narrowed apex.

***Variation***: Of the two non-type specimens mentioned in the original description, only the female could be located. This specimen differs as follows: wing length 2.6 mm; eye height divided by gena height 5.1; eye with smaller patch of setulae dorsally adjoining eye; fronto-orbital plate more abruptly bulging at midpoint to ~ ¼ width of frons, setulae in up to three irregular rows; abdomen with green shine. Possibly not conspecific, although most other possible local species to which it may belong in the *M.virens* group have the head longer towards the dorsum, and none except this species are known from *Anthemis*.

###### Hosts.

Asteraceae subfamily Asteroideae – *Matricariamatricariodes*, *Anthemis* sp., *A.cotula*, *Rudbeckialaciniata*[?].

###### Distribution.

**Canada**: ON. **USA**: MD, PA[?].

###### Type material.

***Holotype*: Canada. ON**: Ottawa, em. 4.vii.1955, ex. “stem mine in pineapple weed” [*Matricariamatricarioides*], leg. G. Lewis, reared J.F. McAlpine, CNC358428 (1♂, CNC; Type No. 10366).

###### Additional material examined.

**Canada. ON**: Ottawa, 25.vii.1962, J.R. Vockeroth, em. 26.vii.1962 from cultivated *Anthemis* (1♀, CNC). **USA. MD**: Ft. Detrich, 29.vi.1976, SWT Batra, AC-1 ex. *Anthemiscotula* L. (1♂, USNM).

###### Comments.

The Maryland male presents the second record of *Melanagromyzamatricarioides* in the United States following Plakidas (1982), who reared specimens from *Rudbeckialaciniata* in western Pennsylvania. Multiple adults from one or more Pennsylvania localities were recorded from 26 August to 3 October. The specimens were originally identified by G. Steyskal, and it is unknown why they were excluded from Spencer and Steyskal (1986b); only one specimen could be relocated, and examination has found this male to be *Melanagromyzavirginiensis* (see below).

This species belongs to the *Melanagromyzavirens* group, revealed by the widely spaced ori, and by the surstylus, which is incredibly similar to that of *M.virens* itself, although the long palisade-like setae are arranged in a much more discrete apical cluster. The distiphallus is slightly more compressed dorsoventrally (not teardrop-shaped) and has one pair of characteristic shallow carinae along the dorsolateral margin.

##### Melanagromyza minimoides

Taxon classificationAnimaliaDipteraAgromyzidae

Spencer

Figs 289–292

Melanagromyza minimoides Spencer, 1966b: 13. Spencer and Stegmaier 1973: 45; Spencer and Steyskal 1986b: 32; Scheffer et al. 2007: 770; Shi and Gaimari 2015: 51; Monteiro et al. 2019: 157; Eiseman et al. 2021: 10.
Melanagromyza radicicola Steyskal, 1981: 40. Spencer and Steyskal 1986b: 246. Syn. Spencer (1990).

###### Description.

Wing length 1.9–2.1 mm (♂), 2.1–2.3 mm (♀). Length of ultimate section of vein M4 divided by penultimate section: 0.6–0.7. Eye height divided by gena height: 5.0–6.3. Gena angulate, highest near midpoint of eye. Clypeus broadly rounded. Fronto-orbital plate and ocellar triangle mostly ill-defined; ocellar triangle rounded anteriorly, extending to region between ors, subshiny. Fronto-orbital plate slightly visible to not visible when head viewed laterally; well-developed but relatively narrow compared to most congeners, slightly subshiny, setae slightly inset.

***Chaetotaxy***: Two ori; two ors. Orbital setulae in two irregular rows, erect to reclinate. Eye bare or with few minute, sparsely arranged hairs. Ocellar and postocellar setae well-developed, but thin. Two dorsocentral setae. Acrostichal setulae in eight irregular rows. Mid tibia with two posteromedial setae.

***Colouration***: Body, including halter, dark brown in base colour. Gena slightly pale. Notum greenish, sometimes becoming bluish anteriorly. Calypter margin and hairs brown. Abdomen metallic green, sometimes coppery.

***Genitalia***: (Figs 289–292) Epandrium with small posterodistal spine. Surstylus small and rounded with tubercle-like setae clustered along inner and inner-marginal surfaces. Apex of hypandrium extremely elongate and tapered, subtriangular with base shallow and broadly arched; inner lobe attached to remainder of hypandrium by lightly sclerotised bridge. Metepiphallus broad and box-like with serrated ridges ventrally and irregular ridges laterally. Distiphallus separated from phallophorus by ~ 1.5 × height of basiphallus; mesophallus produced slightly past base of distiphallus; distiphallus small and ovate in ventral view with thin spinulose internal pads; distoventral plate vestigial, continuing as elongate, irregular duct that often has small, faint subapical sclerotised spot. Ejaculatory apodeme typical of *Melanagromyza*, blade relatively narrow and slightly pointed apically.

###### Hosts.

Asteraceae – *Borrichiafrutescens*, *Heleniumflexuosum*, *Helianthusannuus*, *Heliopsishelianthoides*, *Melantheranivea*, *Rudbeckialaciniata*, *Symphyotrichumsimmondsii*, *Verbesinaencelioides*, *V.virginica*. Cucurbitaceae – *Cucurbitafoetidissima*. Urticaceae – *Urticadioica*, U.gracilissubsp.holosericea (Eiseman et al. 2021).

###### Distribution.

**USA**: AR, CA, FL, IA, MD, MO*, MT*, NM*, OH, TX. Argentina. Bolivia. Brazil. Dominican Republic. Guadeloupe. Mexico. Venezuela. Uruguay.

###### Type material.

***Holotype* [*minimoides*]. USA. FL**: Hialeah, 10–14.ii.1964, ex. seeds of *Verbesina* sp., leg. 2.ii.1964, C.E. Stegmaier (1♂, USNM [on card with female paratype]).

***Paratype* [*minimoides*]. USA. FL**: Same data as holotype (1♀, USNM), Miami Dodge Is., 2.iii.1966, ex seeds *Borrichiafrutescens*, em. 4.iii.1966, K.A. Spencer, CNC358429 (1♂, CNC).

***Holotype* [*radicicola*]. USA. MD**: Bethseda, xii.1979, G.C. Steyskal, “emerged indoors from nettle st.” (1♂, USNM).

###### Additional material examined.

**Mexico.** Durango: 20.ix.1936, C.S. Rude (1♂, USNM). **USA. FL**: Hialeah, 15.xi.1964, C. Stegamier, ex. *Verbesina* (1♂ 1♀, USNM), Key Largo, beach on NE, 13.xii.1985, A.L. Norrbom, reared ex. flower of *Borrichiafrutescens* (L.) DC (14♂ 14♀ [seven with puparia], USNM), Key Largo, Pennekamp State Park, 13.xii.1985, A.L. Norrbom, reared ex. flower head of *Borrichiafrutescens* (L.) (1♂, USNM), Big Pine Key, 13.xii.1985, ex. flower head of *Borrichiafrutescens* (L.) (1♂ 2♀, USNM), Dade Co., Naranja, 12.xii.1985, A.L. Norrbom, reared ex flower head of *Bidensalba* v. *radiata* (1♂, USNM), **IA**: Winneshiek Co., Decorah, Will Baker Park, 43°18'9.12"N, 91°48'42.15"W, 10–13.viii.2017, J. van der Linden, *Rudbeckialaciniata*, em. by 16.viii.2017, #CSE4945, CNC1643663–1643667 (2♂ 3♀, CNC), **MD**: Montgomery Co., Bethseda, G. Steyskal, 31.viii.1970 (1♀, USNM), 30.viii.1970 (1♂, USNM), reared ex. flower heads of *Heliopsishelianthoides*, 21.viii.1970 (2♂ 7♀ 8puparia, USNM), 24.viii.1970 (4♂ 3♀, USNM), 26.viii.1970 (1♂, USNM), 27.viii.1970 (3♂ 1♀, USNM), 28.viii.1970 (2♀, USNM), 30.viii.1970 (1♂ 3♀, USNM), 31.viii.1970 (1♀, USNM), 2.ix.1970 (3♀, USNM), 9.ix.1970 (1♂ 3♀, USNM), 26.ix.1970 (2♀, USNM) reared ex. flower heads of *Rudbeckialacinata*, 13.viii.1970 (1♀, USNM), 24.viii.1970 (1♀, USNM), 28.ix.1970 (1♂, USNM), 1.ix.1970 (3♂, USNM), 2.ix.1970 (1♀, USNM), 6.ix.1970 (2♂, USNM), 8.ix.1970 (1♂ 1♀, USNM), 9.ix.1970 (2♂ 1♀, USNM), 10.ix.1970 (3♂ 1♀, USNM), 11.ix.1970 (1♂ 1♀, USNM), 14.ix.1970 (1♂ 3♀, USNM), 16.ix.1970 (2♂ 1♀, USNM), 17.ix.1970 (2♂ 7♀, USNM), 20.ix.1970 (1♀, USNM), viii-ix.1971 (1♀ 7puparia, USNM), 13.viii.1974 (puparia, USNM), 23.viii.1974 (1♀, USNM), 31.viii.1971 (2♀, USNM), 2.ix.1971 (6♀, USNM), 4.ix.1971 (1♀, USNM), 3.ix.1972 (1♀, USNM), 11.ix.1972 (1♀[with puparium], USNM), 1.ix.1974 (7♀[with puparia], USNM), 12.ix.1974 (12♀, USNM), **MO**: St. Louis, 6.28, reared ex *Helianthuspitcheriana*, Webster Grve, No. 33125, Satterthwaite (5♂ 9♀[gel capsule], USNM), **MT**: St. Louis Webster Grvs, No. 22195, “7.30”, reared from *Heliopsis*, Satterthwaite Collector (2♂ 3♀, USNM), **NM**: Roosevelt Co., Portales, 4000ft, Malaise trap, 23–26.viii.1993, O’Hara and Jorgensen, debu00128110 (1♂, DEBU), **TX**: Hidalgo Co., Weslaco, ii.1975, C.E. Rogers, larva in *Helianthusannuus* (3♂ 2♀, USNM).

##### Melanagromyza rutella sp. nov.

Taxon classificationAnimaliaDipteraAgromyzidae

http://zoobank.org/65749D5B-1014-46C9-91C1-406D56270661

Figs 293–296

###### Description.

Wing length 2.5–2.7 mm (♂), 2.4–2.8 mm (♀). Length of ultimate section of vein M4 divided by penultimate section: 0.6–0.7. Eye height divided by gena height: 2.1–5.9. Clypeus broadly rounded. Ocellar triangle and fronto-orbital plate subshiny. First flagellomere narrow, not broadly rounded below. Fronto-orbital plate and parafacial projecting with portion visible laterally 1/2 height of first flagellomere; fronto-orbital plate well-developed, setae inset. Ocellar triangle subshiny to matt, sometimes appearing to end at posterior ori, but if anterior margin ill-defined, then appearing to continue to lunule. Gena rounded, highest behind midpoint of eye.

***Chaetotaxy***: Three ori (four on one side of IN male); two ors. Orbital setulae short, in up to four irregular rows, erect to reclinate. Eye pilose dorsomedially. Two dorsocentral setae. Acrostichal setulae in eight to ten irregular rows. Mid tibia with one or (more commonly) two posteromedial setae.

***Colouration***: Body, including halter, dark brown in base colour. Notum greenish (faded anteriorly); femora sometimes also slightly reflective. Calypter margin and hairs white. Abdomen with greenish shine, sometimes becoming slightly coppery/reddish.

***Genitalia***: (Figs 293–296) Posterodorsal margin of epandrium slightly produced; posteroventral spine well-developed. Surstylus 1/2 height of epandrium, with up to three rows of tubercle-like setae along inner distal margin. Basiphallus short, U-shaped, and extending slightly past base of distiphallus. Base of mesophallus extending past that of distiphallus; distiphallus with paired ventrolateral tubules shallowly raised from base of distiphallus, or prominent, separate and strongly looped (as illustrated); with dorsoapical margin strongly flared laterally; venter with strong divided carina. Ejaculatory apodeme well-developed with base dark, becoming paler distally on stem; blade large, spade/diamond-shaped, weakly sclerotised but with defined medial rib and strong marginal band (less so laterally) well-defined.

###### Host.

Unknown.

###### Distribution.

**USA**: IN, VA.

###### Etymology.

L. *rutrum* for spade, shovel, referring to the shape of the large ejaculatory apodeme.

###### Type material.

***Holotype*: USA. VA**: Fairfax Co., Great Falls Park, quarry, 38°59.1'N, 77°14.8'W, Malaise trap, D.R. Smith, 24.iv-2.v.2007 (1♂, USNM).

***Paratypes*: USA. IN**: La Fayette, 11.v.1916, J.M. Aldrich (1♂ 2♀, USNM), **VA**: Same collection as holotype (1♂ 2♀, USNM; 2♀, CNC), 3–10.v.2007 (3♀, USNM).

###### Comments.

*Melanagromyzarutella* is dark (i.e., without a slightly paler gena, shoulders, etc.) with a white calypter and three ori on both sides of the frons. The gena is also often relatively high, the blade of the ejaculatory apodeme is nearly diamond-shaped, the distiphallus is stout and strongly flared apically, the ventrolateral tubules of the phallus are distinct and sometimes broadly looped, and the distiphallus has a single internal rectangular pad with numerous transverse rows of small spines.

There is a resemblance to *Melanagromyzaurticae* Eiseman and Lonsdale (Eiseman et al. 2021: figs 16–19, 111–116), especially with regards to the male genitalia, but this other Iowa species has two ori, three ors, the surstylus is not produced slightly posteriorly, the mesophallus is inserted almost basally on the distiphallus (the ventrolateral tubules are more pronounced in *M.rutella*), the dorsal chamber of the distiphallus is stouter and almost parallel-sided in ventral view (not constricted medially) and higher in lateral view, and the tubule emerging from the dorsal chamber apically is longer.

##### Melanagromyza splendida

Taxon classificationAnimaliaDipteraAgromyzidae

Frick

Figs 297–300

Melanagromyza splendida Frick, 1953b: 207. Spencer 1981: 59; Spencer and Steyskal 1986b: 25; Shi and Gaimari 2015: 72.

###### Description.

Wing length 1.8–2.6 mm (♂), 1.9–2.6 mm (♀). Length of ultimate section of vein M4 divided by penultimate section: 0.5–0.7. Eye height divided by gena height: 3.9–6.9. Deepest portion of gena 1/3–1/2 distance from anterior margin. Fronto-orbital plate not projecting, sometimes slightly visible in lateral view; strongly swollen in males, 25–28% frons width, narrowing anteriorly; narrower in females, 16–23% frons width; setae inset along inner margin of plate. Clypeus broadly rounded. Ocellar triangle slightly longer than wide.

***Chaetotaxy***: Two ori with anterior seta strongly displaced anteriorly; two ors. Eye extensively pilose, hairs sparse excluding dense dorsal patch. Orbital setulae erect to reclinate, slightly proclinate anteriorly (mostly inner row), in up to three or four irregular rows; setulae longer in male. Two dorsocentral setae. Acrostichal setulae in eight irregular rows. Mid tibia with two posteromedial setae.

***Colouration***: Body, including halter, dark brown in base colour. Fronto-orbital plate sometimes whitish along anteromedial margin. Notal colour ranging from brilliant green to dull green with coppery tint. Calypter margin and hairs white. Abdomen metallic green, sometimes bronze (Austin, TX).

***Genitalia***: (Figs 297–300) Epandrium with posterodistal spine. Surstylus < 1/2 height of epandrium, distal margin slightly rounded and angled, with several irregular rows of tubercle-like setae along inner-distal surface. Hypandrium subtriangular, long with thin, tapered distal process. Proepiphallus dark and V-shaped. Metepiphallus broad and dark with several sublateral tubercles and lateral ridges. Basiphallus U-shaped. Space between distiphallus and phallophorus less than height of basiphallus. Distiphallus relatively globular with distinct transverse fold laterally on distal 1/3 that curves towards venter; base exceeding that of mesophallus; inner surface only with clusters of shallow bumps (no spines); with short narrow tubule emerging apically below short plate; seen ventrally, width of distiphallus varying from relatively slender (as in illustration) to stouter around middle.

***Variation***: One AZ male (dissected) with fronto-orbital plate swollen to 1/3 width of frons (ie. similar to *M.virens*). Dissected Newark (DE) male, possibly a separate species, differs as follows: dark distoventral process on distiphallus highly reduced, distiphallus slightly more quadrate in outline (seen ventrally), and surstylus not downturned apically, but with long tubercule-like setae typical of *M.matricariodes* and *M.virens*; clypeus distinctly truncated apically and orbit slightly less swollen medially.

###### Hosts.

Asteraceae – *Ambrosia*, *Bidens*, Centaurea*, Cineraria*, Coreopsis*, *Erechtites*, *Flaveria*, *Gaillardia*, Galinsoga*, *Gnaphalium*, *Helianthus*, Hymenoxis*, *Lactuca*, *Parthenium*, *Tagetes*, *Zinnia*. Cucurbitaceae – *Cucurbita*. Apiaceae – *Apium*, *Daucus*. NC record of feeding on potato requires verification.

###### Distribution.

**USA**: AZ, CA, DE*, FL, HI, IL, IN*, MD*, MI[?], MO*, NC*, NY[?], SC[?], TX*. Argentina*. Bahamas. Chile. Jamaica. Mexico.

###### Type material.

***Holotype*: USA. HI**: Kamuela, 5.xii.1950, ex. celery (1♂, USNM).

***Paratype*: USA. HI**: same data as holotype (1♀, USNM).

###### Additional material examined.

**Argentina.** Salta: Rt. 34, 4 km S Metan, i.1983, *Galinsogaparviflora*, tunneling inside stems (1♂ 1♀, USNM), Jujuy: Rt. 9, 1 km S Maimara, 31.xii.1982, *Hymenoxisrobusta*, tunneling inside stem (1♀, USNM). **Bahamas.** Bimini Isl., 22–31.i.1968, light trap, G.M. Stokes (2♂, USNM). **Chile.** Valdivia: Santiago, 4.v.1978, 78.3999, reared ex. sunflower stalk (1♂, USNM). **USA. AZ**: Yuma, 20.v.1968, D.M. Tuttle (2♂ 2♀, USNM), **CA**: Ortega Hiwy., near summit, 11.vi.1944, A.L. Melander (1♂, USNM), San Simeon, 31.viii.1945, A.L. Melander (1♀, USNM), Orange Co., Fullerton, 13.ii.1969, R.D. Goeden and D.W. Ricker, insectary reared on *Ambrosiapsilostachya* Decandolle (2♂, USNM), Santa Barbara Co., Santa Barbara, 22.iv.1969, R.D. Goeden and D.W. Ricker, insectary reared on *Ambrosiapsilostachya* (2♂ [with puparia], USNM), San Bernardino Co., Highland, 2.v.1969, R.D. Goeden and D.W. Ricker, insectary reared on *Ambrosiaacanthicarpa* Hook. (1♀ [with puparium], USNM), Cucamonga, 20.v.1969, R.D. Goeden and D.W. Ricker, insectary reared on *Ambrosiaacanthicarpa* Hook. (1♀ [with puparium], USNM), Salinas, 1.xi.1930, Bred from [illegible]ule (1♂, USNM), L.A. Co., Westwood Hills, 3.xi.1938, ex Cineraria stems acc. 94 (1♂, USNM), **DE**: Lum’s Pond, 17.vii.1974, D. Buntin (2♂, USNM), Newark, D. Buntin, 19.viii.1974 (2♂, USNM), 10.vii.1975 (1♂, USNM), 9.vi.1975 (1♂, USNM), **FL**: Dade Co., Naranja, 12.xii.1985, A.L. Norrbom (1♂, USNM), Highlands Co., Archibold Biological Sta., 24.iii.1969, S.W. Frost, CNC358443 (1♂, CNC), Highland Co., Archibold Biol. Sta., 24.iv.1967, B.V. Peterson, CNC358464 (1♂, CNC), **HI**: Honolulu, 6.v.1929, ex Cornflower (Centaurea), J.F. Illingworth (2♂, USNM), 7.v.1929 (1♂ 1♀, USNM), 8.v.1929 (1♀, USNM), **IL**: Champaign, tomato leaves, 12.ix.1957, J.F. McAlpine, CNC358451 (1♂, CNC), Alhambra, 24.vi.1937, reared from sunflower, destroying stem, Satterthwaite (4♂ 10♀, USNM), **IN**: Lafayette, J.M. Aldrich, “vii-21” (1♂, USNM), “x-14” (1♂, USNM), 4.ix.1916 (1♂, USNM), “vii-26” (1♂, USNM), “6-27” (1♂, USNM), “v-23” (1♂, USNM), “v-27” (2♂, USNM), [no date] (1♂, USNM), **MD**: College Park, 2.viii.1931, C.T. Greene (1♂, USNM), **NC**: Chadboura, 27.v.1910, feeding on potato, E.G. Smyth (1♂, USNM), **MO**: Webster Groves, 17.v.1932, Helianthus tuberosa, Satterthwaite (1♀, USNM), [locality not provided, likely Webster Groves], 32165b, Helianthus tuberosa (1♂, USNM), **TX**: Galveston, vi.1900, A.L. Melander (1♂, USNM), Mexico, in carrot, Laredo, Tx., 31255, 18.i.1943, Lot No. 43-1075 (1♂, USNM), 31457, 13.ii.1943, Lot No. 43-1590 (1♂ 1♀, USNM), Mexico, in carrot root, El Paso, 33818, Lot No. 42-6728 (1♀, USNM).

###### Comments.

The short, squat distiphallus is highly characteristic of this species, especially the bulging laterodistal region that appears as a transverse fold. Male dissection is recommended for confident identification.

While *Melanagromyzasplendida* has previously been recorded as far north as New York and Michigan in the United States, almost all of the northern males identified by previous authors dissected here have been *M.virginiensis*. The remaining specimens for which previous Michigan, New York, and South Carolina records of *M.splendida* were based (Spencer and Steyskal 1986b) have not been found, and it is suspected that these might also be misidentifications of *M.virginiensis*.

Several Texas records, which may represent interceptions from Mexico, note carrot as host. When considering the economic significance of carrots, the widespread occurrence of the fly, and the absence of previous rearing records, it is possible that the labels are in error or *Daucus* is not a preferred host.

##### Melanagromyza subvirens

Taxon classificationAnimaliaDipteraAgromyzidae

(Malloch)

Figs 301–304

Agromyza subvirens Malloch, 1915a: 105.
Melanagromyza subvirens . Frick, 1959: 366; Spencer and Steyskal 1986b: 26.

###### Description.

Wing length 2.8–3.2 mm (♂), 3.4–3.5 mm (♀). Length of ultimate section of vein M4 divided by penultimate section: 0.5–0.6. Eye height divided by gena height: 5.0–5.5. Gena highest at ~ anterior 1/3, angled upwards anteriorly, with distinct cheek. Fronto-orbital plate well-developed, slightly visible laterally, setae slightly inset. Clypeus broadly rounded. Fronto-orbital plate and ocellar triangle slightly less matt than surrounding frons. Ocellar triangle reaching second ori from rear.

***Chaetotaxy***: Five to six fronto-orbitals with posterior one or two reclinate (ors). Ocellar setulae short, reclinate, in two irregular rows. Eye sparsely pilose dorsomedially, sometimes slightly denser in male. Anterior genal seta sometimes vibrissa-like. Two dorsocentral setae; MD male with third dorsocentral on left side. Acrostichal setulae in eight irregular rows. Mid tibia with two posteromedial setae.

***Colouration***: Body, including halter, dark brown in base colour; female darker, blackish. Scutellum with faint greenish shine in male, female sometimes with bluish tint. Calypter margin and hairs white. Abdomen with green or bluish green shine, and females sometimes with strong blue shine only. Slightly reddish, at least on antenna and gena, and sometimes more widespread.

***Genitalia***: (Figs 301–304) Epandrium with small posterodistal spine. Surstylus ~ 1/2 length of epandrium venter (epandrium dorsum shallower); inner surface with several irregular rows of tubercle-like setae. Hypandrium subtriangular with slightly produced apex. Metepiphallus with serrated ventral and lateral ridges. Proepiphallus V-shaped. Basiphallus forming complete ring with ventrodistal margin ill-defined. Distiphallus separated from phallophorus by height of basiphallus; mesophallus slightly exceeding base of distiphallus; distiphallus relatively flat, with internal spinulose pads, strongly upturned and apically widened dorsoapical membranous process; ventrolateral tubules short. Ejaculatory apodeme well-developed with short stem and narrow rib slightly exceeding apex of broad, pale, ovate blade.

###### Host.

Unknown.

###### Distribution.

**USA**: IA, IL, MD*, NC*, PA, VA.

###### Type material.

***Holotype*: USA. IL**: St. Joseph, 17.v.1914, C.A. Hart and J.R. Malloch (1 ♀, INHS). [Not examined].

***Paratype*: USA. IL**: St. Joseph, 17.v.1914, Salt Fork (1♀, USNM).

###### Material examined.

**USA. IL**: Oregon, 21.vi.1917, en copulae; CNC358452 (1♂ 1♀, CNC), **MD**: Laurel, 1.vi.1965, Malaise trap, CNC358454, CNC358455, CNC358456 (3♀, CNC), nr. Plummers Isl., 2.v.1915, R.C. Shannon (1♀, USNM), Montgomery Co., Bethseda, 26.v.1968, G. Steyskal (1♂, USNM), **NC**: Wilson’s Gap, 944 m, 25.v.1957, J.R. Vockeroth, CNC358453 (1♂, CNC).

##### Melanagromyza vernoniae

Taxon classificationAnimaliaDipteraAgromyzidae

Steyskal

Figs 305–308

Melanagromyza vernoniae Steyskal, 1981: 41. Spencer and Steyskal 1986b: 23.

###### Description.

Wing length 3.1–3.5 mm (♂), 2.8–3.4 mm (♀). Length of ultimate section of vein M4 divided by penultimate section: 0.6–0.7. Eye height divided by gena height: 4.6–5.5. Clypeus broadly rounded. Male eye with short, scattered setulae covering dorsomedial region of eye (setulose region relatively narrow compared to M. *vernoniana*) that may sometimes be relatively dense; female eye virtually bare. Fronto-orbital plate slightly widened at middle, only slightly more than one quarter width of frons at widest point; subshiny; bulging anteriorly and slightly visible laterally, sometimes continuing as ring below eye. Ocellar triangle relatively narrow and parallel-sided, reaching level of posterior ori.

***Chaetotaxy***: Two ori with anterior seta strongly displaced anteriorly (one IN male with additional seta between standard ori that is weak on left side and well-developed on right); two ors with anterior seta slightly inclinate. Orbital setulae with up to three irregular, scattered rows, generally erect, slightly reclinate anteriorly and proclinate posteriorly and along outer row; relatively long and bushy, considerably longer than ommatrichia (more so in males). Two dorsocentral setae. Acrostichal setulae in eight irregular rows. Katepisternum with one to several short setae clustered dorsomedially. Mid tibia with two posteromedial setae.

***Colouration***: Body, including halter, dark brown in base colour; pedicel and scape sometimes slightly paler brown. Notum with slight bluish shine that may be slightly greenish, especially on posterior third of scutum and on scutellum. Calypter margin and hairs white. Abdomen with strong metallic green shine. MN male with abdomen blue. IN specimens with notum greenish (not blue) and abdomen greenish coppery in males and green to blue in females.

***Genitalia***: (Figs 305–308) Surstylus (entirely fused to epandrium) with relatively long, flat distal margin ~ 2/3 length of distal section of epandrium (dorsum of epandrium shallower) that is slightly produced at anterior corner; inner surface with two (posteriorly) to four (anteriorly) rows of short, thickened setae, of which a few at slightly produced corner of surstylus are elongate, although state not as exaggerated as seen in *M. virens*. Hypandrium extremely long and produced apically and with longitudinal basal ridge; inner lobe irregular in outline and weakly sclerotised. Metepiphallus with small spines medially and with coalescing ridges laterally. Proepiphallus V-shaped. Basiphallus U-shaped with corners at right angles and arms parallel. Mesophallus and distiphallus level at base. Distiphallus with basal section ~ 2/3 length of segment, relatively narrow, not bulging dorsally, with well-developed internal spinulose pads; ventrolateral tubules relatively long, narrow, recessed.

###### Host.

Asteraceae – *Vernonianoveboracensis*.

###### Distribution.

**USA**: DC, IN*, MN*.

###### Type material.

***Holotype* [*vernoniae*]: USA. DC**: 18.x.1969, D. Anderson, *Vernonianoveboracensis*, emg. indoors after refrigeration 19.xii.1969 (1♂, USNM).

***Paratypes* [*vernoniae*]: USA. DC**: Washington, E Bank CandO Canal nr. Ariz. Ave., reared from larva in stem *Vernonianoveboracensis*, D.M. Anderson, 23.i.1970 (1♂, USNM), 28.iii.1970 (2♂, USNM).

###### Additional material examined.

**USA. DC**: Washington, E Bank CandO Canal nr. Ariz. Ave., 14.x.1969, D.M. Anderson, Reared from larvae in stem *Vernonianoveboracensis*, 23.ii.1970 (3♀, USNM), Washington, E Bank CandO Canal nr. Ariz. Ave., 28.iii.1970, D.M. Anderson, Reared from larvae in stem *Vernonianoveboracensis* (1♀, USNM), **IN**: LaFayette, J.M. Aldrich, v-23 (1♂ 1♀, USNM), v-27 (6♂ 3♀, USNM), v-28 (1♂, USNM), iv-27 (3♂, USNM), vi-10 (1♂, USNM), vi-22 (1♀, USNM), vi-23 (1♂, USNM), vii-8 (1♀, USNM), 6-27 (1♂, USNM), **MN**: New Ulm, 30.v.1916, J.M. Aldrich (1♂, USNM).

##### Melanagromyza vernoniana

Taxon classificationAnimaliaDipteraAgromyzidae

Steyskal

Figs 309–317

Melanagromyza vernoniana Steyskal, 1981: 41. Spencer and Steyskal 1986b: 23.
Melanagromyza verbesinae Spencer in Spencer and Steyskal 1986b: 248. Syn. nov.

###### Description.

Wing length 2.3–2.8 mm. Length of ultimate section of vein M4 divided by penultimate section: 0.6–0.8. Eye height divided by gena height: 4.1–5.0. Clypeus broadly rounded. Pilosity on male eye dense and relatively broad dorsally, covering most of dorsal third of eye. Fronto-orbital plate slightly widened at middle, not exceeding 1/5 width of frons; subshiny; reaching level of posterior ori.

***Chaetotaxy***: Two ori with anterior pair of setae widely separated from second pair; two ors. Orbital setulae in up to three irregular rows, reclinate to slightly lateroclinate with inner row partially proclinate; as long as eye setulae, and slightly darker and much less densely arranged compared to eye setulae. Two dorsocentral setae. Acrostichal setulae in eight irregular rows. Mid tibia with two posteromedial setae.

***Colouration***: Body, including halter, dark brown in base colour; pedicel and portions of frons sometimes slightly paler. Notum with faint greenish to bluish shine; indistinct in poorly preserved specimens. Calypter margin and hairs white. Abdomen with strong metallic green shine, sometimes bronze.

***Genitalia***: (Figs 309–313) Epandrium with small posterodistal spine. Surstylus one 1/2 length of epandrium or slightly less; slightly produced, elongate; inner surface with several irregular rows of tubercle-like setae, posterodistal corner with several of these longer, posteriorly directed (not as pronounced as state seen in *M.virens* and *M.matricariodes*). Metepiphallus with serrated ventral ridges and ventrally coalescing lateral ridges. Proepiphallus very dark and V-shaped. Phallophorus short. Basiphallus U-shaped with minute irregularities in lateral margin. Distiphallus separated from phallophorus by slightly more than height of basiphallus; base of mesophallus produced slightly past base of distiphallus; distiphallus with broad, short base and elongate distal section that is slightly upcurved when seen laterally and elongate oval when seen ventrally; distiphallus sometimes with one pair of short subtriangular plates emerging anterodorsally from swollen basal section.

***Variation***: See comments section.

###### Host.

Asteraceae – Helianthusannuus*, H.tuberosus*, Heleniumautumnale*, *Verbesinaalternifolia*, *Vernonianoveboracensis*.

###### Distribution.

**Canada**: ON*. **USA**: DC, IL*, IN*, MD, MO*, OH, OK*, TN, TX, VA*, VT*, WI*.

###### Type material.

***Holotype* [*vernoniana*]: USA. MD**: Cropley, 20.x.1968, D.M. Anderson, emg. Ex. stem *Vernonianoveboracensis* (1♂, USNM).

***Paratype* [*vernoniana*]: USA. DC**: Washington, E bank CandO Canal nr. Ariz. Ave., 28.iv.1970, D.M. Anderson, reared from larva in stem *Vernonianoveboracensis* (1♂, USNM).

***Holotype* [*verbesinae*]: USA. OH**: Jennings Woods, 6.0mi NE Ravenna, 21.iii.1970, ex. stem *Verbesinaalternifolia* (1♂, USNM).

###### Paratypes examined

[***verbesinae*]: USA. TN**: East Ridge, 6.v.1952, G.S. Walley, CNC358449 (1♂, CNC), **TX**: Kerrville, swept ex meadow, 18.iv.1959, J.F. McAlpine, CNC358448 (1♂, CNC).

###### Additional material examined.

**Canada. ON**: Wellington Co., nr. Arkell, 43°33'18"N, 80°10'16"W, trail beside river, 24.vi.2010, O. Lonsdale, CNC358506 (1♂, CNC). **USA. IL**: Urbana, in Nat. Res. Mus., 5.v.1957, J.F. McAlpine, CNC358450 (1♂, CNC), Hinsdale, 25.x.1929, H[elianthus] tuberosus, Webster Grve, Satterthwaite, No. 29308818 (1♂, USNM), No. 29308817 (1♂, USNM), Hinsdale, [illegible], 25.x.1929, Webster Grvs, No. 29310a, [illegible], 8.i.1930, Satterthwaite (1♀, USNM), Chicago, 6.vi.1903, A.L. Melander (1♂, USNM), **IN**: Lafayette, 27.x.1915, reared from sunflower pith, Cage No. C1490, Satterthwaite (1♂, USNM), **MD**: Montgomery Co., Bethseda, 23.v.1970, G. Steyskal (1♂, USNM), **MO**: Black Jack, 28.x.1930, Webster Grove, Helianthus tuberosa, Satterthwaite, [illegible], No. 40319g, 13.iv.1931 (1♀, USNM), No. 30419a, 20.iv.1931 (1♂, USNM), Web Groves Sta., dead at cold room window, 14.iv.1933 (3♂ 4♀[gel capsule], USNM), 20.iv.1933 (2♀[with 2 Braconidae in gel capsule], USNM), 21.iv.1933 (1♀[with Braconidae in gel capsule], USNM), **OK**: Murray Co., Sulphur, Chickasaw Rec. Area, 4.vi.1979, S.andJ. Peck, prairie vegetation (1♂, DEBU), **MO**: Cross Keys, 1.xii.1932, dead 3.ii.1933, Webster Grv, No. 32S11, R.B. Swain (1[unemerged from puparium], USNM), Webster Grvs, No. 32052C, Helianthusannuus, laboratory garden, [date illegible], dead 11.v.1932, Satterthwaite (1♂, USNM), Webster Groves, Helianthusannuus, 23.iii.1930, No. 30012, issued 1.iv.1930, R.C. Lange (1♂, USNM), 16.iii.1931, No. 31025C, issued 14.iv.1931 (1♂, USNM), Webster Groves, sunflower, iss. [?].iii.1933, No. 33034R, Satterthwaite (1♂, USNM), iss. 4.iv.1933, 26.iii.1933 (1♂ 2♀, USNM), 26.iii.1933, No. 33039f, iss. 3.iv.1933 (1♂ 2♀, USNM), iss. 5.iv.1933, No. No. 33039f, 26.iii.1933 (1♂, USNM), iss. 30.iii.1933, No. 330392, 26.iii.1933 (1♂, USNM), Webster Groves, 26.vii.1929, reared from sunflower, issued 3.viii.1929, dead 3.viii.1929, No. 29162, Satterthwaite (1♂, USNM), Webster Groves, sunflower, issued 12.viii.1929, dead 8.13, No. 29161, 26.vii.1929, Satterthwaite (1♂, USNM), Webster Groves, 9.vi.1929, sunflower, Satterthwaite (1♀, USNM), 21.viii.1928 (1♀, USNM), Maplewood, 24.vii.1930, issued 22.viii.1930, Webster Grvs, No. 30246b, Satterthwaite (1♂, USNM), issued 25.viii.1930, No. 30246a (1♀, USNM), East St. Louis, [?], 9.ix.1929, sunflower, Webster Grvs, No. 2925290, issued ix.1930, J.B. Sahan (1♀, USNM), Cross Keys, Artichoke, Webster Grvs, R.B. Swain, 1.xii.1932, iss. 14.iii.1933, No. 32497p (1♂, USNM), 1.xii.1932, iss. 4.iii.1933, No. 32497t (1♂, USNM), 1.xii.1932, iss. 1.iv.1933, No. 32497aa (1♂, USNM), 1.xii.1932, iss. 29.iii.1933, No. 32497d (3♂ 1♀, USNM), 1.xii.1932, iss. 1.iv.1933, No. 32497ab (1♂, USNM), 1.xii.1932, iss. 5.iv.1933, No. 324970 (1♀, USNM), 1.xii.1932, iss. ?3.iv.1933, No. 32497h (1♀, USNM), 1.xii.1932, iss. 2.20, No. 33003 (1♀, USNM), Florrisant, Artichoke, Webster Grvs, 2.xii.1932, iss. 12.iv.1933, No. 32495 (1♀, USNM), Black Jack, Artichoke, Webster Grvs, R.B. Swain, 18.x.1932, iss. [illegible], No. 32476 (1♂, USNM), 1.xii.1932, iss. 31.iii.1933, No. 32497f (1♂, USNM), 1.xii.1932, iss. 13.iv.1933, No. 32446 (1♀, 6♂ 2♀[gel capsule], USNM), **VA**: Batesville, M.L. Bobb, 6.vi.1940 (1♂, VPIC), 26.iv.1939 (1♂, VPIC), 18.v.1940 (1♀, VPIC), 30.iv.1940 (1♂, VPIC), Fairfax Co., Great Falls Park, quarry, 38°59.1'N, 77°14.8'W, Malaise trap, 3–10.v.2007, D.R. Smith, CNC358447 (1♂, CNC), Charlottesv., 30.xi.1930, Webster Grvs, No. 30480s, Helianthus tuberosa, [illegible], 14.iv.1931, Dr. Phillips (1♀, USNM), 20.xi.1930, No. 30480h (1♀, USNM), **VT**: Plainfield, 24.vi.1980, B.V. Peterson, CNC358504 (1♂, CNC), **WI**: Grant Co., Thomas Wet. Prairie, 30.ix.1997, A.H. Williams, *Heleniumautumnale*, [host plant] stripped of leaves and inflorescences, put into sterile containers over sterile soil and netted with hosiery, outdoors until 2.iii.1998 when caged in lab, em. 4–15.iv.1998, CNC934525–934529 (3♂ 2♀, WIRC).

###### Comments.

While never directly compared in the literature, male terminalia and external morphology reveal strong similarities between *Melanagromyzavernoniana* and *M.verbesinae*, especially the phallus, which includes a distiphallus that is long and curved with the basal section abruptly swollen and globose with suspended ventrolateral tubules.

The examined *Melanagromyzaverbesinae* paratypes have a faint greenish tint on the notum and a relatively broad, truncated surstylus, and were apparently used for the underlying original concept of the species. The wing length is also 2.4–2.8 mm, not 2.1 mm as noted in original description. Hosts are unknown for paratypes. Examination of the genitalia has also revealed that the basal section of the distiphallus is produced into one pair of weakly sclerotised, subtriangular dorsal plates. These genitalic features are also seen in the non-type material reared from “artichoke” (Figs 314–317) and some specimens reared from *Helianthusannuus*. The *M.vernoniana* types (ex *Vernonia*) and the *M.verbesinae* holotype (ex *Verbesina*) have a faint bluish tint on the notum, a slightly shallower epandrium, a narrower, rounded surstylus, and no dorsal plates arising from the basal globose section of the distiphallus; wing length is 2.3–2.8 mm.

Aside from these type specimens, additional material has been examined exhibiting overlap in genitalic morphology between the two extremes outlined above. Some males have well-developed to reduced dorsal plates on the distiphallus and a narrower surstylus; specimens without these dorsal plates on the distiphallus sometimes have a stouter truncated surstylus, such as those reared from *Helianthustuberosus* and one male from *H. annuus*. Colouration is also not as distinct as previously considered. The green or blue shine is faint and difficult to discern, and while some specimens are clearly greener or bluer compared to others, intermediate colouration is sometimes evident. The difference in colouration, however, is much less than that seen in other *Melanagromyza* species (although usually this is manifest on the abdomen, not the notum) and may be insignificant. The continuum of states seen in the genitalia, the similarities in colour and body length (which are not as disparate as previously considered), and the strong similarities in other external morphological features support the synonymy of these two species; this is compounded by the especially strong similarities between the *M.verbesinae* holotype and the type material of *M.vernoniana*. Rearing records of both extreme genitalic types from *Helianthus* further support synonymy.

Some of the newly recorded specimens examined here were reared from “artichoke” at Webster Groves. This may refer to *Cynarascolymus*, the “globe artichoke”, but it is more likely that it refers to *Helianthustuberosus*, otherwise known as “Jerusalem artichoke”, also studied at Webster Groves. The *Helenium*-reared specimens were first reported as *Melanagromyza* sp. in Williams (1999).

##### Melanagromyza virens

Taxon classificationAnimaliaDipteraAgromyzidae

(Loew)

Figs 4 , 56 , 57 , 62 , 63 , 318–324

Agromyza virens Loew, 1869: 46.
Melanagromyza virens . Frick, 1952a: 380, 1957: 200 [lectotype designation], 1959: 367; Spencer 1969: 76; Spencer and Stegmaier 1973: 51; Spencer and Steyskal 1986b: 25; Scheffer et al. 2007: 770; Shi and Gaimari 2015: 85.
Melanagromyza heterothecae Spencer, 1966b: 10. Syn Spencer (1969).

###### Description

**(Figs 4, 56, 57, 62, 63, 324).** Wing length 1.8–2.3 mm (♂), 2.4–2.6 mm (♀); female lectotype and paralectotype 2.9 mm and 3.2 mm, respectively. Length of ultimate section of vein M4 divided by penultimate section: 0.6. Eye height divided by gena height: 4.6–6.5. Gena usually deepest at 1/3 distance from anterior margin, or slightly behind. Clypeus broadly rounded. Ocellar triangle narrow, reaching level of posterior ori, sides concave. Fronto-orbital plate widest medially, broadest behind middle; slightly visible laterally, sometimes more so anteriorly; very broad, each plate usually at least 1/3 width of frons, sometimes nearly touching medially and sometimes slightly narrower in female; less commonly as narrow as 1/4 width of frons. Fronto-orbital plate and most of ocellar triangle subshiny. Frons and eye bulging anterodorsally.

***Chaetotaxy***: Two widely spaced ori; two ors. Orbital setulae relatively dense, long and bushy, especially in males where setulae usually exceed length of setulae on eye; in up to three or four rows; mostly lateroclinate, reclinate to erect, inner rows partially proclinate; brownish yellow. Eye narrowly pilose dorsomedially in male, nearly bare in female; relatively sparse on both. Two dorsocentral setae. Acrostichal setulae in eight irregular rows. Mid tibia with two posteromedial setae.

***Colouration***: Body, including halter, dark brown in base colour. Notum with light greenish shine, sometimes bluish. Calypter margin and hairs white. Abdomen (excluding tergite 1) strongly coppery or metallic green, but sometimes hints of both colours present; sometimes bluish in male or with stronger metallic blue shine in female.

***Genitalia***: (Figs 318–323) Epandrium with regularly developed to slightly elongate posterodistal spine. Surstylus narrow, produced and angled posteroventrally, with two or three irregular rows of tubercle-like setae that sometimes extend along entire length of surstylus or extend nearly to base in one to two rows; apical setae very elongate and either clustered apically or (more frequently) extended in irregular row to midpoint of surstylus. Basiphallus slightly separated from phallophorus, U-shaped with dorsal section straight; overlapping or nearly overlapping distiphallus. Mesophallus inserted before midpoint of distiphallus; base exceeding that of distiphallus. Distiphallus teardrop-shaped with ventrodistal surface dark, plate-like and with margins slightly to strongly sinuate (especially pronounced in illustrated male from Chicago); internal spinulose structure well-developed; ventrolateral tubules small, recessed.

***Variation***: (Figs 62, 63) Lectotype female, DC male and Falls Church, VA male with fronto-orbital plate only 1/4 width of frons, and consequently with fewer rows of setulae; males with orbital setulae as long as setulae on eye. Female lectotype with seta on hind tibia on outer surface at basal 1/3 and abdomen with unusually pronounced blue metallic shine.

###### Hosts.

Asteraceae – *Eupatoriumcapillifolium*, *Heterothecasubaxillaris*.

###### Distribution.

**Canada**: BC, ON, NB*, QC. **USA**: DC, DE*, FL, IL, ID, MA, MD, NC*, NJ, NY*, PA, SC, TN*, VA*.

###### Type material.

***Lectotype* [*virens*]: USA. PA**: “Penn”, “Loew coll”, “virens m.” (1♀, MCZ; type No. 15703).

***Holotype* [*heterothecae*]: USA. FL**: Hialeah, 18.vii.1962, ex. stem of *Heterothecasubaxillaris* (Lam.) Britt. And Rusby, C.E. Stegmaier (1♂, originally deposited in USNM). [Missing]

###### Additional material examined.

**Canada: NB**: Sainte-Anne-de-Kent, 46°34'N, 64°47'W, 28–29.vii.2013, O. Lonsdale, CNC358532 (1♂, CNC). **USA. DC**: Washington, 17.viii.1913, A.L. Melander (1♂, USNM), Washington, E bank CandO canal nr. Ariz. Ave., 23.i.1970, D.M. Anderson, reared from larva in stem *Vernonianoveboracensis*, 25.ii.1970 (1♂, USNM), Washington, 20.i.1969, D.M. Anderson, ex. stem of *Eupatoriumrugosum* [illegible], emerged indoors 5.iii.1969 (1♀, USNM), Washington, E Bank CandO Canal nr. Ariz. Ave., reared from larva in stem *Vernonianoveboracensis*, D.M. Anderson, 23.i.1970 (1♂, USNM), 28.iii.1970 (2♂ 1♀, USNM), 14.x.1969, reared 23.ii.1970 (3♀, USNM), Cropley, 20.x.1968, D.M. Anderson, ex. stem *Vernonianoveboracensis*, emerged indoors 16.ii.1969 (1♂, USNM), **DE**: Gumboro, 2.viii.1952, C. Sabrosky (1♂, USNM), Rehoboth, “4/8/41”, A.L. Melander (1♂, USNM), **FL**: Highlands Co., Archbold Bio. Sta., 13–19.iv.1970, W.W. Wirth (1♀, USNM), Cape Sable, 31.iii.1953, W.R.M. Mason (1♀, CNC), **IL**: Chicago, A.L. Melander, CNC358432 (1♂, CNC), **MD**: Glen Echo, 30.vii.1922, J.R. Malloch (1♂, USNM), Plummers Isl., 20.vii.1913, J.R. Malloch (1♂, USNM), **NC**: Carteret Co., Atlantic Beach, 3–4.ix.1986, G.F. and J.F. Steyskal (1♂ 1♀, USNM), Mongomery Co., 4mi SW of Ashton, Malaise trap, 26.v.1981, G.F. and J.F. Hevel (1♂, USNM), **NY**: Long Island, Cold Spring Harbor, A.L. Melander (1♀, USNM), Ithaca, 31.v.1914, A.L. Melander (1♂, USNM), Cld. Sp. Harb., L.I., A.L. Melander, CNC358430 (1♂, CNC), **PA**: Spring Br., 9.v.1945, DDT Expt (1♂, USNM), **TN**: Cocke Co., Twin Creeks Uplands Res. Lab, Great Smokey Mtn. N.P., 35°41.2'N, 83°30'W, 1900', 27.v.1999, S.D. Gaimari (1♂, USNM), **VA**: Shenandoah, Big Meadows, 3.vii.1939, A.L. Melander (1♂, USNM), Fairfax Co., Dead Run, 5.v.1915, R.C. Shannon, CNC358431 (1♀, CNC), Falls Church, 11.iv.1923, W,. Middleton (1♂, USNM).

###### Comments.

Nearctic *Melanagromyza* with a medially swollen fronto-orbital plate were treated as either *M.virens* or *M.splendida* in most of the previous literature. Spencer later described *M.virginiensis* in Spencer and Steyskal (1986b), and although he did not note the presence of this character in the description or key, a distinctly widened fronto-orbital plate is indeed present. A fronto-orbital plate ¼ the width of the frons is also found in *M.vernoniae*, *M.vernoniana*, *M.walleyi* Spencer, and several undescribed Nearctic species.

In addition to the swollen fronto-orbital plates, *Melanagromyzavirens* was defined by the possession of a distinct surstylus that was narrow, bent and with several long, stout apical setae, a character that led Spencer (1969) to include *M.heterothecae* as a junior synonym. Unfortunately the type specimens of *M.heterothecae* cannot be located, but the illustrations of the phallus in the original description (Spencer 1966b) agree with the structure of dissected *M.virens* from Florida to Canada, and the synonymy is here maintained. While the surstylus is partially diagnostic of the species, a similar (or identical) surstylus is found in other species, including *M.matricarioides*, which was incorrectly illustrated in Spencer (1969), and *M.walleyi* Spencer from Tennessee. The phallus of this latter species (Spencer and Steyskal 1986b: figs 44, 45) is narrower, widest past the middle and abruptly constricted subapically, and the internal spinulose structure is smaller.

The Albertan *Melanagromyzabidenticola* Sehgal (Sehgal 1971: figs 26–31) is also similar to *M.virens*, largely agreeing in external morphology and phallic structure. This species, however, is larger (2.8–3.0 mm ♂, 3.1–3.3 mm ♀), the ocellar triangle is longer, the eye is 4.9–5.9 × higher than the gena, the scutum is slightly more metallic and the overall pigment on the body is darker. With regards to the male genitalia, the surstylus is wider and not downturned, although the posterodistal setae are slightly thicker, the basiphallus is slightly thicker and the mesophallus is slightly longer (although this difference may be negligible).

##### Melanagromyza virginiensis

Taxon classificationAnimaliaDipteraAgromyzidae

Spencer

Figs 58 , 59 , 325–328

Melanagromyza virginiensis Spencer in Spencer and Steyskal 1986b: 248.

###### Description

**(Figs 58, 59).** Wing length 3.1–3.2 mm (♂), 2.8–3.3 (♀). Length of ultimate section of vein M4 divided by penultimate section: 0.6–0.7. Eye height divided by gena height: 3.7–5.9. Clypeus broadly rounded. Ocellar triangle slightly longer than wide. Fronto-orbital plate ¼ width of frons, widest near middle. Fronto-orbital plate and ocellar triangle subshiny.

***Chaetotaxy***: Two widely separated ori; two ors. Eye extensively pilose, with hairs sparse excluding dense dorsal patch. Orbital setulae erect to slightly proclinate, and mostly lateroclinate. Two dorsocentral setae. Acrostichal setulae in eight irregular rows. Mid tibia with two posteromedial setae.

***Colouration***: Body, including halter, dark brown in base colour. Notum with light bluish tint anteriorly (often absent) and more intense greenish shine posteriorly. Calypter margin and hairs white. Female anepisternum with greenish tint. Abdomen (excluding tergite 1) metallic green.

***Genitalia***: (Figs 325–328) Epandrium with small posterodistal spine. Surstylus 1/2-length of epandrium and with several rows of tubercle-like setae on inner surface. Hypandrium subtriangular with short apical process. Metepiphallus smooth ventrally and with short ridge on separate lateral sclerite. Proepiphallus narrow and weakly sclerotised. Basiphallus U-shaped and relatively long. Distiphallus separated from phallophorus by height of basiphallus; base of distiphallus extending slightly past base of closely held mesophallus; large, stout, and well-developed with especially long, broad, high distal section. Ejaculatory apodeme large and well-developed.

###### Hosts.

Asteraceae – Rudbeckialaciniata*.

###### Distribution.

KS*, MA*, MD*, NH*, NY*, PA*, VA.

###### Type material.

***Holotype*: USA. VA**: Montgomeny Co., Blacksburg, 28.v.1962, J.G. Chillcott, CNC358457 (1♂, CNC).

###### Additional material examined.

**USA. KS**: Lawrence, J.M. Aldrich (1♂, USNM), **MA**: Boston, May, A.L. Melander (1♀, USNM), **MD**: Glen Echo, 28.v.1919, J.M. Aldrich (1♀, USNM), Plummers Isl., at light, 28.iv.1914, R.C. Shannon (1♀, USNM), Chain Bridge, 12.ix.1913, R.C. Shannon (1♀, USNM), Montgomery Co., Bethseda, 3.vi.1972 (5♂, USNM), 28.v.1986 (1♀, USNM), 3.vi.1986 (2♀, USNM), 6.ix.1981 (1♂, USNM), Howard Co., Fulton, 21.v.1989, M.J. and R. Molineaux, J.E. Creeden (1♂, USNM), **NC**: Carteret, Co., Atlantic Beach, 3–4.ix.1984, G.F. and J.F. Hevel (1♀, USNM), **NH**: Franconia Ntch, 8.vii.1931, A.L. Melander (2♀, USNM), **NY**: Ithaca, 31.v.1914, A.L. Melander (1♂ 2♀, USNM) , New York City, “vCortlndPk”, 15.v.1926, A.L. Melander (1♀, USNM), **PA**: Pittsburg, McCandless Township, Allegheny Co., J. Plakidas, 20.ix.1978, “fly larva feed on gall tissue” (1♂, USNM), **VA**: Glencarlyn, 12.vi.1920, J.R. Malloch (1♂, USNM), Glencarlyn, 21.viii.1929, emg., “8-9.29”, *Rudbeckia* gall, J.C. Bridwell (1♂, USNM), nr. Plummers Isl., 19.x.1914, R.C. Shannon (1♀, USNM), Montgomery Co., Bethseda, 3.vi.1972, G. Steyskal (2♀, USNM), Talbot Co., McDaniel (Wades Point), 19–21.ix.1986, Malaise trap, salt marsh with flowering *Baccharis*, W.E. Steiner (1♀, USNM), Northampton Co., Kiptopeke, 4–6.x.1986, W.E. Steiner et al. (1♂ 1♀, USNM).

###### Comments.

*Melanagromyzavirginiensis* has widely separated ori, as in all other species of the *M.virens* group, and the fronto-orbital plate is only ¼ the width of the frons. The phallus is most diagnostic, with the distiphallus being especially long and stout-bodied with the distolateral margins nearly parallel and the ventrolateral tubules pronounced.

While the host species is not mentioned on any of the specimen labels, the male collected by Plakidas was reared from *Rudbeckialaciniata* (Plakidas 1982). It is interesting that this *Melanagromyza* atypically induces a gall, as apparent from both Plakidas’ specimen and the reared specimen from Virginia.

##### Ophiomyia

Taxon classificationAnimaliaDipteraAgromyzidae

Braschnikov

Ophiomyia Braschnikov, 1897: 40 [as subgenus of Agromyza]. Type species: Agromyzamaura Meigen, 1838 [misidentified as *curvipalpis* Zetterstedt], by monotypy. Hendel 1920: 114 [as genus]; Frick 1952a: 375; Spencer 1964: 775, 1969: 81; Spencer and Steyskal 1986b: 37; Černý 1994: 455; Lonsdale 2014: 486.
Tylomyza Hendel, 1931: 181 [as subgenus of Ophiomyia]. Type species: Madizapinguis Fallén, 1820, by original designation. Enderlein 1936a: 179 [as genus]. Syn. Spencer (1964a).
Stiropomýza Enderlein, 1936a: 179. Type species: Phytomyzaaeneonitens Strobl, 1893, by monotypy. Syn. Frick (1952a) [not explicit].
Siphonomyza Enderlein, 1936a: 179. Type species: Agromyzaproboscidea Strobl, 1900, by monotypy. Syn. Frick (1952a) [not explicit].
Aulomyza Enderlein, 1936a: 179. Type species: Melanagromyzalongilingua Hendel, 1920, by monotypy. Frick 1952a: 375 [as synonym of Melanagromyza]. Syn Spencer (1966a) [as synonym of Ophiomyia].
Siridomyza Enderlein, 1936a: 179. Type species: Ophiomyiamadizina Hendel, 1920 [=A.nasuta Melander], by monotypy. Syn. Frick (1952a) [as synonym of Tylomyza, not explicit].
Solenomyza Enderlein, 1936a: 179. Type species: Melanagromyzarostrata Hendel, 1920, by monotypy. Frick 1952a: 375 [as synonym of Melanagromyza]. Syn Spencer (1966a) [as synonym of Ophiomyia].
Stirops Enderlein, 1936a: 179 [nomen nudum – no type species designated].
Stirops Enderlein, 1936b: 42 [attributed to Enderlein 1936a]. Type species: Ophiomyiasubmaura Hering, 1926, by original designation [Enderlein 1936b: 42]. Syn. Frick (1952a) [not explicit].
Triopisopa Enderlein, 1936a: 179 [nomen nudum – no type species designated].
Triopisopa Enderlein, 1936b: 42. Type species: Agromyzasimplex Loew, 1869, by original designation. Frick 1952a: 375 [as synonym of Melanagromyza]. Syn. Spencer (1966a) [as synonym of Ophiomyia].
Hexomyza Enderlein, 1936a: 179 [nomen nudum – no type designation].
Hexomyza Enderlein, 1936b: 42 [attributed to Enderlein (1936a)]. Type species: Melanagromyzasarothamni Hendel, 1923, by original designation. Hendel 1936: 570 [as synonym of Melanagromyza – followed by Frick (1952a)]; Spencer 1966a: 38, 1969: 79; Spencer and Steyskal 1986b: 34. Syn. Lonsdale (2014).
Carinagromyza Sasakawa, 1954: 23. Type species: Carinagromyzaheringi Sasakawa, 1954 [= OphiomyiasasakawaiSpencer and Martinez 1987], by original designation. Spencer and Martinez 1987 [synonymy].
Penetagromyza Spencer, 1959: 253. Type species: Penetagromyzaaloes Spencer, 1959, by original designation. Spencer 1990: 390, 1991: 57. Syn. Lonsdale (2014).
Kleinschmidtimyia Spencer, 1986: Spencer, 1986: 249. Type species: Melanagromyzapisi Kleinschmidt, 1961, by original designation. Spencer 1990: 391. Syn. Lonsdale (2014).

*Ophiomyia* is a diverse, widespread genus sometimes mistaken for other Agromyzinae such as *Melanagromyza* and *Euhexomyza*, but many species exhibit unusual diagnostic external features and varied male genitalic morphology. All examined Nearctic species and virtually all global species, can be diagnosed by an apically truncated clypeus, as discussed in Lonsdale (2014). While the clypeus may sometimes be bulging or broadly rounded, the anterolateral margins are angulate, not rounded. The clypeus is usually also very thin and elongate, with the arms frequently bowed outwards, being particularly pronounced in those species with an anteriorly produced gena. Although several *Melanagromyza* such as *M.buccalis* and the new species *M.glyptos* approach the derived state seen in *Ophiomyia*, their relationship with the rest of *Melanagromyza* is revealed by dorsally pilose eyes, ventrolateral tubules on the distiphallus and a symmetrical basiphallus.

Other useful diagnostic characters of *Ophiomyia* are as follows: if present, there is only a single posteromedial seta on the mid tibia (usually two in other Agromyzinae); the calypter is usually brown marginally (white in most other Agromyzinae); there is usually a medial vertical carina separating the antennal bases and the centre of this carina usually also has a medial swelling that is spindle-shaped to subspherical. The gena is also often produced anteriorly, at least slightly, and can be strongly produced with an apical fasciculus. This fasciculus is an aggregation of multiplicated vibrissae that are variably fused to produce a “horn”. In many species, the inner lobe of the hypandrium is differentiated into two distinct sclerites (but see *O.simplex*): a setulose, arched sclerite (also found in *Melanagromyza* and *Euhexomyza*) and a flat subovate sclerite. The proepiphallus is also expanded laterally to form one pair of upcurved, strongly pigmented lobes (plate-like in *O.simplex*), the metepiphallus usually has one pair of ventromedial spines (not multiple spines, as is characteristic of most *Melanagromyza*), and the sclerites of the basiphallus are usually fused basally with the left sclerite atrophied. Lastly, the distiphallus is usually somewhat asymmetric, being slightly twisted sinistrally, and there are no paired ventrolateral tubules (characteristic of *Melanagromyza*).

### ﻿Key to the species of Delmarva *Ophiomyia*

**Table:** 

1	Orbital setulae proclinate (Figs 64, 65). Three pairs of dorsocentral setae. Male without ors (Fig. 65); female with one or two ors (Fig. 64). Distiphallus with minutely tuberculate bilobed distal section (Figs 379, 380). Metepiphallus with multiple pairs of ventromedial spines	***O.nasuta* (Melander)**
–	Orbital setulae reclinate. Two pairs of dorsocentral setae. Two pairs of ors. Distiphallus not as above. Metepiphallus almost always with one pair of ventromedial spines	2
2	Costa extending just past vein R_4+5_. Gena highest behind midpoint of eye (Fig. 70). Fronto-orbital plate and parafacial shiny, prominent, and easily viewed laterally. Bands of basiphallus twisted counter-clockwise (Figs 392, 393). Inner setulose band of hypandrium fused to lobe (Fig. 390)	***O.simplex* (Loew)**
–	Costa extending to vein M_1+2_. Gena highest anterior to midpoint of eye. Fronto-orbital plate and usually parafacial barely visible laterally. Bands of basiphallus either fused and collar-like basally, or with right band produced. Inner band of hypandrium separate from lobe	3
3	Clypeus broad and nearly parallel-sided along length (Fig. 71). Face with weak carina dividing antenna, if present (Fig. 71). Gena not produced, vibrissal angle > 90° (Fig. 72). Male without vibrissal fasciculus. Distiphallus small, bulb-like (Fig. 331). Basiphallus basal and collar-like	4
–	Clypeus usually slightly to strongly narrowed anteriorly (Fig. 77). Face with carina (sometimes indistinct) with medial swelling below antennal base (Fig. 75). Gena at least slightly projecting, vibrissal angle 90° or less (Fig. 76). Male with fasciculus. Distiphallus relatively dark, large, and globular/ovate in shape (Figs 358, 387). Basiphallus produced on right side	7
4	Gena entirely receding along anterior margin. Distiphallus darker and subcylindrical, without flagellum	5
–	Gena slightly angled on anterior margin, not immediately vertical or receding (similar to *Melanagromyza* spp.). Distiphallus pale and bulb-like with terminal clear flagellum	6
5	Eight rows of acrostichal setae. Wing length 2.5 mm. Distiphallus broad and parallel-sided apically (Fig. 406)	***O.ultima* (Spencer)**
–	Six rows of acrostichal setae. Wing length 2.0–2.3 mm. Distiphallus and mesophallus narrow, linear and with bell-shaped apex (Figs 353, 354)	***O.kalia* sp. nov.**
6	Eye 5.0–6.7 × higher than gena. Two ori. Fronto-orbital plate slightly visible laterally. Distiphallus relatively small and spade-shaped, with small distolateral sclerotisations and no ventral elaboration of membrane; apical flagellum directed distally (Figs 330, 331)	***O.abutilivora* Spencer**
–	Eye 2.5–4.3 × higher than gena. Often three ori, at least on one side, with orbital seta between ori variably developed. Fronto-orbital plate and parafacial projecting, with parafacial continuing under eye as distinct cheek. Distiphallus rounded, with large ventral carina, and often with pronounced lateral "wings”; apical flagellum directed ventrally (Figs 401, 402)	***O.tiliae* (Couden)**
7	Facial bulb with extensive shiny section on dorsal 1/2; sides of carina above bulb usually parallel-sided basally. Ocellar triangle shiny, less commonly subshiny. Abdomen with faint metallic shine (often difficult to see). Mid tibia with single posteromedial seta	8
–	Facial bulb and ocellar triangle not shiny (shiny in some *O.coniceps*, but these with very pronounced genal process that is longer than high, and male with very short, wide fasciculus); sides of facial carina above bulb immediately divergent. Abdomen brown to glossy black, rarely coppery (*O.cuprea*). Mid tibia with or without posteromedial seta	**10**
8	Eye 4.0–6.0 × higher than gena. Ocellar triangle attaining anterior margin of frons as thin line. Surstylus narrow, ~ 1/3 length of epandrium. In profile, distiphallus widest subapically (Figs 358, 359)	***O.kwansonis* Sasakawa**
–	Eye 8.0–8.4 × higher than gena. Ocellar triangle not attaining anterior margin of frons, and usually not distinct anterior to ors. Surstylus ~ 1/2 length of epandrium. In profile, distiphallus widest near base	9
9	Three ori sometimes present on one or both sides of frons. Facial bulb smooth or only slightly furrowed (Fig. 78). Phallus with strong distoventral membranous keel. Basiphallus short, not reaching mesophallus. Distiphallus with inner surface tuberculate to weakly spinulose distally, and more strongly spinulose basally; shape subovate (seen ventrally); dorsum smoothly sloping to apex (seen laterally) (Figs 365, 366)	***O.labiatarum* Hering**
–	Only two ori. Facial bulb with strong medial furrow. Phallus with weak, sac-like distoventral membrane. Basiphallus reaching past base of mesophallus. Distiphallus with medial lobe strongly spinulose internally; shape elongate with base tapered (seen ventrally); distal 1/2 abruptly flattened (seen laterally) (Figs 411, 412)	***O.virginiensis* Spencer**
10	Body brown to light brown with fronto-orbital plate and ocellar triangle (excluding tubercle) white/beige. Frons with anteromedial furrow. Wing veins whitish. Eye nearly as wide as high, with long axis nearly vertical. Basiphallus extensively sclerotised along right margin, strongly bent ventrally, and membrane thickened and lightly sclerotised distoventrally (Fig. 346)	***O.galiodes* sp. nov.**
–	Body dark brown with parts of frons sometimes slightly paler, but never whitish. Frons evenly rounded anteriorly. Wing veins light brown to brown. Eye distinctly higher than wide, with long axis often diagonal. Phallus not as above	**11**
11	Five to six rows of acrostichal setulae. Abdomen brown with weak coppery luster. Distiphallus relatively dark and elongate with distal 1/2 tapering and left-distal surface minutely tuberculate; with long, dark, upcurved and pointed posteromedial spur (Figs 344, 345)	***O.cuprea* sp. nov.**
–	Eight to ten rows of acrostichal setulae. Abdomen brown to black. Distiphallus not as above; if with posteromedial spur, then spur short, straight, and blunt	**12**
12	Three ori. Genal angle 70°–90°. Mid tibia with single weak posteromedial seta. Notopleuron and postpronotum reddish or eye 2.8 × higher than gena. Distiphallus widest at midpoint and broadly rounded (viewed ventrally), with surface strongly ridged and furrowed (Figs 386, 387)	***O.sexta* Spencer**
–	Two ori. Genal angle often < 60° or 45°, rarely 90° (*O.heleios*). Mid tibia usually without posteromedial setae. Notopleuron and postpronotum the same colour as the rest of the scutum and gena not particularly high. Shape of distiphallus variable, but not as above	**13**
13	Vibrissal fasciculus shorter than length of genal process (Figs 75–77). Parafacial prominent. Mesophallus with small dark transverse band across point of insertion with distiphallus; distiphallus with small to large lobe projecting from left ventral margin (Figs 340, 341)	***O.coniceps* (Malloch)**
–	Fasciculus longer than genal process. Parafacial barely visible laterally. Mesophallus without transverse band; distiphallus without lobe as above	**14**
14	Genal process short, with dorsal margin straight (not concave), forming a 60°-90° angle (as in Fig. 79). Distiphallus with strong ventromedial sclerotisation (broad to thin and band-like); inner surface of distiphallus lightly spinulose and outer surface smooth	**15**
–	Genal process of variable length, but dorsal margin curving to a narrow point (as in Fig. 76). Distiphallus not excessively sclerotised distoventrally; either inner surface covered with numerous stout spines or outer surface minutely tuberculate apically	**16**
15	Genal process forming a slightly < 90° angle. Wing length 2.5 mm. Notum dark brown with light green reflection (reflection absent on postpronotum and notopleuron). Base of femora paler, yellow to base ventrally on mid and hind legs. Calypter white with hairs brown. Distiphallus with thick distoventral cluster of ridges (Figs 349, 350)	***O.heleios* sp. nov.**
–	Gena strongly produced, forming an approximate 45° angle. Wing length 1.7 mm. Notum and femora dark. Calypter margin and hairs brown. Distiphallus with thin distoventral ridge (Fig. 336)	***O.capitolia* sp. nov.**
16	Scutum bare medially behind anterior dorsocentral. Surstylus with 4 apical tubercle-like spines (Fig. 395). Distiphallus relatively thin and C-shaped in lateral view; posterodorsal margin with tail-like extension (Fig. 397)	***O.texana* (Malloch)**
–	Scutum setulose medially behind anterior dorsocentral, at least partially. Surstylus with numerous apical spines. Distiphallus stout and irregular in lateral view; without tail-like process	**17**
17	Wing length 2.2–2.3 mm. Mid tibia without posteromedial setae. Facial bulb narrow and elongate, not much wider than remainder of keel. Distiphallus pale, subquadrate (ventral view), densely spinulose on inner surface (Figs 368, 369)	***O.laticolis* sp. nov.**
–	Wing length 1.8–2.0 mm. Mid tibia with one posteromedial seta. Facial bulb broadly rounded. Distiphallus longer than wide, with short, but distinct tubule emerging dorsoapically; inner surface smooth (Figs 375, 376)	***O.maura* (Meigen)**

#### Species descriptions

Unless otherwise stated, the following species are assumed to be as follows: Body, including halter, brown. Orbital setulae reclinate. Notum subshiny with two dorsocentrals and eight to ten rows of acrostichal setulae. Costa extending to vein M_1+2_. Hypandrium differentiated into two distinct sclerites: a setulose, arched sclerite and a flat subovate sclerite. Proepiphallus expanded laterally to form one pair of upcurved, strongly pigmented lobes.

##### Ophiomyia abutilivora

Taxon classificationAnimaliaDipteraAgromyzidae

Spencer

Figs 71 , 72 , 329–331

Ophiomyia abutilivora Spencer in Spencer and Steyskal 1986b: 250; Eiseman et al. 2021: 16.

###### Description

**(Figs 71, 72).** Wing length 2.0–2.3 mm (♂♀). Length of ultimate section of vein M4 divided by penultimate section: 0.6. Eye height divided by gena height: 5.0–5.7. Facial carina present with narrow medial bulb. Gena slightly angled forward, highest near midpoint of eye. Clypeus slightly tapering apically. Fronto-orbital plate very narrow, ocellar triangle nearly extending to posterior ori. Notum shiny.

***Chaetotaxy***: Orbital setulae reclinate. Male vibrissal fasciculus absent. Two ori; two ors. Mid tibia with one posteromedial seta.

***Colouration***: Body, including halter dark brown with gena paler. Wing veins yellow to brown. Calypter margin and hairs brown.

***Genitalia***: (Figs 329–331) Inner posterodistal margin of epandrium with rows of minute tubercles. Distal margin of surstylus broad and truncated with rows of pointed tubercle-like setae on inner surface. Metepiphallus with subapical spine. Basiphallus with slender dorsoventral arms, partially fused to phallophorus. Hypophallus membranous, carinate. Distiphallus small and spade-shaped (broad basally and strongly tapered apically); apex with long, tapered flagellum that has base surrounded by light sclerotisations; mesophallus narrow, barely wider than duct. Ejaculatory apodeme stout, well-developed, with blade relatively narrow.

###### Hosts.

Malvaceae – *Abutilontheophrasti*, *A.permolle*. Adults taken from *Sidacordifolia* (Spencer and Steyskal 1986b) and possibly *Alcea*.

###### Distribution.

**USA**: DE*, FL, IA, MD*, MN, MS; stem mines only in IL and WI (Eiseman et al. 2021).

###### Type material.

***Holotype*: USA. MN**: Dakota Co., Rosemount, Agricultural Research Station, 13.ix.1978, R.N. Andersen and R. Ralston (1♂, USNM).

###### Paratypes examined

**. USA. FL**: Broward Co., Ft. Lauderdale, 14.iii.1980, H.E. Walker, Fl. *Abutilonpermolle*, **MN**: Rosemount, 13.ix.1987, ex. *Abutiliontheophrasti* (1♂ 3♀, USNM), **MS**: Stoneville, 22.x.1979, K.E. Frick, 79-25, ex. stem gall *Abutiliontheophrasti* (4♂ 1♀, USNM), Merigold, 22.x.1979, K.E. Frick, 79-25, ex. stem gall *Abutiliontheophrasti* (1♂ 2♀, USNM).

###### Additional material examined.

**USA. DE**: Newark, 6.ix.1960, hollyhock, P. Burbutis (2♂, USNM), Newark, “8/10/1953” (1♂, USNM), **IA**: Winneshiek Co., Decorah, Trout Run Trail, 43°18'5.04"N, 91°48'6.60"W, 10–13.viii.2017, J. van der Linden, *Abutiliontheophrasti*, em. by 26.vii.2017, #CSE4940, CNC1643668, CNC1643669 (1♂ 1♀, CNC), **MD**: Montgomery Co., 4mi SW of Ashton, 6.ix.1981, Malaise trap, G.F. and J.F. Hevel (1♂, USNM).

###### Comments.

The distiphallus of *Ophiomyiaabutilivora* is highly reduced and lobate, with the apex continuing distally as a long membranous flagellum. A similar phallus is found in the larger *O.tiliae*, but the clypeus of this species is nearly rounded, the distiphallus appears pear-shaped when viewed ventrally, the apical flagellum points ventrally and there is one pair of membranous "wings” ventral to the distiphallus. Other putative Nearctic relatives (based on the genitalic figures in Spencer and Steyskal 1986b and Spencer 1969) are more western in distribution, including the Californian species *O.bernardensis* Spencer, *O.shastensis* Spencer, O. *jacintensis* Spencer, and O. *yolensis* Spencer, and possibly O. *monticola* Seghal from northern Canada and Alaska.

##### Ophiomyia capitolia sp. nov.

Taxon classificationAnimaliaDipteraAgromyzidae

http://zoobank.org/2AF86F61-1381-4388-A46E-91DADF1AA0A5

Figs 332–336

###### Description.

Wing length 1.7 mm (♂). Female unknown. Length of ultimate section of vein M4 divided by penultimate section: 0.8. Eye height divided by gena height: 6.2. Eye slightly angled diagonally. Facial carina stout, slightly more narrow than stout bulb. Gena shallowly produced, projecting at 60° angle with margins straight. Clypeus narrow and truncated. Buccal cavity narrowed anteriorly with anterior margin straight. Distance between crossveins more than length of dm-m. Ocellar triangle and fronto-orbital plate (narrow) subshiny and ill-defined.

***Chaetotaxy***: Male with vibrissal fasciculus ~ 1/2 length of gena. Two ori; two ors. Mid tibia with one posteromedial seta.

***Colouration***: Body, including halter dark brown. Wing veins brown. Calypter margin and hairs brown.

***Genitalia***: (Figs 332–336) Metepiphallus small, pale and with one pair of ventromedial spines. Epiphallic lobes clear with dark floating sclerite. Halves of basiphallus broadly fused at base with remainder of left sclerite absent; right sclerite relatively short and truncated with long distoventral process reaching venter. Mesophallus continuing distally as thick ridge that bifurcates apically; distiphallus pale and globular with inner surface minutely spinulose. Ejaculatory apodeme with long dark stalk and several annulations on striated blade; sperm pump with weak sclerotisation.

###### Host.

Unknown.

###### Distribution.

**USA**: DC.

###### Etymology.

The specific name refers to the fact that the type was collected in the United States capitol.

###### Type material.

***Holotype*: USA. DC**: Washington, 17.viii.1913, A.L. Melander (1♂, USNM).

###### Comments.

*Ophiomyiacapitolia* is one of the smaller Delmarva *Ophiomyia* with a male wing length of 1.7 mm. This species also has a distinct genal process, a long fasciculus and a relatively distinct wrinkle under the eye within the cheek. Only examination of the male genitalia can provide reliable identification of this species (see key).

##### Ophiomyia coniceps

Taxon classificationAnimaliaDipteraAgromyzidae

(Malloch)

Figs 75–77 , 82 , 337–341

Agromyza coniceps Malloch, 1915a: 107.
Ophiomyia coniceps . Frick 1952a: 382; Spencer 1969: 85, 1981: 75; Spencer and Steyskal 1986b: 46; Shi, Chunyan and Gao 2015: 59; Eiseman and Lonsdale 2018: 19.

###### Description

**(Figs 75–77, 82).** Wing length 1.7–1.9 mm (♂), 2.4 mm (♀). Length of ultimate section of vein M4 divided by penultimate section: 0.9–1.0. Eye height divided by gena height: 6.8. Facial carina distinct with large medial bulb; carina as broad as bulb dorsally with sides diverging. Eye angled diagonally. Gena strongly produced anteriorly, forming an ~ 45° angle. Parafacial also strongly produced, visible laterally, and blending into gena. Fronto-orbital plate narrow. Clypeus strongly produced and narrowed anteriorly, with apex slightly expanded; sides bowed laterally, widest near base. Sides of ocellar triangle slightly concave; triangle subshiny but sometimes glossier. Crossveins separated by length of dm-m or less.

***Chaetotaxy***: Male vibrissal fasciculus short and upcurved, not longer than genal process. Two ori (not three, as stated in Spencer and Steyskal 1986b); two ors. Mid tibia without posteromedial setae.

***Colouration***: Body, including halter dark brown. Fronto-orbital plate and ocellar triangle reddish in DE male. Calypter margin and hairs dark brown. Abdomen sometimes with faint coppery/golden shine. Wing veins brown.

***Genitalia***: (Figs 337–341) Surstylus broad, rounded, short, slightly produced ventrally, inner-distal surface with two to three rows of tubercle-like setae. Hypandrium pointed apically, sides slightly bowed, basal arms sinuate. Postgonite typical, narrow with dorsal 1/2 weakly attached and dorsally directed. Phallophorus dark and constricted at base, narrow, venter longer, swollen and produced. Basiphallus with arms united at base, left arm short, weak, and ill-defined, right arm long, reaching level of mesophallus. Mesophallus cylindrical, clear, inserted ventromedially into distiphallus. Distiphallus, large, mostly empty with medial shelf-like process that is slightly angled apically; somewhat ovate in ventral view with basal 1/2 narrower and slightly longer; inner surface minutely textured basally, with longer spinules medially, apically with shallow tubercles and striations to left side and longer rounded spinules to right side; mostly open dorsally; left ventrodistal margin produced as characteristic lobe that may be modestly to strongly developed.

###### Host.

Asteraceae – *Antennariaplantaginifolia* and possibly other *Antennaria*; *Sonchusasper* (Eiseman and Lonsdale 2018).

###### Distribution.

**Canada**: BC, MB, ON, QC, SK. **USA**: CA, DE*, IN, LA, MA, OK, UT, VA*; possibly also AL, CT, IA, WI (Eiseman and Lonsdale 2018).

###### Type material.

***Holotype*: USA. UT**: Salt Lake, 14.viii.1914, ex. *Sonchusasper*, P.H. Timberlake (1♀, USNM; type No. 193904).

###### Additional material examined.

**Canada. MB**: Aweme, 2.vi.1916, N. Criddle, J.M. Aldrich (1♂, USNM). **USA. DE**: Newark, 1.vii.1974 (1♂, USNM), **IN**: Evansville, 7.v.1914, J.M. Aldrich (1♂, USNM), **LA**: Lake Charles, 9.vi.1917, J.M. Aldrich (1♂, USNM), **MA**: Hampshire Co., Granby, Mt. Norwottuck, 27.iii.2012, em. 11.iv–24.iv.2012, C.S. Eiseman, ex *Antennariaplantaginifolia* (5♂ 3♀, CNC), **OK**: Payne Co., Mehan, 36.014339°N, 96.996744°W, 10.xii.2015, em. 15.i.2016, M.W. Palmer, ex *Antennariaplantaginifolia*, #CSE2214, CNC653970–653972 (2♂ 1♀, CNC), 12.xii.2015, em. by 12.i.2016, M.W. Palmer, ex *Antennariaplantaginifolia*, #CSE2216, CNC653988 (1♂, CNC), 14.i.2016, em. by 10–16.ii.2016, M.W. Palmer, ex *Antennariaplantaginifolia*, #CSE2237, CNC654000–654002 (3♂, CNC), 27.ii.2017, em. 18.iii– 15.vi.2017, C.S. Eiseman, ex *Antennariaplantaginifolia*, #CSE3229, CNC939913–939917 (3♂ 2♀, CNC), **VA**: Luray, 24.vi.1933, A.L. Melander (1♂, USNM), Page Co., 7mi W of Lunay, 8.vii.1978, G.F. Hevel (1♂, USNM), Shenandoah, Big Meadows, 3.vii.1939, A.L. Melander (1♂, USNM).

###### Comments.

*Ophiomyiaconiceps* will key to *O.apta* Spencer in Spencer and Steyskal (1986b: figs 224–226) because the number of ori in that work is incorrectly recorded as three, not two. This can no longer be directly verified from the holotype, however, since the head is missing, but the original illustration and description in Malloch (1915) clearly indicates two ori. *Ophiomyiaconiceps* has the most pronounced genal process of any Delmarva species, a strongly projecting parafacial and most important for diagnostic purposes, a very short and sometimes wide vibrissal fasciculus that is usually sharply pointed. The male genitalia are similar to those of other *Ophiomyia* with a fasciculus, but the outline of the distiphallus, the longer inner-medial spinules, and especially its small left ventrodistal lobe are unique.

##### Ophiomyia cuprea sp. nov.

Taxon classificationAnimaliaDipteraAgromyzidae

http://zoobank.org/68A6FCAD-96AA-4CB8-876C-6BAE77FA59CA

Figs 342–345

###### Description.

Wing length 2.2 mm (♂). Female unknown. Length of ultimate section of vein M4 divided by penultimate section: 0.9. Eye height divided by gena height: 9.3. Facial carina relatively long below, nearly as wide as bulb and with diverging margins above. Clypeus narrow. Gena slightly projecting, forming almost a 60° angle. Ocellar triangle subshiny around ocelli. Fronto-orbital plate thin and subshiny. Buccal cavity narrowed anteriorly with anterior margin straight. Distance between crossveins more than length of dm-m.

***Chaetotaxy***: Male with vibrissal fasciculus ~ 2/3 length of gena and strongly upcurved. Two ori on right side, three on left; two ors. Setae on mid tibia not visible, possibly absent. Five to six rows of acrostichal setulae.

***Colouration***: Body, including halter dark brown. Calypter margin and hairs brown. Abdomen with weak coppery shine. Veins light brown.

***Genitalia***: (Figs 342–345) Surstylus ~ 1/2 length of epandrium, rounded marginally and with tubercle-like setae on inner surface. Metepiphallus pale with one pair of ventromedial spines. Epiphallic lobe clear with dark sclerite. Halves of basiphallus broadly fused at base with remainder of left sclerite absent; right sclerite apically broad and truncated. Distiphallus dark and well-sclerotised with distinct basal and distal sections – basal section high, tapered to base, minutely spinulose internally and with internal dorsomedial hook; distal section shallower, with left margin receding and minutely tuberculedridged, and right 1/2 longer, internally spinulose and ill-defined apically.

###### Host.

Unknown.

###### Distribution.

**USA**: MD.

###### Etymology.

The specific epithet is derived from the Latin for coppery, referring to the colour of the abdomen, which has a weak metallic lustre.

###### Type material.

***Holotype*: USA. MD**: nr. Plummers Isl., 5.v.1915, R.C. Shannon (1♂, USNM).

###### Comments.

The head (particularly the gena) and genitalia of *Ophiomyiacuprea* are similar to those of the Californian *O.delecta* Spencer (Spencer and Steyskal 1986b: figs 271, 272), but *O.delecta* differs in having eight rows of acrostichal setulae (not five to six), a black (not coppery) abdomen and a facial bulb that is much narrower than the pedicel (Spencer 1981). Furthermore, based on Spencer’s illustration, the distiphallus of that species is more broadly rounded basally and atrophied apically. Spencer (1981) also describes the similar *O.definita* Spencer (later provided the new name *O.subdefinita* Spencer; Spencer and Steyskal 1986b: figs 268, 269) in his Californian revision, but the fasciculus is straight (not curved), the abdomen is also black and the distiphallus is much paler; the number of rows of acrostichal setulae are not mentioned, but it is indicated that there are also eight.

##### Ophiomyia galiodes sp. nov.

Taxon classificationAnimaliaDipteraAgromyzidae

http://zoobank.org/8AD07980-D922-4887-86B7-E18FC80CE9AE

Figs 346 , 347

###### Description.

Wing length 2.2 mm (♂), 1.9–2.2 (♀). Length of ultimate section of vein M4 divided by penultimate section: 0.6–1.0. Eye height divided by gena height: 6.2–9.8. Facial carina (wide dorsally) and bulb distinct. Parafacial, seen laterally, broadly rounded and projecting past anterior margin of gena. Gena slightly produced anteriorly, forming an angle of ca. 70° and with dorsal and ventral margins straight, not curved. Clypeus broadest posteriorly; apex very narrow, produced. Buccal cavity narrowed anteriorly with margin straight. Ocellar triangle concave subapically. Frons slightly furrowed medially. Crossveins separated by more than length of dm-m.

***Chaetotaxy***: Vibrissal fasciculus well-developed, slightly upcurved. Two ori; two ors. Mid tibia without posteromedial setae.

***Colouration***: Body, including halter dark brown, but relatively pale compared to congeners. Fronto-orbital plate and outer margin of ocellar triangle beige. Wing veins white. Calypter margin light brown with hairs brown.

***Genitalia***: (Figs 346, 347) Surstylus slightly < 1/2 length of epandrium, with several marginal tubercle-like setae. Metepiphallus pale and largely desclerotised with one pair of small ventromedial spines. Proepiphallus pigmented medially, darker ventrally. Halves of basiphallus broadly fused at base with remainder of left sclerite absent; right sclerite relatively short and ill-defined; strongly bent ventrally (possibly an artifact of preservation). Distiphallus short, strongly spinulose internally and with base extending past that of mesophallus; membrane distal to phallus relatively broad, flat, and lightly sclerotised.

###### Host.

Rubiaceae – *Galium*.

###### Distribution.

**USA**: MD.

###### Etymology.

The specific name indicates the similarity of this species to its possible sister taxon, *Ophiomyiagalii*, which also feeds on *Galium*.

###### Type material.

***Holotype*: USA. MD**: Beltsville, 16.iv.1975, S.W.T. Batra, ex. *Galium* sp. (1♂, USNM).

***Paratypes*: USA. MD**: Same collection as holotype (1♀, USNM; 1♀, CNC).

###### Comments.

The phallus of *Ophiomyiagaliodes* (only illustrated in lateral view due to inflexibility of the structure) is similar to that of *O.laticolis* (Figs 368, 369), but the distiphallus is narrower, larger and closer to the apex of the basiphallus. Furthermore, the eye is larger, rounder and bulging anteriorly, and the shoulders, fronto-orbital plate and ocellar triangle are pale, although these colour characters may be an artifact of preservation.

*Ophiomyiagaliodes* is also similar to the European *O.galii* Hering, which is the only other *Ophiomyia* known from *Galium* (Spencer, 1990). Their chaetotaxy, wing length and wing vein proportions are nearly the same, and they both have a ventrally curved hypandrium and similar distiphallus. The gena of *O.galii* is produced at a 45° angle (not 70°) (Spencer 1972), the basiphallus is longer and less spinulose, and the mesophallus is longer, narrower and extends past the base of the distiphallus (Papp and Černý 2015: fig. 152).

##### Ophiomyia heleios sp. nov.

Taxon classificationAnimaliaDipteraAgromyzidae

http://zoobank.org/27079288-6A25-4C9F-AC2D-11BCE6DCF7D2

Figs 348–350

###### Description.

Wing length 2.5 mm (♂). Female unknown. Length of ultimate section of vein M4 divided by penultimate section: 0.9. Eye height divided by gena height: 7.5. Facial carina long and narrow with small subspherical bulb at midpoint. Gena not strongly produced, forming slightly < 90° angle. Clypeus narrow with posterior arms curved outwards. Ocellar triangle shiny black, longer than wide and tapering anteriorly.

***Chaetotaxy***: Orbital setulae reclinate. Male vibrissal fasciculus long, narrow, and slightly upcurved. Two ori; two ors. Mid tibia with one small posteromedial seta.

***Colouration***: Body, including halter dark brown, with gena paler, ocellar triangle black, notum (excluding postpronotum and notopleuron) with faint green reflection, and femora paler on basal 3/5 with mid and hind femora yellowish ventrally on basal 2/5. Calypter white with hairs dark brown.

***Genitalia***: (Figs 348–350) Surstylus narrow with few inner-marginal tubercle-like setae. Distal margin of hypandrium long and tapered. Metepiphallus very small and with one pair of spines. Halves of basiphallus very ill-defined apically, slightly fused at base and with left side shorter. Base of distiphallus strongly exceeding base of mesophallus, with short dorsal margin (curled inwards and with several very short inner spines) that is atrophied on left side; distoventral margin of distiphallus well-sclerotised medially, produced as apically converging ridges. Ejaculatory apodeme atrophied to base on one side, clear along margins and with well-developed medial rib.

###### Host.

Unknown.

###### Distribution.

**USA**: VA.

###### Etymology.

Gr. “of a marsh, dwelling in a marsh”, referring to the collection locality of the type.

###### Type material.

***Holotype*: USA. VA**: Fairfax Co., Great Falls Park, swamp trail, 38°59.4'N, 77°15.2'W, Malaise trap, trap #2, 8.ix-11.v.2006, D.R. Smith (1♂, USNM).

###### Comments.

*Ophiomyiaheleios* can be differentiated from Nearctic congeners by a white calypter margin (although the hairs are still typically dark), a greenish notum, a short gena, a slightly atypical ejaculatory apodeme and an unusually atrophied distiphallus that has a thick distoventral sclerotisation medially.

##### Ophiomyia kalia sp. nov.

Taxon classificationAnimaliaDipteraAgromyzidae

http://zoobank.org/701D9943-A178-4AAD-BEFB-F4E34138AD0A

Figs 351–355

###### Description.

Wing length 2.1 mm (♂), 2.0–2.3 (♀). Length of ultimate section of vein M4 divided by penultimate section: 0.7–1.0. Head largely collapsed in holotype. Eye height divided by gena height: 5.0–6.6. Frons entirely pruinose and ocellar triangle indistinct; gena rounded, not produced anteriorly and relatively narrow along entire length. Face with weak carina, barely dividing antennae. Clypeus with sides parallel.

***Chaetotaxy***: Orbital setulae reclinate. Male vibrissal fasciculus absent. Two ori (sometimes three on one side); two ors. Mid tibia without posteromedial setae. Six rows of acrostichal setulae.

***Colouration***: Body, including halter dark brown, with gena slightly paler. Wing veins brown. Calypter margin and hairs brown.

***Genitalia***: Surstylus 1/2 length of epandrium, broadly rounded and with tubercle-like setae along inner-distal margin. Metepiphallus very small with serrated ridges and spines. Proepiphallus with dark patches in lateral extensions. Basiphallus curved in cross-section, ventrally curved and receding dorsomedially. Mesophallus broad, basal to distiphallus (not inserted ventromedially to distiphallus). Distiphallus straight, cup-like with internal folds. Ejaculatory apodeme large with blade pale and ovate, with pronounced medial rib.

###### Host.

Unknown.

###### Distribution.

**USA**: VA.

###### Etymology.

The specific epithet is Greek for bird nest, referring to the nest from where the holotype was collected.

###### Type material.

***Holotype*: USA. VA**: Long Bridge, 22.v.1913, ex. birds nests Pun grackle, R.C. Shannon (1♂, USNM).

***Paratypes*: USA. VA**: Fairfax Co., Turkey Run Park, nr. mouth of Turkey Run, 38°57.9'N, 7°09.4'W, Malaise trap, D.R. Smith, 18–30.v.2007 (1♂ 2♀, USNM; 2♀, CNC), 14–17.v.2006 (8♀, USNM; 2♂, CNC).

###### Comments.

The straight, highly simplified distiphallus of this species is characteristic, as is the basally inserted mesophallus, the large, pale ejaculatory apodeme, and narrow gena. Also see comments for *Ophiomyiaabutilivora*.

##### Ophiomyia kwansonis

Taxon classificationAnimaliaDipteraAgromyzidae

Sasakawa

Figs 356–359

Ophiomyia kwansonis Sasakawa, 1961: 355. Shiao and Wu 1999: 344; Matsumoto and Sasakawa 2006: 20; Williams and Steck 2011: 7, 2014: 421; Shi, Chunyan and Gao 2015: 61.

###### Description.

Wing length 2.0–2.5 mm (♂♀). Length of ultimate section of vein M4 divided by penultimate section: 0.8–1.0. Eye height divided by gena height: 4.0–6.0. Facial carina distinct; bulb narrow, slightly wider than carina, with medial longitudinal furrow and dorsal 1/2 shiny. Clypeus strongly narrowed anteriorly. Anterior portion of buccal cavity narrowed, not much wider than clypeus. Gena slightly produced anteriorly, forming an ~ 80° angle. Notum subshiny. Ocellar triangle with sides convex, nearly meeting past midpoint and attaining anterior margin of frons. Fronto-orbital plate narrow and shiny, slightly widening around base of fronto-orbitals.

***Chaetotaxy***: Male vibrissal fasciculus thick, slightly upcurved and not longer than gena. Two ori; two ors. Mid tibia with small posteromedial seta past midpoint.

***Colouration***: Body, including halter dark brown to black, with nearly indistinct metallic sheen on abdomen, and with fronto-orbital plate and ocellar triangle paler. Wing veins brown. Calypter margin and hairs grey.

***Genitalia***: (Figs 356–359) Surstylus narrow with several scattered rows of tubercle-like setae at apex and inner-distal surface. Metepiphallus with single pair of posteromedial spines. Basiphallus sclerotised basally on dorsobasal and left lateral surfaces, and with long right-lateral process that is widened at apex and attaining level of base of distiphallus. Distiphallus with blunt subconical base that extends posteriorly past narrow mesophallus and is internally spinulose on ventral and left ventrolateral surfaces; widest near midpoint, distally with spinulose right lateral chamber and thin distomedial chamber with sclerotised ventral surface and irregular internal folds. Ejaculatory apodeme relatively narrow with distal margin of blade reduced, pale and irregular.

###### Host.

Asphodelaceae – *Hemerocallisfulva*.

###### Distribution.

**USA**: AL, AR, CA, CT, DE, FL, GA, IL, IN, KA, KY, LA, MA, MD, MI, MO, MS, NC, NH, NY, OH, OK, PA, SC, TN, TX, VA, WI, WV. Japan, Taiwan. Slovenia.

###### Type material.

***Holotype*: Japan.** Honshu: Kyoto, Shimogamo, on *Hemerocallisfulvakwanso*, 21.v.1956, Sasakawa (1♂, Osaka Museum of Natural History). [Not examined]

###### Additional material examined.

**USA. MD**: Anne Arundel Co., Davidsonville, 7.vii.2011, on *Hemerocallis* sp., G.L. Williams (1♂ 11♀ [in alcohol], CNC; 1♂ 1♀, CSCA).

###### Comments.

Originally described from Japan as a leaf miner in the ornamental daylily *Hemerocallisfulvakwanso*, this fly is a recent invasive in North America (Williams and Steck 2011, 2014), and will soon likely occur in most regions where daylily is grown. Life history and immature stages are discussed in Sasakawa (1961) and Williams and Steck (2011). The only other agromyzid known to occur on *Hemerocallis* is *Liriomyzahemerocallis* Iwasaki, also from Japan, but this species feeds on seeds, not within leaves (Iwasaki 1993).

##### Ophiomyia labiatarum

Taxon classificationAnimaliaDipteraAgromyzidae

Hering

Figs 78–80 , 360–366

Ophiomyia labiatarum Hering, 1937: 509. Spencer 1969: 87, 1976: 67; Spencer and Steyskal 1986b: 51; Černý 2018: 122.

###### Description

**(Figs 78–80).** Wing length 2.1–2.4 mm (♂), 2.4–2.5 mm (♀). Length of ultimate section of vein M4 divided by penultimate section: 0.7–1.0. Eye height divided by gena height: 8.0–8.4 (4.0 in one dissected VA male from Turkey Run). Facial carina and medial bulb distinct; carina as wide as bulb dorsally or slightly narrower, with sides usually parallel and never strongly diverging; bulb usually strongly to shallowly furrowed or heart-shaped. Clypeus narrowed and produced anteriorly. Anterior margin of buccal cavity narrowed and straight or rounded. Eye slightly angled diagonally. Gena slightly produced, forming an angle slightly < 90°. Ocellar triangle and fronto-orbital plate subshiny. Notum shiny.

***Chaetotaxy***: Male vibrissal fasciculus distinct, nearly as long as gena, but only forming a discrete point in male with three ori on both sides. Two ori (sometimes three on one side, rarely on both sides); two ors. Mid tibia with one posteromedial seta.

***Colouration***: Body, including halter dark brown. Abdomen sometimes with faint coppery/golden shine. Wing veins brown. Calypter margin and hairs dark brown.

***Genitalia***: (Figs 360–366) Metepiphallus with one pair of ventromedial spines. Ventral subapical membrane around phallus pronounced and keel-like (less developed in male with three ori on both sides). Basiphallus with sclerotised dorsobasal plate and right lateral band. Base of mesophallus and distiphallus level. Distiphallus with small transverse sclerite at base of mesophallus; distiphallus subovate (slightly flattened apically) with inner basal margin spinulose and inner distal margin with shallow bumps; apex ill-defined.

###### Hosts.

Lamiaceae – *Calamintha*, *Clinopodium*, *Galeopsis*, *Lamium*, *Leonurus*, *Nepeta*, *Prunella*, *Salvia*, *Satureja*, *Scutellaria*, *Stachys* (Spencer 1976; Spencer and Steyskal 1986b; Benavent-Corai et al. 2005). Asteraceae – Solidagocanadensis*.

###### Distribution.

**Canada**: AB, NB*, ON, QC. **USA**: DE*, IN, MD*, PA*, VA*. Europe, Cyprus, Egypt, Israel, Turkey (Černý, 2018).

###### Type material.

***Lectotype*: Germany.** Meckelenburg (1♂, ZMHU). [Not examined]

###### Material examined.

**Canada. AB**: Wabamun, caught on Solidago, 1–3.vii.1966, K.A. Spencer, CNC358537 (1♂, CNC), **NB**: Kouchibouguac N.P., 13.vii.1977, J.F. McAlpine, IDEMA illustration, “L. Yuzyk July 1981”, code-6042J, CNC358542–358544 (2♂,1♀, CNC), 23.v.1977, Hanley and Cooper, code-5113Q, CNC358545 (1♀, CNC), 9.vii.1977, J.F. McAlpine, code-6023Q, CNC358546 (1♂, CNC), **ON**: Midland, swampy woods, balsam poplar, 2.v.1959, J.G. Chillcott, CNC358551 (1♂, CNC), Ottawa, 22.vii.1954, W.R.M. Mason, CNC358550, CNC358552 (1♂ 1♀, CNC), St. Lawrence Is. Nat. Park, Thwartway Is., 15.vii.1976, A. Carter, code 4118-J, CNC358547 (1♂, CNC), 21.vii.1976, W. Reid, code 4174-N, CNC358549 (1♀, CNC), 24.vii.1976, W. Reid, code 4198-L, CNC358548 (1♀, CNC), **QC**: Beech Grove, 23.vi.1951, J.F. McAlpine, CNC358539, CNC358540 (2♂, CNC), Harrington Lk., Gatineau Pk., 7.vi.1954, E.E. Sterns, CNC358541 (1♂, CNC). **France.** [Lower Normandy]: Verson b. Caen, 4880, 9.viii.1942, Dr. H. Buhr. Nr., mine an Stachys recta CNC358538 (1♂, CNC). **USA. DE**: Newark, spring 1974, R.W. Rust (1♂, USNM), **IN**: Lafayette, 6.vii.1915, reared from catnip, issued13.vii.1915, Satterthwalt (1♂, USNM), Lafayette, swept from grass, “v-17”, J.M. Aldrich (1♂, USNM), Lafayette, “iv-6”, swept from wint. wheat (1♂, USNM), **MD**: Kent Is., “35-13-4”, Seek and Nixon (1♂, USNM), College Park, 28.v.1939, C.T. Greene (1♂, USM), Beltsville, B.H. Braun, i.1972 (1♂, USNM), i.1972, ex. *Solidago* stems (2♂, USNM), i.1972, reared ex. *Eurostasolidaginis* gall (1♂ 2♀, USNM), Beltsville, 12.iv.1972, em. Indoors, B.H. Braun, ex. stem *Solidagocanadensis* (1♂ 1♀, USNM), Montgomery Co., Clarksburg, Little Bennett Reg. Park, 21.ix.1990, W.E. Steiner, M.J. and R. Molineaux (1♂, USNM), **PA**: Kimblesville, spring 1974, R.W. Rust (1♂, USNM), **VA**: Fairfax Co., Turkey Run Park, nr. mouth of Turkey Run, 38°57.9'N, 7°09.4'W, Malaise trap, 18–30.v.2007, D.R. Smith (1♂, USNM).

###### Comments.

*Ophiomyialabiatarum*, a polyphagous Lamiaceae feeder also found on *Solidago* with a relatively northern distribution, is somewhat variable with regards to head morphology (although the facial bulb is always shiny dorsally), but the male phallus can be readily diagnosed.

##### Ophiomyia laticolis sp. nov.

Taxon classificationAnimaliaDipteraAgromyzidae

http://zoobank.org/F2AB2C7F-6930-49F5-86E5-8400492F4887

Figs 367–371

###### Description.

Wing length 2.2–2.3 mm (♂). Female unknown. Length of ultimate section of vein M4 divided by penultimate section: 0.7–0.8. Eye height divided by gena height: 9.0 (non type). Eye angled diagonally. Facial carina thin along length with narrow medial bulb. Gena shallowly produced, forming a 45° angle. Clypeus produced and abruptly narrowed apically with apex truncated. Buccal cavity narrowed anteriorly with anterior margin nearly straight. Distance between crossveins more than length of dm-m. Ocellar triangle and fronto-orbital plate (narrow) subshiny. Head damaged.

***Chaetotaxy***: Male with vibrissal fasciculus ~ 1/2 length of gena. Two ori (slightly thinner); two ors. Mid tibia with one weak posteromedial seta.

***Colouration***: Gena relatively high, flat and pale in holotype. Body, including halter dark brown with frons darker. Wing veins brown. Calypter margin and hairs brown.

***Genitalia***: (Figs 367–371) Epandrium broad, rounded, narrower in lateral view and slightly shorter to dorsum. Surstylus fused to epandrium, 1/2 length of distal region of epandrium; apex narrower, rounded; inner surface with two scattered rows of tubercle-like setae. Metepiphallus small and pale with one pair of ventromedial spines. Halves of basiphallus broadly fused at base; left sclerite short, weakly sclerotised and ill-defined, and right sclerite longer, truncated and with slight dextral curve. Distiphallus strongly spinulose on inner surface but with few other internal elaborations; slightly flattened and subquadrate in ventral view.

###### Host.

Unknown.

###### Distribution.

**USA**: MD, VA.

###### Etymology.

The specific name compounds the Latin for wide (*latus*) and penis (*colis*).

###### Type material.

***Holotype*: USA. MD**: Montgomery Co., Bethseda, 28.iv.1968, G. Steyskal (1♂, USNM),.

***Paratype*: USA. VA**: Fairfax Co., Turkey Run Park, 0.3 km W mouth Turkey Run, 38°58'N, 77°09.6'W, Malaise trap, river trap, 17–24.v.2006, D.R. Smith (1♂, USNM).

###### Comments.

The new species *Ophiomyialaticolis* is highly similar to *O.ambrosia* Spencer from California (Spencer and Steyskal 1986b: figs 232–234), but the genitalia appear to be slightly smaller, the base of the mesophallus is nearly level with the base of the distiphallus, and the sides are severely flared, almost forming one pair of lateral points. Externally, the gena of *O.ambrosia* is slightly flatter and paler, but this may be an artifact of preservation; the eye is 5.6 × higher than the gena, and wing length is similar (2.2 mm).

The genitalia of this species also closely resemble those of *Ophiomyiaduodecima* Spencer (Quebec; Spencer, 1969: figs 134–136), in that the distiphallus is subquadrate in ventral view. The distiphallus of this Canadian species, however, is 2 × as large, the mesophallus is small and gracile, the left sclerite of the basiphallus is larger and not basally fused to the right sclerite (a possible artifact of illustration) and the gena is much higher (1/4 eye height).

##### Ophiomyia maura

Taxon classificationAnimaliaDipteraAgromyzidae

(Meigen)

Figs 372–376

Agromyza maura Meigen, 1838: 7.
Agromyza curvipalpis Zetterstedt. Misidentification, in part. Frost 1924: 41.
Ophiomyia bicornis Kaltenbach, 1869: 195; Hendel 1920: 130 [as synonym of curvipalpis], 1931: 188 (as synonym maura).
Agromyza affinis Malloch, 1913: 317; Hendel 1920: 130 [as synonym of curvipalpis], 1931: 188 (as synonym of maura).
Ophiomyia maura . Hendel 1920: 130 [as synonym of curvipalpis], 1931: 188 [also includes O.pulicaria Braschnikow, A.curvipalpis and Agromyzatexana Frost as synonyms]; Frick 1952: 382, 1959: 370; Sasakawa 1961: 358; Spencer 1969: 89; Papp and Černý 2015: 323; Scheffer and Lonsdale 2018: 86; Eiseman and Lonsdale 2018: 21.
Ophiomyia curvipalpis . Hendel 1920: 130.
Ophiomyia asteris Kuroda, 1954: 82. Sasakawa 1961 [synonymy].

###### Description.

Wing length 1.8–2.0 mm (♂) 2.0–2.1 mm (♀). Length of ultimate section of vein M4 divided by penultimate section: 0.7–1.1. Eye height divided by gena height: 6.2–7.8. Eye slightly oblique, but still relatively round. Arista short pubescent. Ocellar triangle nearly reaching level of posterior ori. Ocellar plate narrow. Facial carina well-developed, medially with smooth, subshiny, ovate bulb; carina above bulb as wide as bulb, with shallow medial groove continuing onto lunule; lunule height subequal to width of carina dorsally. Genal process nearly as long as high, forming an approximate 60° angle. Clypeus with arms bowed inwards anteriorly, meeting at small subquadrate anteromedial extension that is shallowly concave anteriorly and with one pair of minute anterolateral points. Crossveins separated by length of dm-m or slightly less.

***Chaetotaxy***: Male vibrissal fasciculus thick and upcurved, ~ 2/3 length of gena. Two ori (sometimes one additional ori present on one side); two ors. Two dorsocentral setae, anterior seta ~ 4/5 length of posterior. Acrostichal setulae in six scattered rows. Mid tibia with one or no posteromedial setae.

***Colouration***: Setae black. Body dark brown, including halter. Calypter, including margin, light brown; hairs dark brown; ocellar triangle and ocellar plate paler brown.

***Genitalia***: (Figs 372–376) Metepiphallus pale and narrow with one pair of ventromedial spines. Epiphallic lobes clear with dark sclerite. Epandrium shallow, venter slightly curved anteriorly, fused to surstylus. Surstylus small, short, subtriangular, with tubercle-like setae in three rows that narrow to one anteriorly. Hypandrium subtriangular with pointed apex and bowed basal arms. Phallophorus with narrower, darker base, short dorsum, and higher, longer venter; seen ventrally, venter with one pair of dark ridges. Basiphallus with weak, short left lateral arm connected basally to longer, better-defined right lateral arm that does not reach level of mesophallus. Mesophallus narrow, cylindrical, inserted ventromedially into larger distiphallus; base of mesophallus approximately level with base of distiphallus. Base of distiphallus (overlapping mesophallus) with broad, laterally bulging anterior section that abruptly narrows to a small rounded point apically; basal section with minute divots towards apex and several small internal spinules in right anterolateral section; basal section with broad opening dorsally bearing characteristic large flared membranous fold that encompasses broad, curved, strongly produced plate; distal section of distiphallus with distinct sclerotised medial tubule that is flanked ventrally by one pair of weakly differentiated and minutely textured hemispheres. Ejaculatory apodeme with short, narrow stem emerging from wide asymmetrical base that continues onto pale, irregularly sclerotised blade that is atrophied to one side; sperm pump with dark transverse bar ventrally.

###### Host.

Asteraceae – *Aster*, *Callistephus*, *Doellingeria* (?), *Erigeron*, *Eurybia*, *Euthamia*, *Oclemena*, *Solidago*, *Symphyotrichum* (Benavent-Corai et al. 2005; Eiseman and Lonsdale 2018; Ellis 2021). Possibly *Eupatorium* (Spencer 1990).

###### Distribution.

**Canada**: NB*, QC*. **USA**: CT, DE*, MA, MD*, ME, NY, VA*, VT; possibly also CO, MN, OH (Eiseman and Lonsdale 2018). Europe. Northeastern China. Turkey. Japan. Canary Islands. Oman. Yemen

###### Type material.

***Holotype* [*affinis*]: USA. MD**: Glen Echo, 3.vi.1898, R.P. Currie (1♀, USNM).

***Holotype* [*asteris*]: Japan.** Honshu: Kantō, Yokohama, 24.v.1938, leaf miner in *Asterindicus* L., M. Kuroda (1♀, BLTJ).

***Syntypes* [*bicornis*]: Germany.** [not given]. (Type data unknown)

***Syntypes* [*maura*]: Germany.** [not given]. (Types lost).

###### Additional material examined.

**Estonia.** Tallinn, 18.vii.1995, M. v.Tschirnhaus, ex *Solidago* (1♂, CNC). **Canada. NB**: Kouchibouguac National Park, 46°48'49.90"N, 64°55'40.02"W, 22.v.1977, Hanley and Cooper, Code – 5111O, CNC758931 (1♀, CNC), 23.v.1977, Code – 5113Q, CNC758930 (1♂, CNC), **QC**: Old Chelsea, 45°30'0.40"N, 75°48'52.80"W, 30.v.1952, J.F. McAlpine, CNC758932 (1♂, CNC). **USA. CT**: Litchfield Co., Canaan, 21.vii.2015, em. 29.vii.2015, C. Vispo, ex *Solidagocanadensis*, #CSE2168, CNC564684 (1♀, CNC), **DE**: Newark, 1.vii.1974 (1♂, USNM), **MA**: Berkshire Co., Lenox, Parsons Marsh, 20.vi.2017, em. 24.vi.2017, C.S. Eiseman, ex *Solidagopatula*, #CSE3853, CNC939713 (1♂, CNC), Franklin Co., Montague, Montague Plains Wildlife Management Area, 8.vi.2017, em. 21.vi.2017, C.S. Eiseman, ex *Solidagoarguta*, #CSE3843, CNC939720 (1♂, CNC), Northfield, 276 Old Wendell Rd., 10.x.2016, em. 19.iv.2017, C.S. Eiseman, ex *Eurybiadivaricata*, #CSE3524, CNC939673 (1♂, CNC), 2.vii.2017, em. 15.vii.2017, ex *Euthamiagraminifolia*, #CSE3956, CNC939661 (1♂, CNC), Hampshire Co., Pelham, Butter Hill, 21.vi.2013, em. 24.vi.2013, C.S. Eiseman, ex *Solidagocaesia*, #CSE591 (2♀, CNC), South Hadley, near Lithia Springs Reservoir, 11.v.2016, em. 2.vi.2016, C.S. Eiseman, ex *Solidagoarguta*, #CSE2536 (1♀, CNC), Middlesex Co., Shirley, 42.556117, -71.613381, 3.viii.2017, em. 9.viii.2017, C.S. Eiseman, ex *Solidagogigantea*, #CSE4095, CNC939727, CNC9397278 (1♂ 1♀, CNC), Nantucket Co., Nantucket, Gardner Farm, 13.vi.2013, em. 24.vi.2013, C.S. Eiseman, ex *Solidagolatissimifolia*, #CSE600 (1♀, CNC), Nantucket, Squam Swamp, 12.vi.2013, em. 26–30.vi.2013, C.S. Eiseman, ex *Solidagolatissimifolia*, #CSE609, CNC384817, CNC384818 (1♂ 1♀, CNC), **ME**: Knox Co., Camden, Bald Mountain, 5.x.2013, C.S. Eiseman, ex. *Oclemenaacuminata* em. 20.iii.2014, #CSE1011, CNC384784 (1♂, CNC), **VA**: Montgomery Co., Christiansburg, 2.vi.1962, J.G. Chillcott (1♂, CNC), **VT**: Chittenden Co., South Burlington, Winooski Gorge, 29.vi.2014, ex. *Solidagoflexicaulis*, em. 10.vii.2014, C.S. Eiseman, #CSE1167, CNC384885 (1♂, CNC).

###### Comments.

While relatively indistinct externally, the male genitalia of *Ophiomyiamaura* are more diagnostic: the epiphallic lobes are clear with a dark basal sclerite; the distiphallus is relatively large and well-sclerotised with a dark anterodorsal tubule, it has a transverse dorsomedial “shelf” that is only slightly curved, and there are two minutely tuberculate distoventral hemispheres; the phallophorus has two sclerotised ridges that are obvious when viewed ventrally. It is quite similar in North America to *O.carolinensis* Spencer, *O.parda* Eiseman and Lonsdale and *O.quinta* Spencer, all of which now have small indicators on the distiphallus that reveal identity: *O.carolinensis* has a small basal protuberance on the distiphallus (Spencer and Steyskal 1986b: figs 260, 261) and a darker, broader, more distinct anterodorsal process emerging from the distiphallus, and *O.parda* and *O.quinta* have an inner-medial plate projecting dorsally that is long and more strongly curved (Eiseman and Lonsdale 2018: figs 254, 255). Also see discussion in Eiseman and Lonsdale (2018).

##### Ophiomyia nasuta

Taxon classificationAnimaliaDipteraAgromyzidae

(Melander)

Figs 64–66 , 377–382

Agromyza curvipalpis Zetterstedt. Misidentification, in part. Melader 1913: 251.
Agromyza maura var. nasuta Melander, 1913: 260.
Agromyza youngi Malloch, 1914: 312. Frick 1952a: 384 [as synonym of pinguis Fallén]. Frick (1959) [as synonym of *nasuta* Melander].
Ophiomyia madizina Hendel, 1920: 130. Spencer 1964a [synonymy].
Tylomyza madizina . Hendel 1931: 185. Frick 1952a: 385, 1959: 372
Siridomyza madizina . Enderlein 1936a: 179.
Tylomyza nasuta . Frick, 1952a: 384 [as synonym of pinguis Fallén], 1957: 201 [lectotype designation], 1959: 372.
Ophiomyia nasuta . Spencer, 1964: 789, 1969: 91, 1976: 71, 1990: 261; Spencer and Steyskal 1986b: 42; Scheffer et al. 2007: 770; Černý 2018: 122; Eiseman and Lonsdale 2018: 23.

###### Description

**Figures 64–68.**
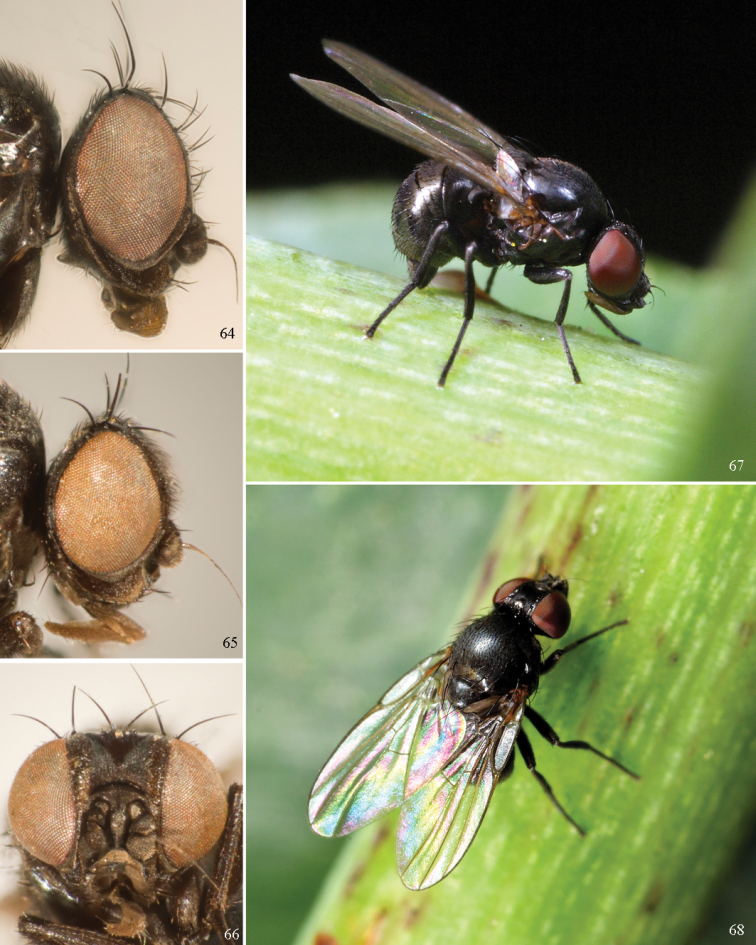
*Ophiomyianasuta* (Melander), head **64** female, lateral **65** male paralectotype, lateral **66** male paralectotype, anterior **67, 68***Ophiomyia* sp. female on *Lactucavirosa* (S. Justis – Virginia, Norfolk).

**(Figs 64–66, 377).** Wing length 2.0–2.2 mm (♂), 2.1–2.5 mm (♀). Length of ultimate section of vein M4 divided by penultimate section: 0.8–1.0. Eye height divided by gena height: 5.4–7.4. Face short, with carina broad and diverging above large, semispherical bulb. Gena stout and produced anteriorly; highest subapically. Clypeus slightly narrowed anteriorly (more pronounced in female). Anterior margin of buccal cavity broad. Anterior angle of ocellar triangle long and tapered, nearly reaching anterior margin of frons. Frons minutely pitted with ocellar triangle and fronto-orbital plate shiny. Fronto-orbital plate and parafacial slightly projecting and continuing under eye as distinct cheek. Body shiny. Distance between crossveins approximately as long as dm-m.

***Chaetotaxy***: Orbital setulae proclinate and relatively long and dense. Male vibrissal fasciculus absent. Two ori; one or two ors present in female, absent in male. Three dorsocentral setae, slightly decreasing in length anteriorly. Mid tibia with one small posteromedial seta.

***Colouration***: Body, including halter dark brown to black. Calypter margin and hairs dark brown. Wing veins light brown to brown, sometimes whitish.

***Genitalia***: (Figs 378–382) Surstylus 1/2-length of epandrium at base, narrowing apically with apex broadly rounded; height approximately equal to length at base; with irregular clusters of tubercle-like setae apically and anteriorly on inner surface. Metepiphallus small with three pairs of medial and basal spines. Phallophorus slightly elongate with constricted base. Halves of basiphallus broadly fused at base with short, ill-defined left lateral sclerite and slightly longer right lateral sclerite. Distiphallus with base extending past base of mesophallus; minutely tuberculate dorsally and apically, inner surface minutely spinulose laterally near base, with small dorsomedial hook and distal section divided into two hemispheres. Ejaculatory apodeme with well-developed stalk, blade with striations and base with thin membranous tubule.

###### Host.

Asteraceae – *Taraxacumofficinale*.

###### Distribution.

Across northern North America from YT to QC, south to northern CA, CO, NM and NC (Eiseman and Lonsdale 2018). Europe, Japan, Turkey, Kazakhstan (Černý 2018).

###### Type material.

***Lectotype* [*nasuta*]: USA. WA**: Kamiac Butte, 1.vi.1912 (1♂, USNM).

***Paralectotypes* [*nasuta*]: USA. ID**: Troy, 14.vi.1908 (1♂, USNM), **WA**: Pullman (4♂, USNM). **Austria.**“Styria” (1♂, USNM).

***Holotype* [*youngi*]: USA. NY**: Albany, 28.iv.1913, D.B. Young (1♂, NYSM). [Not examined].

***Syntypes* [*madizina***]: “Austr., Germ.” (23♂♀, NMW, USNM). [USNM ♂ and ♀ examined].

###### Additional material examined.

**Canada. BC**: nr. Golden Rest Area, rt. 1, 20.vi.2001, C.R. Bartlett (1♀, UDCC), **ON**: Ottawa, 45°19'1.20"N, 75°43'12"W, 90 m, 7.vi.2016, J.E. O’Hara, Malaise trap, CNC629668, CNC629688 (2♀, CNC), **SK**: Duck Lake, 15.iv.1924, K.M. King, CNC358553 (1♀, CNC). **USA. CT**: Redding, 10.v.1930, A.L. Melander (2♀, USNM), **DE**: Pike Creek, 25.vi.1975, D. Buntin (1♀, UDCC), Bridgeville, 8.viii.195, H.E. Milliron, on peppers (1♀, UDCC), New Castle Co., Newark, Thorn Lane beside RR tracks, 18.vii.1993, field sweep, D.S. Chang (1♀, UDCC), UD Newark Farm, 39°40'14.81"N, 75°44'54.89"W, 18.ix.2007, sweep net, M. Frye (1♀, UDCC), Newark, UD Farm, 5.x.1997, W.P. Brown, sweep net (1♀, UDCC), Newark, UofD Gardens, 3.viii.2007, T. Cooper (1♀, UDCC), Sussex Co., Millville, Route 26, 5.x.1997, M.J. Harrison (1♀, UDCC), **ID**: Lafayette, 30.iv.1915, swept from grass, J.M. Aldrich (1♀, USNM), **IL**: Chicago, A.L. Melander (1♀, USNM), **MA**: Franklin Co., Northfield, 276 Old Wendell Rd., 5.v.2016, C.S. Eiseman, *Taraxacumofficinale*, 20–21.v.2016 , #CSE2476, CNC654194 (1♂, CNC), **MD**: Temple Hills, 17.vii.1978, G.F. Hevel (1♀, USNM), Lavale, 9.v.1970, G. Steyskal (1♂, USNM), Montgomery Co., 4mi SW of Ashton, G.F. and J.F. Hevel, 18.iv.1987 (1♀, USNM), 25.iv.1987 (1♀, USNM), 15.viii.1982 (1♂ 2♀, USNM), 17.vii.1978 (1♂, USNM), 18.iv.1987 (1♂, USNM), Colesville, 31.vii.1975, W.W. Wirth (2♂, USNM), Colesville, 31.viii.1973, Malaise trap, W.W. Wirth (1♂ 2♀, USNM), P.G. Co., Temple Hills, 17.vii.1978, G.F. Hevel (2♂, USNM), Camp Springs, 8.vii.1979, G.F. Hevel (2♀, USNM), Oxon Hill, G.F. and S. Hevel, 22.vii.1978 (1♀, USNM), 24.vii.1978 (1♀, USNM), Frederick Co., 1mi N, 4.7mi W of Point of Rocks, 11.vii.1982, G.F. and J.F. Hevel (1♀, USNM), **MI**: Chippewa Co., 3.vi.1937, R.andK. Dreisbach (1♀, USNM), Ingham Co., E Lansing, 22.vii.1963, F.E. Giles (1♀, USNM), E. Lansing, C. Sabrosky, 3.vi.1937 (1♀, USNM), 29.v.1937 (1♀, USNM), **NM**: Cloudcroft, 26.v.1964, J.F. McAlpine, CNC358586 (1♂, CNC), **NY**: Ithaca, A.L. Melander, 28.v.1937 (1♀, USNM), 31.v.1913 (1♀, USNM), White Plains, 1.v.1909, Bueno, A.L. Melander (1♀, USNM), Geneva, 28.v.1914, A.L. Melander (1♂ 5♀, USNM), Chazy, 8.viii.1930, A.L. Melander (1♀, USNM), **OH**: Oxford, Mallot’s Lawn, 12.x.1978, B.A. Steinly (1♀, USNM), **PA**: Chester Co., Toughkenamon, 39°51'37, 75°46'58, 14.ix.2007, E. Lake Stroud Res. Ctr. (1♀, UDCC), Bucks Co., Pineville, 24.v.1969, J.W. Adams (1♀, USNM), **TN**: Gt. Smokies N.P., Chimneys, 20.vi.1941, A.L. Melander (1♀, USNM), Sevier Co., Rte. 441, 3mi NW NC/TN border, Great Smokey Mt. N.P., 35°38.3'N, 83°27.8'W, 4500', S.D. Gaimari, 27.v.1999 (1♀, USNM), **VA**: Pulaski, 7.v.1979, G. Steyskal (4♂ 1♀, USNM), Montgomery Co., Mill Cr., Rt. 785 NE Blacksburg, 30.iv.1978, C.M. and O.S. Flint (1♀, USNM), Giles Co., 10 km NW Blacksburg, Bald Knob, 18.v.1997, S.A. Marshall (1♂, DEBU), Butt Mtn., 8 km NW Blacksburg, 21.v.2005, S.A. Marshall (1♂, DEBU).

###### Comments.

*Ophiomyianasuta* is a widespread and relatively commonly encountered species that can be diagnosed quite readily using external characters: the facial carina is short with the bulb large and semispherical, the gena apically produced and truncated, there is no male fasciculus, there are three dorsocentral setae, the ocellar setulae are dense and proclinate, and there is sexual dimorphism in number of ors.

##### Ophiomyia sexta

Taxon classificationAnimaliaDipteraAgromyzidae

Spencer

Figs 383–387

Ophiomyia sexta Spencer, 1969: 98. Spencer and Steyskal 1986b: 257.

###### Description.

Wing length 2.1–2.4 mm (♂), 2.5 mm (♀). Length of ultimate section of vein M4 divided by penultimate section: 0.8–0.9. Eye height divided by gena height: 2.5–4.2. Facial carina stout with sides diverging above, thin below; bulb well-developed, sometimes with shallow medial furrow. Gena shallowly produced, forming 70°–80° angle. Clypeus produced and strongly narrowed apically. Buccal cavity narrowed anteriorly with anterior margin small and straight. Distance between crossveins as long as dm-m or less. Ocellar triangle and fronto-orbital plate shiny. Excluding VA specimen, fronto-orbital plate and parafacial strongly projecting.

***Chaetotaxy***: Male with vibrissal fasciculus ~ 1/2 length of gena. Three ori; two ors; sometimes only one ors or with two ori on one side only. Mid tibia with one weak distomedial seta.

***Colouration***: Body, including halter dark brown, but sometimes portions of ocellar triangle and fronto-orbital plate paler. Wing veins brown. Calypter margin and hairs brown.

***Genitalia***: (Figs 383–387) Metepiphallus small with one pair of ventromedial spines and several free basal sclerites. Epiphallic lobes with pale basal pigmentation. Halves of basiphallus fused at base, with left sclerite shorter, pale, and ill-defined, and right sclerite well-developed. Distiphallus extremely wide, bilobed and ridged, resembling a brain in ventral view; base high, and distal ridged portion strongly flattened with pale dorsomedial process. Ejaculatory apodeme with stalk short, blade pale and subovate with strong medial rib.

###### Host.

Unknown.

###### Distribution.

**Canada**: AB, MB, QC, NT. **USA**: CO, IL*, MS*, VA*.

###### Type material.

***Holotype*: Canada. AB**: Cypress Hills, 25.vi.1966, K. Spencer (1♂, CNC).

###### Paratypes examined.

**Canada: MB**: 2mi W Stockton, spruce-sand community, 20.v.1958, J.F. McAlpine (1♂, CNC), **NT**: Hay River, 15.vii.1959, P.R. Erlich (1♂, CNC), **QC**: Harrington Lake, Gatineau Park, 31.v.1954, E.E. Sterns (1♂, CNC), Kingsmere, 12.v.1958, J.G. Chillcott (1♀, CNC). **USA. CO**: Flagstaff Co., Boulder, 5800[ft], 10.vi.1961, C.H. Mann (1♂, CNC).

###### Additional material examined.

**USA. CO**: Boulder, Flagstaff Cn., 1767 m, 10.vi.1961, C.H. Mann, CNC358559 (1♂, CNC), **IL**: nr. Forest City, forest, 21.v.1953, J.F. McAlpine, CNC358560–358562 (3♂, CNC), **MS**: Winston Co., Noxubee Nat. Wildlife R., Tripletts, 33°16'N, 88°51'W, pasture road, 19.v.2013, J.M. Cumming, CNC358563 (1♂, CNC), **VA**: Veitch, 9.vi.1912, J.R. Malloch (1♂, USNM).

###### Comments.

Compared to other *Ophiomyia* with a fasciculus, *O.sexta* has a relatively short genal process, three ori and quite closely spaced crossveins. Internally, the incredibly large distiphallus is characteristic, being brain-shaped in ventral view and dorsoventrally compressed.

##### Ophiomyia simplex

Taxon classificationAnimaliaDipteraAgromyzidae

(Loew)

Figs 69 , 70 , 388–393

Agromyza simplex Loew, 1869: 46.
Melanagromyza simplex . Hendel 1920: 128; Frick 1952a: 379, 1959: 366.
Triopisopa simplex . Enderlein 1936a: 179.
Ophiomyia simplex . Spencer 1966a: 55, 1969: 98; Spencer and Steyskal 1986b: 39; Zlobin 2005b: 177.
Hexomyza simplex . von Tschirnhaus 2000: 113; Papp and Černý 2015: 180.

###### Description

**Figures 69–77.**
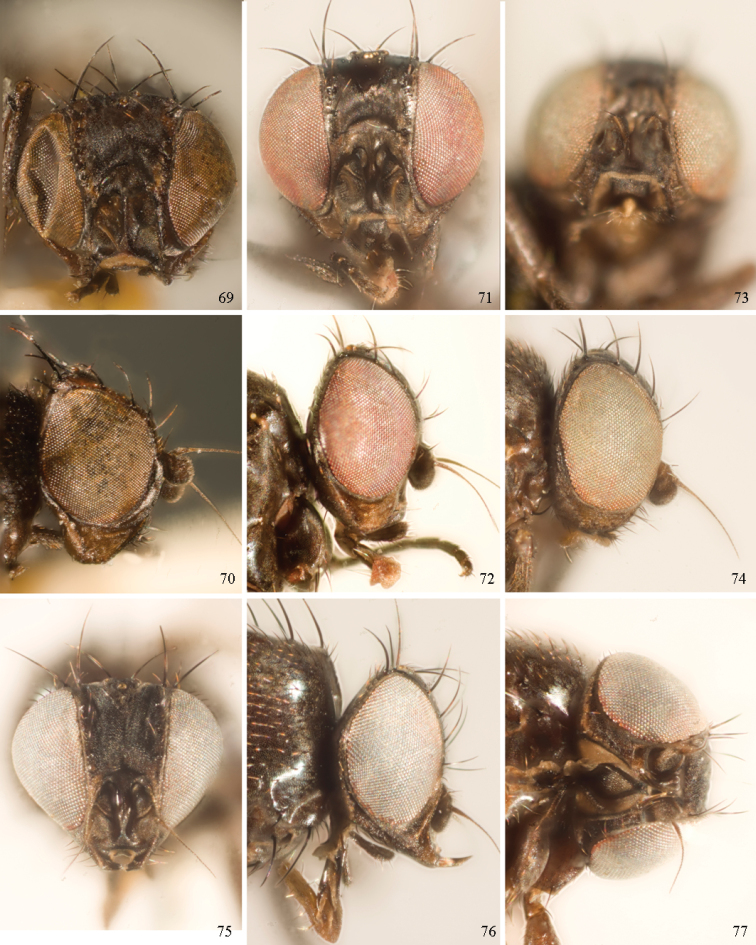
*Ophiomyiasimplex* (Loew), head **69** anterior **70** lateral **71, 72***Ophiomyiaabutilivora* Spencer, head **71** anterior **72** lateral **73, 74***Ophiomyiaultima* (Spencer), head **73** anterior **74** lateral **75–77***Ophiomyiaconiceps* (Malloch), head **75** anterior **76** lateral **77** ventral.

**(Figs 69, 70).** Wing length 2.1–2.4 mm (♂), 2.3–2.5 mm (♀). Length of ultimate section of vein M4 divided by penultimate section: 1.0–1.2. Eye height divided by gena height: 4.5–5.4. Facial carina thin and shallow with bulb absent. Gena strongly recessed anteriorly, highest behind midpoint of eye. Fronto-orbital plate and parafacial strongly projecting and continuing under eye as distinct cheek; parafacial shiny. Ocellar triangle shiny. Clypeus narrow with sides parallel to slightly bowed. Buccal cavity broad. Distance between crossveins narrow. Costa extending just past R_4+5_.

***Chaetotaxy***: Male vibrissal fasciculus absent. Two or three ori; two ors. Mid tibia with one posteromedial seta.

***Colouration***: Body, including halter dark brown and shiny. Calypter margin and hairs dark brown.

***Genitalia***: (Figs 388–393) Epandrium with shallow ventromedial point. Surstylus small and lobate/subtriangular, folded inwards just below midpoint of anterior margin of epandrium, and with scattered, marginal tubercle-like setae. Hypandrium relatively broad with sides parallel at base and arms meeting posteriorly; with stout apical process; additional circular sclerite absent. Metepiphallus short with one pair of basal spines and one pair of basolateral arms. Halves of basiphallus separate and broadly twisted dextrally. Mesophallus broad, inserted slightly off base of distiphallus towards its venter. Distiphallus cup-shaped with ventromedial suture continuing onto mesophallus; with one pair of ventral processes with inner margin shallowly spinulose. Ejaculatory apodeme asymmetrical and relatively thin distally, with dark, well-developed stem and medial rib.

###### Host.

Asparagaceae – *Asparagusofficinalis*. Adults have been collected on potato and beans.

###### Distribution.

**Canada**: BC*, ON, QC. **USA**: Widespread. Europe.

###### Type material.

***Holotype*: USA.** “Middle States” (1♂, destroyed).

###### Material examined.

**Germany.** Berlin, from C. Schirmer (1♂ ♀, USNM), Crossen a.O., 22.v.1932, Hering, Mine an *Asparagusofficinalis* No. 3871, CNC358570 (1♂, CNC). **Canada. BC**: Armstrong, collected from asparagus, 18.v.1976, 76-535, CNC358571 (1♂, CNC), **ON**: Ottawa, 25.v.1941, G. Matthewman, CNC358565–358569 (2♂ 3♀, CNC), Simcoe, 2.vi.1989, G.E. Shewell, CNC358564, CNC358572–358574 (2♂,2♀, CNC), Vineland, 20.vi.1937, G.E. Shewell, CNC358577 (1♀, CNC), **QC**: Abbotsford, 2.ix.1936, G.E. Shewell, CNC358576 (1♀, CNC), 24.vii.1936, CNC358575 (1♀, CNC). **USA. CA**: Lakeside, 4.iv.1944, *Asparagus* (1♂ 1♀, USNM), **DC**: “DC” (1♂, USNM), **DE**: Rising Sun, 12.v.1953, on asparagus, D. MacCreary (1♂, UDCC), Georgetown, 17.v.1955 (1♂ 2♀, UDCC), Stanton, 7.viii.1951 (1♂ 1♀, UDCC), **GA**: Tifton, 24.ix.1896, A.L. Melander (1? [head and abdomen missing], USNM), **IA**: Pleasant Valley, 29.vii.1931, H.M. Harris (1♀, USNM), **IL**: Chicago, A.L.Melander (1♂, USNM), **MD**: Cabin John, 10.v.1897 (2♂, USNM), Chillum, 6.iii.1914, collected on Asparagus (1♂, USNM), Colesville, 20.viii.1975, Malaise trap, W.W. Wirth (1♂, USNM), College Park, “6-29”, C.T. Greene (2♂, USNM), College Park, 10.viii.1914, collected on potato, W.H. White (1♂, USNM), College Park, “6-29”, C.T. Greene (1♀, USNM), **MI**: E Lansing, 1.vi.1929, on asparagus, R.W. Pettit (2♂ 1♀, USNM), Grand Rapids, 13.iv.1931, C.W. Sabrosky (1♂ 1♀, USNM), **NY**: Ithaca, A.L. Melander (2♂ 2♀, USNM), No. 2, Williamsville, Swp. 1, beans, 25.vii.1964 (1♂, UDCC).

###### Comments.

*Ophiomyiasimplex* is one of the few *Ophiomyia* with an anteriorly recessed gena, a strongly shiny and pronounced fronto-orbital plate and parafacial, a costa that extends only slightly past R_4+5_, a basiphallus composed of one pair of twisted bands and a bell-shaped distiphallus.

This species was transferred to *Hexomyza* by von Tschirnhaus (2000), which was rejected by Zlobin (2005), but again considered *Hexomyza* by Papp and Czerny (2015). The boundaries and definition of *Hexomyza* was considered by Lonsdale (2014), who found the genus to be polyphyletic and the type species embedded within *Ophiomyia*; as a result, *Hexomyza* was subsequently included as a junior synonym of *Ophiomyia*, and the new genus *Euhexomyza* Lonsdale was erected for the remaining related orphaned species. *Ophiomyiasimplex* appears to share none of the defining characters of *Euhexomyza*, except for the high lunule, pronounced orbital plate and parafacial and smaller posteriormost intra-alar, which commonly occur throughout the genus group, including *Ophiomyia*. There are no convincing adult or larval characters that would ally this stem miner with the gall-forming species previously considered *Hexomyza*, especially among the highly dissimilar male genitalia.

##### Ophiomyia texana

Taxon classificationAnimaliaDipteraAgromyzidae

(Malloch)

Figs 394–398

Agromyza texana Malloch, 1913: 319. Hendel 1931: 194 (as synonym of *proboscoidea* Strobl).
Ophiomyia texana . Frick 1952a: 383; Spencer and Steyskal 1986b: 259.
Ophiomyia shiloensis Spencer, 1969: 98. Spencer and Steyskal 1986b [synonymy].
Ophiomyia arguta Spencer in Spencer and Stegmaier 1973: 169. Spencer and Steyskal 1986b [synonymy].
Ophiomyia modesta Spencer, 1981: 94. Spencer and Steyskal 1986b [synonymy].

###### Description.

Wing length 1.8–1.9 mm (♂♀). Length of ultimate section of vein M4 divided by penultimate section: 0.8. Eye height divided by gena height: 11.0. Facial carina slender, distinct, with slender, smooth, matt facial bulb; carina not expanding immediately above bulb. Eye angled diagonally. Genal process with a broad, relatively short base approximating 60°, but apex much narrower, extending as a fine point. Clypeus strongly extended anteriorly as narrow process. Ocellar triangle with lateral margins straight to slightly concave, ending between ori and ors, but with sides produced as shallow grooves appearing sometimes to extend to lunule. Anterior margin of buccal cavity narrowed and straight. Notum shiny; bare between dorsocentrals. Crossveins separated by slightly more than length of dm-m.

***Chaetotaxy***: Male vibrissal fasciculus thick and upcurved, pointed, at least 2/3 length of gena. Two ori; two ors. Region between dorsocentrals smooth or with only a few anteromedial setulae. Mid tibia without posteromedial setae.

***Colouration***: Body, including halter dark brown. Gena and inner margin of fronto-orbital plate sometimes light brown. Wing veins dirty white to brown. Calypter margin and hairs dark brown.

***Genitalia***: (Figs 394–398) Surstylus narrow with few apical tubercle-like setae. Proepiphallus with lateral lobes clear with base lightly pigmented. Left sclerite of basiphallus very short, pale, and ill-defined apically, separate from right sclerite; right sclerite elongate, exceeding base of mesophallus. Base of mesophallus extending far past base of distiphallus excluding distinctive long basal tail-like process; distiphallus internally spinulose with paired ventral sclerotised ridges, subovate in ventral view and chevron-shaped in lateral view; with small dorsomedial spine. Ejaculatory apodeme with very large, pale blade past small flattened stem.

###### Host.

Brassicaceae – *Rorippa*, *Descurainia*. DE specimen collected on “spider bush”, likely as an adult.

###### Distribution.

**Canada**: MB. **USA**: CA, DE*, TX. Bahamas. Reported by Frick (1959) as widespread from MI to WA.

###### Type material.

***Holotype* [*texana*]: USA. TX**: Brownsville, “bred from *roripa*”, 27.i.1909, McMillan and Marsh (1♂, USNM; type No. 15582).

***Holotype* [*arguta*]: Bahamas.** Eleuthera Is.: Hatchet Bay, nr. Alicetown, 2.iv.1953, E.B. Hayden and L. Giovannoli (1♂, AMNH). [Not examined]

***Holotype* [*modesta*]: USA. CA**: Ventura Co., Point Mugu State park, 2.iv.1977, K.A. Spencer (1♂, CAS). [Not examined]

***Holotype* [*shiloensis*]: Canada. MB**: 5mi SW of Shilo, 22.vii.1958, J.G. Chillcott (1♂, CNC).

***Paratypes* [*shiloensis*]: Canada. MB**: Churchill, 3.ix.1945, R. Richards (1♂, CNC), Treesbank, 17.viii.1958, J.G. Chillcott (1♂, CNC).

###### Additional material examined.

**USA. DE**: Newark, 12.ix.1960, spider bush, D.F. Bray (1♂, USNM).

###### Comments.

Although *Ophiomyiatexana* can be partially recognised by the long upcurved fasciculus, a relatively small, slender, smooth, matt facial bulb and a scutum that is largely without medial setulae between the dorsocentral setae, examination of the male phallus is essential for confident identification. The distiphallus is chevron-shaped in profile, the inner surface is sparsely but evenly spinulose, and the base has a tail-like process.

The Delaware record presented here significantly expands the northeastern range of this widespread but uncommon taxon.

##### Ophiomyia tiliae

Taxon classificationAnimaliaDipteraAgromyzidae

(Couden)

Figs 399–404

Agromyza tiliae Couden, 1908: 35.
Melanagromyza tiliae . Frick 1952a: 380, 1957: 200 [lectotype designation].
Melanagromyza fastosa Spencer, 1969: 67. Syn. nov.
Hexomyza tiliae . Spencer 1973: 299.
Ophiomyia fastosa . Spencer and Steyskal 1986b: 255.
Ophiomyia tiliae . Spencer and Steyskal 1986b: 260.

###### Description.

Wing length 1.9–2.7 mm (♂), 2.7–3.3 mm (♀). Length of ultimate section of vein M4 divided by penultimate section: 0.6–0.8. Eye height divided by gena height: 2.5–4.3. Fronto-orbital plate varying from slightly visible laterally to relatively pronounced; parafacial sometimes produced, continuing under eye as broad cheek. Gena relatively high and shallowly rounded, only slightly angled anteriorly to produce shallow ventromedial angle. Facial carina indistinct to absent, but antennal bases always slightly separated; bulb slender, not strongly pronounced, usually appearing as slight swelling below antennal bases. Clypeus stout and parallel-sided. Ocellar triangle and fronto-orbital plate shiny. Crossveins narrowly separated.

***Chaetotaxy***: Male vibrissal fasciculus absent. Two to three ori (orbital setula between ori variably strengthened); two ors. Mid tibia with one posteromedial seta.

***Colouration***: Body, including halter dark brown with gena paler. Wing veins light brown. Calypter margin and hairs brown. Abdomen sometimes with very faint, almost indistinct metallic lustre – in NY male, tint greenish with tergites 1–3 bluish.

***Genitalia***: (Figs 399–404) Externally as described for *O.abutilivora*, excluding texturing on epandrium. Phallophorus strongly compressed laterally. Basiphallus ring-shaped and with adjoining dorsobasal membrane lightly sclerotised. Distiphallus subspherical with anterior and ventral margins thick; ventrally bordered by light transverse sclerite (upcurved and pointed at sides; highly faded in *tiliae* holotype, likely resulting from method of preservation as other specimens reared from *Tilia* with this sclerite moderately to well-developed), long thin membranous flagellum (pointed ventrally) and large carinate, membranous hypophallus.

***Variation***: VPIC male with clypeus appearing broadly rounded (actually bulbous medially), but anterior corners still present. Cheek only evident anteriorly. Two ori, closely spaced anteriorly. Anteroventral membrane on phallus reduced, with “wings” of distiphallus barely evident.

###### Host.

Malvaceae – *Tiliaamericana*, *Tilia* sp.

###### Distribution.

**Canada**: AB, ON*, QC. **USA**: CO, IL, IN, MA (Eiseman and Charney 2010), MO, NY*, PA, VA.

###### Type material.

***Lectotype* [*tiliae*]: USA. MO**: Jennings, 4.iv.1907, T.F. Hickey, ex. *Tiliaamericana* (1♀, USNM; type No. 10,028).

###### Paralectotypes examined

[***tiliae*]: USA. MO**: Jennings, Mrs. Hickey, coll., 2.iv.1907, iss. 10.iv.1907 (1♀, USNM), 2.iv.1907, iss. 4.iv.1907 (3♀, USNM), 2.iv.1907, iss. 6.iv.1907 (1♀, USNM), 22.iii.1907, iss. 10.iv.1907 (1♀, USNM), 22.iii.1907, iss. 7.iv.1907 (1♀, USNM).

***Holotype* [*fastosa*]: Canada. QC**: Hull, 24.v.1923, C.H. Curran (1♂, CNC).

###### Additional material examined.

**Canada. ON**: Brantford, 24.i.1978, “775-2306-01”, “Tilia R’rd” (1♂ 2♀, CNC), Col. 17.viii.1977, Host *Tiliaamericana*, feeding gall at base of petiole (1♂, CNC). **USA. CO**: State Bridge nr. Bond, 7000', 24-25.vi.1961, B.H. Poole (1♂, CNC), **NY**: Ithaca, 22.v.1950, J.C. Martin, CNC358463 (1♂, CNC), **VA**: Fairfax Co., Great Falls Park, quarry, 38°59.1'N, 77°14.8'W, Malaise trap, 24.iv-2.v.2007, D.R. Smith (1♂, USNM), Turkey Run Park, nr. mouth of Turkey Run, 38°57.9'N, 7°09.4'W, Malaise trap, D.R. Smith, 26.iv-2.v.2007 (1♂, USNM), 5.v-6.vi.2006 (1♂, USNM), Blacksburg, 5.v.1947, W.B. McIntosh (1♂, VPIC).

###### Comments.

*Ophiomyiatiliae* is much darker than similar species such as *O.abutilivora* and often has a much higher gena. An ovate distiphallus with a ventrally directed apical flagellum and bordered by a wide transverse sclerite are most diagnostic.

*Ophiomyiafastosa* syn. nov. is here included as a junior synonym of *O.tiliae*, which overlaps in wing length, gena height, colour and chaetotaxy. Morphology of the surstylus/epandrium are also similar, as is morphology of the phallus, with variations seen in previously published illustrations of the distiphallus being strongly exaggerated or not evident upon re-examination.

Male genitalic illustration of *Ophiomyiaparvella* Spencer (Spencer and Stegmaier 1973; Sasakawa 1994) almost exactly matches the genitalia of *O.tiliae*, and may also be conspecific. While the host of *O.parvella* is unknown, the host of *O.tiliae* (*Tiliaamericana*, American basswood is found in Florida (USDA 2008), where *O.parvella* was described from. *Ophiomyiashastensis* Spencer has highly similar genitalia, but apparently differs from *O.tiliae* in having a metallic abdomen; the metallic tint is evident in some specimens examined here, including those in the northeast, suggesting that the boundaries of this species require future re-examination.

##### Ophiomyia ultima

Taxon classificationAnimaliaDipteraAgromyzidae

(Spencer) comb. nov.

Figs 73 , 74 , 405–410

Melanagromyza ultima Spencer in Spencer and Steyskal 1986b: 247.

###### Description

**(Figs 73, 74).** Wing length 2.5 mm (♂). Female unknown. Length of ultimate section of vein M4 divided by penultimate section: 0.7. Eye height divided by gena height: 5.6–5.9. Facial carina virtually absent and without medial bulb. Ocellar triangle short, reaching between ori and ors, slightly subshiny with corners matt. Fronto-orbital plate subshiny, narrow with indistinct margins expanding to encompass base of setae. Gena receding, highest near or behind middle. Clypeus relatively stout with sides parallel.

***Chaetotaxy***: Male vibrissal fasciculus absent. Two ori; two ors. Mid tibia without posteromedial setae.

***Colouration***: Body, including halter dark brown with gena slightly paler. Wing veins brown. Calypter margin and hairs dark brown.

***Genitalia***: (Figs 405–410) Surstylus fused to, and longer than epandrium, with several irregular rows of shorter, stout setae along inner surface. Single sclerite of basiphallus narrow, short, fused to dorsomedial margin, curved dextrally. Mesophallus stout, nearly as wide as distiphallus with apex narrower; attached to distiphallus within its slightly recessed base. Distiphallus cup-like, subrectangular in outline (ventral view), with inner surface minutely textured transversely medially with ventral longitudinal sclerotisations extending past apex and enclosing medial tubule; base with shallow lobate dorsolateral extensions. Blade of ejaculatory apodeme elongate oval with dark central rib and marginal striations.

###### Host.

Unknown.

###### Distribution.

**Canada**: ON*, QC*. **USA**: VA.

###### Type material.

***Holotype*: USA. VA**: Giles Co., Stony Cross, 2000', 26.v.1962, J.R. Vockeroth (1♂, CNC).

###### Additional material examined.

**Canada. ON**: Wellington Co., Smith Property Trail nr. Arkell, 43°33'N, 80°11'W, 23.vi.2015, O. Lonsdale, CNC441149 (1♂, CNC), QC: Mt Rigaud, 45°27'54"N, 74°19'24"W, 8.vi.2018, B.J. Sinclair, CNC1706881 (1♂, CNC).

###### Comments.

Like other *Ophiomyia*, this species has a truncated clypeus, a very sparsely setose eye, and no posteromedial tibial setae, and unlike *Melanagromyza*, the basiphallus is asymmetrical and the ventrolateral tubules of the distiphallus are absent, although the base of the distiphallus is swollen on either side of the mesophallus to form superficially similar lobes. The surstylus and the shape and arrangement of the basiphallus, mesophallus and distiphallus are distinctive. This species is newly recorded for Canada.

##### Ophiomyia virginiensis

Taxon classificationAnimaliaDipteraAgromyzidae

Spencer

Figs 411 , 412

Ophiomyia virginiensis Spencer in Spencer and Steyskal 1986b: 260.

###### Description.

Wing length 2.2 mm (♂), 2.5 mm (♀). Length of ultimate section of vein M4 divided by penultimate section: 1.0. Eye height divided by gena height: 8.2. Facial carina distinct with large medial bulb (strongly furrowed medially, shiny on dorsal 1/2), not narrowed above bulb. Eye angled diagonally. Gena strongly produced anteriorly, forming an ~ 60° angle (slightly larger angle in specimens from Turkey Run). Clypeus strongly produced and narrowed anteriorly. Crossveins separated by less than length of dm-m.

***Chaetotaxy***: Male vibrissal fasciculus thick and upcurved, ~ 2/3 length of gena. Two ori; two ors. Mid tibia with one posteromedial seta.

***Colouration***: Body, including halter dark brown. Wing veins brown. Calypter margin and hairs dark brown. Abdomen with coppery/golden shine.

***Genitalia***: (Figs 411, 412) Metepiphallus pale and narrow with one pair of ventrobasal spines. Epiphallic lobes evenly dark. Halves of basiphallus fused at base with remainder of left sclerite highly reduced and ill-defined; right sclerite narrow and elongate, exceeding base of distiphallus. Mesophallus base not exceeding that of distiphallus. Distiphallus divided into a large, rounded subtriangular basal lobe, and a slightly narrower distal section that is nearly parallel-sided and compressed dorsoventrally, with a more densely spinulose basal chamber, several sclerotised ventromedial ridges and an internal tubule. Ejaculatory apodeme large, broad, relatively evenly pigmented and without medial rib.

###### Host.

Unknown.

###### Distribution.

**USA**: VA.

###### Type material.

***Holotype*: USA. Virginia**: Hawksbill, Shenandoah N.P., 3600–4050', 7.vi.1962, J.R. Vockeroth (1♂, CNC).

###### Additional material examined.

**USA. VA**: Fairfax Co., Turkey Run Park, nr. mouth of Turkey Run, 38°57.9'N, 7°09.4'W, Malaise trap, 23.viii-18.ix.2007, D.R. Smith (1♂ 3♀, USNM)

###### Comments.

*Ophiomyiavirginiensis* can be characterised by a strongly pronounced furrow on the facial bulb, the ultimate and penultimate sections of M4 are equal in length, the basiphallus is mostly composed of a single narrow band, and the distiphallus is large with discrete sections, including a high, internally smooth subtriangular basal section.

### ﻿Phytomyzinae

#### Amauromyza

Taxon classificationAnimaliaDipteraPhytomyzinae

Hendel

Redia Lioy, 1864: 1313 [preoccupied by Filippi (1837)]. Type species: Agromyzagyrans Fallén, 1823, by subsequent designation [Coquillett 1910: 599]. Frick 1952a [as synonym of Calycomyza]. Spencer and Martinez 1987 [as synonym of Amauromyza].
Amauromyza Hendel, 1931: 59 [as subgenus of Dizygomyza]. Type species: Agromyzalamii Kaltenbach, 1858, by original designation. Frick 1952a: 393 [as subgenus of Phytobia]; Nowakowski 1962: 97 [as genus]; Spencer 1969: 157; Spencer and Steyskal 1986b: 78; Zlobin 1996: 271; Boucher 2012b: 735.
Irenomyia Nowakowski, 1960: 421. Type species: Xeniomyzaobscura Rohdendorf-Holmanova, 1959, by monotypy. Spencer 1966 [as synonym of Melanophytobia]. Spencer and Martinez 1987 [as synonym of Amauromyza].
Melanophytobia Hering, 1960: 127 [as subgenus of Phytobia]. Type species: Phytobiachamaebalani Hering, 1960, by monotypy. Spencer and Martinez 1987).
Campanulomyza Nowakowski, 1962: 97. Type species: Agromyzagyrans Fallén, 1823, by monotypy. Spencer 1976 [synonymy].
Trilobomyza Hendel, 1931: 71 [as subgenus of Dizygomyza]. Type species: Agromyzaflavifrons Meigen, 1830, by original designation. Spencer 1969: 161 [as genus]. Spencer and Steyskal 1986b [as synonym of Cephyalomyza]. Spencer 1987: 877 [as valid subgenus].

*Amauromyza* is a morphologically diverse genus of at least 60 species. Boucher (2012b) provided the most recent treatment of the genus, revising the Canadian fauna and providing a list of world species. The genus is defined entirely by male genitalic characters, making it difficult to identify externally, although many species have a very high gena that can be > 1/3–1/2 the height of the eye (as in *A.karli*), and all Delmarva species have four scattered rows of acrostichal setulae, no acrostichal setae, four dorsocentrals (the presutural seta is sometimes absent in species found elsewhere), erect to reclinate orbital setulae (proclinate or no setulae occur in a minority of species elsewhere), a weakly sclerotised frons that buckles anteriorly in preserved specimens, a costa that extends to vein M1 (to R_4+5_ in two European species), and a dark, heavily pigmented distiphallus that is surrounded by a spinulose membrane. Compounding this difficulty in identification, the most diagnostic character of the genus, a sclerotised, bowl-shaped sperm pump, is also found in some *Nemorimyza*, *Liriomyza* and Agromyzinae. A stout, semi-circular hypandrium is also characteristic, but this character is also found infrequently elsewhere in the subfamily. Previously, those phytomyzine species with a dark or spotted halter could be quickly assigned to the genus, but Zlobin (1996) produced a more thorough definition of *Amauromyza* and discovered that this character was paralleled elsewhere, albeit uncommonly.

Three subgenera were recognised as occurring in the Nearctic by Spencer and Steyskal (1986b): *Cephalomyza* Hendel, *Catalpomyza* Spencer and *Annimyzella* Spencer (now a synonym of *Nemorimyza*; Zlobin 1996). A fourth subgenus, *Trilobomyza* Hendel, which contains the introduced and increasingly widespread *A.flavifrons*, was included as a junior synonym of *Cephalomyza*, although Spencer (1987) used it as valid only a year later. The nominal subgenus is restricted to the Old World. Zlobin (1996) stated that the validity of the subgenera was uncertain, and they are not considered in the present revision pending future study to determine whether or not they are both monophyletic and useful for diagnosis.

### ﻿Key to the Delmarva *Amauromyza*

**Table:** 

1	Frons and parafacial barely visible laterally (Fig. 85). Height of lunule ~ 0.6–0.8 × width. First flagellomere small and short. Basal 1/2 of oviscape pruinose. Height of eye divided by height of gena: 3.9–4.7. Sperm pump pointed ventrally (Fig. 418). Distiphallus narrow, bifid, and twisted counterclockwise; spicules on membrane small and apical (Figs 423, 424)	***A.flavifrons* (Meigen)**
–	Frons and parafacial strongly projecting, continuing as prominent cheek (Fig. 87). Height of lunule < 1/2 width. First flagellomere 0.3 × longer than wide. Oviscape entirely shiny. Height of eye divided by height of gena: 1.6–3.1. Sperm pump broadly and evenly rounded. Distiphallus globular and very dark; sometimes divided, but not bifid; spicules on membrane small to large and extending along length of distiphallus	2
2	Halter mostly brown. Thorax brown with very light pruinosity. Head brown with gena, lunule and centre of frons paler. Two ors. Ocellar seta as long as postocellar. Vibrissa well-developed. Epistoma ~ 2/3 × length of clypeus with anterior margin relatively straight. Head not strongly projecting anterodorsally and eye not reduced in size (height of eye divided by height of gena: 2.3–3.1). “Bowl” of ejaculatory apodeme wider than blade of ejaculatory apodeme; stem relatively wide (Fig. 414). Distiphallus undivided and somewhat rounded (Figs 415–417)	***A.abnormalis* (Malloch)**
–	Halter completely white. Thorax covered with dense silvery-grey pruinosity. Head almost entirely light yellow (Figs 86, 87). One ors. Ocellar seta barely longer than ocellar tubercle. Vibrissa similar to genal setae. Epistoma at least as long as clypeus and strongly tapered anteriorly. Head strongly projecting anterodorsally and eye very small (height of eye divided by height of gena: 1.6–1.8). Sperm pump narrower than blade of ejaculatory apodeme; stem relatively narrow (Fig. 428). Distiphallus divided medially, narrower at base (Figs 426, 427)	***A.karli* (Hendel)**

#### Taxonomic treatment

##### Amauromyza abnormalis

Taxon classificationAnimaliaDipteraPhytomyzinae

(Malloch)

Figs 413–417

Agromyza abnormalis Malloch, 1913a: 320.
Phytobia (Amauromyza) abnormalis . Frick, 1952a: 393, 1959: 378.
Amauromyza abnormalis . Spencer, 1969: 158; Bautista-Martinez et al. 1997: 461.
Amauromyza (Cephalomyza) abnormalis . Spencer, 1981: 148; Spencer and Steyskal 1986b: 273; Boucher 2012b: 737; Černý et al. 2020: 200.

###### Description.

Wing length 1.8–2.2 mm (♂), 2.2–2.7 mm (♀). Length of ultimate section of vein M4 divided by penultimate section: 1.3–2.5. Eye height divided by gena height: 2.3–3.1. Gena broad, ventral margin straight, highest posteriorly. Epistoma ~ 2/3 length of clypeus with anterior margin relatively straight. Fronto-orbital plate and parafacial projecting, visible laterally but less pronounced than in *A.karli*. First flagellomere ~ 1/3 longer than high and with anterodistal corner slightly to indistinctly angulate. Lunule height < 1/2 width. Distance between cross-veins distinctly longer than dm-m. Thorax with light pruinosity.

***Chaetotaxy***: Three ori (uncommonly two or four); two ors. Postocellar and ocellar setae as long as ors. Setulae on tubercle slender, as long as ocellus. Orbital setulae short and indistinct to absent, reclinate. Four dorsocentrals: one presutural, posterior two well-developed, posterior-most slightly longer, anterior two short. Acrostichal setulae in four scattered rows.

***Colouration***: Setae dark brown. Entire body (including knob of halter, which is only paler around sutures) brown except as follows: gena, parafacial, lunule and frons light brown; lateral and posterior margins of frons darker, with dark stripe on margin extending to surround bases of fronto-orbitals; clypeus and lower margin of gena very dark and shiny; face darker towards centre. Calypter margin and hairs pale brown.

***Genitalia***: (Figs 413–417) Hypandrium stout and broadly arched; inner lobe weakly sclerotised, separated by suture and with several medial setae on narrow, well-sclerotised strip. Halves of basiphallus separate, plate-like; hypophallus weakly sclerotised, split. Paraphallus small, lobate, membranous. Mesophallus approximately as long as wide, slightly compressed dorsoventrally. Distiphallus dense, dark, globular; surrounded by minute spinules apically and larger spicules basally (also see comments below). Ejaculatory apodeme broad on distal 2/3 with margin pale; stem broad; sperm pump bowl-shaped, broadly rounded, lightly sclerotised.

###### Hosts.

Amaranthaceae – *Amaranthus* (Benavent-Corai et al. 2005).

###### Distribution.

**Canada**: BC, MB, NB*, ON, QC. **USA**: AZ, CA, CO*, DC, IA, KS, MD*, MO*, NM. Mexico. Germany (Černý et al. 2020). While previously reported Palaearctic records of *A.abnormalis* were determined to represent *A.chenopodivora* Spencer (Spencer 1976; Boucher 2012b), Černý et al. (2020) listed specimens of *A.abnormalis* collected at a single locality in Germany that they suggest result from introduction of its New World host *Amaranthusretroflexus* in Europe.

###### Type material.

***Holotype*: USA. DC**: Washington, vi.1903, “97270, on aphid”, “on roots of *Amarantha*” (1♀, USNM; type No. 15583).

***Paratype*: USA. KS**: Twilight, Lawrence, July, E.S. Tucker (1♀, USNM).

###### Additional material examined.

**Canada. CANADA**. **BC**: Robson, 13.vi.1952, H.R. Foxlee, CNC358588 (1♀, CNC), **MB**: Brandon, 10.viii.1958, J.G. Chillcott, CNC358587 (1♀, CNC), **NB**: Dannebrog, 18.viii.1960, W.F. Rapp (1♀, USNM), **ON**: Chatham, 29.viii.1928, A.B. Baird, 18333A, CNC358590 (1♀, CNC), Ottawa, 11.vii.1957, J.E.H. Martin, CNC358589 (1♀, CNC), **QC**: Abbotsford, 19.vi.1937, G. Shewell, CNC358591 (1♂, CNC). **USA. CO**: Custer Co., 10 mi SW of Wetmore, 8.viii.1973, G.F. and S. Hevel (1♀, USNM), **IA**: Ames, 16.viii.1924, H.L. Sweetman, “2690” (1♀, USNM), Mills Co., 2mi SW Glenwood, 27.viii.1969, R.R. Pinger (1♂, USNM), **MD**: Montgomery Co., 4mi S of Ashton, Malaise trap, G.F. and J.F. Hevel, 6.ix.1981 (1♂, USNM), 31.v.1986 (1♂, USNM), **MO**: Boone Co.: Columbia, Malaise trap, 7am-4pm, 9.ix.1967, P.D. Parker (1♂, USNM), Columbia, Malaise trap, F.D. Parker, 17–31.viii.1968 (1♂ 1♀, USNM), 16–31.vii.1968 (1♂ 2♀, USNM), 1–15.vii.1968 (1♂, USNM), 6.vii.1968 (1♀, USNM), 6.ix.1968 (1♀, USNM), 7.viii.1968 (4♀, USNM).

###### Comments.

*Amauromyzaabnormalis*, an internal stem borer, is a widespread species that can be distinguished from other Delmarva Phytomyzinae by an entirely or predominantly dark halter. Other *Amauromyza* with brown maculations on the halter are known only from California, and the halter of *Nemorimyzamaculosa* (Malloch) is mostly white with a brown spot.

The shape of the distiphallus of the dissected male from Maryland is of an intermediate morphology between those illustrated from California and Quebec in Spencer and Steyskal (1986b), being quite similar to the New Mexico specimen photographed in Boucher (2012b). These intermediates support the concept of a single morphologically variable species, not two separate species, as suggested by Spencer and Steyskal (1986b).

While tentatively treating the European representatives of *Amauromyzaabnormalis* as conspecific with the new World fauna, Spencer (1976) later considered them distinct, placing them in the new species *A.chenopodivora* Spencer. Aside from a difference in host plant genus (*Chenopodiumalbum*), he made this distinction on the basis of slight variation in the male genitalia. The male examined by Sasakawa (1961) has a strong resemblance to the illustration in Spencer (1971) and it is likely that Spencer would have also classified these as *A.chenopodivora*. The illustration of the Californian male in Spencer and Steyskal (1986b), however, has a very strong resemblance to the British male illustrated by Spencer (excluding a slight strengthening of the apex of the basiphallus and a basal proliferation of spinules on the membrane surrounding the distiphallus), and it is uncertain as to what specifically he used as evidence to differentiate the two. The status of *A.chenopodivora* was treated in Boucher (2012b), who examined material from Sweden and found that the specimens were slightly larger with differences in the phallus and concluded that they were distinct from those in the Nearctic.

##### Amauromyza flavifrons

Taxon classificationAnimaliaDipteraPhytomyzinae

(Meigen)

Figs 84 , 85 , 418–424 , 429

Agromyza flavifrons Meigen, 1830: 184.
Agromyza exigua Meigen, 1830: 184. Hendel 1931 [synonymy]. Spencer and Martinez 1987 [synonymy].
Agromyza xanthocephala Brischke, 1881: 242 [nec. Zetterstedt]. Hendel 1931 [synonymy].
Dizygomyza (Trilobomyza) flavifrons . Hendel 1931: 71.
Trilobomyza flavifrons . Spencer 1969: 160.
Amauromyza (Trilobomyza) flavifrons . Spencer 1972: 46, 1987: 877.
Amauromyza (Cephalomyza) flavifrons . Spencer and Steyskal 1986b: 275; Scheffer et al. 2007: 770; Boucher 2012b: 744; Černý et al. 2020: 201.
Amauromyza flavifrons . Scheffer and Lonsdale 2018: 86; Eiseman and Lonsdale 2018: 25; Eiseman et al. 2021: 20.

###### Description

**(Figs 84, 85, 429).** Wing length 1.8–2.3 mm (♂), 2.0–2.4 mm (♀). Length of ultimate section of vein M4 divided by penultimate section: 2.3–3.3. Eye height divided by gena height: 3.9–4.7. Gena shallow, highest posteriorly and strongly angled upwards anteriorly. Epistoma shallow and broadly rounded. Fronto-orbital plate and parafacial soft, fronto-orbital plate somewhat visible laterally. Lunule height ~ 0.6–0.8 × width. First flagellomere subcircular, with minute tuft of slightly longer hairs apically. Distance between cross-veins approximately as long as dm-m or shorter.

***Chaetotaxy***: Two or three ori; two or three ors (rarely one). Orbital setulae short, dark and erect, but sometimes strengthened and appearing as shorter additional ori. Setulae on tubercle strong, erect. Postocellar and ocellar setae as long as ors, with ocellar slightly shorter. Four dorsocentrals, one presutural; decreasing in length anteriorly; sometimes with additional strengthened setula near suture. Acrostichal setulae in four to five scattered rows.

***Colouration***: Setae dark brown. Body colour faintly to strongly infused with orange tint. Head mostly yellow; first flagellomere light brown to brown excluding base, but sometimes more broadly yellowish; pedicel brownish (sometimes yellow apically); back of head dark brown; posterolateral corner of frons dark brown to base of vertical setae; palpus, face, clypeus, and lower margin of gena brown; gena sometimes brownish. Thorax dark brown with light dusting of pruinosity. Halter yellow. Calypter margin and hairs brown. Legs dark brown with apex of fore femur yellow. Abdomen dark brown.

***Genitalia***: (Figs 418–424) Epandrium fused with surstylus, interface evident by partial suture; surstylus setose, short, broadly lobate; surstylus and posterodistal margin of epandrium curved inwards. Subepandrial sclerite consisting of one pair of well-sclerotised lateral plates (one seta each apically) joined by broad membranous space medially; with weakly sclerotised triangular lobe underneath cerci. Epiphallus weakly sclerotised, but divided into one pair of ill-defined plates with four strong ridges on anterior plate that are weakly attached to strong saddle-shaped basal plate meeting phallophorus. Hypandrium small, broadly rounded and stout. Postgonite bare, curved medially, with weak inner-medial lobe, apically subcircular with distal 1/2 weakly sclerotised. Ejaculatory duct broad, lightly pigmented, becoming darker and slightly wider apically. Basiphallus with weak subquadrate dorsobasal plate distally branching (from left side) into two darker bands, the right band wider with dorsum paler; apically narrowed and paler, medially fusing with broad, black ventral extension of distiphallus (possibly homologous to paraphalli). Mesophallus not discernible, likely fused into base of distiphallus. Distiphallus as long as distance from its base to apex of phallophorus; black, split along most of length into two stout processes that slightly widen apically; membrane surrounding distiphallus with minute clear spinulae around apex with medial line smoother. Ejaculatory apodeme paler to apex, broad blade continuous with stem, gradually tapering to base; sperm pump sclerotised, broadly bowl-shaped or somewhat pointed ventromedially.

###### Hosts.

Amaranthaceae – *Beta*, *Spinacia*. Asteraceae – Bidens*; two ON females reared from "Wild Aster”. Caryophyllaceae – *Agrostemma*, *Atocion*, *Atriplex*, *Cerastium*, *Dianthus*, *Gypsophila*, *Honckenya*, *Lychnis*, *Melandrium*, *Moehringia*, *Myosoton*, *Saponaria*, *Silene*, *Stellaria*, *Vaccaria*. Chenopodiaceae – *Chenopodium*, *Spinacia* (Benavent-Corai et al. 2005; Eiseman et al. 2021; Ellis 2021).

###### Distribution.

**Canada**: BC, ON, QC. **USA**: DE, IL, MA, MD*, MI, MN, NC*, NY, OH, OR, PA, VA*, VT, WA; leaf mines only in CT, IA, ID, KY, WI (Eiseman and Lonsdale 2018). Tunisia, Western Europe to the Kyrghyz Republic and North Korea (Spencer 1976; Černý et al. 2020).

###### Type material.

***Holotype***: **Germany** (1♀, MNHN). [Not examined]

###### Material examined.

**Canada. BC**: Vancouver, Point Grey, 17.ix.1972, J.R. Vockeroth (3♂, CNC), **ON**: Ottawa, 695 Malibu Tr., 45°22'16.29"N, 75°42'57"W, 20.vi.2017, O. Lonsdale, ex *Silenevulgaris*, em. 6.vii.2017, CNC799453, CNC799454 (2♀, CNC), Ottawa, 23.viii.1971, ex wild Aster, H.J. Teskey (2♀, CNC), Ottawa, winter 1965–66, bred from leaf mine in *Dianthus*, M. Hildebrand, reared in lab (1♂, CNC), Ottawa, 23.v.1975, H.C.W. Walther (1♀ 4 puparia, CNC), Ottawa, 21.viii.1974, J.R. Vockeroth (1♂, CNC), Ottawa, damp second-growth *Acer*-*Betula* wood, J.R. Vockeroth, 21.v.1991 (1♂, CNC), 25.v.1991 (2♂, CNC), 26.vi.1991 (2♀, CNC), 6.vii.1991 (1♀, CNC), 9.vii.1991 (1♀, CNC), 11.vii.1991 (1♀, CNC), 17.vii.1993 (5♂, CNC), 8.viii.1993 (1♀, CNC), 13.viii.1993 (1♂, CNC), 28.viii.1993 (1♂, CNC), 12.vii.1994 (4♂, CNC), 30.v.1995 (1♂, CNC), 29.viii.1997 (1♂, CNC), 1.ix.1997 (1♂, CNC), 6.xi.1997 (1♂, CNC), 14.viii.1998 (1♂, CNC), 5.vii.2000 (1♂, CNC), 11.vii.2000 (1♂, CNC), 12.vii.2000 (1♂, CNC), 12.viii.2000 (1♂, CNC), 15.viii.2000 (1♂, CNC), 21.viii.2000 (1♂, CNC), 27.viii.2000 (1♂, CNC), 29.viii.2000 (1♂, CNC), 2.x.2000 (1♀, CNC), 16.vii.2002 (1♂, CNC), 22.viii.2002 (1♂, CNC), 16.ix.2002 (1♂, CNC), 14.vii.2003 (2♂, CNC), 15.vii.2003 (2♂, CNC), 3.viii.2003 (1♂, CNC), 21.viii.2003 (1♂, CNC), 24.viii.2002 (2♂, CNC), Thornhill, J.R. Vockeroth, 30.v.1964 (29♂ 33♀, CNC), Metcalf, 28.vii.1993, B.E. Cooper (2♂, CNC), St. Lawrence Is. N.P., Grenadier I. Centre, 28.vii.1975, E. Sigler (2♂, CNC), **QC**: Gatineau Pk., Old Chelsea, H.J. Teskey, 28,vii.1971 (1♂ 1♀[with puparium], CNC), viii.1971 (1♀, CNC), Hull, Diptera on *Bidens*, 25.viii.1960, C.D. Miller (1♀[with puparium and host leaf], CNC). **England.** Torcross, Devon, 14.vii.1942, G.E. Shewell (1♀, CNC). **Europe.** No data, “Dizygom. *flavifrons* Mg. ♂ det. Hendel” (1♂, USNM), “Dizygom. *flavifrons* Mg. det. Hendel” (1♂, USNM). **Germany.** Mine an: *Cerastiumholosteoides*, Hering Z: Crossen a.O., 1964, Mühlhausen Thüringen, H. Buhr, 2298 (2♂[same pin], CNC), Mine an: *Coronariafloscuculi* [=*Lychnisflos-cuculi* L.], Crossen a.O., Hering: Z. (1♀, CNC). **Hungary.** Budapest, Janoshed, em. 21.vi.1964, mine *Melandrium* sp. 28.v.1964, K.A. Spencer (1♀[with puparium], CNC). **USA. MD**: Colesville, W.W. Wirth, 11.vii.1974 (1♀, USNM), 24.vii.1974 (1♂ 2♀, USNM), 14.vi.1975 (1♀, USNM), 4.vii.1976 (2♀, USNM), 28.vii.1976 (2♀, USNM), 14.vi.1977 (1♀, USNM), 18.vi.1977 (1♀, USNM), 28.v.1977 (1♂, USNM), 26.vi.1977 (2♂, USNM), Montgomery Co., Colesville, 3.viii.1979, Malaise trap, W.W. Wirth (1♀, USNM), **OH**: Portage Co., 3mi E Kent, 10.vi.1969, Biol. Note No. 6901, S. Whitney (1♀, USNM), Hocking Co., South Bloomingville, Deep Woods Farm, 24.x.2013, em. 26– 28.iii.2014, C.S. Eiseman, ex *Silenerotundifolia*, #CSE1028, CNC384742–384747 (6♀, CNC), **OR**: Lane Co., Blue River, 24.vii.2016, M.W. Palmer, *Silenecoronaria* em. 18–30.vii.2016, #CSE3037, CNC638887–638890 (3♂,1♀, CNC), **NC**: Jackson Co., Dulaney Bog, 7mi S Cashiers, 19.vi.1986, Malaise trap, W.W. Wirth (1♀, USNM), **VA**: Giles Co., Mountain Lake, 7.ix.1976, G.C. Steyskal (1♂, USNM), Fairfax Co., Turkey Run Park, 0.3 km W mouth Turkey Run, 38°58'N, 77°09.6'W, Malaise trap, 12–26.vii.2007, D.R.Smith (1♀, USNM).

###### Comments.

*Amauromyzaflavifrons*, a distinct black and yellow to orange-tinted species that often has numerous fronto-orbitals, was first collected in North America by J.R. Vockeroth in Thornhill, Ontario in 1964. Spencer (1969) suggested that it may have been introduced from Europe on cultivated *Dianthus*. Collection records support this concept, showing the geographic spread of this species in subsequent years: from Ohio in 1969, Vancouver in 1972, Washington in 1973, Maryland in 1974, Wisconsin in 1975 (Spencer and Steyskal 1986b), Edmonton in 1975 (G.C. Griffiths, pers. comm.), Virginia in 1976, Delaware in 1979 (Spencer and Steyskal 1986b), Vermont in 1980 and North Carolina in 1986. The Edmonton specimens were reared from *Dianthusbarbatus*, emerging on 24–26 July; other mines were only seen within the city on garden plants and weeds, supporting the idea that they were first introduced on commercial plants (G.C. Griffiths, pers. comm.).

*Amauromyzaflavifrons* is known to regularly occur on beet and spinach, but normally low population densities keep it from becoming a serious pest (Dempewolf 2004). The anthomyiid *Pegomya* Macquart also occurs on beet, and mines of one may be confused for the other, although those of *Pegomyia* are much larger, darker, and more damaging to the leaves (C. Eiseman, pers. comm.). Similar damage on *Dianthus* (carnations) causes proportionally more damage, significantly decreasing market value.

##### Amauromyza karli

Taxon classificationAnimaliaDipteraPhytomyzinae

(Hendel)

Figs 86 , 87 , 425–428 , 430

Dizygomyza Karli Hendel, 1927: 253.
Dizygomyza (Cephalomyza) Karli. Hendel 1931: 34.
Amauromyza karli . Spencer 1969: 158, 1977: 242.
Amauromyza (Cephalomyza) karli . Gil-Ortiz et al. 2009, Boucher 2012b: 746; Černý 2018: 123; Černý et al. 2020: 201.

###### Description

**(Figs 86, 87, 430).** Wing length 1.7–2.0 mm (♂), 2.0–2.5 mm (♀). Length of ultimate section of vein M4 divided by penultimate section: 1.8–2.3. Eye height divided by gena height: 1.6–1.8. Gena broad, highest posteriorly; cheek distinct. Epistoma at least as long as very narrow clypeus, strongly pointed anteriorly. Fronto-orbital plate and parafacial produced, visible laterally and strongly projecting anteriorly. First flagellomere ~ 1/3 longer than high with anterodistal corner somewhat angulate, sometimes slightly shorter, and more rounded. Lunule height < 1/2 width. Distance between cross-veins distinctly longer than dm-m.

***Chaetotaxy***: Cephalic setae very slender and relatively short. Vibrissa barely larger than genal setae. Three ori; one ors. Orbital setulae short and indistinct to absent, reclinate. Postocellar and ocellar setae slightly longer than ocellar tubercle; setulae on tubercle slender, as long as ocellus. Four to six dorsocentrals; one or two presutural with anterior two short. Acrostichal setulae in 2–4 straight to scattered rows.

***Colouration***: Setae light brown. Head light yellow with ocellar tubercle and back of head above foramen brown, posterolateral corner of frons with brown spot encompassing vertical setae or just reaching base of inner vertical; base of ors sometimes with brownish spot; clypeus brownish to brown. Thorax dark brown (notopleuron paler) with colour largely obscured by silvery pruinosity. Halter yellow. Calypter white with margin and fringe often lemon yellow, but sometimes slightly brownish. Legs brown with fore coxa light brown, and trochanters, apices of femora (sometimes distal 1/3) and sometimes apices of coxae light yellow; base of tibiae sometimes yellow. Abdomen brown.

***Genitalia***: (Figs 425–428) Hypandrium stout and broadly rounded; inner lobe separated by suture, largely membranous and with several setae on small sclerotised medial plate. Plates of basiphallus short, distinct, approximate. Hypophallus membranous medially and with lateral margins sclerotised. Length of mesophallus ~ 2 × width. Distiphallus very dark, laterally “haired”, C-shaped; entirely split and narrowly divided sagittally; base dorsal, narrower, stem-like; surrounding membrane with minute dark spinules laterally. Ejaculatory apodeme paler to apex, blade gradually narrowing basally to stout stem; sperm pump sclerotised, broad, hemispherical.

###### Host.

Amaranthaceae – *Chenopodiumvulvaria* (Gil-Ortiz et al. 2009), C.album*, C.quinoa*.

###### Distribution.

**Canada**: AB, BC*, MB, NS*, ON, QC, SK*. **USA**: MD*. Europe, Mongolia, South Korea (Černý et al. 2020).

###### Type material.

***Holotype*: Poland.**“bei Stolp in Pommern ”, 9.vii.1924 (1♀, NMW). [Not examined]

###### Material examined.

**CANADA. BC**: Yoho N.P., Kicking Horse Cmpgrnd, forested cmpgrnd, 51.4245°N, 116.4294°W, 1317 m asl, BIOBus (1♀, CNC), **NS**: Cumberland Co., Malagash Blue Sea Beach Day Park, 45°48'17"N, 63°18'15"W, 4.viii.2013, O. Lonsdale (1♂, CNC), Kouchibouguac N.P., 2.vii.1977, J.R. Vockeroth (1♂, CNC), **ON**: Ottawa, 45°19'1.20"N, 75°43'12"W, 90 m, 20.vi.2016, J.E. O’Hara, Malaise trap, CNC629539, CNC629543 (2♂, CNC),Ottawa, 23.vi.1964 (1♀, CNC), Essex Co., Kingsville, ex *Chenopodiumalbum*, em. i.1990, 17.ix.1989, K.L.P. Heal (2♂ 1♀, CNC), **SK**: near Clavet, *quinoa* field, 6.ix.2017, CNC939976 (1♂, CNC), vii-viii.2017, CNC939980, CNC939982–939984 (1♂ 3♀, CNC), near Colonsay, *quinoa* field, 5.vii.2017, NIS 2017-298, CNC939974, CNC939975 (2?, CNC). **USA. MD**: Montgomery Co., Colesville, 26.vi.1977, Malaise trap, W.W. Wirth (1♂, USNM), 30.vi.1977 (1♂, USNM), 21.v.1977 (1♂, USNM), 14.vi.1977 (1♀, USNM), 4.vii.1976 (1♀, USNM), 18.ix.1977 (1♂ 1♀, USNM), 4mi SW of Ashton, Malaise trap, G.F. and J.F. Hevel, 1.ix.1981 (1♂ 1♀, USNM), 6.vi.1986 (1♂, USNM), Bethseda, 10.vii.1970, G. Steyskal (1♀, USNM).

###### Comments.

*Amauromyzakarli* is here newly recorded for the United States. The large, bright yellow head, large epistoma, thick grey thoracic pruinosity and unusual phallus are diagnostic.

##### Aulagromyza

Taxon classificationAnimaliaDipteraPhytomyzinae

Enderlein

Aulagromyza Enderlein, 1936a: 180. Type species: Phytagromyzahamata Hendel, 1932: 283, by monotypy. von Tschirnhaus 1991: 304.
Paraphytomyza Enderlein, 1936a: 180. [nomen nudum – no type species designated].
Paraphytomyza Enderlein, 1936b: 43. Type species: Phytomyzaxylostei Robineau-Desvoidy, 1851: 398, by original designation [=Phytagromyzaluteoscutellata Meijere, 1924 – original type misidentified, corrected by ICZN (1988)]. Spencer 1969: 203, 1987a: 255, 1987b: 557; Spencer and Steyskal 1986b: 162. von Tschirnhaus 1991 [synonymy].
Rubiomyza Nowakowski, 1962: 102. Type species: Agromyzasimilis Brischke, 1881: 258, by original designation. Spencer 1969 [synonymy].

Literature prior to Spencer (1969) often grouped the Nearctic species of this genus in *Phytagromyza*, which became a synonym of *Cerodontha* when Nowakowski (1962) found that the type species, *Agromyzaflavocingulata* Strobl, was a senior synonym of C. (Poemyza) semiatra Hendel – see Spencer (1969) for discussion. Following that publication, the name *Paraphytomyza* was used until von Tschirnhaus (1991) found that name to be a junior synonym of the previously monotypic *Aulagromyza*. Mirroring earlier proposals by Nowakowski (1962) and Spencer (1969), preliminary studies (Zlobin 2007c; Winkler et al. 2009) are increasingly supportive of a polyphyletic *Aulagromyza*, with the unusual bright yellow Salicaceae-feeding species possibly being more closely related to *Phytomyza*. These include *A. tridentata* (Loew), which is treated below.

Species of *Aulagromyza* are diagnosed by a costa that ends at vein R_4+5_, the relative position of the cross-veins (if dm-m is present, it is distal to r-m, and if dm-m is absent then r-m is positioned far from bm-m), orbital setulae that are never proclinate (differentiating it from the similar *Phytomyza*), and an absence of the posteromedial mid tibial setae. Larvae are stem and leaf miners in Apocynaceae, Asteraceae, Brassicaceae, Caprifoliaceae, Dipsacaceae, Fabaceae, Oleaceae, Rosaceae, Rubiaceae, and Salicaceae (Zlobin 2007c).

Zlobin (2007c) recognised 52 World species, 40 of which were Palaearctic. Three species have been confirmed in the Delmarva states, but Eiseman and Lonsdale (2019) also reported mines likely attributable to *Aulagromyzacornigera* (Griffiths), a leaf miner on Caprifoliaceae.

### ﻿Key to the Delmarva *Aulagromyza*

**Table:** 

1	Body almost entirely yellow (Fig. 89). Calypter entirely white. Frons strongly widened. Eye 2 × height of gena	***A.tridentata* (Loew)**
–	Body almost entirely brown (Fig. 88). Calypter margin and hairs dark. Frons not much wider than eye. Eye at least 3 × height of gena	2
2	Vein dm-m present (Fig. 435). Wing length 1.9–2.0 mm♂, 1.9–2.2 mm♀. Ocellar triangle as long as wide. Gena horizontal on posterior 1/2, angled upwards on anterior 1/2. Three ori. Two dorsocentral setae. Acrostichal setulae in six irregular rows	***A.nitida* (Malloch)**
–	Vein dm-m absent (Fig. 436). Wing length 1.8–2.4 mm♂, 2.1–2.6 mm♀. Ocellar triangle wider than long. Genal margin straight, angled upwards. Two ori with anterior seta sometimes reduced. Five dorsocentral setae. Acrostichal setulae in four scattered rows	***A.orbitalis* (Melander)**

#### Species descriptions

##### Aulagromyza nitida

Taxon classificationAnimaliaDipteraPhytomyzinae

(Malloch)

Figs 88 , 431–434 , 435

Agromyza nitida Malloch, 1913a: 288. Frick 1952a: 373.
Phytagromyza nitida . Frick, 1953: 74, 1959: 417.
Paraphytomyza nitida . Spencer, 1969: 207; Spencer and Steyskal 1986b: 163.
Aulagromyza nitida . von Tschirnhaus, 1991: 305; Scheffer et al. 2007: 771.

###### Description

**(Figs 88, 435).** Wing length 1.9–2.0 mm (♂), 1.9–2.2 mm (♀). Length of ultimate section of vein M4 divided by penultimate section: 2.4–2.8. Eye height divided by gena height: 3.7–5.0. First flagellomere relatively narrow, not higher than pedicel. Fronto-orbital plate slightly projecting, especially anteriorly. Gena horizontal on posterior 1/2, angled upwards on anterior 1/2. Proboscis relatively narrow, elongate, and geniculate. M_1+2_ weak. Cross-veins separated by length of dm-m. Ocellar triangle as long as wide.

***Chaetotaxy***: Three ori; two ors (slightly curved inwards, difficult to differentiate from ori). Ocellar seta as long as or slightly longer than tubercle, sometimes narrower than postocellar; postocellar longer, well-developed. Orbital setulae very few in number, weak, slightly reclinate. Two dorsocentrals, anterior seta 1/2–3/5 length. Six irregular rows of acrostichal setulae.

***Colouration***: Setae dark brown. Body predominantly dark brown, subshiny. Antenna brownish yellow with distal 2/3 or more of first flagellomere brown; clypeus and back of head brown; frons brownish yellow with ocellar triangle dark brown and dark spot at base of vertical setae extending to posterior or anterior ors along eye margin; remainder of fronto-orbital plate variably brownish from lateral margin; lunule, gena, palpus and parafacial yellow with shiny brown strip along ventral margin of gena; mouthparts brownish, partly brown along proboscis; face brown to dark brown, sometimes becoming paler laterally. Postpronotum light yellow to dirty white with brown anterior spot or anterior region fading to brown, but sometimes only posterior margin pale; notopleuron often paler brown with posterior margin sometimes yellowish. Fore knee (and occasionally mid and hind knees) yellowish. Halter yellow. Calypter margin and hairs dark brown. Wing veins brown.

***Genitalia***: (Figs 431–434) Surstylus separate from epandrium, minutely setose, small and rounded with extended posterobasal margin. Cerci separated by membranous region with weakly sclerotised transverse striations. Subepandrial sclerite composed of two converging lateral arms that are weakly connected to one pair of apical setae. Hypandrium stout and broadly rounded with apex slightly produced; inner lobe with small medial floating setose sclerite, and separate, narrow, curved sclerite with several basal setulae. Postgonite long and narrow with apex cleft and setulose. Phallus weakly sclerotised. Plates of basiphallus broad with bases converging and interlocking. Paraphallus small and leaf-like with apices pointed and bent. Mesophallus short, curved dorsally, laterally flattened, fused to distiphallus. Distiphallus bifid on distal 1/2 with slightly wider apical cups. Ejaculatory apodeme short and not strongly widened apically.

###### Host.

Unknown – likely a stem miner on *Galium* (Rubiaceae) based on similarities to related species (Spencer 1990).

###### Distribution.

**Canada**: AB. **USA**: CO*, CT*, DC*, IA, IL, KS, MD, NC, NY*, VA, WA*.

###### Type material.

***Holotype*: USA. MD**: Cabin John Bridge, 28.iv.1912, Knab and Malloch (1♀, USNM; type No. 15566).

###### Additional material examined.

**Canada. AB**: George Lk., 21.vi.1966, K.A. Spencer, CNC165181 (1♀, CNC). **USA. CO**: Boulder, Flagstaff Cn., 1767 m, on side of stream, 10.vi.1961, C.H. Mann, CNC358592 (1♀, CNC), **CT**: Stamford, 18.v.1919, A.H. Stutevant Collection, 1970 (1♀, USNM), **DC**: 11.vi.1926, J.M Aldrich (1♀, USNM), **IL**: White Heath, 8.v.1915, J.M. Aldrich (1♂, USNM), **KS**: Nat. Hist. Res., Lawrence, 26.v.1956, J.G. Chillcott, CNC391383, CNC391384 (2♂, CNC), 27.iii.1954, CNC358595, CNC358596 (2♂, CNC), 28.iv.1956, CNC391380–391382, CNC391385–391393, CNC358593–358594,CNC358597–358598 (16♂, CNC), **MD**: Lavale, 9.v.1970, G. Steyskal (1♂, USNM), Montgomery Co., Bethseda, 5.v.1968, L.V. Knutson (1♀, USNM), Bethseda, 4.v.1969, G. Steyskal (1♂, USNM), **NY**: Tuxedo, 29.v.1926, A.L. Melander (1♂, USNM), **VA**: Chain Bridge, 23.iv.1922, J.R. Malloch (1♀, USNM), Fairfax Co., Turkey Run Park, nr. Headquarters bldg. 38°57.7'N, 77°08.9'N, Malaise trap, 29.iii-17.iv.2007, D.R.Smith (2♂, USNM), Turkey Run Park, 0.3 km W mouth Turkey Run, 38°58'N, 77°09.6'W, Malaise trap, 29.iii-25.iv.2007, D.R.Smith (2♂ 6♀, USNM), Great Falls Park, swamp trail, 38°59.4'N, 77°15.2'W, Malaise trap, 18.iv-2.v.2007, D.R.Smith, trap #2 (1♂ 1♀, USNM), **WA**: Vashon, 28.v.1937, A.L. Melander (1♀, USNM).

###### Comments.

The two common Delmarva *Aulagromyza*, *A.nitida* and *A.orbitalis*, are primarily brown with whitish shoulders, and can occur in relatively large numbers. The only other common eastern *Aulagromyza* is *A.luteoscutellata* (Spencer and Steyskal 1986b: figs 1044–1046), which has a more yellowish head and notum, and is found in Europe, Canada, and the northern United States.

##### Aulagromyza orbitalis

Taxon classificationAnimaliaDipteraPhytomyzinae

(Melander)

Figs 436–441

Phytomyza orbtalis Melander, 1913: 271.
Phytagromyza orbitalis . Frick 1952a: 416, 1957: 204 [lectotype designation], 1959: 417.
Paraphytomyza orbitalis . Spencer 1969: 207; Spencer and Steyskal 1986b: 165.
Aulagromyza orbitalis . von Tschirnhaus 1991: 305; Scheffer et al. 2007: 771; Eiseman and Lonsdale 2018: 26; Eiseman et al. 2021: 21.

###### Description

**(Fig. 436).** Wing length 1.8–2.4 mm (♂), 2.1–2.6 mm (♀). Vein dm-m absent. M_1+2_ nearly spectral. First flagellomere slightly longer than high. Eye height divided by gena height: 4.8–8.1. Parafacial very narrow. Gena straight, angled upwards. Ocellar triangle length ~ 2/3 width with posterior ocelli widely spaced. Lunule shallow, semi-circular.

***Chaetotaxy***: Two ori (anterior seta sometimes reduced); two ors. Postocellar and ocellar setae well-developed. Orbital setulae very small, fine, reclinate or more erect, in one row. Five to six dorsocentrals, four postsutural, decreasing in length anteriorly. Four relatively straight to scattered rows of acrostichal setulae. Prescutellar acrostichal seta small to strong. Small seta medial to presutural supra-alar.

***Colouration***: Setae brown to dark brown. Base colour of body dark brown with brown pruinosity. Head dirty yellow to brownish; antenna yellow to brownish with first flagellomere brown to dark brown (inner base sometimes yellowish); frons with broad brown spot surrounding base of vertical setae that sometimes extends along eye margin lateral to posterior fronto-orbital; fronto-orbital plate sometimes brownish; ocellar triangle dark brown with broadly rounded corners that only slightly extend outside tubercle; gena brown below cheek with ventral margin dark brown; palpus light brown; face with brown mottling that predominates laterally, or face brown with yellow tint and yellow vertical stripe medially; back of head and clypeus dark brown. Postpronotum light yellow with large dark brown spot reaching anterior margin; notopleuron light yellow; anterolateral corner of postsutural scutum usually narrowly light yellow; posterolateral corner of scutum lateral to scutellum with ill-defined light yellow spot. Pleuron dark with limited light yellow margins posterodorsally on anepisternum. Halter yellow. Calypter pale with hairs brown. Wing veins brown. Legs with apex of femora yellow for length approximately equal to width of femur apex; fore tibia dirty yellow to yellow, sometimes brownish medially; base of mid and hind tibiae sometimes narrowly yellow; tarsi dirty yellow.

***Genitalia***: (Figs 437–441) Epandrium shallow, loosely articulated with surstylus. Surstylus small, lobate, short setose. Cercus small, narrow. Hypandrium relatively thin with inner lobe broad and wrapped around dorsum of postgonite; lobe with two long inner setae and anteromedial process with several sockets. Postgonite long and narrow with dorsomedial tooth and upcurved split apex with two subapical setae. Phallus slightly twisted. Phallophorus short. Basiphallus composed of two broad, narrow plates, right 1/2 emarginate ventromedially; weakly connected to lobate paraphallus. Mesophallus not evident. Distiphallus 1/2 length of basiphallus, subcylindrical with base swollen and basally desclerotised, with indistinct ventromedial suture, apically widened and fringed; surrounded by subspherical sclerotised ball that is open dorsoapically and strengthened by transverse band dorsobasally. Ejaculatory apodeme evenly pigmented with narrow stalk and relatively broad apex.

###### Hosts.

Caprifoliaceae – *Diervilla*, *Lonicera*, *Symphoricarpos*, *Triosteum* (Eiseman et al. 2021).

###### Distribution.

**Canada**: AB, BC*, MB, ON*, QC*, SK*. **USA**: CA, CO*, IA, ID, IL*, KS, MA, MD*, MI, NC, OK, WA, WI*.

###### Type material.

***Lectotype*: USA. WA**: Kamiac Butte, 1.vi.1912, A.L. Melander collection (1♂, USNM).

***Paralectotypes*: USA. ID**: Troy, 14.vi.1908, A.L. Melander (2♀, USNM), Moscow, Mt., A.L. Melander, 3.vi.1911 (2♂, USNM), 6.vii.1912 (1♀, USNM), 5.vi.1912 (1♂, USNM), Collins, “Aug.”, A.L. Melander (1♂, USNM), **WA**: Kamiac Butte, 1.vi.1912, A.L. Melander (2♀, USNM), Pullman, A.L. Melander, 10.vi.1911 (1♂, USNM), 12.v.1912 (1♀, USNM), 19.v.1912 (1♂ 2♀, USNM), Oroville, A.L. Melander (1♀, USNM), Oroville, 1.v.1912, A.L. Melander (1♂, USNM).

###### Additional material examined.

**Canada. AB**: Edmonton, “Em. 31-8-71”, ex honeysuckle (1♀, CFS), Edmonton, White Mud Creek, G.C.D. Griffiths, died after spraying, 9.vii.1975 (2♂ 1♀, CNC), 14.vii.1975 (3♂ 3♀, UASM), 23.vii.1975 (6♂ 8♀, UASM), Elk Island Nat. Park, 19.vi.1971, from larvae on *Symphoricarposoccidentalis*, emerged 13–14.v.1972, E50, G.C.D. Griffiths (3♂ [with puparia], UASM), Elk Island Nat. Park, 4.vii.1971, from larva on *Symphoricarposoccidentalis*, emerged 11–13.v.1972, E87, G.C.D. Griffiths (6♂ 4♀[with puparia], UASM), Edmonton, 15.ix.1973, from larvae on *Loniceradioica*, emerged 18–26.iv.1974, E203, G.C.D. Griffiths (1♂ 2♀[with puparia], UASM), Edmonton, White Mud Creek, 11–12.vi.1975, from larvae on *Symphoricarposoccidentalis*, emerged 5–7.vii.1975, E220, G.C.D. Griffiths (8♂ 6♀[with puparia], UASM), Edmonton, White Mud Creek, 19.vi.1975, from larva on *Symphoricarposalbus*, emerged 11–13.vii.1975, E226, G.C.D. Griffiths (3♂ 1♀[with puparia], UASM), Edmonton, White Mud Creek, 23.ix.1975, from larvae on *Symphoricarposoccidentalis*, emerged 18.iv.1976, E286, G.C.D. Griffiths (1♀[with puparium], UASM), C.E. Lee Sanctuary, Devon Sandhills, 25.viii.1979, from larvae on *Symphoricarposoccidentalis*, emerged 27.iv.1980, E396, G.C.D. Griffiths (3♂[with puparia], UASM), George Lake, near Busby, 12.vi.1973, from larva on *Symphoricarposalbus*, emerged 22–24.iv.1974, G12, G.C.D. Griffiths (4♂ 4♀[with puparia], UASM), George Lake, near Busby, 2.vii.1975, from larva on *Symphoricarposalbus*, emerged 21.vii.1975, 19–21.iv.1976, G41, G.C.D. Griffiths (2♂ 2♀[with puparia], UASM), George Lake, near Busby, 27.vii.1875, from larvae on *Loniceradioica*, emerged 11.v.1976, G52, G.C.D. Griffiths (1♂ 1♀[with puparia], UASM), Brandon, 18.vii.1958, R.B. Madge (1♀, CNC), Morrin, 12.vi.1929, G.F. Manson (1♂, CNC), Lethbridge, J.H. Pepper, 3.vi.1929 (1♂, CNC), 5.vi.1929 (1♂, CNC), Okotoks, K.A. Spencer, 10.vi.1966 (1♀, CNC), 11.vi.1966 (1♀, CNC), **BC**: Robson, H.R. Foxlee, 10.v.1947(1♀, CNC), viii.1947 (1♀, CNC), 6.v.1950 (1♀, CNC), 26.v.1947 (1♂, CNC), Elkwater L., 20.vii.1956, O. Peck (1♂ 1♀, CNC), Trinity Valley, 22.vi.1937, H.B. Leech (1♀, CNC), Hagensborg, 12.vii.1992, A. Borkent (1♂, CNC), 50 km E Bella Coola, 8.vii.1992, A. Borkent (1♂, CNC), **ON**: Simcoe, 29.v.1939, G.E. Shewell (1♀, CNC), Ottawa, 5.vii.1938, G.E. Shewell (1♀, CNC), Ottawa, 16.x.1956, J.R. Vockeroth (1♀, CNC), St. Lawrence Is. Nat. Park, Thwartway Is., 20.vi.1976, W. Reid (1♀, CNC), **QC**: Wakefield, 26.vi.1946, G.E. Shewell (1♀, CNC), Mt. St. Hilare, 500–700', 3.vi.1964, J.R. Vockeroth (1♀, CNC), **SK**: 18 km S Maple Creek, by small clear stream, 49°45'N, 109°29'W, 10.vii.2005, J. R. Vockeroth, yellow pan trap, CNC287400 (1 ♀, CNC). **USA. CA**: San Diego Co., Julian, *Symphoricarops*, 4300', 9.iv.1977, K.A. Spencer (1♀, USNM), Cuyamaca, S.P, 20.iv.1977, 4200', K.A. Spencer (1♀, USNM), Alameda Co., Berkeley, [illegible], 15.v.1977, on *Lonicerainvolucrata*, K.A. Spencer (1♀, USNM), **CO**: Boulder, Flagstaff Cn., 5800', 10.vi.1961, C.H. Mann (1♂, CNC), **IA**: Winneshiek Co., 43°26'32.50"N, 92°0'10.32"W, 17.vi.2013, em. by 6.vii.2013, M.J. Hatfield, ex *Triosteumaurantiacum* (1♂, CNC), **ID**: Waha, 30.v.1924, A.L. Melander (1♀, USNM), **IL**: White Heath, 18.v.1917 (1♀, USNM), 2.vi.1917 (1♀, USNM), **KS**: Nat. Hist. Res., Lawrence, 28.iv.1936, J.G. Chillcott (1♂, USNM), Nat. Hist. Res., Lawrence, J.G. Chillcott, 27.iii.1954 (4♂, CNC), 28.iii.1956 (1♂, CNC), 28.iv.1956 (2♂, CNC), 27.iii.1957 (1♂, CNC), **MA**: Hampshire Co., Pelham, Butter Hill Wildlife Sanctuary, 2.vi.2015, em. 23–27.vi.2015, C.S. Eiseman, ex *Diervillalonicera*, #CSE1646, CNC564730, CNC564731 (2♂, CNC), **MD**: Chesapeake Bch., 5.vii.1924, J.R. Malloch (1♀, USNM), **NC**: Scotland Co., Laurinburg, St. Andrews University, 14.iv.2016, T.S. Feldman, *Lonicerasempervirens*, em. 13.v.2016, #CSE2460, CNC653946, CNC653947 (2♀, CNC), **WI**: Valleyford, A.L. Melander, 25.vi.1924 (3♂, USNM), 19.vi.1924 (1♀, USNM), 17.v.1924 (1♀, USNM).

###### Comments.

Only 16 of the 21 described paralectotypes of this species are present in the USNM. The location of the remaining types is unknown.

##### Aulagromyza tridentata

Taxon classificationAnimaliaDipteraPhytomyzinae

(Loew)

Figs 89 , 442–445

Agromyza tridentata Loew, 1858: 76. Hendel 1932: 295.
Paraphytomyza tridentata . Spencer 1972: 67, 1976: 323.
Aulagromyza tridentata . von Tschirnhaus 1991: 305; Scheffer et al. 2007: 771; Papp and Černý 2016: 349.

###### Description

**(Fig. 89).** Wing length 1.6 mm (♂), 2.2 mm (♀). Vein dm-m absent. Eye height divided by gena height: 1.8–2.1. Gena very high and broadly rounded. Fronto-orbital plate projecting, but not as prominent as broad, rounded parafacial and cheek. Ocellar triangle slightly larger than tubercle, weakly defined, corners rounded. Vein M_1+2_ spectral and close to wing apex, M4 and CuA+CuP similarly weak. Body with faint greyish pruinosity that is slightly denser on thorax.

***Chaetotaxy***: Two ori; two ors (possibly three ors and one ori). Postocellar and ocellar setae well-developed. Orbital setulae few, minute, slightly reclinate. Four dorsocentrals; one presutural, length decreasing anteriorly. Four rows of acrostichal setulae, becoming two irregular rows posteriorly.

***Colouration***: Setae light yellow to slightly brownish, with notal setae brown to black in female and sometimes browner posteriorly on notum in male. Body predominantly lemon yellow; ocellar tubercle brown; small brown spots sometimes present lateral to vertical setae; back of head with broad brown stripe extending from foramen; mediotergite and sometimes anterior region of anatergite brown; scutum with medial stripe ending before posterior dorsocentral, fused anteriorly (examined European specimen) or completely to one pair of posteriorly narrowing intra-alar stripes that reach neither anterior nor posterior margins; pleuron with small light greyish brown spots ventrally on katatergite and meron. Halter yellow. Calypter white. Wing veins light yellow.

***Genitalia***: (Figs 442–445) Epandrium pale and broad. Surstylus large, broadly rounded and twisted so that setulose inner face visible posteriorly. Subepandrial sclerite subrectangular, flat, weakly sclerotised and bare. Hypandrium small, thin, and broadly rounded; inner lobe with two setae. Postgonite large, broad, and thick with flat dorsum, inner-posterior ridge, long posterobasal extension, and pointed apical process on inner face. Phallophorus fused to base of single plate of basiphallus, which is membranous on right margin and bifid apically. Distiphallus membranous and flagellate; paraphallus distinct, narrow, and medially curved. Ejaculatory apodeme small and pale past base and with apex clear.

###### Host.

Salicaceae – *Salix* sp.

###### Distribution.

**USA**: CO, DE*, MD*. Europe, extending eastward to Kazakh Republic.

###### Type material.

***Holotype***: **Poland.** Poznan area (ST ?, ZIL). [Not examined]

###### Material examined.

**GERMANY.** Berlin, Dahlem, 19.v.1953, mine an *Salixalba*, No. 5941, CNC165187 (1♂, CNC). **USA. DE**: Bridgeville, 14.vii.1960, willow, P. Burbutis (2♂ 2♀ [with mined leaf], USNM), Bridgeville, 15.vi.1960, willow leaf miner, P.Burbutis (1♂, USNM), **MD**: Montgomery Co., Colesville, 26.vi.1977, Malaise trap, W.W. Wirth (1♀, USNM).

###### Comments.

This is the second record of *Aulagromyzatridentata* in the United States, with Scheffer et al. (2007) rearing material from *Salix* in Colorado. European specimens of *A.tridentata* differ in having the notal patch more conspicuously divided into bands. These uncommon Salicaeae-feeding *Aulagromyza* differ from Nearctic congeners in being lemon-yellow with pale setae, and are likely to be mistaken for paler *Phytoliriomyza* or a species of *Liriomyza*. The only other previous Nearctic records of this group were Ontario specimens of *A.populicola* (Walker) reared from *Populusdeltoides* in Ottawa (Frick 1959; Spencer 1969) and two females from Oklahoma reared from the same species (Eiseman and Lonsdale 2018). *Aulagromyzapopulicola* is a vittate species highly similar in appearance to *A.tridentata*, but it has the scutal pattern brownish orange (not darker brown), and the phallus is more extensively sclerotised and structured apically (Spencer 1976: figs 579, 580).

The identity of this species was determined by comparison to a description and illustrations of *Aulagromyzatridentata* presented by Spencer (1976). A similar description for *A.tridentata* appears in Papp and Černý (2016), but the genitalic illustration does not match; an illustration approximating the expected genitalic morphology for this species is provided for the species *A.populicola* (Walker). The genitalic and external morphology of *A.populicola* was verified from a series collected in Ottawa that was reported in Spencer (1969).

##### Calycomyza

Taxon classificationAnimaliaDipteraPhytomyzinae

Hendel

Calycomyza Hendel, 1931: 65 [as subgenus of Dizygomyza]. Type species: Agromyzaartemisiae Kaltenbach, 1856: 236, by original designation. Frick 1952a: 394 [as subgenus of Phytobia], 1956: 284, 1959: 387; Nowakowski 1962: 97 [as genus]; Spencer 1969: 144; Spencer and Steyskal 1986b: 140.

Adult *Calycomyza* are usually readily diagnosed by a whitish yellow head and shoulders (the dark Caribbean species *C.obscura* is an exception) with a black scutellum and antenna, and usually two dorsocentrals. Many species are relatively uniform in appearance and are often only separable on the basis of slight external and male genitalic characters. Many *Liriomyza* are similarly coloured, but almost all have a medially yellow scutellum, there are four dorsocentrals, and the ejaculatory duct is apically swollen and pigmented. Other diagnostic features of *Calycomyza* include weak reclinate orbital setulae in a single row, a shallow, semi-circular lunule and unmistakable *Calycomyza*-like genitalia, including a dense patch of scattered tubercle-like setae on the inner-distal margin of the epandrium and surstylus. A character not previously noted in the genus is a minute, round, heavily pigmented sclerite floating between the mesophallus and distiphallus. This “medial sclerite” (Figs 450, 451) is absent in several species, including *C.humeralis* (Roser), *C.platyptera* (Thompson) and *C.verbenae* (Hering), but it is otherwise widespread in the genus and possibly synapomorphic. Frick (1956) provided an excellent treatment of the Nearctic species known at the time, including a detailed description of biology and host plant species.

### ﻿Key to the Delmarva *Calycomyza*

**Table:** 

1	First flagellomere slightly produced anterodistally, forming an angle (Fig. 95). Mid tibia with one posteromedial seta that is sometimes reduced to absent. Hypophallus divided into two J-shaped lateral bands, each 1/2 having a short basomedial curve (Figs 468–470)	2
–	First flagellomere circular (Fig. 96); if with a slight angle (some *C.promissa*), then with two mid tibial setae. Mid tibia usually with two or three posteromedial setae (one or more setae reduced in *C.platyptera* and some *C.promissa*). Hypophallus without medial curve as above	3
2	Face entirely brown (Fig. 94). Wing length 1.4–1.6 mm ♂, 1.6–2.0 mm ♀. Small medial sclerite between mesophallus and distiphallus absent (Fig. 468). Halves of distiphallus dark, closely spaced; each tubule (seen laterally) relatively narrow and opening distally	***C.humeralis* (Roser)**
–	Face yellow, sometimes with brown region below antennal bases. Wing length 1.6–2.0 mm ♂, 2.0–2.3 mm ♀. Small medial sclerite between mesophallus and distiphallus present (Fig. 470). Halves of distiphallus relatively pale, widely spaced; each tubule (seen laterally) relatively broad with opening angled dorsally	***C.solidaginis* (Kaltenbach)**
3	Five or six pairs of fronto-orbital setae. Wing length 2.9–3.2 mm	4
–	Four pairs of fronto-orbitals; additional ori sometimes present only on one side in larger specimens. Wing length 1.5–2.7 mm	5
4	Scutum with one pair of large yellow posterolateral spots overlapping margin of scutellum (Fig. 98). Fronto-orbital plate entirely yellow. Face, scape, pedicel, lower margin of gena and apex of fore femur yellow. Two well-developed posterior dorsocentral setae and one smaller anterior dorsocentral. Distiphallus widest at basal cup-like structure; fringed inner structures projecting (Fig. 459)	***C.flavinotum* (Frick)**
–	Scutum with smaller, faded yellowish posterolateral spots. Fronto-orbital plate brown in part, at least to base of posterior ors. Face, pedicel, scape, lower margin of gena and apex of fore femur brown. Six dorsocentral setae, steadily decreasing in size anteriorly. Distiphallus widest apically; fringed inner processes concealed (Fig. 465)	***C.gigantea* (Frick)**
5	Calypter margin and hairs entirely pure white. Puparium elevated on a lump or distinct stalk of frass	6
–	Calypter margin yellow or dirty-white to brown (rarely white), with hairs golden to dark brown. Puparium not known to be elevated on frass stalk	7
6	Anterior (third) dorsocentral thin, < 1/2 length of mid dorsocentral; fourth dorsocentral never present. Posteromedial tibial setae sometimes variably reduced. Wing length 1.5–1.6 mm ♂ (up to 1.8 in Canadian specimens), 1.6–2.2 mm ♀. Hypophallus well-sclerotised on both sides and fused to basiphallus. Mesophallus slender with width ~ 1/3 length. Outer sheath on distiphallus dark and tapered (Figs 473, 474). Ejaculatory apodeme relatively clear and narrow (Fig. 472)	***C.platyptera* (Thompson)**
–	Third dorsocentral nearly as thick as second dorsocentral and ~ 1/2–4/5 as long; small fourth sutural dorsocentral present. Mid tibia always with two posteromedial setae. Wing length 1.8–2.1 mm ♂, 2.3–2.6 mm ♀. Hypophallus membranous on left margin and separate from basiphallus. Mesophallus broad with width more than 1/2 length. Outer sheath on distiphallus relatively clear and flared (Figs 482, 483). Ejaculatory apodeme relatively dark, with width exceeding length (Fig. 480)	***C.promissa* (Frick)**
7	Fronto-orbital plate dark at least to base of posterior ors, often continuing anteriorly as long distinct stripe or weak lateral stripe fading to base of anterior ors. Distiphallus bifid	8
–	Fronto-orbital plate at most with faint brownish stripe from posterior margin of frons to base of posterior ors along fronto-orbital plate. Distiphallus branched or undivided	9
8	Wing length 1.8–2.2 mm. Hypophallus membranous with lateral sclerotisation. Mesophallus stout, not much longer than wide. Halves of distiphallus subspherical, stout, with long membranous distolateral extension (Figs 476, 477)	***C.malvae* (Burgess)**
–	Wing length 2.3–2.7 mm. Hypophallus large, plate-like. Mesophallus narrow, much longer than wide. Halves of distiphallus narrower, longer than wide (Figs 478, 479)	***C.orientalis* Spencer**
9	Base of posterior ors surrounded by yellow. Paraphallus well-developed. Distiphallus composed of single tapered structure (Figs 461, 462). Mesophallus subcylindrical	***C.frickiana* Spencer**
–	Frons with light brown stripe extending from posterior margin of frons to base of posterior ors along fronto-orbital plate. Paraphallus absent. Distiphallus not forming single, tapered sclerite as above. Mesophallus bulging near midpoint or base	**10**
10	Wing length 1.8–2.0 mm. Eye height divided by gena height: 3.4–3.6. Hypophallus large with medial triangular sclerotised section. Distiphallus narrow, subcylindrical and partially fused to mesophallus (Figs 454, 455). Mesophallus short and strongly constricted medially (seen ventrally)	***C.barbarensis* Spencer**
–	Wing length 1.8–2.6 mm. Eye height divided by gena height: 3.8–5.4. Hypophallus narrower with centre membranous. Distiphallus broad, not cylindrical. Mesophallus long and not strongly constricted medially	**11**
11	Mesophallus not much longer than wide; small dark sclerite present between mesophallus and distiphallus; outer cover of distiphallus minutely spinulose internally, short, and exposing strongly produced and haired central structure (Figs 450, 451). Lateral margins of hypophallus membranous	***C.avira* Eiseman & Lonsdale**
–	Mesophallus with length ~ 2 × width and bent ventrally at midpoint; dark sclerite between mesophallus and distiphallus absent; outer cover on distiphallus very dark, surrounding central haired structure (Figs 488, 489). Lateral margins of hypophallus well-sclerotised	***C.verbenae* (Hering)**

#### Species descriptions

##### Calycomyza avira

Taxon classificationAnimaliaDipteraPhytomyzinae

Eiseman & Lonsdale

Figs 446–451

Calycomyza avira Eiseman & Lonsdale, 2018: 28; Eiseman et al. 2019: 306.

###### Description

**(from Eiseman and Lonsdale 2018).** Wing length 2.1–2.5 mm (♂), 2.6 mm (♀). Length of ultimate section of vein M4 divided by penultimate section: 2.1–2.3. Eye height divided by gena height: 4.5–5.3. First flagellomere rounded. Arista pubescent. Notum subshiny.

***Chaetotaxy***: Two ori; two ors. Postocellar and ocellar setae well-developed. Three dorsocentral setae, third thin, < 1/2 length of second dorsocentral. Six rows of acrostichal setulae. Two posteromedial setae on mid tibia.

***Colouration***: Setae dark brown. Head yellow with back of head, clypeus, palpus, ocellar tubercle and antenna dark brown; frons brownish in posterolateral corner, with faint stripe to base of posterior ors along fronto-orbital plate that is slightly darker in tentatively associated female. Thorax dark brown with postpronotum (excluding dark anteromedial spot confluent with margin), notopleuron (excluding dark elongate sublateral spot) and small anterolateral spot behind suture yellow; posterodorsal corner of anepisternum yellow along suture. Calypter margin yellow and hairs brown. Halter white. Legs and abdomen dark brown with fore knee yellow.

***Genitalia***: (Figs 446–451) Basiphallus composed of one pair of lateral sclerotised bars on distal 1/2. Paraphallus absent. Hypophallus short and entirely membranous. Mesophallus short, slightly longer than wide, round and slightly compressed dorsoventrally; minute, dark medial sclerite present between mesophallus and distiphallus. Distiphallus short with tubules fused and folded, lateral margins sclerotised at stem-like base, short medial bowl with inner face heavily spinulose. CT males with bowl of distiphallus narrower and inner processes separate and not as strongly haired.

###### Hosts.

Asteraceae – *Bidensaristosa* (Eiseman et al. 2019), *B.frondosa*.

###### Distribution.

**USA**: CT, MA, NC, NY, WV.

###### Type material.

***Holotype*: USA. CT**: Redding, 31.v.1930, A.L. Melander (1♂, USNM).

***Paratypes*: USA. CT**: Redding, 1.vi.1929, A.L. Melander (1♂, USNM), **MA**: Berkshire Co., Sheffield, 9.vii.2014, em. by 28.vii.2014, C.S. Eiseman, ex *Bidensfrondosa*, #CSE1225, CNC384838 (1♂, CNC), **NY**: Letchworth State Park, 13.vi.1963, pond margin, W.W. Wirth (1♂, CNC), **NC**: Durham Co., Durham, Grandale Drive, 14.vii.2017, em. 1–3.viii.2017, T.S. Feldman, ex *Bidens*, #CSE4047, CNC939843, CNC939844 (1♂ 1♀, CNC), **WV**: White Sulfur Springs, 16.vi.1970, G. Steyskal (1♂, USNM).

###### Additional material examined.

**USA. MA**: Plymouth Co., W Bridgewater, 41°59'44.50"N, 71°3'18.48"W, 15.viii.2013, ex. *Bidensfrondosa*, em. by 5.v.2014, C.S. Eiseman, #CSE1101, CNC384790 (1♀, CNC).

##### Calycomyza barbarensis

Taxon classificationAnimaliaDipteraPhytomyzinae

Spencer

Figs 452–455

Calycomyza barbarensis Spencer, 1981: 298. Spencer and Steyskal 1986b: 151.

###### Description.

Wing length 1.8 mm (♂). Female unknown. Length of ultimate section of vein M4 divided by penultimate section: 1.6–2.4. Eye height divided by gena height: 3.4–3.6. First flagellomere rounded. Notum shiny.

***Chaetotaxy***: Two ori; two ors. Postocellar and ocellar setae well-developed, the latter slightly smaller. Two well-developed dorsocentral setae. Six rows of acrostichal setulae. Ocellar seta slightly less developed than postocellar. Two posteromedial setae on mid tibia.

***Colouration***: Setae dark brown. Head yellow with back of head, clypeus, palpus, ocellar tubercle and antenna dark brown; frons brownish in posterolateral corner with light brown stripe extending from posterior margin to base of posterior ors along fronto-orbital plate Thorax dark brown with postpronotum (excluding dark anteromedial spot confluent with margin), notopleuron (excluding dark elongate sublateral spot) and small anterolateral spot behind suture yellow. Halter white. Calypter margin light brown with brown hairs. Legs and abdomen dark brown with fore knee narrowly yellow.

***Genitalia***: (Figs 452–455) Epandrium and surstylus with dense patch of tubercle-like setae on inner-distal margin. Hypandrium narrow, inner lobe following dorsal margin of postgonite and with two short setae on inner face. Postgonite short, pointed apically, with two outer-distal setulae. Basiphallus broadly sclerotised dorsally. Paraphallus nearly vestigial, produced as lightly sclerotised triangular extensions of membrane. Hypophallus large and broad with basolateral margins and triangular medial section well-sclerotised. Distiphallus and mesophallus short and subequal in length, fused; mesophallus constricted medially; distiphallus subcylindrical, undivided, widest at base; medial floating sclerite absent.

###### Host.

Unknown.

###### Distribution.

**USA**: CA, DE*, KS*, MO*, SC*, TX*.

###### Type material.

***Holotype*: USA. CA**: Santa Barbara Co., Los Prietos, 23.vi.1965, J.A. Powell (1♂, CASC). [Not examined]

###### Material examined.

**USA. CA**: Riverside, 2.xii.1934, A.L. Melander (1♂, USNM), **DE**: Georgetown, 3.viii.1977, W.R. Allen, “79-3513”, ex. trap in soy bean plot (1♂, USNM), **KS**: Riley Co., 4.ix.1967, G.F. Hevel (1♂, USNM), **MO**: Boone Co., Columbia, 4.ix.1968, Malaise trap, F.D. Parker (1♂, USNM), **SC**: “Spartanbrg”, G.G. Ainslie, Webster No. 4853 (1♂, USNM), **TX**: San Antonio, 2.iv.1942, A.L. Melander (1♂, USNM).

###### Comments.

Previously known only from California, *Calycomyzabarbarensis* now appears to be widespread throughout the United States. The narrow, undivided distiphallus is characteristic, as is the dark triangular plate on the hypophallus.

##### Calycomyza flavinotum

Taxon classificationAnimaliaDipteraPhytomyzinae

(Frick)

Figs 98 , 456–459

Agromyza allecta . Misidentification, in part. Frost 1924: 38.
Phytobia (Calycomyza) flavinotum Frick, 1956: 297.
Calycomyza flavinotum . Spencer, 1969: 147; Spencer and Steyskal 1986b: 146; Scheffer et al. 2007: 771; Eiseman and Lonsdale 2018: 31.

###### Description

**(Fig. 98).** Wing length 2.8–3.5 mm (♂), 3.0–3.7 mm (♀). Length of ultimate section of vein M4 divided by penultimate section: 1.9–2.6. Eye height divided by gena height: 4.7–6.0. First flagellomere circular, slightly longer than wide. Fronto-orbital plate slightly visible anteriorly (viewed laterally). Notum subshiny.

***Chaetotaxy***: Two ors, three or four ori. Postocellar and ocellar setae well-developed. Three dorsocentral setae; anterior seta < 1/2 length of second dorsocentral. Eight to ten rows of acrostichal setulae. Two posteromedial setae on mid tibia.

***Colouration***: Setae dark brown. Head yellow with back of head, clypeus, palpus and ocellar tubercle dark brown; antenna dark brown with scape and pedicel paler and/or with distal margin yellowish; frons brown to dark brown in posterolateral corner with spot usually reaching base of inner vertical seta Thorax dark brown with postpronotum (excluding dark anteromedial spot confluent with margin), notopleuron (excluding dark elongate sublateral spot), lateral postsutural margin and large posterolateral spot on scutum yellow; most sutures on pleuron yellow with posterodorsal corner of anepisternum and anepimeron around wing base more widely yellow. Halter white. Calypter margin and hairs brown. Legs and abdomen dark brown with fore knee and male cercus and perianal region yellow.

***Genitalia***: (Figs 456–459) Epandrium and surstylus with dense patch of tubercle-like setae on inner-distal margin. Hypandrium thin, inner lobe extending to surround outer surface of postgonite, with one seta. Postgonite small and simple. Basiphallus sclerotised laterally and anterodorsally, confluent with hypophallus. Paraphallus absent. Hypophallus long and narrow with sides sclerotised on basal 2/3. Distiphallus and mesophallus subequal in length; mesophallus dorsoventrally compressed, subovate in ventral view. Distiphallus with tubules short, with narrow, mostly clear basal stem, outer cover short and spinulose, and inner process coiled, long-haired and widely exposed.

###### Hosts.

Asteraceae – *Arctium*, *Carduus*, *Eupatorium*, *Eutrochium* (Spencer and Steyskal 1986b; Spencer 1990; Eiseman and Lonsdale 2018). Spencer notes that this species probably occurs on *Ageratina*, referring to unspecified records in Spencer and Steyskal (1986b); this host record may be attributable to observed mines of *C.eupatoriphaga* Eiseman and Lonsdale. *Eupatorium* is probably the primary host for this species (Spencer 1990), and in Ontario, it is regularly observed on *Arctium*, with many larvae mining much of the surface area of the leaves, which can be quite large.

###### Distribution.

**Canada**: ON. **USA**: CA*, MA, MD*, ME, MN, NY, PA, VA*, WI.

###### Type material.

***Holotype*: USA. NY**: Elma, 20.viii.1911, M.C. vanDuzee (1♂, USNM).

###### Paratypes examined.

**Canada. ON**: Ottawa, 14.vii.1946, A. Brooks, No. 6367, CNC391397 (1♀, CNC), Ottawa, [adult] on *Viburnumpubescens*, 19.vii.1946, G.E. Shewell, No. 6367, CNC391401 (1♂, CNC).

###### Additional material examined.

**Canada. ON**: Bells Corners, 30.viii.1984, H.J. Teskey, ex *Arctium*, “REARED Assoc. Vial”, CNC391398, CNC391399 (1♂ 1♀, CNC), Ottawa, Central Experimental Farm, 8.vii.2014, O. Lonsdale, *Arctium* sp., em. 28.vii.2014, CNC352948, CNC349315–349318 (10 ex, CNC), St. Lawrence Is. Nat. Park, McDonald Is., 23.viii.1976, A. Carter, Code 4497-A, CNC391403 (1♀, CNC), St. Lawrence Is. Nat. Park, Thwartway Is., 10.viii.1976, G. Calderwood, Code 4382-P, CNC391402 (1♀, CNC), Wallacetown, 18.ii.1980, “no host”, det. lot 80-1283, 9, 795-2319-01, CNC391400 (1♂, CNC). **USA. CA**: Mpls. Orium, Minnehaha, [illegible], 3.viii.1980, mine *Eupatoriumrugosum*, 29.vi.1980 (1♂, USNM), **IL**: White Heath, 25.vi.1939, J.C. Dirke, herb No. 45 (1♂, USNM), **MA**: Berkshire Co., Savoy, Tannery Falls, 12.vii.2012, C.S. Eiseman, ex leaf mine *Eupatoriumperfoliatum*, em. 31.vii.2012, #CSE15, CNC391394–391396 (1♂ 2♀, CNC), Franklin Co., Northfield, 276 Old Wendell Rd., 8.ix.2013, C.S. Eiseman, ex *Arctiumminus*, em. 11.x.2013,em. 17.iv.2014, #CSE936, #CSE1083, CNC392639, CNC384749–384753 (3♂,3♀, CNC), **MD**: Elkron, 84-31, DFB, em. viii.1984, “Hoor ?” (1♂, USNM), **VA**: Great Falls, 9.vii.1936, A.L. Melander (1♂, USNM).

###### Comments.

A pair of broad yellow posterolateral spots on the scutum is diagnostic of *Calycomyzaflavinotum*, as is the large plate-like hypophallus and the pair of haired, coiled processes apically on the distiphallus.

In the original description, the depository of the holotype was incorrectly listed as CASC, not USNM.

##### Calycomyza frickiana

Taxon classificationAnimaliaDipteraPhytomyzinae

Spencer

Figs 460–463

Calycomyza allecta . Misidentification, in part. Frick 1956: 298 [see Spencer and Steyskal (1986b)for details].
Calycomyza frickiana Spencer & Steyskal, 1986b: 299.

###### Description

**(Fig. 463).** Wing length 2.3–2.5 mm (♂), 2.5–2.9 mm (♀). Length of ultimate section of vein M4 divided by penultimate section: 1.9–2.6. Eye height divided by gena height: 3.8–4.8. First flagellomere circular. Notum subshiny.

***Chaetotaxy***: Two ori; two ors; third ori sometimes present on one side. Ocellar seta long, but sometimes not much longer than tubercle. Postocellar well-developed. Two well-developed dorsocentral setae, with setula in front of anterior dorsocentral short but well-developed, appearing as third dorsocentral. Six rows of acrostichal setulae. Two posteromedial setae on mid tibia.

***Colouration***: Setae dark brown. Head yellow with back of head, clypeus, palpus, ocellar tubercle and antenna dark brown; frons brownish in posterolateral corner. Thorax dark brown with postpronotum (excluding dark anteromedial spot confluent with margin), notopleuron (excluding dark elongate sublateral spot) and lateral margin of postsutural scutum yellow. Halter white. Calypter margin and hairs brown. Legs and abdomen dark brown with fore knee yellow.

***Genitalia***: (Figs 460–462) Epandrium and surstylus with dense patch of tubercle-like setae on inner-distal margin. Hypandrium narrow, lobe wrapped around postgonite, with one anteromedial seta. Postgonite narrow posteriorly and strongly tapered apically. Basiphallus sclerotised laterally and subapically, ventral membrane also slightly sclerotised. Hypophallus short, rounded and lightly sclerotised laterally at base. Paraphallus pointed, strongly sclerotised, not connected medially by membrane. Mesophallus as long as basiphallus and subcylindrical. Distiphallus globular and undivided with subbasal constriction and paired ventromedial sclerotised bands.

###### Hosts.

Asteraceae – *Rudbeckia* spp. (including *R.laciniata*), *Helianthus* sp., *Bidensfrondosa* (Spencer and Steyskal 1986b). *Bidens* is considered to be the primary host (Spencer 1990).

###### Distribution.

**USA**: IL, IN, MD, MN, NY, PA, SD, VA, WI(?).

###### Type material.

***Holotype*: USA. NY**: Seneca Co., East Varick, ex. *Bidensfrondosa*, 30.x.1956 (1♂, lost [originally deposited USNM]).

###### Paratypes examined.

**USA. IL**: Urbana, 20.vi.1915, J.M. Aldrich (1♀, USNM), Lafayette, v.1927, J.M. Aldrich (1♂, USNM), **SD**: Erwin, vi.1908, J.M. Aldrich (1♂ 1♀, USNM), **VA**: Blacksburg, 2100', 28.v.1962, J.G. Chillcott, CNC391404 (1♀, CNC).

###### Additional material examined.

**USA. MD**: Elkton, 84-53, DFB, em. 28.viii.1984, a leaf miner (1♀, USNM), **VA**: Fairfax Co., Turkey Run Park, nr. Mouth of Turkey Run, 38°57.9'N, 7°09.4'W, Malaise trap, 4–27.ix.2007, D.R. Smith (1♀, USNM).

###### Comments.

*Calycomyzafrickiana* is the only Delmarva *Calycomyza* with a dark calypter and a fronto-orbital plate that is entirely yellow around the base of the fronto-orbitals. Outside of the Delmarva states, some *C.ambrosiae* (Frick) (Spencer and Steyskal 1986b: figs 939, 940) also share these features, but the unique distiphallus and strong paraphallus of C. *frickiana* are diagnostic.

##### Calycomyza gigantea

Taxon classificationAnimaliaDipteraPhytomyzinae

(Frick)

Figs 464 , 465

Agromyza platyptera . Misidentification, in part. Malloch 1913: 293.
Phytobia (Calycomyza) gigantea Frick, 1956: 296.
Calycomyza gigantea . Spencer 1969: 149; Spencer and Steyskal 1986b: 145.

###### Description.

Wing length 3.1–3.2 mm (♂), 2.9–3.3 mm (♀). Length of ultimate section of vein M4 divided by penultimate section: 2.3–2.5. Eye height divided by gena height: 3.4–4.1. First flagellomere circular. Notum with relatively dense greyish pruinosity.

***Chaetotaxy***: Three or four ori, with anterior seta sometimes smaller; two ors. Postocellar and ocellar setae well-developed. Six closely spaced dorsocentrals, two presutural, decreasing in size anteriorly. Acrostichal setulae usually in five to six irregular rows; relatively long and sparse. Two posteromedial setae on mid tibia.

***Colouration***: Setae dark brown. Head yellow with back of head, clypeus, palpus, ventral margin of gena, ocellar triangle (slightly larger than tubercle with corners rounded) and antenna dark brown; frons dark brown in posterolateral corner with stripe extending anteriorly to base of anterior ors, with stripe sometimes broadly enclosing both ors and (sometimes) posterior ori and with lateral margin of eye thinly brown, or fronto-orbital plate with variable mottling near bases of ori (fainter anteriorly); dorsomedial section of parafacial sometimes brown; face evenly dark brown or with pigment faded medially and ventrally. Thorax dark brown with postpronotum (excluding dark anteromedial spot confluent with margin), notopleuron (excluding dark elongate sublateral spot) and supra-alar spot yellow. Halter white. Calypter margin and hairs dark brown. Legs dark brown. Abdomen dark brown, tergites 1–5 with narrow yellow band along posterior margin.

***Genitalia***: (Figs 464, 465) Epandrium and surstylus with dense patch of tubercle-like setae on inner-distal margin. Hypophallus broad and membranous with medial sclerotisation that is divided on basal 1/2 and with short supapical projection. Basiphallus sclerotised dorsally. Paraphallus absent. Mesophallus slightly broader at midpoint and ~ 2.5 × longer than wide. Distiphallus bifid, widest apically, and with inner fringe of hairs concealed within small apical cup; floating medial sclerite minute. Ejaculatory apodeme dark and fan-shaped with sublateral sclerotised band and marginal striations on blade; sperm pump sclerotised ventrally.

###### Host.

Unknown – swept from *Clematis* (Ranunculaceae) (Frick 1956).

###### Distribution.

**Canada**: ON, QC. **USA**: MD, IL, CA, NY, VA, WV*.

###### Type material.

***Holotype*: USA. MD**: Cabin John Bridge, 28.vi.1912, Knab and Malloch (1♂, USNM).

###### Paratypes examined.

**Canada**. **ON**: Bell’s Cor., wild Clematis, 4.vi.1952, J.F. McAlpine, sweeping, No. 6369, CNC391407 (1♀, CNC), Niagra Glen, 15.vi.1926, G.S. Walley, No. 6369, CNC391408 (1♂, CNC), **QC**: Wakefield, 26.vi.1946, G.S. Walley, No. 6369, CNC391406 (1♀, CNC).

###### Additional material examined.

**Canada. ON**: Wellington Co., Guelph, Stone Rd. E, 16.ix.1991, Malaise, R.A. Cannings (1♀, RBCM). **USA. VA**: Blacksburg, 2100', 28.v.1962, J.G. Chillcott, CNC391405 (1♀, CNC), **WV**: Elk Garden, 25.vi.1968, R. and E. Froeschner (1♀, USNM), Logan Co., Island Creek, 2mi N Mountain View, 19.vi.1991, S.M. Clark (1♀, VPIC).

###### Comments.

*Calycomyzagigantea* is an easily diagnosed species, having a large body size, more than four fronto-orbitals, numerous dorsocentral setae that steadily decrease in height anteriorly, a greyish thorax and an elongate bifid distiphallus with a clear basal stalk (normally short or fused in congeners).

##### Calycomyza humeralis

Taxon classificationAnimaliaDipteraPhytomyzinae

(Roser)

Figs 94 , 95 , 466–469

Agromyza humeralis Roser, 1840: 63.
Agromyza atripes Brischke, 1881: 259. Spencer and Martinez 1987 [synonymy].
Dizygomyza (Calycomyza) humeralis . Hendel, 1931: 68; Meijere 1938: 75, 1940: 175, 1943: 68; Hering 1951: 42.
Phytobia (Calycomyza) humeralis . Frick 1952a: 394, 1956: 290, 1959: 390.
Agromyza bellidis Kaltenbach, 1858: 82. Hendel 1931 [synonymy].
Dizygomyza bellidis . Hendel, 1920: 136, 1923: 394; Meijere 1925: 257; Hering 1925: 133, 1927: 57.
Calycomyza humeralis . Spencer, 1969: 149; Spencer and Steyskal 1986b: 144; Boucher and Wheeler 2001: 613; Scheffer and Lonsdale 2018: 86; Eiseman and Lonsdale 2018: 31.

###### Description

**(Figs 94, 95).** Wing length 1.4–1.6 mm (♂), 1.6–2.0 mm (♀). Length of ultimate section of vein M4 divided by penultimate section: 2.6–3.5. Eye height divided by gena height: 1.5–4.1. First flagellomere with shallow angle on anterodorsal margin. Notum with light pruinosity.

***Chaetotaxy***: Two ori; two ors. Ocellar seta thin, not much longer than tubercle to slightly longer. Postocellar well-developed. Two well-developed dorsocentral setae, setula in front of anterior dorsocentral relatively well-developed but short, sometimes appearing as third dorsocentral. Six rows of acrostichal setulae, less commonly four. One posteromedial seta on mid tibia that is sometimes reduced to absent.

***Colouration***: Setae dark brown. Head yellow with back of head, clypeus, palpus, ocellar triangle (slightly larger than tubercle and rounded) and antenna dark brown; frons brownish to brown in posterolateral corner to base of posterior ors; sometimes fronto-orbital plate brownish to brown to level of anterior ors, sometimes with lateral margin very thinly brownish along margin of eye and around base of fronto-orbitals (less commonly with plate entirely brown to base of anterior fronto-orbital); face and lower margin of gena dark brown with face often slightly paler along midline; dorsomedial region of parafacial brownish. Thorax dark brown with postpronotum (excluding dark anteromedial spot confluent with margin), notopleuron (excluding dark elongate sublateral spot) and sometimes small anterolateral spot behind suture yellow. Halter white. Calypter margin and hairs white. Legs and abdomen dark brown.

***Genitalia***: (Figs 466–469) Epandrium and surstylus with dense patch of tubercle-like setae on inner-distal margin. Hypandrium with one seta on inner lobe that surrounds postgonite. Postgonite rounded apically and deeply cleft. Basiphallus well-sclerotised dorsolaterally on left side and dorsoapically. Hypophallus with one pair of long, narrow sclerites approximately as long as basiphallus with irregular outline and small inner-basal process. Paraphallus rod-like with base wider; paraphalli diverging, angled anteroventrally, joined basally by narrow transverse sclerite. Mesophallus slightly longer than basiphallus, slightly bulbous at base; small sclerite between mesophallus and distiphallus absent. Distiphallus with two short, dark, completely divided tubules that are relatively broadly separated, especially at tapered base; base slightly compressed in lateral view, apex slightly swollen and cup-like with apical opening constricted and directed distally; inner fringed structure slightly emerged apically. Ejaculatory apodeme similar to that of *C.platyptera*.

###### Hosts.

Asteraceae – *Aster*, *Baccharis*, *Bellis*, *Bellium*, *Callistephus*, *Conyza*, *Dicrocephala*, *Erigeron*, *Haplopappus*, *Helianthus*, *Heterotheca*, *Hysterionica*, *Madia*, *Solidago*, *Symphyotrichum*, *Tithonia*, *Zinnia* (Spencer and Steyskal 1986b; Spencer 1990; Eiseman and Lonsdale 2018). Also known from *Pentstemonprocerus* (Scrophulariaceae) in CA (Spencer 1981).

###### Distribution.

**Canada**: AB*, ON, QC, SK, YT. **USA**: widespread, but not known from FL. Africa. Australia. Argentina. Chile. Europe. Oriental Region.

###### Type material.

***Holotype***: **Germany.** Baden-Wurttemburg (1♂, SMNS). [Not examined]

###### Material examined.

45♂ 64♀, CNC, USNM (USA: AR, AZ, CA, DC, IN, KS, MA, MD, MS, NC, NM, NV, OH, OK, SC, TX, UT, VT, WA, WI; Canada: SK; Germany).

###### New provincial records.

**Canada. AB**: C.E. Lee Sanctuary, Devon Sandhills, on *Erigeronphiladelphicus*, larva collected 2.vi.1980, emerged 18.vi.1980, G.C.D. Griffiths, E404 (1♂, UASM), N shore Cooking L., 53°26–27'N, 113°00–01'W, on *Asterbrachyactis*, puparium collected 8.viii.1977, emerged 18.viii.1977, G.C.D. Griffiths (1♂, UASM).

###### Comments.

Compared to other species of *Calycomyza*, the height of the gena varies greatly in *C.humeralis* and *C.solidaginis*, which are likely sister-species and further characterised by a shallow angle on the anterodorsal margin of the first flagellomere, only one posteromedial seta on the mid tibia and very similar male genitalia. A very slight point is also found on the antenna of *C.minor* (Florida), which is much smaller (wing length 1.1–1.3 mm), the length of the ultimate section of vein M4 divided by the penultimate section is 4.0, there are only three fronto-orbitals, there is no posteromedial seta on the mid tibia and the face is yellow. South Carolina specimens of *C.minor* Spencer discussed in Spencer and Steyskal (1986b) do not share these combinations of characters and are not conspecific, including one from Coosawhatche here treated as *C.humeralis*. Aside from the angulate flagellomere, *C.humeralis* can be diagnosed in part by an entirely brown face (not yellow with the dorsal region brown), which is relatively uncommon in the genus.

##### Calycomyza malvae

Taxon classificationAnimaliaDipteraPhytomyzinae

(Burgess)

Figs 97 , 475–477

Oscinis malvae Burgess, 1880: 202. Frick 1952a: 395 [as synonym of *jucunda* Wulp].
Agromyza jucunda . Misidentification, in part. Coquillett 1898: 77.
Agromyza cassiae . Misidentification, in part. Frost 1936: 306.
Phytobia (Calycomyza) malvae . Frick 1956: 298 [lectotype designation], 1959: 391.
Calycomyza althaeae Spencer, 1969: 146. Spencer and Stegmaier 1973 [synonymy].
Calycomyza malvae . Spencer and Stegmaier 1973: 81; Spencer and Steyskal 1986b: 149; Martinez and Etienne 2002: 31; Scheffer and Lonsdale 2018: 86; Eiseman and Lonsdale 2018: 32; Monteiro et al. 2019: 167.

###### Description

**(Fig. 97).** Wing length 1.8–2.0 mm (♂), 1.9–2.2 mm (♀). Length of ultimate section of vein M4 divided by penultimate section: 2.3–2.8. Eye height divided by gena height: 4.6–5.0. First flagellomere circular. Notum subshiny.

***Chaetotaxy***: Two ori; two ors. Ocellar seta slightly thinner and shorter than well-developed postocellar. Two well-developed dorsocentral setae. Six rows of acrostichal setulae. Two or three posteromedial setae on mid tibia.

***Colouration***: Setae dark brown. Head yellow with back of head, clypeus, palpus, ocellar triangle (restricted to, or barely larger than tubercle) and antenna dark brown; frons brown in posterolateral corner to base of posterior ors along fronto-orbital plate, and lateral margin of frons usually brownish to level of anterior ors; if fronto-orbital plate with stripe reduced to faded brownish stripe reaching posterior ors (uncommon), apex of fore femur narrowly yellow; face usually yellow with small brownish spot below base of antenna faintly, but sometimes light brown to brown on dorsal 1/2. Thorax dark brown with postpronotum (excluding dark anteromedial spot confluent with margin), notopleuron (excluding dark elongate sublateral spot) and small anterolateral spot behind suture yellow. Halter white. Calypter margin and hairs brown. Legs and abdomen dark brown.

***Genitalia***: (Figs 97, 475–477) Epandrium and surstylus with dense patch of tubercle-like setae on inner-distal margin. Hypandrium thin, with one seta on broad inner lobe. Postgonite bare, simple. Basiphallus sclerotised along distal and left lateral margins. Paraphalli leaf-like, not meeting, lightly and gradually sclerotised medially; left lobe reduced. Hypophallus broad apically, sclerotised along base and right lateral margin. Mesophallus nearly as wide as long, slightly expanded distolaterally. Distiphallus entirely divided with stalk absent, base bulbous and subspherical, outer cover narrow and pointed laterally, and inner process well-developed. Ejaculatory apodeme as long as phallus, stem stout and blending into slightly flared blade with clear distal margin.

###### Hosts.

Malvaceae – *Abutilon*, *Alcea*, *Althaea*, *Gaya*, *Hibiscus*, *Malva*, *Malvastrum*, *Sida*, *Sphaeralcea*, *Urena*. Fabaceae – *Centrosema*, *Glycine*, *Phaseolus*, *Senna*. (Spencer and Steyskal 1986b; Benavent-Corai et al. 2005; Servín et al. 2013; Eiseman and Lonsdale 2018).

###### Distribution.

**Canada**: ON. **USA**: AZ, CA, DC, FL, IN, MA, MD, MS, NC, NM, NY, OK, PA, TX, VA*, WI. Argentina. Bahamas. Brazil. Chile. Dominican Republic*. Jamaica. Mexico. Panama. Venezuela.

###### Type material.

***Lectotype* [*malvae*]: USA. DC**: Washington, 14.xi.1879, T. Pergande (1♀, USNM [specimen on edge away from puparium]; type No. 19127).

###### Paralectotype examined

[***malvae*]: USA. DC**: [same pin as lectotype] (1♀, USNM).

***Holotype* [*althaeae*]: Canada. ON**: Toronto, em. 24.vii.1967, mine Althaearosea 14.vii.1967 (1♂, CNC).

###### Paratype examined

[***althaeae*]: Canada. ON**: Toronto, em. 26.vii.1967, mine Althaearosea 14.vii.1967 (1♀, CNC).

###### Additional material examined.

**Bahamas**. New Providence Isl.: Nassau, 3.i.1953, E.B. Hayden and L. Giovannoli, A.M.N.H. Bahama Isls. Exped., CNC391453 (1♂, CNC). **Canada. ON**: Harrow, 29.ix.1968, H.R. Boyce, leaf mine *Abutilontheophrasti*, CNC391459, CNC391460 (1♂,1♀, CNC). **Dominican Republic.** Espaillat Prov., 24.viii.1967, L.H. Rolston, CNC391451 (1♂, CNC). **USA. AZ**: Tempe, V.L. Wilder, Webster No. 7286 (1♀ 3♂, USNM), **FL**: Gainesville, 25.iv.1952, O. Peck, CNC391452 (1♂, CNC), Homestead, 28.iii.1952, G.S. Walley, CNC391455 (1♀, CNC), J.R. Vockeroth, CNC391454 (1♂, CNC), 4.iv.1952, G.S. Walley, CNC391457 (1♀, CNC), J.R. Vockeroth, CNC391456, CNC391458 (2♀, CNC), **IN**: Lafayette, leaf-miner in *Abutiliontheophrasti*, J.M. Aldrich (1♂ 1♀ [with puparia], USNM), Lafayette, bred from hollyhock 16.xi.1913, J.M. Aldrich (2♂ 2♀, USNM), Lafayette, hollyhock leaf miner 2–5.viii, emerged ? (1♂ [with puparium], USNM), “x-17”, “leaf miner in Malva rot..folia”, J.M. Aldrich (1♂ 2♀, USNM), Lafayette, cage No. C1367a, issued by x.18.15, reared from *Sidaspinosa*, Satterthwalt (1♀, USNM), Lafayette, 19.ix.1915, cage No. C1367b, issued by Jan 10, reared from *Sidaspinosa*, Satterthwalt (1♀, USNM), Lafayette, from *Malvarotundifolia* (1♂, USNM), Lafayette, from *Malvarotundifolia*, 15.xi.1913 (1♂, USNM), **MA**: Hampshire Co., Pelham, Quarry St., 2.vii.2013, ex. Malvacf.rotundifolia, em. 13–15.vii.2013, C.S. Eiseman, #CSE686, CNC384778 (1♂, CNC), **MS**: Shaw, 24.x.1979, H. Walker, ex. leaf mines on *Abutilontheophrasti* (1♂ 2♀, USNM), **NC**: Scotland Co., Laurinburg, St. Andrews University, 17.viii.2015, T.S. Feldman, *Sidarhombifolia*, em. 28.viii-ix.2015 , #CSE2119, CNC653958–653965 (5♂ 3♀, CNC), 29.vi.2016, T.S. Feldman, em. 14.vii.2016, #CSE2754, CNC659975 (1♀, CNC), **OK**: Payne Co., Perkins, 30.vi.2015, M.W. Palmer, *Sennamarilandica*, em. by 12.vii.2015, #CSE1821, CNC564700–564702 (2♂ 1♀, CNC), Payne Co., Mehan, 7.vii.2016, M.W. Palmer, *Sennamarilandica*, em. by 31.vii.2016 , #CSE3047, CNC653983, CNC653984 (1♂ 1♀, CNC), **VA**: Fairfax Co., Great Falls Park, quarry, 38°59.1'N, 77°14.8'W, Malaise trap, 30.x-28.xi.2007, D.R. Smith (1♂, USNM).

###### Comments.

*Calycomyzamalvae* can be recognised by sometimes having three thick setae on the mid tibia, its characteristic basally subspherical distiphallus, and ejaculatory apodeme.

##### Calycomyza orientalis

Taxon classificationAnimaliaDipteraPhytomyzinae

Spencer

Figs 478 , 479

Calycomyza orientalis Spencer, in Spencer and Steyskal 1986b: 301.

###### Description.

Wing length 2.3–2.7 mm (♂), 2.7 mm (♀). Length of ultimate section of vein M4 divided by penultimate section: 2.3–2.7. Eye height divided by gena height: 2.5–2.6. First flagellomere circular. Notum subshiny.

***Chaetotaxy***: Setae relatively stout, including dark brown orbital setulae. Two ori, sometimes with additional ori present anteriorly; two ors. Postocellar and ocellar setae both long, well-developed. Two well-developed dorsocentral setae, setula in front of anterior dorsocentral slightly enlarged. Six rows of acrostichal setulae. Two posteromedial setae on mid tibia.

***Colouration***: Setae dark brown. Head yellow with back of head, clypeus, palpus, ventral margin of gena, ocellar tubercle and antenna dark brown; frons dark brown in posterolateral corner and along orbital plate to anterior ors or slightly past level of posterior ors; with minute brown spots at base fronto-orbitals anterior to stripe; dorsal 1/2 of face brown. Thorax dark brown with postpronotum (excluding dark anteromedial spot confluent with margin), notopleuron (excluding dark elongate sublateral spot) and small anterolateral spot behind suture yellow. Halter white. Calypter margin and hairs golden yellow with margin sometimes brownish. Legs and abdomen dark brown.

***Genitalia***: (Figs 478, 479) Epandrium and surstylus with dense patch of tubercle-like setae on inner-distal margin. Hypandrium with one seta on broadly rounded and partially membranous inner lobe. Postgonite bare. Basiphallus sclerotised dorsolaterally and anteromedially, slightly produced on left lateral margin. Hypophallus approximately as long as mesophallus, subrectangular with apex downcurved, nearly parallel-sided. Paraphallus 2/3 length of mesophallus, abruptly widened on distal 1/3, inner margin ill-defined. Mesophallus narrow, dark, subcylindrical with slight ventral curve at apex, broader and rounded at base, and slightly widening apically; small narrow floating sclerite near ventroapical margin of mesophallus. Distiphallus dark, short inner structures concealed, widest when viewed laterally with rounded apex and narrower stem (~ 2/3 width of distal part); when viewed ventrally, stem-like basal section narrowing to midpoint and distal section abruptly widened.

###### Host.

Unknown, but adult collected ex *Rubusalleghaniensis* (Rosaceae).

###### Distribution.

**USA**. ME, NY, TN, VA.

###### Type material.

***Holotype*: USA. VA**: Mountain Lake, Giles Co., 37°21'36"N, 80°32'2"W, 1158 m, ex blossoms *Rubusalleghaniensis*, 28.v.1962, J.G. Chillcott, CNC1105015 (1♂ [genitalic dissection missing], CNC).

***Paratypes*: USA. NY**: Suffolk Co., 30.v.1964, M.I. Blenderman, CNC1150956 (1♂, CNC), **TN**: Chapin Sanctuary, East Ridge, 9.v.1952, O. Peck, CNC1150955 (1♂, CNC), **VA**: Giles Co., Mountain Lake, 1158 m, 31.v.1962, J.G. Chillcott, CNC1150957 (1♂, CNC), ex blossoms *Rubusalleghaniensis*, 28.v.1962, CNC1150958 (1♀, CNC).

##### Calycomyza platyptera

Taxon classificationAnimaliaDipteraPhytomyzinae

(Thompson)

Figs 472–474

Agromyza platyptera Thomson, 1868: 608. Melander 1913: 257; Malloch 1913: 293; Frost 1924: 50; Frick 1956: 288 [as synonym of *jucunda* Wulp].
Calycomyza platyptera . Steyskal, 1973a: 191; Spencer and Steyskal 1986b: 142; Martinez and Etienne 2002: 30; Diaz et al. 2015: 390; Scheffer and Lonsdale 2018: 86; Eiseman and Lonsdale 2018: 34; Eiseman et al. 2019: 308; Monteiro et al. 2019: 167.

###### Description.

Wing length 1.5–1.8 mm (♂), 1.6–2.2 mm (♀). Length of ultimate section of vein M4 divided by penultimate section: 2.4–3.1. Eye height divided by gena height: 3.7–5.1. First flagellomere circular. Notum subshiny to variably pruinose.

***Chaetotaxy***: Two ori; two ors. Ocellar seta relatively thin and ~ 2/3 length of postocellar. Two well-developed dorsocentral setae. Acrostichal setulae in six straight to irregular rows. Two posteromedial setae on mid tibia, with one or both reduced to absent.

***Colouration***: Setae dark brown. Head yellow with back of head, clypeus, palpus, ocellar tubercle and antenna dark brown; frons brown in posterolateral corner to base of posterior ors along fronto-orbital plate; dorsal 1/2 of face variably brown Thorax dark brown with postpronotum (excluding dark anteromedial spot confluent with margin), notopleuron (excluding dark elongate sublateral spot) and small anterolateral spot behind suture yellow. Halter white. Calypter margin and hairs white. Legs and abdomen dark brown.

***Genitalia***: (Figs 472–474) Epandrium and surstylus with dense patch of tubercle-like setae on inner-distal margin. Inner lobe of hypandrium surrounding outer margin of postgonite and with one seta. Basiphallus broadly sclerotised on dorsal/left-lateral surface with apical corner on left side well-sclerotised and distinctly pointed. Hypophallus ca. as long as basiphallus, made up of one pair of narrow, irregular, rod-like sclerites. Paraphalli ill-defined, elongate, and pale with base darker; diverging at base and nearly parallel apically, fused to transverse bar emerging from base of mesophallus in front of point of duct insertion. Mesophallus slightly longer than basiphallus with base especially bulbous, contrasting longer, more slender sclerite in C. *humeralis* and *C.solidaginis*; small medial sclerite between mesophallus and distiphallus present. Distiphallus divided into two short tubules that have a dorsoventrally flattened “stem” that is marked laterally by a subbasal constriction; distal section slightly angled dorsally, relatively pale and with thin cover enclosing small fringed inner structure. Ejaculatory apodeme with stout asymmetrical base grading into sclerotised origin of duct and sclerotised bar across sperm pump; stem stout, blade not especially large, pale marginally.

###### Hosts.

Asteraceae – *Ambrosia*, *Arctium*, *Artemisia*, *Aster*, *Baccharis*, *Bidens*, *Conyza*, *Cynara*, *Erechtites*, *Erigeron*, *Eupatorium*, *Gaillardia*, *Gamochaeta*, *Grindelia*, *Helenium*, *Helianthus*, *Heterotheca*, *Iva*, *Mikania*, *Senecio*, *Silphium*, *Solidago*, *Symphyotrichum*, *Tagetes*, *Tithonia*, *Xanthium*, *Zinnia* (Spencer and Steyskal 1986b; Spencer 1990; Benavent-Corai et al. 2005; Eiseman and Lonsdale 2018). The rearing record of “*Aplopappussquarrosa*” below, originally noted in Frost (1924), corresponds to *Hazardiasquarrosa* (C. Eiseman, pers. comm.).

###### Distribution.

**Canada**: MB, NS, ON, SK. **USA**: CA, CO, FL, GA*, IA, IL*, IN*, KS, LA*, MA, MD*, MI*, MO*, MS, NC, OH*, OK, TN*, TX*, VA*, WV*. Argentina. Brazil. Cuba. Grand Cayman. Ecuador. Guadeloupe. Jamaica.

###### Type material.

***Holotype* [*platyptera*]: USA. CA** (1♀, NRS). [Not examined]

###### Material examined.

**Canada. MB**: Aweme, 4.ix.1912[?], N. Criddle (1♀, USNM), Brandon, 1.vii.1946, P.H. Westdal, coll. on potato (1♂, CFS), **ON**: Wellington Co., Stone Rd E, 16.ix.1991, Malaise, R.A. Cannings (1♂, RBCM), Moose Factory, 10.vi.1949, D.P. Whillans, CNC391482 (1♂, CNC), **SK**: Sintaluta, 23.vii.1987, A. Paton, reared from *Ivaxanthifolia*, CNC391466, CNC391467 (1♂ 1♀, CNC). **Grand Cayman**: Georgetown, 15–30.iii.1965, J.R. McLintock, Malaise trap, CNC391471 (1♀, CNC). **USA. CA**: Los Angeles Co., “ace 255”, ex. *Zinnia* leaf blotches, 1.ix.1940, R.M. Bohart (1♀, USNM), Los Angeles, leaf-miner of *Aplopappussquarrosa*, bred in June, Coquillett (2♀, USNM), Los Angeles Co., Coquillett (1♂ 1♀, USNM), Napa/Lake Co., McLaughlin Reserve, 13.vii.2015, E. LoPresti and C.S. Eiseman, *Helianthusexilis* em. 20.vii-11.viii.2015, #CSE1811, CNC564639–564646 (4♂,4♀, CNC), **CO**: Boulder, 1828 m, 4.vi.1961, B.H. Poole, CNC391469 (1♀, CNC), **FL**: Orlando, 28.ii.1918, “GGA”, J.M. Aldrich (1♀, USNM), Bade Co., farm near Royal Palm Hammock, 4.xii.1961, Munroe, Holland and Chillcott, CNC391477 (1♂, CNC), Chattahoochee, 26.iv.1952, O. Peck, CNC391480 (1♀, CNC), Everglades N.P., Paradise Key, 30.iii.1953, W.R.M. Mason, CNC391472 (1♂, CNC), Gainesville, 26.iv.1952, O. Peck, CNC391481 (1♂, CNC), Homestead, 4.iv.1952, J.R. Vockeroth, CNC391474 (1♂, CNC), Key Largo, 5–6.xii.1961, Munroe, Holland and Chillcott, mercury vapour light, CNC391473 (1♂, CNC), Miami-Dade Co., Redlands, 25°31'N, 80°30'W, 7.xi.2011, R. Diaz and J. McClurg, reared from leaves of *Mikaniamicrantha*, CNC391491, CNC391490 (1♂ 1♀, CNC), Pasco Co., Moon L., 16.iv.1952, J.R. Vockeroth, CNC391479 (1♂, CNC), Quincy, 13.v.1964, [K.A. Spencer], mine *Xanthium*, em. 18–26.v.1964, CNC391476 (2♂/♀, CNC), Sanford, 12.vi.1964, [K.A. Spencer], ex *Ambrosiaartemisifolia*, CNC391478 (2♂, CNC), St. Lucie Co., Fort Pierce, U of FL quarantine, greenhouse #3, 27°26'N, 80°25'W, 10.xi.2011, R. Diaz and J. McClurg, colony, CNC391485–391489 (1♂ 4♀, CNC), St. Lucie Co., Fort Pierce, near U of FL campus, 27°26'N, 80°25'W, 12.xi.2011, R. Diaz and J. McClurg, reared from leaves of *Mikaniascandens* CNC391492–391494 (1♂ 2♀, CNC), E.D. Bennett, leaf miner *Baccharishalimifolia*, Lakewales, v.1960 (1?, USNM), North Fort Charlotte (1♂, USNM), O'Neil (1♂ 1♀, USNM), Summerville S.C. (1♂, USNM), Daytona Beach, v.1960, F.D. Bennett (1♀, USNM), **GA**: Richmond Hill, v.1960, F.D. Bennett, coll. as adult on *Baccharishalimifolia* (1♀, USNM), Tifton, x.1896, J.M. Aldrich (1♂, USNM), **IA**: Howard Co., Hayden Prairie State Preserve, 15.vii.2015, C.S. Eiseman, *Silphiumperfoliatum*, em. by 19–23.vii.2015, #CSE1743, CNC654359, CNC654360 (1♂ 1♀, CNC), **IL**: K-hook, “6-7.32”, hack, “262 amb.”, ex. *Ambrosiatrifina*, “Lawson, ‘33”, **IN**: Logansport, 10.viii.1915, J.M. Aldrich (1♀, USNM), Lafayette, J.M. Aldrich, 10.iv.1915 (1♀, USNM), v.1927 (2♀, USNM), “x.13” (2♂, USNM), “x.15” (2♂, USNM), “x.16” (1♀, USNM), Lafayette, J.M. Aldrich, from star-shaped mine in burdock, 29.ix.1913 (1♀, USNM), Shelby, 24.v.1914, J.M. Aldrich (1♀, USNM), **KS**: Riley Co., Konza Prairie, 3.vii.2015, C.S. Eiseman, *Helianthusannuus* em. 13.vii.2015, #CSE1702, CNC564652 (1♀, CNC), Medora, sand dunes, 17.iv.1982, G.W. Sabrosky (1♀, USNM), Manhattan, D.A. Wilbur, 17.x.1933 (1♀, USNM), 2.v.1930 (1♀, USNM), 8.x.1930 (1?, USNM), 17.ix.1933 (1♂ 1♀, USNM), **LA**: Baton Rouge, 28.v.1917, collected on Globe artichoke, T.H. Jones, Chittenden No. 4257-1, issued iv.1917 (1♀, USNM), issued iii.17 (1♂, USNM), issued 12.iii.1917 (1♂, USNM), Lake Charles, 9.vi.1917, J.M. Aldrich (2♀, USNM), **MA**: Franklin Co., Northfield, 263 Capt. Beers Plain Rd., 11.vii.2012, C.S. Eiseman, ex *Ambrosiaartemisiifolia*, em. 14.vii.2012, CNC391483,CNC391484 (2♀, CNC), **MD**: Cabin john Bridge, 28.iv, “19/2”, Knab and Malloch (1♀, USNM), P.G. Co., Camp Springs, 16.vii.1979, Malaise trap, G.F. Hevel (1♀, USNM), Colesville, 4.vi.1977, W.W. Wirth (1♀, USNM), Cabin John, “x-21”, J.M. Aldrich (1♂, USNM), **MI**: S Haven, 23.vi.1938, C.W. Sabrosky (1♀, USNM), Nottawa, 8.vi.1941, C.W. Sabrosky (1♀, USNM), Hart, 20.vi.1989, C.W. Sabrosky (1♀, USNM), E Lansing, 29.vii.1941, C. Sabrosky (1♀, USNM), E Lansing, 6.viii.1941, B. Wilson (1♀, USNM), St. Joseph, 28.vi.1942, C.W. Sabrosky (1♂, USNM), Manhattan, 1.x.1933, H.M. Smith (1♂, USNM), **MO**: McDonald Co., nr. Lanagan, 8.v.1984, G.F. and J.F. Hevel (1♀, USNM), **NC**: Carteret Co., Atlantic Beach, 3–4.ix.1986, G.F. and J.F. Hevel (1♂ 3♀, USNM), **OH**: Champion Co., Kiser Lake S.P., 40°11.6'N, 83°58.8'W, 5.ix.1976, S.A. Steinly (1♀, USNM), **OK**: Payne Co., Mehan, 20.v.2016, M.W. Palmer, *Silphiumlaciniatum*, em. 1.vi.2016, #CSE2666, CNC654004 (1♂, CNC), **TN**: East Ridge, 9.v.1952, O. Peck, CNC391470 (1♂, CNC), **TX**: Welder Wildlife Ref. nr Sinton, 19–23.iii.1965, J.G. Chillcott, CNC391468 (1♂, CNC), Clarendon, 19.ix.1905, on Grindeliasquarrosa, “9/23/05, VI ia”, Hunter No. 108a, W.D. Pierce (1♀, USNM), Colemon, 6.xi.1936, R.H. Painter (1♂, USNM), **VA**: Arlington, 23.viii.1906, leaf mine on *Zinnia*, issued 25.viii.1926 (1♀, USNM), issued 24.viii.1926 (1♂ 1♀, USNM), Great Falls, 21.vi.1931, A.L. Melander (3♀, USNM), **WV**: Morgan Co., nr. Great Cacapon, 3.vii.1983, G.F. and J.F. Hevel (1♀, USNM).

##### Calycomyza promissa

Taxon classificationAnimaliaDipteraPhytomyzinae

(Frick)

Figs 480–483

Phytobia (Calycomyza) jucunda . Misidentification, in part. Frick 1952a: 395.
Phytobia (Calycomyza) promissa Frick, 1956: 287.
Calycomyza promissa . Spencer, 1969: 153; Spencer and Steyskal 1986b: 143; Scheffer and Lonsdale 2018: 86; Eiseman and Lonsdale 2018: 35.

###### Description.

Wing length 1.8–2.1 mm (♂), 2.3–2.6 mm (♀). Length of ultimate section of vein M4 divided by penultimate section: 2.1–2.6. Eye height divided by gena height: 5.0–5.9. First flagellomere circular (one female with angulate segment reported in Spencer 1969). Thorax with light pruinosity.

***Chaetotaxy***: Two ori; two ors. Ocellar seta stout and straight, with length ranging from that of tubercle to that of postocellar. Two large, well-developed dorsocentrals posteriorly, third dorsocentral 1/2–4/5 length of second dorsocentral, fourth dorsocentral sutural and barely larger than surrounding setule. Six rows of acrostichal setulae. Usually two posteromedial setae on mid tibia, sometimes one.

***Colouration***: Setae dark brown. Head yellow with back of head, clypeus, palpus, ocellar tubercle and antenna dark brown; male frons brownish in posterolateral corner to just past posterior ors along fronto-orbital plate, female with posterolateral spot extending to midpoint between vertical setae and ocellar tubercle, distant from base of posterior ors; posteroventral margin of gena brown. Thorax dark brown with postpronotum (excluding dark anteromedial spot confluent with margin), notopleuron (excluding dark elongate sublateral spot) and small anterolateral spot behind suture yellow. Calypter white. Halter white. Legs and abdomen dark brown.

***Genitalia***: (Figs 480–483) Epandrium and surstylus with dense patch of tubercle-like setae on inner-distal margin. Hypandrium with two setae on inner lobe. Postgonite bare and simple with broadly rounded medial process. Basiphallus sclerotised along apical and left lateral margins. Paraphallus narrow, pointed, exclinate. Hypophallus broad and ill-defined with J-shaped medial sclerite. Mesophallus short, wide, and broadly rounded. Distiphallus with outer cover pale, bulging medially and with slight apical flare, and inner process small, broad, and short-haired. Ejaculatory apodeme very broad, well-sclerotised, stem short, base confluent with sclerotised base of duct and margin of sperm pump.

###### Hosts.

Asteraceae – *Symphotrichum*; possibly also *Ampelaster* (Eiseman and Lonsdale 2018).

###### Distribution.

**Canada**: MB, ON. **USA**: CA, CO, DE*, FL (leaf mines and females), KS*, LA, MA, MI*, NC, NY, OK. Widespread from southern Canada to FL and CA (Spencer 1990).

###### Type material.

***Holotype*: USA. CA**: Alameda Co., Albany, 28.ix.1948, K.E. Frick, reared from a larva mining a leaf of *Asterchilensis* (1♂, USNM).

***Paratype*: USA. CA**: Alameda Co., Albany, K.E. Frick, reared from a larva mining a leaf of *Asterchilensis*, 21.vii.1948 (1♂, USNM), 28.viii.1948 (1♂ 1♀, USNM).

###### Additional material examined.

**Canada. MB**: Aweme, N. Criddle, 27.viii.1917, CNC391562 (1♂, CNC), 28.viii.1917, CNC391563 (1♀, CNC), **USA. CO**: Park Co., High Creek Fen, 13.vii.2015, C.S. Eiseman, Symphotrichumcf.lanceolatum em. 25.vii.2015, #CSE1828, CNC564673 (1♂, CNC), Larimer Co., Estes Park, 1.ix.2015, N.D. Charney, *Symphyotrichum*, #CSE2041, CNC654334 (1♂, CNC), **DE**: Newark, 31.v.1974 (1♂, USNM), **KS**: Manhattan, D.A. Wilbur, 24.x.1930 (1♂, USNM), **LA**: Alexandria, 11 mi SW, J.G. Chillcott, 21.iii.1960, CNC391564, CNC391565 (2♂, CNC), **MA**: Berkshire Co., Stockbridge, Agawam Lake, 14.viii.2017, em. 20.viii.2017, C.S. Eiseman, ex *Symphyotrichumpuniceum*, #CSE4152, CNC939711 (1♀, CNC), Franklin Co., Northfield, 276 Old Wendell Rd., 4.viii.2017, em. 10.viii.2017, C.S. Eiseman, ex *Symphyotrichumlateriflorum*, #CSE4101, CNC939663 (1♂, CNC), **MI**: Midland, 6–7.vi.1936, C.W. Sabrosky (1♂, USNM), **NC**: Durham Co., Durham, 20.v.2015, T.S. Feldman, *Symphotrichum*, em. by 25.v.2015, #CSE1581, CNC564611, CNC564612 (1♂ 1♀, CNC), Scotland Co., Laurinburg, St. Andrews University, 15.v.2015, T.S. Feldman, *Symphotrichum*, em. v.2015, #CSE1563, CNC564633 (1♂, CNC), 17.viii.2015, T.S. Feldman, Symphotrichumcf.pilosum, em. 18.viii.2015, #CSE2122, CNC564666 (1♀, CNC), Scotland Co., Laurinburg, St. Andrews University, 11.v.2016, T.S. Feldman, *Symphyotrichumpilosum*, em. 18.v.2016, #CSE2469, CNC653957 (1♀, CNC), 15.ix.2015, T.S. Feldman and C.S. Eiseman, *Symphyotrichumcf.pilosum*, em. 24-28.ix.2015, #CSE2098, CNC653942, CNC653943 (2♂, CNC), x.2015, T.S. Feldman, *Symphyotrichum*, em. by xi.2015, #CSE2160, CNC653937 (1♀, CNC), **OK**: Payne Co., Mehan, 23.iii.2016, M.W. Palmer, *Symphyotrichumdrummondii*, em. by iv.2016, #CSE2657, CNC653987 (1♂, CNC), 24.iii.2016, M.W. Palmer, *Symphyotrichumoolentangiensis*, em. 30.iii.2016, #CSE2659, CNC653995 (1♀, CNC), Payne Co., Mehan, 36°0'51.62"N, 96°59'48.28"W, 6.vi.2016, M.W. Palmer, *Symphyotrichumericoides*, em. by 17.vi.2016, #CSE2596, CNC634974, CNC634975 (2♂, CNC).

###### Tentatively identified material.

**USA. FL**: Lake Co., Alexander Springs, 26.iii.2013, em. 12–18.iv.2013, C.S. Eiseman, ex *Ampelastercarolinianus*, #CSE270, CNC384821–384824 (4♀, CNC).

##### Calycomyza solidaginis

Taxon classificationAnimaliaDipteraPhytomyzinae

(Kaltenbach)

Figs 470 , 471

Agromyza solidaginis Kaltenbach, 1869: 196.
Dizygomyza solidaginis . Hering 1925: 133, 1927: 56.
Dizygomyza (Calycomyza) solidaginis . Hendel 1931: 69; Hering 1951: 42.
Phytobia (Calycomyza) solidaginis . Frick 1953: 70, 1956: 292.
Dizygomyza bellidis . Misidentification, in part. Hendel 1920: 136, 1923: 394.
Calycomyza solidaginis . Spencer, 1969: 155; Spencer and Steyskal 1986b: 145; Boucher and Wheeler 2001: 614; Scheffer et al. 2007: 771; Scheffer and Lonsdale 2018: 86; Eiseman and Lonsdale 2018: 36.

###### Description.

Wing length 1.6–2.0 mm (♂), 2.0–2.3 mm (♂). Length of ultimate section of vein M4 divided by penultimate section: 2.1–2.9. Eye height divided by gena height: 1.8–3.6. First flagellomere with shallow angle on anterodorsal margin. Notum subshiny to heavily dusted.

***Chaetotaxy***: Two ori; two ors. Ocellar seta sometimes thinner and/or shorter than postocellar. Two well-developed dorsocentral setae, setula in front of anterior dorsocentral small to long and well-developed, sometimes reaching 2/3 length anterior dorsocentral. Six rows of acrostichal setulae. One posteromedial seta on mid tibia.

***Colouration***: Setae dark brown. Head yellow with back of head, clypeus, palpus, ocellar tubercle and antenna dark brown; frons brownish in posterolateral corner; lateral margin of frons sometimes either with narrow brown infuscation along fronto-orbital plate that occasionally extends to encompass base of fronto-orbitals, or with brown stripe extending from vertical setae to posterior or anterior ors; dorsal margin to dorsal 1/3 of face brown. Thorax dark brown with postpronotum (excluding dark anteromedial spot confluent with margin), notopleuron (excluding dark elongate sublateral spot) and small anterolateral spot behind suture yellow. Calypter white. Halter white. Legs and abdomen dark brown.

***Genitalia***: (Figs 470, 471) Epandrium and surstylus with dense patch of tubercle-like setae on inner-distal margin. Hypandrium and postgonite as in *C.humeralis*. Basiphallus sclerotised along dorsal/left-lateral surface. Hypophallus with one pair of long, narrow sclerites approximately as long as basiphallus with irregular outline and small inner-basal process. Paraphallus rod-like with base wider and darker; diverging, angled anteroventrally; joined basally by narrow transverse sclerite. Mesophallus as long as basiphallus, slightly bulbous basally, with dark basal plate in front of point of duct insertion; small medial sclerite between mesophallus and distiphallus present. Distiphallus entirely divided into two short tubules, dark, very closely spaced; base stem-like (lateral view) and most of distal region broader, cup-like, angled slightly dorsally; inner fringed structure slightly emerging apically. Ejaculatory apodeme similar to that of *C.platyptera*.

###### Hosts.

Asteraceae – Erigeronglabellus*, *Solidago*, Canadanthusmodestus*, Symphyotrichumciliolatum*.

###### Distribution.

**Canada**: AB*, NB*, NS, ON, QC, YT. **USA**: widespread outside of AK and HI. Europe. Russia*. China, Yemen (Papp and Černý 2017).

###### Type material.

***Syntypes*: Austria** [not given]. [Types from Germany lost (Spencer 1981)]

###### Material examined.

**Canada: AB**: Killen, 13.iv.1941, A. Stone (1♂, USNM), Kananaskis, 0.5 km SE Barrier Lk. Univ. Fld. Stn., 2.viii.2001, 51°02'N, 115°02'W, C.J. Borkent (1♂, RBCM), C.E. Lee Sanctuary, Devon Sandhills, on *Erigeronglabellus*, larva collected 8.viii.1980, emerged 28.viii.1980, G.C.D. Griffiths, E432 (1♀, UASM), larva collected 9.vi.1980, emerged 20.vi.1980, G.C.D. Griffiths (1♀, UASM), Elk Island Nat. Park, on *Astermodestus*, puparium collected 20.vii.1975, emerged 1.viii.1975, G.C.D. Griffiths, E260 (1♂ 2♀, UASM), Swan Hills, 54°42'N, 115°49–50'W, 3700–3750 ft, on *Asterciliolatus*, puparium collected 7.viii.1977, emerged 26.viii.1977, G.C.D. Griffiths, SW79 (1♀, UASM), emerged 17.viii.1977 (1♂, UASM), **NB**: Pt. Lepreau, 10.viii.1956, A.H. Sturtevant (1♀, USNM), **NS**: Bridgetown, 29.viii.1912, [illegible], CNC391417 (1♀, CNC), Truro, 5.ix.1973, CNC391424 (1♂, CNC), **ON**: Ancaster, 22.vii.1946, T.N. Freeman, CNC391420 (1♀, CNC), Grand Bend, 10.vii.1939, G.E. Shewell, CNC391461, CNC391423, CNC391427 (2♂ 1♀, CNC), 11.vii.1939, G.E. Shewell, CNC391419 (1♂, CNC), 12.vii.1939, G.E. Shewell, CNC391418 (1♀, CNC), Harwood Plains, Constance Bay Rd., 19.vii.1967, G.E. Shewell, CNC391428, CNC391429 (2♀, CNC), Ottawa, 16.ix.1952, J.R. Vockeroth, CNC391426 (1♂, CNC), 22.v.1946, A.R. Brooks, CNC391425, CNC391561 (1♂ 1♀, CNC), 30.viii.1963, O. Peck, CNC391465 (1♀, CNC), Rockliffe, E of, 18.viii.1941, O. Peck, CNC391464 (1♀, CNC), Simcoe, 14.vi.1930, G.E. Shewell, CNC391463 (1♀, CNC), 23.vi.1930, G.E. Shewell, CNC391462 (1♀, CNC), St. Lawrence Is. Nat. Park, Grenadier I. Centre, 24.vi.1975, B.E. Cooper, Code 220M-15, CNC391430 (1♀, CNC), St. Lawrence Is. Nat. Park, McDonald Is., 4.ix.1976, W. Reid, Code 4620-T, CNC391444 (1♀, CNC), **ON**: Wellington Co., Stone Rd E, Malaise, R.A. Cannings, 21.ix.1991 (1♂, RBCM), 2.vii.1992 (1♀, RBCM), 5.vii.1992 (1♂, RBCM), 7.x.1992 (1♀, RBCM), **QC**: Ottawa River, 13.vii.1967, [K.A. Spencer], CNC391422 (1♂, CNC). **RUSSIA**. Moscow Oblast: Moskau-Wladykino, mine an *Solidagovirgaurea*, 27.vi.1956, Buhr, 1175, CNC391421 (2♀, CNC). **USA. AZ**: Cochise Co., Rustler park, 9.vi.1972, W.W. Wirth (1♀, USNM), **CA**: Meyers Sta., 10.viii.1921, 7000', A.H. Sturtevant (1♀, USNM), Riverside, 22.ii.1955, A.L. Melander (1♂, USNM), Lakeside Tahoe, 29.vi.1929, J.M. Aldrich (1♂, USNM), Mentone, 13.v.1953, A.L. Melander (1♂, USNM), **CO**: Tenn. Pass, JMA, 23.vii.1917 (1♀, USNM), 24.vii.1917 (1♀, USNM), **CT**: Goose Island, 21.vii.1913 (1♀, USNM), Doolittle Ranch, Mt. Evans, 2987 m, poplar woods, 22.vii.1961, J.G. Chillcott, CNC391446 (1♀, CNC), Idaho Springs, 3mi SW, 2438 m, 27.vii.1961, C.H. Mann, CNC391449 (1♀, CNC), Jackson Co., Muddy Pass, 2682 m, 15.viii.1961, B. Poole, CNC391443 (1♂, CNC), Loveland Pass, W slope, 3002 m, 28.vii.1961, J.G. Chillcott, CNC391448 (1♀, CNC), 8.viii.1961, B.H. Poole, CNC391447 (1♀, CNC), **DC**: Washington, “AP”, 21.x.1956, P.H. Arnaud, Jr. (2♂, USNM), Washington, 17.viii.1913, A.L. Melander (1♀, USNM), **FL**: Jackson Co., Florida Caverns St. Park, 26.v.1973, Malaise trap, W.W. Wirth (1♀, USNM), Leon Co., Tall Timber Res. Sta., 29.v.1973, Malaise trap, W.W. Wirth (1♀, USNM), **GA**: Waynesboro, 26.v.1943, leaf-miner of goldenrod, emerged 7.vi.1943, C.H. Hoffman (1♂, USNM), **IA**: Winneshiek Co., Decorah, Community Prairie, 43°18'5.04"N, 91°48'6.60"W, 18.vi.2017, J. van der Linden, *Solidago*, em. vii.2017, #CSE4949, CNC1288659 (1♂, CNC), **ID**: Chateolet, viii.1915, A.L. Melander (1♀, USNM), **IN**: Lafayette, 30.iv.1915, swept from grass, J.M. Aldrich (1♀, USNM), Lafayette, J.M. Aldrich, 15.iv.1915 (1♀, USNM), “x-15” (1♂, USNM), “x-13” (1♂, USNM), **MA**: Vineyard Haven, 17.viii.1954, A.H. Sturtevant (1♂, USNM), Woods Hole, viii.1922, A.H. Sturtevant (1♂, USNM), Forest Hills, 22.ix.1913, A.L. Melander (1♀, USNM), Nantucket Co., Nantucket, Dead Horse Valley, 6.viii.2012, C.S. Eiseman, ex *Solidago* leaf mine, em. 15.viii.2012, #CSE32, CNC391435–391439 (4♂ 1♀, CNC), Nantucket Co., Nantucket, Ice Pond Lot, 26.vii.2014, C.S. Eiseman, ex. *Solidagolatissimifolia*, em. 5.viii.2014, #CSE1269, CNC384825, CNC384826 (2♀, CNC), 5.viii.2012, em. 8–15.viii.2012, #CSE22, CNC391431–391434 (3♂ 1♀, CNC), Nantucket Co., Nantucket, Squam Swamp, 12.vi.2013, C.S. Eiseman, ex. *Solidagolatissimifolia*, em. 23.vi.2013, #CSE587, CNC384812 (1♀, CNC), Nantucket Co., Tuckernuck Island, 14.vii.2012, C.S. Eiseman, J.A. Blyth, leaf mine in *Solidago*, em. 22.vii.2012, #CSE58, CNC391440 (1♂, CNC), **MD**: Montgomery Co., Colesville, 4.ix.1977, Malaise trap, W.W. Worth (1♂, USNM), P.G. Co., Camp Springs, 25.viii.1979, G.F. Hevel (1♂, USNM), **MI**: E Lansing, 20.v.1939, C. Sabrosky (1♀, USNM), Leelanau Co., 22.vi.1937, C. Sabrosky (1♂, USNM), Mio, 29.v.1937, H. Millron (2♀, USNM), **NC**: Hyde Co., 3mi E Swan Quarter, 19.iv.1981, G.F. and J.F. Hevel (2♂, USNM), Base of Wayah Bald, 10.viii.1957, W.R. Richards, CNC391442 (1♂, CNC), Bubbling Spring Creek, nr. Tennessee Bald, 1554 m, 17.vii.1957, J.G. Chillcott, CNC391445 (1♀, CNC), **SC**: Aiken, 13.vi.1957, J.R. Vockeroth, CNC391441 (1♂, CNC), Coosawhatche, 1.i.1972, J.R. Vockeroth, CNC391450 (1♀, CNC), **NC**: Scotland Co., Laurinburg, St. Andrews University, 31.iii.2016, T.S. Feldman, *Solidagocanadensis*, em. 12–15.iv.2016 , #CSE2374, CNC653966–653969 (4♂, CNC), **NH**: Mt. Wash., Alpine Garden, 30.viii.1957, A.L. Melander (1♂, USNM), **NJ**: Morristown, 1.v.1925, A.H. Sturtevant (1♂, USNM), **NY**: L.I., Cold Spring Harbor, July, A.L. Melander (2♂ 2♀, USNM), Bear Mt., 31.v.1941, A.L. Melander (1♂, USNM), **OH**: Indian Cr. C.P., nr. Oxford, 25.v.1976, S.A. Steinly (1♂, USNM), Woodside, 8.vi.1940, A.L. Melander (1♂, USNM), **OR**: Mt. Hood, 29.vii.1921, A.L. Melander (1♀, USNM), **PA**: York Co., 2mi W of Airville, 29.viii.1981, G.F. and J.F. Hevel (1♀, USNM), **SC**: Florence, “6-1-43”, emerged 5.vi.1943, leaf-miner of goldenrod (1♀, USNM), **UT**: Timpanogos Mt., 25.vi.1940, A.L. Melander (1♂, USNM), **VA**: Alexandria, v.1915, J.M. Aldrich (1♀, USNM)reene (1♀, USNM), Great Falls, 8.vi.1938, C.T. Greene (1♀, USNM), **WY**: Yellowstone Park, W Craigs Pass, 7900', 8.viii.1918 (1♂, USNM), Yellowstone Park, 9.viii.1918, A.L. Melander (1♂, USNM), Yellowstone Park, Biscuit Basin, 2.viii.1934, A.L. Melander (1♂, USNM).

##### Calycomyza verbenae

Taxon classificationAnimaliaDipteraPhytomyzinae

(Hering)

Figs 484–489

Agromyza jucunda . Misidentification, in part. Coquillett 1898: 77.
Dizygomyza (Calycomyza) verbenae Hering, 1951: 42.
Phytobia (Calycomyza) verbenae . Frick, 1953: 70, 1956: 300.
Calycomyza verbenae . Spencer and Steyskal 1986b: 151; Martinez and Etienne 2002: 30; Eiseman and Lonsdale 2018: 36.

###### Description.

Wing length 1.8–2.1 mm (♂), 2.0–2.4 mm (♀). Length of ultimate section of vein M4 divided by penultimate section: 1.8–3.0. Eye height divided by gena height: 4.5–5.4. First flagellomere circular. Notum subshiny.

***Chaetotaxy***: Two ori; two ors. Postocellar and ocellar setae well-developed. Two well-developed dorsocentral setae, setula in front of anterior dorsocentral thin and nearly 1/2 the length of that seta, appearing as third dorsocentral. Six rows of acrostichal setulae. Two posteromedial setae on mid tibia.

***Colouration***: Setae dark brown. Head yellow with back of head, clypeus, palpus, ocellar tubercle and antenna dark brown; frons brownish in posterolateral corner with faint stripe extending from posterior margin of frons to base of posterior ors Thorax dark brown with postpronotum (excluding dark anteromedial spot confluent with margin), notopleuron (excluding dark elongate sublateral spot), small anterolateral spot behind suture yellow, and scutum faintly to distinctly yellowish on suture near sides of scutellum. Halter white. Calypter margin brownish and hairs brown. Legs and abdomen dark brown with fore knee yellow.

***Genitalia***: (Figs 484–489) Epandrium and surstylus with dense patch of tubercle-like setae on inner-distal margin. Hypandrium with one seta on broad inner lobe. Postgonite large, broad, rounded apically. Basiphallus sclerotised along apical and left lateral margins. Paraphallus absent. Hypophallus broad, wider apically and with sclerotised lateral margins. Mesophallus relatively broad with base bulbous and with strong ventral curve subapically. Distiphallus medially divided with halves closely adjoined, very dark; outer cover broadly rounded, slightly angled dorsally, enclosing dark inner process that slightly protrudes. Ejaculatory apodeme stout, typical of genus.

###### Hosts.

Verbenaceae – *Glandularia*, *Verbena*, *Stachytarpheta* (Frick 1953, Benavent-Corai et al. 2005; Eiseman and Lonsdale 2018)

###### Distribution.

**USA**: AZ, DC, FL, IA, IL, IN, NC, NJ, NM, OH; verification required for GA, MD, MS, NY, PA; leaf mines requiring verification from KS, MA, MD (Eiseman and Lonsdale 2018). Brazil.

###### Type material.

***Holotype*: USA. NM**: State College, near Las Cruces, 7–10.vii.1949, J.R. Eyer, ex. *Verbenahybrida*, cultivated var. (1♀, USNM).

###### Additional material examined.

**USA. DC**: Washington, 23.viii.1928, reared from *Verbena*, W.H. White, Chittenden No. 1084 (2♀ [one with puparium], USNM), Washington, 23.viii.1915, leaf miner on *Verbena*, W.H. White (1♀, USNM), **IL**: Algonquin, Coquillett (1♂, USNM), Chicago, A.L. Melander (1♂, USNM), **IN**: Bluffton, 15.viii (1?, USNM), Bluffton, 1905, *Verbena* leaf miner (1♂ 1♀ 1puparium, USNM), Layfayette, “x-13”, J.M. Aldrich (1♂, USNM), **OH**: Dayton, 13.vi.1906, mining in *Verbena* leaf, N.B. Jervett (2♂, USNM), Dayton, 13.vi.1906, mining in *Verbena* leaf, issued 28.vi.1906 (1♀, USNM), Dayton, 18.vi.1906, mining in *Verbena* leaf, issued 28.vi.1906 (1♀, USNM), **TX**: Llano Co., Enchanted Rock, 15.vi.1953 (1♂, USNM). **State unknown [likely IN**]: *Verbena* leaf miner, “7/30, 82, Agromyzajucunda v.d.Wulp” (1♂ 4♀ [with puparia, all on same pin], USNM), “5465(?), Oscinis malváe Burg.” (3♂ 2♀ [with puparia, all on same pin], USNM), “5465(?), July 20/74”, collection C.V. Riley (1♂, USNM), “214L(?), Oscinismalvae Bur. On Verbena” (2♂ 1♀ 1destroyed, USNM). **No locality data**: *Verbena* leaf miner, 30.vii.1882 (1♂ 4♀ [two with puparia, same pin], USNM), [illegible] (3♂ 2♀ [four with puparia, same pin), USNM], [illegible], 20.vii.1914, C.V. Riley (1♂, USNM), Dept. Noja[?] 35, 31.x.1881 (1♀, USNM), No. 1035, leaf miner on *Verbena*, 1.x.1881 (1♂ [with puparium], USNM), 214L[?], *Oscinismalvae* on *Verbena* (2♂ 1♀ 1?, USNM), Dept. No. 1035, *Oscinismalvae*, on 26.x.1881 (1♂ [with puparium], USNM).

###### Comments.

The genitalia of *Calycomyzaverbenae* are highly similar to those of *C.eupatorivora* Spencer (Spencer and Stegmaier 1973), which is found on *Eupatoriumodoratum* in Jamaica, but possibly also occurs in Brazil and Venezuela (reared from *Alomiafastigata* and *Eupatoriumodoratum*, respectively), although the latter differs in having a broad yellow posterolateral spot on the scutum, a slightly yellowish scape and pedicel, developed paraphalli, a shorter mesophallus that appears nearly ovate in profile, and a distiphallus that is much more strongly angled dorsally.

See comments for *Calycomyzaavira*.

##### Cerodontha

Taxon classificationAnimaliaDipteraPhytomyzinae

Rondani

Odontocera Macquart, 1835: 614. Type species: ChloropsdenticornisPanzer 1806: 104, by original designation. Preoccupied by Audinet-Serville (1833, Cerambycidae).
Cerodontha Rondani, 1861: 10. Type species: ChloropsdenticornisPanzer 1806: 104, by original designation. Nowakowski 1973: 7; Spencer 1969: 109; Spencer and Steyskal 1986b: 87.
Odonthocera . Misspelling. Rondani 1861: 10.
Ceratomyza Schiner, 1862: 434 [unnecessary replacement name for Odontocera]. Type species: ChloropsdenticornisPanzer 1806: 104, by automatic designation. Hendel 1931 [synonymy?].
Cerodonta . Misspelling. Hendel 1920: 114, 1932: 265; Hering 1926: 223.

*Cerodontha* is a speciose and morphologically diverse genus divided into seven subgenera: *Butomomyza*, *Cerodontha* s. s., *Dizygomyza*, *Icteromyza*, *Phytagromyza*, *Poemyza* and *Xenophytomyza*. All of these occur in the Nearctic, although *Phytagromyza* is not yet known from the Delmarva states. Approximately 260 species are known, all of which are leaf miners in the monocot families Cyperaceae, Iridaceae, Juncaceae and Poaceae (Boucher 2010).

*Cerodontha* species have a lunule that is higher than wide, or at least it usually appears to be higher than wide because the broader base of some species is often concealed by the overlapping inner-anterior margin of the fronto-orbital plate. The male genitalia are also characteristic, as the mesophallus is well-developed, stalk-like and fused to a similarly well-developed, long distiphallus with one pair of apical tubules that are secondarily fused in very few species. Furthermore, the venter of the subepandrial sclerite is produced into one pair of long, well-sclerotised processes. This process has often been referred to in the literature as the “Langfortsatz”, which simply means a long process and is here referred to descriptively as the ventral process of the subepandrial sclerite. It has also been referred to as the “upper surstylar lobe” by Zlobin (1995, 2007d) based on similarities to a structure in a Palaearctic *Metopomyza*; this is an understandable interpretation, and still potentially valid because there is certainly confluence between this structure and the fused surstylus/epandrium, and not just the subepandrial sclerite; the point of confluence, however, actually appears to be along the infolded surface of the posteroventral margin of the epandrium, not the surstylus itself. The homology of this structure is still in need of verification, as no intermediate forms appear to exist, and parallel fusion of the epandrium and/or surstylus and the subepandrial sclerite occurres throughout the family, including in the genera *Agromyza*, *Aulagromyza*, *Japanagromyza*, *Metopomyza*, *Nemorimyza*, and *Ophiomyia*. For the purpose of this study, the position of this sclerite and its confluence with the remainder of the subepandrial sclerite proper appears to make homology with this latter structure the most efficient explanation.

##### Cerodontha (Butomomyza)

Taxon classificationAnimaliaDipteraPhytomyzinae

Nowakowski

Cerodontha (Butomomyza) Nowakowski, 1967: 633. Type species Agromyzaangulata Loew, by original designation. Spencer 1969: 110; Nowakowski 1973: 141; Spencer and Steyskal 1986b: 97; Zlobin 2001: 113.

Species in the subgenusButomomyza, as defined by Nowakowski (1967), are recognisable by a lunule that is as wide as high or slightly higher, and at least in the Nearctic species examined here, the acrostichal setae are present and the body is often pale brown to brown. Furthermore, the shape of the lunule is subtriangular with bowed sides and often a pointed apex, and the surface is not textured, but covered with a minute velvety pubescence. Larvae also have one pair of rounded areas flanking the anus that are covered with black cuticular teeth that may reach the posterior spiracles. There are more than two dozen species, mostly in the north temperate regions, where they are known to mine in Cyperaceae and Poaceae (Zlobin 2001).

### ﻿Key to the Delmarva Cerodontha (Butomomyza)

**Table:** 

1	Distiphallus more strongly S-shaped in profile, directed more dorsally than apically; tubules separate, slightly divergent apically (Figs 493, 494). Paraphallus lobate with ventral sclerotisation	***C.angulata* (Loew)**
–	Distiphallus relatively straight, directed apically; tubules parallel, sometimes nearly confluent along length (Figs 503–505). Paraphallus band-like	***C.subangulata* (Malloch)**

#### Species descriptions

##### Cerodontha (Butomomyza) angulata

Taxon classificationAnimaliaDipteraPhytomyzinae

(Loew)

Figs 99 , 490–494 , 495–502

Agromyza angulata Loew, 1869: 47. Melander 1913: 254; Malloch 1915c: 359; Frick 1952a: 391 [as synonym of Agromyzaatra Meigen, 1830], 1957: 202 [lectotype designation].
Agromyza neptis Loew, 1869: 50. Frick 1952: 373. Frick 1957 [synonymy].
Dizygomyza semiposticata Hendel, 1920: 131. Frick 1957 [synonymy].
Dizygomyza (Poemyza) semiposticata . Hendel 1931–1936: 49; Nowakowski 1973: 152 [synonymy?].
Agromyza cinereifrons Frost, 1931: 276. Frick 1957 [synonymy].
Phytobia (*Poëmyza*) cinereifrons. Frick, 1952: 392.
Phytobia (*Poëmyza*) angulata. Shewell, 1953: 466; Frick 1957: 202, 1959: 380.
Phytobia (*Poëmyza*) neptis. Shewell, 1953: 468 [lectotype designation].
Cerodontha (Butomomyza) semiposticata . Nowakowski, 1967: 634.
Cerodontha (Dizygomyza) angulata . Spencer, 1969: 113.
Cerodontha (Butomomyza) angulata . Spencer & Steyskal, 1986b: 99; Scheffer et al. 2007: 771; Černý 2018: 124; Eiseman and Lonsdale 2018: 39; Černý et al. 2020: 202; Eiseman et al. 2021: 21.

###### Description

**(Fig. 99).** Wing length 2.0–2.7 mm (♂), 2.1–2.8 mm (♀). Length of ultimate section of vein M4 divided by penultimate section: 0.7–1.2. Eye height divided by gena height: 10.6–18.6. First flagellomere small, rounded, higher than long; with longer hairs on anterior margin above middle that may appear as a more or less discrete tuft. Lunule as high, or slightly higher than wide, subtriangular; minutely tomentose, velvety. Fronto-orbital plate and ocellar triangle not distinct. Posterior ocelli slightly displaced laterally.

***Chaetotaxy***: Two ori (anterior seta slightly shorter, straighter, and more angled inwards); two ors. Orbital setulae in one reclinate row. Ocellar and postocellar setae sometimes longer than fronto-orbitals. Four dorsocentrals slightly decreasing in length anteriorly. Acrostichal seta small to well-developed. Acrostichal setulae in five or six irregular rows.

***Colouration***: Setae brown to dark brown. Base colour of body pale brown, sometimes appearing yellowish, with surface subshiny; ocellar tubercle and ventral margin of gena dark brown; clypeus either dorsally or entirely dark brown; palpus, face, parafacial, back of head (sometimes excluding yellowish dorsomedial region) and abdomen darker brown; if posterolateral corner of frons to inner vertical also darker brown, then darker region sometimes extending to base of posterior fronto-orbital; postpronotum and notopleuron slightly more yellowish with posterior corner of both sometimes light yellow; lateral margin of postsutural scutum narrowly yellow; wing veins light brown, becoming light yellowish to white at base; calypter yellowish white; halter white; femora yellow apically for length equal to femur width; fore tibia yellowish; tarsi yellowish; dorsomedial region of epandrium, including process above anus, yellow to yellowish brown.

***Genitalia***: (Figs 490–502) Epandrium with small rounded process above anus. Cercus small, setulose. Surstylus small, incurved, fused to epandrium; with shallow oblique ridge of pronounced tubercle-like setae on inner surface. Subepandrial sclerite divided medially with one pair of flat plate-like dorsal sclerites with one ventral seta, and one pair of darker rod-like ventral processes with one outer subapical spine. Hypandrium subtriangular with apex broadly rounded; inner lobe with narrow, weak attachment to outer frame, with several empty sockets, weak inner plate, and long basal arm. Postgonite small, narrow, dark. Phallophorus subcylindrial, dorsally fused to basiphallus. Basiphallus composed of a single bifid sclerite; left branch flat, pale, narrow to thick plate with apex shallowly divided; right branch produced into long dark arm. Hypophallus with left sclerite L-shaped with basal section darker, thicker, and mirroring extended arm of basiphallus; right sclerite pale, small, vestigial. Paraphallus (one pair) rounded, membranous, lobate, flanking base of mesophallus, venter sclerotised. Mesophallus ca. as long as distance from base of phallophorus to base of mesophallus; stalk slightly narrower than high; apical bulb 2/5 length of segment, broadly ovate in ventral view. Distiphallus slightly longer than mesophallus, entirely divided into one pair of sinuate arms with short, shallowly curved basal section and more sharply curved apical section with relatively straight intervening space; apex of distiphallus sometimes unwidened or slightly flared at opening, but sometimes apex more broadly subconical (Fig. 501) or barrel-shaped (Nowakowski 1973: fig. 145L and Fig. 502 for specimens from NC: Indian Gap discussed by Spencer and Steyskal 1986b). Ejaculatory apodeme with short, apically widened stem grading into large, pale blade; base dark, asymmetrical; sperm pump with ventral sclerotised plate widening into one pair of large lateral rounded extensions; sperm pump clear with one pair of floating sclerotised bars at either end in Indian Gap male.

***Variation***: Many specimens from North Carolina and Tennessee (including Indian Gap male discussed and illustrated in Spencer and Steyskal 1986b) differ as follows. Acrostichal setulae in straight or irregular rows. Pigment darker overall, including on all antennal segments, which more sharply contrast paler regions; wing veins darker brown; tibiae and tarsi either darker or as described above for “typical” specimens.

A male from Massachusetts with acrostichal setulae in straight rows. Head dark brown to brown with fronto-orbital plate slightly paler; mid and hind femora entirely brown. Subepandrial sclerite with large hook on ventral process; apex of distiphallus swollen, barrel-like. Males from Washington State also similarly dark externally, but otherwise as above for “typical” specimens, with acrostichal setulae in mostly straight rows.

***Variation***: *tentatively included*: Specimens from northeastern states sometimes differ as follows. Acrostichal setulae in straight rows. Setae brown; pigment on head and thorax sometimes darker in females; first flagellomere slightly darker; clypeus entirely dark brown; notopleuron and postpronotum more evenly yellowish; legs entirely dark brown with apex of fore femur and sometimes apical margin of mid femur yellow. Spine on process of subepandrial sclerite highly reduced with surrounding area minutely textured, except in CNC male from Maryland, where spine is long, but more basally situated and angled towards base; paraphallus more produced; left lateral plate of basiphallus wider apically; distal section of distiphallus broadly rounded and C-shaped.

###### Hosts.

Cyperaceae – *Carex*, *Scirpus*. Juncaceae – *Juncus*, *Luzula*. Poaceae – *Dichanthelium* (Eiseman et al. 2021).

###### Distribution.

**Canada**: AB, ON. **USA**: widespread. Widespread in Palaearctic (Černý et al. 2020).

###### Type material.

***Lectotype* [*angulata*]: USA. PA**: “Pennsylvania, Loew coll., *angulata* m” (1♀, MCZ; type no. 13,434).

***Lectotype* [*neptis*]: USA. DC**: “DC, No. 53, Loew coll., *neptis* m.” (1♀, MCZ; type no. 13,440).

***Lectotype* [*semiposticata*]: Germany.** Pommern, Insel Rügen, 1908, Hendel (1♀, NMW).

***Holotype* [*cinereifrons*]: USA. NY**: Florida, 7.vii.1917, S.W. Frost (1♂, USNM; type no. 62,963).

###### Additional material examined.

**Canada. ON**: Ottawa, Stony Swamp, Beaver Trail, 45°18'0"N, 75°49'16"W, 13.vi.2015, O. Lonsdale, CNC441055, CNC441087 (1♂ 1♀, CNC), 7.vi.2015, CNC440794, CNC440870 (2♀, CNC), Rattlesnake Point Conservation Area, 11.vi.2011, Lonsdale and Richard, sweeping, CNC481026, CNC481027 (2♀, CNC), Wellington Co., Smith Property Trail nr. Arkell, 43°32'55"N, 80°11'0"W, 23.vi.2015, O. Lonsdale, CNC441179, CNC441188 (2♀, CNC), Kent Co., Rondeau Prov. Pk., South Point, east parking lot, 42°15'42"N, 81°50'49"W, oak savannah, Malaise, 3–15.v.2003, S.A. Marshall, debu01121095 (1♀, DEBU). **USA. CT**: Redding, 31.v.1930, A.L. Melander (1♀, USNM), **IN**: Lafayette, vii.1931, J.M. Aldrich (1♀, USNM), **MA**: Franklin Co., Northfield, 42.648774, -72.429433, 26.x.2015, em. 30.iii–3.v.2016, C.S. Eiseman, ex *Carex*, pupation external, #CSE2296, CNC654203–654211 (1♂ 8♀, CNC), em. 17.iv.2016, pupation internal, #CSE2400, CNC654195 (1♀, CNC), 276 Old Wendell Rd., 1.xi.2015, em. 3.v.2016, C.S. Eiseman, ex *Carex*, pupation external, #CSE2443, CNC654170 (1♀, CNC), 18.vii.2016, puparium 19.vii.2016, em. 4.viii.2016, C.S. Eiseman, ex *Dichantheliumclandestinum*, #CSE2842, CNC654169 (1♂, CNC), 23.vii.2016, em. 11.viii.2016, C.S. Eiseman, ex *Scirpushattorianus*, #CSE2868, CNC659970 (1♀, CNC), Hampshire Co., Northampton, 42.366360, -72.671754, 15.vi.2017, em. 6.vii.2017, C.S. Eiseman, ex *Carexstipata*, #CSE3899, CNC939710 (1♀, CNC), Pelham, Quarry St., 4.vii.2013, em. 18.vii.2013, C.S. Eiseman, ex *Dichantheliumclandestinum*, #CSE713, CNC392665 (1♂, CNC), Pelham, Butter Hill Wildlife Sanctuary, 19.iv.2015, em. 8.v.2015, C.S. Eiseman, ex *Luzula*, #CSE1543, CNC564618 (1♀, CNC), Nantucket Co., Nantucket, 41.289665, -70.010519, 30.vii.2017, em. 18.viii.2017, C.S. Eiseman, ex *Carexcrinita*, #CSE4140, CNC939651 (1♂, CNC), Worcester Co., Charlton, Flint Rd., 21.ix.2016, em. 25.ix.2016, C.S. Eiseman, ex *Dichantheliumclandestinum*, #CSE3010, CNC638884 (1♀, CNC), **MD**: Calvert Co., 8 km S Prince Frederick, 2.v.1987, J.M. Cumming, CNC481028, CNC481029 (1♂ 1♀, CNC), Bethseda, 17.v.1981, G.C. Steyskal (2♂, USNM), Montgomery Co., Colesville, 19.ix.1977, Malaise trap, W.W. Wirth (1♂, USNM), 26.vi.1977 (1♀, USNM), Cabin John, 24.v.1931, J.M. Aldrich (1♀, USNM), **NC**: Gt Smokies NP, Clingman Dome, 21.vi.1941, A.L. Melander (1♀, USNM), Smokies, Andrews Bald, 9.vii.1941, A.L. Melander (1♂ 2♀, USNM), Durham Co., Durham, Leigh Farm Park, 9.v.2017, em. 13.vi.2017, T.S. Feldman, ex *Dichanthelium*, #CSE3826, CNC939783 (1♂, CNC), 14.vi.2017, em. 3.vii.2017, T.S. Feldman, ex *Dichanthelium*, #CSE3891, CNC939773 (1♂, CNC), Pelham Road, 13.v.2017, em. 3–6.vi.2017, T.S. Feldman, ex Dichantheliumacuminatumssp.acuminatum, #CSE3784, CNC939827–939829 (1♂ 2♀, CNC), Sandy Creek Park, 16.v.2017, em. 8.vi.2017, T.S. Feldman, ex *Dichanthelium*, #CSE3806, CNC939841 (1♀, CNC), Scotland Co., Laurinburg, St. Andrews University, 10.i.2017, em. 15.ii.2017, T.S. Feldman, ex *Carex*, #CSE3150, CNC939737 (1♀, CNC), 18.i.2017, em. 6.iii.2017, T.S. Feldman, ex *Dichanthelium*, #CSE3158, CNC939751 (1♂, CNC), **NY**: Bear Mt., 31.v.1941, A.L. Melander (2♂ 3♀, USNM), **PA**: Ono, 7.vi.1940, Melander (1♂, USNM), **VA**: Hawksbill, Shenandoah, 1097–1234 m, 7.vi.1962, J.R. Vockeroth, CNC480663 (1♀, CNC), Shenandoah, Big Meadows, 3.vii.1939, Alexander (1♂, USNM), 15.vi.1941, A.L. Melander (2♂, USNM), Blacksburg, 2100', 29.v.1962, J.R. Vockeroth (1♂, USNM), Chain Bridge, 29.vii.1923, J.M. Malloch (1♂, USNM), Giles Co., Jefferson Nat. Forest, Cascades Trail, 16.v.1977, D.C. Caloren (1♂, DEBU), Giles Co., Mountain Lake Biol. Stn., 37°22'31"N, 80°31'18"W, 24.v.2001, O. Lonsdale, debu1007754 (1♂, DEBU), Alexandria Co., Maywood, 21.v.1922, W.L. McAtee (1♀, USNM), Fairfax Co., Dead Run, 28.vii.1915, R.C. Shannon (1♂, USNM), **VT**: Essex Co., 5mi W Bloomfield, 28.vi.1972, H.J. Teskey, CNC481030 (1♀, CNC), **WA**: Jefferson Co., 10.x.2012, em. 4.v.2013, C.S. Eiseman, ex broad-leaved wetland *Carex*, #CSE412, CNC358502 (1♂, CNC), 11.x.2012, em. 20.v.2013, C.S. Eiseman, ex narrow-leaved upland *Carex*, #CSE528, CNC358501 (1♂, CNC), **WV**: Parkersburg, 21.vi.1970, G. Steyskal (1♂, USNM), Parkersburg, 21.vi.1970, G. Steyskal (1♂, USNM), **State unknown**: Gt. Smokie NP, Newf’nd Ridge, 11.vii.1941, A.L. Melander (1♂ 1♀, USNM).

###### Additional material examined

[“**variation”]. USA. MA**: Hampshire Co., Pelham, Quarry St., 4.vii.2013, C.S. Eiseman, ex *Dichantheliumclandestinum*, em. 18.vii.2013, #CSE713, CNC392665 (1♂, CNC), Worcester Co., Charlton, Flint Rd., 21.ix.2016, C.S. Eiseman, *Dichantheliumclandestinum*, em. 25.ix.2016, #CSE3010, CNC638884 (1♀, CNC), Franklin Co., Northfield, 276 Old Wendell Rd., 1.xi.2015, C.S. Eiseman, *Carex*, em. 3.v.2016, #CSE2443, CNC654170 (1♀, CNC), 18.vii.2016, C.S. Eiseman, *Dichantheliumclandestinum*, em. 4.viii.2016, #CSE2842, CNC654169 (1♂, CNC), by beaver pond, 26.x.2015, C.S. Eiseman, *Carex*, em. 17.iv.2016, #CSE2400, CNC654195 (1♀, CNC), 26.x.2016, C.S. Eiseman, *Carex*, em. 30.iii-3.v.2016, #CSE2296, CNC654203–654211 (1♂ 8♀, CNC), **NC**: Gr. Smoky Mt. Nat. Pk., Clingman’s Dome, 1920–2024 m, 28.v.1957, J.R. Vockeroth, CNC480667 (1♂, CNC), Highlands, Little Bear Pen Mt., 5.viii.1957, W.R. Richards, CNC480665 (1♂, CNC), Highlands, Whitesides Cove, 853 m, 11.viii.1957, J.G. Chillcott, CNC480662 (1♀, CNC), Highlands, Wilson’s Gap, 944 m, 25.v.1957, J.R. Vockeroth, CNC480664 (1♂, CNC), Highlands, 1158 m, 12.vii.1957, J.G. Chillcott, CNC480668 (1♂, CNC), 12.viii.1957, W.R. Richards, CNC480654 (1♂, CNC), 1158 m, 29.viii.1957, J.G. Chillcott, CNC480659 (1♀, CNC), 8.vi.1957, J.R. Vockeroth, CNC480657 (1♀, CNC), Macon Co., Wayah Gap, 1249 m, 28.vii.1957, J.G. Chillcott, CNC480658 (1♀, CNC), 29.vii.1957, CNC480660, CNC480661 (2♀, CNC), Wilkes Co., Doughton Gap, 853 m, 6.vi.1962, J.G. Chillcott, CNC480655 (1♂, CNC), **TN**: East Ridge, 6.v.1952, O. Peck, CNC480656 (1♂, CNC), 9.v.1952, G.S. Walley, CNC480669 (1♂, CNC), Gr. Smoky Mt. Nat. Pk., Greenbrier Cove, 609 m, 18.v.1957, J.R. Vockeroth, CNC480666 (1♂, CNC), Indian Gap, G.S.M.N.P., 1584 m, 3.vi.1957, C.D. Hines, CNC480653 (1♂, CNC), **WA**: Jefferson Co., 10.x.2012, C.S. Eiseman, ex *Carex*, em. 20.v.2013, #CSE412, CNC358502 (1♂, CNC), 11.x.2012, ex *Carex*, em. 20.v.2013, #CSE528, CNC358501 (1♂, CNC).

###### Tentatively included specimens.

**Canada. ON**: Bruce Co., Cameron Lake Rd., 20–26.vi.1998, spring fen, RET, S.A. Marshall, debu00071175 (1♂, DEBU). **USA. CT**: Redding, 8.vi.1930, A.L. Melander (1♂, USNM), **MD**: Garrett Co., Deep Creek Lake State Park, 39°30'N, 79°23'W, 17–20.vii.2000, G.F. Hevel (2♀, USNM), Montgomery Co., Bethseda, 22.viii.1970, G. Steyskal (1♂, USNM), Montgomery Co., Dickerson, 14.vii.1974, G.A. Foster (1♂, USNM), Montgomery Co., 4mi SW of Ashton, 19.v.1985, G.F. and J.F. Hevel (1♂, USNM), 24.vii.1982 (1♂, USNM), Plummers I., H.L. Viereck (1♂, USNM), **TN**: Smokies, Chimneys, 4.vii.1941, A.L. Melander (1♂, USNM), **VA**: Giles Co., Mt. Lake, 9.ix.1976, G. Steyskal (1♂, USNM), Augusta Co., Reddish Knob, 29.viii.1953, W.W. Wirth (3♂ 1♀, USNM), Alexandria Co., Maywood, 21.v.1922, W.L. McAtee (1♀, USNM), Arlington, vi.1938, J.R. Malloch (1♀, USNM), **WV**: Morgantown, 27.vi.1970, G. Steyskal (1♂, USNM).

###### Comments.

*Cerodonthaangulata* is broadly defined here, encompassing variation that the present and previous authors (Spencer and Steyskal 1986b) recognise as likely representative of additional cryptic species than cannot at present be confidently separated due to an overlap of characters, especially those of pigment, acrostichal setulae, and the subepandrial sclerite and phallus. In all specimens, however, the mesophallus is relatively straight in profile with a shallow, wide apical bulb, the left sclerite of the hypophallus is L-shaped with a narrow apical section, and the distiphallus has the basal curve small and shallow and the apical curve more pronounced with a relatively straight intervening section. The apex of the distiphallus may be only barely widened towards the terminal opening, but some have a more pronounced apical swelling that may be more conical or barrel-shaped. The ventral process of the subepandrial sclerite also has a pronounced subapical point, but this is highly reduced in specimens with a distiphallus that is more C-shaped in profile.

The phallus of this species is unusually close to that of *Cerodonthabutomomyzina* Spencer from New Brunswick (Spencer 1969: fig. 196). Unfortunately the holotype and only known specimen of that species, originally deposited in the CNC, was lost in the mail while on loan in the 1990’s. In the original description, Spencer noted that it belonged to the lineage containing *C.angulata*, but that the prescutellar setae were weak. Prescutellar seta length in species of this subgenus appears to vary from well-developed to relatively weak, and should not in and of itself differentiate it from *C.angulata*. Other differences include calypter hairs that are yellow-ochreous, a narrowly yellow upper margin of the pleuron (likely referring to the exposed membrane), a distiphallus that is not widened apically, and a mesophallus that appears to be slightly narrower and longer. Some *C.angulata*, however, have a combination of an apically narrow distiphallus, a yellowish white calypter and reduced prescutellars, and the two species may be conspecific. Any *minor* differences in the original figure provided in Spencer (1969) may be an artifact of illustration.

##### Cerodontha (Butomomyza) subangulata

Taxon classificationAnimaliaDipteraPhytomyzinae

(Malloch)

Figs 503–507

Agromyza subangulata Malloch, 1916: 51.
Phytobia (*Poëmyza*) subangulata. Frick, 1952a: 392, 1959: 382; Spencer and Steyskal 1986b: 100.

###### Description

**(NC male).** As described for Cerodontha (Butomomyza) angulata, except as follows. Wing length 2.6 mm (♂). Female unknown. Length of ultimate section of vein M4 divided by penultimate section: 1.1. Eye height divided by gena height: 11.9. Acrostichal seta well-developed; acrostichal setulae in six straight rows. Frons between fronto-orbital plates rougher, pitted. Setae dark brown; frons darker brown, but dark brown pigment only present on tubercle; remainder of head brown to dark brown with clypeus, palpus and back of head clearly darker; brown pigment on remainder of body darker, contrasting more with paler regions; colour of postpronotum and notopleuron blotchier, with anterior and posterior regions of notopleuron yellower; wing veins brown to light brown, only yellow basal to basal cells; apices of all femora distinctly yellow, base and apex of fore tibia yellowish, and tarsi brownish yellow with apical one or two segments darker. Sclerotized portion of paraphallus flatter, band-like; distiphallus relatively straight, especially on distal 2/3, and with apex not swollen (Fig. 503); paired tubules of distiphallus very closely held along length.

*Variation*: The Illinois holotype, based on the original description, differs as follows: additional ors present, possibly up to eight rows of acrostichal setulae, eye ~ 8 × higher than gena, palpus yellowish apically, apices of mid and hind femora not yellow; additionally, the length of vein dm-m is the same as the distance from vein r-m; distiphallus slightly more curved with apex slightly swollen (Figs 504–507).

###### Host.

Unknown.

###### Distribution.

**USA**: IL. Possibly MD, NC.

###### Type material.

***Holotype*: USA. IL**: St. Joseph, 3.v.1914, Hart and Malloch (1♂, INHS). [Not examined]

###### Additional material examined.

**USA. NC**: Haywood Co., Pisgah Nat. Forest, Chestnut Bald, 5900', 2.viii.1957, J.G. Chillcott (1♂, CNC).

###### Comments.

The Maryland male from Cabin John (Spencer and Steyskal 1986) and the North Carolina male examined and illustrated here, were both identified as *Cerodontha* “near angulata” by Spencer, and tentatively assigned to *Cerodonthasubangulata*. Compared to the holotype, the tubules of the distiphallus of these males are more slender and shallowly curved. Since the length of crossvein dm-m is shorter than the length of this vein from r-m, as seen in *C.angulata*, this character is no longer considered diagnostically valuable. The collection of additional material is essential to properly establishing species boundaries and morphological variation, which will hopefully supply additional characters to use in the identification key.

##### Cerodontha (Cerodontha)

Taxon classificationAnimaliaDipteraPhytomyzinae

Rondani

Cerodontha (Cerodontha) . Nowakowski, 1962: 656, 1967: 656, 1973: 42; Spencer 1969: 142; Spencer and Steyskal 1986b: 91; Boucher 2002: 579.

The nominal subgenus is quite readily diagnosed, as the first flagellomere has a narrow, produced point on the anterodorsal corner and the lateral scutellar setae are absent. The point on the first flagellomere is sometimes reduced so that the segment has only a slight anterodorsal angle (Boucher 2002). The habitus is also quite characteristic, with species being relatively slender and elongate, and often quite heavily marked with yellow. The subgenusXenophytomyza is similar in that the lateral scutellars are also absent and the first flagellomere is angled, but this angle is slight, never produced as a point, and the body is entirely dark, never with yellow markings.

The subgenus contains ca. 60 species, with the ten Nearctic species revised by Boucher (2002). The widespread and morphologically variable species *C.dorsalis* (Loew) occurs in the Delmarva states.

#### Species description

##### Cerodontha (Cerodontha) dorsalis

Taxon classificationAnimaliaDipteraPhytomyzinae

(Loew)

Figs 106 , 508–512

Odontocera dorsalis Loew, 1863: 54.
Cerodontha dorsalis . Melander, 1913: 249; Malloch 1913: 331; Hendel 1931: 269; Curran 1934: 163; Frost 1936: 318.
Cerodontha (Cerodontha) dorsalis . Frick, 1952a: 399, 1959: 399; Spencer 1969: 143; Spencer and Steyskal 1986b: 91; Boucher 2002: 581; Scheffer et al. 2007: 771; Scheffer and Lonsdale 2018: 87; Eiseman and Lonsdale 2018: 41.
Cerodonta femoralis Meigen, 1838. Misidentification (in part). Melander 1913: 249.

###### Description

**(Fig. 106).** Wing length 2.0–2.4 mm (♂), 2.0–2.9 mm (♀). Length of ultimate section of vein M4 divided by penultimate section: 0.8–1.1. Eye height divided by gena height: 2.6–3.7. Arista relatively stout and thickened on basal 1/2, distinctly thicker than width of distal 1/2. First flagellomere slightly longer than high, longest subdorsally, where segment ends in pronounced spine; sometimes very stout apically, appearing almost subrectangular, but often more gradually tapering to apex; distal margin to distal 1/2 of ventral margin with relatively long, white hairs. Fronto-orbital plate slightly visible laterally, with anterior region more strongly projecting, continuing as narrow parafacial; cheek evident on anterior 1/2 of gena.

***Chaetotaxy***: One ori, slightly inset, usually also with additional weak ori anteriorly; two subequal ors. Orbital setulae sparse, in single row; partially reclinate, but becoming erect to proclinate anteriorly. Postocellar subequal to fronto-orbitals. Ocellar longer than fronto-orbitals. Four dorsocentral setae, slightly decreasing in length anteriorly. Anterior notopleural seta, lateral scutellar seta, acrostichal seta and acrostichal setulae absent.

***Colouration***: Setae black. Base colour of head light yellow to slightly whitish, contrasting rest of body; yellower between fronto-orbital plates on frons, on face, palpus, scape, pedicel and gena; first flagellomere dark brown; fronto-orbital plate with narrow dark brown marking posterolaterally to base of anterior or posterior ors; posterolateral corner of frons dark brown to dorsal margin of eye and outer vertical seta; clypeus yellow to brown with lateral arms usually browner; back of head dark brown. Notum mostly dark brown with sparse grey pruinosity, but often with yellowish to distinctly yellow pattern, usually including a vestige of the following: most or all of posterior margin, one pair of postsutural intra-alar stripes and one stripe between dorsocentral rows to approximate level of the second dorsocentral seta that continue to transverse suture along dorsocentral rows as one pair of very narrow lines; at least part of notopleuron and scutellum (usually wide central stripe) yellow, and postpronotum yellow with dark anteromedial spot that may predominate; metanotum usually yellow lateral scutellum. Pleuron dark brown with at least part of anepisternum yellow. Legs yellow with tibiae and tarsi light brown to brown. Halter white. Calypter margin greyish, hairs brown. Abdomen dark brown.

***Variation***: Darker forms differ as follows: dark posterolateral spot on frons sometimes reaching base of inner vertical seta; scutum, scutellum and anepisternum entirely dark, sometimes excluding yellowish medial marking on scutellum; femora sometimes with dark striping or mottling, at least basally. Paler forms with distinct yellow markings on notum; scutellum widely yellow medially with lateral margins dark; anepisternum usually entirely yellow with venter sometimes darker, katepisternum entirely yellow or with variable dark brown ventral spot.

***Genitalia***: (Figs 508–512) Epandrium rounded, without process protruding above anus. Cercus small, setose. Surstylus setose, produced inwards anterodorsally. Subepandrial sclerite with one pair of strong setae and one pair of stout ventrolateral processes; rounded apically and with shallow outer subapical point. Hypandrium thin, halves approximate, with one pair of sockets on inner lobe. Postgonite small, bare. Phallophorus not much longer than wide, narrower to base, left distolateral margin more produced. Basiphallus with one broad left lateral sclerite slightly extending onto dorsal and left ventolateral surfaces. Hypophallus asymmetrical, with left sclerite darker, well-defined and with regular margin; right sclerite much larger but irregular, ill-defined, with minute sclerotised patches and hairs distally, and with base narrow, darker and discrete; clear tubule emerging from between sclerites. Mesophallus with dark, swollen irregular base enclosing apex of duct; otherwise cylindrical with basal 1/2 swollen, narrowest point at 2/3 length, and distoventral margin produced basally as thick lip. Distiphallus entirely divided into one pair of long tubules equal to length of phallophorus + basiphallus; somewhat S-shaped with basal curve short and apex directed anterodorsally; distal 1/3 thicker, darker, barrel-shaped, with narrow seam ventromedially. Ejaculatory apodeme with short, stout base and stem, blade relatively small with clear margin; sperm pump with one pair of sclerotisations continuing onto base of duct.

###### Hosts.

Poaceae – *Agrostis*, *Avena*, *Bromus*, *Dactylis*, *Dichanthelium*, *Digitaria*, *Echinochloa*, *Ehrharta*, *Eleusine*, *Elymus*, *Eragrostis*, *Hordeum*, *Lolium*, *Panicum*, *Phalaris*, *Phleum*, *Poa*, *Secale*, *Setaria*, *Sorghum*, ×*Triticosecale*, *Triticum*, *Zea* (Frick 1959; Spencer and Steyskal 1986b; Eiseman and Lonsdale 2018).

###### Distribution.

**Canada**: AB, BC, MB, NB, NS, ON, QC, SK. **USA**: “probably present in all states” (Spencer and Steyskal 1986b). Brazil. Colombia. Ecuador. Guatemala. Jamaica. Mexico. Peru. Puerto Rico. Eastern Russia. Mongolia.

###### Type material.

***Holotype*: USA. DC**: “51, DC, Loew Coll, dorsalis” (1♀, MCZ; type no. 13,433). [Not examined]

###### Additional material examined.

**Canada. AB**: Waterton Lakes National Park, 49°5'N, 113°53'W, 1347 m, meadow, 5.vi.2005, Goulet and Boudreault, CNC480672, CNC480676 (1♂ 1♀, CNC), **BC**: Agassiz, Res. Sta., rhodo patch, 49°15'N, 121°46'W, 23.vii.2004, S.A. Marshall, Malaise trap, CNC287186, CNC287220, CNC287251, CNC287283 (2♂ 2♀, CNC), Mt. Kobau, 49°5'N, 119°38'W, fallow field, 23.v.2005, Goulet and Boudreault, sweeping, CNC480673–480685 (11♂ 1♀, CNC), Osoyoos, 49°2'N, 119°27'W, alfalfa, 26.v.2005, Goulet and Boudreault, site 8, CNC480687 (1♀, CNC), Apex Mt. Ski Area, 2.viii.2004, 49°28'56"N, 119°56'04"W, J.E. Swann (1♂, CNC), **MB**: Aweme, Criddle homestead, 49°43'N, 99°35'W, mixed grass prairie, 24.vii.2007, H. Goulet, sweeping, CNC480686 (1♂, CNC), Assiniboine River Conservation Area, 49°40'42.48"N, 99°36'46.56"W, 1.vi.2007, Goulet, Boudreault and Fernandez, CNC352493 (1♀, CNC), **NB**: Madawaska Co., Saint-Jacques, Baisley Rd. at QC border, 27.vii.2013, O. Lonsdale, CNC271171, CNC271188 (2♂, CNC), **ON**: Moose Factory, 51°16'N, 80°36'W, 11.vi.1949, D.P. Whillans, CNC_Diptera109362 (1 ex, CNC), Ottawa, 3.vi.2007, J.R. Vockeroth, CNCDiptera164460 (1♂, CNC), Algonquin Provincial Park, Whitefish Lake Group Campground, 45°33'47.01"N, 78°26'13"W, 414 m, 28.vi-2.vii.2008, J.andA. Skevington, Malaise trap, CNC339235, CNC339249 (1♂ 1♀, CNC), Almonte, Burnt Lands Provincial Park, 45°15'43"N, 76°9'8"W, 5.vii.2015, O. Lonsdale, sweeping, CNC454712 (1♂, CNC), Sudbury Dist., Killarney Provincial Park, Cranberry Bog Trail, 46°1'8"N, 81°23'42"W, 1.ix.2014, O. Lonsdale, CNC380415 (1♀, CNC), Sudbury Dist., Killarney Provincial Park, Lake of the Woods Trail, 46°6'36"N, 81°11'48"W, 2.ix.2014, O. Lonsdale, CNC380790, CNC380793 (2♂, CNC), **SK**: Maple Creek, mowed grass, 15.vii.2005, J.R. Vockeroth, CNCDiptera166564 (1♂, CNC), 7 km E Leask, 10.viii.2005, J.R. Vockeroth, CNCDiptera193131 (1♀, CNC), Cypress Hills Provincial Park, E Block, 9.vii.2005, J.R. Vockeroth, CNCDiptera166367 (1♂, CNC). **USA. CO**: Chaffee Co., Poncha Springs, South Arkansas River, 8.vii.2015, em. by 21.vii.2015, C.S. Eiseman, ex ×*Triticosecale*, #CSE1777, CNC634812 (1♀, CNC), Boulder Co., Corona Pass, 3230 m, marshy meadow at timberline, 6.vii.1961, J.G. Chillcott, CNC480693 (1♂, CNC), Doolittle Ranch, Mt. Evans, 2987 m, 12.vii.1961, C.H. Mann, CNC480720 (1♂, CNC), 27.vii.1961, J.G. Chillcott, CNC480717–480719, CNC480722 (4♂, CNC), 3.viii.1961, CNC480725 (1♀, CNC), 8.vii.1961, C.H. Mann, CNC480721, CNC480723, CNC480724 (1♂ 2♀, CNC), Echo L., Mt. Evans, 3230 m, 11.viii.1961, C.H. Mann, CNC480696 (1♀, CNC), 12.viii.1961, CNC480694 (1♂, CNC), 24.viii.1961, CNC480695 (1♂, CNC), Idaho Springs, 5mi SW, 2621 m, 27.vii.1961, C.H. Mann, CNC480713 (1♂, CNC), Lake Co., Independence Pass, 3688 m, tundra, 31.vii.1961, J.G. Chillcott, CNC480714 (1♂, CNC), 8.viii.1961, B.H. Poole, CNC480715, CNC480716 (2♂, CNC), Mt. Evans, 3566 m, timberline, 22.vii.1961, W.R.M. Mason, CNC480698 (1♀, CNC), 3962 m, on tundra, 28.vii.1961, C.H. Mann, CNC480699 (1♀, CNC), 4267 m, 4.viii.1961, B.H. Poole, CNC480697 (1♀, CNC), Nederland, 3mi N, 2590 m, marshy stream margin, 2.vii.1961, J.G. Chillcott, CNC480711 (1♀, CNC), dry gravelly meadow, CNC480710 (1♂, CNC), Nederland, Science Lodge, 2895 m, 27.vi.1961, B.H. Poole, CNC480712 (1♀, CNC), Nederland, 2529 m, seepage area, 5.vii.1961, J.G. Chillcott, CNC480700–480709 (7♂ 3♀, CNC), Niwot Ridge, nr. Ward, 3444 m, 28.vi.1961, C.H. Mann, CNC480690, CNC480692 (2♂, CNC), Vernon Cn., nr. Golden, 2194 m, 31.vii.1961, C.H. Mann, CNC480688, CNC480689, CNC480691 (2♂ 1♀, CNC), 2003, CNC484171 (4 ex, CNC), **CT**: Litchfield Co., Canaan, Falls Village, 21.vii.2015, em. 27.vii.2015, C. Vispo, ex *Elymus*, #CSE2170, CNC564678 (1♂, CNC), **NC**: Clingmans Dome, 35°35'45"N, 83°29'54"W, 6500', 28.v.1999, J.R. Vockeroth, CNCDiptera193694 (1♀, CNC), Durham Co., Durham, Pelham Rd., 29.iv.2017, em. ~17–22.v.2017, T.S. Feldman, ex *Dichanthelium*, #CSE3675, CNC939801–939802 (2♀, CNC), 29.iv.2017, em. ~17.v.2017, T.S. Feldman, ex *Elymusvirginicus*, #CSE3676, CNC939821 (1♀, CNC), 13.v.2017, em. 19.v.2017, T.S. Feldman, ex Dichantheliumdichotomumssp.nitidum, #CSE3693, CNC939803 (1♀, CNC), Scotland Co., Laurinburg, St. Andrews University, 18.iv.2017, em. 18.v.2017, T.S. Feldman, ex *Agrostishyemalis*, #CSE3685, CNC939750 (1♂, CNC).

###### Comments.

*Cerodonthadorsalis* is widespread in North America, and the only member of the subgenus found east of Manitoba and Colorado. The subgenus is more diverse in the western Nearctic, where differentiating *C.dorsalis* from congeners can be difficult, requiring measurements of body size and examining the relative width of the arista, frons and parafacial. Dissection of the male is the most reliable method of diagnosis, with the large, thickened apical section of the distiphallus being unique.

##### Cerodontha (Dizygomyza)

Taxon classificationAnimaliaDipteraPhytomyzinae

Hendel

Dizygomyza Hendel, 1920: 130. Type species: AgromyzamorosaMeigen 1830, by original designation. Hendel 1931: 83.
Phytobia (Dizygomyza) . Frick 1952a: 383, 1959: 383.
Cerodontha (Dizygomyza) . Nowakowski 1962: 102, 1967: 638; Spencer 1969: 113; Spencer and Steyskal 1986b: 100.

Dizygomyzais a predominantly north temperatesubgenuscharacterised most obviously by a large, broad semi-circular lunule extending laterally to the fronto-orbital plate, the bases of the antennae are clearly separated, and the male first flagellomere is usually considerably enlarged and clothed in long, dense whitish hairs. Species are also often predominantly greyish tomentose across the body, including the lunule, with variable yellow or yellowish markings. Differences in external and genitalic morphology between species can be very slight, making global or even Nearctic studies difficult, but the four Delmarva species can be readily identified.

### ﻿Key to the Delmarva Cerodontha (Dizygomyza)

**Table:** 

1	Notopleuron and postpronotum light yellow to whitish. Apices of femora all distinctly light yellow. Right sclerite of hypophallus, if present, small and dark. Distiphallus as long as mesophallus, very shallowly curved (Figs 527, 528)	***C.magnicornis* (Loew)**
–	Notopleuron and postpronotum brown, sometimes with yellowish tint and/or small yellow spots in posterolateral corners, but never broadly and distinctly pale as above. Apices of mid and hind femora usually much darker than fore femur (some *C.morosa* with apices of all femora equally pale). Right sclerite of hypophallus faint to absent. Distiphallus longer, more strongly curved	2
2	Abdomen dark brown with tergites 1–4(5) light yellow excluding faded medial spot. Mesophallus with stem strongly swollen medially. Mesophallus stout, stem strongly swollen medially. Paraphallus well-sclerotised, pointed ventrally. Distiphallus not much longer than mesophallus; basal curve nearly as long as distal; tubules diverging on distal 1/2 (Figs 523, 524)	***C.maclayi* Spencer**
–	Abdomen entirely dark brown. Paraphallus clear, lobate, at most with faint patch of pigment. Mesophallus longer and thinner than above. Distiphallus longer than above, basal curve much shorter than distal curve; tubules parallel along length	3
3	Fronto-orbital plate dirty yellowish brown, contrasting rest of body. Apices of mid and hind femora darker than apex of fore femur. Surstylus with three stout tubercle-like setae. Distal curve of distiphallus ca. as long as mesophallus, semi-circular, slightly swollen apically (Figs 517, 518). Left sclerite of basiphallus small, clavate	***C.fasciata* (Strobl)**
–	Fronto-orbital plate brown to dark brown, not paler than centre of frons (Fig. 105). Apices of femora either dark, as above, or all equally pale. Surstylus with two rows of slender tubercle-like setae. Distal curve of distiphallus very large, slightly straightened medially, not swollen apically (Figs 533, 534). Left sclerite of basiphallus large, pointed	***C.morosa* (Meigen)**

#### Species descriptions

##### Cerodontha (Dizygomyza) fasciata

Taxon classificationAnimaliaDipteraPhytomyzinae

(Strobl)

Figs 513–518

Phyllomyza fasciata Strobl, 1880: 38.
Agromyza grossicornis var. fasciata . Strobl, 1893: 135.
Dizygomyza morosa Meigen. Misidentification. Hendel 1931–1936: 90.
Dizygomyza plumbea Hendel, 1931–1936: 92. Spencer 1971 [synonymy].
Phytobia (Dizygomyza) plumbea . Groschke, 1957: 116.
Cerodontha (Dizygomyza) plumbea . (in part) Nowakowski, 1967: 645.
Dizygomyza grisea Ryden, 1952: 26. Nowakowski 1967: 645 [as synonym of plumbea]; Spencer 1971 [as synonym of fasciata]
Cerodontha (Dizygomyza) fasciata . Nowakowski, 1967: 644, 1972: 761; Spencer 1971: 153 (lectotype designation), 1976: 220; Spencer and Steyskal 1986b: 282; Benavent-Corai et al. 2005: 11.
Cerodontha (Dizygomyza) chaixiana (Groschke). Misidentification. Spencer, 1969: 115.

###### Description.

Wing length 2.3–2.6 mm (♂), 2.7–2.8 mm (♀). Length of ultimate section of vein M4 divided by penultimate section: 1.3–1.7. Eye height divided by gena height: 6.0–10.8. Male first flagellomere much enlarged, circular or slightly higher than long with anterodorsal margin sometimes slightly angled; covered with long, whitish hairs that end before base; female first flagellomere not enlarged or haired as for male, but with anterodorsal margin sometimes slightly angled. Arista slightly thickened on basal ¼-1/3. Male orbital not strongly projecting, but evident along length when viewed laterally with anterior margin most prominent; female fronto-orbital plate weakly visible laterally. Lunule large, broadly semi-circular with lateral margin meeting fronto-orbital plate; slightly narrower in female. Fronto-orbital plate widest medially, not exceeding 1/5 frons width. Posterior ocelli slightly displaced.

***Chaetotaxy***: Two ori (anterior seta slightly shorter); two ors; sometimes with additional ors or minute anterior ori on one side. Orbital setulae in one sparse row; erect with anterior setulae slightly proclinate. Postocellar and ocellar setae subequal to fronto-orbitals. Apex of palpus sometimes with two slightly stronger setae. Four dorsocentrals, decreasing in length anteriorly, anterior dorsocentral almost 1/2 length of posterior dorsocentral. Six irregular rows of acrostichal setulae, reduced to two rows posteriorly, nearly attaining posterior margin of scutum; slightly longer posteriorly with one pair sometimes appearing as prescutellar acrostichal setae.

***Colouration***: Setae dark brown, paler when reflecting light. Antenna brown to dark brown with first flagellomere darker; frons dirty yellowish brown with greyish pruinosity, palest on fronto-orbital plate, region between triangle and fronto-orbital plate darker, speckled with minute brown pits; lateral margin of fronto-orbital plate darker brown and base of setae sometimes with minute brown spot; ocellar triangle dark brown, slightly larger than tubercle, confluent with dark brown margin along back of head; slightly paler triangular region surrounding ocellar triangle; dark brown spot in posterolateral corner of frons reaching base of inner vertical seta; lunule smooth, velvety greyish, sometimes slightly darker brown dorsally; clypeus and venter of gena dark brown, remainder of gena dorsally with colour and texture as seen medially on frons; face dark brown with paler regions medially and ventrally. Thorax dark brown with faint pruinosity that is thicker on notum and dorsally on pleuron; notopleuron yellowish, at least in posterolateral corner, where yellow may be more pronounced; scutum with minute light yellow spot at lateral corner of scutellum and sometimes anterior corner of postpronotum. Calypter margin and hairs yellowish white. Wing veins yellow basal to medial 1/2 of basal cells. Halter yellow. Legs dark brown; apex of fore femur light yellow for length equal to width of femur; similar faint yellowish pigment often evident on mid and hind femora with hind leg darker; tarsi paler. Abdomen dark brown.

***Genitalia***: (Figs 513–518) Epandrium with pronounced process above anus that has base constricted (absent in male from Ohio); surstylus fused to epandrium, small, directed inwards, with three stout tubercle-like setae. Subepandrial sclerite with weak transverse dorsal band and one pair of medial setae; ventral lobe dark, apically tapering and medially curved; outer margin of process minutely serrated subapically and with shallow apical point. Phallophorus with narrow process on left margin, dorsally confluent with basiphallus. Basiphallus with dorsal plate that extends along right side as downturned, pointed process mirroring single sclerite of hypophallus, which is L-shaped and narrow and paler apically; left sclerite of basiphallus dark, clavate and weakly attached to dorsobasal section. Paraphallus (one pair) clear, lobate, with weak comma-shaped sclerotisation. Mesophallus very dark, rod-shaped, narrowest basally; with complete ventral suture; distal 2/5 slightly swollen to enclose chamber, with dorsum, ventral surface and lateroventral plate better-sclerotised. Distiphallus S-shaped, divided into one pair of separate parallel tubules; darker to base with clear basal section meeting mesophallus; with small basal curve and larger apical curve both semi-circular in outline; apex slightly swollen for length equal to 2 × width. Ejaculatory apodeme with basally tapering stem with broad base; blade large, clear; sperm pump with basal mottling.

###### Host.

Poaceae – *Poa*.

###### Distribution.

**Canada**: AB, BC*, NS*, ON. **USA**: MA*, MD*, MI*, NC, NY*, OH*, VA*, WV*. Europe (Papp and Černý 2016).

###### Type material.

***Lectotype* [*fasciata*]: Austria**: Karnten: Ossiach (1♂, Coll. Strobl, Admont). [Not examined]

***Lectotype* [*plumbea*]: Austria** (1♂, NMW). [Not examined]

***Holotype* [*grisea*]: Sewden**: Gotland: Fridhem, 22.vi (1♂, ZIL). [Not examined]

###### Material examined.

**Canada. AB**: Jumping Pd. Cr., 20 mi W Calgary, 28.vi.1962, K.C. Hermann, CNC480756 (1♂, CNC), **BC**: Atlin, 6.vii.1955, B.A. Gibbard, CNC480770 (1♀, CNC), Royston, 7.vi.1955, R. Coyles, CNC480760 (1♂, CNC), Terrace, marshy meadow, 11.vi.1960, J.G. Chillcott, CNC480740–480742 (3♂, CNC), 31.v.1960, C.H. Mann, CNC480743–480747, CNC480771–480778 (5♂ 8♀, CNC), R.J. Pilfrey, CNC480779 (1♀, CNC), 31.vi.1960, J.G. Chillcott, CNC480748–480754, CNC480780–480785 (7♂ 6♀, CNC), Zymagotitz River, 6mi W Terrace, 57 m, 20.iii.1960, R. Pilfrey, CNC480739 (1♂, CNC), **NS**: Kentville, 6.viii.1958, J.R. Vockeroth, CNC480757, CNC480768, CNC480769 (1♂ 2♀, CNC), **ON**: Dresden, 2.vii.1962, S.M. Clark, CNC480767 (1♀, CNC), Midland, swamp woods, balsam poplar, 2.v.1959, J.G. Chillcott, CNC480765 (1♀, CNC), North Gower, 14.vii.1985, D. Bell, light trap, CNC480758 (1♂, CNC), Ottawa, 14.v.1957, J.G. Chillcott, CNC480766 (1♀, CNC), swept from Sagittaria, 3.ix.1989, J.R. Vockeroth, CNC480759 (1♂, CNC), St. Lawrence Is. Nat. Par., McDonald Is., 14.vii.1976, A. Carter, Code 4092-J, CNC480761 (1♂, CNC), St. Lawrence Is. Nat. Park, Thwartway Is., 17.vii.1976, A. Carter, Code 4133-Y, CNC480762 (1♂, CNC), St. Lawrence Is., Thwartway Is., 4.viii.1976, W. Reid, Code 4322-H, CNC480764 (1♀, CNC), Bells Corners, 5.vii.1973, F. Crombie and P. Nash, CNC480763 (1♂, CNC). **USA. MA**: Concord, 17.vii.1961, W.W. Wirth (1♂, USNM), Woods Hole, vii.1918, A.H. Sturtevant (1♂, USNM), **MD**: Lavale, 9.v.1970, G. Steyskal (1♂, USNM), Montgomery Co., Clarksburg, Little Bennett Reg. Park, 21.ix.1990, W.E. Steiner and M.J. and R. Molineaux (1♂, USNM), Montgomery Co., Bethseda, 4.v.1969, G. Steyskal (1♂, USNM), Montgomery Co., 4mi SW of Ashton, 25.iv.1987, G.F. and J.F. Hevel (1♂, USNM), 28.iv.1985 (1♂, USNM), **MI**: Isle Royale, 3–7.viii.1936, C. Sabrosky (1♂, USNM), **NC**: Mitchell Co., Roan Mtn., 1889 m, 13.viii.1957, J.G. Chillcott, CNC480755 (1♂, CNC), **NY**: Geneva, 28.v.1914, A.L. Melander (1♂, USNM), **OH**: Columbiana Co., Beaver Creek, 40°43.8'N, 80°36.5"W, 7.vii.1976, B.A. Steinly (1♂, USNM), Stark Co., Berlin Reservoir, 40°58.9'N, 81°06.0'W, 7.vii.1976, B.A. Steinly, sand shore, 200 net sweeps (1♂, USNM), **VA**: Big Meadows, 3.vii.1939, A.L. Melander (1♂, USNM), **WV**: Greenbrier Co., Charmco, 6.ix.1982, G.F. and J.F. Hevel (1♂, USNM), Morgan Co., near Great Cacapon, 3.vii.1983, G.F. and J.F. Hevel (1♂, USNM), White Sulfur Springs, 16.vi.1970, G. Steyskal (1♂, USNM).

##### Cerodontha (Dizygomyza) maclayi

Taxon classificationAnimaliaDipteraPhytomyzinae

Spencer

Figs 519–524

Cerodontha (Dizygomyza) maclayi Spencer, 1981: 196. Spencer and Steyskal 1986b: 105.

###### Description.

Wing length 2.4–3.0 mm (♂), 2.8–3.2 mm (♀). Length of ultimate section of vein M4 divided by penultimate section: 1.2–1.3. Eye height divided by gena height: 4.9–8.5. Male first flagellomere much enlarged, broadly kidney-shaped to subcircular, and covered with long, whitish hairs that end before base; female first flagellomere ovate, not enlarged as in male, but with slightly longer hairs along anterior margin. Arista slightly thickened on basal 1/4. Eye prominent anteriorly above midpoint. Fronto-orbital plate not projecting except slightly in front of prominent anterior margin of eye; fronto-orbital plate widest medially, not exceeding ¼ frons width. Lunule large, broadly semi-circular with lateral margin meeting fronto-orbital plate; slightly narrower in female. Posterior ocelli slightly displaced.

***Chaetotaxy***: Two ori (anterior seta slightly shorter, entirely absent in Glen Echo male); two ors. Orbital setulae erect to reclinate, in one sparse row. Postocellar and ocellar setae subequal to fronto-orbitals. Two or three (rarely four) stronger apical setae on palpus. Four dorsocentral setae, slightly decreasing in length anteriorly, with anterior seta as small as 2/3 length of posterior seta. Four to five scattered rows of acrostichal setulae not reaching level of posterior dorsocentral; posterior pair of setulae larger, almost seta-like.

***Colouration***: Setae dark brown. Body dark brown with faint pruinosity that is moderately dense on notum and dorsally on pleuron. Frons slightly paler, inner margin of fronto-orbital plate narrowly yellowish; ocellar triangle (slightly larger than tubercle), face, clypeus, palpus and venter of gena darker; pedicel and remainder of gena slightly yellowish; centre of frons and gena with minute brown pits; lunule velvety and slightly iridescent. Notopleuron and postpronotum slightly paler brown with yellowish mottling. Calypter margin and hairs yellowish white. Wing veins yellowish basally. Halter yellow. Apex of fore femur light yellow for length equal to femur width; apices of mid and hind femora narrowly and faintly yellowish, darker on hind leg; base of fore tibia narrowly light yellow. Abdominal segments 1–5 light yellow, with faint, elongate medial spot on tergites 2–5, which becomes larger on successive tergites; tergite 1 sometimes with brownish dorsal infuscation; sometimes tergite 5 brownish to brown with centre darker, and specimens from USA with tergite 5 dark brown.

***Genitalia***: (Figs 519–524) Epandrium with pronounced, rounded process above anus. Surstylus fused to epandrium, directed inwards, with irregular row of long tubercle-like setae. Subepandrial sclerite with transverse dorsal band nearly divided medially with one pair of setae; ventral lobe dark, elongate, slightly curved and with minute outer-apical point. Phallophorus with thin left lateral extension, dorsally fused to mesophallus. Mesophallus extends along dorsal to right lateral surface where it produces narrow dark process distally that mirrors strong left sclerite of hypophallus; with irregular dorsomedial sclerite and thin, apically clavate left lateral sclerite. Left lateral sclerite of hypophallus dark, mostly straight with slight medial bend; with small, very faint to absent rod-like right lateral sclerite. Paraphallus directed ventrally, relatively dark and curved, appearing pointed when viewed laterally. Mesophallus dark, with complete ventral suture (largely indistinct), relatively short and stout with apical swelling 2/5 length of segment; stem strongly swollen medially, tapered at base and apex; apical bulb most heavily sclerotised along dorsum, ventrolaterally and along ventral suture. Distiphallus divided into one pair of tubules that are S-shaped in profile, as long as distance from apex of mesophallus to apex of phallophorus, and diverging on distal 1/2; basal curve nearly as long as distal curve, but shallower and with irregularly sclerotised base; distal curve semi-circular with apical segment that is paler and as long as wide. Ejaculatory apodeme with wide stem that is nearly symmetrical; blade paler to margin; sperm pump pale but base of duct lightly pigmented.

###### Host.

Unknown.

###### Distribution.

**Canada.** NB*, ON*. **USA**: CA, MA*, MD*, NC*, NY*, TN*.

###### Type material.

***Holotype*: USA. CA**: Mono Co., Leavitt Meadow, 11.vii.1961, A.T. McClay (1♂, UCD). [Not examined]

###### Material examined.

**Canada. NB**: Kouchibouguac N.P., 12.vii.1977, J.F. McAlpine, Code – 6041I, CNC480799 (1♀, CNC), 6.vii.1977, Code – 6039G, CNC480793–480798 (4♂ 2♀, CNC), 9.vii.1977, Code – 6023Q, CNC480788–480792 (5♂, CNC), 13.vii.1977, Code – 6042J, CNC480800 (1♀, CNC), 26.vi.1977, J.R. Vockeroth, CNC480787 (1♂, CNC), 30.vi.1977, Code – 5456V, CNC480801 (1♀, CNC), **ON**: Iroquois Falls, nearly bare damp sand, 30.vi.1987, J.R. Vockeroth, sweeping, CNC480786 (1♂, CNC). **USA. MA**: Greenfield, 1.vi.1914, A.L. Melander (1♂, USNM), Boston, May, A.L. Melander (1♂, USNM), **MD**: Glen Echo, 26.v.1923, J.R. Malloch (1♂, USNM), **NC**: Gt Smokies N.P., Clingman’s Dome, 21.vi.1941, A.L. Melander (1♂, USNM), **NY**: Bear Mt., 31.v.1937, A.L. Melander (2♂, USNM), Smokies, Andrews Bald, 9.vii.1941, A.L. Melander (3♂, USNM), **TN**: Smokies, Chimneys, 21.vi.1941, A.L. Melander (1♂, USNM), Gt Smokies N.P., Newfnd Ridge, 11.vii.1941, A.L. Melander (1♂, USNM). **Locality unknown.** [illegible], 5.vii.1913, CNC480802 (1♂, CNC).

###### Comments.

The pale abdominal segments readily diagnose Cerodontha (Dizygomyza) maclayi in the Delmarva states, but similar species exist elsewhere that should be considered in broader studies, including potentially new species in western Canada and nearby states in the eastern USA. The records provided here greatly expand the known distribution of this species in the eastern USA and Canada.

##### Cerodontha (Dizygomyza) magnicornis

Taxon classificationAnimaliaDipteraPhytomyzinae

(Loew)

Figs 525–528

Agromyza magnicornis Loew, 1869: 46.
Phytobia (Dizygomyza) magnicornis . Frick 1952: 396, 1959: 384.
Cerodontha (Dizygomyza) magnicornis . Nowakowski 1973: 222; Spencer 1969: 121 [as synonym of morosa]; Spencer and Steyskal 1986b: 283; Eiseman and Lonsdale 2019: 10.

###### Description.

Wing length 2.0–2.4 mm (♂), 2.3–2.6 mm (♀). Length of ultimate section of vein M4 divided by penultimate section: 1.1–1.6. Eye height divided by gena height: 4.5–6.2. Male first flagellomere much enlarged, circular or slightly higher than long, and covered with long, whitish hairs that end before base; female first flagellomere not enlarged as for male, but with anterodorsal margin sometimes slightly angled and with slightly longer whitish hairs anteromedially. Arista slightly thickened on basal 1/4–1/3. Eye large and rounded, projecting anteromedially. Fronto-orbital plate weakly visible laterally, widest medially, not more than 1/5 width of frons. Lunule large, broadly semi-circular with lateral margin meeting fronto-orbital plate; slightly narrower in female. Posterior ocelli slightly displaced.

***Chaetotaxy***: Two ori (anterior seta shorter, but rarely absent); two ors. Orbital setulae erect to reclinate, in one sparse row. Postocellar and ocellar setae subequal to fronto-orbitals. Four dorsocentral setae, slightly decreasing in length anteriorly with anterior seta as short as 2/3 length of posterior seta. Acrostichal seta present. Five irregular rows of acrostichal setulae. Apex of palpus with one or two slightly longer setae.

***Colouration***: Setae dark brown. Head brown with face and first flagellomere darker; ocellar triangle (slightly larger than tubercle), back of head, venter of gena and clypeus dark brown; male fronto-orbital plate and pedicel dark brown, female sometimes with pigment faded anteriorly on fronto-orbital plate and pedicel; lunule paler, dorsum beige. Gena and frons between triangle and fronto-orbital plates with minute brown pits. Thorax dark brown with greyish pruinosity strongest on notum and dorsally on pleuron; postpronotum and notopleuron light yellow to whitish yellow excluding dark narrow lateral spot on notopleuron and small to large anteromedial spot on postpronotum; supra-alar margin of scutum sometimes yellowish. Calypter margin and hairs yellowish white. Wing veins light brown to brown, becoming whitish on basal 1/3–1/2. Halter yellow. Legs dark brown with apices of femora distinctly light yellow for length equal to width of femur. Abdomen dark brown with pregenital tergites narrowly and faintly yellow laterally.

***Genitalia***: (Figs 525–528) Epandrium with very shallow dorsal process above anus. Surstylus fused to epandrium, turned inwards and expanding dorsally, with row of six to seven tubercle-like setae. Subepandrial sclerite with transverse dorsal rod that is medially broken and with one pair of setae; ventral lobe long, curved, narrow and with slight outer-distal point. Phallophorus produced along left lateral margin, dorsally fused to relatively broad dorsomedial section of basiphallus that extends along right lateral surface as flat downturned process mirroring left sclerite of hypophallus; left lateral process plate-like, slightly longer than wide, abutting dorsal sclerite on basal 1/2, dark along concave apical margin; basiphallus relatively short and compact. Hypophallus dominated by left lateral sclerite that is dark and rod-like medially with paler, broad, curved lateral extension; sometimes with small, dark right lateral sclerite. Paraphallus weak, lobate, with small, faint irregular sclerotised patch ventrally. Mesophallus dark, narrow, rod-like, with distal 1/3 abruptly swollen and with dorsal, ventrolateral, and ventromedial surfaces darker. Distiphallus entirely divided into two tubules, relatively short, as long as mesophallus; S-shaped, darker to base, with middle either perpendicular to long axis of phallus or slightly more angled (as in figure); tapering apically with minutely textured and sclerotised dorsolateral membrane; curves shallow, basal curve slightly shorter. Ejaculatory apodeme not observed.

###### Host.

Cyperaceae – *Carex*.

###### Distribution.

**Canada**: BC [unconfirmed record from Frick (1959)], MB*, NB*, NS*, ON*, QC*. **USA**: CO, CT*, DC, DE*, IA, IL, IN, MI, NC*, NH, NY*, OH, OK, PA, TN, VA*.

###### Type material.

***Holotype*: USA. PA**: “Penn.“ (1♂, MCZ). [Not examined]

###### Material examined.

**Canada. MB**: Aweme, 18.vii.1916, N. Criddle, Teste Aldrich, CNC480810 (1♂, CNC), 28.viii.1917, CNC480803, CNC480806 (1♂ 1♀, CNC), 30.viii.1917, CNC480815 (1♀, CNC), Ninette, Oak-Aspen community, 11.vi.1958, J.F. McAlpine, CNC480827 (1♂, CNC), Maple-Elm floodplain community, 12.vi.1958, J.F. McAlpine, CNC480828 (1♀, CNC), ex *Betulaglandulosa*, 15.vii.1958, CNC480826 (1♂, CNC), **NB**: Chamcook, 9.viii.1957, G.E. Shewell, CNC480824, CNC480825 (1♂ 1♀, CNC), **NS**: Smith’s Cove, 6.viii.1925, A. Gibson, CNC480804 (1♂, CNC), **ON**: Bell’s Cor., 16.vi.1954, D. Cobb, CNC480820 (1♂, CNC), Britannia, 17.vi.1938, G.E. Shewell, CNC480805 (1♂, CNC), Grand Bend, 15.vii.1939, G.E. Shewell, CNC480813 (1♀, CNC), Iroquois Falls, overgrown wet shrubby bog, 18.vi.1987, J.R. Vockeroth, CNC480819 (1♂, CNC), Marmora, 25.viii.1952, C. Boyle, CNC480823 (1♂, CNC), Midland, 20.v.1959, J.G. Chillcott, CNC480821 (1♂, CNC), Ottawa, 3.vi.1958, J.R. Vockeroth, CNC480822 (1♂, CNC), Simcoe, 9.vi.1939, G.E. Shewell, CNC480808, CNC480812 (1♂ 1♀, CNC), Smith’s Falls, 22.vi.1984, J.R. Vockeroth, CNC480816 (1♀, CNC), Strathroy, 9.viii.1916, H.G. Crawford, CNC480809 (1♂, CNC), **QC**: Wakefield, 9.vii.1946, G.E. Shewell, CNC480814 (1♀, CNC). **USA. CO**: Middle St., Vrain Cr., 3mi SW Raymond, 2499 m, 11.viii.1961, J.G. Chillcott, CNC480811 (1♀, CNC), **CT**: Redding, 12.viii.1930, A.L. Melander (1♂, USNM), **DE**: Gumboro, 2.viii.1952, C. Sabrosky (1♂, USNM), **IA**: Ames, 10.vii.1947, A.R. Brooks, CNC480817, CNC480818 (1♂ 1♀, CNC), **MI**: Leelanau Co., 22.vi.1937, C. Sabrosky (4♂, USNM), **NC**: Macon Co., Highlands Lake Revenel, 17.vi.1986, W.W. Wirth, Malaise trap (1♂, USNM), **NY**: Orleans Co., Albion, Burma Woods, 11.vi.1963, W.W. Wirth (1♂, USNM), **OH**: Columbiana Co., Beaver Creek S.P., 40°43.8'N, 80°36.5'W, 7.vii.1976, B.A. Steinly (1♂, USNM), Champion Co., Kiser Lake S.P., 41°22.9'N, 82°19.0'W, 22.ix.1976, B.A. Steinly (1♂, USNM), Lorain Co., Vermillion River, Mill Hollow C.P., 41°22.9'N, 82°19.0'W, 22.ix.1976, B.A. Steinly, grass shore, temporary pool, 200 net sweeps (2♂, USNM), **PA**: Erie Co., Lake Erie, Presque Isle S.P., 42°08.3'N, 80°08.5'W, 6.v.1977, B.A. Steinly (1♂, USNM), **TN**: Knoxville, Univ. Farm, 20.v.1957, J.R. Vockeroth, CNC480807 (1♂, CNC), **VA**: Montgomery Co., Jefferson Nat. Forest, Poverty Creek Trail, 15.v.1997, D.C. Caloren (1♂, DEBU), Giles Co., Mountain Lk. Biol. Stn., trail to Bear Cliff, 23.v.1997, D.C. Caloren (1♂, DEBU), M. Lk. Biological Stn., 37°22'31"N, 80°31'18"W, Malaise trap, 13–26.v.2001, Sylvatica Pond, J. Knopp (1♂, DEBU), Big Meadows, 1.vii.1939, A.L. Melander (1♀, USNM).

##### Cerodontha (Dizygomyza) morosa

Taxon classificationAnimaliaDipteraPhytomyzinae

(Meigen)

Figs 105 , 529–534

Agromyza morosa Meigen, 1830: 170. Becker 1902: 338.
Agromyza hyalipennis Meigen, 1838: 397. Schiner 1864: 306; Becker 1902: 338. Nowakowski 1973 [synonymy?].
Agromyza grossicornis Zetterstedt, 1860: 6456. Strobl 1893: 135, 1894: 141. Nowakowski 1973 [synonymy?].
Dizygomyza morosa . Hendel 1920: 132.
Dizygomyza (Dizygomyza) morosa . Hendel 1931: 90.
Phytobia (Dizygomyza) morosa . Frick 1959: 385.
Cerodontha (Dizygomyza) morosa . Nowakowski 1967: 644; Spencer 1969: 121; Spencer and Steyskal 1986b: 102; Papp and Černý 2016: 165; Eiseman and Lonsdale 2018: 44; Eiseman et al. 2021: 22.

###### Description

**(Fig. 105).** Wing length 2.3–2.8 mm (♂), 2.4–2.8 mm (♀). Length of ultimate section of vein M4 divided by penultimate section: 1.2–1.4. Eye height divided by gena height: 5.4–11.8. Male first flagellomere much enlarged, circular or slightly higher than long, and covered with long, whitish hairs that end before base; female first flagellomere not enlarged as for male, but with slightly longer whitish hairs anteromedially. Arista slightly thickened on basal 1/4–1/3. Fronto-orbital plate weakly visible laterally, most prominent anteriorly, widest medially, not more than 1/5 width of frons. Lunule large, broadly semi-circular with lateral margin meeting fronto-orbital plate; slightly narrower in female. Posterior ocelli slightly displaced.

***Chaetotaxy***: Two ori (anterior seta slightly shorter to 2/3 length posterior ori); two ors. Orbital setulae erect to reclinate, in one sparse row. Postocellar and ocellar setae subequal to fronto-orbital setae, but fronto-orbitals sometimes reduced to 1/2 of normal length. Four dorsocentral setae varying in length, sometimes only slightly decreasing in length anteriorly, but anterior seta as small as 1/3 length posterior seta.

***Colouration***: Setae dark brown. Antenna brown to dark brown with first flagellomere darker; pedicel and scape dark brown in female; frons brown to dirty yellowish brown with greyish pruinosity; fronto-orbital plate dark brown, but sometimes brown (not yellowish) with darker pigment reduced to posterolateral vestige; ocellar triangle dark brown, slightly larger than tubercle, confluent with dark brown margin along back of head; dark brown spot in posterolateral corner of frons reaching base of inner vertical seta; slightly paler triangular region bordering ocellar triangle; lunule smooth, velvety greyish; clypeus, venter of gena and face dark brown; gena and frons between triangle and fronto-orbital plates with minute brown pits. Thorax dark brown with notopleuron and postpronotum slightly paler brown with posterolateral corners sometimes yellowish to yellow; with greyish pruinosity that is strong on notum and dorsally on pleuron. Calypter margin and hairs yellowish white. Wing veins brown, yellowish white from base to basal cells. Halter yellow. Legs dark brown with apex of fore femur light yellow for length equal to width of femur; apices of mid and hind femora varying from slightly yellowish to broadly light yellow as seen on fore leg.

***Genitalia***: (Figs 529–534) Epandrium with strong process above anus that is constricted basally. Subepandrial sclerite with transverse dorsal bar that is broken medially and with one pair of setae; ventral lobe long, dark, relatively straight and with minute outer-apical point. Surstylus fused to epandrium, directed inwards, with double row of many long tubercle-like setae. Phallophorus dorsally fused to dorsomedial plate of mesophallus. Mesophallus medially split into curved, pointed arms, with right lateral arm elongate and mirroring left sclerite of hypophallus. Left sclerite of hypophallus dark, rod-like and with outer carina that can be pronounced or much reduced; right sclerite apparently absent. Paraphallus clear, lobate. Mesophallus and distiphallus narrow, elongate, and dark. Mesophallus with slight apical swelling. Distiphallus divided into one pair of subparallel S-shaped tubules; basal curve very small, shallow; apical curve longer than mesophallus, sometimes recurved apically (curve less pronounced in smaller specimens) and with middle slightly to more *obviously* flattened/straightened. Ejaculatory apodeme relatively dark, with stem expanding into broad blade that is slightly clearer marginally; base of sperm pump and base of duct pigmented.

###### Host.

Cyperaceae – *Carex*.

###### Distribution.

**Canada**: NB*, ON*, QC* [previous records considered *magnicornis* (Spencer and Steyskal 1986b)]. **USA**: CA, DE*, MA, MO, NC*, OH*, OK (tentatively identified females; Eiseman et al. 2021), PA*, TN*, VA*, WV*; records from IL, IN, MD, MI and SD (Frick 1959; Priest et al. 2020) require verification. Europe, Canary Islands, China, Japan, North Korea, Russia, India, Philippines (Papp and Černý 2016).

###### Type material.

***Holotype* [*morosa*]: Germany.** [not given] (1♂, NMW). [Not examined]

***Holotype* [*grossicornis*]: Sweden.** Lappland, 13.viii.1855 (1♂, ZIL). [Not examined]

***Holotype* [*hyalipennis*]: Germany.** Stollberg, Sachsen, coll. Meigen (1♂, MNHN). [Not examined]

###### Material examined.

**Canada. NB**: Kent Co., Sainte-Anne-de-Kent, 46°34'N, 64°47'W, 28–29.vii.2013, O. Lonsdale, CNC480850 (1♀, CNC), **ON**: Normandale, 42°42'N, 80°19'W, 22.v.1956, J.R. Vockeroth, CNC480868, CNC480869 (1♂ 1♀, CNC), 24.v.1956, CNC480870 (1♀, CNC), 27.v.1956, CNC480867 (1♂, CNC), Ottawa, Dow’s Swamp, 3.vi.1958, L. Smith, CNC480865 (1♀, CNC), Ottawa, 19.vi.1954, W.R.M. Mason, CNC480866 (1 ex, CNC), 19.vii.1954, CNC480858 (1♂, CNC), 26.vii.1959, J.R. Vockeroth, CNC480862 (1♂, CNC), 30.v.1958, CNC480864 (1♀, CNC), 31.v.1959, CNC480860 (1♂, CNC), 9.vi.1958, CNC480861, CNC480863 (1♂ 1♀, CNC), 8.vii.1952, G.E. Shewell, CNC480859 (1♂, CNC), **QC**: Old Chelsea, summit of King Mt., 350 m, 21.vi.1959, J.R. Vockeroth, CNC480857 (1♀, CNC), Old Chelsea, 11.viii.1959, C.H. Mann, CNC480855 (1♂, CNC), 13.ix.1956, J.R. Vockeroth, CNC480854 (1♂, CNC), 16.ix.1958, CNC480856 (1♀, CNC), sweeping, *Pediculariscanadensis* L., 14.v.1987, J.R. Vockeroth, CNC480851 (1♂, CNC), Perkins Mills, 25.viii.1949, G.E. Shewell, CNC480853 (1♀, CNC). **USA. DE**: New Castle Co., Newark, Rittenhouse, 6.ix.2003, K. Bennett, sweeping (1♀, UDCC), **MA**: Berkshire Co., Lenox, Mahanna Cobble, 23.vi.2016, C.S. Eiseman, *Carexgracillima* em. 28.vi.2016, #CSE2644, CNC634778 (1♂, CNC), Berkshire Co., Lenox, Mahanna Cobble, 23.vi.2016, C.S. Eiseman, *Carexhitchcockiana*, em. 30.vi-4.vii.2016, #CSE2678, CNC654235, CNC654236 (1♂ 1♀, CNC), **MD**: Lavale, 9.v.1970, G. Steyskal (3♂, USNM), Montgomery Co., Bethseda, 5.v.1968, L.V. Knutson (1♂, USNM), 9.iv.1968, G. Steyskal (5♂, USNM), 13.iv.1968, G. Steyskal (1♂, USNM), Colesville, 11.vii.1974, W.W. Wirth (1♂, USNM), **NC**: Grt. Sm. Mt. Nat. Park, Clingman’s Dome, 6.viii.1957, C.J. Durden, CNC480830, CNC480844–480849 (5♂ 2♀, CNC), Highlands, 14.vii.1957, W.R. Richards, CNC480843 (1♀, CNC), **OH**: Hancock Co., Rocky Ford River, 41°06.7'N, 83°45.6'W, 21.ix.1976, B.A. Steinly (1♂, USNM), **PA**: Allegheny Co., nr. Clairton, 4.vii.1997, C.R. Bartlett, sweeping (1♀, UDCC), Chester Co., Avondale, Stroud Water Rsch. Ctr., 15.ix.2000, A.L. Park, sweep net (2♀, UDCC), **TN**: Gr. Sm. Mt. Nat. Park, Collins Gap, 1737 m, 22.viii.1957, J.G. Chillcott, CNC480842 (1♀, CNC), Gr. Sm. Mt. Nat. Park, Indian Gap to Clingman’s Dome, 1584–2011 m, 6.viii.1957, J.G. Chillcott, CNC480829, CNC480832–480840 (8♂ 2♀, CNC), 6.viii.1957, CNC480841 (1♂, CNC), **VA**: Giles Co., Mountain Lake, 1158 m, sedge meadows, 31.v.1962, J.G. Chillcott, CNC480852 (1♂, CNC), Great Smoky Mt. N.P., NC Tenn., Indian Gap, 1584 m, 23.v.1957, W.R.M. Mason, CNC480831 (1♂, CNC), Giles Co., Mountain Lake Biol. Stn., 37°22'31"N, 80°31'18"W, 24.v.2001, O. Lonsdale (1♀, DEBU), Giles Co., Mountain Lake, 7.ix.1976, G.C. Steyskal (2♂, USNM), Northampton Co., Kiptopeke, 4–6.x.1986, W.E. Steiner et al., Malaise trap, dunes between cliff and beach (1♂, USNM), Chain Bridge, 20.iv.1924, J.R. Malloch (1♂, USNM), **WV**: White Sulfur Springs, 16.vi.1970, G. Steyskal (1♂, USNM).

###### Tentatively identified material.

**USA. OK**: Payne Co., Marena, 28.v.2016, em. 8–13.vi.2016, M.W. Palmer, ex *Carexfestucacea*, #CSE2568, CNC634779 (1♀, CNC), Mehan, 36.014339°N, 96.996744°W, 6.v.2016, em. 8.v.2016, M.W. Palmer, ex *Carexfestucacea*, #CSE2665, CNC634805 (1♀, CNC).

##### Cerodontha (Icteromyza)

Taxon classificationAnimaliaDipteraPhytomyzinae

Hendel

Dizygomyza (Icteromyza) Hendel, 1931: 51. Type species: Agromyzageniculata Fallén 1823, by original designation.
Phytobia (Icteromyza) . Frick 1952a: 392, 1959: 385.
Cerodontha (Icteromyza) . Nowakowski 1962: 102, 1967: 654, 1973: 30; Spencer 1969: 137; Spencer and Steyskal 1986b: 88; Zlobin 2000: 52, 2007d: 179; Boucher 2008: 557, 2012: 124.

The subgenusIcteromyza was most recently treated by Boucher, who first revised those species occurring in the north (Boucher 2008), and then treating the entire Nearctic fauna (Boucher 2012). These studies were preceded by Zlobin (2000, 2007d) and Nowakowski’s (1962, 1967, 1973) Palaearctic treatments, and of course, Spencer and Steyskal’s (1986b) treatment of the fauna of the USA. More than 30 species are known, including 12 in the Nearctic, of which only two occur in the Delmarva states: *C.longipennis* (Loew) and *C.vockerothi* Boucher.

In this subgenus, the antennae are widely separated (but less so than in *Dizygomyza*), the lunule is large, semi-circular, and often bulging, the ocellar triangle extends far anteriorly towards the lunule, and the frons and distal regions of the legs are often yellow, at least in part. Larvae mine in Cyperaceae, the anterior spiracles form long knob-like projections and the hind spiracles are displaced ventrally; puparia have a ventral curve posteriorly (Nowakowski 1973; Spencer and Steyakal 1986b; Zlobin 2000).

### ﻿Key to the Delmarva Cerodontha (Icteromyza)

**Table:** 

1	One or 2 ori. Palpus, frons and calypter hairs yellow (Fig. 103). Distal 35–50% of femora light yellow (sometimes reduced on mid and hind legs). Distiphallus long, strongly curved backwards (Figs 537, 538)	***C.longipennis* (Loew)**
–	Three ori. Palpus, frons and calypter hairs brown. Only apex of femora light yellow. Distiphallus as long as mesophallus, angled dorsally (Figs 539, 540)	***C.vockerothi* Boucher**

#### Species descriptions

##### Cerodontha (Icteromyza) longipennis

Taxon classificationAnimaliaDipteraPhytomyzinae

(Loew)

Figs 103 , 535–538

Agromyza longipennis Loew, 1869: 48. Melander 1913: 255; Malloch 1913: 296, 1934: 478.
Dizygomyza (Icteromyza) longipennis . Hendel 1931: 56 [as possible synonym of *lineella* Zetterstedt].
Phytobia (Icteromyza) longipennis . Frick 1952a: 393, 1959: 386.
Cerodontha (Icteromyza) longipennis . Spencer 1969: 140, 1990: 346; Sehgal 1971: 324; Spencer and Steyskal 1986b: 89; Boucher 2012: 139; Eiseman and Lonsdale 2019: 10.

###### Description

**(Fig. 103).** Wing length 1.6–2.6 mm (♂), 2.5–2.9 mm (rarely 1.9–2.2 mm) (♀). Length of ultimate section of vein M4 divided by penultimate section: 1.0–1.1. Eye height divided by gena height: 5.6–7.7. Lunule semi-circular, slightly higher than wide. Fronto-orbital plate not defined. Ocellar triangle ill-defined, apparently ending at or before lunule.

***Chaetotaxy***: Two ori (anterior seta slightly reduced to absent, but usually 1/2–2/3 length); two ors. Ocellar and postocellar setae slightly longer than fronto-orbitals. Four dorsocentral setae, slightly decreasing in length anteriorly, but sometimes two anterior setae both short. Acrostichal seta absent. Apparently six irregular rows of acrostichal setulae ending in front of level of posterior dorsocentral.

***Colouration***: Body subshiny with frons slightly velvety. Setae dark brown with paler shine. Antenna dirty orange to brownish, with pedicel browner and distal margin yellow, and male first flagellomere browner on basal 1/2 of inner surface and along dorsal margin; female first flagellomere dark brown to brown with orange tint that may predominate along base; frons light yellow, becoming whitish anteriorly on fronto-orbital plate; ocellar spot larger than tubercle, dark brown; posterolateral corner of frons dark brown to base of inner vertical seta or slightly beyond; dark brown posteriorly to base of posterior or anterior ors, with pigment often fading to absent in front of posterior ors; lunule light yellow; occiput, gena, parafacial and face yellowish white; mouthparts, including palpus yellow; clypeus brown with centre paler brown to yellow; back of head dark brown. Thorax dark brown with moderate greyish pruinosity. Calypter margin and hairs light yellow. Wing veins brown with base yellow. Halter yellow. Legs dark brown with tibiae and tarsi paler brown; distal 1/2 of coxae variably yellowish, at least apically on mid coxa; distal 35–50% of femora light yellow, but this sometimes reduced to 25% on mid and hind legs; tibiae narrowly yellow basally. Abdomen dark brown.

***Genitalia***: (Figs 535–538) Epandrium with process above anus well-developed. Surstylus fused to epandrium, with four to seven tubercle-like setae. Cercus large, prominent. Lobe of subepandrial sclerite dark, tapering apically. Hypandrium tapering to a point. Basiphallus with right lateral process emerging medially. Hypophallus narrow, rod-like. Paraphallus absent. Mesophallus very narrow, elongate, cylindrical, constricted medially and with abruptly narrower, darker base; narrowed basal section of variable length, ranging from 1/4–1/3 length of segment. Distiphallus divided into one pair of very narrow tubules that become paler apically and are slightly flared at opening; broadly arched backwards, sometimes reaching midpoint of mesophallus. Ejaculatory apodeme with moderately large blade that becomes paler marginally; sperm pump with basal pigment. See Boucher (2012) for discussion of variation of northern and western specimens.

###### Host.

Cyperaceae – *Carexpseudocyperus* L. Juncaceae – *Juncusxiphioides* E. Mey.

###### Distribution.

**Canada**: AB, BC, MB, NT, NS, ON, QC, SK. **USA**: AK, AR, CA, CT, FL, IA, ID, IL, IN, KS, LA, MA, MD, ME, MI, MN, MS, MT, NC, NE, NH, NJ, NV, NY, OK, OR, PA, SD, TN, TX, VA, VT, WA, WI, WV, WY.

###### Type material.

***Holotype*: USA. DC.** “Loew coll.”, Type No. 13436 (1♀, MCZ). [Not examined]

###### Material examined.

**Canada. AB**: Lethbridge, 5.vi.1929, J.H. Pepper, CNC480922 (1♂ CNC), McMurray, 6.viii.1963, G.E. Ball, CNC480978 (1♀, CNC), **BC**: Terrace, 10 mi N, 16.vii.1960, B. Heming, CNC481008 (1♀, CNC), **MB**: Aweme, 24.viii.1916, N. Criddle, CNC480933 (1♂ CNC), 27.viii.1917, CNC480905, CNC480934, CNC480982 (2♂ 1♀, CNC), 28.viii.1917, CNC480981 (1♀, CNC), 4.ix.1923, H.A. Robertson, CNC480935 (1♂ CNC), Treesbank, 18.x.1927, N. Criddle, CNC480979 (1♀, CNC), **NB**: St. Andrews, 8.vii.1957, G.E. Shewell, CNC480977 (1♀, CNC), **NT**: Yellowknife, Kam Lake, 20.vi.1966, G.E. Shewell, CNC480908 (1♂ CNC), **ON**: Algonquin Provincial Park, Whitefish Lake Group Campground, 45°33'47.01"N, 78°26'13"W, 414 m, 28.vi-2.vii.2008, J.andA. Skevington, Malaise trap, CNC339240 (1♀, CNC), Ottawa, Stony Swamp, Beaver Trail, 45°18'0"N, 75°49'16"W, 13.vi.2015, O. Lonsdale, CNC441040, CNC441081, CNC441097 (2♂ 1♀, CNC), Sudbury Dist., Killarney Provincial Park, Lake of the Woods Trail, 46°6'36"N, 81°11'48"W, 2.ix.2014, O. Lonsdale, CNC380796 (1♀, CNC), Bell’s Cor., 21.ix.1951, J.F. McAlpine, CNC480880, CNC480912, CNC480971 (2♂ 1♀, CNC), Bells Corners, 28.vi.1971, H.J. Teskey, CNC480915 (1♂ CNC), Constance Bay Rd., Harwood Plains, 19.vii.1967, G.E. Shewell, CNC480972, CNC480973 (2♀, CNC), Grand Bend, 15.vii.1939, G.E. Shewell, CNC480970 (1♀, CNC), Marmora, 12.viii.1952, J.F. McAlpine, CNC481014 (1♀, CNC), on weeds by water, 7.viii.1952, CNC481015 (1♀, CNC), Mattawa, 16.vi.1987, J.R. Vockeroth, CNC480911 (1♂ CNC), Metcalfe, 30.vii.1983, B.E. Cooper, CNC480907 (1♂ CNC), Midland, 20.v.1959, J.G. Chillcott, CNC480909 (1♂ CNC), 20.viii.1955, J.G. Chillcott, CNC480969 (1♀, CNC), Miners B., 26.v.1939, G.E. Shewell, CNC480920, CNC480960–480962 (1♂ 3♀, CNC), Newtonville, 26.ix.1983, J.R. Vockeroth, CNC481011 (1♀, CNC), Normandale, 42°42'N, 80°19'W, 2.vi.1956, J.R. Vockeroth, CNC480963, CNC480964 (2♀, CNC), Ottawa, Black Rapids, 28.vii.1959, J.R. Vockeroth, CNC481013 (1♀, CNC), Ottawa, 12.vi.1957, J.G. Chillcott, CNC480872 (1♂ CNC), 14.v.1957, CNC480879, CNC480954 (1♂ 1♀, CNC), 19.vi.1957, CNC480951 (1♀, CNC), 23.v.1957, CNC480953 (1♀, CNC), 26.v.1958, CNC480878, CNC480952 (1♂ 1♀, CNC), 12.vii.1956, J.R. Vockeroth, CNC480950 (1♀, CNC), 2.vi.1958, CNC480946 (1♀, CNC), 3.vi.1958, CNC480873–480877, CNC480947–480949 (5♂ 3♀, CNC), 19.vii.1946, G.E. Shewell, CNC480871 (1♂ CNC), Point Pelee, 9.ix.1954, W.R. Mason, CNC480966, CNC480967, CNC480968 (3♀, CNC), W.R.M. Mason, CNC480924, CNC480925 (2♂ CNC), Port Severn, 3mi N, Black spruce bog, 18.v.1959, J.G. Chillcott, CNC480910, CNC480917, CNC480965 (2♂ 1♀, CNC), Pr. Edward Co., Outlet Beach, 14.viii.1968, J.R. Vockeroth, CNC480914 (1♂ CNC), Pt. Pelee Nat. Pk., 1.ix.1983, S. Marshall, C. Logan and S. Grigsby, dry Malaise, CNC480916 (1♂ CNC), Rockport, 12.v.1959, J.R. Vockeroth, CNC481012 (1♀, CNC), Rondeau Park, 7.ix.1954, W.R.M. Mason, CNC480926, CNC480927, CNC480928 (3♂ CNC), Simcoe, 13.vi.1939, G.E. Shewell, CNC480929 (1♂ CNC), 24.vi.1939, CNC480958 (1♀, CNC), 29.v.1939, CNC480930 (1♂ CNC), 30.v.1939, CNC480931, CNC480932 (2♂ CNC), 6.vi.1939, CNC480959 (1♀, CNC), St. Williams, 42°40'N, 80°25'W, 23.v.1956, J.R. Vockeroth, CNC480919 (1♂ CNC), Thornhill, 30.v.1964, J.R. Vockeroth, CNC480881–480884, CNC480904, CNC480918, CNC480955–480957 (6♂ 3♀, CNC), **QC**: Gatineau Park, Lac Philippe, 45°36'N, 76°0'W, 23.v.2011, O. Lonsdale, CNC481032 (1♀, CNC),Beech Grove, 15.v.1961, J.F. McAlpine, CNC480975 (1♀, CNC), Beechgrove, 45°39'N, 76°8'W, 5.vii.1984, J.R. Vockeroth, CNC480906, CNC481010 (1♂ 1♀, CNC), Cap Rouge, 4.vii.1953, R. Lambert, CNC480974 (1♀, CNC), Wakefield, 9.vii.1946, G.E. Shewell, CNC480921, CNC480976 (1♂ 1♀, CNC), **SK**: Hudson Bay, 15.ix.1959, J.R. Vockeroth, CNC481009 (1♀, CNC), Waskesiu Lake, 12.vii.1939, A.R. Brooks, CNC480923 (1♂ CNC), Yorkton, 15.ix.1959, J.R. Vockeroth, CNC480980 (1♀, CNC). **USA. IL**: 5mi S Bath, 21.v.1953, J.F. McAlpine, CNC480890 (1♂ CNC), Champagne Co., 26.iv.1926, J. Savage, CNC480983 (1♀, CNC), Champagne, 3.x.1956, J.F. McAlpine, CNC480985–480987 (3♀, CNC), Champaigne, 2.x.1956, J.F. McAlpine, CNC480887 (1♂ CNC), nr. Forest City, Mason St. Forest, 21.v.1953, J.F. McAlpine, CNC480984, CNC480888 (1♂ 1♀ CNC), **KS**: Lawrence, Nat. Hist. Res., 28.iv.1956, J.G. Chillcott, CNC480886 (1♂ CNC), **LA**: Alexandria, 11 mi SW, Palmetto swamp, 26.iii.1960, J.G. Chillcott, CNC480899–480903, CNC480988–480990 (5♂ 3♀, CNC), **MN**: Crookston, river valley, 21.v.1960, J.G. Chillcott, CNC481016 (1♀, CNC), **NC**: Highlands, 1158 m, 23.viii.1957, J.G. Chillcott, CNC481006 (1♀, CNC), 27.viii.1957, CNC481007 (1♀, CNC), 10.v.1957, J.R. Vockeroth, CNC480940 (1♂ CNC), 16.vi.1957, CNC481001 (1♀, CNC), 5.v.1957, CNC480885, CNC480938, CNC480939, CNC481002–481005 (3♂ 4♀, CNC), 6.v.1957, CNC480891 (1♂ CNC), 1158 m, leaf mine in submerged Carex in stream, 8.vii.1957, CNC480889–481000 (1♂ 2♀, CNC), **PA**: Union Co., Lewisburg, 26.viii.1981, J.R. Vockeroth, CNC480936, CNC480937 (2♂ CNC), **TN**: East Ridge, 9.iv.1952, G.S. Walley, CNC480945 (1♂ CNC), 9.v.1952, CNC480997, CNC480998 (2♀, CNC), **TX**: Big Bend N.P., Oak Springs, 1371 m, 1.v.1959, J.F. McAlpine, CNC480894 (1♂ CNC), Big Bend N.P., Oak Spring, 1.v.1959, J.F. McAlpine, CNC480895 (1♂ CNC), Brazos Co., College Station, 26.iii.1966, D.M. Wood, CNC480892, CNC480893, CNC480991 (2♂ 1♀, CNC), Kerrville, cedar, 1.iv.1959, J.F. McAlpine, CNC480913, CNC480941–480944, CNC480992, CNC480993 (5♂ 2♀, CNC), **VA**: Giles Co., Mountain Lake, 1158 m, 26.v.1962, J.G. Chillcott, CNC480995 (1♀, CNC), Giles Co., Stony Creek, 609 m, 26.v.1962, J.R. Vockeroth, CNC480994 (1♀, CNC), Patrick Co., Fairy Stone St. Pk., 304 m, 30.v.1962, J.R. Vockeroth, CNC480896–480898, CNC480996 (3♂ 1♀, CNC).

##### Cerodontha (Icteromyza) vockerothi

Taxon classificationAnimaliaDipteraPhytomyzinae

Boucher

Figs 539 , 540

Cerodontha (Icteromyza) vockerothi Boucher, 2012: 145.

###### Description.

Wing length 2.2 mm (♂), ~2.9 mm (♀). Length of ultimate section of vein M4 divided by penultimate section: 1.2. Eye height divided by gena height: 6.9–5.0. Frons slightly projecting anteriorly. Fronto-orbital plate ill-defined; ocellar triangle ill-defined, appearing to nearly reach lunule. Arista densely pubescent, narrow along length. First flagellomere small, circular. Lunule semi-circular, slightly higher than wide.

***Chaetotaxy***: Three thin ori (as long as ors or ~ 3/4 length, with anterior setae often shortest); two stout ors. Ocellar and postocellar setae slightly longer than fronto-orbitals. Four dorsocentral setae, decreasing in length anteriorly; second dorsocentral apparently shifted anteriorly. Four scattered rows of acrostichal setulae reaching level of third dorsocentral.

***Colouration***: Setae dark brown. Body subshiny with frons micropubescent, almost velvety. Frons brown with faint orange tint, becoming darker posteriorly to vertex and laterally along fronto-orbital plate past ors, paler along fronto-orbital plate anteriorly; dark brown spot encompassing ocellar tubercle; lunule contrastingly light yellow; back of head, clypeus and palpus dark brown; lunule and face light yellow; dorsum of gena, parafacial and narrow region around base of scape orange with brown tint. Thorax dark brown. Calypter margin yellow, hairs light brown. Wing veins dark brown, becoming yellowish orange to base. Halter yellow. Legs dark brown with apices of femora light yellow for length equalling width of femur; bases of tibiae narrowly light yellow. Abdomen dark brown.

***Genitalia***: (Figs 539, 540) (from Boucher 2012: figs 83–86). Hypophallus appearing rod-like, apically split. Paraphallus simple, rod-like, small. Mesophallus subequal in length to distiphallus, narrow with slight basal swelling. Distiphallus split into one pair of narrow tubules that are divergent and curved upwards. Ejaculatory apodeme small with narrow blade.

###### Host.

Unknown.

###### Distribution.

**Canada**: ON. **USA**: VA.

###### Type material.

***Holotype*: USA. VA**: Arlington, vi.1938, J.R. Malloch (1♂, USNM). [Not examined]

***Paratype*: Canada. ON**: Mer Bleue, 19.vii.1963, in marsh, J.R. Vockeroth (1♀, CNC).

##### Cerodontha (Poemyza)

Taxon classificationAnimaliaDipteraPhytomyzinae

Hendel

Dizygomyza (*Poëmyza*) Hendel, 1931: 35. Type species AgromyzapygmaeaMeigen 1830, by original designation.
Phytobia (*Poëmyza*). Frick 1952a: 391, 1959: 379.
Cerodontha (Poemyza) . Nowakowski, 1962: 102, 1967: 645, 1973: 73; Spencer 1969: 127; Spencer and Steyskal 1986b: 93.

The lunule of *Poemyza* is characterised as being relatively high and narrow, often being much higher than wide. This is exaggerated in many species where the fronto-orbital plate is produced over the lunule’s wider base. Some species, such as *C.inconspicua* (Malloch), however, have a shorter lunule similar to that of species in the subgenusButomomyza, but the texture of the lunule in *Poemyza* species is pitted and bare (not flat and velvety) and the acrostichal seta is usually absent (not present). The buccal cavity between the arms of the clypeus is also densely covered with minute spicules, at least in Nearctic species, but this has not yet been confirmed for the global fauna. A similar lunule is seen in *Xenophytomyza*, which has a pointed first flagellomere similar to some *Poemyza*, but *Xenophytomyza* differs in lacking lateral scutellar setae.

### ﻿Key to the Delmarva Cerodontha (Poemyza)

**Table:** 

1	Calypter hairs and often calypter margin white	2
–	Calypter hairs light to dark brown; margin usually also dark	**4**
2	Postpronotum, notopleuron and lunule light yellow. First flagellomere with anterodorsal margin angled. Eye 4.3–5.0 × higher than gena at highest point. Epandrium with large transverse carina (Figs 559, 560)	***C.superciliosa* (Zetterstedt)**
–	Postpronotum, notopleuron and lunule brown. First flagellomere rounded. Eye 6.8–10.7 × higher than gena at highest point. Epandrium without carina	**3**
3	Wing length 2.3–3.4 mm. Anterior section of fronto-orbital plate up to 1/3 width of frons. Only apex of fore femur light yellow for length equal to width of femur apex; mid and hind femora with extreme apex fading to brownish or yellowish. Ocellar spot dark, well-defined. Bulb of mesophallus bulging ventrally over stem. Distiphallus slender, hooked with slender bell-shaped apex (Figs 543, 544)	***C.incisa* (Meigen)**
–	Wing length 1.8–2.2 mm. Anterior section of fronto-orbital plate not exceeding ¼ width of frons. Apices of all femora light yellow for length equal to width of femur apex. Ocellar spot ill-defined, sometimes faded. Bulb of mesophallus not strongly overlapping stem. Distiphallus stouter, straighter (Figs 557, 558)	***C.pygminoides* Spencer**
4	At least distal 1/3 of femora yellow. Fronto-orbital plate and ocellar triangle yellow in part	5
–	Only apex of fore femur yellow, sometimes with mid and hid femora also somewhat yellowish apically. Head entirely brown to dark brown (rarely with yellowish border around ocellar tubercle)	**6**
5	At least distal ¾ of femora yellow, sometimes only with narrow brown region at base. Fourth dorsocentral almost as small as surrounding setulae. Distiphallus straight	***C.kennethi* Zlobin**
–	Distal 1/3–1/2 of femora yellow. Fourth dorsocentral at least 1/3 length of first dorsocentral. Distiphallus strongly twisted to right (Figs 548, 549)	***C.muscina* (Meigen)**
6	Ring around eye shallow, not projecting anteriorly. First flagellomere rounded. Eye 6.0–8.3 × higher than gena at highest point. Six rows of acrostichal setulae. Hairs on calypter dark brown. Only left paraphallus distinct, directed laterally. Mesophallus stem bulging basally; apical bulb overlapping mesophallus. Distiphallus tubules mostly clear with lateral sclerotised band, ?-shaped with small apical cup (Figs 553, 554)	***C.pygmaea* (Meigen)**
–	Ring around eye very pronounced, projecting anteriorly. First flagellomere with shallow dorsal angle. Eye 3.0–4.1 × higher than gena at highest point. Four rows of acrostichal setulae. Hairs on calypter light brown. Paraphalli weakly developed, almost indistinct. Mesophallus narrow in lateral view; apical bulb small, globose. Distiphallus tubules dark, slightly curved at base, expanded apically into broad, dark cups (Figs 565, 566)	***C.ungula* sp. nov.**

#### Species descriptions

##### Cerodontha (Poemyza) incisa

Taxon classificationAnimaliaDipteraPhytomyzinae

(Meigen)

Figs 102 , 541–544

Agromyza incisa Meigen, 1830: 182.
Agromyza carbonella Zetterstedt, 1860: 6455. Hendel 1920 [synonymy].
Agromyza graminis Kaltenbach, 1874: 738. Hendel 1920 [synonymy].
Agromyza atra Meigen. Misidentification. Brischke 1881: 53.
Agromyza luctuosa Meigen. Misidentification. Strobl 1893: 134; Melander 1913: 254.
Agromyza angulata Loew. Misidentification. Malloch 1913: 304.
Dizygomyza incisa . Hendel, 1920: 135.
Dizygomyza (*Poëmyza*) incisa. Hendel, 1931: 38.
Phytobia (*Poëmyza*) incisa. Frick, 1952a: 392, 1959: 381.
Cerodontha (Poemyza) incisa . Nowakowski, 1962: 112, 1967: 651, 1973: 116; Spencer 1969: 128; Spencer and Steyskal 1986b: 95; Benavent-Corai et al. 2005: 12; Scheffer et al. 2007: 771; Papp and Černý 2016: 221; Scheffer and Lonsdale 2018: 87; Eiseman and Lonsdale 2018: 44.
Phytobia incisa . Kania, 1962: 66.

###### Description

**(Fig. 102).** Wing length 2.3–3.0 mm (♂), 2.6–3.4 mm (♀). Length of ultimate section of vein M4 divided by penultimate section: 1.7–2.0. Eye height divided by gena height: 6.8–10.7. First flagellomere ovate, slightly longer than high. Arista pubescent. Lunule as high as wide, with dorsum strongly tapering, narrow; minutely pitted. Fronto-orbital plate 1/4 width of frons along most of length, but widening anteriorly to cover base of lunule laterally, sometimes reaching 1/3 width of frons. Cheek evident anteriorly on gena.

***Chaetotaxy***: Two ori; two ors; slightly decreasing in length anteriorly, sometimes with additional ori on one side. Ocellar and postocellar setae subequal to fronto-orbitals. One strong row of ocellar setulae. One pair of prominent setae apically on each palpus. Four dorsocentral setae decreasing in length anteriorly, with fourth dorsocentral ~ 1/3 length of first dorsocentral; third dorsocentral sometimes also 1/3 length of first dorsocentral, sometimes duplicated (i.e., five dorsocentral setae). Approximately six rows of scattered acrostichal setulae. Acrostichal seta uncommonly present.

***Colouration***: Setae dark brown. Head dark brown with gena paler brown (excluding ventral margin) and inner margin of ocellar triangle and fronto-orbital plate sometimes narrowly yellow; centre of frons outside ocellar triangle and fronto-orbital plate with dense blackish pits that are also present sparsely on gena centrally; buccal cavity inside clypeus with minute dark brown spicules. Remainder of body mostly dark brown with apex of fore femur light yellow for length not exceeding width of femur apex; apex of mid and hind femora with much narrower brownish to yellowish region; base of fore tibia sometimes narrowly light yellow to yellowish, and fore tibia and tarsus often paler brown, especially on tarsus and apex of tibia; wing veins light brown, becoming yellow to base; halter light yellow; calypter entirely white.

***Genitalia***: (Figs 541–544) Epandrium with shallow supra-anal process. Surstylus directed inwards, fused to epandrium, with scattered short stout setae. Ventral process of subepandrial sclerite short, with broad stout base and narrow apical portion with outer wrinkles. Basiphallus fused to anteroventral margin of phallophorus, developed on left surface of shaft and narrowly along dorsomedial surface where it extends to become a narrow right dorsolateral bar; left distal margin continuing as narrow process flanking paraphallus. Hypophallus flat, broad, M-shaped, with thick basomedial ridge. Paraphallus only present on left side, elongate and directed laterally with margin irregular and base fused to mesophallus. Mesophallus tubular with base slightly swollen and distal section dorsoventrally compressed to narrow point of fusion with distiphallus; apically with bulging bilobed base that partially overlaps stem. Distiphallus tubules question mark-shaped, fused to mesophallus, as long as mesophallus, with narrow sclerotised band along inner-basal surface and band along outer surface that meets small, sclerotised apical cup.

###### Host.

Poaceae – *Agropyron*, *Agrostis*, *Alopecurus*, *Ammophila*, *Anthoxanthum*, *Avena*, *Briza*, *Bromus*, *Calamagrostis*, *Cinna*, *Coix*, *Dactylis*, *Deschampsia*, *Digitaria*, *Echinochloa*, *Elymus*, *Elytrigia*, *Festuca*; *Hierochloe*, *Holcus*, *Hordelymus*, *Hordeum*, *Leymus*, *Lolium*, *Melica*, *Milium*, *Molinia*, *Panicum*, *Pascopyrum*, *Phalaris*, *Phleum*, *Phragmites*, *Poa*, *Secale*, *Setaria*, *Trisetum*, *Triticum*, *Zea*, *Zizania* (Frick 1959; Spencer 1969; Spencer and Steyskal 1986b; Eiseman and Lonsdale 2018; Ellis 2021).

###### Distribution.

**Canada**: AB, NT, ON, QC, SK, YT. **USA**: AK, “widespread in the northern 1/2 of the united states” (Frick 1959), NC, WY. Europe, Turkey, Uzbekistan, Russia, China, Japan, Pakistan (Papp and Černý 2016).

###### Type material.

***Holotype* [*incisa***]: Germany [not given] (1♂, NMW). [Not examined]

***Holotype* [*graminis***]: Germany [not given]. [Not examined]

***Syntypes* [*carbonella*]: Sweden.** Skane [= Scania]: Kungsmarken between Fagelsang [Fogelsang] and Lund; Ostergotland: Bockestad [= Bokestad]. [Not examined]

###### Additional material examined.

**Canada. AB**: Banff, Johnston Canyon, 1432 m, 18.vii.1962, K.C. Hermann, CNC481039 (1♂, CNC), **BC**: Bowser, 5.vi.1955, R. Coyles, CNC481143 (1♀, CNC), Miracle Beach nr. Oyster River, 11.vi.1955, J.R. McGillis, CNC481145 (1♀, CNC), Oliver, 20.vi.1953, J.R. McGillis, CNC481144 (1♀, CNC), Osoyoos, 2 km S, 12.v.1989, A. Borkent, CD1034, CNC481126 (1♀, CNC), Salmon Arm, 6 km E, 28.vii.1989, A. Borkent, CD1061, CNC481125 (1♀, CNC), Summit Lake, Mi 392 Alaska Hwy., 1280 m, 21.vii.1959, R.E. Leech, CNC481147, CNC481149, CNC481150 (3♀, CNC), 31.vii.1959, CNC481148 (1♀, CNC), 1524 m, 6.vii.1959, CNC481146 (1♀, CNC), 1371 m, 2–4.vii.1959, CNC481153 (1♂, CNC), 1280 m, 31.vii.1959, CNC481086, CNC481154 (2♂, CNC), **NB**: Kouchibouguac N.P., 23.v.1977, Hanley and Cooper, Code-5113Q, CNC481082 (1♂, CNC), **NL**: St. John’s, Agric. Exp. Sta., 29.vii.1967, J.F. McAlpine, CNC481142 (1♀, CNC), **NT**: Salmita Mines, 64°5'N, 111°15'W, 4.vii.1953, J.G. Chillcott, CNC481122 (1♀, CNC), Yellowknife, 5.vi.1953, J.G. Chillcott, CNC481121 (1♀, CNC), **NS**: CBHNt. Pk., French L., slope fen, 24–30.vi.1984, H.J. Teskey, PG633770, CNC481083 (1♂, CNC), Cranberry I., Lockeport, 9.viii.1958, J.R. Vockeroth, CNC481124 (1♀, CNC), **ON**: Belwood, 23.vi.1968, D.H. Pengelly (1♀, DEBU), Ancaster, 26.vi.1955, O. Peck, CNC481114 (1♀, CNC), Atikokan, 4mi E on Hwy. 11, 5.vii.1978, H.J. Teskey, CNC481111 (1♀, CNC), Chatham, 25.vii.1928, Baird, 18333A, CNC481113 (1♀, CNC), Marmora, 2.vii.1952, J.R. Vockeroth, CNC481157 (1♂, CNC), Mer Bleue, 23.viii.2014, O. Lonsdale, CNC380695 (1♀, CNC), Milton, 7161 Appleby Line, 23°28'19"N, 79°54'40"W, 23–25.vii.2015, O. Lonsdale, CNC461884 (1♂, CNC), Ottawa, 695 Malibu Tr., 45°22'16.29"N, 75°42'57"W, 19.vii.2019, O. Lonsdale, leaf mine Triticeae em. 21.vii.2019, CNC1482941 (1♀, CNC), Ottawa, 20 mi NW, 17.v.1963, H. Rutz, CNC481081 (1♂, CNC), Ottawa, C. E. Farm, Arboretum, 29.vii.1962, W.G. Dore, ex leaf mine in *Zizaniaaquatica* L. CNC481056–481110, CNC481078, CNC481042 (3♂ 4♀, CNC), Ottawa, Can. Exp. Farm, W.G. Dore, leaf mine in Zizaniaaquaticavar.angustifolia em. 14.vii-18.viii.1962, CNC481043–481055, CNC481057–481075, CNC481077 (13♂ 20♀, CNC), Ottawa, 11.ix.1947, G.E. Shewell, CNC481106 (1♀, CNC), 14.v.1946, A.R. Brooks, CNC481076 (1♂, CNC), Ottawa, marshy river shore, 2.ix.1984, J.R. Vockeroth, CNC481137 (1♀, CNC), 26.viii.1984, CNC481155 (1♂, CNC), Ottawa, damp, second growth *Acer*-*Betula* wood, 20.vi.2003, J.R. Vockeroth, CNC481102 (1♀, CNC), 9.viii.1993, CNC481103 (1♀, CNC), 4.vii.2003, CNC481104 (1♀, CNC), 5.vii.1989, CNC481139 (1♀, CNC), Ottawa, 5.vi.1946, G.E. Shewell, CNC481041 (1♂, CNC), 6.v.1987, J.R. Vockeroth, CNC481105 (1♀, CNC), iv-vii.1956, J.R. Vockeroth, CNC481138 (1♀, CNC), Picton, 10.vii.1970, J.F. McAlpine, CNC481140 (1♀, CNC), Port Severn, 3mi N, Black spruce bog, 18.v.1959, J.G. Chillcott, CNC481141 (1♀, CNC), Simcoe, 26.vi.1939, G.E. Shewell, CNC481087 (1♂, CNC), 29.vi.1939, CNC481112 (1♀, CNC), Simcoe, 22.vi.1939, G.E. Shewell, CNC481079 (1♂, CNC), St. Lawrence Is. Nat. Park, McDonald Is., 12.vii.1976, W. Reid, Code 4174-N, CNC481101 (1♀, CNC), 22.viii.1976, Code 4492-V, CNC481100 (1♀, CNC), 5.viii.1976, A. Carter, Code 4334-T, CNC481099 (1♀, CNC), Thornhill, 30.v.1964, J.R. Vockeroth, CNC481080 (1♂, CNC), Wilno, ex *Pineus*-infested white pine, 16.v.1960, J.F. McAlpine, CNC481156 (1♂, CNC), **QC**: Abbotsford, 2.vii.1936, G.E. Shewell, CNC481118 (1♀, CNC), 20.viii.1936, CNC481117 (1♀, CNC), Breckenridge, 14.vii.1959, C.H. Mann, CNC481133, CNC481134 (2♀, CNC), Farnham, 7.vii.1937, G.E. Shewell, CNC481084, CNC481119 (1♂ 1♀, CNC), Hemmingford, 8.vii.1925, Hammond, leaf miner in Timothy, CNC481085, CNC481115 (1♂ 1♀, CNC), 9.vii.1925, CNC481116 (1♀, CNC), Kingsmere, 26.v.1958, J.G. Chillcott, CNC481136 (1♀, CNC), Old Chelsea, 16.v.1958, J.R. Vockeroth, CNC481135 (1♀, CNC), **SK**: 18 km S Maple Creek, by small clear stream, 49°44'31.93"N, 109°28'40.77"W, 10.vii.2005, J. R. Vockeroth, yellow pan trap, CNC287490 (1♂, CNC), Saskatoon, 9.v.1949, A.R. Brooks, CNC481123 (1♀, CNC), **YT**: North Fork Crossing, mi. 43, Peel Plt. Rd., 1066 m, 3.vii.1962, P.J. Skitsko, CNC481040 (1♂, CNC), Whitehorse, 24.viii.1959, R. Madge, CNC481120 (1♀, CNC). **Germany.** Mecklenburg-Vorpommern: Mecklenburg, Rostock 270, 10.viii.1932, H. Buhr, *Zeamays* CNC481098 (1♂, CNC). **Norway**. Heia, S. Grong NT, 8.vii.1972, *Phalariserundinacea* em. 23.vii.1962,em. 23.vii.1972, CNC481093, CNC481092 (2♀, CNC). **Spain**. Granada, 700 m, 10.vii.1960, J.R. Vockeroth, CNC481094 (1♀, CNC), Veleta, Sierra Nevada, N. slope, 2800–3000 m, 20.vii.1960, J.R. Vockeroth, CNC481095 (1♀, CNC), 2300–2550 m, 22.vii.1960, J.R. Vockeroth, CNC481097 (1♀, CNC), 2200 m, 30.vii.1960, J.R. Vockeroth, CNC481096 (1♀, CNC). **USA. AK**: Anchorage, 27.vi.1959, R.S. Bigelow, CNC481130 (1♀, CNC), 29.vi.1959, CNC481129 (1♀, CNC), Big Delta, 18.vi.1951, W.R.M. Mason, CNC481088 (1♂, CNC), Umiat, 11.vii.1959, J.E.H. Martin, CNC481089 (1♂, CNC), 12.vii.1959, CNC481091 (1♂, CNC), 13.vii.1959, CNC481131, CNC481132 (2♀, CNC), 23.vii.1959, R. Madge, CNC481090 (1♂, CNC), **CO**: Boulder, 1828 m, 4.vi.1961, B.H. Poole, CNC481152 (1♀, CNC), **DC**: “Dist Columbia”, 11.vi.1926, J.M. Aldrich (1♀, USNM), **DE**: New Castle Co., Port Penn, Port Penn Nature Trail, 7.ix.2006, sweeping, K. Styler (1♀, UDCC), **IA**: Howard Co., Hayden Prairie State Preserve, 15.vii.2015, em. 30.vii.2015, C.S. Eiseman, ex ?*Phalaris*, #CSE1910, CNC564724 (1♀, CNC), **IN**: LaFayette, 12.vii.1915, CNC481127 (1♀, CNC), **MA**: Franklin Co., Northfield, 263 Capt. Beers Plain Rd., 11.vii.2012, em. by 27.vii.2012, C.S. Eiseman, ex *Phalarisarundinacea*, CNC358500 (1♀, CNC), 276 Old Wendell Rd., 2.viii.2014, em. 11.viii.2014, C.S. Eiseman, ex *Phleumpratense*, #CSE1302, CNC384827–384829 (1♂ 2♀, CNC), Plymouth Co., West Bridgewater, 41.991852, -71.033550, 7.viii.2013, em. 17.viii.2013, C.S. Eiseman, ex *Cinnaarundinacea*, #CSE816, CNC392642 (1♀, CNC), Bridgewater, 16.viii.2013, em. 18–29.viii.2013, C.S. Eiseman, ex *Cinnaarundinacea*, #CSE826, CNC384722–384724 (2♂ 1♀, CNC), Worcester Co., Phillipston, along Burnshirt River (42.52149046, -72.09219652), 4.vii.2017, em. 15.vii.2017, C.S. Eiseman, ex *Calamagrostiscanadensis*, #CSE3955, CNC939733 (1♂, CNC), **MD**: Montg. Co., Clarksburg, Little Bennett Reg. Park, 21.ix.1990, W.E. Steiner, M.J. and R. Molineaux (1♂, USNM), Prince Georges Co., Patuxent Wildlife Research Center, 14.vii.1978, W.W. Wirth (1♂, USNM), Montgomery Co., Bethseda, 9.iv.1968, G. Steyskal (1♂, USNM), Bethseda, 27.viii.1981, G.C. Steyskal (1♂, USNM), Mg. Co., 4mi SW of Ashton, 25.iv.1987, G.F. and J.F. Hevel (2♀, USNM), 8.vi.1981, Malaise trap (1♀, USNM), Mg. Co., Camp Springs, 15.ix.1979, Malaise trap, G. Hevel (1♀, USNM), Montgomery Co., Forest Glen, 25.vi.1967, W.W. Wirth (1♀, USNM), Cab. John Br, 14.vi.1913, R.C. Shannon (1♀, USNM), **NC**: Macon Co., Highlands, Lake Ravenel, 15.vi.1986, Malaise trap, W.W. Wirth (1♀, USNM), Cecil Co., Fair Hill, 771 Hill Top Rd, 22.vii.2003, Malaise trap, M. Spellman (1♀, UDCC), Highlands, Whitesides Cove, 853 m, 11.viii.1957, J.G. Chillcott, CNC481151 (1♀, CNC), **NY**: Columbia Co., Ghent, Wolfe wild meadow, 17.vii.2015, C. Vispo, *Phalarisarundinacea*, em. 3.viii.2015, #CSE2172, CNC654350 (1♂, CNC), Payne Co., Mehan, 36.014339°N, -96.996744°W, 2.vi.2017, em. 21.vi.2017, M.W. Palmer, ex *Elymuscanadensis*, #CSE3935, CNC939907 (1♀, CNC), **TN**: East Ridge, 9.v.1952, G.S. Walley, CNC481128 (1♀, CNC), **WV**: Hardy Co., Lost R. St. Pk., 11.vii.1974, G.C. Steyskal (1♂, USNM), Morgan Co., near Great Cacapon, 3.vii.1983, G.F. and J.F. Hevel (1♀, USNM), **WY**: Battle L. Road, Sierra Madre Range, 2590 m, seepage area, 18.vii.1961, J.G. Chillcott, CNC481038 (1♂, CNC).

###### Tentatively identified material.

**USA. MA**: Nantucket Co., Nantucket, Squam Swamp, 27.vii.2017, em. by 22.viii.2017, C.S. Eiseman, ex *Juncustenuis*, #CSE4157, CNC939650 (1♀, CNC).

###### Comments.

*Cerodonthaincisa* is a distinct species externally, being relatively large and dark with an entirely white calypter, but the male genitalia reveal a close relationship with *C.pygmaea* and *C.superciliosa*, both of which are also quite easily diagnosed externally (see key). All three species have an M-shaped hypophallus with a medial carina, the left paraphallus is present and well-developed (the right paraphallus is also present but smaller in *C.superciliosa*), a mesophallus that is bulging basally and compressed distally before it extends into an apical chamber that bulges to extend over the stem. Furthermore, the tubules of the distiphallus are clear with sclerotisation limited to a small apical cup and one pair of lateral bands, the inner of which only extends to the medial curve. The left distal margin of the basiphallus is also extended past the mesophallus, but this is slightly reduced in *C.pygmaea*, and bifid in *C.superciliosa*.

##### Cerodontha (Poemyza) kennethi

Taxon classificationAnimaliaDipteraPhytomyzinae

Zlobin

Cerodontha (Poemyza) kennethi Zlobin, 1997: 89.
Cerodontha (Poemyza) kennethsi. Lapsus. Zlobin 1997: 89 [figure caption].

###### Description.

Wing length 1.7 mm (♂), 1.7–2.1 mm (♀). Length of ultimate section of vein M4 divided by penultimate section: 1.2–1.8. Eye height divided by gena height: 6.8–8.7.

Chaetotaxy and colour as described for C. (P.) *muscina*, except as follows: anterior ori reduced to absent; dorsocentral setae strongly decreasing in length anteriorly with fourth dorsocentral barely evident; fronto-orbital plate always at least partially brown anterior to anterior ors; cheek sometimes dark brown; apex of fore coxa yellow; basal 1/5–1/4 of femora brown with pigment on fore femur more reduced; tibiae and tarsi light brown with fore leg palest, and apical segments on mid and hind tarsi darker with apical segment sometimes brown. Paratypes with only extreme base of femora brown and notopleuron with distinct, large irregular light yellow spot.

***Genitalia***: Not examined. Illustrated in Zlobin (1997: figs 7–9).

###### Host.

Unknown.

###### Distribution.

**Canada**: ON. **USA**: MD*, NC*, PA, TX, VA*, WV*.

###### Type material.

***Holotype*: Canada. ON**: Renfrew Co., 15 km S Palmer Rapids, 27.vii.1986, R. Danielsson (1♂, ZIL). [Not examined]

###### Paratypes examined.

**USA. TX**: Kerrville, 21.iv.1959, J.F. McAlpine, CNC48115, CNC481160 (2♀, CNC).

###### Additional material examined.

**USA. MD**: P.G. Co., Camp Springs, 15.vii.1979, Malaise trap, G.F. Hevel (1♀, USNM), **NC**: Highlands, 16.viii.1957, W.R. Richards, CNC481161 (1♀, CNC), **VA**: Shenandoah, Big Meadows, 2.vii.1939, A.L. Melander (1♂, USNM), **WV**: Hardy Co., Lost River St. Park, 10.vii.1977, Malaise trap, W.W. Wirth (1♀, USNM).

###### Comments.

This species is highly similar in appearance to the relatively abundant *Cerodonthamuscina*, but can be differentiated by having the fore femur yellow for at least the distal 3/4 of its length, not the distal 1/2 or less. Newly examined material greatly expands the known distribution of this species in the northeastern United States.

##### Cerodontha (Poemyza) muscina

Taxon classificationAnimaliaDipteraPhytomyzinae

(Meigen)

Figs 101 , 545–549

Agromyza muscina Meigen, 1830: 177.
Agromyza marginata Loew, 1869: 49. Frick 1957 [synonymy].
Agromyza vittata Meigen. Misidentification. Strobl 1880: 37.
Dizygomyza (*Poëmyza*) muscina. Hendel, 1931: 44.
Phytobia (*Poëmyza*) muscina. Frick ,1952a: 392, 1957: 202, 1959: 382.
Phytobia (*Poëmyza*) marginata. Shewell, 1953: 468.
Cerodontha (Poemyza) muscina . Nowakowski, 1967: 649, 1973: 104 [lectotype designation]; Spencer 1969: 132; Spencer and Steyskal 1986b: 93; Benavent-Corai et al. 2005: 12; Scheffer et al. 2007: 771; Papp and Černý 2016: 235; Černý et al. 2020: 204.
Cerodontha muscina . Boucher, 2001: 614.

###### Description

**(Fig. 101).** Wing length 1.7–2.5 mm (♂), 1.4–2.3 mm (♀). Length of ultimate section of vein M4 divided by penultimate section: 1.2–1.8. Eye height divided by gena height: 5.4–7.3. First flagellomere short, ovate. Arista pubescent. Lunule appearing as high or higher than wide when fronto-orbital plates cover base laterally; surface minutely pitted. Fronto-orbital plate ~ 1/4 frons width, smooth, well-sclerotised, contrasting soft, often buckled medial section of frons.

***Chaetotaxy***: Two ori (sometimes additional ori present on one side); two ors; slightly decreasing in length anteriorly overall, even if one seta sometimes slightly longer or shorter; sometimes with only anterior ori shorter. One sparse, irregular row of orbital setulae. Four dorsocentral setae, decreasing in length anteriorly with fourth dorsocentral more abruptly shortened, being ~ 1/3–1/2 length of first dorsocentral. Approximately six irregular rows of acrostichal setulae. Acrostichal seta absent. One pair of prominent setae apically on each palpus.

***Colouration***: Setae dark brown. Antenna brown with first flagellomere dark brown and pedicel yellowish, but sometimes entirely dark brown; fronto-orbital plate light yellow to yellow, with variable light brown to brown pigment that is darker anteriorly; fronto-orbital plate sometimes appearing entirely yellow, but minute brownish spots always present at base of setae and setulae medially and anteriorly; frons with minute dark brown markings between fronto-orbital plates that are denser and darker anteriorly; ocellar spot dark brown, slightly larger than tubercle, enclosed within larger, smooth yellow triangle; back of head, posterolateral corner of frons to base of inner vertical, clypeus and mentum dark brown; gena yellow to brown with cheek light brown, ventral margin dark brown, and minute, sparsely arranged medial pits below cheek dark brown; parafacial dirty yellow; lunule, face and palpus brown to dark brown; buccal cavity within clypeus covered with dense dark brown spicules. Remainder of body dark brown, subshiny, with postpronotum and notopleuron slightly paler; distal 1/3–1/2 of femora yellow, fore femur always more extensively yellow with yellow region uncommonly extending below midpoint; tibiae and tarsi slightly paler with fore leg palest; some specimens from the north uncommonly darker with fore femur only yellow on distal 1/3 and mid and hind femora yellow for length slightly exceeding width of femur apex; abdomen sometimes yellowish laterally on tergites 1–3. Wing veins brown to light brown, whitish to yellow at base. Halter light yellow. Calypter margin light brown to whitish (uncommonly darker), and hairs brown.

***Genitalia***: (Figs 545–549) Epandrium with or without supra-anal process; shorter dorsally, fused to surstylus. Surstylus directed inwards with scattered stout, short setae. Ventral process of subepandrial sclerite with broad, stout base and highly reduced, rounded distal section. Phallophorus fused ventrally to basiphallus. Basiphallus sclerotised along left surface of shaft, and partially along venter; produced anterodorsally as short, flat process. Hypophallus divided into one pair of broad, flat, distally narrowed plates. Mesophallus and distiphallus with very distinct torsion to right and slight asymmetry. Mesophallus with slightly widened, uneven base; length of stem ~ 2 × width; apical chamber globular. Distiphallus tubules relatively short, strongly twisted to right, dark and with narrow, slightly differentiated cups. Sperm pump with faint basal sclerotised patch; ejaculatory apodeme with short base expanding into narrow base of blade, which is distally expanded with margin dark.

###### Hosts.

Poaceae – *Agropyron*, *Bromus*, *Dactylis*, *Echinochloa*, *Elytrigia*, *Ehrharta*, *Festuca*, *Hierochloe*, *Holcus*, *Hordeum*, *Lolium*, *Milium*, *Poa* (Ellis 2021).

###### Distribution.

**Canada**: AB, BC, MB, NB*, NL, NS, NT, NU, ON, QC, SK*, YT. **USA**: AK, CA, CT*, DC, DE*, GA, ID, IL, IN, MA, MD, MI, MN, NC, NH*, NY, OR, PA, TN, VA*, WA, WV*. Europe, Kyrgyzstan, Russia, South Korea (Černý et al. 2020).

###### Type material.

***Lectotype* [*muscina*]: Germany.** [not given] (1♀, NMW). [Not examined]

***Holotype* [*marginata*]: USA.** “Loew coll., *marginata* m.” (1♂, MCZ; type no. 13,438). [Not examined]

###### Additional material examined.

363♂ 299♀ (CNC, UDCC, USNM). **New provincial/state records: Canada. NB**: Chamcook, 16.viii.1957, G.E. Shewell, CNC481036 (1♀, CNC), Kouchibouguac N.P., 21.v.1977, B.E. Cooper, Code-5107K, CNC481033 (1♂, CNC), St. Andrews, 8.viii.1957, G.E. Shewell, CNC481035 (1♀, CNC), **SK**: Saskatoon, 9.ix.1959, J.R. Vockeroth, CNC481034 (1♀, CNC). **USA. CT**: Redding, 8.vi.1928, A.L. Melander (1♀, USNM), **DE**: Rehoboth, 18.vii.1976, Malaise trap, W.W. Wirth (3♀, USNM), New Castle Co., Newark, White Clay Creek, 11.vi.2001, sweeping, C.R. Bartlett (1♀, UDCC), **NH**: Mt. Madison, Dolly Copp Cmpgrd., 8–9.vii.1986, J.M. and S.H. Cumming, CNC481037 (1♀, CNC), **VA**: Arlington, vi.1938, J.R. Malloch (2♀, USNM), Falls Church, Holmes Run, 17.vii.1960, light trap, W.W. Wirth (1♀, USNM), 1.vii.1961 (1♀, USNM), 27.vii.1968 (1♂, USNM), Grayson Co., 15 km S Galax at Chestnut Creek, 28.vi.1986, W.E. Steiner (1♂, USNM), Giles Co., Mountain Lake, 7.ix.1976, G.C. Steyskal (2♂, USNM), **WV**: Great Cacapon to Largent, 2–3.vii.1977, Malaise trap, W.W. Wirth (1♀, USNM), Morgan Co., near Great Cacapon, 3.vii.1983, G.F. and J.F. Hevel (1♂, USNM).

###### Comments.

*Cerodonthamuscina* is a relatively abundant species in North America with a characteristic twisted distiphallus. External structure and colouration are also relatively distinct, although it can be easily confused for the slightly paler and much less common *C.kennethi*.

##### Cerodontha (Poemyza) pygmaea

Taxon classificationAnimaliaDipteraPhytomyzinae

(Meigen)

Figs 550–554

Agromyza pygmaea Meigen, 1830: 183.
Dizygomyza pygmaea . Hendel, 1920: 135.
Dizygomyza (Poemyza) pygmella Hendel, 1931: 48. Spencer 1969 [synonymy].
Dizygomyza (Poemyza) verrucosa Hendel, 1931: 51. Nowakowski 1973 [synonymy?].
Dizygomyza (Poemyza) pygmaea . Hendel, 1931–1936: 46.
Phytobia (Poemyza) pygmaea . Hennig, 1953: 138.
Phytobia pygmaea . Kubska, 1961: 34.
Cerodontha (Poemyza) pygmaea . Nowakowski, 1962: 112, 1967: 651, 1973: 127; Spencer 1969: 133; Spencer and Steyskal 1986b: 279; Benavent-Corai et al. 2005: 12; Scheffer and Lonsdale 2018: 87.
Phytobia (Poemyza) verrucosa . Rohdendorf, 1970: 251.

###### Description.

Wing length 2.1–2.5 mm (♂), 2.4–3.2 mm (♀). Length of ultimate section of vein M4 divided by penultimate section: 1.9–2.2 (2.4–3.2 in European specimens). Eye height divided by gena height: 6.0–8.3. Arista short pubescent. First flagellomere ovate, length equal to or slightly greater than height. Ring around eye narrow but strong. Lunule narrow, up to 1/2 length of frons, minutely pitted. Frons slightly wider compared to other *Poemyza*, resulting in fronto-orbital plate being less than 1/4 width of frons. Anterior region of fronto-orbital plate slightly expanded, covering lateral base of lunule; inner margin clearly delimited, but not as strongly sclerotised or raised, as in other *Poemyza*.

***Chaetotaxy***: Two ori; two ors; relatively stout. Ocellar and postocellar setae slightly shorter and thinner than fronto-orbitals. Orbital setulae in single row. One pair of prominent setae apically on each palpus. Four dorsocentral setae, decreasing in length anteriorly with fourth seta 1/3–2/5 length first seta. Acrostichal setulae in six scattered to irregular rows. Acrostichal seta relatively strong to slightly developed, not much larger than surrounding setulae.

***Colouration***: Setae dark brown. Head dark brown, with back of head, ventral margin of gena, face, clypeus and palpus darker; ocellar spot darker, relatively small; centre of frons with dense, deep minute pits; gena often slightly paler; space between clypeus with minute dark brown spicules. Body dark brown, subshiny, with moderate greyish brown pruinosity; postpronotum and notopleuron slightly paler; apex of fore femur and base of fore tibia light yellow for length equal to width of femur apex; apex of mid and hind femora faintly and narrowly yellowish, or entirely dark brown. Wing veins brown, only yellow at very base. Halter light yellow. Calypter margin and hairs dark brown.

***Variation***: Delmarva specimens with highly reduced pruinosity on body, appearing shinier; acrostichal setae absent. English specimens with apex of mid femur nearly as pale as fore femur.

***Genitalia***: (Figs 550–554) Epandrium with supra-anal process; anteroventral margin weakly sclerotised; fused to surstylus. Surstylus small, directed inwards, with short thin setae. Ventral process of subepandrial sclerite with broad, stout base and short apical section with irregular outer margin. Basiphallus ventrally fused to phallophorus; sclerotised along ventral and left lateral surfaces with anterodorsal process extending along right distal surface. Hypophallus broad and flat, becoming thinner and paler apically; M-shaped with slight basomedial carina and other lesser striations. Left paraphallus present, directed laterally, meeting base of mesophallus. Mesophallus stem swollen subbasally in ventral view, much more so in lateral view, and dorsoventally compressed to narrow distal point of fusion with apical chamber; apical chamber with broad, bilobed base that broadly overlaps stem. Distiphallus tubules elongate, question mark-shaped, clear, with narrow sclerotised band on inner surface on basal 1/2, and complete narrow outer band reaching small apical cup. Ejaculatory apodeme with small, narrow blade.

###### Host.

Poaceae – *Agropyron*, *Agrostis*, *Ammophila*, *Apera*, *Arrhenatherum*, *Avena*, *Avenula*, *Brachypodium*, *Bromus*, *Calamagrostis*, *Dactylis*, *Deschampsia*, *Drymochloa*, *Elymus*, *Festuca*, *Glyceria*, *Holcus*, *Hordeum*, *Lolium*, *Melica*, *Milium*, *Molinia*, *Phalaris*, *Phleum*, *Phragmites*, *Poa*, *Secale*, *Setaria*, *Triticum* (Ellis 2021).

###### Distribution.

**Canada**: BC*, NL, ON, QC. **USA**: AK, DE*, MD*, NY, TN*, WV*. Europe. Russia.

###### Type material.

***Holotype* [*pygmaea***]: Germany [not given]. [Lost].

***Holotype* [*verrucosa*]: Finland.** Birkkala (1♂ UZMH). [Not examined].

***Syntypes* [*pygmella*]: Russia.** Far East: Kamtschatka. Ozernaja, coll. Wuorentaus (2♂ 2♀ UZMH).

###### Additional material examined.

**Austria**. Oberösterreich: Linz, 16.vii.1962, Hering, mine an *Dactylisglomerata* Nr. 6804, CNC481163 (1♀, CNC). **Canada. BC**: Terrace, 50 mi SW, around and on wet cliff face, 9.vii.1960, J.G. Chillcott, CNC481213 (1♀, CNC), **NL**: St. John’s, Agric. Exp. Sta., 12.vii.1967, J.F. McAlpine, Malaise trap, CNC481218, CNC481219 (2♀, CNC), 9.viii.1967, CNC481216, CNC481217 (2♀, CNC), St. John’s, Agric. Exp. Sta., on *Ranunculus*, 18.vii.1967, J.F. McAlpine, CNC481214, CNC481215 (2♀, CNC), Stevenville, 19.vi.1979, B.V. Peterson, CNC481220 (1♀, CNC), **ON**: Dundas, 27.v.1955, O. Peck, CNC481209 (1♀, CNC), Normandale, 29.v.1956, J.R. Lonsway, CNC481185 (1♂, CNC), Point Pelee, 7.vii.1980, D.L. Krailo, CNC481210 (1♀, CNC), Pt. Pelee, 7.vii.1980, S. Beierl, CNC481211 (1♀, CNC), Simcoe, 2.vi.1939, G.E. Shewell, CNC481167 (1♂, CNC), **QC**: Fort Chimo, on *Solidago*, 2.vii.1954, J.G. McAlpine, CNC481208 (1♀, CNC). **USA. AK**: Anchorage, 15.vi.1953, R.S. Bigelow, CNC481189, CNC481190 (2♀, CNC), 18.vii.1953, CNC481171 (1♂, CNC), 26.vi.1951, CNC481168 (1♂, CNC), 27.vi.1951, CNC481186, CNC481188 (2♀, CNC), 27.vi.1953, CNC481169 (1♂, CNC), 29.vi.1951, CNC481187 (1♀, CNC), 29.vi.1953, CNC481170 (1♂, CNC), Chowiet Island (Aleutian), 56°2'5.82"N, 156°44'25.14"W, prairie meadow, 10.vii.2009, Goulet and Boudreault, sweeping, CNC340749 (1♂, CNC), Cold Bay, on tundra, 21.viii.1952, W.R. Mason, 163°W, CNC481173 (1♂, CNC), King Salmon, Naknek, 11.vii.1952, W.R. Mason, CNC481198 (1♀, CNC), 6.vii.1952, CNC481184, CNC481199 (1♂ 1♀, CNC), 4.vii.1952, CNC481200 (1♀, CNC), 3.viii.1952, CNC481182, CNC481183 (2♂, CNC), 13.vii.1952, J.B. Hartley, CNC481205 (1♀, CNC), 13.viii.1952, CNC481207 (1♀, CNC), 3.vii.1952, CNC481201–481203 (3♀, CNC), 4.vii.1952, CNC481204 (1♀, CNC), 4.viii.1952, CNC481181 (1♂, CNC), 5.viii.1952, CNC481180 (1♂, CNC), 8.viii.1952, CNC481206 (1♀, CNC), Naknek, on tundra, 2.viii.1952, W.R. Mason, CNC481179 (1♂, CNC), 21.vii.1952, CNC481174 (1♂, CNC), CNC481172–481196 (1♂ 4♀, CNC), 8.viii.1952, CNC481178 (1♂, CNC), 9.viii.1952, CNC481176 (1♂, CNC), 3.vii.1952, J.B. Hartley, CNC481175, CNC481177, CNC481197 (2♂ 1♀, CNC), Unalakleet, 18.vii.1961, R. Madge, CNC481191 (1♀, CNC), 24.vii.1961, CNC481192 (1♀, CNC), **DE**: Rehoboth, 18.vii.1976, Malaise trap, W.W. Wirth (1♀, USNM), **MD**: Colesville, 28.v.1977, W.W. Wirth (1♀, USNM), 4.vi.1977 (1♀, USNM), 14.vi.1977 (1♀, USNM), **TN**: Gr. Sm. Mt. Nat. Park, Indian Gap, 1554 m, 24.vii.1957, J.G. Chillcott, CNC481212 (1♀, CNC), **WV**: Morgan Co., near Great Cacapon, 3.vii.1983, G.F. and G.F. Hevel (1♀, USNM). **England.** Chippenham Fen., Cambs., 5.vii.1958, [K.A. Spencer], Brachypodium Syl. em. 19.vii.1958, CNC481166 (1♀, CNC), Hering, mine an Brachypod. silvaticum em. 27.vii.1958, 6315, CNC481162 (1♂, CNC), Marlow, Buck., 11.vii.1954, [K.A. Spencer], Gramineae [em.] 26.vii.1954, CNC481164 (2♂/♀, CNC), Scratch Wood, Mddx., [K.A. Spencer], em. 20.vii.1956, CNC481165 (1♀, CNC).

##### Cerodontha (Poemyza) pygminoides

Taxon classificationAnimaliaDipteraPhytomyzinae

Spencer

Figs 555–558

Cerodontha (Poemyza) pygminoides Spencer, 1981: 183. Spencer and Steyskal 1986: 279.

###### Description.

Wing length 1.8–2.2 mm (♂). Female unknown. Length of ultimate section of vein M4 divided by penultimate section: 1.5–1.7. Eye height divided by gena height: 7.1–8.9. Arista short pubescent. First flagellomere small, ovate, slightly longer than high. Fronto-orbital plate ¼ frons width. Lunule minutely pitted, appearing as high as wide or slightly higher if lateral base concealed by fronto-orbital plate.

***Chaetotaxy***: Two ori; two ors. Orbital setulae in one row. Ocellar and postocellar setae subequal to fronto-orbitals. Four dorsocentral setae, strongly decreasing in length anteriorly, with fourth seta almost setula-like and sometimes apparently absent. One pair of prominent setae apically on each palpus.

***Colouration***: Setae dark brown. Head mostly brown to dark brown; if head paler, first flagellomere, anterior region of frons, face, back of head, ventral margin of gena, clypeus and palpus always darker, fronto-orbital plate, ocellar triangle, gena and pedicel light brown, and ocellar tubercle brown; if darker than surrounding frons, pigment on ocellar tubercle ill-defined and often faded (not dark and sharply delimited); soft central region of frons heavily pitted with dark brown to orange spots, always dark brown anteriorly, brownish yellow to dark brown posteriorly; pedicel sometimes yellowish apically. Body dark brown with postpronotum and notopleuron slightly paler and apices of femora light yellow for length equal to width of femur apex; wing veins light brown to yellowish, light yellow to base; halter light yellow; calypter white.

***Genitalia***: (Figs 555–558) Epandrium with small supra-anal process; fused to surstylus. Anterior portion of surstylus small, rounded, directed inwards but with longer anteroventral section; with small setae. Ventral process of subepandrial sclerites with arm as long as stout base, smooth, and with shallow outer subapical point. Basiphallus fused to venter of phallophorus; sclerotised along ventral surface of shaft, with broad, bifid apical section flanking base of mesophallus; left distal margin extended as long process. Hypophallus weakly sclerotised along surface of membranous fold and with right lateral carina; ventrally continuous with basiphallus. Paraphallus absent. Mesophallus fused to distiphallus, stem ~ 6 × longer than wide; apical chamber small, bilobate, slightly overlapping stem. Distiphallus tubule lightly sclerotised, gradually widening to broad apical cup with inner marginal row of bumps; with broad, shallow medial curve that ends very close to base.

###### Host.

Unknown.

###### Distribution.

**USA**: CA, CO, MD*, VA*, WV*.

###### Type material.

***Holotype*: USA. CA**: Inyo Co., 2mi N of Cartago, 15.vii.1953, E.I. Schlinger (1♂, UCD). [Not examined]

###### Additional material examined.

**USA. CO**: Boulder, Flagstaff Cn., 1767 m, on side of stream, 10.vi.1961, C.H. Mann, CNC481158 (1♂, CNC), **MD**: Seaside, 23.vi.1931, A.L. Melander (1♂, USNM), **VA**: Chain Bridge, 23.iv.1922, J.R. Malloch (1♂, USNM), **WV**: Bluefield, 17.vi.1970, G. Steyskal (2♂, USNM).

###### Comments.

The distiphallus of the eastern material examined here (Maryland male illustrated) deviates slightly from that of the holotype, being more sinuate with the apex angled ventrally, not with a relatively elongate, straight middle section and a distally pointing apex. The male from Colorado identified by Spencer illustrates an intermediate shape, suggesting that there is a continuum of morphology, although this variation should be re-examined following the collection of more material.

What is most noticeable in the re-evaluation of this species is that the previous key character used for identification in Spencer and Steyskal (1986b) does not work for specimens aside from the holotype. The length of ultimate section of vein M4 was described as being ~ 2 × length of the penultimate section in the holotype, but it is actually 1.73 in the holotype and 1.5–1.6 in the remaining specimens. While smaller than the ratio seen in the similar Cerodontha (Poemyza) attenuata Spencer, it is closer to that seen in C. (P.) inconspicua (California, Colorado, North Carolina, Utah), which mostly differs in being more matt on the thorax and having the dark, triangular spot around the ocelli very dark and clearly defined.

##### Cerodontha (Poemyza) superciliosa

Taxon classificationAnimaliaDipteraPhytomyzinae

(Zetterstedt)

Figs 559–563

Agromyza superciliosa Zetterstedt, 1860: 6455. Frick 1959: 381 [as synonym of Agromyzalateralis Macquart, followed by Spencer (1969)].
Agromyza coquilletti Malloch, 1913: 295. Shewell 1953: 466. Nowakowski 1973 [synonymy].
Phytobia (*Poëmyza*) *lateralis* (Macquart). Misidentification. Frick 1959: 381.
Dizygomyza (*Poëmyza*) lateralis. Hendel 1931: 20.
Phytobia (*Poëmyza*) coquilletti. Frick 1952: 392.
Cerodontha (Poemyza) superciliosa . Nowakowski 1967: 650 [lectotype design.], 1973: 112; Sehgal 1971: 323; Spencer 1971: 319, 1981: 183; Spencer and Steyskal 1986b: 93; Benavent-Corai et al. 2005: 12; Papp and Černý 2016: 254.

###### Description.

Wing length 2.3–2.8 mm (♂), 2.5–3.0 mm (♀). Length of ultimate section of vein M4 divided by penultimate section: 0.9–1.2. Eye height divided by gena height: 4.3–5.0. Lunule higher than wide, smooth with broad, shallow pits, relatively well-sclerotised and sharply bent medially to follow curve of frons; with comparatively smooth, level interface with soft portion of frons.

***Chaetotaxy***: Two ori; two ors; length equal or decreasing anteriorly, or only with posterior seta long; often relatively short and straight, at least for ori, with length not exceeding width of soft portion of frons. One pair of prominent setae apically on each palpus.

***Colouration***: Setae dark brown. Frons light yellow to brownish with minute brown spots at base of fronto-orbitals and setulae; soft central region of frons with rows of dark pits that predominate towards lunule; back of head, first flagellomere, ocellar spot (larger than tubercle), clypeus, ventral margin of gena and palpus dark brown; postocellars on yellowish region; face mottled yellow, light brown and brown; pedicel, scape and ring around eye excluding venter of cheek brown; lunule brownish around base of antennae; lateral region of fronto-orbital plate narrowly brownish to orange with margin along eye sometimes brown, fading inwards to base of fronto-orbitals; central pitted region of gena light brown. Body dark brown with moderate greyish brown pruinosity; postpronotum light yellow with large anteromedial dark brown spot; notopleuron light yellow with small, lateral brown stripe; apex of fore femur light yellow for distance slightly more or less than width of femur apex; base of fore tibia light yellow; dorsal transverse carina on epandrium paler brown or yellowish. Halter light yellow. Calypter white.

***Genitalia***: (Figs 559–563) Epandrium very flattened dorsally, further emphasizing large, unusual supra-anal process, which is thick, flat, shelf-like and nearly as wide as epandrium. Surstylus rounded, small, and with additional broad, flat inwardly (and slightly ventrally) directed lobe; cluster of tubercle-like setae on surstylus proper and inner lobe. Basiphallus ventrally fused to phallophorus; sclerotised along left lateral surface, dividing into one pair of bands on distal 1/2, one of which extends to left distal margin and again bifurcates, and one of which wraps around dorsum to right distal margin. Hypophallus with one pair of long plate-like sclerites that fade distally and are irregularly fused via ridges medially. Paraphalli directed laterally at base, then sharply angled towards venter at midpoint; right sclerite reduced. Mesophallus stem swollen at base (bulge exaggerated in lateral view) and distal 2/3 dorsoventrally compressed; with basal process in front of duct insertion and with small shallow bump ventromedially; apical chamber swollen, bilobed, mostly produced below stem. Distiphallus tubules S-shaped with basal curve smaller and not straightened at origin (apex more strongly curved in figure provided in Nowakowski 1973); basal 1/2 well-sclerotised, distal 1/2 mostly clear with small apical cup and single lateral band sclerotised. Ejaculatory apodeme well-developed with darker base and slightly darker margin; sperm pump with venter sclerotised.

###### Host.

Poaceae – *Agropyron*, *Ammophila*, *Apera*, *Avena*, *Dactylis*, *Echinochloa*, *Elymus*, *Hordeum*, *Phleum*, *Poa*, *Secale*, *Triticum*, *Zea*.

###### Distribution.

**Canada**: AB, BC*, MB, NB, NS*, NT, ON, QC, SK, YT. **USA**: “many of the States in the northern 1/2 of the United States”; published state occurrences include AK, CA, CO, IN, KS, MA, ND, NH, NV, OH, PA, VT. Europe, Russia, China, Japan (Papp and Černý 2016).

###### Type material.

***Lectotype* [*superciliosa*]: Sweden.** Stockholm, Nr. 363/65 (1♂ NRS, coll. Bohemann).

***Holotype* [*coquilletti*]: USA. CO**: Fort Collins, Webster’s No. 6610, C.N. Ainslie (1♀, USNM).

###### Paratypes examined

[***coquilletti*]. IN**: Lafayette, P. Luginbill, issued 2.vi.1912, “PARATYPE No. 15569” (1♂, USNM), **NV**: Lincoln, G.I. Reeves, Webster No. 2952, “PARATYPE No. 15569” (1♀, USNM) [Not mentioned in original description].

###### Additional material examined.

**Canada. AB**: Banff Natl. Park, Eisenhower Junction, 1432 m, 6.vi.1955, J.R. McGillis, CNC481240 (1♂, CNC), Banff, 1357 m, along roadside, 26.v.1960, B.S. Heming, CNC481295 (1♀, CNC), Black Foot Coulee, 9.viii.1940, A.R. Brooks, CNC481278, CNC481288 (2♀, CNC), Black Foot Hills, 9.viii.1940, A.R. Brooks, CNC481226–481233, CNC481279–481287 (8♂ 9♀, CNC), Elkwater L., swept from range grass, 2.vii.1956, O. Peck, CNC481292 (1♀, CNC), Lethbridge, Oldman River, 22.vi.1956, O. Peck, CNC481296 (1♀, CNC), **BC**: Atlin, 24.vi.1955, B.A. Gibbard, CNC481251 (1♂, CNC), Oliver, 18.viii.1953, J.R. McGillis, CNC481250 (1♂, CNC), Osoyoos, 49°1'40.68"N, 119°27'8.10"W, alfalfa, 26.v.2005, Goulet and Boudreault, Site 8, CNC481269 (1♀, CNC), Summit Lake, mi392 Alaska Hwy, 1371 m, 8.vii.1959, R.E. Leech, CNC481297 (1♀, CNC), **MB**: 1 km NW of Winnipegosis, Hwy 20, 51°39'3.96"N, 99°56'42.96"W, prairie habitat, 16.vi.2007, Goulet and Boudreault, sweeping, CNC481270 (1♀, CNC), 5mi N Minnedosa, 8.vii.1958, R.L. Hurley, CNC481293, CNC481294 (2♀, CNC), Aweme, 18.vii.1916, N. Criddle, CNC481225 (1♂, CNC), 4.vii.1917, CNC481289, CNC481290 (2♀, CNC), 3.vii.1922, H.A. Robertson, CNC481291 (1♀, CNC), Shilo, 5mi SW, 13.vii.1958, J.G. Chillcott, CNC481234 (1♂, CNC), floodplain comminuty nr. tamarack bog, open grassy marsh, 2.viii.1958, CNC481235, CNC481274, CNC481276, CNC481277, CNC481275 (1♂ 4♀, CNC), Treesbank, 23.vii.1915, N. Criddle, CNC481236 (1♂, CNC), 6.viii.1915, N. Criddle, Teste Aldrich, CNC481237–481239, CNC481271, CNC481272 (3♂ 2♀, CNC), Whitewater L., 4mi N Whitewater, swept from *Hordeumjubatum*, 14.viii.1958, J.G. Chillcott, CNC481273 (1♀, CNC), **NB**: Richibucto, on tidal flat, 12.vii.1977, J.R. Vockeroth, CNC481267 (1♂, CNC), **NT**: Yellowknife, 64°5'0"S, 111°15'0"W, 5.vi.1953, J.G. Chillcott, CNC_Diptera59172, CNC481252 (2♂, CNC), **NS**: S. Harbor, 11.vii.1984, H.J. Teskey, PG961944, CNC481268 (1♂, CNC), **ON**: Atikokan, 4mi E of Hwy. 11, 5.vii.1978, H.J. Teskey, CNC481257 (1♂, CNC), Grand Bend, 19.vii.1939, G.E. Shewell, CNC481259 (1♂, CNC), One Sided Lake, 29.vi.1960, Kelton and Whitney, CNC481310 (1♀, CNC), Ottawa, 24.v.1946, A. Brooks, CNC481311 (1♀, CNC), Penetang, 1.viii.1955, J.G. Chillcott, CNC481258 (1♂, CNC), Simcoe, 15.vi.1939, G.E. Shewell, CNC481260–481263 (4♂, CNC), Stratford, 5.vii.1916, CNC481256 (1♂, CNC), Waubamick, vi.1915, H.S. Parish (1♂, USNM), **QC**: Breckenridge, 28.vi.1959, C.H. Mann, CNC481266 (1♂, CNC), Ile de Montreal, 5.viii.1906, Beaulieu, CNC481264, CNC481313 (1♂ 1♀, CNC), Mi. 139 Rte. 58, La Verendrye Prov. Pk., 28.vi.1965, D.M. Wood, CNC481312 (1♀, CNC), Rupert House, 29.vii.1949, E.J. LeRoux, CNC481265 (1♂, CNC), **SK**:11 km S Empress, AB, riverine cottonwood, maple, 12–19.viii.1986, J. Acorn, Malaise trap, CNC481298, CNC481299 (2♀, CNC), Buchanan, 6.viii.1925, K.M. King, CNC481303 (1♀, CNC), Great Deer, 8.ix.1950, J.R. Vockeroth, CNC481300 (1♀, CNC), Indian Head, 6.ix.1920, S. Stewart, CNC481242 (1♂, CNC), Pheasant City, 13.vii.1937, A.R. Brooks, CNC481241 (1♂, CNC), Saskatoon, 16.viii.1939, A.R. Brooks, CNC481243, CNC481302 (1♂ 1♀, CNC), 6.viii.1939, CNC481301 (1♀, CNC), Saskatoon, 22.vii.1933, K.M. King, 16447-1129B, CNC481305 (1♀, CNC), 22.viii.1927, 16446-126BG, CNC481248 (1♂, CNC), 25.viii.1923, 16410-3N9B, CNC481244, CNC481245 (2♂, CNC), 26.vi.1930, 16424-330B, D360, CNC481246 (1♂, CNC), 27.vii.1923, 16410-4N10B, CNC481304 (1♀, CNC), 4.vii.1930, 16448-1227AN, CNC481306 (1♀, CNC), 6.viii.1931, 164464-832B, CNC481247 (1♂, CNC), 6.viii.1935, 164464-832B, CNC481249 (1♂, CNC), **YT**: Kusawa Lake Road, 60°42'12.66"N, 136°4'18.72"W, 699 m, 6.vii.2006, Goulet and Boudreault, sweeping, CNC481221–481224 (4♂, CNC), Rampart House, 67°25'26.56"N, 140°59'0.68"W, 2.vi.1951, C.C. Loan, CNC_Diptera59173 (1♀, CNC), Whitehorse, 23.viii.1959, R. Madge, CNC481253 (1♂, CNC). **Denmark.** Gudhjem, Bornholm, F. Budman, mine an *Ammophilaarenaria* coll. Hypon., M. Hering, 3329, CNC481315 (1♂, CNC), Maribo, Bota[?], Sönderup, 3.viii.1932, mine an *Psammaarenaria* coll. Hypon., M. Hering, CNC481314 (1♀, CNC). **USA. AK**: Fairbanks, 13.vi.1952, J.B. Hartley, CNC481307 (1♀, CNC), **CO**: Boulder Co., Corona Pass, 3230 m, 6.vii.1961, J.G. Chillcott, CNC481309 (1♀, CNC), Mt. Evans, 3566 m, timberline, 22.vii.1961, W.R.M. Mason, CNC481254 (1♂, CNC), Niwot Ridge, nr. Ward, 3505 m, on tundra, 4.vii.1961, C.H. Mann, CNC481308 (1♀, CNC), C.N. Ainslie, reared from *Agromyza* in *Agrobyron* Webster No. 13784, Wimbeldon NBJ16915, CNC481255 (1♂, CNC), Crook, 15.vi.1940, A.L. Melander (1♂, USNM), **NH**: Mt. Wash., Alpine Garden, 2.vii.1956, 5900', A.L. Melander (1♂, USNM).

###### Comments.

While Cerodontha (Poemyza) superciliosa has not been confirmed in the Delmarva states, Frick (1959) noted that he had seen specimens from “many of the States in the northern 1/2 of the United States”, and it is thus included here. Characteristic of this species are light yellow shoulders and a light yellow, smooth (shallowly and gently textured) and medially angulate lunule that meets a similarly yellow frons that is darker, pitted, and softer anteriorly. The large, wide, and flat supra-anal process of the male is also highly characteristic.

##### Cerodontha (Poemyza) ungula sp. nov.

Taxon classificationAnimaliaDipteraPhytomyzinae

http://zoobank.org/79B1D520-22A6-4500-85EB-35C70734353F

Figs 564–566

###### Description.

Wing length 2.1 mm (♂), 2.3–2.4 mm (♀). Length of ultimate section of vein M4 divided by penultimate section: 1.1–1.6. Eye height divided by gena height: 3.0–4.1. First flagellomere slightly longer than high, dorsal margin with very shallow angle between apex and middle. Arista short pubescent. Lunule angled posteriorly near base, visible portion (lateral base concealed by fronto-orbital plate) not much higher than wide, small, separated from ocellar triangle by its own height. Fronto-orbital plate and parafacial prominent, appearing thick when viewed laterally, continuing under eye as strong cheek; fronto-orbital plate, parafacial and anterior region of frons slightly bulging outwards so that anterior surface of head not appearing flat when viewed from above; fronto-orbital plate well-sclerotised, appearing continuous with ocellar triangle. Gena comparatively broad and rounded, not as projecting posteriorly.

***Chaetotaxy***: Two ori (sometimes three ori on one side); two ors; slightly decreasing in length anteriorly. Ocellar and postocellar setae subequal to fronto-orbitals. One row of strong orbital setulae. One pair of prominent setae apically on each palpus. Four dorsocentral setae, strongly decreasing in length anteriorly, with fourth seta barely larger than surrounding setulae. Four rows of acrostichal setulae. Acrostical seta absent; one female with duplicated dorsocentral in one acrostichal row.

***Colouration***: Setae dark brown with paler brown shine. Head dark brown; slightly darker or paler centrally on frons, which is covered with blackish pits; lunule and gena slightly paler; ocellar spot, back of head, clypeus, face, and venter of gena darker; ocellar triangle yellowish around dark ocellar spot; buccal cavity within arms of clypeus with dense orange to brown spicules. Remainder of body dark brown with notopleuron slightly paler and fore femur yellow for length less than apical width of femur; mid femur sometimes with slight yellowish tint apically. Wing veins light brown, becoming yellower towards base and often whitish posteriorly. Halter light yellow. Calypter margin and hairs light brown.

***Genitalia***: (Figs 564–566) Epandrium without supra-anal process. Surstylus small, rounded, directed inwards, with small setae. Ventral process of subepandrial sclerites with arm length equal to base width; smooth, with very small subapical point on outer surface. Basiphallus fused to ventroapical margin of phallophorus; sclerotised along left lateral and ventral surfaces, with nearly separate dorsal to right dorsolateral plate on distal 2/3. Hypophallus with one pair of flat, apically narrowing and desclerotised plates. Only left paraphallus evident as clear lobe. Mesophallus stem slightly wider at base, width ~ ¼ length; apical chamber small with simple rounded base, wider than long. Distiphallus tubule slightly longer than base + mesophallus, relatively straight with slight basal curve, lightly sclerotised, narrow with apex widened into darker, textured cup. Ejaculatory apodeme as long as basiphallus+ phallophorus.

*Variation*: Delaware female with first flagellomere darker and as long as high; paler brown or yellowish pigment absent from head, which is entirely dark brown; fourth dorsocentral more closely set to third dorsocentral.

###### Host.

Unknown, but the label of one paratype mentions “wheat”, which may represent a rearing record.

###### Distribution.

**USA**: DE, MI.

###### Etymology.

The specific name is Latin for “finger ring”, referring to the pronounced ring around the eye.

###### Type material.

***Holotype*: USA. MI**: Mio, 29.v.1937, H. Milliron (1♂, USNM).

***Paratypes*: USA. DE**: Middleton, 17.v.1960, wheat (1♀, UDCC), **MI**: Mio, 29.v.1937, H. Milliron (2♀, USNM), E. Lansing, 29.v.1937, C. Sabrosky (1♀, USNM), Lapeer Co., 30–31.v.1937, C. Sabrosky (1♀, USNM).

###### Comments.

The new species Cerodontha (Poemyza) ungula is unique among the Nearctic *Poemyza* is its dark colouration, although the calypter is light brown; there are only four rows of acrostichal setulae, there is a slight point on the first flagellomere, and the fronto-orbital plate, parafacial and cheek are strong and projecting. The latter character is especially distinct but also seen in *C.chillcottiella* Spencer (Colorado), although in this western species the first flagellomere is more conspicuously pointed and the notopleuron and the centre of the frons are yellow. The genitalia are similar to those of *C.pygminoides*, although the surstylus of the new species is small and simple, the phallus is slightly stouter and shorter, the lateral and dorsal plates of the basiphallus slightly vary in shape and the left lateral margin is not produced, the apical chamber of the mesophallus is more rounded, the distiphallus is less broadly arched medially and with a more pronounced basal curve, and the apical cup on the tubule is larger and darker.

##### Cerodontha (Xenophytomyza)

Taxon classificationAnimaliaDipteraPhytomyzinae

Frey

Cerodontha (Xenophytomyza) Frey, 1946: 51. Type species: HaplomyzaatronitensHendel 1920, by original designation. Frick 1959: 396; Nowakowski 1967: 655; Spencer and Steyskal 1986b: 91; Boucher 2003: 2.
Xenophytomyza . Hering 1955: 170.

*Xenophytomyza* is primarily north temperate in distribution, with seven exclusively Palaearctic species (mostly Russian), one Holarctic and Jamaican species, and one eastern Nearctic species. The three species with known hosts have all been reared from Poaceae (Boucher 2003).

The single Delmarva species is distinct among other local Agromyzidae in having three dorsocentrals that strongly decrease in length anteriorly, and there is a mix of characters seen in other *Cerodontha* subgenera: the lateral scutellar seta is absent (also seen in *Cerodontha* s. s.), the first flagellomere has a strong anterodorsal angle (but not produced to a point as seen in *Cerodontha* s. s.), the lunule is narrow and slightly higher than wide (similar to some *Poemyza*) and the body is darkly pigmented. Support for the subgenus is weak and its relationships to other subgenera require investigation (Boucher 2003).

#### Species description

##### Cerodontha (Xenophytomyza) illinoensis

Taxon classificationAnimaliaDipteraPhytomyzinae

(Malloch)

Figs 104 , 567–573

Agromyza illinoensis Malloch, 1934: 483. Frick 1952a: 374.
Cerodontha (Xenophytomyza) illinoensis . Frick 1952c: 151; Spencer and Steyskal 1986b: 92; Boucher 2003: 6.
Phytobia (Xenophytomyza) illinoensis . Frick 1959: 397.
Cerodontha (Poemyza) simcoensis Spencer, 1969: 135. Spencer and Steyskal 1986b [synonymy].

###### Description

**(Fig. 104).** Wing length 2.0–2.2 mm (♂), 2.2–2.4 mm (♀). Length of ultimate section of vein M4 divided by penultimate section: 1.5–2.0. Eye height divided by gena height: 9.0–10.0. First flagellomere slightly longer than high, anterodorsal margin forming strong angle. Arista pubescent. Frons slightly narrowing anteriorly, very soft, not smoothly meeting lunule. Fronto-orbital plate 1/4 width of frons, slightly visible laterally; ocellar triangle large, approximately equilateral with sides rounded; parafacial very narrow, continuing as narrow “cheek”; gena angled upwards. Gena and frons with minute scattered pits that are much denser and darker on frons; pits not present on fronto-orbital plate, reduced on ocellar triangle, becoming absent medially on tubercle.

***Chaetotaxy***: Two to three ori; two ors; decreasing in length anteriorly with anterior seta as small as 2/3 length of posterior ors. Orbital setulae relatively long, scattered, in one or two rows on either side of fronto-orbitals. Ocellar and postocellar setae subequal to ors; ocellar setae slightly displaced laterally to level of outer ocelli. Four dorsocentral setae strongly decreasing in length anteriorly, with fourth seta often indistinguishable from surrounding setulae (not visible in material examined), and third at most 1/3 length of first seta. Acrostichal seta absent. Approximately eight scattered rows of acrostichal setulae reaching level of posterior dorsocentral. Lateral scutellar seta absent.

***Colouration***: Setae dark brown. Body dark brown; fronto-orbital plate and region around margin of ocellar triangle paler; antenna paler with first flagellomere darkest of segments; apex of fore femur yellow for length less than width of femur. Calypter margin and hairs brown. Halter yellow. Wing veins brown, only yellow at very base.

***Genitalia***: (Figs 567–573) Epandrium without process above anus; fused to surstylus with suture absent. Surstylus broad, short, slightly produced past margin of epandrium, directed inwards, margin setose. Subepandrial sclerite with ventral lobe narrow, dark, apically tapering and with slight outer-distal point. Basiphallus apically split. Hypophallus flat, long, faded and narrowed apically. Paraphallus absent. Mesophallus fused to distiphallus, forming long, clear, narrow tubule that is longer than remainder of phallus; slightly swollen basally and appearing partially split apically. Ejaculatory apodeme with small, rounded, mostly clear blade, and dark, narrow stem with minute clear subbasal process; sperm pump clear.

###### Host.

Unknown, likely Poaceae (Boucher 2003).

###### Distribution.

**Canada**: ON. **USA**: IL, MS*, TN, VA, VT*, WV*.

###### Type material.

***Syntypes* [*illinoensis*]: USA. IL**: White Heath, 22.v.1915 (3♀), 8.v.1915 (1♀ 1♂), 9.v.1915 (2♂), 16.v.1915 (2♀), 30.v.1915 (2♀). [Types in INHS, USNM]. [Not examined]

***Holotype* [*simcoensis*]: Canada. ON**: Simcoe, 9.vi.1939, G.E. Shewell, CNC481017 (1♂, CNC).

***Paratypes* [*simcoensis*]: Canada. ON**: Simcoe, 9.vi.1939, G.E. Shewell, CNC481018–481020 (2♂ 1♀, CNC).

###### Additional material examined.

**Canada. ON**: Ottawa, Stony Swamp, Beaver Trail, 45°18'0"N, 75°49'16"W, 13.vi.2015, O. Lonsdale, CNC441042, CNC441054, CNC441059, CNC441073, CNC441084 (5♂, CNC), Ottawa, 17.vi.1954, C.D. Miller, CNC481023 (1♀, CNC). **USA. MS**: Winston Co., Noxubee NWR, Loakfoma Crk., at Dummy Line Rd., 33°15'9"N, 88°50'53.01"W, 17–21.v.2013, J.M. Cumming, Malaise trap, CNC481024 (1♀, CNC), **TN**: East Ridge, 4.v.1952, O. Peck, CNC481021 (1♂, CNC), 6.v.1952, CNC481022 (1♀, CNC), **VA**: Fairfax Co., Dead Run, 19.vi.1915, R.C. Shannon (1♂, USNM), **VT**: Rutland Co., nr. Benson, hwy 22A, 1mi N hwy 144, 22.vi.1997, sweeping, C.R. Bartlett (1♀, UDCC), **WV**: Grafton, 25.vi.1970, G. Steyskal (1♂, USNM), Summers Co., Pipestem, 18.vi.1970, G. Steyskal (1♂, USNM).

##### Haplopeodes

Taxon classificationAnimaliaDipteraPhytomyzinae

Steyskal

Haplopeodes Steyskal, 1980: 141. Type species: PhytomyzaminutaFrost 1924: 86, by original designation. Spencer and Steyskal 1986b: 138.
Haplomyza . Lapsus. Spencer 1987: 878.

When *Antineuratogata* Melander, the type species for *Haplomyza*, was moved to *Liriomyza*, the remaining species placed in *Haplomyza* were left orphaned. The name *Haplopeodes* was provided by Steyskal (1980) to encompass these orphaned taxa, which were found to represent a natural group. The genus occurs throughout the New World on Amaranthaceae, Chenopodiaceae, Portulacaceae and Solanaceae, producing short, irregular, and somewhat straight mines (Steyskal 1980; Spencer and Steyskal 1986b). One species occurs in Africa (Spencer 1961), but this was likely misplaced to genus. Steyskal (1980) last revised the genus, and Spencer and Steyskal (1986b) last treated the fauna of the United States. A single new species was described from California in Eiseman et al. (2021).

Morphologically, *Haplopeodes* species are very similar to *Liriomyza*, but they exhibit a strong reduction or simplification of both external and genitalic features, likely due in no small part to their minute body size. There is no stridulating mechanism, only one ors, dm-m is lost, the phallus is simplified and largely membranous, and the ejaculatory apodeme is narrow with little pigment on the sperm pump. Similar to many *Liriomyza*, the posterodistal margin of the epandrium has a single tubercle-like seta and the surstylus is small with few apical tubercles, but the ejaculatory duct is not pigmented and does not appear to be much swollen apically.

#### Species description

##### Haplopeodes minutus

Taxon classificationAnimaliaDipteraPhytomyzinae

(Frost)

Figs 120 , 574–581

Phytomyza minuta Frost, 1924: 86. Frick 1952a: 427.
Haplomyza minuta . Frick, 1953: 73 (lectotype designation); 1959: 413.
Haplopeodes minutus . Steyskal, 1980: 148; Spencer and Steyskal 1986b: 140; Martinez and Etienne 2002: 32.
Haplomyza togata . Misidentification. Stegmaier, 1967: 197; Spencer 1969: 201, 1981: 338.
Haplomyza minuta . Spencer and Stegmaier 1973: 112.

###### Description

**(Figs 120, 581).** Wing length 1.3–1.4 mm (♂), 1.5–1.6 mm (♀). Vein dm-m absent. Eye height divided by gena height: 2.6–3.3. First flagellomere sometimes with shallow anterodorsal angle. Frons soft medially. Fronto-orbital plate and parafacial somewhat visible laterally. Clypeus narrow along length. Lunule semi-circular.

***Chaetotaxy***: Three ori (slender with length ~ 1/2 width of frons); one ors. Postocellar and ocellar setae as long as ocellar tubercle. Orbital setulae few, minute, erect to proclinate, in a single row. Four dorsocentrals, one presutural, decreasing in length anteriorly. Acrostichal setulae in two sparse rows on anterior 1/2 of scutum.

***Colouration***: Head light yellow with ocellar tubercle, back of head (often excluding margins) and clypeus brown; vertical setae on yellow. Notum dark with pale grey pruinosity, with notopleuron (excluding dark sublateral spot) and postpronotum (excluding small anteromedial spot) light yellow, and scutellum broadly light yellow along midline. Metanotum brown. Pleuron yellow with dark spot on anterior margins of anepisternum and anepimeron, meron dark medially and posteriorly, and ventral 4/5 of katepisternum brown, not including base of seta; dark portions obscured by grey pruinosity. Halter white. Calypter white. Legs light yellow with base of mid and hind coxae brown, and tarsi brownish; fore coxa sometimes brown basally. Abdomen brown on tergites 1(2)-4 with lateral margin yellow and posterior margin of latter three tergites yellow; tergites 5 and 6 yellow with one pair of confluent anterior spots (very small on tergite 6). Female as described for male except as follows: abdomen brown with oviscape dark and posterior margin of tergites 2–5 yellow (medially emarginate on tergite 5), and tergite 6 yellow with one pair of confluent anterior spots.

***Variation***: BC male as above except outer vertical seta sometimes near dark spot emerging from back of head; first flagellomere with anterior margin brown infuscated, pigment extending to outer 1/2; fore femur sometimes with dorsal streak. One female (CA) with distal 1/2 of first flagellomere brown.

***Genitalia***: (Figs 574–580) Epandrium broad and shallow, with width 3 × length; posterodistal margin with one tubercle-like seta. Surstylus small and rounded with three apical tubercle-like setae. Hypandrium thin with bare, ovate inner lobe; lightly sclerotised membrane uniting both sides along length. Postgonite with ventral extension and tapered apex. Ejaculatory apodeme finger-like, only slightly widened at base, and sperm pump only slightly sclerotised to one side. Phallus short, clear and highly reduced; boundary between basiphallus and distiphallus indistinct; ejaculatory duct only gradually widened at apex, where it emerges from surrounding basiphallus/distiphallus. Phallophorus narrowest at midpoint.

###### Hosts.

Amaranthaceae – *Amaranthus* spp., *Chenopodium* spp. (Spencer and Steyskal 1986b).

###### Distribution.

**Canada**: BC*, SK. **USA**: CA, FL, KS, ND, NM, TX, UT*, VA*, WA. Cuba. Guadeloupe(?).

###### Type material.

***Lectotype*: USA. ND**: Fargo, 13.vi.1918 (1♀, USNM).

###### Additional material examined.

**Canada. BC**: Kinbasket Lake, Cooper Beauchesne and Assoc. Ltd., 21.vi.2010, BC Hydro drawdown study (12MTRT01) CNC391566 (1♂, CNC). **USA. CA**: Bakersfield, 9.viii.1948, ex. *Amaranthus* sp., W.H. Lange (1♀, USNM), Imp. Co., Meloland, 27.iv.1977, *Chenopodium*, K.A. Spencer (1♀, USNM), Orange Co., Irving, 24.iv.1977, *Chenopodiummurale*, K.A. Spencer (1♀, USNM), Riverside Co., Corona, 19.x.1958, P. Rude (1♀, USNM), **KS**: Manhattan, 26.viii.1949, C. Stegmaier, No. 146 50-228, ex. *Amaranthusretroflexus* (1♂, USNM), **ND**: Bismarck, 14.vi.1918 (1♂, USNM), **NM**: Las Cruces, J.M. Aldrich, 16.vi.1917 (1♂, USNM), 14.vi.1917 (1♀, USNM), Las Lunas, 17.vii.1963, I.G. Watts, prairie sandroad (1♂, USNM), **TX**: Marfa, 13.vi.1917, J.M. Aldrich (1♀, USNM), Crystal City, reared from pigweed, J.A. Harding, 16.iv.1962 (1♂, USNM), 21.vi.1962 (1♀, USNM), 15.viii.1962 (1♀ 1?, USNM), 14.v.1962 (2♀, USNM), **UT**: Corinne, on celery, 9/7/49, G.F. Knowlton (1♀, USNM), Farmington, on celery, 31.viii.1949, G.F. Knowlton (1♀, USNM), **VA**: Northhampton Co., Kiptopeke, 2–5.x.1987, on flowers of *Solidagosepmervirens*, W.E. Steiner, J.M. Swearingen, J.M. Hill and J.J. Marshall (1♂, USNM).

###### Comments.

Otherwise known from British Columbia, Saskatchewan and the western and southern United States, the Virginia male of *Haplopeodesminutus* listed here represents a significant range extension, both for the species and for the genus. The Virginia specimen largely agrees with the western material except the inner lobe of the hypandrium is of a slightly different shape.

This species can be easily confused for *Liriomyzatogata* (Lonsdale 2011: figs 225–228) in the west, which is found on *Baccharisdouglasii* and *Artemisiadouglasiana* (Spencer 1990). Like *Haplopeodes* species, this *Liriomyza* is sometimes also missing the posterior cross-vein, has a single ors and similar colouration, but the male genitalia are entirely dissimilar. As such, males should be dissected for confident identification.

##### Liriomyza

Taxon classificationAnimaliaDipteraPhytomyzinae

Mik

Agrophila Lioy, 1864. Type species Agromyzastrigata Meigen, 1830 (as AgromyzaexilisMeigen 1830), by subsequent designation (Coquillett 1910). Preoccupied by Boisduval 1840 [Noctuidae]. Frick 1952a [synonymy].
Liriomyza Mik, 1894: 289. Type species: Liriomyzaurophorina Mik, 1894, by monotypy. Spencer and Steyskal 1986: 107; Zlobin 1996: 277, 1999: 129; Lonsdale 2011: 18 [California], 2017: 17 [Canada].
Antineura Melander, 1913: 249. Type species: Antineuratogata Melander, 1913: 249, by original designation. Preoccupied by Osten Sacken (1881) [Platystomatidae].
Haplomyza Hendel, 1914: 73. Type species: Antineuratogata Melander, 1913: 250, by automatic designation. Replacement name for Antineura. Steyskal, 1980 [synonymy].
Praspedomyza Hendel, 1931: 77. Type species: Dizygomyzaapproximata Hendel, 1920: 135, by original designation. Nowakowski 1962 [synonymy].
Craspedomyza . Misspelling. Enderlein, 1936: 181.
Triticomyza Blanchard, 1938: 358. Type species: *cruciata* Blanchard, 1938, by original designation. Frick 1952a: 397 [as syn. Cerodontha]; Spencer 1963: 331 [as synonym of Cerodontha], 1982: 25 [as syn. Liriomyza].
Galiomyza Spencer, 1981: 288. Type species: Agromyzamorio Brischke, 1881, by original designation. Spencer and Steyskal 1986b: 136; Papp and Černý 2017: 28. Lonsdale 2017 [synonymy].

*Liriomyza* is a widespread and diverse genus encountered with relative frequency. Most species can be readily diagnosed by a dark brown notum with yellow shoulders and a sharply defined yellow medial stripe on the scutellum, and sometimes a stridulatory file anterolaterally in the male abdominal membrane (Fig. 107). Much emphasis was previously placed on the presence of this stridulatory organ in the definition of *Liriomyza* (von Tschirnhaus 1971), but its presence is inconsistent across the genus, difficult to observe when present, and its real phylogenetic importance is yet to be fully appreciated. Definition of the genus is unfortunately somewhat nuanced at the moment. As discussed in Lonsdale (2011, 2017) and Zlobin (1996), species of many other genera superficially resemble *Liriomyza*, or are indistinguishable from *Liriomyza* species externally, and a number of *Liriomyza* depart from the “typical” colour form described above. These species are either predominantly dark on the thorax and sometimes the head, such as those species previously treated as *Galiomyza*, or they have reduced chaetotaxy and venation and/or pale grey pruinosity on the thorax, resembling *Haplopeodes*, or they are predominantly yellow, as in some *Phytoliriomyza*. The genus is presently defined solely by a male genitalic character that is consistently present: an ejaculatory duct that is both swollen and pigmented apically. A single genitalic character is clearly not ideal in the definition of a genus, and it is hoped that ongoing phylogenetic work can provide additional characters by clarifying *Liriomyza*’s position with respect to related groups, ideally characters that are visible externally in both sexes.

*Liriomyza* contains many of the most pestiferous species of Agromyzidae, the Nearctic species of which were discussed by Lonsdale (2011), who provided a list of host genera. Of these pests, only *L.brassicae* (Riley), *L.sativae* Blanchard and *L.trifolii* (Burgess) occur in the eastern United States.

In addition to the species redescribed below, Spencer and Steyskal (1986) listed *Liriomyzaendiviae* Hering as occurring in Maryland and Washington State. The Maryland record is represented only by observations of empty leaf mines in *Lactuca*, and since species identity cannot be verified and may have been in error, *L.endiviae* is here not considered to occur in the eastern United States. All other Nearctic specimens previously identified as *L.endiviae* were examined by Lonsdale (2017) and have been determined to belong to another species, most likely *L.taraxaci*.

### ﻿Key to the Delmarva *Liriomyza*

**Table:** 

1	Frons and notum entirely brown. Surstylus with three anterior tubercle-like setae (Fig. 609)	***L.galiivora* (Spencer)**
–	Frons predominantly yellow (Figs 108–115). Notum with most of notopleuron, postpronotum and scutellum yellow (scutellum mostly or entirely brown in some *L.violivora*). Surstylus usually only with one or two apical tubercle-like setae (*L.quadrivittata* with numerous minute setae), but not as above	2
2	Scutum yellow in front of scutellum, either broadly (Fig. 108) or narrowly	3
–	Scutum dark in front of scutellum, with yellow markings restricted to lateral and sometimes posterolateral margins	5
3	Eye sparsely short-haired. Posteromedial setulae on scutum inclinate. Mid tibia with two small posteromedial setae. Third dorsocentral (of four) reduced. Surstylus with dark, elongate, apical process (Fig. 584). Epandrium yellow; posterodistal margin with dark bar on inner face. Apex of phallus bifid with tubules spinulose on inner surface (Figs 586, 587)	***L.blechi* Spencer**
–	Eye bare. Posteromedial setulae on scutum reclinate. Mid tibia without posteromedial setae. Four well-developed dorsocentrals, decreasing in height anteriorly. Surstylus with or without spine-like setae. Epandrium brown; posterodistal margin produced into small dark spine. Apex of phallus cup-like	4
4	Anepisternum with small brown spot. Clypeus brownish. Ocellar tubercle brown. Abdomen brown when viewed dorsally. Wing length 1.6–1.7 mm. Height of eye divided by height of gena 4.3. Length of ultimate section of vein M4 divided by penultimate 2.1–4.0. Surstylus tapering apically and with two apical spines (Fig. 598)	***L.eupatoriella* Spencer**
–	Anepisternum yellow. Clypeus yellow. Ocellar tubercle yellow with brown lines radiating from inner margins of ocelli. Abdomen yellow with ill-defined brownish medial stripe dorsally. Wing length 1.8–2.3 mm. Height of eye divided by height of gena 4.6–4.8. Length of ultimate section of vein M4 divided by penultimate 2.0–2.3. Surstylus with curled distal process and no spines (Figs 612, 613)	***L.philadelphivora* Spencer**
5	Parafacial strongly projecting anterior to eye when viewed laterally. At least three ori on each side of frons. One ors	***L.deceptiva* (Malloch)**
–	Parafacial only barely extending past eye when viewed laterally. Two ori, sometimes with third present on one side. Two or (less commonly) one ors	6
6	Both vertical setae surrounded by yellow. (Fig. 111) Two to four (some *L.trifolii*) rows of acrostichal setulae. Scutum with light dusting of greyish pruinosity	7
–	Outer vertical setae surrounded by dark brown, inner vertical either enclosed or bordering on brown (Fig. 110); some *L.brassicae* with only vestiges of pigment around base of vertical setae. Always four rows of acrostichal setulae. Scutum shiny to subshiny	8
7	Femora sometimes brown at base on mid and hind legs, and fore femur sometimes also with extensive dorsal mottling. Spot on anepisternum long and rectangular, ~ 1/2 length of sclerite, but sometimes larger (Fig. 112). Posterior margin of tergites (1)2–6 entirely yellow. Acrostichal setulae usually in four rows (sometimes two or three). Surstylus tapering and somewhat truncated apically, with one strong distal spine (Fig. 637). Distiphallus narrow and weakly sclerotised (Figs 638, 639)	***L.trifolii* (Burgess)**
–	Femora entirely yellow. Spot on anepisternum small to absent. Posteromedial margin of tergites brown. Acrostichal setulae in two or (less commonly) three rows. Surstylus broadly lobate and with one strong apical and one weak subapical spine (Fig. 604). Distiphallus large, globular and minutely tuberculate (Figs 606, 607)	***L.fricki* Spencer**
8	Palpus and face brown. Scutellum usually mostly to entirely brown with medial yellow stripe atrophied. Surstylus without spines (Fig. 645–648). Paraphallus widened apically, recurved to fuse with mesophallus medially (Figs 650, 651)	***L.violivora* (Spencer)**
–	Palpus and face yellow. Scutellum with distinct medial yellow stripe. Surstylus with one or two spines. Paraphallus various, but not as above. Only males can be identified further	9
9	Surstylus with two spines. Distiphallus sometimes pale and cup-like, but usually either entirely split medially or very dark and subcylindrical	**10**
–	Surstylus with one spine. Distiphallus always pale and cup-like	**12**
10	Anepisternum yellow on dorsal quarter or less. Paraphallus triangular, conspicuous and held out wing-like laterally. Distiphallus composed of two short, separate tubules (Figs 634, 635)	***L.trifoliearum* Spencer**
–	Anepisternum yellow on dorsal 1/2. Paraphallus pale, narrow. Distiphallus undivided	**11**
11	Wing length 1.2–1.8 mm. Lateral margin of frons sometimes with narrow brown margin. Distiphallus very dark and cylindrical with slight medial constriction (Figs 593, 594)	***L.brassicae* (Riley)**
–	Wing length 2.1–2.7 mm. Fronto-orbital plate entirely yellow. Distiphallus pale, cup-like (Figs 628–631)	***L.temperata* Spencer**
12	Mesophallus fused to distiphallus, which is narrow with wider apical chamber (Figs 642–644). Paraphallus subrectangular and usually much broader apically	***L.helianthi* Spencer (in part)**
–	Mesophallus separate from broader distiphallus. Paraphallus not wider apically	**13**
13	Wing length 1.7–2.2 mm. Eye height divided by gena height 3.0–4.4. Distiphallus relatively large and broad (seen ventrally), with base wide and truncated (Figs 601, 602)	***L.eupatorii* (Kaltenbach)**
–	Wing length 1.3–1.8 mm. Eye height divided by gena height 4.4–5.8 (Fig. 109). Distiphallus smaller, base slender and rounded; apical, basal, and ventral surfaces better-sclerotised, forming weak C-shape in profile (Figs 624, 625)	***L.sativae* Blanchard**

#### Species descriptions

Unless otherwise specified, all *Liriomyza* species described below are characterised as follows: orbital plate and parafacial not projecting; first flagellomere small and ovate with short pubescence (ie. not long-haired); epistoma very narrow to absent; setae and setulae black; postpronotum yellow with small dark anteromedial spot, notopleuron yellow with dark narrow sublateral stripe and scutellum yellow with lateral corner brown; four dorsocentrals, decreasing in length anteriorly, anterior seta presutural; acrostichal setulae entirely reclinate; halter white; tibiae without posteromedial setae; surstylus small, directed inwards; epandrium with small anteroventral spine on inner margin; basiphallus sclerotised along dorsal and left lateral surfaces, and with small membranous to lightly sclerotised lobe on left apical margin; apical section of ejaculatory duct swollen, elongate and pigmented; ejaculatory apodeme with transverse sclerite on sperm pump.

Descriptions of species occurring in Canada are redescribed using text from Lonsdale (2017).

##### Liriomyza blechi

Taxon classificationAnimaliaDipteraPhytomyzinae

Spencer

Figs 582–587

Liriomyza blechi Spencer in Spencer and Stegmaier,1973: 98. Spencer and Steyskal 1986b: 285; Lonsdale 2017: 35; Eiseman and Lonsdale 2018: 47; Monteiro et al. 2019: 170; Eiseman et al. 2021: 24.

###### Description.

Wing length 2.0–2.1 mm (♂), 1.9–2.3 mm (♀). Length of ultimate section of vein M4 divided by penultimate section: 1.3–1.4. Eye height divided by gena height: 5.3–7.0. Eye with small, scattered hairs.

***Chaetotaxy***: Two ori (anterior seta small); two ors. Acrostichal setulae in four rows; posteromedial setulae inclinate. Third dorsocentral from back reduced to absent. Mid tibia with two small posteromedial setae.

***Colouration***: Head yellow with ocellar tubercle, back of head above foramen and posterior margin of frons lateral to vertical setae brown; clypeus sometimes brown. Scutum very shiny and yellow with dark medial stripe on anterior 2/3, one pair of lateral presutural spots and one pair of postsutural bifid stripes; markings usually fused medially, and (south of Canada) sometimes forming large spot nearly reaching lateral and posterior margins. Scutellum yellow, sometimes with lateral corner brown. Metanotum yellow with mediotergite dark brown and venter of katatergite and anatergite brown. Pleuron and legs yellow with faint triangular spot on katepisternum; southern specimens sometimes with spot more distinct and meron with small spot. Calypter margin and hairs dark. Abdomen mostly yellow, sometimes with faint dorsal stripe narrowing to tergite 5 or 6.

***Genitalia***: (Figs 582–587) Epandrium large with sides broadly rounded; posterodistal corner with wide, elongate bar on inner surface. Surstylus subtriangular with apex elongate and dark. Hypophallus broad, faintly sclerotised, lateral margin thicker and folded, with small, weak medial plate with small apical hairs; basomedial region elaborated into one pair of thick folds that meet medially. Basiphallus with dorsal plate fused to phallophorus. Mesophallus indistinct, possibly evident as basal stem of distiphallus and sclerotised ventromedial folds, the latter of which may also be modified paraphalli. Distiphallus perpendicular to basiphallus; length ~ 2 × width; consisting of two broad, fused lobes that slightly widen apically; inner surface minutely spinulose on distal 1/2. Ejaculatory apodeme very large, broadly rounded and pale with sperm pump heavily sclerotised with flat lateral extensions.

###### Hosts.

Acanthaceae – *Blechum*, *Dicliptera*, *Ruellia*. Boraginaceae – *Heliotropium*. Loganiaceae – *Spigelia*. Gentianaceae – *Chelonanthus*. Phrymaceae – *Mimulus*. Plantaginaceae – *Plantago*. Poaceae – *Digitaria*[?], *Panicum*, *Paspalum*. Verbenaceae – *Phyla*. See host species list and comments in Eiseman et al. (2021).

###### Distribution.

**Canada**: AB, NL, ON, QC. **USA**: DE, FL, GA, IA, ID, IL, MA, MD, MI, MS, NC, NY, OH, OK, PA, SC, SD, TX, VA, WV; leaf mine only in KS, WI (Eiseman et al. 2021). Bermuda. Bolivia. Brazil. Dominica. Dominican Republic. Guadeloupe. Martinique.

###### Type material.

***Holotype***: **USA. FL**: Dade Co., Miami, 11.vii.1963, ex. leaf mine on *Blechumpyramidatum* (HT ♂, USNM).

###### Additional material examined.

See Lonsdale (2017).

##### Liriomyza brassicae

Taxon classificationAnimaliaDipteraPhytomyzinae

(Riley)

Figs 588–594

Phytomyza diminuta . Nomen dubium. Walker, 1858: 233. Frick 1952a [synonymy].
Oscinis brassicae Riley 1884: 322.
Agromyza pascuum Meigen, 1830. Misidentification. Melander 1913: 258; Frick 1952a: 402.
Liriomyza cruciferarum Hering, 1927: 461. Frick 1952a: 402 [tentative synonymy, not maintained here].
Liriomyza brassicae . Frick, 1952a: 402, 1957: 68, 1959: 402, Spencer 1963: 356, 1969: 170; Spencer and Steyskal 1986: 127; Scheffer et al. 2007: 772; Lonsdale 2011: 33, 2017: 37; Scheffer and Lonsdale 2018: 87; Eiseman and Lonsdale 2018: 48.
Agromyza diminuta Walker. Misidentification, in part. Coquillett 1898: 78.
Phytomyza mitis Curran, 1931: 97. Frick 1952a: 427, 1959: 402. Spencer 1967 [synonymy not explicit].
Liriomyza hawaiiensis Frick, 1952b: 513. Spencer 1963 [synonymy].
Liriomyza bulnesiae Spencer, 1963: 360. Spencer and Stegmaier 1973 [synonymy].
Liriomyza ornephila Garg, 1971: 241. Sasakawa 1977 [synonymy].

###### Description.

Wing length 1.2–1.6 mm (♂), 1.7–1.8 mm (♀). Length of ultimate section of vein M4 divided by penultimate section: 2.5–3.5. Eye height divided by gena height: 2.9–5.0. Scutum shiny.

***Chaetotaxy***: Two or rarely three ori; two ors. Acrostichal setulae in four rows.

***Colouration***: Lateral margin of frons sometimes with narrow brown margin, varying in strength from indistinct (common) to reaching base of fronto-orbitals; posterolateral corner of frons brown to base of inner or outer vertical seta, sometimes light brown between bases of setae; remainder of head light yellow with back of head and ocellar triangle brown. Scutum dark brown with complete lateral yellow stripe. Scutellum yellow with lateral corner brown. Katatergite yellow with posteroventral margin brown; anatergite light brown with dorsum yellow; mediotergite dark brown. Anepisternum usually with most of ventral margin brown, although sometimes also with posterior margin broadly pigmented or only with small anteroventral spot; anepimeron mottled; meron brown with dorsal 1/3 yellow; katepisternum with large brown triangular spot (not enclosing seta). Calypter margin and hairs grey. Legs yellow with tibiae, tarsi and base of fore coxa brown (fore and mid legs paler, particularly towards apex); sometimes base of femora (often only dorsally) and scraper on hind femur brown; uncommonly with brown streaking on fore femur, but if present, then lateral margin of frons narrowly brown, dark line present between base of vertical setae and hind coxa brown. Abdomen brown with lateral and sometimes posterior margin of tergites 2–4 yellow; tergite 2 sometimes with yellowish mottling and tergites 2–4 sometimes with narrow medial dividing yellow line, and tergite 4 often nearly divided medially into two pairs of spots; tergite 5 and sometimes 6 yellow with large brown medial spot; epandrium with yellow dorsal mottling.

***Genitalia***: (Figs 588–594) Epandrium with one posterodistal spine. Surstylus small and lobate with short apical spine and smaller ventromedial spine, widely spaced. Basiphallus with left lateral and dorsoapical surfaces sclerotised. Hypophallus small, narrow, curved anteriorly and with few apical hairs. Paraphallus narrow or slightly expanded distally. Distiphallus and mesophallus fused, narrow, cylindrical, dark, with small, pale apical chamber; gradual constriction at point of fusion between distiphallus and mesophallus. Ejaculatory apodeme dark and broad, with corners pronounced, width equal to length and stalk narrow.

###### Hosts.

Host genus use by *L.brassicae* is discussed in Lonsdale (2011), with the addition of *Streptanthus* in Eiseman and Lonsdale (2018).

###### Distribution.

Widespread in Nearctic, Oriental and Australasian Regions. Africa. Arabian Peninsula. Europe. Canary Islands. Japan.

###### Type material.

***Holotype* [*brassicae*]: USA. MO**: St. Louis, 30.iv.1876 (1♀, USNM).

***Holotype* [*bulnesiae*]: Venezuela.** Caracas, Botanical Gardens, caught on *Bulnesiaarborea* Engl. (Zygophyllaceae), 5.xii.1958, K.A. Spencer (1♂, NHMUK). [Not examined]

***Syntypes* [*cruciferarum*]: Canary Islands.** La Palma: Santa Cruz (2?, ZMHU) [Not examined]

***Holotype* [*hawaiiensis*]: USA. HI**: Oahu, Honolulu, 1.i.1947, E.C. Zimmerman, ex. leaf of *Cleone* (1♀, BPBM).

***Holotype* [*mitis*]: Canada. MB**: Aweme, 20.vii.1929, R.H. Handford, Type No. 3407 (1♂, CNC).

###### Additional material examined.

**USA. MD**: 4mi SW of Ashton, 29.v.1986, Malaise trap, G.F. and J.F. Hevel (1♂, USNM), Prince Georges Co., Beltsville, 18.ix.1969, R.E. Holmes (1♀, USNM), **NC**: Durham Co., Durham, Pelham Road, 21.vi.2017, em. 8.vii.2017, T.S. Feldman, ex *Cleomehassleriana*, #CSE3909, CNC939820 (1♀, CNC), **NY**: Essex Co., Upper Jay, by Ausable River, East Branch, 2.vi.2012, em. by 23.vi.2012, C.S. Eiseman, ex *Cardaminediphylla* (1♀, CNC), **VA**: Shenandoah Co., Bayse, 21.vii.1974, G.A. Foster (1♀, USNM), **WV**: Bluefield, 17.vi.1970, G. Steyskal (1♂, USNM). Also see Lonsdale (2011, 2017).

###### Comments.

*Liriomyzabrassicae* is difficult to differentiate from *L.sativae* externally and has been reared from many of the same plant species. Colour characters can be used to distinguish most individuals, but since there is some overlap in these features, only the male genitalia can be considered authoritative for identification. The dark, cylindrical, and medially constricted distiphallus is most diagnostic for this species.

While this polyphagous leaf-miner is nearly cosmopolitan, *Liriomyzabrassicae* is not often considered a serious threat to agriculture due to its generally small population sizes (Spencer, 1973a). Several Californian morphotypes that are potentially separate host races or species are not treated here; see Lonsdale (2011) for details.

##### Liriomyza deceptiva

Taxon classificationAnimaliaDipteraPhytomyzinae

(Malloch)

Agromyza deceptiva Malloch, 1918a: 78.
Liriomyza deceptiva . Frick 1952a: 402, 1959: 403; Spencer and Steyskal 1986b: 286.

###### Description.

Wing length 2.0–2.2 mm (♀). Male unknown. Length of ultimate section of vein M4 divided by penultimate section: 1.2. Eye height divided by gena height: 2.6. First flagellomere small with anterodorsal margin angulate. Ocellar triangle and parafacial shiny, parafacial broad and projecting, cheek pronounced. Ocellar tubercle slightly shifted forward, leaving gap between posterior margin and postocellars.

***Chaetotaxy***: Setae relatively thin. Three ori (relatively close to anterior margin of frons); one ors; five fronto-orbitals in holotype. Approximately six scattered rows of acrostichal setuae. Four dorsocentral setae, anterior two pairs very reduced, but larger than surrounding setulae.

***Colouration***: Frons orange, becoming darker to posterior margin; tubercle surrounded by large brown spot; face brownish to venter; clypeus, lower margin of gena and back of head brown; remainder of head yellow with “orbits glossy black” in holotype. Scutum and laterotergites dark brown and shiny with postpronotum, notopleuron and small supra-alar spot yellow. Scutellum yellow with lateral corner brown. Calypter margin and hairs dark. Pleuron brown. Legs yellow with tibiae, tarsi, and dorsal margin of coxae brown. Abdomen dark brown.

###### Distribution.

**USA**: IL, VA. Frick (1952a) notes the presence of this species in VT, but later (Frick 1959) notes that only two females from IL and VA are known.

###### Host.

Unknown.

###### Type material.

***Holotype*: USA. IL**: Alto Pass, 8.v.1917, J.R. Malloch (1♀, INHS). [Not examined]

###### Material examined.

**USA. VA**: Chain Bridge, 23.iv.1922, J.R. Malloch (1♀, USNM).

###### Comments.

*Liriomyzadeceptiva* differs from most *Liriomyza* in having a darker pleuron and head, gracile setae, a clustering of three ori on the anterior margin of the frons and an unusual shape of the head. This unusual combination of characters led Spencer and Steyskal (1986b) to speculate that the species instead belongs to *Amauromyza*, which should be confirmed by dissection when males are located.

##### Liriomyza eupatoriella

Taxon classificationAnimaliaDipteraPhytomyzinae

Spencer

Figs 595–598

Liriomyza eupatoriella Spencer in Spencer and Steyskal 1986b: 288. Eiseman and Lonsdale 2018: 49.

###### Description.

Wing length 1.6–1.7 mm (♂), 1.8 mm (♀). Length of ultimate section of vein M4 divided by penultimate section: 4.0. Eye height divided by gena height: 2.1–4.3.

***Chaetotaxy***: Two ori; two ors. Acrostichal setulae in four rows.

***Colouration***: Head yellow with clypeus brownish, ocellar tubercle and back of head brown, vertical setae on yellow. Scutum shiny and dark brown with postpronotum, notopleuron, and posterolateral (to inner postalar) and posterior margins yellow. Scutellum yellow with lateral corner brown. Metanotum yellow with mediotergite brown. Pleuron yellow with small anteroventral spot on anepisternum, and most of anepimeron and meron brown. Calypter margin and hairs brown. Legs yellow with tibiae and tarsi light brown, hind leg darkest. Abdomen dark brown.

***Genitalia***: (Figs 595–598) Epandrium with single posterodistal spine. Surstylus small and lobate/subtriangular with two long subapical spines. Hypandrium thin with inner lobe not discernable in original holotype dissection. Postgonite cleft on inner face apically and with small inner subapical lobe. Basiphallus evenly sclerotised on left side and with apical projection. Hypophallus small, narrow, and curved. Mesophallus short with small floating ventrobasal sclerite and dorsobasal extension. Distiphallus slightly longer than mesophallus, narrow and cup-shaped.

###### Hosts.

Asteraceae – *Ageratina*, *Vernonia* (Spencer and Steyskal 1986b; Eiseman and Lonsdale 2018).

###### Distribution.

**USA**: IA, KS, MA, MD, MN [leaf mine], VA, WI.

###### Type material.

***Holotype*: USA. WI**: Dane Co., Madison, 12.ix.1976, mining *Eupatoriumrugosum*, em. 28.ix.1976, S. Tavormina (1♂, USNM).

###### Paratypes examined.

**USA. WI**: Dane Co., Madison, 13.ix.1976, S. Tavormina, adult on *Eupatoriumrugosum* (2♀, USNM).

###### Additional material examined.

**USA. IA**: Winneshiek Co., 43°25'55.97"N, 92°0'34.78"W, 16.vii.2015, C.S. Eiseman, *Ageratinaaltissima*, em. 22–23.vii.2015, #CSE1797, CNC654352, CNC654353 (2♂, CNC), **KS**: Riley Co., Konza Prairie Biological Station, 3.vii.2015, em. 21–26.vii.2015, C.S. Eiseman, ex *Vernoniabaldwinii*, #CSE1768, CNC564709, CNC564710 (2♂, CNC), **MA**: Franklin Co., Northfield, 276 Old Wendell Rd., 6.vi.2016, C.S. Eiseman, *Ageratinaaltissima*, em. 22–28.vi.2016, #CSE2613, CNC654063–654069 (5♂ 2♀, CNC), 276 Old Wendell Rd., 6.vi.2016, C.S. Eiseman, *Ageratinaaltissima*, em. 30.vi-7.vii.2016, #CSE2677, CNC654081- 654087 (3♂ 4♀, CNC), **MD**: Somerset Co., Snow Hill, 16.vii.1968, swamp margin, W.W. Wirth (1♀, USNM), **VA**: Shenandoah, Big Meadows, 3.vii.1939, A.L. Melander (1♀, USNM).

###### Comments.

The pale, narrow phallus of *Liriomyzaeupatoriella* closely resembles that of the much more common and widespread species *L.trifolii* and *L.sativae*, but the yellow posterior margin on the scutum and the rounded surstylus with two closely spaced subapical spines are distinctive.

##### Liriomyza eupatorii

Taxon classificationAnimaliaDipteraPhytomyzinae

(Kaltenbach)

Figs 599–602

Agromyza eupatorii Kaltenbach, 1874: 320.
Liriomyza eupatorii . Hendel, 1920: 143, 1931: 217; Frick 1959: 404; Spencer 1969: 174, 1976: 245 [as synonym of orbitella, designation of orbitella lectotype], 1981: 230; Sehgal 1971: 333; Spencer and Steyskal 1986: 129; Lonsdale 2011: 46, 2017: 47; Scheffer and Lonsdale 2018: 87; Eiseman and Lonsdale 2018: 49; Papp and Cerny 2017: 209 [as synonym of L.pusilla (Meigen)]; Eiseman et al. 2021: 25 [stat. reinst.].
Liriomyza orbitella Hendel, 1931–1936: 236. Spencer 1976 [synonymy].

###### Description.

Wing length 1.7–2.2 mm (♂), 1.9–2.0 mm (♀). Length of ultimate section of vein M4 divided by penultimate section: 2.0–2.7. Eye height divided by gena height: 3.0–4.4.

***Chaetotaxy***: Two ori (sometimes three on one side); two ors. Acrostichal setulae in four irregular rows.

***Colouration***: As described for *L.sativae* except as follows: lateral margin of frons brown (not enclosing fronto-orbitals) if first flagellomere brownish on distal margin; only base of hind femur sometimes brown dorsally, or in western North America, femora brown basally and with light dorsoapical mottling (rarely with more extensive pigmentation), but less commonly with only basal markings or entirely yellow; yellow posterolateral spots on scutum sometimes large and distinct; anepisternum dark along anteroventral and ventral margins, sometimes with spot reaching base of anepisternal seta.

***Genitalia***: (Figs 599–602) Epandrium and surstylus as for *L.sativae*. Basiphallus sclerotised along dorsoapical, left lateral and part of right lateral surfaces. Paraphallus narrow. Hypophallus small, narrow, and strongly curved. Mesophallus 1/2 width of distiphallus; mesophallus and distiphallus with complete ventral suture. Distiphallus broadly bell-shaped with sides slightly converging apically and basal margin thick and truncated. Ejaculatory apodeme with stalk narrow and blade broad with dark distal margin.

###### Hosts.

Apocynaceae – *Asclepias*. Asteraceae – *Aster*, *Baccharis*, *Eupatorium*, *Lapsana*, *Mikania*, *Solidago*, *Symphyotrichum*, *Xanthium*; possibly *Callistephus*. Lamiaceae – *Galeopsis* (Benavent-Corai et al. 2005; Lonsdale 2011, 2017; Eiseman and Lonsdale 2018). Putative host genera for which only leaf mines are known: *Ambrosia*, *Erigeron*, *Euthamia*, *Senecio*, and *Zinnia* (Eiseman et al. 2021).

###### Distribution.

**Canada**: AB, BC, MB, NB, NS, ON, QC, SK. **USA**: CA, DE, GA, IL, MA, MI, MS, MT, NC, NY, OK, PA, SC, TN, VA, WA, WV. Europe.

###### Type material.

***Syntypes* [*eupatorii*]: Austria.** [not given]. [Type data unknown]

***Lectotype* [*orbitella*]: Finland.** Esbo (1♂, NMW). [Not examined]

###### Material examined.

**England.** Chippenham Fen., Cambs., Em., “Eup. Cann.”, K.A. Spencer (1♂ [with puparium], USNM), Cornwall, Huckett, 11.vi.1976, on *Eupatorium* (1♂, USNM). **USA. GA**: Robun Co., Addie Branch, E. Fork Chattooga River, 2400', 1.viii.1957, J.G. Chillcott (1♀, USNM), **MA**: 4mi SW of Ashton, 3.vi.1984, G.F. and J.F. Hevel (1♀, USNM), Walden Pond, Concord, 26.vii.1961, W.W. Wirth (1♂, USNM), Hampshire Co., South Hadley, nr. Lithia Springs Reservoir, 11.v.2016, C.S. Eiseman, *Symphyotrichumcordifolium*, em. 27.v.2016, #CSE2505, CNC654242- 654244 (3♂, CNC), **MD**: Somerset Co., Snowhill, 19.v.1968, swamp margin, W.W. Wirth (1♀, USNM), Plummers Isl., 8.v.1915, J.C. Crawford (1♀, USNM), Montgomery Co., Clarksburg, 20–22.v.1988, W.E. Steiner and J.M. Swearingen (1♂, USNM), Colesville, 14.vi.1976, W.W. Wirth (1♂, USNM), Bethseda, G. Steyskal, 4.v.1969 (3♂ 1♀, USNM), 26.v.1968 (1♀, USNM), 27.v.1972 (1♂, USNM), 26.v.1974 (1♂, USNM), “7-12” (1♀, USNM), **MS**: Leland, experimental forest, 11.v.1979, K.A. Spencer (1♀, USNM), **NC**: Smokies, Forneys Ridge, 26.vi.1941, A.L. Melander (1♀, USNM), **OK**: Payne Co., Mehan, 23.iii.2016, M.W. Palmer, *Symphyotrichumpraealtus*, em. 14.iv.2016, #CSE2650, CNC653986 (1♂, CNC), **PA**: Spring Br., 11.vi.1945, DDT Expt. (1♀, USNM), **TN**: East Ridge, 6.v.1952, O. Peck (1♀, USNM), **VA**: Alexandria, 29.vi.1952, W.W. Wirth (1♀, USNM), Warsaw, 26.vii.1952, W.W. Wirth (1♀, USNM), Falls Church, Holmes Run, 21.vi.1961, light trap, W.W. Wirth (1♂, USNM), Shenandoah, Big Meadows, 5.vii.1939, A.L. Melander (1♂ 1♀, USNM), Giles Co., Mountain lake, 7.ix.1977, G.C. Steyskal (4♂, USNM), Great Falls, 4.v.1963, G. Steyskal (1♂, USNM). Also see Lonsdale (2011, 2017).

##### Liriomyza fricki

Taxon classificationAnimaliaDipteraPhytomyzinae

Spencer

Figs 603–607

Liriomyza fricki Spencer, 1965: 35. Spencer 1969: 175; Sehgal 1971: 333; Spencer and Steyskal 1986: 136; Scheffer et al. 2007: 772; Lonsdale 2011: 49, 2017: 51; Scheffer and Lonsdale 2018: 87; Eiseman and Lonsdale 2018: 50.

###### Description.

Wing length 1.3–2.0 mm (♂), 1.4–1.8 mm (♀). Length of ultimate section of vein M4 divided by penultimate section: 1.7–3.5. Eye height divided by gena height: 3.0–4.2, sometimes up to 5.1 in Canadian specimens. Scutum lightly dusted with pruinosity.

***Chaetotaxy***: Two ori (anterior seta small to absent); two ors. Acrostichal setulae in two rows (sometimes three anteriorly).

***Colouration***: Head yellow with posterolateral margin of frons lateral to (and not touching) vertical setae yellow to brown; back of head brown dorsally; ocellar tubercle brown; clypeus brown to light brown with centre yellow. Scutum with complete lateral yellow stripe broadly overlapping margin of scutellum. Scutellum yellow with lateral corner brown. Katatergite yellow; anatergite sometimes brownish ventrally; mediotergite dark brown. Pleuron yellow with large spot on katepisternum and meron, and with small (sometimes very faint to indistinct) anteroventral spot on anepisternum and anepimeron; anepimeron sometimes brown mottled. Calypter margin brownish. Legs yellow with fore tibia and tarsi brownish, mid and hind tarsi brown, and mid and hind tibiae brown at base, apex and on dorsal surface; material from western United States with tibiae yellow with dorsum faintly brown (paler to entirely yellow on anterior legs) and tarsi only brownish on distal three segments; femora of Canadian specimens sometimes with pale dorsal streaking or with brown dorsobasal spot. Abdomen yellow with dorsum, epandrium and surstylus brown. Variation in Canadian material discussed in Lonsdale (2017).

***Genitalia***: (Figs 603–607) Epandrium with one posterodistal spine. Surstylus darkly pigmented with one large and one small subapical spine. Inner surface of epandrium with one pair of dark bars with apical spine. Basiphallus broadly sclerotised along left lateral and dorsal surfaces. Swollen apical section of ejaculatory duct short, wide, and narrowed apically. Hypophallus relatively short with long apical hairs. Paraphallus absent. Mesophallus narrow, cylindrical, and fused to distiphallus; mesophallus and distiphallus with ventral suture. Distiphallus large, weakly pigmented, bowl-shaped with minute internal reticulations and spinules, angled dorsally, slightly bilobed, elongate, and with one pair of short, wide membranous tubules. Ejaculatory apodeme well-developed and dark with base of duct lightly sclerotised, stem narrow and blade large and thickened along margin; sclerite on sperm pump broad, dark, with thick margin.

###### Hosts.

Fabaceae – *Caraganapubescens* (uncertain record; Spencer, 1969), *Lathyrus*, *Medicago*, *Melilotus*, *Oxytropis*, *Pisum*, *Trifolium*, *Vicia*.

###### Distribution.

**Canada**: AB, BC, MB, NB, NS, NT, ON, PE, QC, SK, YT. **USA**: CA, MA, MD, MI, MN, NY, WA, WI, WY.

###### Type material.

***Holotype*: USA. WA**: Benton Co., Prosser (1♂, Location unknown).

###### Additional material examined.

**USA. MA**: Hampshire Co., Pelham, Quarry St., 6.vii.2013, em. 17.vii.2013, C.S. Eiseman, ex *Trifoliumrepens*, #CSE709, CNC384758 (1♂, CNC). Also see Lonsdale (2011, 2017).

##### Liriomyza galiivora

Taxon classificationAnimaliaDipteraPhytomyzinae

(Spencer)

Figs 608–611

Praspedomyza galiivora Spencer, 1969: 199.
Galiomyza galiivora . Spencer 1981: 291; Spencer and Steyskal 1986: 137; Papp and Černý 2017: 28; Černý et al. 2020: 208.
Liriomyza galiivora . Spencer & Martinez, 1987: 261 (attrib. to Tschinhaus); Spencer 1987: 877, 1990: 235; Lonsdale 2017: 55; Eiseman and Lonsdale 2018 : 50.

###### Description.

Wing length 1.5–1.9 mm (♂), 1.8–1.9 mm (♀). Length of ultimate section of vein M4 divided by penultimate section: 2.7–5.0. Eye height divided by gena height: 3.2–9.3. Scutum shiny.

***Chaetotaxy***: Two ori (rarely one); two ors; sometimes appearing as three ori and one ors. Acrostichal setulae in four rows.

***Colouration***: Body brown with first flagellomere yellow; halter white; scutellum, postpronotum and notopleuron slightly paler. Calypter margin and hairs dark.

***Genitalia***: (Figs 608–611) Epandrium relatively long and tapered ventrally, with one posteromedial and one apical tubercle. Surstylus small, subtriangular, and with two spines. Basiphallus largely membranous with dorsum lightly sclerotised. Hypophallus broad, flat, and lightly sclerotised. Distiphallus composed of narrow stem fused to similarly narrow mesophallus, with broad interfolding cup-like apex with smaller, bilobed (each lobe sclerotised medially), mostly membranous distal section. Ejaculatory apodeme with dark, gradually tapering stalk and smooth, pale blade; sclerite of sperm pump with dark rounded lateral extensions continuous with sclerotised base of duct and base of ejaculatory apodeme.

###### Hosts.

Rubiaceae – *Diodia*, *Galium* (Eiseman and Lonsdale 2018).

###### Distribution.

**Canada**: AB, BC, ON, QC (puparium and reared braconid parasitoid). **USA**: AK, MA, MD, MN, OH, WV. Europe, Russia (Černý et al. 2020).

###### Type material.

***Holotype*: Canada. AB**: White Mud Cr., nr. Edmonton, 23.vi.1966, ex. *Galiumboreale*, Type No. 10421 (1♂, CNC).

###### Material examined.

See Lonsdale (2017).

##### Liriomyza helianthi

Taxon classificationAnimaliaDipteraPhytomyzinae

Spencer

Figs 640–644

Liriomyza helianthi Spencer, 1981: 240. Spencer and Steyskal 1986: 289; Lonsdale 2011: 56, 2017: 58; Eiseman et al. 2019: 313.
Liriomyza virginica Spencer in Spencer and Steyskal 1986: 297. Lonsdale 2011 [synonymy].

###### Description.

Wing length 1.3–1.5 mm (♂), 1.5–1.6 mm (♀). Length of ultimate section of vein M4 divided by penultimate section: 2.1–3.7. Eye height divided by gena height: 4.0–5.3. Scutum subshiny.

***Chaetotaxy***: Two ori; two ors (rarely three). Acrostichal setulae in four rows.

***Colouration***: Head light yellow with ocellar tubercle, back of head, clypeus, and posterolateral region of frons to inner vertical seta (paler between verticals) dark brown. Scutum dark with complete lateral yellow stripe. Scutellum yellow with lateral corner brown. Katatergite yellow and anatergite brown with posterodorsal corner yellow; mediotergite dark brown. Anepimeron mostly brown, anepisternum with variable brown striping on ventral 1/2, meron brown with dorsal margin yellow and katepisternum brown on ventral 3/4 (yellow around base of seta). Calypter margin grey. Tibiae, tarsi, and bases of coxae brown, with tarsi and sometimes tibiae becoming paler basally; femora sometimes with very small, weak basal spot. Abdomen brown, tergites narrowly yellow posteriorly and broadly yellow laterally.

***Genitalia***: (Figs 640–644) Epandrium with one posterodistal spine. Surstylus with single subapical spine. Basiphallus sclerotised along left lateral and most of dorsal surfaces. Paraphallus large and well-defined, becoming broader distally and sometimes strongly clavate; sometimes not strongly widened apically and base of distiphallus slightly thicker. Hypophallus well-developed. Mesophallus short and slightly wider and darker than distiphallus base; completely fused to distiphallus. Distiphallus with complete ventral suture widening apically; gradually widening in ventral view, with broad apical chamber enclosing paired fringed structures; distiphallus straight to slightly sinuate in profile and with relative dimensions and amount of pigment slightly variable between specimens. Ejaculatory apodeme large, dark, and well-developed; stem narrow at base and blade broad; sperm pump with sclerotised bar broad, thick and wide at ends.

***Variation***: Some material darker than “typical” specimens described above. Sometimes with yellow lateral stripe on scutum entirely brown to mottled postsuturally; sometimes lateral margin of frons brown (sometimes also including base of ors), femora striated or darker apically (at least on fore femur), and pleuron darker, sometimes with only dorsal margin of anepisternum, katepisternum and katatergite yellow; abdomen sometimes entirely brown. Additional variation among Canadian specimens discussed in Lonsdale (2017).

###### Hosts.

Asteraceae – *Artemisiabiennis*, *Chrysogonum*, *Helianthusannuus* L., *Xanthiumstrumarium* L., *Ambrosiapsilostachya* DC, “Nasturtium” (Spencer and Steyskal 1986; Lonsdale 2011, 2017; Eiseman et al. 2019).

###### Distribution.

**Canada.** AB, BC, MB, NB, NS, ON, QC, SK. **USA.** CA, CO, MD, NC, NM, OR, VA, WA.

###### Type material.

***Holotype* [*helianthi*]: USA. CA**: Stanislaus Co., Patterson, 27.ix.1948, swept on *Helianthusannuus*, Lot 162-1, K.E. Frick (1♂, CASC).

***Holotype* [*virginica*]: USA. VA**: Patrick Co., Vesta, 2800ft, 30.v.1962, J.R. Vockeroth (1♂, CNC).

###### Additional material examined.

**USA. MD**: Montgomery Co., Bethseda, 7.vii.1970, G. Steyskal (1♂, USNM), **NM**: Cimarron, 26.v.1969, W.W. Wirth, river margin (1♂ [head missing], USNM), **OR**: Lake Co., 3.vii.1971, 10 mi NE Christmas Valley, G. Steyskal (1♂ [head missing], USNM), **WA**: Kamiac Butte, 25.vii.1914, A.L. Melander (1♂, USNM). Also see Lonsdale (2011, 2017).

##### Liriomyza philadelphivora

Taxon classificationAnimaliaDipteraPhytomyzinae

Spencer

Figs 612–615

Liriomyza philadelphivora Spencer, 1969: 182. Spencer and Steyskal 1986b: 291; Scheffer et al. 2007: 772; Lonsdale 2017: 73; Scheffer and Lonsdale 2018: 87.

###### Description.

Wing length 1.8–2.0 mm (♂), 2.0–2.3 mm (♀). Length of ultimate section of vein M4 divided by penultimate section: 2.0–2.3. Eye height divided by gena height: 4.6–4.8. Notum shiny to subshiny.

***Chaetotaxy***: Two ori (three on left side in holotype); two ors. Acrostichal setulae in four rows.

***Colouration***: Head yellow, with back of head (excluding margin) brown, and small medial spot on ocellar tubercle brown with stripes extending to inner margin of ocelli. Scutum dark brown with lateral margins yellow and posterior margin yellow, including large subquadrate region anterior to scutellum; posterolateral margin of brown spot with one pair of rounded extensions on each side, suggesting underlying vittate pattern similar to that seen in *L.blechi*. Scutellum and metanotum yellow with mediotergite brown. Pleuron yellow with ventral 1/2 of katepisternum and meron dark brown. Calypter margin dark. Legs yellow with brownish tint on mid and hind tibiae (darker on hind leg). Abdomen yellow with epandrium and ill-defined dorsomedial stripe brown.

***Genitalia***: (Figs 612–615) Epandrium with one posterodistal spine. Surstylus with three dorsal setae and no spine; produced ventrally into long, pointed, outwardly curved process. Basiphallus sclerotised on dorsal and left lateral surfaces, with left distal margin produced past distiphallus as strong lobe. Hypophallus linear, narrow and pointed. Mesophallus narrow, dark, slightly longer than high or wide, with sides of ventral suture produced as irregular carina. Distiphallus cup-like, pale (darker laterally), widest medially and slightly compressed dorsoventrally.

###### Hosts.

Hydrangeaceae – *Philadelphus* spp.

###### Distribution.

**Canada**: ON. **USA**: DC, NY.

###### Type material.

***Holotype*: Canada. ON**: Ottawa, em. 4.vii.1962 ex. *Philadelphus* (1♂, CNC).

###### Additional material examined.

**Canada. ON**: Ottawa, Central Experimental Farm, adult on *Philadelphus* sp., 8.vi.2017, O. Lonsdale, CNC799487 (1♀, CNC). Also see Lonsdale (2017).

##### Liriomyza sativae

Taxon classificationAnimaliaDipteraPhytomyzinae

Blanchard

Figs 22 , 109 , 110 , 621–625

Liriomyza sativae Blanchard, 1938: 354. Frick 1959: 405; Spencer 1973a: 219, 1984: 23; Spencer and Steyskal 1986: 292; Rauf et al. 2000: 257; Scheffer and Lewis 2005: 181; Lonsdale 2011: 93, 2017: 81; Eiseman and Lonsdale 2018: 54.
Liriomyza subpusilla Frost, 1943: 255 [preoccupied by Malloch, 1914].
Liriomyza verbenicola Hering, 1951: 43. Spencer and Steyskal 1986b [synonymy].
Liriomyza pullata Frick, 1952b: 509. Spencer 1973a [synonymy].
Liriomyza canomarginis Frick, 1952b: 511. Spencer 1973a [synonymy].
Liriomyza minutiseta Frick, 1952b: 512. Spencer 1973a [synonymy].
Liriomyza propepusilla Frost, 1954: 73 [replacement name for subpusilla]. Frick 1957: 62. Steyskal 1973 [synonymy].
Liriomyza munda Frick, 1957: 61. Spencer 1973a [synonymy].
Liriomyza pictella (Thompson). Misidentification, in part. Frick 1957: 66.
Liriomyza guytona Freeman, 1958: 344. Steyskal (1964) [as synonym of munda].

###### Description

**(Figs 109, 110).** Wing length 1.3–1.6 mm (♂), 1.4–1.8 mm (♀). Length of ultimate section of vein M4 divided by penultimate section: 1.6–4.0. Eye height divided by gena height: 4.4–5.8. Scutum shiny to subshiny.

***Chaetotaxy***: Two ori (anterior seta sometimes reduced to absent), sometimes three; two ors. Acrostichal setulae in four irregular rows.

***Colouration***: Posterolateral corner of frons brown, usually fading to yellow at base of outer or inner vertical seta; few western specimens with narrow brown margin along fronto-orbital plate tapering to anterior ori; back of head above foramen, ocellar tubercle and clypeus brown; venter of gena with narrow brownish stripe that sometimes fades posteriorly; anterior margin of first flagellomere rarely appearing lightly infuscated. Scutum with complete lateral yellow stripe, sometimes with brown mottling posteriorly. Scutellum yellow with lateral corner dark brown. Katatergite sometimes with posterior margin to posterior 1/2 brown; anatergite brown; mediotergite dark brown. Pleuron yellow with ventral 2/3 of katepisternum, meron, small spot(s) on anepimeron and anteroventral corner of anepisternum brown; anepisternum sometimes predominantly brown along ventral margin; specimens from western North America sometimes darker with only dorsal 1/4 of anepisternum (as well as deep posterodorsal emargination), meron and katepisternum yellow; if only dorsal margin of anepisternum narrowly yellow (rare), lateral margin of frons infuscated, femora more extensively mottled dorsally or only yellow apically and distoventrally, lateral margin of scutum sometimes brownish postsuturally, metanotum darker and abdominal tergites entirely brown. Calypter margin grey. Legs yellow with tibiae, tarsi and bases of coxae light brown; bases or dorsal bases of hind and (less commonly) mid femora sometimes brown (if so, base of fore femur occasionally also brown); fore femur, and much less commonly mid and hind femora sometimes with outer-dorsal striations. Abdomen brown with lateral and sometimes posterior margins yellow (sometimes widely on tergite 5); epandrium dark with dorsum and perianal region usually yellowish.

***Genitalia***: (Figs 621–625) Epandrium with one posterodistal spine. Surstylus lobate with one apical spine. Phallophorus well-developed with high dorsum. Basiphallus sclerotised along most of left-lateral and dorsomedial surfaces, leaving distal section of duct exposed. Hypophallus linear, narrow, with several apical hairs. Paraphallus small, linear. Mesophallus separate from distiphallus, small, narrow, subcylindrical with slight ventral carina along suture. Distiphallus simple, cup-like, narrowed basally; with apical, basal, and ventral surfaces more well-sclerotised, forming weak C-shape in profile. Ejaculatory apodeme pale and with base of blade and stem relatively dark and narrow, sometimes broader apically with corners more pointed.

###### Hosts.

Host genera of the polyphagous *L.sativae* are discussed in Lonsdale (2011); *Rafinesquia* (Asteraceae) and *Astragalus* (Fabaceae) added by Eiseman and Lonsdale (2018).

###### Distribution.

**Canada**: ON (likely to be widespread in southern Canada and in greenhouses, although the cold climate will likely prevent it from ever becoming a serious pest on outdoor crops). **USA**: TX to CA, CO and SC, and further north in greenhouses (OH, MD, PA). Neotropics. Introduced globally throughout growing regions.

###### Type material.

***Holotype* [*sativae*]: Argentina.** “las larvas producen galerias en las hojas de la alfalfa en General Pico, Pampa; halladas por mi excelente colabodaro Juan Williason, xi.1937”, ex. *Medicagosativa* (1♀, Museu de la Plata, Buenos Aries, Argentina). [Not examined]

***Holotype* [*canomarginis*]: USA. HI**: Oahu, Kaimuki, 12.iv.1921, O.H. Swezey, ex. *Indigofera* sp. (1♀, BPBM). [Not examined]

***Holotype* [*guytona*]: USA. AL**: Auburn, 20.iv.1957, ex. beans, C.C. Freeman (1♂, USNM).

***Holotype* [*minutiseta*]: USA. HI**: Oahu, Honolulu, 7.ix.1951, W.C. Mitchell, ex. tomato (1♀, BPBM). [Not examined]

***Holotype* [*munda*]: USA. AL**: San Joaquin Co. Tracy, 22.ix.1949, L.L. Lewallen, ex. leaf of tomato (1♂, USNM).

***Holotype* [*subpusilla*]: USA. KS**: Manhattan, 14.x.1933, C.W. Sabrosky (1♂, USNM).

***Holotype* [*pullata*]: USA. HI**: Kanoa, Molokai, 3.iii.1929, O.H. Swezey, ex. *Datura* sp. (1♀, BPBM). [Not examined]

***Holotype* [*verbenicola*]: USA. NM**: Las Cruces, ex. *Verbena* sp. (1♀, ZMHU). [Not examined]

###### Additional aterial examined.

**Costa Rica.** San Rafael de Oro de Aqua, 3.ii.1982, ex. beans, 1.iii.1982, K.A. Spencer (1♂ 1♀, USNM). **USA. CA**: San Diego Co., Coyote Canyon, 9.iii.2017, em. 2.iv.2017, C.S. Eiseman, ex *Lupinusarizonicus*, #CSE3351, CNC940107 (1♂, CNC), Tubb Canyon, 11.iii.2017, em. 28.iii–6.iv.2017, C.S. Eiseman, ex *Rafinesquianeomexicana*, #CSE3311, CNC940098, CNC940099 (2♂, CNC), **CO**: Pitkin Co., Redstone, Avalanche Creek, near Rte. 133, 12.vii.2015, em. 25.vii.2015, C.S. Eiseman, ex *Astragaluscicer*, #CSE1832, CNC564629 (1♂, CNC), **FL**: Floral Acres, Inc., “on *Chrysantemum* leaves”, 2.ii.1965, intercepted at San Juan, P.R., J. Lojo (1♂, USNM), Ruskin, 15.i.1945, English pea (2♂ 2♀, USNM), **GA**: Tifton, reared from Lupine leaf, G.R. Manglitz, 6.iv.1955 (1♀, USNM), 27.iv.1955 (2♀, USNM), 28.iv.1955 (1♂ 3♀, USNM), 2.v.1955 (3♂ 2♀, USNM), **HI**: Maui, Omaopio, 4.iii.1966, ex. tomato, N. Miyahira (2♂, USNM), **LA**: Chase, tomato leaf mine (1♀, USNM), **MD**: Montgomery Co., Colesville, 4.ix.1977, Malaise trap, W.W. Wirth (1♂, USNM), **OH**: Medina Co., reared from greenhouse tomato leaves, 23.vii.1963, R.B. Neiswander (4♂ 5♀, USNM), **VA**: Giles Co., Mountain Lake Biol Stn., 37°22'31"N, 80°31'18"W, Mal. over pond inflow, 23–30.v.2005, S.A. Marshall (1♂, DEBU), **WY**: Red Gulch Road, Hwy. 14 nr. Shell, pans in barren area nr. cottonwds., cow dung, J.E. Swann (1♂, DEBU). Also see Lonsdale (2011, 2017).

##### Liriomyza temperata

Taxon classificationAnimaliaDipteraPhytomyzinae

Spencer

Figs 626–631

Liriomyza temperata Spencer in Spencer and Steyskal 1986: 294. Lonsdale 2017: 94.

###### Description

[**from Lonsdale (2017)].** Wing length 2.4 mm (♂), 2.1–2.7 mm (♀). Length of ultimate section of vein CuA1 divided by penultimate section: 2.1–2.9. Eye height divided by gena height: 3.8–6.9 [head missing in holotype]. Scutum subshiny.

***Chaetotaxy***: Two ori; two ors. Acrostichal setulae in four rows.

***Colouration***: Calypter margin brown. Head yellow with ocellar triangle and back of head dark brown; lateral corner of frons dark brown, becoming paler to base of outer vertical seta; clypeus dark brown with centre sometimes paler. Scutum with complete yellow stripe laterally. Lateral corner of scutellum with small brown spot. Katatergite brown posteriorly; anatergite brown with dorsum yellow; mediotergite dark brown. Anepisternum with brown stripe across ventral 1/2; anepimeron yellow with brown streaking (paler posteriorly); meron brown with dorsum yellow; ventral 2/3 of katepisternum brown. Legs yellow with bases of coxae brown, tibiae, and tarsi brown (anterior legs paler); hind femur sometimes brown dorsobasally, and if so, fore and mid femora sometimes also similarly brown. Abdomen dark brown with lateral margin of tergites yellow.

***Genitalia***: (Figs 630, 631) Surstylus broad with two long posterobasal spines (not apical to subapical). Phallophorus with long, narrow dorsal process that is sharply bent ventrally. Basiphallus with left lateral and dorsoapical surfaces sclerotised. Hypophallus short with long subapical hairs. Paraphallus pale and narrow with venter darker. Mesophallus short, thick-walled dorsally, narrowed basally and distally; mesophallus and distiphallus with complete ventral suture. Distiphallus cup-shaped, slightly compressed dorsoventrally towards darkened base; with few spines along distal margin of shallow medial and apical chambers. Ejaculatory apodeme with narrow stem and clear marginal band; sclerite of sperm pump highly reduced.

***Variation***: (Figs 626–629) Non-type males differ as follows: wing length 1.9–2.2 mm; eye height divided by gena height 6.7–8.0; two ori and ors; femora entirely yellow; stripe on anepisternum sometimes narrower; extension on the left distal margin of the basiphallus shorter and narrower; paraphallus slightly thicker; mesophallus ~ 1/3 shorter; distiphallus higher, stouter, with more conspicuously delimited short medial chamber and with base broader (apparently shorter with more rounded base in illustration in original publication, but this may be an artifact); ejaculatory apodeme more weakly sclerotised marginally on blade and not more heavily sclerotised on lateral margin of blade furthest from duct; sclerite of sperm pump weaker and not produced laterally.

###### Host.

Unknown.

###### Distribution.

**Canada**: ON. **USA**: NC, TN, VA.

###### Material examined.

See Lonsdale (2017).

##### Liriomyza trifoliearum

Taxon classificationAnimaliaDipteraPhytomyzinae

Spencer

Figs 632–635

Liriomyza pictella . Misidentification. Frick 1959: 408.
Liriomyza trifoliearum Spencer in Spencer and Stegmaier 1973: 107. Spencer and Steyskal 1986: 296; Scheffer et al. 2007: 772; Lonsdale 2011: 104, 2017: 96; Scheffer and Lonsdale 2018: 87; Eiseman and Lonsdale 2018: 56.

###### Description.

Wing length 1.8–2.3 mm (♂), 1.8–2.2 mm (♀). Length of ultimate section of vein M4 divided by penultimate section: 1.5–4.0. Eye height divided by gena height: 2.8–3.2. Scutum subshiny.

***Chaetotaxy***: Two ori (sometimes three on one side); two ors. Acrostichal setulae in four or five irregular rows.

***Colouration***: Head mostly yellow; ocellar triangle, back of head, clypeus and posterolateral margin of frons to base of inner vertical seta dark brown; fronto-orbital plate brown to base of posterior or anterior ors (fading anteriorly), or with light mottling at base of setae; face light brown to yellow; posteroventral margin of gena with narrow brownish stripe; first flagellomere sometimes with distal margin infuscated. Scutum with complete lateral yellow stripe that is sometimes brown posteriorly. Scutellum yellow with lateral corner brown. Metanotum brown, usually with katatergite partially yellow dorsally. Pleuron brown with dorsal 1/4 or less of anepisternum, mottling on anepimeron, dorsal margin of meron and dorsal or dorsomedial margin of katepisternum yellow; katepisternal seta usually enclosed by brown but sometimes on yellow border. Calypter margin and hairs dark. Coxae (sometimes excluding apex to distal 2/3 of fore coxa), tibiae and tarsi brown; base of femora brown (sometimes only dorsally in eastern material or with mid femur entirely yellow), remainder of segment variably patterned, but usually paler on fore or mid femora, and infrequently entirely brown with only apex and anteroventral surface yellow. Abdomen brown, sometimes with posterior margin of tergites 2–5 yellow.

***Genitalia***: (Figs 632–635) Epandrium with one posterodistal spine. Surstylus slightly narrowing distally and with two subapical spines with outer spine slightly smaller. Apical membrane of basiphallus produced into one pair of pointed paraphalli. Hypophallus absent or present as small offset sclerotised plate. Mesophallus slightly longer than wide, most heavily sclerotised laterally and dorsally; mesophallus and distiphallus with complete ventral suture. Distiphallus short, entirely divided medially with halves narrow, weakly sclerotised apically and with several inner-marginal points. Ejaculatory apodeme narrow and poorly developed with venter of sperm pump broadly sclerotised.

###### Hosts.

Cleomaceae – *Cleome*. Fabaceae – *Coronilla*, *Lupinus*, *Medicago*, *Phaseolus*, *Pisum*, *Securigera*, *Trifolium*. Lamiaceae – *Trichostema*. Malvaceae – *Eremalche*. Solanaceae – *Solanum*. (Lonsdale 2011, 2017; Eiseman and Lonsdale 2018)

###### Distribution.

**Canada**: AB, BC, NB, NS, ON, PE, QC, SK. **USA**: AZ, CA, DE, FL, MA, MD, NM, NY, OH, OR, PA, UT, VA, WA, WI, WV.

###### Type material.

***Holotype*: USA. FL**: Gainesville, 24.iv.1964, ex. *Trifoliumrepens*, D.H. Habeck (1♂, USNM).

###### Additional material examined.

**USA. CA**: Lake Co., McLaughlin Natural Reserve, 18.iv.2016, E. LoPresti, *Trichostemalaxum*, em. 1–4.v.2016, #CSE2437, CNC654495, CNC654496 (2♂, CNC), **FL**: Gainesville, R.N. Wilson (1♂, USNM), **MD**: Montgomery Co., Colesville, 4.vi.1977, W.W. Wirth (1♀, USNM), Colesville, 4.ix.1977, Malaise trap, W.W. Wirth (1♀, USNM), **OR**: Mt. Hood, Hoods Rapids, 29.vii.1921, A.L. Melander (1♂, USNM), Lake Co., 9mi SE Fort Rock, 3.vii.1971, G. Steyskal (2♂, USNM), **UT**: Timpanogos Mt., 25.vi.1940, A.L. Melander (1♂, USNM), Salt Lake Laboratory, 22.viii.1913, L.P. Rockwood (1♂ 2♀, USNM), Salt Lake, C.N. Ainslie, 15.vii.1912, reared from *Agromyza* mines (3♂ 3♀, USNM), Salt Lake, C.N. Ainslie, reared from alfalfa leaf mines, “May 11” (1♂, USNM), Salt Lake, C.N. Ainslie, 2.ix.1912, reared from alfalfa mine (1♀, USNM), Salt Lake, T.H. Parks (1♂ 2♀, USNM), **WA**: Eusum, 28.vi.1917, A.L. Melander (1♂, USNM). Also see Lonsdale (2011, 2017).

##### Liriomyza trifolii

Taxon classificationAnimaliaDipteraPhytomyzinae

(Burgess)

Figs 111 , 112 , 636–639

Oscinis trifolii Burgess, 1880: 201.
Agromyza trifolii . Coquillett, 1898: 78; Malloch 1913: 278 [as synonym of Agromyzapusilla Meigen].
Liriomyza trifolii . De Meijere, 1925: 282; Hendel 1931: 213; Frick 1952a: 405, 1959: 410; Spencer 1965: 37 [neotype designation], 1973a: 226, 1984: 25; Spencer and Steyskal 1986: 296; Scheffer and Lewis 2006: 991; Scheffer et al. 2007: 772; Lonsdale 2011: 106, 2017: 97; Eiseman and Lonsdale 2018: 56; Monteiro et al. 2019: 173; Eiseman et al. 2021: 32.
Liriomyza phaseolunata Frost, 1943: 256. Frick 1952a: 404, 1959: 408. Spencer and Steyskal 1986 [synonymy].
Liriomyza alliovora Frick, 1955: 88. Frick 1959: 401. Spencer 1973a [synonymy].

###### Description

**(Figs 111, 112).** Wing length 1.2–1.7 mm (♂), 1.5–1.9 mm (♀). Length of ultimate section of vein M4 divided by penultimate section: 1.7–3.1. Eye height divided by gena height: 2.1–3.0. Scutum with light greyish pruinosity, rarely subshiny. First flagellomere rounded or with slight anterodorsal angle.

***Chaetotaxy***: Two ori; two ors. Acrostichal setulae in two to four scattered rows.

***Colouration***: Head yellow with back of head above foramen, ocellar tubercle, clypeus, and posterolateral margin of frons (not reaching base of outer vertical seta) brown. Scutum with complete lateral yellow stripe. Scutellum yellow with lateral corner brown. Metanotum brown with sclerites lateral to scutellum paler, sometimes with katatergite entirely yellow. Pleuron yellow with large ventral spots on katepisternum (not including seta base) and meron, and anepisternum and anepimeron with small anteroventral spots. Calypter margin and hairs brownish. Legs yellow with base of fore coxa sometimes brown, fore femur sometimes with dorsal mottling, base of mid and hind femora sometimes partially brown dorsally, and tibiae and tarsi brown (paler on fore legs). Abdomen brown with lateral margin broadly yellow and posterior margin of tergites (1)2–4 yellow; tergites 2–4 sometimes with yellow posteromedial emargination (sometimes forming complete line on tergite 4) and tergite 6 with anteromedial spot; epandrium brown, often with dorsum light brown to yellow; tergite 2 sometimes yellow along midline in females; if male abdomen entirely brown (some South American material), only two rows of acrostichal setulae present and most of anepisternum brown.

***Genitalia***: (Figs 636–639) Epandrium with one posterodistal spine. Surstylus subtriangular, apex slightly truncated and with small inner-distal spine. Basiphallus sclerotised along left lateral margin and partially along dorsal margin; largely recessed distally where swollen section of ejaculatory duct dominates. Paraphallus narrow, weakly sclerotised. Hypophallus small, narrow. Mesophallus narrow, pale, slightly longer than wide and weakly fused to distiphallus. Distiphallus small, cup-shaped with subapical constriction; apex with characteristic minute triangular-shaped sclerotisations. Ejaculatory apodeme small with base curved and apex relatively narrow and clear; sperm pump with transverse sclerotisation.

###### Distribution.

Widespread throughout the Americas, Europe, and Asia.

###### Known hosts.

Host genera are listed in Lonsdale (2011); *Abronia* (Nyctaginaceae) and *Mecardonia* (Plantaginaceae) were added by Eiseman and Lonsdale (2018) and Eiseman et al. (2021), respectively.

###### Type material.

***Neotype* [*trifolii*]: USA. IN**: Lafayette, from Alfalfa, 3.ix.1913, J.M. Aldrich (1♂, USNM).

***Holotype* [*phaseolunata*]: USA. NJ**: Bridgeton, 24.viii.1942, B.B. Pepper (1♂, Lost).

***Holotype* [*alliovora*]: USA. IA**: Ames, 8.vi.1932, H.M. Harris, ex. leaf of onion (1♂, USNM).

###### Additional material examined.

**Bermuda.** Devonshire, Par., Devonshire Marsh, 20.ix.1987, D.J. Hilburn, N.E. Woodley (1♂, USNM), Pembroke Par., Admirality House Park, 15.xi.1987, D.J. Hilburn, N.E. Woodley (1♂, USNM). **USA. CA**: Imperial Co., Algodones Dunes, along Rte. 78, 7.iii.2017, em. 19.iii.2017, C.S. Eiseman, ex *Abroniavillosa*, #CSE3250, CNC940076 (1♂, CNC), **DE**: Gumboro, 2.viii.1952, C. Sabrosky (1♀, USNM), Rehoboth, 2.viii.1941, A.L. Melander (1♀, USNM), **FL**: Lake Co., Alexander Springs, 26.iii.2013, em. 12–20.iv.2013, C.S. Eiseman, ex *Hydrocotyleverticillata*, #CSE275, CNC358471 (1♂, CNC), **MD**: Laurel, Chrysanthemum, 31.i.1962 (10♂18♀, USNM), Montgomery Co., Colesville, 28.v.1977 (1♀, USNM), Colesville, Malaise trap, W.W. Wirth, 26.vi.1977 (2♀, USNM), 30.vi.1977 (1♀, USNM), 4.ix.1977 (1♂, USNM), **MI**: Mio, 29.v.1937, H. Milliron (1♀, USNM), **NC**: Scotland Co., Laurinburg, St. Andrews University, 2.vi.2015, em. 20.vi.2015, T.S. Feldman, ex *Trifoliumrepens*, #CSE1633, CNC564626 (1♂, CNC), **NV**: Clark Co., Charleston Peak, Kyle Canyon, ca 2200 m, 14.vii.1966, P.H. Arnaud, Jr. (1♀, CASC), **NY**: Orange Co., Florida, L.L. Pechuman, reared October, leaf miner on celery (1♂, USNM), **OH**: Wooster, 10.v.1972, ex. celery leaves, J.P. Sleesman (3♂ 3??, USNM), **VA**: Northampton Co., Kiptopeke, 4–6.x.1986, W.E. Steiner et al., Malaise trap, dunes between cliff and beach (3♀, USNM). Also see Lonsdale (2011, 2017).

##### Liriomyza violivora

Taxon classificationAnimaliaDipteraPhytomyzinae

(Spencer)

Figs 116 , 117 , 645–652

Galiomyza violivora Spencer in Spencer and Steyskal 1986b: 298; Scheffer et al. 2007: 772.
Liriomyza violivora . Lonsdale, 2017: 101; Scheffer and Lonsdale 2018: 87; Eiseman and Lonsdale 2018: 59.

###### Description

**(Figs 116, 117, 652).** Wing length 1.7–2.2 mm (♂), 1.8–2.0 mm (♀). Length of ultimate section of vein M4 divided by penultimate section: 1.9–2.5. Eye height divided by gena height: 2.5–3.4. Face and centre of frons soft. Fronto-orbital plate visible laterally and slightly projecting anteriorly. Clypeus broad anteriorly. Notum subshiny.

***Chaetotaxy***: Two to three ori; two ors. Postocellar and ocellar setae as long as ors. Orbital setulae dark, pronounced and reclinate. Four dorsocentrals, one presutural, decreasing in length anteriorly with anterior two subequal. Acrostichal setulae in four scattered rows.

***Colouration***: Head light brown to dirty yellow, with clypeus, palpus, back of head, face, and lower margin of gena (very dark and shiny) brown; lunule darker ventrally, broad and shallow; frons yellow with fronto-orbital plate dark brown; first flagellomere brownish to brown with dorsum darker and base yellowish (paler on inner surface); entire antenna dark brown in USA specimens. Scutum with complete yellow lateral stripe. Scutellum and metanotum dark brown. Pleuron brown with sutures yellowish (widest on anepimeron). Calypter margin and hairs dark. Legs brown with femora apices light brown.

***Genitalia***: (Figs 645–651) Epandrium with single spine (sometimes two on one side). Surstylus without spine, completely fused to epandrium. Basiphallus sclerotised on dorsal and left lateral surfaces, and with narrow sclerotised extension on right lateral surface; left and right apical margins produced into narrow extensions. Hypophallus membranous. Mesophallus cylindrical, approximately as long as, but narrower than swollen section of ejaculatory duct. Paraphallus narrow basally but broadly expanded apically and with inner margin arched inwards and narrowly fused to ventral suture on mesophallus. Distiphallus 1/2 length of mesophallus, darker than mesophallus, and entirely bifid with halves cup-shaped, tapering to base and with several minute spinules on inner surface. Ejaculatory apodeme with narrow blade expanding from short stem; sperm pump with hemispherical sclerotisation.

***Variation***: Dorsal margin of anepisternum yellow in Canadian specimens. USA specimens with fore coxa yellow and scutellum sometimes mostly to entirely dark brown.

###### Host.

Violaceae – *Viola*.

###### Distribution.

**Canada**: AB. **USA**: MD, MS, NC, NY, OH, PA.

###### Type material.

***Holotype*: USA. MS**: Washington Co., Leland, emerged 4.vi.1979, G. McMinn (1♂, USNM).

###### Additional material examined.

**USA. OH**: Delaware Co., Sunbury, Monkey Hollow Rd., 17.ix.2014, em. 8.x.2014, C.S. Eiseman, ex *Violapubescens*, #CSE1395, CNC384890 (1♀, CNC), em. 23.iii.2015, #CSE1482, CNC654470 (1♂, CNC). Also **s**ee Lonsdale (2017).

##### Metopomyza

Taxon classificationAnimaliaDipteraPhytomyzinae

Enderlein

Metopomyza Enderlein, 1936a: 180. Type species: AgromyzaflavonotaHaliday 1833, by monotypy. Spencer and Steyskal 1986b: 161; Zlobin 1995: 143, 2002b: 247.

*Metopomyza*, defined and treated in reviews by Zlobin (1995, 2002b), is a small Holarctic genus hypothesized to be an ancient offshoot of *Phytoliriomyza* that specialises on monocots (Spencer 1976a, 1990). The dense arrangement of tubercle-like setae on the epandrium and the morphology of the hypandrial complex would certainly seem to support this notion, but similarities to the Palaearctic *Selachops* are also numerous and require further examination. *Metopomyza* superficially resembles *Liriomyza* in having a yellow scutellum (uncommonly entirely dark) and a dark shiny scutum, but the head is darkly pigmented, at least laterally along the fronto-orbital plate, and the fronto-orbital plate is relatively pronounced and well-sclerotised, reaching (or nearly reaching) the lateral corner of the ocellar triangle to form the outline of an “M” along the posterior margin of the frons. Internally, the epandrium has two comb-like bands of tubercle-like setae. The posterior larval spiracles have minute spherical bulbs on long stalks (Zlobin 1995).

Six species of *Metopomyza* are known from the Nearctic. Only *M.interfrontalis* (Melander) is known from the Delmarva states.

#### Species description

##### Metopomyza interfrontalis

Taxon classificationAnimaliaDipteraPhytomyzinae

(Melander)

Figs 118 , 119 , 653–659

Agromyza interfrontalis Melander, 1913: 263.
Liriomyza xanthaspida Hendel, 1920: 144. Spencer 1981 [synonymy].
Liriomyza (Haplomyza) xanthaspida . Hendel, 1931: 263.
Liriomyza interfrontalis . Frick, 1952: 403.
Metopomyza interfrontalis . Frick, 1959: 412. Spencer 1969: 198, 1981: 333; Spencer and Steyskal 1986b: 161; Zlobin 1995: 152.

###### Description

**(Figs 118, 119, 653).** Wing length 1.4–1.8 mm (♂), 1.8–2.2 mm (♀). Length of ultimate section of vein M4 divided by penultimate section: 2.0–3.5. Costa ending at vein M (ending at R_4+5_ in some *Metopomyza*). Eye height divided by gena height: 3.1–4.0. First flagellomere slightly longer than high with anterodistal margin pointed, with point reduced to pronounced. Ocellar triangle rounded, barely larger than tubercle. Cheek weakly developed. Thorax with light pruinosity, but usually more matt; venter of pleuron shiny.

***Chaetotaxy***: Two to three ori; two ors; decreasing in size anteriorly. Orbital setulae well-developed, densely clustered anteriorly. Postocellar and ocellar setae slender, as long as ors. Three dorsocentrals, decreasing in length anteriorly, sometimes with much smaller fourth sutural to presutural dorsocentral. Acrostichal setulae in four irregular rows.

***Colouration***: Head brown with face (shiny medially) slightly darker, clypeus dark brown, and postgena and gena light brown with lower margin of gena dark and shiny; frons soft and yellowish brown to light brown with fronto-orbital plate (narrowing anteriorly), posterior margin and ocellar triangle dark brown. Scutum dark brown with wide yellow stripe on scutellum, and with notopleuron, postpronotum and supra-alar region slightly paler. Calypter hairs light brown, margin narrowly light brown. Legs dark brown with apices of femora yellow with spots on mid and hind femora sometimes reduced. Abdomen brown, tergites 1–5 uncommonly yellow laterally.

***Genitalia***: (Figs 654–659) Epandrium slightly produced and narrowed ventrally; with numerous tubercle-like setae on inner-medial ridge and along inner-distal margin, forming two dense, irregular comb-like rows; posterior margin ventral to cerci with several tubercle-like setae placed irregularly. Cercus very narrow and slender, with few short setae. Surstylus positioned and directed anteriorly, apex broadly rounded, free from epandrium; with several stout apical setae and one tubercle-like seta. Subepandrial sclerite broad and plate-like with dorsal region thicker and with one pair of short, ill-defined lateral arms, venter shallowly bilobed medially. Hypandrial lobe well-developed, with one seta; with pronounced anterobasal extension. Postgonite rounded apically, bare, with several sockets anteriorly, posteriorly split. Phallophorus slightly elongate and narrow. Basiphallus flat, dorsally confluent with phallophorus, apically split. Hypophallus broad, flat; lateral margins sclerotised and with inner-medial process. Paraphalli forming broad membranous leaf-like plates. Mesophallus cylindrical, constricted medially, length 2 × width. Distiphallus bifid with weak connection between halves and apex flared; sclerotised medially; slightly longer than basiphallus. Ejaculatory apodeme with abbreviated base, narrow stem that continues onto pale blade as sublateral rib; blade marginally striated; sperm pump clear.

###### Host.

Unknown – possibly Cyperaceae or Poaceae (Spencer and Steyskal 1986b).

###### Distribution.

**Canada**: AB, BC, MB, NL, NT, NU, ON, QC, YT*. **USA**: AK, CA, CO*, CT*, ID*, IL, KS, MA*, MI, MO*, NH*, NY*, OR, TX, VA, WA. Europe. Russia.

###### Type material.

***Holotype* [*interfrontalis*]: USA: DC**: Washington (1♀, USNM).

***Syntypes* [*xanthaspida*]: Germany**: “Germ.” (1♂ 1♀, NMW). [Not examined]

###### Additional material examined.

**Canada. AB**: Bilby, 11.vi.1924, O. Bryant (1♀, USNM), **BC**: Ketchum L., 58°22'N, 131°45'W, 23.viii.1960, W.W. Moss, CNC391602 (1♂, CNC), Kinbasket Lake, 13.vi.2008, Cooper Beauchesne and Assoc. Ltd., CNC391604 (1♂, CNC), Toad R. Lodge, Mi422, Alaska Hwy, 1371 m, 20.vii.1959, E.E. MacDougall, CNC481025 (1♀, CNC), **MB**: Aweme, 19.vi.1917, N. Criddle, CNC391576 (1♂, CNC), 24.viii.1917, CNC391580 (1♀, CNC), Churchill, 58°46'N, 94°10'W, 17.viii.1952, J.G. Chillcott, Ecological data f-C, CNC_Diptera59194 (1 ex, CNC), Farnworth Lake, near Churchill, 58°41'N, 94°3'W, 26.vi.1952, J.G. Chillcott, Ecological data F-B, CNC_Diptera97098 (1♂, CNC), Fort Churchill, 58°46'N, 94°10'W, 10.vi.1952, J.G. Chillcott, Ecological data T-B 12, CNC_Diptera59199 (1 ex, CNC), 11.vi.1952, Ecological data F-B 12, CNC_Diptera97094 (1 ex, CNC), 11.vii.1952, Ecological data F-D 21, F-E 21, CNC_Diptera59201, CNC_Diptera97097, CNC_Diptera59202 (1♂ 2 ex, CNC), 17.vi.1952, Ecological data F-B 12, T-D 18, CNC_Diptera97088, CNC_Diptera97090, CNC_Diptera97095, CNC_Diptera59195–59197 (7 ex, CNC), 23.vi.1952, Ecological data T-G 11, CNC_Diptera97091 (1 ex, CNC), 24.vi.1952, Ecological data T-C 18, CNC_Diptera59200, CNC_Diptera97092 (2 ex, CNC), 28.vi.1952, Ecological data F-B 12, F-B 12, CNC_Diptera97087, CNC_Diptera97089, CNC_Diptera59193 (3 ex, CNC), 5.viii.1952, Ecological data F-B 12, CNC_Diptera97095 (1 ex, CNC), 15.vii.1949, L.A. Miller, CNC_Diptera97093 (1 ex, CNC), Mile 505, Hudson Bay Ry., J.G. Chillcott, 13.v.1952, Biological data F-D, CNC391575 (1♂, CNC), **NL**: Labrador, Cartwright, 26.vi.1955, E.E. Sterns, CNC391569 (1♂, CNC), Jack’s Pond, 75’, 19.vi.1961, C.P. Alexander (1♂, USNM), Terra Nova N.P., 6.vii.1961, C.P. Alexander (1♂, USNM), Gander, 17.vi.1961, C.P. Alexander (1♂, USNM), Lomond, 12.vi.1961, C.P. Alexander (1♀, USNM), **NT**: Salmita Mines, 64°5'N, 111°15'W, 8.vii.1953, J.G. Chillcott, CNC391598 (1♂, CNC), Yellowknife, 8.vi.1953, J.G. Chillcott, CNC391599, CNC391600 (2♂, CNC), **NS**: Smith’s Cave, 6.viii.1925, A. Gibson, CNC391581 (1♀, CNC), **NU**: N.W.T., Muskox Lake, 64°45'N, 108°10'W, 5.vi.1953, J.G. Chillcott, CNC391596, CNC391597 (2♂, CNC), 5.vi.1953, CNC391585 (1♀, CNC), **ON**: Britannia, 1.vi.1948, G.E. Shewell, CNC391582 (1♀, CNC), Mer Bleue, 23.v.2015, O. Lonsdale, CNC440465(1♀, CNC), Normandale, 42°42'N, 80°19'W, 2.vi.1956, J.R. Vockeroth, CNC391574 (1♂, CNC), 24.v.1956, CNC391573(1♂, CNC), Ottawa, 17.vi.1955, J.G. Chillcott, CNC391571(1♂, CNC), 6.vi.1958, CNC391572(1♂, CNC), 20.vii.1954, J.R. Vockeroth, CNC391586 (1♀, CNC), 20.vii.1959, CNC391570 (1♂, CNC), Perth Road, 28.vii.1964, J.R. Vockeroth, CNC391603 (1♂, CNC), **QC**: Fort Chimo, 2.vii.1954, W.R. Richards, CNC391583 (1♀, CNC), Mi139, Rte 58, La Verendrye Prov. Pk., 28.vi.1965, D.M. Wood, CNC391567 (1♂, CNC), Mistassini, Mistassini Post, 50°25'N, 73°53'W, 7.vii.1956, J.R. McGillis, CNC391584 (1♀, CNC), Port Chimo, 2.vii.1954, W.R. Richards, CNC391568 (1♂, CNC), **YT**: North Fork Pass, Ogilvie Mts., 18.vi.1962, R.E. Leech, CNC391590 (1♀, CNC), Otter Lake, 62°30'N, 130°25'W, 17.vii.1960, J.E.H. Martin, CNC391587 (1♀, CNC). **Sweden.** T. Ipm., Maunu, starr på torvmosse [=sedge peat moss], nr 64, CNC391605, CNC391606 (1♂ 1♀, CNC). **USA. AK**: Unalakleet, 19.vii.1961, B.S. Heming, CNC391589 (1♀, CNC), 22.vi.1961, R. Madge, CNC391577, CNC391595 (2♂, CNC), 24.vi.1961, CNC391578, CNC391579, CNC391601 (3♂, CNC), Umiat, 8.vii.1959, R. Madge, CNC391591 (1♀, CNC), **CO**: Nederland, 3mi N, 2.vii.1961, J.G. Chillcott, CNC391588 (1♀, CNC), **CT**: Putnam Park, 18.vii.1939, A.L. Melander (1♂ 1♀, USNM), **ID**: Chateolet, viii.1915, A.L. Melander (1♂, USNM), **IL**:Savanna, 9.vii.1917, J.M. Aldrich (1♂, USNM), Champaign Co., 12.vii.1925, M.W. Shackleford, CNC391592 (1♀, CNC), 19.vii.1925, CNC391593 (1♀, CNC), 26.vii.1925, CNC391594 (1♀, CNC), **KS**: Manhattan, 9.vi.1934, C.W. Sabrosky, **MA**: Bedford, 20.vii.1961, swamp, W.W. Wirth (1♂, USNM), Concord, 27.vii.1961, marshy pond, W.W. Wirth (1♂ 2♀, USNM), **MI**: St. Joseph, 30.v.1938, C.W. Sabrosky (2♀, USNM), **MO**: Columbia, 7.viii.1968, Malaise, F.D. Parker (1♂, USNM), Boone Co., Columbia, 16–31.vii.1968, Malaise trap, F.D. Parker (1♂, USNM), **NH**: Stinson Lake, 23.vii.1961, W.W. Wirth (1?, USNM), **NY**: Allegany State park, 28.v-3.vi.1963, stream margin, W.W. Wirth (1♂ 2♀, USNM), Chautauqua Co., S. Dayton, 1.vi.1963, marsh area, W.W. Wirth (1♂, USNM), **OR**: Lane Co., 8mi S Florence, 29.vi.1971, G. Steyskal (1♂, USNM), **TX**: Sonora Exp. Sta., 23.iii.1955, W.W. Wirth (1♂, USNM), **VA**: Dead Run, 10.vi.1922, W.L. McAtee (1♀, USNM), Chain Bridge, 10.ix.1922, J.R. Malloch (1♀, USNM), Montgomery Co., Pandapas Pond, 37°16'30N, 80°28'00W, 14.v.2001, O. Lonsdale (1♀, USNM), **WA**: Mt. Consititution, Orcas Id., 7.vii.1905, J.M. Aldrich (1♀, USNM).

##### Nemorimyza

Taxon classificationAnimaliaDipteraPhytomyzinae

Frey

Nemorimyza Frey, 1946: 46 [as subgenus of Dizygomyza]. Type species: Agromyzaposticata Meigen, 1830, by monotypy. Frick 1959: 377 [as subgenus of Phytobia]; Nowakowski 1962: 97 [as genus]. Spencer 1986b: 87; Zlobin 1996: 273.
Annimyzella Spencer, 1981: 144 [as subgenus of Amauromyza]. Type species: Agromyzamaculosa Malloch, 1913. Zlobin 1996 [synonymy].

*Nemorimyza* is a robust-bodied genus of Phytomyzinae that closely resembles *Phytobia* in its size and semi-circular, pale, silvery lunule (pale or dark in *Phytobia*), strong setae with the prescutellar acrostichals present, reclinate orbital setulae, and in having the costa extend to M1 (R_4+5_ in some *Phytobia*) and the apex of the wing between the ends of veins R_4+5_ and M1. Differences from other genera are few, however, making this a difficult taxon to diagnose. Zlobin (1996) was the most recent to thoroughly redefine the genus, and noted that the epandrium has a characteristic morphology, and the surstylus is free from the epandrium, angled posteroventrally and articulates with the subepandrial sclerite. Most species also have brown markings on the halter (Chen and Wang 2008) and some have a small mediolateral seta on the fore tibia.

Within the genus, hosts are only known for two widespread species that also happen to occur in the Delmarva states. Both *Nemorimyzamaculosa* and *N.posticata* are leaf-miners of Asteraceae.

### ﻿Key to the Delmarva species of *Nemorimyza*

**Table:** 

1	Eye height divided by gena height 6.4–8.4. Three subequal ori. Four dorsocentral setae. Fore tibia without mediolateral seta. Halter with large brown anteroventral spot. Male abdomen entirely dark brown	***N.maculosa* (Malloch)**
–	Eye height divided by gena height 10.9–17.2 (Fig. 121). Two ori with anterior seta smaller. Three dorsocentral setae, but sometimes with enlarged setula anterior to third seta. Fore tibia with small mediolateral seta. Halter entirely white. Male abdomen dirty white past tergite 3	***N.posticata* (Meigen)**

#### Species descriptions

##### Nemorimyza maculosa

Taxon classificationAnimaliaDipteraPhytomyzinae

(Malloch)

Figs 660–666

Agromyza setosa Loew, 1869. Misidentification. Coquillett 1898: 78.
Agromyza maculosa Malloch, 1913: 302. Frost 1924: 45; Malloch 1934: 476.
Agromyza guaranitica Brèthes, 1920: 283. Spencer 1963 [synonymy].
Dizygomyza maculosa . Blanchard, 1938: 358.
Phytobia (Amauromyza) maculosa . Frick, 1952a: 393, 1957: 393, 1959: 378; Spencer 1963: 336.
Amauromyza maculosa . Spencer, 1967: 8.
Amauromyza (Annimyzella) maculosa . Spencer, 1981: 144; Spencer and Steyskal 1986b: 80.
Nemorimyza maculosa . Zlobin, 1996: 275; Martinez and Etienne 2002: 41; Benavent-Corai et al. 2005: 32; Scheffer and Lonsdale 2018: 87; Eiseman and Lonsdale 2018: 62; Monteiro et al. 2019: 175.

###### Description.

Wing length 2.2–2.3 mm (♂), 2.2–3.1 mm (♀). Length of ultimate section of vein M4 divided by penultimate section: 0.6–0.8. Eye height divided by gena height: 6.4–8.4. First flagellomere rounded, slightly higher than long, hairs slightly longer and denser along distal margin. Gena narrow, strongly angled upwards; cheek very narrow. Fronto-orbital plate slightly to moderately distinct, extending slightly medially to base of fronto-orbitals, widest at level of posterior ors. Lunule semi-circular. Apex of wing between R_4+5_ and M1.

***Chaetotaxy***: Three ori; two ors. Ocellar and postocellar setae finer and slightly shorter than fronto-orbitals. Ocellar setulae reclinate, in one row. Four dorsocentrals, one presutural, decreasing in length anteriorly. Acrostichal seta short but well-developed. Six rows of acrostichal setulae. Fore tibia without lateromedial seta. Mid tibia with two strong lateromedial setae.

***Colouration***: Body mostly dark brown to blackish; lunule brownish orange to yellowish with silvery pruinosity; antenna and frons between fronto-orbital plates sometimes slightly paler; gena sometimes paler or reddish, ventral margin with dark brown strip; postpronotum and notopleuron sometimes with small, limited orangish mottling; halter yellow; calypter entirely white. Notum subshiny.

***Genitalia***: (Figs 660–666) External genialia, subepandrial sclerite, and hypandrium as described for *N.posticata*. Postgonite with anterior 1/2 relatively short and stout with apex cleft and single seta shifted anteriorly; posterior 1/2 pale and split. Phallus strongly twisted to right past phallophorus. Halves of basiphallus narrow, flat and curved ventrally around mesophallus at apex; left 1/2 with broad mediobasal plate-like extension reaching venter of shaft. Hypophallus broad and plate-like with lateral margin more thickly sclerotised and anterior surface with one pair of semi-circular fins medially. Paraphallus relatively dark, U-shaped, and wrapping around sides of mesophallus; paler and united ventromedially. Distiphallus with broad, dark, basal section that is confluent with mesophallus and ventromedially strengthened with longitudinal bands; apically with one pair of pale, diverging tubules that are ca. as long as basal section and appear to be haired, although surface is continuous. Mesophallus subovate, nearly as large as basal section of distiphallus, enclosing swollen apical section of ejaculatory duct; with large ventral fossa. Ejaculatory apodeme with wide asymmetrical base, stem narrow with apex grading into narrow, paler blade; sperm pump sclerotised along venter.

###### Host.

Asteraceae – *Acanthospermum*, *Ageratum*, *Arctium*, *Artemisia*, *Aster*, *Baccharis*, *Bellis*, *Bidens*, *Calendula*, *Chromolaena*, *Chrysanthemum*, *Conyza*, *Cynara*, *Dahlia*, *Dendranthema*, *Emilia*, *Erechtites*, *Erigeron*, *Eupatorium*, *Gaillardia*, *Gamochaeta*, *Grindelia*, *Helenium*, *Helianthus*, *Lactuca*, *Melanthera*, *Mikania*, *Packera*, *Senecio*, *Solidago*, *Sonchus*, *Synedrella*, *Tagetes*, *Taraxacum*, *Zinnia* (Benavent-Corai et al. 2005; Diaz et al. 2015; Eiseman and Lonsdale 2018).

###### Distribution.

**Canada**: ON (Spencer, 1981). **USA**: widespread in United States, including Hawaii, but not Alaska. Argentina, Bahamas, Barbados, Bermuda, Bolivia, Brazil, Chile, Colombia, Costa Rica, Cuba, Guadeloupe, Grand Cayman, Guyana, Martinique, Mexico (Valenzuela-Escoboza et al. 2017), Peru, Puerto Rico, Dominican Republic, Saint-Martin, Trinidad, Uruguay, Venezuela (Martinez and Etienne 2002). Introduced to Madeira and Canary Islands (Černý et al. 2018).

###### Type material.

***Holotype* [*maculosa*]: USA. NY**: Jamaica, x.1986, bred from chrysanthemum leaves, Cat. No. 15641 (1♀, USNM). [Not examined]

***Syntype* [*guaranitica*]: Brazil.** Rio Grande do Sul, larvae mining leaves of cultivated Chrysanthemum [type information not given]. [Not examined]

###### Material examined.

**Canada. ON**: Ottawa, 16.vii.1976, P. Dang, leaf mine in Aster, CNC480518 (6 puparia [gel capsule], CNC), St. Lawrence Is. Nat. Park, McDonald Is., 20.viii.1976, W. Reid, Code 4472-B, CNC480517 (1♂, CNC). **Chile.** Elqui: Port Tres Cruces, Coquimbo, 30.x.1957, L.E. Pena, CNC480520 (1♀, CNC), Hda. Illapel, Coquimbo, 600–1000 m, 24–25.x.1954, L. Pena, CNC480196 (1♂, CNC). **USA. FL**: Miami-Dade Co., Redlands, 25°31'N, 80°30'W, 7.i.2012, R. Diaz and J. McClurg, ex leaves of *Mikaniamicrantha* CNC480525–480530 (2♂, 4♀, CNC), Palm Beach Co., Boynton Beach, 2.i.2016, T.S. Feldman, *Emilia* em. 4.i.2016, #CSE2227, CNC654342–654345 (2♂ 1♀ 1puparium, CNC), **HI**: Oahu, Honolulu, 0–121 m, J.R. Vockeroth, ex blotch mine in *Bidenspilosa* leaf, em. 26.ix.1966, CNC480522, CNC480521 (1♂ 1♀, CNC), **MA**: Worcester Co., Sturbridge, 42°2'28.84"N, 72°5'32.47"W, 5.vii.2016, C.S. Eiseman, *Erechtiteshieraciifolia*, em. 23–24.vii.2016, #CSE2797, CNC654276- 654289 (7♂ 6♀ 1puparium, CNC), Nantucket Co., Nantucket, Little Sesachacha Pond, 2.ix.2012, J.A. Blyth, ex *Erechtiteshieracifolia* em. 3.x.2012, #CSE109, CNC480523, CNC480524 (2♂, CNC), Nantucket Co., Nantucket, N. Liberty St., 41°17'N, 70°6'W, 7.viii.2012, C.S. Eiseman, ex *Erigeroncanadensis*, em. 23.viii.2012, #CSE43, CNC480555, CNC480556 (1♂ 1♀, CNC), **NC**: Scotland Co., Laurinburg, St. Andrews University, 2.vi.2015, em. 15–16.vi.2015, T.S. Feldman, ex *Erechtiteshieraciifolia*, #CSE1622, CNC653950–653953 (3♀ 1 puparium, CNC), **OH**: Hocking Co., South Bloomingville, Deep Woods Farm, 6.viii.2016, C.S. Eiseman, *Erigeroncanadensis*, em. 24.viii.2016, #CSE2935, CNC654477 (1♂, CNC), Hocking Co., South Bloomingville, Deep Woods Farm, 6.viii.2016, C.S. Eiseman, *Erechtiteshieraciifolia* em. 26–29.viii.2016, #CSE2953, CNC638902 (1♀, CNC), **SC**: Hilton Head Is., 12.vii.1965, H.F. Howden, CNC480519 (1♀, CNC), **OK**: Payne Co., Mehan, 36°0'51.62"N, 96°59'48.28"W, 30.v.2016, em. 23.vi.2016, M.W. Palmer, *Grindeliasquarrosa*, #CSE2615, CNC634780, CNC634781 (2♀, CNC).

##### Nemorimyza posticata

Taxon classificationAnimaliaDipteraPhytomyzinae

(Meigen)

Figs 121–123 , 667–674

Agromyza posticata Meigen, 1930: 172. Malloch 1913: 308; Frost 1924: 50.
Agromyza virgauriae Kaltenbach, 1869: 195. Hendel 1920 [synonymy].
Agromyza virgaureae . Emendation. Kaltenbach 1874: 331.
Agromyza terminalis Coquillett, 1895: 318. Malloch 1913 [synonymy].
Agromyza taeniola Coquillett, 1904: 191. Malloch 1913 [synonymy].
Agromyza argenteolunulata Strobl, 1909: 294. Hendel 1920 [synonymy].
Agromyza parvicornis (Loew). Misidentification, in part. Melander 1913: 254.
Dizygomyza (Dendromyza) posticata . Hendel, 1931: 30.
Dizygomyza (Nemorimyza) posticata . Frey, 1946: 42.
Phytobia (Phytobia) posticata . Frick, 1957: 390.
Phytobia (Nemorimyza) posticata . Frick, 1959: 377.
Nemorimyza posticata . Nowakowski, 1962: 97; Spencer 1969: 161, 1976: 308, 1981: 162; Spencer and Steyskal 1986b: 87; Zlobin 1996: 275; Martinez and Etienne 2002: 40; Benavent-Corai et al. 2005: 32; Scheffer et al. 2007: 772; Mlynarek and Heard 2018: 885; Scheffer and Lonsdale 2018: 87; Eiseman and Lonsdale 2018 : 62.

###### Description

**(Figs 121–123).** Wing length 2.8–3.2 mm (♂), 2.9–3.5 mm (♀). Length of ultimate section of vein M4 divided by penultimate section: 0.6–0.9. Eye height divided by gena height: 10.9–17.2. First flagellomere small, rounded, distal region with slightly longer, denser hairs that medially form an ill-defined to discrete denser subovate patch (similar to some *Agromyza*). Fronto-orbital plate evenly narrow, reaching level of inner base of fronto-orbitals. Gena very narrow, strongly angled upwards; cheek very narrow. Lunule semi-circular. Apex of wing between R_4+5_ and M1. Body subshiny, less so on fronto-orbital plate.

***Chaetotaxy***: Two ori (anterior seta thinner, 2/3–3/5 length); two ors. Orbital setulae reclinate, in one row. Three strong dorsocentral setae postsuturally, decreasing in length anteriorly; enlarged setula sometimes close in front of anterior dorsocentral. Acrostichal seta strong. Acrostichal setulae in six rows. Fore tibia with weak lateromedial seta. Mid tibia with two strong lateromedial setae.

***Colouration***: Body mostly dark brown; lunule dirty yellow with silvery pruinosity; antenna and sometimes legs slightly paler brown; scutum slightly paler laterally; apex of fore femur and sometimes base of fore tibia usually narrowly yellow, and if so, mid and hind legs sometimes similarly pigmented; male abdomen dirty white dorsally past tergite 3 with tergite 4 browner and with ill-defined brown anteromedial spot, but sometimes tergite 4 mostly dark with only posterior margin whitish, or tergite 3 with whitish pigment posterolaterally. Wing veins yellow with costa and marginal section of veins brownish.

***Genitalia***: (Figs 667–674) Epandrium broad, shallow, free from surstylus. Surstylus apically rounded, appearing small and lobate externally, but continuing along inner surface of epandrium as elongate plate; mostly short setose with minute pale tubercles along inner margin. Cercus narrow, tapering apically. Subepandrial sclerite broad, dark, somewhat H-shaped with longer dorsal arms and ventromedial bar with ventral margin shallowly lobate and minutely textured; with one pair of very large, stout and striated ventral sublateral setae. Hypandrium subtriangular with irregular apex, inner lobe well-developed and setose, extending along outer margins of postgonite. Postgonite bare; well-developed with apex cleft and posterior region paler and ill-defined with arm nearly separate. Phallus strongly twisted to right past phallophorus. Halves of basiphallus strongly curved inwards at apex, which is lobate, weakly joined to remainder of sclerite, and with distal margin thick and dark; left sclerite subquadrate, wider than long, wrapped around venter of shaft; base of right 1/2 wider and with inner margin thicker and darker. Hypophallus broad, plate-like and with inner-medial emargination. Paraphallus forming irregular dorsal and lateral complex around base of meso/distiphallus. Mesophallus somewhat pear-shaped, fusing to ventromedial surface of distiphallus, with one pair of ventromedial fossae (basal fossa larger). Distiphallus with broad basal section that is slightly compressed dorsoventrally (more so towards base), and with large dorsomedial plate emerging before darker, shallower shelf supporting numerous minute spicules; apically with U-shaped arch directed distally that supports fringe of hairs that continue dorsally along a flat, elongate dorsoapical process to form a ring. Ejaculatory apodeme with wide asymmetrical base and short, ill-defined stem grading into small, pale blade; sperm pump with irregular, somewhat patchy sclerotisation on venter.

###### Host.

Asteraceae – *Aster*, *Baccharis*, *Buphthalmum*, *Doellingeria*, *Elephantopus*, *Erechtites* (?), *Eurybia* (leaf mines), *Euthamia*, *Helianthus*, *Oclemena*, *Polymnia*, *Solidago*, *Symphyotrichum*, *Teucrium*, *Verbesina*, *Vernonia*. Lamiaceae – *Teucrium*. (Benavent-Corai et al. 2005; Eiseman and Lonsdale 2018; Ellis 2021).

###### Distribution.

**Canada**: AB, BC, MB*, ON, QC. **USA**: Likely present throughout (Spencer and Steyskal 1986b; Eiseman and Lonsdale 2018). Europe. Costa Rica, Venezuela (Martinez and Etienne 2002). Japan. Republic of Korea*.

###### Type material.

***Holotype* [*posticata*]: Germany.** Stolberg near Aachen (1♂, MNHN). [Not examined]

**Type [*virgaureae***]: [Type data unknown]. [Not examined]

***Syntypes* [*terminalis*]: USA. FL**: Welaka, 9.v.1894, C.W. Johnson (1♂, USNM), **PA**: Delaware County, 23.vii.1893, C.W. Johnson (1♂, USNM). [Not examined]

***Syntype* [*taeniola*]: USA.CA**: Mountains near Claremont, C.F. Baker Type No. 8040 (1♂, USNM). [Not examined]

***Holotype* [*argenteolunulata*]: Austria.** Admont (1♀, location unknown). [Not examined]

###### Material examined.

**Canada. AB**: Kananaskis, For. Exp. Sta. Seebe, 18.vii.1968, H.J. Teskey, CNC480551 (1♀, CNC), **MB**: Western Manitoba, Riding Mountain National Park, Moon Lake hiking trail, 50°53'N, 100°3'W, 662 m, 13.vii.2008, J. Cossey, N. Jeffery, J. Straka, CNC480531 (1♂, CNC), **NB**: Kouchibouguac N.P., 11.viii.1978, D.B. Lyons, Code-7432V, CNC480548 (1♀, CNC), 24.viii.1978, S.J. Miller, Code-7485W, CNC480549 (1♂, CNC), **NS**: Highland Rd., 15–20 mi N Hunter, 8.vii.1984, H.J. Teskey, CNC480552 (1♂, CNC), **ON**: Ottawa, 45°19'1.20"N, 75°43'12"W, 90 m, 13.vi.2016, J.E. O’Hara, Malaise trap, CNC629594 (1♂, CNC), Griffith, 10.vii.1991, J.R. Vockeroth, CNC480545 (1♂, CNC), Mer Bleu[e], 5mi E, 6.viii.1966, D.D. Munroe, Malaise trap, CNC480550 (1♀, CNC), Metcalfe, 29.vi.1994, B.E. Cooper, CNC480546 (1♂, CNC), Ottawa, Montfort Hosp. wood, 2.viii.1993, J.R. Vockeroth, aerial sweep, CNC480539 (1♂, CNC), 28.viii.1993, CNC480543 (1♀, CNC), 7.viii.1993, CNC480537 (1♀, CNC), 9.viii.1993, CNC480544 (1♀, CNC), Ottawa, damp second growth *Acer*-*Betula* wood, 19.ix.2000, J.R. Vockeroth, CNC480538 (1♀, CNC), 2.vii.2003, CNC480541, CNC480542 (1♂ 1♀, CNC), 29.vi.1994, CNC480540 (1♀, CNC), **QC**: Gatineau Co., Masham Twp., 10–20.vii.1974, D.M. Wood, CNC480547 (1♂, CNC). **Republic of Korea.** Gangwon-do: Chuncheon Nam-myeoh, Balsan, Hongchron River., 37°44'N, 127°35'E, 14.vi.2006, P. Tripotin, Malaise trap, large sandbar CNC475540, CNC475546 (2♂, CNC). **USA. FL**: Highlands Co., Sebring, Highlands Hammock State Park, 29.iii.2013, em. 30.iv.2013, C.S. Eiseman, ex *Elephantopuselatus*, #CSE395, CNC422917–422919 (2♀ 1 puparium, CNC), **IA**: Winneshiek Co., Cresco, Cold Water Creek Rd., 43°25'55.97"N, 92°00'34.78"W, 16.vii.2015, em. 5.viii.2015, C.S. Eiseman, ex *Solidagogigantea*, #CSE1962, CNC564703 (1♀, CNC), **MA**: Berkshire Co., Lenox, Mahanna Cobble, 12.vii.2016, C.S. Eiseman, *Euthamiagraminifolia*, em. 2.viii.2016, #CSE2836, CNC654234 (1♂, CNC), Franklin Co., Northfield, 276 Old Wendell Rd., 11.x.2013, C.S. Eiseman, ex. *Solidago*?, em. 27.iii.2014, #CSE1032, CNC384732 (1♂, CNC), Franklin Co., Northfield, 276 Old Wendell Rd., 30.ix.2013, C.S. Eiseman, ex. *Euthamiagraminifolia*, em. 26.iii.2014, #CSE1027, CNC384779, CNC384780 (1♂ 1♀, CNC), Franklin Co., Northfield, Crag Mountain, 16.x.2013, C.S. Eiseman, ex. *Oclemenaacuminata*, em. 29.iii.2014, #CSE1039, CNC384754–384757 (3♂ 1♀, CNC), Hampshire Co., Northampton Bikeway west of King St., 13.ix.2013, C.S. Eiseman, ex. *Symphyotrichumcordifolium*, em. 29.iii.2014, #CSE1041, CNC384734 (1♀, CNC), Hampshire Co., Northampton, Mineral Hills, 28.ix.2013, C.S. Eiseman, ex. *Symphyotrichumpuniceum*, em. 27.iii.2014, #CSE1031, CNC384792 (1♀, CNC), Nantucket Co., Nantucket, Dead Horse Valley, 6.viii.2012, C.S. Eiseman, ex *Solidagolatissimifolia*, em. 26.viii.2012, #CSE45, CNC480533 (1♂, CNC), Nantucket Co., Nantucket, Ice Pond Lot, 5.viii.2012, C.S. Eiseman, leaf mine in *Baccharishamilifolia*, em. 26.viii.2012, #CSE46, CNC480534 (1♀, CNC), Nantucket Co., Nantucket, UMass field station, 11.vi.2013, C.S. Eiseman, ex. *Solidagoaltissima*, em. 2.vii.2013, #CSE641, CNC384796 (1♀, CNC), Nantucket Co., Squam Swamp, 12.vi.2013, C.S. Eiseman, ex *Solidagolatissimifolia*, em. 3.vii.2013, #CSE646, CNC392660 (1♂, CNC), **ME**: Hancock Co., Penobscot, Battle Is., 18.vi.2012, C.S. Eiseman, ex *Solidagosempervirens* CNC480536 (1♂, CNC), **MO**: Williamsville, 14.vii.1969, Malaise trap, CNC480553, CNC480554 (1♂ 1♀, CNC), Franklin Co., Gray Summit, Shaw Nature Reserve, 1.vii.2015, em. 27.vii.2015, C.S. Eiseman, ex *Helianthus ?hirsutus*, #CSE1869, CNC564677 (1♂, CNC), **NC**: Scotland Co., Laurinburg, St. Andrews University, 15.v.2015, em. 7.vi.2015, T.S. Feldman, ex *Solidago*, #CSE1592, CNC564670 (1♂, CNC), **NY**: Essex Co., Upper Jay, nr. Bartlett Rd., 3.vi.2012, C.S. Eiseman, ex *Solidago* CNC480535 (1♂, CNC), **OH**: Delaware Co., Sunbury, Monkey Hollow Rd., 2.viii.2016, C.S. Eiseman, *Teucriumcanadense* em. 24.viii.2016, #CSE2934, CNC638881 (1♂, CNC), Adams Co., West Union, Eulett Center (4274 Waggoner Riffle Road), 30.vii.2016, C.S. Eiseman, *Vernonia*, em. 19.viii.2016, #CSE2917, CNC654484, CNC654485 (2♀, CNC), **SC**: Hilton Head Is., 12.vii.1965, H.F. Howden, CNC480532 (1♀, CNC), **OK**: Payne Co., Mehan, 13.x.2015, M.W. Palmer, *Elephantopuscarolinianus* em. 11.iv.2016, #CSE2363, CNC653989 (1♂, CNC), 24.viii.2015, M.W. Palmer, *Elephantopuscarolinianus* em. 14.ix.2015, #CSE2056, CNC654005 (1♂, CNC), **TN**: Davidson Co., Oak Hill, Radnor Lake, 22.viii.2017, em. 19–23.ix.2017, C.S. Eiseman, ex *Polymniacanadensis*, #CSE4291, CNC939947–939956 (4♂ 6♀, CNC), **TX**: Edwards Co., 1.3 miles NW of Campwood, 16.iii.2017, em. 30.iv.2017, C.S. Eiseman, ex *Verbesinavirginica*, #CSE3600, CNC941261 (1♀, CNC).

###### Comments.

*Nemorimyzaposticata* was found by Mlynarek and Heard (2018) to be a “complex of differentiated lineages “ that had “substantial and complex genetic structure”. Within this putative species, which as a whole exhibited a wide range of host and habitat preference, there was evidence for mostly host-associated, but also habitat-associated differentiation within and between genetic groups.

##### Phytobia

Taxon classificationAnimaliaDipteraPhytomyzinae

Lioy

Phytobia Lioy, 1864: 1313. Type species: AgromyzaerransMeigen 1830: 178, by monotypy. Spencer 1969: 101; Spencer and Steyskal 1986b: 73; Zlobin 2007a: 57, 2007b: 47, 2008a: 67, 2008b: 61.
Dendromyza Hendel, 1931: 22 [as subgenus of Dizygomyza]. Type species: AgromyzacarbonariaZetterstedt 1848: 2739, by original designation. Kangas 1935: 1 [as genus]. Frick 1952a [synonymy].
Liomyzina Enderlein, 1936a: 180 [nomen nudum – no type species designated]. Frick 1952a [synonymy].
Liomycina Enderlein, 1936b: 42 [attributes to Enderlein 1936a]. Type species: DomomyzalunulataHendel 1920: 124, by original designation. Syn. nov. [all previous synonymies of the genus name described in the paper are misspelled “Liomyzina”, that is, the nomen nudum given in Enderlein (1936a)]
Shizukoa Sasakawa, 1963: 38. Type species: ShizukoaseticopiaSasakawa 1963: 41, by original designation. Spencer 1965d [synonymy].

*Phytobia* is a genus of relatively large and stout-bodied species found in shrubs and trees as larvae. The larvae feed on young xylem, incorrectly referred to as “cambium” in much of the literature (Ylioja et al. 1998). These larvae are far less obvious to the collector compared to those in other genera feeding within leaves or even stems, and as such, biological data are incomplete for many species. This method of larval feeding is essential to the definition of the genus, as external adult morphology broadly overlaps with those of other genera, and has historically resulted in a broad fluxuation of it boundaries. Zlobin (2007a) suggested that *Phytobia* likely consisted of at least three separate genera, and while structures of the distiphallus do appear to support a deep split between several main lineages, the relative uniformity of external adult morphology, commonalities of the remainder of the male genitalia, and the complex, unique larval habit suggest otherwise, although little can be stated with any certainty until more is learned about this understudied group.

*Phytobia* can be easily confused for some *Agromyza*, but the latter always has an apical bend on vein R1 in Nearctic species, and the lateral margin of the first and second tergite has a conspicuous file. The relatively common *Nemorimyzaposticata* is also superficially similar, but in this species the fore tibia has a lateromedial seta, and the lunule is semi-circular and silvery (not shallow and yellow to brown with a light pruinosity); *N.maculosa* is also similar, but it has a dark spot on the halter.

Zlobin (2007a, 2007b, 2008a, 2008b) noted that nearly 100 species have been described in this genus, but this likely far underrepresents its actual diversity. Sixteen species are known in the United States and Canada, including Spencer and Steyskal’s (1986b) “Sp. n. Salix” from New York. Five species occur in the Delmarva states. Contrasting other groups of Agromyzidae, many species show a relative uniformity in genitalic morphology, leaving external characters such as colour, chaetotaxy and venation to predominate in keys and diagnoses.

### ﻿Key to the Delmarva *Phytobia*

**Table:** 

1	Distance between cross-veins ~ 1/2 length of vein dm-m, sometimes nearly 2/3 length (Fig. 690). Frons well-sclerotised and evenly rounded, shiny around base of fronto-orbitals. Clypeus stout and broad, width equal to length, length at midpoint longer than epistoma. Ejaculatory apodeme narrow and not much longer than wide. Hypophallus with one pair of flat, toothed sclerites. Distiphallus short with paired leaf-like membranous lobes medially (Figs 691, 692)	***P.waltoni* (Malloch)**
–	Distance between cross-veins at least as long as dm-m (Fig. 689), uncommonly slightly shorter. Frons sometimes soft anteriorly, evenly pruinose. Clypeus relatively narrow, width less than length, length at midpoint much shorter than epistoma. Ejaculatory apodeme large and fan-shaped. Hypophallus smooth. Distiphallus without lobes at midpoint; usually quite long	2
2	Palpus bright orange to yellow; at least parafacial, gena and anterior 1/3 of frons similarly bright orange to yellow, including at least part of fronto-orbital plate. Antenna bright orange, with anterior 1/2 of first flagellomere sometimes lightly infuscated	***P.betulivora* Spencer**
–	Palpus and frons usually brown; if palpus brownish orange to orange, frons dull orange on less than anterior 1/3 of frons, not including fronto-orbital plate, and parafacial and gena mostly grey/brown (some *P.amelanchieris*). First flagellomere brown, sometimes dark orange with distal 1/2–2/3 of first flagellomere lightly to darkly infuscated	3
3	Frons entirely dark brown with faint pruinosity. Antenna entirely and evenly dark brown, sometimes with orange tint. Distiphallus clavate, barely divided apically (Figs 680, 681)	***P.calyptrata* (Hendel)**
–	Frons dark brown with grey pruinosity, sometimes with anterior 1/3 or less faintly to distinctly orange. Antenna usually dark orange with distal region of first flagellomere infuscated to dark brown; uncommonly mostly dark brown with base (more so on inner surface) conspicuously orange. Distiphallus with one pair of elongate tubules	4
4	Four to six ori, uncommonly three on one or both sides; one ors. Sides of buccal cavity converging anteriorly. Base of distiphallus (seen laterally) slightly wider than tubules and distinctly darker (Fig. 677)	***P.amelanchieris* (Greene)**
–	Usually two ori (Figs 124, 125), sometimes three on one or both sides; two ors, with anterior ors sometimes weakly inclinate. Sides of buccal cavity parallel. Base of distiphallus (seen laterally) as slender as tubules; entire distiphallus dark brown, tubules not slightly clearer as above (Figs 687, 688)	***P.setosa* (Loew)**

#### Species descriptions

##### Phytobia amelanchieris

Taxon classificationAnimaliaDipteraPhytomyzinae

(Greene)

Figs 675–677

Agromyza amelanchieris Greene, 1917: 316.
Phytobia amelanchieris . Frick, 1952a: 390, 1959: 375; Spencer 1969: 102; Spencer and Steyskal 1986b: 78.

###### Description

**(Figs 675–677).** Wing length 3.0–4.4 mm (♂), 3.1–4.8 mm (♀). Length of ultimate section of vein M4 divided by penultimate section: 1.2–1.3. Eye height divided by gena height: 2.1–2.7.

As described for *P.setosa* except as follows: fronto-orbitals usually slender, four to six ori, one ors (difference in orientation pronounced); uncommonly with three ori on one or both sides, but if so, wing length always smaller and orange tint reduced to absent on frons and gena; orbital setulae erect; ocellar and postocellar setae longer than fronto-orbitals; acrostichal seta as long or longer than anterior dorsocentral; mid tibia with only two posteromedial setae; sides of buccal cavity converging anteriorly; length of epistoma usually equal to width of space between arms of clypeus; antenna always mostly orange with distal region of first flagellomere brown; cheek and parafacial thicker, more pronounced; frons grey, usually with only anteromedial region narrowly to more broadly orangish; epandrium brown to light brown with orange tint; base of distiphallus slightly wider than and distinctly darker than tubules (seen laterally). Paratype dissected by Spencer with long, cruciate, posterolateral “interfrontal” setae.

***Variation***: Some ON specimens with palpus dirty orange to orange, similar to *P.betulivora*, but orange colour dull, and pale region on frons restricted to anterior 1/3 or less. Female from WA with very long fronto-orbitals (6 ori) and large epistoma; with small fifth dorsocentral; pruinosity brownish, not grey; parafacial and fronto-orbital plate strongly swollen; eye height divided by gena 1.4.

###### Host.

Rosaceae– *Amelanchiercanadensis*.

###### Distribution.

**Canada**: AB*, BC, MB, NB*, NS*, ON, QC, SK. **USA**: CA*, KS*, MA, MI, NC, TN, VA, WA, WV.

###### Type material.

***Holotype***: **USA. WV**: French Creek, Quaintance No. 9444, F.E. Brooks (1♀, USNM; type No. 21063).

###### Paratypes examined.

**USA. MI**: Gladwin Co., 18.v.1946, R.R. Dreisbach (1♀,USNM), **WV**: French Creek, F.E. Brooks, Quaintance No. 9444 (3♂ 2♀, USNM).

###### Additional material examined.

**Canada. AB**: Waterton, 8.vi.1962, K.C. Hermann, CNC391688 (1♀, CNC), **BC**: Fife, 5mi E, 8.vi.1959, R.E. Leech, CNC391646 (1♀, CNC), Lethbridge, vi.1967, N.L.H. Krauss (1♀, USNM), **MB**: Ninette, bur oak community,7.v.1958, J.F. McAlpine, CNC391614, CNC391632, CNC391633, CNC391615 (2♂ 2♀, CNC), at margin of beaver pond, 9.v.1958, J.F. McAlpine, CNC391631, CNC391609, CNC391611, CNC391616 (3♂,1♀, CNC), **NB**: Kouchibouguac N.P., 20.v.1977, W.P. Hanley, Code – 5098B, CNC391682 (1♂, CNC), 22.v.1977, Hanley and Cooper, Code – 5111O, CNC391651 (1♀, CNC), 23.v.1977, B. Cooper, Code – 5113Q, CNC391648 (1♀, CNC), W.P. Hanley, Code – 5112P, CNC391649 (1♀, CNC), 25.v.1977, B. Cooper, Code – 5133K, CNC391650 (1♀, CNC), **NS**: CBHNt. Pk., Mackenzie Mtn., 400 m, birch and fir, 27.v.1984, B.E. Cooper, PG639848, CNC391680 (1♂, CNC), 28.v.1984, CNC391687 (1♀, CNC), 29.v.1984, CNC391681 (1♂, CNC), **ON**: Griffith, 7mi E, 1.vi.1985, B.E. Cooper, CNC391675 (1♂, CNC), 1.viii.1982, CNC391686 (1♀, CNC), Griffith, 15.v.1982, B.E. Cooper, CNC391647 (1♀, CNC), Lanark Co., N. Burgess Twp., 30.iv.1972, D.M. Wood, CNC391685 (2♂/♀, CNC), Mer Bleue Bog, Ottawa, 14.v.1965, CNC391676 (1♂, CNC), Metcalfe, 12.v.1982, B.E. Cooper, CNC391619 (1♂, CNC), 12.v.1983, CNC391663–391665, CNC391669, CNC391670, CNC391674 (5♂ 1♀, CNC), 14.v.1983, CNC391661, CNC391620, CNC391621, CNC391626, CNC391628, CNC391629 (6♂, CNC), 14.v.1994, CNC391652, CNC391653, CNC391655, CNC391656, CNC391671 (5♂, CNC), 17.v.1994, CNC391673 (1♂, CNC), 18.v.1983, CNC391624 (1♂, CNC), 25.v.1983, CNC391662 (1♂, CNC), 3.v.1983, CNC391666, CNC391668 (2♂, CNC), 3.v.1994, CNC391659, CNC391660 (2♂, CNC), 30.iv.1983, CNC391622, CNC391623, CNC391625, CNC391627 (4♂, CNC), 4.v.1994, CNC391658 (1♂, CNC), 6.v.1983, CNC391667 (1♂, CNC), 7.v.1994, CNC391654, CNC391657 (2♂, CNC), 9.v.1983, CNC391672 (1♂, CNC), Ottawa, 45°21'N, 75°45'W, city garden, 1–15.iv.2010, H. Goulet, Malaise trap, CNC391679 (1♂, CNC), Ottawa, 12.v.1965, B.V. Peterson, CNC391677 (1♂, CNC), Ottawa, damp second growth Acer-Betula wood, 29.v.1997, J.R. Vockeroth, CNC391678 (1♂, CNC), Rockport, 9.v.1961, C.H. Mann, CNC391612 (1♂, CNC), **QC**: Beechgrove, 15.v.1961, J.F. McAlpine, CNC391634, CNC391635 (2♀, CNC), 45°39'N, 76°8'W, 15.v.1961, J.R. Vockeroth, CNC391683, CNC391610, CNC391636-CNC391645 (2♂,10♀, CNC), Farnham, 5.vi.1963, J.R. Vockeroth, CNC391608 (1♂, CNC), Gatineau Pk., Harrington Lk., 30.v.1954, J.E.H. Martin, CNC391607 (1♂, CNC), **SK**: Sask. Landing, 50°39'N, 107°56'W, 25.v.1955, J.R. Vockeroth, CNC391618 (1♂, CNC), Saskatoon, 23.v.1940, A.R. Brooks, CNC391630 (1♀, CNC), 29.iv.1949, CNC391613 (1♂, CNC), 6.v.1949, CNC391617 (2♂/♀, CNC). **USA. CA**: Lake Tahoe, 20.vi.1963, A.L. Melander (1♂, USNM), **KS**: Lawrence, Nat. Hist. Res., 28.iv.1956, J.G. Chillcott, CNC391684, CNC391815, CNC391816 (3♂, CNC), **WA**: Deer Park, 5.v.1912, A.L. Melander (1♀, USNM).

###### Comments.

Greene (1917) discussed the larvae of *Phytobiaamelanchieris*, which were collected “Nearly full-grown” from the roots and the base of the trunk of Canadian serviceberry from early June to early July. Differences between this species and *P.setosa* are very slight and provided only tentatively here, and specimens with fewer, thicker fronto-orbitals can be difficult to diagnose; the boundaries of these two species should be re-evaluated following the collection of additional specimens.

##### Phytobia betulivora

Taxon classificationAnimaliaDipteraPhytomyzinae

Spencer

Fig. 678

Phytobia betulivora Spencer, 1969: 103. Spencer and Steyskal 1986b: 76.

###### Description.

Wing length 2.8–4.2 mm (♂), 3.8–4.5 mm (♀). Length of ultimate section of vein M4 divided by penultimate section: 1.0–1.4. Costa extending to vein M. Eye height divided by gena height: 2.2–3.1. Frons soft. Ocellar triangle indistinct. Parafacial wide, most pronounced dorsally, continuing as cheek on anterior 1/2 of gena. No space between antennal bases. Clypeus narrow, sides of buccal cavity slightly converging anteriorly and epistoma large and triangular (length not exceeding width). Space between cross-veins as long as, or slightly shorter than dm-m. Lunule sometimes sunken and obscured.

***Chaetotaxy***: Four or six ori; one ors. Postocellar and ocellar setae as developed as, or longer than fronto-orbitals. Orbital setulae erect to slightly reclinate, mostly in one irregular row but with some also between bases of fronto-orbitals. Vibrissa not much larger than genal setae, which are long and well-developed. Four strong dorsocentrals (one presutural). Small to weak seta medial to presutural supra-alar. One pair of prescutellar acrostichal setae as long as anterior dorsocentral. One or two posteromedial setae on mid tibia.

***Colouration***: Body dark brown with grey pruinosity that is especially dark on thorax, except as follows: antenna (sometimes excluding infuscation around base of arista), parafacial (sometimes tinged with brown/grey in part), face (brownish below antennae or only centrally) and gena (lower margin brown) orange to yellow, sometimes tinged with brown/grey; frons bright orange to yellow, at least on anterior 1/3–2/3 of frons including part of fronto-orbital plate, but sometimes orange region more extensive, at most with dark brownish grey region restricted to ocellar tubercle, paler region behind tubercle, and posterolateral corner of frons including base of vertical setae and base of posterior fronto-orbital; palpus orange; frons usually slightly darker around base of fronto-orbitals; scutellum, notopleuron, postpronotum and legs (more so on tarsi) with orange tint; base of tibiae and apices of femora sometimes faintly to more strongly orange; halter yellow; epandrium centrally or more widely reddish. Calypter margin brown to dark brown, sometimes slightly orange, with hairs dark brown.

***Genitalia***: (Fig. 678) As described for *P.setosa*, except as follows: right distolateral process of basiphallus slightly broader, but more weakly sclerotised along its length; mesophallus slightly stouter (especially apically) and more curved; base of distiphallus more bulbous and projecting ventrally; base of ejaculatory apodeme wider.

###### Hosts.

Betulaceae – *Betulanigra*, *Alnus* (Spencer 1969, 1990). Record of possible oviposition in Fraxinus (Oleaceae) (see below).

###### Distribution.

**Canada**: ON, QC*. **USA**: DC, IL, KS, MA*, NY, PA*, WA*. Czech Republic, Hungary (Papp and Černý 2016).

###### Type material.

***Holotype*: USA. DC**: Chain Bridge, 15.iv.1913, ex. cambium of *Betulanigra*, C.T. Greene (1♂ [with puparium], USNM).

**Paratype examined. Canada. ON**: Wilno, 16.v.1960, J.F. McAlpine, CNC Type No. 10460, CNC391689 (1♂, CNC).

###### Additional material examined.

**Canada. ON**: Mainfleet Bog, 8 km S Welland, 14–20.vi.1988, 021, pt. 2 – 1962 zone, A. Stirling (1♂, DEBU), Metcalfe, 4.vii.1985, B.E. Cooper, CNC391695 (1♂, CNC), Nr. Picton, 7.vii.1970, J.F. McAlpine, CNC391692 (1♀, CNC), Ottawa, between Carling and Driveway, 12.vi.1962, L.K. Smith, ovipositing in sucker stem of ash in CNR ditch, CNC391691 (1♀, CNC), St. Lawrence Is. Nat. Park, Grenadier I. Centre, 25.vi.1975, E. Signer, Malaise trap, Code – 231×-107, CNC391690 (1♀, CNC), **QC**: Duncan Lake, nr. Rupert, 21.v.1971, J.F. McAlpine, CNC391696, CNC391693 (2♂, CNC). **USA. DC**: Chain Bridge, 10719, Hopk. U.S., *Betulanigra*, C.T. Greene, reared 12.v.1913 (1♂ [with puparium], USNM), reared 22.iv.1913 (1♂ [with puparium], USNM), reared 18.iv.1913 (1♂ [with puparium], USNM), **IL**: St. Joseph, 3.v.1914, Salt Fork, J.M. Aldrich (1♀, USNM), **KS**: Lawrence, Nat. Hist. Res., 28.iv.1956, J.G. Chillcott, CNC391694 (1♂, CNC), **MA**: Boston, May, A.L. Melander (1♂, USNM), **NY**: Oneonta, 30.v.1935, H.K. Townes (1♀, USNM), **PA**: Hawley, 30.v.1937, A.L. Melander (1♂, USNM), **WA**: Oroville, A.L. Melander (1♀, USNM).

###### Comments.

Some Canadian specimens have the orange colour on the frons highly reduced, thereby resembling *Phytobiaamelanchieris*, but colour of the palpus, gena and parafacial will still differentiate the two.

##### Phytobia calyptrata

Taxon classificationAnimaliaDipteraPhytomyzinae

(Hendel)

Figs 679–682

Agromyza nigrisquama Malloch, 1916: 53 [preoccupied by Malloch (1914)].
Agromyza calyptrata Hendel, 1923: 145 [new name]. Frick 1952a: 372.
Phytobia (Trilobomyza) calyptrata . Frick, 1953: 70, 1959: 393.
Phytobia septentrionalis Spencer, 1969: 106. Spencer and Steyskal 1986b [synonymy].
Phytobia calyptrata . Spencer & Steyskal, 1986b: 270.

###### Description.

Wing length 2.9–3.5 mm (♂♀). Length of ultimate section of vein M4 divided by penultimate section: 1.1–1.2. Costa extending to vein M. Eye height divided by gena height: 3.1–7.0. Frons well-sclerotised and evenly rounded with fronto-orbital plate ill-defined and projecting. Parafacial projecting, venter less pronounced, continuing as narrow cheek on anterior 1/2 of gena; narrow, slightly widened and projecting dorsally. Lunule semi-circular, shallow. Space between antennae ~ 1/3 width of scape. Clypeus narrow and broadly rounded, buccal cavity subquadrate, and length of epistoma 0.2 × width or less. Space between cross-veins longer than length of dm-m, sometimes nearly 1.5 × length of dm-m.

***Chaetotaxy***: Two ori (sometimes three ori on one side); two ors. Postocellar and ocellar setae well-developed. Orbital setulae reclinate, in one irregular row. One presutural and three postsutural dorsocentrals; slightly decreasing in length anteriorly or with anterior two much shorter, closer in length to surrounding setulae. Small to weak seta medial to long presutural supra-alar. Acrostichal seta slightly thinner than, and nearly as long as fronto-orbitals. One posteromedial seta on mid tibia.

***Colouration***: Body predominantly dark brown with slight orange-reddish tint that is stronger on antenna, legs and venter of pleuron; body with light greyish brown pruinosity that is greyer and denser dorsally on head and thorax (shinier on ocellar tubercle and in posterolateral corner of frons); dorsum of thorax slightly, and frons sometimes much darker; lunule whitish to beige; parafacial sometimes whitish to beige medially (type specimens *septentrionalis*); legs brown with base of tibiae slightly yellowish; halter yellow; epandrium sometimes reddish. Calypter margin and hairs dark brown.

***Genitalia***: (Figs 679–682) Hypandrial lobe medially setose and plate-like, nearly separate. Postgonite deeply cleft and upcurved anteriorly. Basiphallus long and narrow, bifid on distal 1/3. Paraphallus sometimes evident as one pair of weak bars. Hypophallus sometimes visible as weak ventral plate. Mesophallus cylindrical with base rounded, slightly longer than wide, entirely fused to distiphallus. Distiphallus tubular and weakly sclerotised, constricted subbasally, widening distally with apex more abruptly expanded; sometimes appearing apically bifid.

***Variation***: NB female with lunule very shallow, nearly straight along dorsal margin, parafacial thicker and projecting, and dorsocentrals becoming only slightly shorter anteriorly.

###### Host.

Unknown – possibly *Salix* (Spencer and Steyskal 1986b).

###### Distribution.

**Canada**: NB*, ON, QC. **USA**: DC, IL, KS, MD, NY.

###### Type material.

***Holotype* [*nigrisquama*]: USA.** Illinois (1♀, INHS). [Not examined]

***Holotype* [*septentrionalis*]: Canada. QC**: Kingsmere, 12.v.1958, CNC Type No. 10389, J.G. Chillcott (1♂, CNC).

###### Paratype examined

[***septentrionalis*]: Canada. ON**: Simcoe, 20.v.1939, G.E. Shewell, CNC Type No. 10389, CNC391706 (1♂, CNC).

###### Additional material examined.

**Canada. NB**: Kouchibouguac N.P., 24.v.1977, B. Cooper, Code – 5132J, CNC391698 (1♀, CNC), **ON**: St. Lawrence Is. Nat. Park, McDonald Is., 7.viii.1979, W. Reed, Code 4353.M (1♂, CNC), St. Lawrence Is. Nat. Park, McDonald Is., 21.vii.1976, W. Reid, Code 4166-F, CNC391699 (1♀, CNC), 7.viii.1976, W. Reid, Code 4353-M, CNC391697 (1♂, CNC), Stratford, 15.viii.1959, D.H. Pengelley (7♀, DEBU), Midland, 12.v.1959, J.G. Chillcott, CNC391702 (1♂, CNC), 5.v.1959, CNC391700, CNC391701 (2♂, CNC), Ottawa, damp second growth Acer-Betula wood, 8.vii.2003, J.R. Vockeroth, CNC391705 (1♀, CNC), **QC**: Duncan Lake, nr. Rupert, 21.v.1971, J.F. McAlpine, CNC391703 (1♂, CNC), 29.v.1971, CNC391704 (1♀, CNC). **USA. MD**: Colesville, 24.vii.1974, W.W. Wirth (1♂, USNM).

##### Phytobia setosa

Taxon classificationAnimaliaDipteraPhytomyzinae

(Loew)

Figs 3 , 124 , 125 , 683–688 , 689

Agromyza setosa Loew, 1869: 45. Frick 1952a: 373.
Agromyza aceris Greene, 1917: 313. Frick 1952a: 390. Frick 1959 [synonymy not explicit].
Phytobia (Phytobia) setosa . Frick 1959: 376.
Phytobia setosa . Spencer 1969: 107; Spencer and Steyskal 1986b: 77.

###### Description

**(Figs 3, 124, 125, 689).** Wing length 3.4–4.6 mm (♂), 3.7–4.4 mm (♀). Length of ultimate section of vein M4 divided by penultimate section: 1.2–1.4. Costa extending to vein M. Eye height divided by gena height: 3.0–4.1. Frons soft if anterior margin orange. Space between antennae ~ 1/3–2/3 width of scape. Clypeus narrow, sides of buccal cavity parallel and length of epistoma 1/2 width. Space between cross-veins as long as dm-m.

***Chaetotaxy***: Two or three ori; two ors (anterior ors sometimes slightly inclinate). Orbital setulae reclinate, in a single irregular row. Postocellar and ocellar setae well-developed, subequal to fronto-orbitals. Small to weak seta medial to presutural supra-alar. Prescutellar acrostichal seta nearly as long as anterior dorsocentral. Two to three posteromedial setae on mid tibia.

***Colouration***: Body predominantly dark brown. Lunule orange. Frons slate-grey with posterolateral margin and anterior 1/3 or less sometimes orange; if frons between fronto-orbital plates partially orange, sides of face and gena orange to rusty with all or dorsal margin of gena grey and ventral margin brown. Antenna dark orange, distal margin of first flagellomere brownish (more so on outer face, particularly in individuals with entirely grey frons); uncommonly dark brown with base (more so on inner surface) conspicuously orange. Mid trochanter and coxa sometimes orange laterally. Halter yellow. Calypter margin and hairs dark brown. Abdomen with epandrium sometimes variably to entirely orange or reddish, brightest near base.

***Genitalia***: (Figs 683–688) Epandrium slightly produced ventrally. Surstylus separate from epandrium, small, heavily setose apically, directed inwards. Cercus narrow, setose. Hypandrium well-developed, with narrower, stout, rounded apex; inner lobe with several setae. Phallophorus small. Basiphallus narrowly sclerotised along dorsum, split apically with left process hook-like and right process abruptly paler on distal 1/2; halves flanking base of mesophallus. Hypophallus narrow, curved, longer than mesophallus. Mesophallus cylindrical with base rounded, slightly bent at midpoint, width ~ 1/3 length; dorsally fused to base of distiphallus. Distiphallus divided into one pair of long, slender tubules along most of length; evenly dark along length. Ejaculatory apodeme stout with rounded blade, short, apically widening stem and stout base; sperm pump with transverse sclerotised bar.

###### Hosts.

Sapindaceae – *Acerrubrum*, A.saccharum*.

###### Distribution.

**Canada**: NB*, NS*, ON, QC, SK*. **USA**: AZ*, CT*, DC, IA, IN, MA, MD, MI, NY, PA*, VA, WI, WV. Mexico (Durango).

###### Type material.

***Holotype*: [*setosa*]: USA.** District of Columbia (1♂, MCZ; type No. 13442).

***Holotype* [*aceris*]: USA. VA**: Falls Church, Chain Bridge, *Acerrubrum*, reared 26.iv.1916, “12973 Hopk US”, C.T. Greene (1♀ 1puparium, USNM; type No. 21062).

###### Paratypes examined

[***aceris*]: USA. VA**: Falls Church, Chain Bridge, *Acerrubrum*, reared 26.iv.1916, “12971-a Hopk US”, *Acer*, C.T. Greene (1♂ 1puparium, USNM), **WV**: French Creek, F.E. Brooks, Quaintance No. 9444 (1♀, USNM).

###### Additional material examined.

**Canada. NB**: Kouchibouguac N.P., 10.vii.1977, J.F. McAlpine, Code 6025S, CNC391749 (1 ex [abdomen missing], CNC), 11.vii.1977, Code – 6026T, CNC391753 (1♀, CNC), 12.vii.1977, Code – 6041I, CNC391754 (1♀, CNC), 13.vii.1977, Code – 6042J, CNC391755 (1♀, CNC), Code – 6039G, CNC391756 (1♀, CNC), 8.vii.1977, Code – 6021O, CNC391752 (1♀, CNC), 15.vi.1978, S.J. Miller, Code – 7066T, CNC391811 (1♀, CNC), 18.v.1977, B. Cooper, Code – 5065U, CNC391744 (1♂, CNC), 20.v.1977, Code – 5097A, CNC391750, CNC391812 (2♀, CNC), 19.v.1977, W.P. Hanley, Code – 5087Q, CNC391748 (1 ex [abdomen missing], CNC), Code – 5098B, CNC391745, CNC391751 (1♂ 1♀, CNC), 6.vii.1977, **NS**: CBHNt. Pk., Mackenzie Mtn., 400 m, birch and fir, 1.vi.1984, B.E. Cooper, PG639848, CNC391765 (1♂, CNC), 27.v.1984, CNC391767 (1♂, CNC), 28.v.1984, CNC391769, CNC391814 (1♂,1♀, CNC), 29.v.1984, CNC391768 (1♂, CNC), 30.v.1984, CNC391766 (1♂, CNC), 31.v.1984, CNC391770 (1♂, CNC), 7.vi.1984, CNC391813 (1♀, CNC), **ON**: Midland, 18.v.1976, J.T. Huber (1♂, DEBU), Guelph, 10.v.1982, A. John (1♂, DEBU), Essex Co., Windsor, ~ 1.5 km S Ojibway Prairie, 5–12.vi.2001, private prairie, Malaise, S. Paiero (1♂, USNM), Bell’s Cor., 1.v.1951, J.F. McAlpine, CNC391732 (1♂, CNC), Greenbush, at lights, 6.v.1999, R. Hainault, CNC391807 (1♀, CNC), Lanark Co., N. Burgess Twp., 15.v.1971, D.M. Wood, CNC391808–391810 (3♀, CNC), Marmora, 25.iv.1952, J.F. McAlpine, CNC391734 (1♂, CNC), 28.iv.1952, CNC391757 (1♀, CNC), 29.iv.1952, CNC391733 (1♂, CNC), Metcalfe, 12.v.1983, B.E. Cooper, CNC391785 (1♂, CNC), 14.v.1994, CNC391788, CNC391789 (2♂, CNC), 17.v.1994, CNC391786, CNC391787 (2♂, CNC), 7.v.1994, CNC391791 (1♂, CNC), 8.v.1994, CNC391790 (1♂, CNC), Midland, 5.v.1959, J.G. Chillcott, CNC391780 (1♂, CNC), Normandale, 42°42'N, 80°19'W, 22.v.1956, J.R. Vockeroth, CNC391730, CNC391731 (2♂, CNC), Ottawa, 9.v.1923, C.H. Curran, CNC391740 (1♂, CNC), Rockport, 9.v.1961, J.R. Vockeroth, CNC391781 (1♂, CNC), **QC**: Abbotsford, 22.v.1936, G.E. Shewell, CNC391758 (1♀, CNC), Beechgrove, 45°39'N, 76°8'W, 10.v.1962, J.R. Vockeroth, CNC391727–391729 (3♂, CNC), 15.v.1961, CNC391723 (1♂, CNC), 16.v.1962, CNC391724–391726, CNC391760 (3♂,1♀, CNC), Chelsea, 24.iv.1933, G.S. Walley, CNC391741 (2♂/♀, CNC), Duncan Lake, nr. Rupert, 21.v.1971, J.F. McAlpine, CNC391793, CNC391794, CNC391762–391764, CNC391802, CNC391805, CNC391806 (3♂ 5♀, CNC), 29.v.1971, CNC391792, CNC391795–391801, CNC391803 (9♀, CNC), 5.vi.1971, CNC391804 (1♀, CNC), Gatineau Pk., Harrington Lk., 31.v.1954, E.E. Sterns, CNC391759 (1♀, CNC), Gatineau Pk., Lac Philippe, 23.v.2011, O. Lonsdale, CNC391779 (1♂, CNC), Hull, 22.v.1923, R. Ozburn, CNC391720 (1♂, CNC), 26.iv.1929, C.H. Curran, CNC391707 (1♂, CNC), Kingsmere, 12.v.1958, J.G. Chillcott, CNC391721, CNC391722 (2♂, CNC), Lac Mondor, Ste. Flore, 21.v.1951, E.G. Munroe, CNC391746 (1♂, CNC), 5.v.1951, CNC391713, CNC391714 (2♂, CNC), 5.vi.1951, CNC391717 (1♂, CNC), 6.v.1951, CNC391708–391712, CNC391715, CNC391716, CNC391718 (8♂, CNC), Lac Philippe, 75°36'N, 76°0'W, 25.iv.1987, J.R. Vockeroth, CNC391773, CNC391777 (2♂, CNC), Mt. St. Hilaire, North Ck., 3–6.v.1982, B.M. Nelson, CNC391771, CNC391772, CNC391774–391776 (5♂, CNC), Old Chelsea, 17.v.1947, G.E. Shewell, CNC391735–391739 (5♂, CNC), 17.v.1990, J.R. Vockeroth, CNC391778 (1♂, CNC), Tetreau val, 20.v.1923, C.H. Curran, CNC391719 (1♂, CNC), **SK**: Sask. Landing, 50°39'N, 107°56'W, 25.v.1955, J.R. Vockeroth, CNC391761 (1♂, CNC). **USA. AZ**: Portal SW Res. Sta., 5–9.vi.1972, Malaise trap, W.W. Wirth (1♂, USNM), **CT**: Redding, 19.v.1934, A.L. Melander (1♂, USNM), **IN**: Lafayette, 1.iv.1918, J.M. Aldrich (1♂, USNM), **MA**: Petersham, 2.vi.1914, A.L. Melander (1♂, USNM), Woods Hole, 20–30.vi.1923, A.H. Sturtevant (1♀, USNM), New Bedford, 25.iv, A.L. Melander (1♀, USNM), Boston, A.L. Melander, May (1♂, USNM), June (1♀, USNM), **MD**: near Plummers Isl., 7.iv.1915, R.C. Shannon (1♂, USNM), Montgomery Co., Clarksburg, 20–22.v.1988, W.E. Steiner and J.M. Swearingen (1♂, USNM), Bethseda, 5.v.1969, G. Steyskal (2♂, USNM), Colesville, 21.iv.1977, W.W. Wirth (1♂, USNM), Bethseda, 27.iv.1969, W.W. Wirth (1♂, USNM), Prince Georges Co., 7.v.1977, J.F. Reinert (1♂, USNM), **MI**: Otsego Co., 26.iv.1941, R.R. Dreisbach (1♂, USNM), **NY**: Savage Rt. Pond, 3mi W Geneva, Knutson, pupa coll. 21.iii.1966, emerged 2.iv (1♂ [with puparium], USNM), W Nyack, 31.v.1936, A.L. Melander (2♂, USNM), Long Island, 17.ix.1988, R. Latham (1♂, USNM), Long Island, Orient., R. Latham, 6.viii.1952 (1♀, USNM), 4.ix.1962 (1♀, USNM), **PA**: Sullivan Co., Wyoming State For., 8 km NW Laporte, 6.v.1987, J.M. Cumming, CNC391782–391784 (3♂, CNC), **VA**: Fairfax Co., Great Falls Park, quarry, 38°59.1'N, 77°14.8'W, Malaise trap, D.R. Smith, 3–10.v.2007 (1♂ 1♀, USNM), 10–17.v.2007 (1♀, USNM), 24.iv-2.v.2007 (1♀, USNM), **WI**: Sawyer Co., 7.v.1964, J.B. Hanson, ex. sugar maple root (1♂ 1♀, USNM). **Location unknown.** ”Mel. It. / v.18.10”, CNC391742 (2♂/♀, CNC), 3-51Y / BRI-97 / 75-714, CNC391747 (1♂, CNC).

###### Comments.

Feeding on relatively widespread and abundant hosts, *Acerrubrum* and *A.saccharum*, *Phytobiasetosa* is encountered with relative frequency east of Manitoba and New Mexico and is often well-represented in collections. External and male genitalic morphology strongly resembles that of the sometimes similarly large *Phytobiaamelanchieris*, especially in those *P.setosa* with the anterior ors slightly inclinate, but *P.amelanchieris* often has more slender fronto-orbitals with the ori and ors always clearly differentiated, and the sides of the buccal cavity are slightly convergent anteriorly.

##### Phytobia waltoni

Taxon classificationAnimaliaDipteraPhytomyzinae

(Malloch)

Figs 690–692

Agromyza waltoni Malloch, 1913a: 303.
Phytobia (Phytobia) waltoni . Frick, 1952a: 391, 1959: 377.
Phytobia waltoni . Spencer, 1969: 109; Spencer and Steyskal 1986b: 272.

###### Description

**(Fig. 690).** Wing length 3.2–3.9 mm (♂), 3.6–4.0 mm (♀). Length of ultimate section of vein M4 divided by penultimate section: 1.1–1.5. Costa extending to vein M. Space between cross-veins ~ 1/2 length of dm-m, but sometimes nearly 2/3 length. Eye height divided by gena height: 3.2–3.6. Frons well-sclerotised; ocellar triangle indistinct. Space between antennae ~ 2/3–3/3 width of scape. Clypeus stout and broad, width equal to length, length at midpoint longer than epistoma. Epistoma shallow and broad. Frons shiny around base of fronto-orbitals (pruinose in other species).

***Chaetotaxy***: Three ori; two ors (inset). Postocellar seta as long as ors; ocellar seta slightly longer. Four dorsocentrals, one presutural, slightly decreasing in size anteriorly. One pair of strong prescutellar acrostichal setae, subequal to dorsocentrals. One usually indistinct posteromedial mid tibial seta.

***Colouration***: Head dark brown with lunule orange to brown. Thorax brown to dark brown with orange-reddish tint (paler on postpronotum and notopleuron, particularly on anterior margin, or with pale spot on either side of postpronotum) and brownish grey pruinosity; halter yellow. Calypter margin and hairs dark. Legs and abdomen brown to dark brown.

***Genitalia***: (Figs 691–692) Inner lobe of hypandrium broadly arched anteriorly with apex setulose. Postgonite narrow with apex broadly expanded dorsally and with one inner seta. Basiphallus relatively short with well-developed recurved subapical branch. Hypophallus with one pair of narrow, weakly pigmented and textured plate-like lobes with truncated, serrated apical margins. Paraphallus absent. Mesophallus narrow, ~ 1/2 length of distal section, broadly rounded basally, narrowed apically to point of fusion with bulbous base of distiphallus. Distiphallus split along most of length into two narrower tubules with flared apex; base surrounded by membranous fringe with paired leaf-like processes anteriorly. Ejaculatory apodeme short, blade reduced to stump; sperm pump with reduced ventral sclerotisation.

###### Host.

Unknown.

###### Distribution.

**Canada**: NB*, QC. **USA**: IA, NC, NH*, NY, SC, TN*.

###### Type material.

***Holotype*: USA. NY**: “Amer. Sept. Horv., 1907”, Adirondack, Long Lake (1♀, USNM; type No. 15572).

###### Additional material examined.

**Canada. NB**: Kouchibouguac N.P., 10.vii.1977, J.F. McAlpine, Code – 6025S, CNC391826 (1♂, CNC), 11.vii.1977, Code – 6026T, CNC391822, CNC391823 (2♂, CNC), 12.vii.1977, Code – 6040H, Code – 6041I, CNC391829, CNC391830, CNC391825 (3♂, CNC), 13.vii.1977, Code – 6042J, CNC391832–391824, CNC391827 (4♂, CNC), 6.vii.1977, Code – 6039G, CNC391831 (1♂, CNC), 9.vii.1977, Code – 6023Q, Code – 6023a, Code – 6024R, CNC391819–391821, CNC391828 (3♂ 1♀, CNC), 12.vii.1977, J.R. Vockeroth, CNC391836 (1♀, CNC), 12.vii.1978, S.J. Miller, Code – 7250V, CNC391835 (1♂, CNC), 22.vii.1978, Code – 7325S, CNC391834 (1♂, CNC), **QC**: Mt. Albert, 28.vii.1954, J.E.H. Martin, CNC391817, CNC391818 (2♀, CNC). **USA. NC**: Smokies, Andrews Bald, 9.vii.1941, A.L. Melander (16♂ 3♀, USNM), Great Smokies N.P., Clingmans Dome, A.L. Melander, 18.vii.1941 (5♂, USNM), 19.vii.1941 (1♂, USNM), Great Smokies N.P., “Newfnd Ridge”, 11.vii.1941, A.L. Melander (9♂ 2♀, USNM), Smokies, Forney Ridge, 26.vi.1941, A.L. Melander (1♂, USNM), **NH**: Lost River, vii.1931, A.L. Melander (1♂, USNM), Franconia Ntch, 8.vii.1931, A.L. Melander (1♂, USNM), **TN**: Smokies, Arch Rock, 28.vi.1941, A.L. Melander (1♂, USNM).

##### Phytoliriomyza

Taxon classificationAnimaliaDipteraPhytomyzinae

Hendel

Phytoliriomyza Hendel, 1931: 203 [as subgenus of Liriomyza]. Type species: AgromyzaperpusillaMeigen 1830: 181, by monotypy. Frick 1952a: 410, 1959: 413; Spencer 1969: 201; Spencer and Steyskal 1986b: 151.
Xyraeomyia Frick, 1952a: 412. Type species: XyraeomyiaconjuctimontisFrick 1952a: 413, by original designation. Spencer 1965 [synonymy].
Pteridomyza Nowakowski, 1962: 97. Type species: AgromyzahilarellaZetterstedt 1848: 2776, by original designation. von Tschirnhaus 1971 [synonymy].
Lemurimyza Spencer, 1965b: 26. Type species: LiriomyzaenormisSpencer 1963c: 114, by original designation. von Tschirnhaus 1971 [synonymy].
Nesomyza Spencer in Spencer and Stegmaier 1973: 190. Type species: Nesomyzafusculoides Spencer in Spencer and Stegmaier 1973: 190, by original designation. Spencer 1973b [synonymy].

Six species of *Phytoliriomyza* are recognised in the Delmarva states. Many are small and pale with a grey pruinosity that is visible on the darker sclerites, or are larger with a black first flagellomere, but *P.melampyga* is predominantly yellow and will be easily confused for some *Liriomyza*. The genus is very heterogenous and likely not monophyletic, but much additional study is required before the component lineages can be confidently divided and treated as separate entities.

### ﻿Key to the Delmarva *Phytoliriomyza*

**Table:** 

1	Three dorsocentral setae, strongly decreasing in size anteriorly, with anterior seta not much larger than surrounding setulae	***P.robiniae* (Valley)**
–	Four long dorsocentral setae	2
2	Orbital setulae proclinate. Eye minutely setulose. Sides of frons with light silvery pubescence (Fig. 126). Acrostichal setulae absent or only a few present anteriorly. Tibiae brown, fore coxa with brownish base. Apical surface of halter brownish (sometimes indistinct) (Fig. 127). Vein r-m near basal 1/3 of cell dm. Epandrium with one tubercle-like seta on distal margin of epandrium at most	3
–	Orbital setulae erect or absent. Eye bare. Sides of frons without reflective pubescence. Acrostichal setulae in four rows; if in two rows, these extending nearly to posterior margin of scutum. Tibiae yellow, fore coxa entirely yellow. Halter entirely white. Vein r-m at midpoint of cell dm or beyond. Epandrium with comb of setae below surstylus on distal margin	4
3	Posterior notopleural seta very thin and short. Mid and hind coxae usually brown, at least in part on hind coxa (Fig. 127). Epandrium without dark tubercle-like seta on distal margin (Fig. 694)	***P.arctica* (Lundbeck)**
–	Posterior notopleural seta as thick as anterior seta and not much shorter. Mid and hind coxae yellow. Epandrium with single dark tubercle-like seta on distal margin (Fig. 711)	***P.pilosella* Spencer**
4	Scutellum and posterior margin of scutum brown. Notum with greyish pruinosity. Gena 1/2 height of eye. Ultimate section of M4 ~ 2 × length of penultimate section. Wing length 1.5–1.8 mm. Abdomen brown. Phallus long, clear and curved at apex (Figs 721, 722)	***P.pulchella* Spencer**
–	Scutellum and posterior margin of scutum yellow (Fig. 128). Notum shiny. Gena 1/3 height of eye. Ultimate section of M4 nearly as long as penultimate section (~ 4/5 length). Wing length 2.0–2.5 mm. Abdomen predominantly yellow. Phallus strongly coiled if clear	5
5	First flagellomere yellow (Fig. 129). Two to three ori. Four rows of acrostichal setulae. Epandrium brown. Surstylus projecting, easily seen when viewed laterally; with comb of palisade-like setae (Figs 705–707). Basiphallus reduced to long, narrow, dark left lateral rod. Hypophallus absent. Distiphallus long, clear and coiled (Figs 709, 710)	***P.melampyga* (Loew)**
–	First flagellomere dark brown. One ori. Two rows of acrostichal setulae. Epandrium dark yellow. Surstylus barely visible when viewed laterally; with single posterior tubercle-like seta (Fig. 699). Basiphallus small, pale and flat. Hypophallus well-developed. Distiphallus stout, dark and as long as long as basiphallus + mesophallus (Figs 703, 704)	***P.dorsata* (Siebke)**

#### Species descriptions

##### Phytoliriomyza arctica

Taxon classificationAnimaliaDipteraPhytomyzinae

(Lundbeck)

Figs 126 , 127 , 693–698

Agromyza arctica Lundbeck, 1900: 304.
Phytobia (Icteromyza) arctica . Hendel, 1931: 57; Frick 1952a: 393.
Phytoliriomyza arctica . Shewell, 1953: 469; Frick 1957: 204 [lectotype designation], 1959: 414; Spencer 1969: 202; Spencer and Steyskal 1986b: 154; Černý et al. 2020: 213.
Odinia immaculata . Misidentification. Coquillett 1902: 185.

###### Description

**(Figs 126, 127, 693).** Wing length 1.6–1.9 mm (♂), 1.9–2.2 mm (♀). Length of ultimate section of vein M4 divided by penultimate section: 1.5–1.7. Eye height divided by gena height: 2.6–3.8. Eye minutely pilose. Vein r-m near basal 1/3 of cell dm. Frons soft medially and with broad groove surrounding well-sclerotised tubercle.

***Chaetotaxy***: One ori; two ors. Orbital setulae proclinate. Postocellar and ocellar setae well-developed. Four dorsocentrals, one presutural, anterior two dorsocentrals smaller. Very few acrostichal setulae in two scattered rows anteriorly; intra-alar setulae similarly reduced.

***Colouration***: Frons brownish, never light yellow, but sometimes fading to light yellow or yellow laterally; lateral margin with whitish pruinosity; ocellar tubercle dark brown; back of head brown; first flagellomere lightly to darkly infuscated on outer face on basal 2/3–3/4 or less with pigment fading anteroventrally, and dorsal margin often more darkly and more extensively infuscated, but sometimes mostly dark with venter yellow; remainder of head light yellow with clypeus dark brown, and face (sometimes lightly infuscated along midline) and parafacial paler. Notum dark, densely covered with grey pruinosity that may become more coppery posteriorly; notopleuron and postpronotum sometimes with pale mottling or faint yellow tint, but sometimes bright yellow with yellow stripe continuing along side of scutum to posterior margin. Metanotum brown with mediotergite darker and katatergite and anatergite sometimes yellowish to light yellow. Pleuron dark with lighter pruinosity, usually with dorsal margin of katepisternum (rarely including base of seta) and meron yellowish to yellow, and often at least with dorsum of anepisternum and anepimeron yellow; pigment variably faded, sometimes with most of anepisternum and anepimeron yellow, excluding limited ventral brown markings. Apical surface of halter light brown, but pigment sometimes faint to indistinct. Calypter margin and hairs brown. Legs varying from mostly brown to mostly yellow, but at least brown on base of coxae (most extensive on hind leg), femora with faint dorsal mottling (sometimes indistinct on mid and hind legs), and tibiae and tarsi brown; legs sometimes almost entirely brown to dark brown with distal 2/3 of fore femur yellow, and mid and hind femora yellow apically with additional extensive yellow(ish) mottling. Abdomen brown to dark brown with lateral margin of tergites often narrowly to widely yellow, but sometimes mostly yellow with only dorsomedial stripe and epandrium light brown to brown (ovipositor always dark brown).

***Genitalia***: (Figs 694–698) Surstylus directed inwards, angled anteriorly, subtriangular with shallow posterobasal process, setulose. Subepandrial sclerite with one pair of anteriorly directed arms and with broader posteriorly directed process at midpoint; seta on narrow membrane between arm and process; arms meet above cerci as clear bilobate plate. Hypandrium narrow and elongate distally with wide inner membrane along length and one pair of basal setulae on each lobe. Postgonite lobate, apically bifid and with two setulae. Basiphallus long and narrow with one pair of basally joined sclerites. Mesophallus and paraphallus not evident. Hypophallus small, membranous. Distiphallus clear, extremely long and flagellate, with base stouter and slightly more sclerotised. Ejaculatory apodeme small with short, wide blade and narrow stalk; venter of sperm pump dark.

###### Distribution.

Greenland. North America. Europe. Iran, South Korea (Černý et al. 2020). Taiwan. Sri Lanka. Brazil. Chile.

###### Hosts.

Asteraceae – *Crepis*, *Lapsana*, *Solidago*, *Sonchus* (Spencer and Steyskal 1986b; Spencer 1990).

###### Type material.

***Lectotype*: Denmark**: Greenland (1♂, ZMUC). [Not examined]

***Paralectotype*: Canada. AB**: Aweme, J.M. Aldrich, 11.x.1916 (2♀, USNM), 19.ix.1916 (2♀, USNM), Kushla, iv.1915, A.H. Sturtevant (1♀, USNM), Elkwater, 12.vi.1956, O. Peck, CNC479918, CNC479933 (1♂ 1♀, CNC), Orion, 49°28'N, 110°50'W, 6.vi.1955, J.R. Vockeroth, CNC391890 (1♀, CNC), Walsh, 2.viii.1927, [illegible], CNC391875 (1♂, CNC), **BC**: Kaslo, “17-7”, R.P. Currie (1♀, USNM), Creston, 8.v.1958, H.andA. Howden, CNC479938 (1♀, CNC), Ketchum L., 58°22'N, 131°45'W, 1097 m, dry sedge grass and moss bogs, 26.viii.1960, R. Pilfrey, CNC479941–479944 (4♀, CNC), W.W. Moss, CNC479940 (1♀, CNC), Kinbasket Lake, 10.vii.2010, Cooper Beauchesne and Assoc. Ltd., Malaise trap, BC Hydro drawdown study (00MRFTa15), CNC479964 (1♂, CNC), 21.vi.2008, (6RS09-15), CNC479965 (1♀, CNC), 29–30.vii.2009, (87MLRT01), CNC479966 (1♀, CNC), 4.vii.2010, (83MCOT01), (84MCOT15), (84MTRTa01), CNC479963, CNC479967–479969 (1♂ 3♀, CNC), Laird R. Hot Sprgs, 1725, 24.viii.1962, P.J. Skitsko, CNC479939 (1♀, CNC), Mt. Revelstoke, 1645 m, 13.viii.1952, G.J. Spencer, CNC479919, CNC479920 (2♂, CNC), Oliver, 13.v.1953, J.R. McGillis, CNC479937 (1♀, CNC), Trinity Valley, 29.vi.1937, H.B. Leech, CNC391896 (1♀, CNC), Vernon, 9.vi.1937, H. Leech, CNC391897 (1♀, CNC), **MB**: Aweme, 18.vii.1916, Aldrich, CNC391877 (1♂, CNC), Churchill, 58°46'N, 94°10'W, 21.vii.1952, J.G. Chillcott, Ecological data T-C 1, CNC_Diptera109368 (1♂, CNC), Farnworth Lake, near Churchill, 58°41'N, 94°3'W, 12.vi.1952, J.G. Chillcott, Ecological data F-DE, CNC_Diptera109363 (1♀, CNC), Fort Churchill, 58°46'N, 94°10'W, 10.vi.1952, J.G. Chillcott, Ecological data T-B 12, CNC_Diptera109364 (1♀, CNC), 20.vi.1952, J.G. Chillcott, Ecological data f-B 15, CNC_Diptera109365 (1♂, CNC), 21.vii.1952, J.G. Chillcott, Ecological data F-E 21, CNC_Diptera109367 (1♂, CNC), 3.vi.1952, C.D. Bird, “f-B 12” CNC_Diptera109378-109380 (1♂ 2♀, CNC), 5.viii.1952, J.G. Chillcott, Ecological data f-B 12, CNC_Diptera109366 (1♂, CNC), Mile 500, Hudson Bay Ry., 8.viii.1952, J.G. Chillcott, Ecological data F-H, along RR, CNC479932 (1♀, CNC), Treesbank, 27.viii.1915, N. Criddle, CNC391889 (1♀, CNC), **NL**: Lab. Hebron, taken on fungi, 9.viii.1954, J.F. McAlpine, CNC391853 (1♂, CNC), Labrador, Tessiujak, Nagvak Fjord, 15.viii.1954, J.F. McAlpine, CNC479922 (1♂, CNC), **NT**: Salmita Mines, 64°5'N, 111°15'W, 18.vi.1953, J.G. Chillcott, CNC391870 (1♀, CNC), **NU**: Bathurst Inl., N.W.T., Baychimo Harb., M.T.S. Gravity Survey Camp, 2.viii.1966, G.E. Shewell, CNC479936 (1♀, CNC), **ON**: Ancaster, 26.vi.1955, O. Peck, CNC391977 (1♂, CNC), Belleville, 3.vii.1950, J.C. Martin, CNC479930 (1♀, CNC), Marmora, marsh vegetation, 14.viii.1952, J.F. McAlpine, sweeping, CNC391976 (1♂, CNC), Normandale, 23.v.1956, J.R. Lonsway, CNC391978 (1♂, CNC), Ottawa, C.E.F., buckwheat, 18.vii.1951, O. Peck, sweeping, CNC391975 (1♂, CNC), Ottawa, Green Valley Motel, on Solidago, 10.vii.1967, K.A. Spencer, CNC391840, CNC391841, CNC391844, CNC391845 (4♂, CNC), 9.vii.1967, CNC391842, CNC391843 (2♂, CNC), Ottawa, Science Serv. Bldg., on window, 3.vii.1956, J.R. Vockeroth, CNC391972 (1♂, CNC), Ottawa, 12.vii.1964, J.R. Vockeroth, CNC391837 (1♀, CNC), 26.vii.1959, CNC391974 (1♂, CNC), 3.ix.1950, CNC479928 (1♀, CNC), 30.x.1956, CNC479929 (1♀, CNC), 31.x.1956, CNC391973 (1♂, CNC), damp second growth Acer-Betula wood, 4.vii.2003, CNC479927 (1♀, CNC), 9.viii.2000, CNC479972 (1♂, CNC), Strathroy, 12.vii.1916, Aldrich, CNC391893 (1♀, CNC), 5.vii.1916, Aldrich, CNC391876, CNC391892 (1♂,1♀, CNC), **QC**: Gatineau Pk., Harrington Lk., 30.v.1954, W.R. Coyles, CNC479931 (1♀, CNC), Great Whale River, 8.viii.1959, W.R.M. Mason, CNC391979 (1♂, CNC), Lac Brule, rugosa, 25.vii.1947, O. Peck, sweeping, CNC391895 (1♀, CNC), Wakefield, 9.vii.1946, A.R. Brooks, CNC391894 (1♀, CNC), **SK**: Indian Head, K. Ste[cut off], coll. 20.ix.19[??], CNC391888 (1♀, CNC), S of Moosomin Pipestone Creek, 50°2'N, 101°41'W, 570 m, prairie habitat, 2.vi.2007, Goulet, Boudreault and Fernendez, CNC315597 (1 ex, CNC), Saskatoon, 19.ix.1923, K.M. King, 16410, 5N11B, CNC391883 (1♀, CNC), 22.ix.1925, 16421, 30BS, CNC391881 (1♂, CNC), 25.v.1926, 16422, 63AN, CNC391880 (1♂, CNC), 26.v.1926, 16446, 63ASN, CNC391878 (1♂, CNC), 26.vii.1940, 16446G, 1330B, CNC391885, CNC391887 (2♀, CNC), 3.vi.1926, 16422, 64B, CNC391879 (1♂, CNC), 30.vi.1940, 16446G, 1327B, 2^nd^ ser, CNC391886 (1♀, CNC), 9.v.1949, A.R. Brooks, CNC479917 (1♂, CNC), King, coll. 22.vii.19[??], 16446G, 1229B, CNC391884 (1♀, CNC), **YT**: Aklavik, 10.vi.1953, C.D. Bird, CNC391846 (1♂, CNC), Dawson, 14 mi E, 396 m, 3.viii.1962, P.J. Skitsko, CNC479935 (1♀, CNC), North Fork Crossing, Mi. 43 Peel Plt. Rd., 64°34'N, 138°15'W, 1067 m, 4.vii.1962, P.J. Skitsko, CNC_Diptera109369 (1♀, CNC), North Fork Pass, Ogilvie Mountains, 64°34'N, 138°15'W, 11.vi.1962, R.E. Leech, CNC_Diptera109370, CNC_Diptera109371 (1♂ 1♀, CNC), 12.vi.1962, CNC_Diptera109373-109376 (2♂ 2♀, CNC), 20.vi.1962, CNC_Diptera109372, CNC_Diptera109377 (1♂ 1♀, CNC), Otter Lake, 62°30'N, 130°25'W, 1219 m, 15.vii.1960, J.E.H. Martin, CNC391847 (1♂, CNC), Takhini Hot Sprgs., 731 m, 16.viii.1962, R.E. Leech, CNC479934 (1♀, CNC). **Greenland.** Sondrestrom Air Base, 28.vii.1952, W.J. Brown, CNC479923 (1♂, CNC), 7.viii.1952, CNC479948 (1♀, CNC), 8.viii.1952, CNC479925, CNC479926, CNC479949–479951 (2♂ 3♀, CNC), 9.viii.1952, CNC479924, CNC479952–479955 (1♂ 4♀, CNC), Acc. No. 71429, “type”, “6/9 1889”, “Ipiüitat”, Sündbeck (1♂ 1♀, USNM, type No. 26953). **England**: Devon, Dawlish Warren, Pinus sylvestrus, 25.viii.1960, J.R. Vockeroth, sweeping, CNC391941–391971 (26♂ 5♀, CNC). **Spain.** Granada: N slope Veleta Sierra Nevada, 2400 m, 25.vii.1960, J.R. Vockeroth, CNC391872–391874 (3♀, CNC). **USA. AK**: Summit L., Isabella Pass, 3.viii.1951, Mason and McGillis, m-194, CNC391861 (1♀, CNC), Unalakleet, 18.vi.1961, R. Madge, CNC479959, CNC479960 (2♀, CNC), 19.vi.1961, R. Madge, CNC391854, CNC479956–479958, CNC479961 (2♂ 3♀, CNC), **CA**: Eldorado Co., Fallen Leaf, 3mi W, 1981 m, dry grassy meadow, 13.vii.1961, J.G. Chillcott, CNC479947 (1♀, CNC), Helendale, 18.v.1955, W.R. Richards, CNC479970 (1♂, CNC), Thousand Palms, 25.iii.1955, W.R.M. Mason, CNC479971 (1♂, CNC), **CO**: Hoosier Pass, 3657 m, 8.viii.1961, J.G. Chillcott, CNC391860 (1♀, CNC), Jackson Co., Rabbit Ears Pass, 7.vii.1961, J.G. Chillcott, CNC391852 (1♂, CNC), Mt. Evans, 4267 m, 25.vii.1961, B.H. Poole, CNC391839, CNC391855, CNC391856 (1♂ 2♀, CNC), J.G. Chillcott, CNC391838 (1♂, CNC), 1319 m, 8.viii.1973, J.R. Vockeroth, CNC479946 (1♀, CNC), Nederland, 2590 m, marshy stream margin, 2.vii.1961, J.G. Chillcott, CNC391857 (1♀, CNC), Niwot Ridge, nr. Ward, 3444 m, 28.vi.1961, C.H. Mann, CNC391858, CNC391859 (2♀, CNC), 3505 m, on tundra, 4.vii.1961, C.H. Mann, CNC391851 (1♂, CNC), Mt. Evans, 14,000', 25.vii.1961, J.G. Chillcott (1♀, USNM), **GA**: Rabun Bald, Rabun, alt. 4200', 16.vii.1957, J.G. Chillcott (1♀, USNM), **ID**: Moscow, 3.vii.1912, J.M. Aldrich (1♂, USNM), Michigan City, 29.vi.1915, J.M. Aldrich (1♀, USNM), Lafayette, 10.vi.1915, J.M. Aldrich (1♀, USNM), **IL**: Champaign, ex elm leaves, 25.v.1957, J.F. McAlpine, CNC479945 (1♀, CNC), **KY**: Lexington, 2.xi.1915, J.M. Aldrich (1♂, USNM), **MD**: Montgomery Co., Bethseda, G. Steyskal, 7.vii.1970 (1♂, USNM), 20.vii.1967 (1♀, USNM), Forest Glen, 25.vi.1967, W.W. Wirth (1♂, USNM), Colesville, 14.vi.1975, malaise trap, W.W. Wirth (1♂ 2♀, USNM), **MO**: 2mi W St. Louis, 30.iv.1904 (1♀, USNM), **NC**: Gr. Smoky Mt. Nat. Pk., Clingman’s Dome, 1920–2024 m, 20.v.1957, J.R. Vockeroth, CNC391849, CNC391863–391868 (1♂ 6♀, CNC), Highlands, Whiteside Mt., 1493 m, 1.vii.1957, J.R. Vockeroth, CNC391869 (1♀, CNC), Macon Co., Wayah Bald, 1615 m, 14.vii.1957, J.G. Chillcott, CNC391862 (1♀, CNC), Pisgah Nat. Forest, Chestnut Bald, 1798 m, 2.viii.1957, J.G. Chillcott, CNC391850 (1♂, CNC), **NY**: L.I., Riverhead, Veg. Res. Farm, at light, 7–20.viii.1938, CNC391882 (1♂, CNC), **OH**: Ashtabula Co., Geneva S.P., Lake Erie, 41°51.5'N, 80°58.2'W, 16.vi.1976, P. Brooks (1♀, USNM), **PA**: Centre Co., Fillmore Gap, 17.v.1983, A.L. Norrbom (1♀, USNM), **SD**: Elk Point, C.N. Ainslie, 5.vii.1915 (1♂ 2♀, USNM), CNC391891 (1♀, CNC), **TN**: Gr. Smoky Mt. Nat. Pk., Indian Gap, 1584 m, 20.v.1957, J.R. Vockeroth, CNC391848 (1♂, CNC), **VA**: Northampton Co., Kiptopeke, 4–6.x.1986, W.E. Steiner et al., Malaise trap, dunes between cliff and beach (1♀, USNM), Shenandoah Co., Mt. Jackson, 25.v.1962, J.G. Chillcott, CNC391871 (1♀, CNC), **WA**: Pullman, 25.vi.1916, J.M. Aldrich (1♀, USNM), **WY**: Sierra Madre Range, Battle L. Road, 2438 m, on side of stream, 18.vii.1961, J.G. Chillcott, CNC479962, CNC479921 (2♂, CNC).

###### Comments.

*Phytoliriomyzaarctica* is a widespread species with diverse colouration belonging to a lineage characterised by proclinate orbital setulae, a minutely setulose eye, and usually an apically brown halter and at most one small tubercle-like seta on the posterodistal corner of the epandrium. This basal offshoot also includes the Delmarva species *P.pilosella*. Many other species are highly similar in size and colouration, however, including *P.imperfecta* (Malloch) (Spencer and Steyskal 1986b: figs 979–982), which also has a faded medial yellow stripe on the scutellum (usually entirely brown-grey in *P.arctica*), and dissections should be made in all uncertain cases.

##### Phytoliriomyza dorsata

Taxon classificationAnimaliaDipteraPhytomyzinae

(Siebke)

Figs 699–704

Agromyza dorsata Siebke, 1864: 169.
Agromyza reverberata Malloch, 1924: 191. Spencer 1969 [synonymy].
Liriomyza striata Hendel, 1931: 249. Spencer 1972 [synonymy?].
Liriomyza reverberata . Frick, 1952a: 375, 1959: 409.
Lemurimyza dorsata . Spencer, 1965b: 28, 1969: 194.
Phytoliriomyza dorsata . Spencer, 1976: 294; Spencer and Steyskal 1986b: 158; Černý et al. 2020: 214.

###### Description.

Wing length 2.1–2.5 mm (♂), 1.9–2.6 mm (♀). Length of ultimate section of vein M4 divided by penultimate section: 1.2–1.5. Eye height divided by gena height: 3.9–5.0. Eye bare. First flagellomere slightly longer than high, rounded, long axis slightly angled ventrally. Vein r-m near midpoint of cell dm.

***Chaetotaxy***: One ori; two ors. Orbital setulae erect, in a single row. Postocellar and ocellar setae well-developed. Four dorsocentrals, one presutural, decreasing in length anteriorly. Acrostichal setulae in two long rows, relatively long, posterior pair slightly to distinctly convergent.

***Colouration***: Head light yellow with first flagellomere dark brownish black, back of head brown above foramen excluding margin, ocellar tubercle yellow to light brown medially with brown margins or extensions to ocelli; face, gena, parafacial and postgena whitish. Thorax with greyish pruinosity distinct on pigmented regions. Scutum yellow with dark medial stripe on anterior 2/3, one pair of presutural spots, and two pairs of postsutural stripes, with outer supra-alar pair small, narrow, and more posterior, and inner intra-alar pair sometimes connected to presutural spot (only fused to postsutural intra-alar stripe in one Ontario female). Scutellum yellow with lateral corner sometimes narrowly brownish grey. Mediotergite dark brown; katatergite and anatergite yellow with venter sometimes narrowly brown. Pleuron yellow, anatergite sometimes with minute, faint anteroventral spot, meron mostly brown, and katepisternum with brown spot not reaching base of seta. Halter entirely white. Calypter margin and hairs brown. Legs light yellow, sometimes with tarsi and tibiae faintly brownish. Abdomen yellow, sometimes with faint dorsomedial stripe and epandrium brownish.

***Genitalia***: (Figs 699–704) Inner surface of epandrium with comb of long, fused tubercle-like setae beside one longer spine; ventral margin with minute tubercle-like setae, widely spaced in straight to irregular row. Surstylus small and rounded with one to three tubercle-like setae posteriorly. Subepandrial sclerite consisting of V-shaped sclerite with one pair of medial setae and flat, pale, ventral bilobed process. Hypandrium thin with one seta and lobe sclerotised only along outer margin. Postgonite bare and broadly rounded apically. Basiphallus with narrow plate on left side (broad apically) and lightly sclerotised anterodorsal margin. Paraphallus absent. Hypophallus broad and membranous with one pair of small, converging sclerites medially and lateral margin very lightly sclerotised. Mesophallus dark, cylindrical, as long as distiphallus. Distiphallus comprised of one pair of stout, elongate tubules that are parallel basally; basal 1/2 composed of dark bulbous sclerite and weaker medial region that is nearly band-like; distal 1/2 cylindrical, pigmented. Ejaculatory apodeme pale and fan-shaped with narrow stalk, broad base, and clear sperm pump.

###### Host.

Unknown.

###### Distribution.

**Canada**: BC*, MB*, NT*, ON, QC*, YT*. **USA**: CA, LA, MD, MI, PA, VA, WY*. Europe, Russia, Iran, Japan (Černý et al. 2020).

###### Type material.

***Holotype* [*dorsata*]: Norway.** Opland: Jerkin (1♂, ZMUN). [Not examined]

***Syntypes* [*striata*]: Romania.** Mehadia.; **Austria.** “Ossiacher-See und Dobratsch, Karnten… Donau-Auen bei Wien”; **Russia.** Leningrader Bezirk (7♂♀, NMW). [Not examined]

***Holotype* [*reverberata*]: USA. MD**: Glen Echo, 14.v.1922, J.R. Malloch (1♀, [Lost]).

***Paratype* [*reverberata*] : USA. MD**: same collection as holotype (1♀, CNC). [Not examined]

###### Additional material examined.

**Canada. BC**: Kinbasket Lake, 17–18.vii.2009, Cooper Beauchesne and Assoc. Ltd., Malaise trap, BC Hydro drawdown study (12MTRT15), CNC479993 (1♀, CNC), **MB**: Shilo, 5mi SW, tamarack, 2.viii.1958, J.G. Chillcott, CNC479997 (1♀, CNC), Western MB, Riding Mountain Nat. Park, Clear Spring spruce bog, 50°41'N, 99°48'W, 650 m, 10.vii.2008, J. Crossey, N. Jeffery, J. Straka, CNC391899, CNC391900 (2♂, CNC), **NT**: Norman Wells, 25.vi.1969, G.E. Shewell, CNC479991 (1♂, CNC), Wood Buffalo National Park, Benchmark weather station, 59°34'N, 112°16'W, 219 m, aspen stand, 27.vii.2012, N. Labine, CNC391901 (1♀, CNC), **ON**: Ottawa, Dow’s Swamp, 5.vii.1947, CNC391902 (1♂, CNC), Ottawa, 17.vi.1946, G.E. Shewell, CNC391903 (1♀, CNC), 18.vi.1957, J.G. Chillcott, CNC479999, CNC480000 (2♀, CNC), damp second growth Acer-Betula wood, 5.vii.2000, J.R. Vockeroth, CNC479998 (1♀, CNC), **QC**: Cap Rouge, 4.vii.1953, R. Lambert, CNC479994 (1♀, CNC), Old Chelsea, 18.vii.1961, J.R. Vockeroth, CNC479995 (1♀, CNC), 30.vi.1985, CNC479996 (1♀, CNC), **YT**: Dawson, 14 mi E, 396 m, 31.vii.1962, P.J. Skitsko, CNC479992 (1♂, CNC). **USA. LA**: Alexandria, 11 mi SW, 26.iii.1960, J.G. Chillcott, CNC391904 (1♀, CNC), **VA**: Giles Co., Stony Creek, 609 m, 26.v.1963, J.R. Vockeroth, CNC391905 (1♀, CNC), Shenandoah, vii.1939, A.L. Melander (1♂, USNM), **WY**: Sierra Madre Range, Battle L. Road, 2438 m, on side of stream, 18.vii.1961, J.G. Chillcott, CNC479990 (1♂, CNC).

###### Comments.

*Phytoliriomyzadorsata* is distinct among the known Delmarva species, but it is highly similar to *P.pacifica* (Melander) (Spencer and Steyskal 1986b: figs 1006, 1007), which occurs from Ontario to Saskatchewan in Canada, and Washington and Idaho in the United states, but is likely more widespread. Externally, *P.pacifica* differs in having the scutal stripes confluent, or at least connected by a brownish orange infuscation (not with medial and postsutural supra-alar stripes discrete), the anepisternum has an anteroventral spot that is longer than high (not absent to minute), the tibiae and tarsi are brown, at least on the hind leg (not yellow to faintly brownish). With regard to the male genitalia, the ventral margin of the epandrium has one or two minute tubercle-like setae (not several widely spaced in a straight to irregular row), the surstylus has one spine (one to three in *P.dorsata*), and the distiphallus is diverging basally and with a dark basal section that is relatively long and narrow (not parallel with dark basal section shorter and rounded).

##### Phytoliriomyza melampyga

Taxon classificationAnimaliaDipteraPhytomyzinae

(Loew)

Figs 129 , 705–710

Agromyza melampyga Loew, 1869: 48.
Agromyza impatientis Brischke, 1881: 245. Spencer 1969 [synonymy].
Liriomyza impatientis . Hendel, 1931: 225.
Liriomyza melampyga . Frick, 1952a: 404, 1957: 203 [lectotype designation]; Spencer 1969: 178; Shewell 1953: 467.
Phytoliriomyza melampyga . von Tschirnhaus, 1971: 562; Spencer and Steyskal 1986b: 157; Černý 2018: 131; Scheffer and Lonsdale 2018: 87; Černý et al. 2020: 200.

###### Description

**(Fig. 129).** Wing length 2.1–2.4 mm (♂), 2.0–2.5 mm (♀). Length of ultimate section of vein M4 divided by penultimate section: 1.3. Eye height divided by gena height: 3.7–4.3. Eye bare. Vein r-m at midpoint of cell dm or beyond.

***Chaetotaxy***: Two to three ori; two ors. Orbital setulae reclinate and well-developed, in one row. Postocellar and ocellar setae well-developed, longer than ors. Four dorsocentrals, one presutural, decreasing in length anteriorly. Acrostichal setulae in four irregular rows.

***Colouration***: Head light yellow with ocellar tubercle light brown to yellow and back of head brown above foramen excluding margins. Thorax mostly light yellow with light greyish pruinosity evident on pigmented sections; scutum with medial stripe on anterior 2/3 that is sometimes confluent with one pair of lateral presutural spots; postsuturally with one very narrow pair of floating supra-alar stripes posteriorly and one pair of wider intra-alar stripes that taper posteriorly and sometimes fuse to presutural spot; pattern sometimes enlarged with stripes extensively fused, encompassing all but lateral and posterior margins of scutum. Scutellum yellow, sometimes with lateral corner brownish. Metanotum yellow with mediotergite dark brown and anatergite and katatergite variably brown posteroventrally. Pleuron yellow with meron brown excluding dorsum, and katepisternum with large brown spot that does not reach base of seta. Halter entirely white. Calypter margin and hairs brown. Legs yellow with tibiae dark yellow to brownish. Abdomen light yellow with at least sides of epandrium brown; sometimes with faint paired brownish spots on pregenital tergites.

***Genitalia***: (Figs 705–710) Epandrium with broad anterior emargination basal to fused surstylus; posteromedial margin with comb of long tubercle-like setae. Surstylus subquadrate, only slightly converging and with posteromedial margin with comb of long tubercle-like setae; outer surface bare, inner surface setose. Subepandrial sclerite bare and subrectangular with centre paler and corners produced; ventrally with one pair of dark narrow arms and shallow rounded medial process. Hypandrium bare, long, and thin with only margin of lobe sclerotised. Postgonite rounded and cleft apically. Basiphallus consisting of single dark elongate rod on left side, Mesophallus cylindrical, widest at base, which is 1/2 length. Distiphallus made up of one pair of stout, clear, elongate tubules that are laterally pigmented subbasally. Ejaculatory apodeme small and dark with base stout and blade highly reduced; sperm pump clear.

###### Distribution.

**Canada**: AB*, NB*, NS*, ON, PE*, QC*, SK*. **USA**: DC, MA, MD, MI, NC*, NJ, NM*, NY, PA*, VA*, MN, WI. Europe, India, Kazakhstan, Russia, South Korea (Černý et al. 2020).

###### Hosts.

Balsaminaceae – *Impatiens*.

###### Type material.

***Lectotype* [*melampyga*]: USA. DC** [not given]: “Loew coll.; *melampyga* m.” (1♀, MCZ). [Not examined]

***Syntype* [*impatientis*]: Poland.** Gdansk (as “Oliva”) [type information unknown]. [Not examined]

###### Material examined.

**Canada. AB**: Elk Island NP, Wood Bison Trail, 53°34'N, 112°50'W, 722 m, aspen forest, 2.vii.2012, BIOBus, CNC391917 (1♂, CNC), **NB**: Shediac, 8.vii.1967, N.L.H. Krauss (♂, USNM), **NS**: CBHNt. Pk., Beulach Ban Falls, along fast rocky stream, 11.vii.1983, J.R. Vockeroth, sweeping, PG812870, CNC479986 (1♂, CNC), **ON**: Cyrville Road, 2mi E Ottawa, 31.v.1965, B.V. Peterson, CNC479985 (1♀, CNC), Elizabethtown, 4452 Rowsome Rd., 44°37'N, 76°16'W, 120 m, 2.vi.2010, James Sones, CNC391918 (1♂, CNC), 4.vi.2010, CNC391919 (1♀, CNC), Grand Bend, 14.vii.1939, G.E. Shewell, CNC391929 (1♀, CNC), Ottawa, Black Rapids, 28.vi.1959, J.R. Vockeroth, CNC479983 (1♂, CNC), Ottawa, Dow’s Swamp, 5.vii.1947, W.R.M. Mason, CNC391930, CNC391926 (1♂ 1♀, CNC), Ottawa, 20.vii.1963, J.R. Vockeroth, CNC391908 (1♀, CNC), 28.vi.1953, J.R. Vockeroth, CNC391909–391914 (4♂ 2♀, CNC), 27.vii.1946, A.R. Brooks, CNC391931 (1♀, CNC), 8.viii.1946, G.E. Shewell, CNC391932, CNC479984 (2♀, CNC), Puslinch, Property of Bob Hanner, 43°27'N, 80°15'W, 335 m, hardwood forest, 18.ix.2008, T. Terzin, CNC391924 (1♂, CNC), 21.viii.2008, CNC391920 (1♀, CNC), 28.viii.2008, CNC391922 (1♀, CNC), 3.viii.2008, CNC391921, CNC391923, CNC391925 (1♂ 2♀, CNC), St. Lawrence Is. Nat. Park, Thwartway Is., 13.vii.1976, H.J. Teskey, Code – 4073Q, CNC391927 (1♂, CNC), **PE**: Charlottetown, vii.1967, N.L.H. Krauss (1♂, USNM), **QC**: Abbotsford, 18.v.1936, Shewell, CNC391928 (1♀, CNC), Beech Grove, 7.vi.1955, J.F. McAlpine, CNC479982 (1♀, CNC), Gatineau Hills, in park, King Mt. footpath, 30.vii.1959, L.K. Smith, CNC391906 (1♂, CNC), Old Chelsea, 11.viii.1959, C.H. Mann, CNC479980 (1♂, CNC), 25.vi.1959, J.G. Chillcott, CNC391907 (1♀, CNC), Rigaud, 11.vi.1981, J.R. Vockeroth, CNC479981 (1♂, CNC), **SK**: Prince Albert NP, Narrow Penninsula Trail, 53°59'N, 106°17'W, 530 m, white spruce and poplar forest, 14.vii.2012, BIOBus, CNC391916 (1♂, CNC). **USA. MA**: Bedford, 20.vii.1961, swamp, W.W. Wirth (3♂ 2♀, USNM), Woods Hole, “7-15-2”, A.L. Melander (1♀, USNM), **MD**: Plummers Isl., 30.v.1913, R.C. Shannon (1♀, USNM), Glen Echo, 9.vii.1922, J.R. Malloch (1♀, USNM), Montgomery Co., Bethseda, G.C. Steyskal, 29.vii.1972 (1♂, USNM), 23.v.1970 (2♂, USNM), 17.v.1969 (1♂, USNM), 4.vii.1977 (1♂, USNM), Dickerton, 14.vii.1974, G.A. Foster (2♂ 1♀, USNM), Sometset Co., Snow Hill, 16.vii.1968, swamp margin, W.W. Wirth (2♂ 1♀, USNM), **MI**: Clinton Co., Rose Lake, 24.v.1941, C. Sabrosky (1♀, USNM), **NC**: Gr. Smoky Mt. Nat. Pk., Clingman’s Dome, 1920–2024 m, 18.vi.1957, J.R. Vockeroth, CNC479987 (1♂, CNC), Gr. Smoky Mt. Nat. Pk., Indian Gap, 1584 m, 2.vii.1957, J.R. Vockeroth, CNC479988 (1♀, CNC), Mt. Mitchell, 2072 m, 12.viii.1957, J.G. Chillcott, CNC479989 (1♀, CNC), **NM**: Cloudcroft, 9000', June, W. Knaus (1♂, USNM), **NY**: New York, 28.ix.1921, A.H. Sturtevant (1♂, USNM), Long Island, Cow Neck, 24.v.1963, spring seep, W.W. Wirth (1♀, USNM), Erie Co., E Concord Bog, 1.vi.1963, maple swamp, W.W. Wirth (1♀, USNM), **PA**: Chester Co., Oxford, 127 West Locust, 29.ix.1998, Malaise trap, RLS, 26-iix, 6-ix (1♂, USNM), **VA**: Shenandoah, Big Meadows, 15.vi.1941, A.L. Melander (3♂, USNM), Fairfax Co., Dead Run, R.C. Shannon, 19.vi.1915 (1♂, USNM), 28.vii.1915 (1♀, USNM).

###### Comments.

*Phytoliriomyzamelampyga* is a distinct bright yellow species that is encountered with relative frequency around *Impatiens*. *Liriomyzablechi* is similar in external appearance, but the pregenital abdomen is brownish dorsally, not entirely yellow or with faint spots. Also similar is the Canadian *P.viciae* (Spencer) (Spencer 1969: figs 346, 347), which has a darker phallus that is more stongly arched and with a single elongate black basal section (not two smaller, paler sections), and only a few single spines on the epandrium; externally, tergites 1 and 2 are mostly brown in the male and some females, ground colour is yellow, not yellow to whitish yellow, the orbital setulae are indistinct, and most diagnostically, there are two rows of acrostichal setulae (not four).

##### Phytoliriomyza pilosella

Taxon classificationAnimaliaDipteraPhytomyzinae

Spencer

Figs 711–715

Phytoliriomyza pilosella Spencer in Spencer and Stegmaier 1973: 116. Spencer and Steyskal 1986b: 153.

###### Description.

Wing length 1.5 mm (♂), 1.3–1.6 mm (♀). Length of ultimate section of vein M4 divided by penultimate section: 1.8–2.0. Eye height divided by gena height: 3.8–10.5. Eye minutely setulose dorsally. Vein r-m near basal 1/3 of cell dm. Frons soft medially.

***Chaetotaxy***: One ori; two ors. Postocellar and ocellar setae well-developed, but not much longer than ocellar tubercle. Orbital setulae proclinate, in one row. Four dorsocentrals, one presutural, decreasing in length anteriorly with anterior two pairs considerably thinner and smaller, being < 1/2 length of first dorsocentral. Several acrostichal setulae in two rows anteriorly that are often reduced to absent. Hairs on first flagellomere slightly longer than, or as long as arista base.

***Colouration***: Frons brownish with tubercle brown; face, gena and parafacial light yellow; male first flagellomere lightly infuscated posteriorly; female first flagellomere and pedicel light brown, sometimes yellowish ventrally or basally. Notum brown with grey pruinosity, anterolateral margin of scutum yellow, and anatergite below scutellum darker; postpronotum and notopleuron yellowish; yellowish postsutural supra-alar stripe sometimes visible. Pleuron yellow with most of katepisternum and meron brown, remainder with limited, narrow spots sometimes visible, including anteroventrally and sometimes anteriorly on anepisternum. Apical surface of halter light brown. Calypter margin and hairs faintly brownish. Legs yellow with base of fore coxa light brown, tibiae brownish distally and tarsi brownish with distal segments darker; dorsal brown mottling faint apically on mid and hind femora, more developed subapically and apically on fore femur, pigment pronounced in [dissected] Ontario male and Californian specimens. Abdomen light brown, paler laterally and apically, epandrium yellow, oviscape brown to yellow with partial brown markings.

***Genitalia***: (Figs 711–715) Epandrium rounded apically and with one tubercle-like seta on inner-distal margin. Surstylus narrow, curved, emerging from anterior margin of epandrium, with several apical setulae. Subepandrial sclerite H-shaped with lateral margins bowed. Hypandrium thin with lobe broad and bare. Postgonite narrowly rounded and cleft apically. Basiphallus sclerotised on dorsal margin. Paraphallus and hypophallus absent. Mesophallus not evident. Distiphallus composed of one pair of extremely elongate, narrow, clear tubules with base dark and slightly swollen. Ejaculatory apodeme stout, pale and fan-shaped with stem very short; sperm pump with lightly sclerotised band.

*Variation*: Specimens from California with brownish palpus, specimens from Florida and Costa Rica with dark brown palpus. Florida specimen with narrow, distinct yellow postsutural intra-alar line and complete dorsocentral line; with enlarged setula in front of anterior dorsocentral; setulae on eye virtually absent.

###### Host.

Asteraceae – *Cotula* (Spencer 1990).

###### Distribution.

**Canada**: ON*. **USA**: CA*, FL, KY*, MD*, MS*, VA*. Costa Rica. Puerto Rico. Spain (Canary Islands).

###### Type material.

***Holotype*: Puerto Rico**: El Yunque, 20–22.iii.1954, J. Maldonado and S. Medina (1♂, USNM).

###### Paratypes examined.

**Costa Rica.** San Jose: La Caja, 8 km W, 1930, Schmidt, CNC481318 (1♀, CNC). **Puerto Rico.** El Yunque, 20–22.iii.1954, J. Maldonado Capriles (1♀, USNM). **USA. FL**: Dade Co., Farm near Royal Palm Hammock, 4.xii.1961, Munroe, Holland and Chillcott, CNC481317 (1♀, CNC).

###### Additional material examined.

**Canada. ON**: Puslinch, Property of Bob Hanner, 43°26'47.04"N, 80°15'4.32"W, hardwood forest, 3.viii.2008, T. Terzin, CNC481319 (1♂, CNC). **USA. CA**: Marin Co., Alpine Lk., Lily Pond, 457 m, 17–25.v.1971, Malaise trap, CNC481320, CNC481321 (1♂,1♀, CNC), iv-v.1970, D.D. Munroe, CNC481323–481325 (3♀, CNC), v-vi.1970, CNC481322 (1♀, CNC), **KY**: Lexington, 2.xi.1915, J.M. Aldrich (1♂, USNM), **MD**: “ChespkBch” “viii-2”, J.M. Aldrich (1♀, USNM), **MS**: Noxubee National Wildlife Refuge, near Bluff Lake, River Road, 33°17'12"N, 88°46'42"W, 65 m, 18–19.v.2013, J.E. O’Hara, Malaise trap, CNC758941 (1♀, CNC), **VA**: Falls Church, Holmes Run, 13.ix.1960, light trap, W.W. Wirth (1♀, USNM).

###### Comments.

*Phytoliriomyzapilosella* is a small, delicate species most readily diagnosed by longer hairs on the first flagellomere and a long, clear coiled tubular distiphallus. The new material listed here represents a significant Nearctic range extension for this species since it was only otherwise known as far north as the southern tip of Florida.

Spencer (1990) recommended additional study of this species in comparison to *Phytoliriomyzascotica* Spencer, which may be conspecific. Re-evaluation of the boundaries of this species is also recommended here, due to variation in palpus and antenna colour, but also chaetotaxy. The long-haired first flagellomere is also seen in *P.minutissima* Spencer, but this differs in having a medially yellow scutellum.

##### Phytoliriomyza pulchella

Taxon classificationAnimaliaDipteraPhytomyzinae

Spencer

Figs 717–722

Phytobia (Praspedomyza) clara (Melander). Misidentification, in part. Frick 1959: 394.
Pteridomyza hilarella . Misidentification, in part. Spencer 1969: 200 (synonymy of clara).
Phytoliriomyza clara (Melander). Misidentification, in part. Spencer and Steyskal 1986b: 159.
Phytoliriomyza pulchella Spencer in Spencer and Steyskal 1986b: 304.

###### Description.

Wing length 1.5–1.6 mm (♂), 1.8–1.9 mm (♀). Length of ultimate section of vein M4 divided by penultimate section: 1.2–2.0. Eye height divided by gena height: 1.9–2.7. Eye bare. Vein r-m near distal 1/3 of cell dm. Frons soft medially.

***Chaetotaxy***: Two ori with anterior seta reduced to (rarely) absent; two ors. Orbital setulae minute, erect to reclinate, sometimes indistinct, in one row. Postocellar and ocellar setae well-developed, not much longer than tubercle. Four dorsocentrals, one presutural, decreasing in length anteriorly. Acrostichal setulae in four sparse rows. Setae sometimes appearing slightly to very conspicuously thin.

***Colouration***: Setae dark with orbital setulae, vibrissa and genal and subgenal setae yellow in male, yellow to brownish or brown in female. Head mostly light yellow, whitish below antenna; ocellar tubercle and posterolateral margin of frons (including base of vertical setae) brown with faint stripe sometimes extending to base of posterior fronto-orbital; back of head brown above foramen; female first flagellomere with orange to brownish tint that fades at base. Notum dark brown, covered with short, relatively dense greyish pruinosity; lateral margin of scutum with yellow stripe that is narrower postsuturally, and with brown spot on notopleuron (minute) and postpronotum. Metanotum brown with most of katatergite yellow anteriorly. Pleuron light yellow with most of katepisternum (not reaching base of seta) and meron brown. Halter entirely white. Calypter margin and hairs brown. Legs light yellow with tarsi darker apically. Abdominal tergites brown with lateral margin yellow.

***Genitalia***: (Figs 717–722) Epandrium with irregular row of long tubercle-like setae along posterior margin. Surstylus small and rounded with comb of long tubercle-like setae on inner surface of posterior margin; longer, pointed tubercles on shallow inner-basal process. Subepandrial sclerite U-shaped with one pair of medial setae, irregular lateral sclerotisations and one pair of thin ventral lobes. Hypandrium thin and narrowly arched, with several setae on long inner lobe. Basiphallus dark, not much longer than wide, positioned distally, separate from phallophorus. Mesophallus not evident. Hypophallus long and dark with membranous apex. Distiphallus a single pale tubule along most of length with base dark and heavily sclerotised ventrally; apex bifid, with tubules narrower and strongly divergent. Ejaculatory apodeme large and well-sclerotised stem short and base wide to one side; sperm pump clear.

###### Hosts.

Unknown – possibly the bracken fern *Pteridiumaquilinum* (Spencer and Steyskal 1986b), upon which its putative sister species *Phytoliriomyzaclara* is found. Adults have been swept from ferns in Ontario.

###### Distribution.

**Canada**: NB*, NL*, NS*, ON, PE*, QC. **USA**: FL*, MD*, MI*, NH*, NY, VA*.

###### Type material.

***Holotype*: USA. NY**: Long Island, Farmingdale, 2.vi.1935, Blanton and Borders (HT ♂, CUIC). [Not examined]

###### Material examined.

**Canada. NB**: Kouchibouguac N.P. Forest, 46°50'N, 64°55'W, edge, 30.vii-8.viii.2013, O. Lonsdale, CNC316814 (1♀, CNC), **NL**: St. John’s, Agric. Exp. Sta., 30.vii.1967, J.F. McAlpine, CNC391939 (1♂, CNC), **NS**: CBHNt. Pk., Mackenzie Mt., 13.vii.1984, PG640848, CNC479976–479979 (1♂, 3♀, CNC), **ON**: Griffith, 7mi east, 9.vii.1991, J.R. Vockeroth, CNC479975 (1♂, CNC), Simcoe, 14.vi.1939, G.E. Shewell, CNC391934–391936 (3♀, CNC), Bruce Co., Sauble Falls, ferns in clearing, 28.vii.1977, W. Maddison (2♂ 1♀, ROM), Simcoe, 14.vi.1939, G.E. Shewell, CNC391934–391936 (3♀, CNC), **PE**: Charlottetown, vii.1967, N.L.H. Krauss (2♀, USNM), **QC**: Cap Rouge, 7.vii.1953, R. Lambert, CNC391933 (1♂, CNC), Gatineau Pk., 13 km N of Eardley, 24.vi.1988, H.C. Walther, CNC479973, CNC479974 (1♂,1♀, CNC), Lac Brule, 7.viii.1951, O. Peck, CNC391938 (1♀, CNC). **USA. FL**: Mount Pleasant, 1.v.1952, O. Peck, CNC391937 (1♀, CNC), Orange Park, 25.iii.1952, O. Peck, CNC391940 (1♂, CNC), **MD**: Colesville, 4.vi.1977, W.W. Wirth (1♂, USNM), **MI**: Hunt Ck., Exp. Sta., nr. Lewiston, 20.vii.1942, C.W. Sabrosky (1♀, USNM), Cadillac, 15.vi.1941, C.W. Sabrosky (1♀, USNM), Hart, 19.vi.1939, C.W. Sabrosky (1?, USNM), Traverse City, 17.vi.1943, C. Sabrosky (1♀, USNM), **NH**: White Mts., Stinson lake, 23.vii.1961, W.W. Wirth (1♀, USNM), **VA**: Shenandoah, 14.vi.1982, H. Goulet, big meadows (1♂, USNM).

###### Comments.

The similar species *Phytoliriomyzapulchella* (North America), *P.clara* (USA) and *P.hilarella* (Europe) are rediagnosed below. Differences are still comparatively slight, and definitions should be reapproached when more numerous specimens from a broader geographic range are available.

The geographical distribution of *Phytoliriomyzaclara* is uncertain, but at present there is no evidence that it occurs in the Delmarva states. Spencer and Steyskal (1986b) restate Frick’s distribution of California, Washington, Michigan, Tennessee, Maine, and Ontario, and then add Maryland, New Mexico, and Arizona. Of the material examined by Frick, only the type series of *Agromyzacitreiformis* Malloch (= *P.clara*) from California can be found, as well as non-type material from Ontario, Michigan, and Maryland, which are here re-identified as *P.pulchella*. The Ontario and Quebec specimens from the CNC examined by Spencer (1969) as *Pteridomyzahilarella* are also here determined to be *P.pulchella*. Oregon* is here added to the distribution of *P.clara* – otherwise only confidently known from California and Washington. Data are as follows: USA. OR: Curry Co., Cape Blanco, 29.vi.1972, G. Steyskal (4♂, USNM). Locating the remaining specimens examined by Frick will be important in clarifying whether or not this species occurs further to the east. Females from New Brunswick (Kouchibouguac, 1♀, CNC), Nova Scotia (Cape Breton, Glasgow Lake, 2♀, CNC) and British Columbia (Pacific Rim N.P., 1♀, CNC) have a brown first flagellomere and appear to fit the new description of *P.clara* provided here, but are only tentatively identified as they are mostly in poor condition and lack the additional diagnostic male genitalic features. Melander’s holotype of *P.clara* at the USNM is largely destroyed, currently represented by only a crumpled wing embedded in glue, part of a leg, and Spencer’s genitalic dissection.

All Canadian specimens of *Phytoliriomyzahilarella* reported by Spencer (1969) are here determined to be *P.pulchella*, restricting *P.hilarella* to Europe. *Phytoliriomyzahilarella* can be diagnosed as follows: setae dark brown with only orbital setulae sometimes brown; scutum sometimes without yellow lateral regions, being entirely brown; acrostichal setulae sometimes in two to three scattered rows; brownish stripe extending from posterior margin of frons along fronto-orbital plate to anterior ors, but sometimes to anterior or posterior ors along inner margin of fronto-orbital plate; distiphallus strongly sinuate with short apical curve (Spencer 1976: fig. 532).

*Phytoliriomyzaclara* differs from *P.pulchella* as follows: male wing length 1.8–2.0 mm; setae not thinner; always one ori (never two); oral setae dark brown (never yellow to brown); first flagellomere pale; hypophallus slightly longer, darker and apically split; phallus slightly longer, more curved and with light pigment on apical cup (Fig. 716).

##### Phytoliriomyza robiniae

Taxon classificationAnimaliaDipteraPhytomyzinae

(Valley)

Figs 723–726

Liriomyza robiniae Valley, 1982: 781. Spencer and Steyskal 1986b: 108.
Phytoliriomyza robiniae . Spencer 1990: 401; Scheffer and Lonsdale 2018: 88 (likely misidentification; Eiseman and Lonsdale 2018).

###### Description.

Wing length 1.6–1.8 mm (♂), 1.8–2.1 mm (♀). Length of ultimate section of vein M4 divided by penultimate section: 1.4–1.6. Eye height divided by gena height: 2.2–2.6. Eye bare. Arista short, as long as scape to first flagellomere. Fronto-orbital plate and parafacial produced, continuing as “cheek” under eye. Veins R_4+5_ and M1 very closely spaced. Vein r-m at midpoint of cell dm.

***Chaetotaxy***: Three ori (sometimes only two on one side); two ors. Orbital setulae absent. Postocellar and ocellar setae well-developed. Three postsutural dorsocentrals shorter than scutellar setae, strongly decreasing in length anteriorly, with third dc barely larger than surrounding setulae. Acrostichal setulae in four irregular rows that narrow to two posteriorly.

***Colouration***: Head yellow with ocellar tubercle and spot between vertical setae brown (wider in male); clypeus dark brown (sometimes yellow medially); back of head dark brown with venter yellow; examined female with frons brownish with sides white pruinose and first flagellomere brownish posterodorsally; sometimes with spot at base of posterior one to three fronto-orbitals. Scutum dark brown with grey pruinosity, with postpronotum (excluding large spot), notopleuron, supra-alar region, and posterolateral spot yellow. Scutellum brown with ill-defined yellow medial stripe that widens apically. Metanotum dark brown with katatergite at least partially yellow anteriorly. Pleuron brown with dorsal margin of meron and dorsal margin of katepisternum (including base of seta) yellow; anepisternum and anepimeron mostly yellow in examined female, but almost entirely brown in holotype. Halter entirely white. Calypter margin and hairs dark brown. Male legs yellow with dorsal mottling on femora, tibiae brown dorsomedially (indistinct on fore leg and pronounced on hind leg), and tarsi brown with basal three segments on fore leg and basal two on mid leg yellow; female with tibiae and tarsi brown. Male abdomen brown with lateral and posterior margins of tergites 1–6 yellow, and anterolateral region of tergite 1 yellow; female abdomen brown to midpoint of tergite 6 (remainder of segment yellow), yellow beyond with oviscape dark brown.

***Genitalia***: (Figs 723–726) Epandrium and surstylus simple, without spines or outstanding setae. Surstylus articulating with epandrium. Cercus small and weakly sclerotised, minutely setose. Subepandrial sclerite made of one pair of short, dark, widely separated lateral bars. Phallophorus cylindrical, relatively long, fused to atrophied and membranous phallus, which has one pair of narrow, weakly sclerotised basal bands, one pair of leaf-like extensions, and one short flagellate apical extension. Ejaculatory apodeme highly reduced, pale, and finger-like; sperm pump membranous.

###### Host.

Fabaceae – *Robiniapseudoacacia*.

###### Distribution.

**USA**: MD*, PA. Records from NY, VA and WV are likely misidentifications (Eiseman and Lonsdale 2018).

###### Type material.

***Holotype*: USA. PA**: Dauphin Co., Harrisburg, 2301 N. Cameron Street, 22.iv.1981, A.G. Wheeler, Jr., [adult] taken on Robiniapseudoacacia (1♂, USNM).

###### Additional material examined.

**USA. MD**: Pr. Georges Co., Oxon Hill, 19.iv.1972, at black light, G.F. Hevel, “Phytoliriomyza sp. 1 ♀” (1♀, USNM).

###### Comments.

This species was originally treated as *Liriomyza* by Valley (1982) on the basis of stridulatory spicules on the abdominal membrane, but Spencer (1990) later moved the species to *Phytoliriomyza*, being unable to locate the file or femoral scraper (their absence is verified here upon examination of the holotype, and while a number of spicules appear to be present, they are largely scattered along the anterior abdominal membrane, sometimes in short, ill-defined rows). Spencer also noted that the phallus does not appear to be consistent with those of *Liriomyza* species, but this structure is atrophied to the point that it may have retained little phylogenetically relevant information. Other features of this unusual species are the presence of only three short dorsocentrals, a grey pruinose notum with a faded medial stripe on the scutellum and lateral yellow stripes on the scutum, a medially atrophied subepandrial sclerite and a bare surstylus with only several simple setae.

When Spencer placed this unusual species in *Phytoliriomyza*, he only did so tentatively, suggesting the possibility of erecting a new monotypic genus for the species. Support for this concept came later in the molecular phylogeny of Scheffer et al. (2007), where *P.robiniae* was recovered far from the *Phytoliriomyza* genus group, closer to *Amauromyza*, *Phytobia*, and *Phytomyza*, although basal support for many of these branches was weak.

##### Phytomyza

Taxon classificationAnimaliaDipteraPhytomyzinae

Fallén

Phytomyza Fallén, 1810: 10. Type species: Phytomyzaflaveola Fallén, 1810 [= Muscaranunculi Schrank, 1803], by monotypy. Frick 1952a: 421, 1959: 420; Spencer 1969: 218; Spencer and Steyskal 1986b: 172; Winkler et al. 2009: 260; Papp and Černý 2020: 175.
Phytomyia . Misspelling. Haliday, 1833: 150.
Napomyza Curtis, 1837: 282 [attributed to Haliday manuscript name]. Type species: PhytomyzanigricornisMacquart 1835, by monotypy.
Napomyza Westwood, 1840: 152 [as subgenus of Phytomyza]. Type species: Phytomyzafestiva Meigen, 1830 [= Phytomyzaelegans Meigen, 1830], by monotypy by first reviser action of Hendel (1920). Hendel 1920: 111 [attrib. to Haliday; as genus]; Frick 1952a: 419, 1959: 419; Spencer 1969: 210; Griffiths and Steyskal 1986: 170 [proposed supression Napomyza Curtis]; Spencer and Steyskal 1986b: 167; Zlobin 1994: 289; ICZN 1988: 77 [suppression of Napomyza Curtis]; Winkler et al. 2009: 271 [as subgenus]; Papp and Černý 2020: 121.
Chromatomyia Hardy, 1849: 390. Type species Phytomyzapericlymeni de Meijere, 1924 (misidentified as Phytomyzaobscurella Fallén, 1823 by Hardy and Coquillett), by subsequent designation (Coquillett 1910: 523) – see discussion in Griffiths (1974: 36). This name is not preoccupied by Chromatomyia Walker, as Walker’s name is not available. Braschnikov 1897: 40; Coquillett 1910: 523; Frick 1952a: 421 [as synonym of Phytomyza]; Griffiths 1974: 36; Spencer and Steyskal 1986b: 173; Spencer 1987: 255; Papp 1984: 315 [as synonym of Phytomyza]; Spencer and Martinez 1987: 255 [stat reinst.]; Winkler et al. 2009: 276 [as synonym of Phytomyza]; Papp and Černý 2020: 19; von Tschirnhaus 2021: 105.
Dineura Lioy, 1864: 1315. Type species: Phytomyzafestiva Meigen, 1830 [=PhytomyzaelegansMeigen 1830], by original designation. Preoccupied by Dahlbom (1835) and Selys (1859).
Napomyia . Misspelling. Schiner, 1868: 227.
Phythomyza . Misspelling. Rondani, 1874: 51.
Lonicera . Error for Phytomyza. Meijere 1924: 147 [in describing and naming Phytomyzaluteoscutellata Meijere on page 147, the genus group name of the host (Lonicera) was used for the fly genus; the correct combination is given on pages 126 and 147].
Ptochomyza Hering, 1942: 530. Type species: Ptochomyzaasparagi Hering 1942, by original designation. Winkler et al. 2009: 282 [as subgenus]; Lonsdale 2015: 637.

*Phytomyza* is the most speciose genus of Agromyzidae with ca. 800 species worldwide, and certainly the most diverse with respect to morphology and host use. The classification of the group was developed by Winker et al. (2009) when they synonymized *Chromatomyia*, and included *Napomyza* and the small genus *Ptochomyza* as subgenera, finding that all of these rendered the larger *Phytomyza* paraphyletic. Further discussion is provided in Lonsdale (2015) and Lonsdale and Eiseman (2021).

Species of the subgenusNapomyza occur globally, occurring mostly towards the west in North America, and while none are yet known from the Delmarva states, it is likely that they will eventually be found. Zlobin (1994) discussed the characters used by Spencer to diagnose *Napomyza*, finding them to be plesiomorphic and not sufficient to maintain the genus as a separate entity. Zlobin instead provided tentative characters of the male genitalia, although similar characters are found in some *Phytomyza* s. s. and should be used with caution (Winkler et al. 2009). Species of the subgenusPtochomyza are mostly restricted to the Palaearctic Region; a minority also occur in the Afrotropics, as discussed by Lonsdale (2015).

In previous keys and diagnoses, the length of the fronto-orbital setae and the lengths of the costal sectors were used to differentiate those species of *Phytomyza* with a dark frons. These characters appear to vary much more widely than previously appreciated among many of those species, however, making them unreliable for diagnosis and they are not used in the key provided below. Male dissections should be made whenever possible to verify identifications, as genitalic characters in this genus are among the most complex and distinct of any Agromyzidae.

### ﻿Key to the Delmarva *Phytomyza*

**Table:** 

1	Posterior cross-vein (dm-m) present near base of wing	***P.davisii* (Walton)**
–	Posterior cross-vein absent	2
2	Frons light yellow, as pale or nearly as pale as halter; fronto-orbital plate entirely pale, without any dark markings around or anterior to posterior fronto-orbital	3
–	Frons brown, distinctly darker than halter; in questionable cases, fronto-orbital plate darker along lateral margin or at least dark from posterior margin of frons to base of hind fronto-orbital	**13**
3	First flagellomere slightly to strongly enlarged, especially in female, and covered with elongate hairs at least as long as several times the width of the base of the arista. Hypophallus U-shaped with bases parallel. Distiphallus simple, short, narrow, tubular (Figs 803, 804)	***P.lactuca* Frost**
–	First flagellomere sometimes enlarged, but hairs usually inconspicuous, and never as long as above. If male genitalia as above (*P.aquilegivora*), then hypophallus very large and distiphallus bent backwards	4
4	Four pairs of fronto-orbital setae, with anterior seta sometimes reduced in length. Wing length 2.2–3.8 mm (rarely less than 2.5 mm)	5
–	Two or three pairs of fronto-orbital setae. Wing length 1.6–2.8 mm	9
5	Two or more rows of acrostichal setulae. Notopleuron and postpronotum dark to slightly yellowish. Pleuron entirely dark. Femora dark with knees yellow. Hypophallus membranous	6
–	Two or no rows of acrostichal setulae. Notopleuron and postpronotum pale yellow, sometimes with dark mottling. Pleuron with extensive yellow patches. Femora predominantly yellow. Hypophallus with complete transverse U-shaped bar	8
6	Pedicel yellow to brownish. Frons distinctly projecting above eye. Wing length 2.8–3.3 mm. One pair of long, dark sclerotised paraphalli converging on single weakly sclerotised apical tube of distiphallus that has dark subapical band (Figs 755, 756)	***P.chelonei* Spencer**
–	Pedicel black. Frons narrowly visible above eye (seen laterally). Wing length 2.2–2.8 mm. Paraphallus variable. Distiphallus bifid	7
7	Four or more rows of acrostichal setulae. Tarsi yellow, becoming darker apically. Distiphallus with two separate tubules. Basiphallus composed of two separate sclerites (Figs 739, 740)	***P.aquilegiana* Frost**
–	Two rows of acrostichal setulae. Tarsi entirely brown. Distiphallus short and subcylindrical with narrow inner process. Basiphallus T-shaped (Figs 746, 747)	***P.avicursa* sp. nov.**
8	Two rows of acrostichal setulae. Smaller medial presutural supra-alar seta present. Palpus small and narrow. Ocellar spot small, restricted to tubercle. Scutellum yellow with brownish spots. Male abdomen entirely yellow (Fig. 132); female tergites 1–4 brownish in part and oviscape brown, sometimes with dorsum yellow. Wing length 2.9–3.8 mm. Hypophallus relatively small and rounded (Fig. 763)	***P.compta* (Spencer)**
–	No acrostichal setulae. Only a single presutural supra-alar seta present. Palpus broadly ovate, nearly as wide as long. Large circular spot surrounding ocellar tubercle. Scutellum brown (Fig. 133). Abdomen brown. Wing length 2.2–2.8 mm. Hypophallus large and subquadrate, with additional pair of sclerotised plates present beneath (Figs 809, 810)	***P.nervosa* Loew**
9	One notopleural seta (posterior seta absent). Basiphallus with spinulose ventromedial patch. Basiphallus with one pair of large lateral sclerites as well as several smaller interlocking dorsomedial. Paraphallus C-shaped, distal 1/2 flanking small tubular distiphallus (Figs 817, 818)	***P.plantaginis* Robineau-Desvoidy**
–	Both notopleural setae present. Phallus not as above	**10**
10	Arista laterally compressed and broad (Fig. 134). Epandrium large and bulbous, with length more than 1/2 that of abdomen. Postgonite flattened with broad inner plates. Apices of basiphallus dark, and ventrally arched and expanded; with membranous extension on left medial margin. Distiphallus short, dark and subcylindrical (Figs 769, 770)	***P.crassiseta* Zetterstedt**
–	Arista narrow and circular in cross-section, with base slightly swollen. Male terminalia not as above	**11**
11	Clypeus, palpus, pedicel, epandrium and most of thorax yellow. Scutum yellow with three greyish stripes. Sclerites of hypophallus strongly curved (viewed laterally) with ill-defined distal regions meeting medially. Distiphallus approximately as long as basiphallus, branches nearly parallel, flared apically (Figs 761, 762)	***P.clematiphaga* Spencer**
–	Clypeus, palpus, pedicel, epandrium and most of thorax brown to black. Scutum dark brown with notopleuron, postpronotum, and sometimes supra-alar region yellow. Hypophallus essentially straight (viewed laterally), sclerites separate. Distiphallus not as above	**12**
12	Anterior ori strong. Acrostichal setulae in 2 rows. Scutellum with narrow, sometimes inconspicuous, ill-defined medial stripe. Calypter margin and hairs brown. Paraphalli lobate, extended laterally. Hypophallus long, widest subbasally, apex curved inward. Distiphallus long, tubules strongly divergent at base, approximate distally	***P.aldrichi* Spencer**
–	Anterior ori not more than 2/5 length of posterior ori. Acrostichal setulae in 3 or 4 scattered rows. Scutellum entirely dark. Calypter entirely white. Paraphalli band-like, not flared laterally. Hypophallus small, rod-like, straight. Distiphallus short, tubules strongly diverging	***P.aesculi* Eiseman & Lonsdale**
13	Fronto-orbital plate and notum brilliantly shiny, sharply contrasting remainder of frons. Head entirely dark brown. Notum strongly shiny. Wing length 1.6–2.1 mm. Postgonite spindle-shaped in outline and very large. Paraphalli uniting distally and curved to right. Distiphallus small and Y-shaped, directed dorsally (Figs 806, 807)	***P.loewii* Hendel**
–	Fronto-orbital plate as pruinose as centre of frons. Head colour variable, usually brownish/grey or yellowish medially. Notum matt to subshiny. Wing length variable. Postgonite narrow. Paraphalli variable, but never as above. Distiphallus larger and directed distally	**14**
14	Calypter margin and hairs bright yellow to white. Hypophallus with narrow, produced dorsomedial sclerotisation in addition to lateral sclerites	**15**
–	Calypter margin and hairs grey to brown. Hypophallus not as above	**16**
15	Wing length 3.2–4.1 mm. Three or four ori, one ors. Four rows of acrostichal setulae. All knees distinctly yellow. Paraphallus absent. Mesophallus with thick rounded base. Distiphallus strongly curved dorsally (Figs 823, 824). Ejaculatory apodeme with blade exceptionally broad and rounded	***P.pulchelloides* Henshaw & Howse**
–	Wing length 2.2–2.4 mm. Two ors, two ori. Six rows of acrostichal setulae. Only fore knee with small yellow spot. Paraphallus present. Mesophallus with constricted base. Distiphallus only slightly angled, sinuate (Figs 820, 821). Ejaculatory apodeme with narrow blade	***P.seghali* Spencer**
16	Wing length 3.2–3.8 mm. At least posterior margins of tergites 2–5 yellow, but abdomen usually more extensively yellow (Fig. 138). Two ors, two to four ori; all well-developed. Distiphallus with one pair of very narrow, diverging tubules, mirrored basally by paired paraphalli (Figs 748, 749)	***P.bicolor* Coquillett**
–	Wing length 2.7 mm or less. Abdomen entirely brown. Two ors, often with posterior seta reduced; two ori, often with anterior seta reduced. Phallus not as above	**17**
17	Acrostichal setulae absent or in two sparse medial rows. Dorsocentral setae short and gracile. Female first flagellomere 1/2 height of eye. Frons light yellow with fronto-orbital plate brown. One ors. Hypophallus large, U-shaped (Figs 742–744)	***P.aquilegivora* Spencer**
–	Acrostichal setulae in four or rarely two scattered rows. Dorsocentral setae long and relatively thick. Female first flagellomere not enlarged. Frons and fronto-orbital plate more concolorous. Two ors. Hypophallus with one pair of separate sclerites	**18**
18	Ocellar tubercle ovate; ocelli forming a near equilateral triangle, with posterior ocelli separated by width of two ocelli at most. Thorax subshiny to silvery pruinose. Arms of distiphallus united or nearly so	**19**
–	Ocellar tubercle subrectangular to broadly ovate; posterior ocelli separated by distance of at least three ocelli. Thorax silvery pruinose. Arms of distiphallus long, black, narrow, and curved backwards	**22**
19	Notum subshiny, only with light dusting of pruinosity. Head dark brown, becoming paler immediately around ocellar tubercle. Distiphallus short, linear, very narrow, nearly indistinct (Figs 726, 727). Paraphalli short, finger-like, converging and directed distally	***P.vockerothi* Winkler**
–	Notum with light to very distinct pruinosity. Head mostly light brown/grey. Distiphallus large and distinct, more obvious than paraphalli, which are variable in shape	**20**
20	Pruinosity on notum brownish. Ocellar spot partially, or only barely enclosing anterior ocellus, with region immediately in front of ocellus pale. Wing length 2.1–2.6 mm	***P.osmorhizae* Spencer**
–	Pruinosity on notum silvery. Ocellar spot enclosing anterior ocellus, with region immediately in front of ocellus dark brown, if only marginally. Wing length 1.8–2.2 mm	**21**
21	Two to four irregular rows of acrostichal setulae around transverse suture. Base colour of head yellow; antenna black. Distiphallus long and membranous with apex darker (Figs 831, 832). Paraphalli united, forming flat U-shaped plate below base of distiphallus	***P.winkleri* sp. nov.**
–	Four rows of acrostichal setulae. Base colour of head brown; antenna brown with first flagellomere darker. Distiphallus small, dark, and V-shaped with narrow lateral plate on basal 2/3 (Figs 752, 753). Paraphalli small and separate	***P.catenula* sp. nov.**
22	Fronto-orbital plate entirely dark. First tergite often slightly paler than second. Epandrium evenly rounded. Arms of distiphallus extremely long, nearly forming a complete circle in lateral view (Figs 811, 812)	***P.persicae* Frick**
–	Fronto-orbital plate whitish, at least anteriorly or along inner margin. First and second tergites dark. Epandrium with supra-anal protuberance. Arms of distiphallus not longer than mesophallus	***P.ilicis* group (23)**
23	Wing length 2.2–2.6 mm. Lateral sclerite of hypophallus fused to basiphallus. Mesophallus (seen laterally) barely 2 × longer than wide. Arms of distiphallus fused at base (Figs 782, 783)	***P.glabricola* Kulp**
–	Wing length 1.4–2.2 mm. Lateral sclerite of hypophallus separate from basiphallus. Mesophallus (seen laterally) at least 3 × longer than wide. Arms of distiphallus separate, usually with pigmented spot between bases	**24**
24	Mesophallus arched, long and narrow, with length at least 10 × width at midpoint. Mesophallus and distiphallus broadly separated. Distiphallus relatively short, with base > 2 × its own length from base of mesophallus. Surface of hypophallus well-sclerotised, nearly cup-like, with anterior sclerite less well-defined (Figs 797, 798)	***P.verticillatae* Kulp**
–	Mesophallus relatively straight and stout, with length not > 9 × width at midpoint. Mesophallus and distiphallus closely approximated. Distiphallus relatively long, with base less than 2 ×its own length from base of mesophallus. Hypophallus well-defined and plate-like	**25**
25	Lateral sclerite of hypophallus semi-circular. Mesophallus never swollen on distal 1/2 (Figs 794, 795)	***P.opacae* Kulp**
–	Lateral sclerite of hypophallus narrow along length or clavate (some *P.leslieae* with slight basomedial bulge on sclerite, but shape never semi-circular; in uncertain cases, mesophallus of *P.leslieae* swollen on distal 1/2)	**26**
26	Fronto-orbital plate sometimes entirely dark to level of posterior ori. Lateral sclerite of hypophallus clavate, with apex broad and base narrowed	**27**
–	Fronto-orbital plate, if dark to level of posterior ori, only dark laterally and with oblique medial extensions to base of setae. Lateral sclerite of hypophallus of uniform width along entire length	**28**
27	Epandrial process wider than long (Fig. 778). Lateral sclerite of hypophallus with short basal “stem”, and large, subquadrate apical section. Mesophallus gradually widening apically or as wide at base as at midpoint; rarely swollen at base, but if so, lateral sclerite of hypophallus clearly large and quadrate apically. Distiphallus (seen laterally) strongly widened at base and with apex pointing apically (i.e., sinuate) (Figs 779, 780)	***P.ditmani* Kulp**
–	Epandrial process longer than wide. Lateral sclerite of hypophallus with long, narrow basal section and gradually widening apex. Mesophallus more abruptly widened apically, narrowest medially. Distiphallus (seen laterally) only slightly widened at base and with apex pointing dorsally (Figs 800, 801)	***P.wiggii* Lonsdale & Scheffer**
28	Lateral sclerite of hypophallus < 1/2 width of mesophallus and with margins smooth (Figs 791, 792)	***P.lineata* Lonsdale & Scheffer**
–	Lateral sclerite of hypophallus as wide as mesophallus and with margins ill-defined and/or irregular	**29**
29	Gena and occiput dark along posterior and posterolateral margins of eye; gena not strikingly whiter than frons. Face dark grey laterally. Fore femur dark, sometimes becoming gradually paler at apex. Lateral sclerite of hypophallus nearly 3–4 × longer than wide and very slightly upcurved. Mesophallus constricted and very slender before midpoint	***P.nemopanthi* Griffths & Piercey-Normore**
–	Gena and occiput whitish along posterior and posterolateral margins of eye; gena often noticeably whiter than frons. Face often whitish laterally. Fore femur often yellow apically. Lateral sclerite of hypophallus either 4 × longer than wide and strongly curved or 2 × longer than wide and straight. Mesophallus neither strongly constricted nor slender	**30**
30	Face sometimes dark grey laterally. Lateral sclerite of hypophallus 4 × longer than wide, curved and with distinct edges. Mesophallus slender along length. Arms of distiphallus not strongly produced laterally, largely hidden in ventral view (Figs 785, 786)	***P.ilicicola* Loew**
–	Face always whitish laterally. Lateral sclerite of hypophallus less than 2 × longer than wide, straight and with ill-defined edges. Mesophallus bulging on distal 1/2. Arms of distiphallus strongly produced laterally (Figs 788, 789)	***P.leslieae* Lonsdale & Scheffer**

#### Species descriptions

##### Phytomyza aesculi

Taxon classificationAnimaliaDipteraPhytomyzinae

Eiseman & Lonsdale

Figs 727–731

Phytomyza aesculi Eiseman & Lonsdale, 2018: 66. Eiseman et al. 2021: 32 (females tentatively identified).

###### Description

**(from Eiseman and Lonsdale 2018 and Eiseman et al. 2021).** Wing length 1.9 mm (♂), 2.3–2.5 mm (♀). Vein dm-m absent. Eye height divided by gena height: 2.3–3.1. First flagellomere small, rounded. Arista pubescent. Cheek present, ~ 2/5 height of gena. Posterior ocelli slightly displaced. Vein dm-cu absent. Body pruinose.

***Chaetotaxy***: Two ori (anterior ori thin, setula-like to 2/5 length posterior ori, sometimes absent on one side); one ors. Ocellar and postvertical setae subequal to anterior ors. Four dorsocentral setae decreasing in length anteriorly with anterior two much shorter. Acrostichal setulae in three or four irregular to scattered rows that are clearly separated from dorsocentral rows.

***Colouration***: Setae dark brown. Head mostly light yellow; very narrow brown margin around eye that is thickest ventromedially; antenna, back of head, clypeus, palpus and ventral line on gena dark brown; ocellar spot slightly larger than tubercle, dark brown to black, ovate, wider than long; posterolateral corner of frons dark brown to base of outer vertical seta, brown to light brown to base of inner vertical, sometimes with narrow dark line between base of setae; face with irregular brown colour with yellowish patches. Notum with light brown pruinosity that is denser and greyer on notum. Thorax dark brown with white lateral stripe narrowing posteriorly from postpronotum to supra-alar region (except for large dark spot on postpronotum), scutum with small whitish spot anterolateral to corner of scutellum, posterodorsal region of anatergite whitish, katatergite white with posteroventral region dark brown, meron dorsally white and anepimeron white with anterior 1/2 mostly brown. Wing veins whitish, paler to base. Calypter white. Haltere white. Legs dark brown with light yellow spot on femora apices as long as wide, base and apex of tibiae light yellow, and tarsi light yellow with brown tint and with apical tarsomere light brown; male and one female with mid tibia yellower and fore tibia mostly yellow. Abdomen dark brown.

***Genitalia***: (Figs 727–7﻿31) Surstylus fused to epandrium, small, internally setose. Epandrium with narrow flat plate along posterior margin. Hypandrium well-developed, rounded. Postgonite stout, strongly curved medially, with one medial seta. Phallophorus well-developed. Basiphallus composed of two narrow plates; right plate extending onto dorsum basally; left plate shorter with basal arm wrapping around shaft. Hypophallus divided into two narrow lateral sclerotised bars with lobate extension on anterior margin near base. Paraphallus dark but strongly reduced to one pair of small pointed anteroventral extensions arising from mesophallus. Mesophallus small, cylindrical, laterally bulging distally and tapered basally where it is as wide as duct, fused to distiphallus. Distiphallus divided into one pair of long, narrow tubules, as long as remainder of phallus combined, that are strongly divergent at base (initially perpendicular to long axis of phallus), and converging and slightly curved along most of length. Ejaculatory apodeme well-developed with especially broad blade and base; sperm pump with transverse sclerotised bar that is broadly thickened at lateral margins.

*Variation*: Virginia females differ as follows: face darker, venter of lunule and region around antennal bases blackish, and apices of mid and hind femora dark.

###### Host.

Sapindaceae – *Aesculusglabra*; VA females from *A.flava*, and leaf mines seen on *A. pavia* and *A.sylvatica*.

###### Distribution.

**USA**: OH, VA[tentatively identified female]. Leaf mines from Canada (ON) and AL, AR, GA, IA, KS, MN, MO, NC, PA, SC, VA.

###### Type material.

***Holotype*: USA. OH**: Delaware Co., Sunbury, Monkey Hollow Rd., 6–11.v.2015, em. 9–12.iii.2016, J. A. Blyth, ex *Aesculusglabra*, #CSE2239, CNC654467 (1♂, CNC).

***Paratypes*: USA. OH**: same collection as holotype, CNC654468–654469 (2♀, CNC).

###### Additional material examined.

**USA. VA**: Radford, Wildwood Park, 3.v.2017, em. 8–11.iv.2018, N.V. Kent, ex *Aesculusflava*, #CSE4400, CNC1135662–1135664 (3♀, CNC).

##### Phytomyza aldrichi

Taxon classificationAnimaliaDipteraPhytomyzinae

Spencer

Figs 732–734

Phytomyza aldrichi Spencer in Spencer and Steyskal 1986b: 309; Eiseman and Lonsdale 2018: 67.

###### Description.

Wing length 2.0–2.3 mm (♂), 2.5 mm (♀). Vein dm-m absent. Eye height divided by gena height: 3.1. Cheek short, but deep medially

***Chaetotaxy***: Two ori (posterior ori sometimes appearing as ors); one ors. Two irregular rows of acrostichal setulae, ending medially or near posterior dorsocentral. Very few intra-alar setulae anteriorly. Ocellar and postocellar setae subequal to fronto-orbitals. Four dorsocentral setae, decreasing in length anteriorly. Smaller medial presutural supra-alar present.

***Colouration***: Ocellar tubercle, back of head and palpus brown; clypeus brownish yellow to brown; posterior margin of frons dark brown lateral to base of outer vertical seta, and with faded spot behind base of inner vertical seta that may reach base of one or both verticals; first flagellomere and scape black; remainder of head light yellow (paler below antenna); base of fronto-orbitals sometimes with minute brown spot. Scutum dark brown with greyish pruinosity, with postpronotum, notopleuron and sometimes narrow to wide supra-alar region yellow, with small to large brownish medial spot on postpronotum. Scutellum brown with greyish pruinosity, excluding ill-defined yellow medial stripe that is constricted at midpoint; ON male with stripe nearly absent, most evident apically. Metanotum dark brown with katatergite at least partially yellow. Pleuron variable in colouration, darkest specimens being entirely dark brown with greyish pruinosity with dorsal margin of anepisternum narrowly yellow; paler specimens (ME male palest) with dorsum of katepisternum yellow (including base of seta in posterior mottled region), anepimeron mottled yellow, anepisternum yellowish brown with dorsal ¼ yellow, and posteromedial margin and large anteroventral spot darker brown. Halter white. Calypter margin and hairs brown. Legs variable in colouration, darkest specimens brown with knees broadly yellow; paler specimens with only base of coxae brown, tibiae paler, and sometimes femora with pale mottling. Abdomen dark brown.

***Genitalia***: (Figs 732–734) Hypandrium stout and broadly rounded, with inner lobes narrow, curved, joined medially and with two anterior setae. Postgonite dark, apically hooked, with one seta. Right 1/2 of basiphallus joined to dorsomedial margin of phallophorus; left side with ventrobasal process around shaft; rounded apically, nearly parallel-sided along length. Hypophallus membranous with one pair of flat, clavate laterobasal sclerites with outer margins subparallel. Mesophallus short, with narrow basal stem meeting duct; flanked by one pair of broad, rounded, flat paraphalli. Distiphallus with one pair of very narrow, dark, diverging tubules, subequal to length of mesophallus. Ejaculatory apodeme with stem short, with lateral subbasal swelling and blade broad with margins pointed.

###### Host.

Ranunculaceae – *Ranunculus*.

###### Distribution.

**Canada**: NS*, ON*, QC*. **USA**: ID, MD*, ME, OH.

###### Type material.

***Holotype*: USA. ID**: Moscow, J.M. Aldrich (1♂ [head missing], USNM).

###### Additional material examined.

**Canada. NS**: Truro, 26.ix.1913, CNC480058 (1♂, CNC), **ON**: Marmora, 8.v.1952, J.C. Mitchell, CNC480059 (1♂, CNC), **QC**: Old Chelsea, 11.vi.1959, J.R. Vockeroth, CNC480057 (1♀, CNC). **USA. MD**: Montgomery Co., Colesville, 26.vi.1977, Malaise trap, W.W. Wirth (1♂, USNM), **ME**: Washington Co., Perry, Birch Point, 15.vi.2014, C.S. Eiseman, ex. *Ranunculusrepens* em. 29.vi.2014, #CSE1141, CNC384880 (1♂, CNC), **OH**: Hocking Co., South Bloomingville, Deep Woods Farm, 6.viii.2016, C.S. Eiseman, *Ranunculusrecurvatus*, em. 20.viii.2016, #CSE2927, CNC654479 (1♂, CNC).

###### Comments.

Yellow shoulders and a medially yellow scutellum are diagnostic of this species, as is the short, dark, branched distiphallus and the broad, flat paraphallus.

##### Phytomyza aquilegiana

Taxon classificationAnimaliaDipteraPhytomyzinae

Frost

Figs 735–740

Phytomyza aquilegiae Hardy. Misidentification, in part. Melander 1913: 271.
Phytomyza bipunctata Loew. Misidentification, in part. Melander 1913: 271.
Phytomyza plumiseta Frost. Misidentification, in part (specimens on Aquilegia). Frost 1930: 459.
Phytomyza aquilegiana Frost, 1930: 459. Frick 1959: 424; Spencer 1969: 227; Spencer and Steyskal 1986b: 192; Scheffer and Lonsdale 2018: 88; Eiseman and Lonsdale 2018: 68.

###### Description.

Wing length 2.2–2.8 mm (♂), 2.5–2.8 mm (♀). Vein dm-m absent. Eye height divided by gena height: 3.3–4.5. Hairs on first flagellomere slightly longer and bushier, but not comparable to other species such as *P.lactuca*. Cheek narrow.

***Chaetotaxy***: Two ori (anterior ori slightly to strongly reduced in length); two ors. Acrostichal setulae in four scattered rows that may become more regular anteriorly; sometimes appearing as five rows. Four dorsocentrals, decreasing in length anteriorly.

***Colouration***: Head yellow with antenna black, and back of head and clypeus dark brown; palpus brownish to dark brown; posterolateral corner of frons to base of inner vertical seta dark brown, with spot sometimes faintly continuing narrowly along margin of eye to posterior ors; ocellar triangle dark brown, anterior corner rounded, slightly larger than tubercle, especially posterolaterally where corners bulge outwards to almost touch dark spot at base of vertical setae; face faintly brownish. Thorax dark brown with light dusting of brownish grey pruinosity (less so on pleuron); sometimes indistinct yellowish mottling on postpronotum and notopleuron along suture. Halter white. Calypter white with hairs brown. Legs dark brown with apex of femora yellow, tibiae yellowish to base and apex, and tarsi yellow with distal segments becoming darker. Abdomen dark brown.

***Genitalia***: (Figs 735–740) Hypandrium well-developed, thickest at apex, inner lobe with projecting medial spinulose membrane and several empty sockets. Postgonite broad and pointed apically with one seta and two sockets. Halves of basiphallus narrow, separate; left sclerite abruptly S-shaped at base, right sclerite with wide basal extension. Hypophallus with one pair of short, narrow converging sclerites. Paraphalli converging, small, with rounded irregular base and narrow apical process. Mesophallus tubular, lightly sclerotised, apically widened to join distiphallus; with small dorsobasal band across surface. Distiphallus divided into two dark, slightly twisted arms that are shorter than mesophallus. Ejaculatory apodeme as long as wide, with stem and base short and narrow, blade becoming clear to distal margin.

###### Hosts.

Ranunculaceae – *Aquilegia*, *Ranunculus* (Benavent-Corai et al. 2005).

###### Distribution.

**Canada**: AB, BC, MB, ON, YT. **USA**: MA, MD, NC, NY, PA, VA*.

###### Type material.

***Holotype*: USA. PA**: Arendteville, 3.v.1928, S.W. Frost, blotch mine *A.vulgaris* (1♂, USNM, type No. 50023).

###### Paratypes examined.

**USA. PA**: Arendteville, S.W. Frost, blotch mine *A.canadensis* (1♂, USNM), Arendtville, S.W. Frost, meadow rue (2♀, USNM), Arendteville, S.W. Frost, blotch mine, *Aq.vulgaris*, 8.v.1928 (1♀, USNM), 2.v.1928 (1♀, USNM).

###### Additional material examined.

**Canada. MB**: Aweme, 1.v.1930, R.M. White, Columbine leaf miner CNC480062, CNC480060 (1♂,1♀, CNC), **ON**: Ottawa, damp second growth Acer-Betula wood, 13.viii.1991, J.R. Vockeroth, CNC480065 (1♂, CNC), 2.viii.1993, CNC480064 (1♂, CNC), 6.viii.1992, CNC480066 (1♂, CNC), 7.viii.2000, CNC480067 (1♂, CNC), St. Lawrence Is. Nat. Park, McDonald Is., 7.vii.1976, A. Carter, Code 4352-L, CNC480061 (1♂, CNC). **USA. CA**: SL Obispo, I.J. Condit, 13.i.1912, bred from *Aquilegia* (5♂ 2♀, USNM), 13.i.1912 (6♂ 2♀, USNM), Los Angeles Co., Westwood Hills, 19.x.1940, ex. *Columbina*, R.M. Bohart (2♀, USNM), **MA**: Hampshire Co., Pelham, 88 Arnold Rd., 31.v.2012, em. 25.v.2012, C.S. Eiseman, ex blotch mine on *Aquilegiavulgaris* (2♂[with puparia] 1♀, CNC), **MD**: Montgomery Co., Colesville, W.W. Wirth, 21.v.1977 (1♂, USNM), 4.vi.1977 (1♀, USNM), 1.v.1977 (1♂ 1♀, USNM), Malaise trap, Bethseda, 22.vii.1972, G.C. Steyskal (1♂, USNM), **ME**: bred, Bar Harbor, 8.viii.1938, A.E. Brower (1♂ 3♀, USNM), **NC**: Durham Co., Durham, Pelham Rd., 21.v.2015, T.S. Feldman, *Aquilegia*, em. 6.vi.2015, #CSE1623, CNC564653–564656;CNC564657 (1♂ 4♀, CNC), **VA**: Shenandoah, Big Meadows, A.L. Melander, 15.vi.1941 (1♂, USNM), 1.vii.1939 (1♀, USNM). **Location unknown.** miner *Aquilegia* CNC480063 (6♂/♀ [same pin], CNC).

##### Phytomyza aquilegivora

Taxon classificationAnimaliaDipteraPhytomyzinae

Spencer

Figs 741–744

Phytomyza aquilegivora Spencer, 1969: 229. Spencer and Steyskal 1986b: 198; Scheffer and Lonsdale 2018: 88; Eiseman and Lonsdale 2018: 68.
Phytomyza aquilegiae Hardy. Misidentification. Cory 1916: 419.

###### Description.

Wing length 1.5–1.7 mm (♂), 1.7–2.0 mm (♀). Vein dm-m absent. Eye height divided by gena height: 2.4–3.8. Female first flagellomere large, rounded, 1/2 height of eye; male first flagellomere slightly larger than that of sibling species, as high or higher than long. Fronto-orbital plate well-sclerotised, shiny and clearly delimited, slightly widened, more evident posteriorly; soft medial portion of frons wider than long behind relatively broad, deep lunule.

***Chaetotaxy***: Setae short and gracile, particularly on notum. One ori, sometimes with smaller anterior seta; one longer medial ors. Orbital setulae very few in number, slightly longer than average. Postocellar and ocellar setae nearly as long as, and slightly thinner than ori. Acrostichal setulae in two sparse rows or absent. Four dorsocentral setae, decreasing in length anteriorly.

***Colouration***: Head dirty yellow with tubercle, back of head, posterior region of postgena, clypeus, palpus, ventral margin of gena, fronto-orbital plate and ring around eye including parafacial and cheek dark brown; lunule usually brownish to brown, anteromedial region of frons sometimes browner; antenna dark brown with first flagellomere nearly black; face brown; gena usually brownish or distinctly brown. Thorax dark brown, subshiny. Halter white. Calypter margin brownish, hairs brown. Legs brown with apex of femora yellow for a distance sometimes greater than width of femur apex. Abdomen brown. Female slightly darker.

***Genitalia***: (Figs 741–744) Hypandrium broadly arched; inner lobe short and broad with three setae. Postgonite long, upcurved and cleft apically, with one seta. Halves of basiphallus relatively short, as long as enlarged hypophallus; crossing near base, strongly diverging apically to base of hypophallus. Mesophallus indistinct. Distiphallus narrow, tubular, lightly pigmented, apex swollen, angled dorsally, as long as basiphallus. Ejaculatory apodeme small, not much longer than wide, with much of sperm pump and base of duct pigmented.

###### Host.

Ranunculaceae – *Aquilegia*.

###### Distribution.

**Canada**: AB, ON. **USA**: CA, CT, DC, DE*, IL, MA, MD*, NC, NY.

###### Type material.

***Holotype*: Canada. ON**: Pelee, em. 25.ix.1967, ex. leaf mine on *Aquilegiacanadensis*, leg. 15.vii.1967, K.A. Spencer (1♂, CNC).

###### Additional material examined.

**Canada. ON**: Ancaster, 26.vi.1955, O. Peck, CNC480072 (1♀, CNC), Ottawa, Central Experimental Farm, 31.v.2013, O. Lonsdale, leaf mine *Aquilegia* CNC454640-CNC454659 (10♂ 10♀, CNC), Constance Bay, 31.v.2013, O. Lonsdale, leaf miner on *Aquilegia* sp., CNC799378 (1♂, CNC). **USA. CT**: Newhaven, 2.ix.1984, C398 (1♂, USNM), 29.ix.1894 (1♂ 1♀, USNM), **DC**: Washington, 14.v.1915, from columbine, R.L. Woglum (3♂ 8♀, USNM), **DE**: Newark, 15.vi.1979, R.M. Hendrickson, Jr., reared ex. *Aquilegia* sp. (6♂ 4♀, USNM), **IN**: Lafayette, 28.ix.1916, Satterthwait coll., reared from leaf miner, Cage No. C862b (1♂, USNM), **MA**: Hampshire Co., Pelham, 88 Arnold Rd., 25.v.2012, em. 4.vii.2012, C.S. Eiseman, ex linear mine *Aquilegiavulgaris* (1♂, CNC), **MD**: Montgomery Co., Bethseda, v.1972, ex. cwt *Aquilegia* (9♂ 6♀ [with puparia and chalcidoid], USNM), “May 26”, “Cory 1914, bred from columbine“, “exp. No. [illegible] (1♂, USNM), **NY**: Ithaca, reared from columbine, S.W. Frost, Lot A-25, sub 5 (1?, USNM). **Location Unknown.** mine in Aquilegia CNC480071 (7♂/♀, CNC), ILLR (1♀, USNM).

###### Comments.

Spencer (1969) suspected that Frick’s (1959) eastern specimens of the Holarctic species *Phytomyzaminuscula* Goureau were actually *P.aquilegivora*, thereby reducing *P.minuscula*’s North American range to the western United States until Eiseman and Lonsdale (2018) recorded it from Vermont. The material examined by Frick (originally USNM, but noted as still being “on loan” from 4.i.1956) cannot be located to verify their identity. *Phytomyzaminuscula* differs from this species in having a medially brownish (not yellow) frons and an apically curved (not straight) distiphallus. The USNM specimen collected by Cory in 1914 appears to refer to the species discussed by Cory (1916) as P. *aquilegiae*, which is here therefore considered a misidentification, at least in part; the state is not listed on the label, but derived from the publication.

##### Phytomyza avicursa sp. nov.

Taxon classificationAnimaliaDipteraPhytomyzinae

http://zoobank.org/21D6EB7D-FBBC-405B-9E01-01DAC234809C

Figs 745–747

###### Description.

Wing length 2.4 mm. Vein dm-m absent. Eye height divided by gena height: 3.0. First flagellomere slightly enlarged and subrectangular, thick. Border of fronto-orbital plate not evident. Cheek distinct, deepest part of ring around eye, but anterior region of fronto-orbital plate also clearly visible laterally.

***Chaetotaxy***: Two ori on one side and one on the other; two ors. Four dorsocentrals, decreasing in height anteriorly with anterior seta nearly 1/2 length of posterior seta. Acrostichal setulae in two irregular rows to level of second dorsocentral that sometimes appear as three rows. Very few scattered intra-alar setulae.

***Colouration***: Head light yellow with back of head, ocellar spot, posterolateral corner of frons (enclosing base of vertical setae), antenna, palpus and clypeus dark brown; ocellar spot small but relatively broad, subquadrate. Thorax dark brown with greyish pruinosity. Wing veins light brown to brown. Halter white. Calypter margin white with hairs light brown. Legs dark brown with apices of femora light yellow for length less than apical width of femora, more faded on mid and hind legs. Abdomen dark brown.

***Genitalia***: (Figs 745–747) Hypandrial lobe narrow and transverse, following inner margin of postgonite. Postgonite large, strongly hooked anteriorly, broadly flattened behind apex and with one outer seta. Phallophorus fused to T-shaped basiphallus. Hypophallus membranous and with two overlapping transverse lobes. Paraphalli made up of two flattened, widely spaced plates that are perpendicular to basiphallus. Mesophallus not evident. Distiphallus shorter than paraphallus, cylindrical, slightly compressed dorsoventrally, with base widened in ventral view and narrower in profile; apex clear, membranous, enclosing inner-ventral sclerotisation.

###### Host.

Unknown.

###### Distribution.

**USA**: VA.

###### Etymology.

The specific name compounds the Latin for bird (*avis*) and run (*cursus*), referring to the type locality.

###### Type material.

***Holotype*: USA. VA**: Fairfax Co., Turkey Run Park, 0.3 km W mouth Turkey Run, 38°58'N, 77°09.6'W, Malaise trap, river, 14–17.v.2006, D.R. Smith (1♂, USNM).

###### Comments.

While unremarkable externally, the elongate, T-shaped, and unusual flat basiphallus and short distiphallus of *Phytomyzaavicursa* are highly unique and easily characterise this new species, which is currently known only from the holotype male. Also see comments for *P.winkleri*.

##### Phytomyza bicolor

Taxon classificationAnimaliaDipteraPhytomyzinae

Coquillett

Figs 138 , 748–750

Phytomyza bicolor Coquillett, 1902: 191. Frick 1959: 426; Spencer and Steyskal 1986b: 203.

###### Description

**(Fig. 138).** Wing length 3.2–3.5 mm (♂), 3.4–3.8 (♀). Vein dm-m absent. Eye height divided by gena height: 2.1–2.8. Eye relatively small and round; parafacial and fronto-orbital plate produced dorsally, visible laterally. Labellum relatively long and pointed. With faint brown pruinosity that is denser dorsally on head and thorax.

***Chaetotaxy***: Two to four ori; two ors (anterior seta inset). Ocellar and postocellar setae exceeding length of ors. Four dorsocentrals, one presutural, decreasing in height anteriorly. Up to six scattered rows of acrostichal setulae, strongly reduced in number posterior to second dorsocentral.

***Colouration***: Head light brown to yellowish with palpus brown, ocellar tubercle, posterior margin of frons, lateral margin of frons (stripe barely touching base of fronto-orbitals, disappearing anteriorly), clypeus, venter of gena, face, first flagellomere, lunule (at least widely above antennal bases) and back of head dark brown; gena and frons sometimes browner. Thorax dark brown with postpronotum and notopleuron sometimes reddish. Calypter white with hairs dark brown. Halter white. Legs dark brown. Female abdomen yellow with oviscape dark brown and anterior margin of tergite 6 (and sometimes 4 and 5) brownish; male abdomen as described for female except tergite six, sternite 8 and epandrium brownish; sometimes abdomen brown past tergite 3 or anterior 1/2 of tergite 3 brown and tergites 1 and 2 brownish, but posterior margins of tergites 2–5 always yellow.

***Genitalia***: (Figs 748–750) Inner lobe of hypandrium with separate, elongate medial process. Postgonite well-developed. Basiphallus halves narrow, flat, dorsal, left sclerite longer and fused to phallophorus. Hypophallus membranous. Paraphalli dark, narrow, converging to base of distiphallus; wider and paler basally. Mesophallus short, pigmented, slightly wider than duct, widening to distiphallus. Distiphallus made up of one pair of dark, very narrow tubules that diverge basally and are nearly parallel on distal 1/2. Ejaculatory apodeme very small with distal 1/2 of blade pale to clear; sperm pump with small, sclerotised patch.

###### Host.

Unknown – adult ON female photographed on *Calthapalustris* (Ranunculaceae).

###### Distribution.

**Canada.** ON*. **USA.** CT*, IN, NY, TN*, VA.

###### Type material.

***Holotype*: USA. NY**: Niagra Falls, 23.vi (1♀, USNM, type No. 6663).

###### Additional material examined.

**Canada. ON**: Spring Creek, 10.vi.2007 [1♀, photo voucher by S.A. Marshall]. **USA. CT**: Stanford, 18.v.1919, A.H. Sturtevant (2♂ 1♀, USNM), **NY**: Ithaca, 3.vi.1917, S.H. Emerson (1♂, USNM), Ithaca, 11.vi.1935, H.K. Townes (1♀, USNM), Remus Pt., Chautauqua Lake, 31.v.1963, swampy woods, W.W. Wirth (2♂, USNM), Allegany State Park, 28.v-3.v.1963, stream margin, W.W. Wirth (1♀, USNM), **TN**: Sevier Co., Rte. 441, 3mi NW NC/TN border, Great Smokey Mtn. N.P., 35°38.3'N, 83°27.8'W, 4500', 27.v.1999, S.D. Gaimari (1♀, USNM), **VA**: Shenandoah, Big Meadows, 15.vi.1941, A.L. Melander (3♂, USNM), Fairfax Co., Turkey Run Park, nr. Mouth of Turkey Run, 38°57.9'N, 7°09.4'W, Malaise trap, 18–30.v.2007, D.R. Smith (1♀, USNM), Smyth Co., Mt. Rodgers, 1432–1615 m, 1.v.1962, J.R. Vockeroth, CNC480074 (1♀, CNC).

###### Comments.

The pale abdominal tergites of *Phytomyzabicolor* are highly diagnostic, making this species easy to identify externally. The photo voucher included here (Fig. 138) is the first record of this species in Canada.

##### Phytomyza catenula sp. nov.

Taxon classificationAnimaliaDipteraPhytomyzinae

http://zoobank.org/5196AFA9-45BA-44ED-BDEF-F6EB69033DA5

Figs 751–753

###### Description.

Wing length 1.9 mm (♂). Female unknown. Vein dm-m absent. Eye height divided by gena height: 3.7. First flagellomere ovate, slightly longer than high. Inner margin of fronto-orbital plate not evident, but slightly more sclerotised and raised at and lateral to base of fronto-orbitals. Cheek present.

***Chaetotaxy***: Two ori (anterior seta 2/3 length); two ors. Four dorsocentrals, decreasing in height anteriorly, with anterior three pairs slightly thinner than posterior pair and more equal in length. Acrostichal setulae in four scattered rows. Intra-alar region with moderate presutural setulae and only two postsutural setulae.

***Colouration***: Head brown with antenna, first flagellomere, posterior margin of frons, posterior 1/2 of fronto-orbital plate from second ors, back of head, clypeus, palpus and ventral margin of gena dark brown; anterior 1/2 of frons slightly darker; ocellar tubercle very dark, broadly triangular with rounded corners, not touching back of head, leaving postocellars on paler ground. Notum dark brown with moderate brownish silver pruinosity; notopleuron slightly paler. Wing veins brown. Halter white. Calypter margin yellow and hairs light brown. Legs dark brown with apices of femora yellowish.

***Genitalia***: (Figs 751–753) Hypandrium short and thick with broad haired membrane attached to small setose inner lobe. Postgonite emarginate on inner-distal margin and with one seta. Sclerites of basiphallus long and narrow with bases overlapping and interlocking. Sclerite of hypophallus small, pale, flat, separate, in line with basiphallus. Mesophallus and paraphallus not evident. Distiphallus narrow and flat, apically emarginate, dorsally angled and with dark V-shaped sclerotisation; sides with narrow flared lateral wing. Ejaculatory apodeme short, narrow and dark.

###### Host.

Unknown – likely Caprifoliaceae.

###### Distribution.

**USA**: VA.

###### Etymology.

The specific epithet is Latin for “little chain”, referring to this species’ size and type locality.

###### Type material.

***Holotype*: USA. VA**: Chain Bridge, 14.v.1924, J.R. Malloch (1♂, USNM).

###### Comments.

Based on the structure of the male terminalia, *Phytomyzacatenula* can be placed within a lineage of morphologically similar Caprifoliaceae-feeding *Phytomyza* that was revised by Griffiths in 1974 (as *Chromatomyia*), with additional species described by Eiseman and Lonsdale (2018) and Eiseman et al. (2021). Within this group, the new species is similar in external and genitalic morphology to three western species that have the apex of the basiphallus exceeding the base of the distiphallus (“supporting sclerite complex” of Griffiths 1974): *P.caprifoliae* Spencer (AB), *P.fricki* (Griffiths) (CA, ID, WA, WY) and *P.linnaeae* (Griffiths) (AB, YT, OR). *Phytomyzacaprifoliae* differs in having the distiphallus evenly thick along its length when viewed in profile, and in ventral view, the distiphallus is smooth laterally, more angulate posterolaterally and distally, and it has a narrower stem on a discrete Y-shaped sclerotisation; furthermore, the hypophallus sclerite is larger, longer, darker and better defined (Griffiths 1974: figs 26, 27). The latter two species are highly similar, and like the new species, have basal and subapical lobes on the distiphallus laterally, but both also have a better defined hypophallus, the distiphallus is slightly angled distally (not basally), the outline and medial pattern of sclerotisation on the distiphallus differs (Griffiths 1974: figs 29–32), and *P.fricki* is slightly smaller.

##### Phytomyza chelonei

Taxon classificationAnimaliaDipteraPhytomyzinae

Spencer

Figs 24 , 25 , 754–756

Phytomyza chelonei Spencer, 1969: 234. Spencer and Steyskal 1986b: 312.

###### Description.

Wing length 3.1–3.5 mm (♂), 3.6 mm (♀). Vein dm-m absent. Eye height divided by gena height: 2.4–3.6. Fronto-orbital plate slightly projecting. Parafacial narrow; cheek distinct, at least as high as ventral region of gena.

***Chaetotaxy***: Two ori; two ors. Four dorsocentrals, one presutural, decreasing in height anteriorly. Four to five sparse, scattered rows of acrostichal setulae that nearly reach posterior margin of scutum.

***Colouration***: Head mostly pale yellow; antenna light brown to brown with first flagellomere dark brown; ocellar triangle not distinct, but brown to dark brown spot slightly larger than tubercle, bulging anterolaterally to sometimes form a rounded square; posterolateral margin of frons brown, spot reaching base of inner vertical seta and extending slightly anteriorly, but not reaching base of fronto-orbitals; clypeus dark yellow to dark brown; palpus light to dark brown; back of head dark brown. Thorax dark brown with dense grey pruinosity that is strongest dorsally; remainder of body with faint pruinosity evident on pigmented regions; similar whitish pruinosity along lateral margin of frons (as seen in some *Phytoliriomyza*). Halter white. Calypter entirely whitish yellow. Legs brown with bases of tibiae narrowly yellow and apices of femora yellow for length nearly equal to width of femur apex. Abdomen brown, sometimes with posterior margin of tergites yellow (holotype with most of first and second tergites yellow with wide brownish medial spot).

***Genitalia***: (Figs 754–756) Inner lobe of hypandrium with one seta and broadly membranous, with membrane spreading around inner margin of hypandrium. Postgonite long, broad and mostly flat with large outer lobe and folded inner layer. Basiphallus Y-shaped with short apical split and longer dorsomedial plate that is irregular to right side and better sclerotised along left. Paraphallus narrow, dark, and sinuate with small pigmented spot below base. Mesophallus shorter than paraphallus, clear, and laterally compressed, being only broader at rounded base (base tapered when viewed laterally). Distiphallus as high as mesophallus, appearing bifid ventrally but fused along length, and with narrow transverse pigmented band basally; “halves” of distiphallus straight, as in HT, or V-shaped, as figured. Ejaculatory apodeme short, barely widened apically, and with stout base; sperm pump without ventral sclerotisation.

###### Host.

Plantaginaceae – *Cheloneglabra*.

###### Distribution.

**Canada.** QC. **USA**: IN, NC, TN.

###### Type material.

***Holotype*: Canada. QC**: Old Chelsea, em. 18.vii.1965, ex. fruit of *Cheloneglabra*, J.R. Vockeroth (1♂, CNC, type No. 10435).

###### Additional material examined.

**Canada. QC**: Old Chelsea, 18.vii.1965, J.R. Vockeroth, ex fruit Cheloneglabra, CNC480075 (1 puparium [gel capsule], CNC). **USA. NC**: Mt. Mitchell, 6800', 12.viii.1957, J.G. Chillcott (1♂, USNM), Mt. Mitchell, 2072 m, 12.viii.1957, J.G. Chillcott, CNC480076 (1♂, CNC), Highlands, 1158 m, 21.viii.1957, J.G. Chillcott, CNC480078 (1♀, CNC), **TN**: Great Smoky Mountains N.P., Indian Gap to Clingman’s Dome, 5200–6600', 6.viii.1957, J.G. Chillcott (1♂, USNM), Gr. Sm. Mt. Nat. Park, Indian Gap to Clingman’s Dome, 1584–2011 m, 6.viii.1957, J.G. Chillcott, CNC480077 (1♂, CNC).

###### Comments.

The male terminalia of *Phytomyzachelonei* are similar to those of *P.osmorhizae*, in that the distiphallus is clear and membranous with a pigmented subapical band, but the terminalia are otherwise different, including the presence of a sclerotised hypophallus in the latter. *Phytomyzaosmorhizae* is also smaller (wing length 2.1–2.4 mm) and darker with fewer than four fronto-orbitals.

##### Phytomyza clematiphaga

Taxon classificationAnimaliaDipteraPhytomyzinae

Spencer

Figs 760–762

Phytomyza clematiphaga Spencer, 1969: 236. Sehgal 1971b: 361; Spencer and Steyskal 1986b: 176; Eiseman and Lonsdale 2018: 69.

###### Description.

Wing length 2.0–2.4 mm (♂), 2.4–2.5 mm (♀). Vein dm-m absent. Eye height divided by gena height: 2.3–3.6. First flagellomere relatively large, broad and longer than high, with shape subcircular to more subrectangular; length at most 1/3 more than height; hairs on most of segment elongate and sometimes bushy (more so towards anterior margin), with length usually not exceeding width of base of arista. Cheek distinct.

***Chaetotaxy***: One ori; two ors. Postocellar and ocellar setae subequal to fronto-orbitals. Four dorsocentrals decreasing in size anteriorly. Two rows of acrostichal setulae.

***Colouration***: Setae brown with pale shine (not dark brown to black). Head mostly light yellow; ocellar tubercle brown medially with spot extending to ocelli; fronto-orbital plate to posterolateral corner of frons around vertical setae whitish, faintly white pruinose; back of head with dark brown spot above foramen (not reaching margin); first flagellomere black; palpus brownish to brown or yellow, sometimes only darker apically. Notum light yellow with wide medial stripe between dorsocentral rows on anterior 2/3, one pair of floating intra-alar stripes near centre, and sometimes one pair of narrow, sometimes curved, postsutural supra-alar stripes; grey pruinosity evident on darker regions. Metanotum light yellow with mediotergite and venter of anatergite dark brown with greyish pruinosity. Pleuron mostly light yellow with faint grey pruinosity evident on pigmented regions; anepisternum with nearly indistinct, narrow, irregular anteroventral stripe; katepisternum with dark ventral spot that does not reach base of seta; meron mostly brown. Halter white. Calypter white. Legs yellow with distal segments of tarsi becoming more brown or brownish apically. Abdomen yellow (excluding oviscape), with dorsum brownish anterior to terminalia in male; posterior margin of tergites yellow.

***Genitalia***: (Figs 760–762) Hypandrium with two setae and haired membrane projecting from inner lobe. Postgonite cleft apically and with several outer subapical sockets and one seta. Epiphallus with broad, flat lateral arm. Halves of basiphallus broad and interlocking at base, with strong ventrobasal arm on left 1/2. Sclerotized halves of hypophallus broadly arched and well-sclerotised with apices broad and narrow, nearly meeting medially; basal 1/2 curved, stouter and darker, distal 1/2 paler, similarly curved and directed downwards at apex. Mesophallus rounded at base, longer than wide, dark and subcylindrical, fused to distiphallus; mesophallus + distiphallus as long as basiphallus. Distiphallus dark, entirely split into one pair of narrow tubules that are slightly flared apically. Ejaculatory apodeme with base and margin closest to duct dark with remainder pale to clear; sperm pump dark ventrally.

***Variation***: MD males differs as follows: notal stripes fused into one large irregular spot; side of scutellum brownish; tibiae and tarsi brownish; eye height divided by gena height: 2.6; wing length 1.9 mm.

###### Hosts.

Ranunculaceae – *Clematis* sp., *C.columbiana*, *C.verticillaris*.

###### Distribution.

**Canada**: AB. **USA**: CO, MD*, MT.

###### Type material.

***Holotype*: Canada. AB**: Edmonton, em. 7.viii from leaf mine on *Clematis* sp., leg. 26.vii.1966, B. Hocking (1♂, CNC).

###### Paratypes examined.

**USA. MO**: 15 mi NE Polson, 26.vii.1967, Note No. 6702, S. Whitney (2♀, USNM).

###### Additional material examined.

**USA. CO**: Fremont Co., Bear Creek, Forest Rd 101, off of 49 Rd, 7.vii.2015, C.S. Eiseman, *Clematis*, em. 19–22.vii.2015, #CSE1728, CNC654335, CNC654336 (1♂,1♀, CNC), **MD**: Colesville, W.W. Wirth, 14.vi.1977 (1♀, USNM), 18.vi.1977 (1♂, USNM), 28.v.1977 (3♂, USNM).

###### Comments.

*Phytomyzaclematiphaga* is here recorded for the first time in eastern North America in Maryland, with males and females that are slightly darker than their western counterparts. The species *P.compta* is also brightly coloured, but it has a much larger wing (2.8–3.8 mm); the first flagellomere is more densely long-haired; the calypter hairs, tibiae and tarsi are darker; the notum is darker, although the scutum sometimes maintains a similar, but more extensive vittate pattern; and the hypophallus and distiphallus differ.

##### Phytomyza compta

Taxon classificationAnimaliaDipteraPhytomyzinae

(Spencer)

Figs 132 , 763–765

Chromatomyia compta Spencer in Spencer and Steyskal 1986b: 324.
Phytomyza compta . Winkler et al., 2009: 290.

###### Description

**(Fig. 132).** Wing length 2.9–3.3 mm (♂), 3.0–3.8 mm (♀). Vein dm-m absent. Eye height divided by gena height: 2.7–3.3. Hairs on first flagellomere long, bushy; segment rounded, longer than high, narrowing or wider apically, sometimes appearing subrectangular. Cheek distinct.

***Chaetotaxy***: Two ori with anterior seta shorter and much thinner (not more than 3/5 length posterior ori), sometimes absent on one side; two ors. Two rows of acrostichal setulae. Four subequal dorsocentral setae. Smaller medial presutural supra-alar present.

***Colouration***: Overall colour yellow, more faintly greyish on pleuron; greyish pruinosity evident on pigmented regions, densest dorsally on thorax. Lunule, fronto-orbital plate and posterolateral corner of frons light yellow; ocellar tubercle brown medially with extensions to ocelli; frons posterior to postocellar yellow; back of head brown above foramen; first flagellomere black; palpus brown with base variably yellow. Scutum dark brown with postpronotum, notopleuron, anterior margin beside postpronotum to dorsocentral row, lateral and posterolateral margins to corner of scutellum yellow; sometimes with regions anterior to scutellum between dorsocentrals yellowish; if posteromedial region more clearly yellow, then pattern partially vittate, with posterolateral supra-alar stripe partially differentiated and with nearly indistinct yellow line along dorsocentral row, thereby appearing as a more widely infuscated *P.clematiphaga*. Scutellum yellow with brownish lateral and anteromedial spots. Pleuron yellow with faded brownish anteroventral stripe on anepisternum sometimes evident, meron brown ventrally, katepisternum with large spot not reaching base of seta, and anepimeron sometimes with brownish mottling. Mediotergite dark brown, anatergite brown, katatergite yellow with posteroventral corner brown. Halter white. Calypter white with hairs brown. Tibiae brown to brownish yellow and tarsi brown. Male abdomen yellow; female abdomen brownish dorsally or with reduced pigment medially on tergites 1 and 2 and anteriorly on tergites 3–5; oviscape brown laterally, posteriorly, and sometimes dorsally, but always yellowish or with yellow mottling.

***Genitalia***: (Figs 763–765) Hypandrium short, stout and broadly rounded with narrower, projecting apex; inner lobe with two setae and haired membranous extension. Postgonite broad and relatively flat with one outer seta. Halves of basiphallus narrow, interlocking at base, with dorsal subapical extension nearly reaching paraphallus, and with small, pale ventroapical extension. Hypophallus U-shaped with arms subparallel; basally with tubular membranous chamber with one pair of ill-defined sclerotised strips flanking it where it differentiates into a free, upcurved flagellum. Paraphalli dark, ~ 3 × longer than wide, connected by membrane along venter, covering base of distiphallus; angled dorsally. Ejaculatory duct sclerotised subapically, meeting small, ill-defined, and enclosed mesophallus. Distiphallus entirely split into one pair of diverging tubules ca. as long as hypophallus that are angled towards base of phallus; tubules sclerotised on distal 1/2, but with slightly longer sclerotised ventral extension basally; single rod-like sclerite floating between arms of distiphallus. Ejaculatory apodeme large, clear to blade margin and with venter of sperm pump dark.

###### Host.

Unknown – possibly *Clematis* (Spencer and Steyskal 1986b).

###### Distribution.

**Canada**: ON*. **USA**: ME*, NC, NY*, TN, VA, WA*.

###### Type material.

***Holotype*: USA. NC**: Swain Co., Great Smoky Mts. N.P., Clingman’s Dome, 6300–6642ft, 20.v.1957, J.R. Vockeroth (1♂, CNC).

###### Paratypes examined.

**USA. NC**: Gr. Sm. Mt. Nat. Park, Clingman’s Dome, 6.viii.1957, C.J. Durden, CNC480088 (1♀, CNC), Gr. Sm. Mt. Nat. Park, Tenn., Clingman’s Dome, 2011 m, 22.viii.1957, J.G. Chillcott, CNC480085 (1♂, CNC), 2026 m, 21.v.1965, CNC480089 (1♀, CNC), Gr. Smoky Mt. Nat. Park, Clingman’s Dome, 1920–2024 m, 28.v.1957, J.R. Vockeroth, CNC480092 (1♀, CNC), 20.v.1957, CNC480091 (1♀, CNC), 28.v.1957, CNC480084 (1♂, CNC), Mt. Mitchell, 2072 m, 12.viii.1957, J.G. Chillcott, CNC480083 (1♂, CNC), Mt. Richland-Balsam, Blue Ridge Pkwy, 1828–1950 m, 30.v.1965, J.G. Chillcott, CNC480087 (1♀, CNC), **TN**: Gr. Smoky Mt. Nat. Pk., Indian Gap, 1584 m, 28.v.1957, J.R. Vockeroth, CNC480090 (1♀, CNC), **VA**: Hawksbill, Shenandoah N.P., 1097–1234 m, 7.vi.1962, J.R. Vockeroth, CNC480080–480082 (2♂1♀, CNC), Smyth Co., Mt. Rogers, 1615–1737 m, 1.vi.1962, J.R. Vockeroth, CNC480086 (1♂, CNC).

###### Additional material examined.

**Canada. ON**: Thornhill, 30.v.1964, J.R. Vockeroth, CNC480079 (1♀, CNC). **USA. ME**: Belfast, 16–20.ix.1946, C.W. Sabrosky (1♀, USNM), **NY**: Slide Mt., 25.viii.1935, 4200', H.K. Townes (1♂ 4♀, USNM), **WA**: Mt. Rainier, VanTrump Crk., 2.ix.1917, A.L. Melander (1♀, USNM).

###### Comments.

*Phytomyzacompta* is a large species with a characteristically pale body (also see comments for *P.clematiphaga*). The first flagellomere is also fringed with relatively long hairs and the phallus is unusual: the paraphalli form a single rounded bar, and the two dark arms of the distiphallus are separate from each other and bent posterodorsally. The latter character likely caused this species to be initially classified in the genus *Chromatomyia*, but it does not appear to be related to any of the other lineages of this synonymized genus.

##### Phytomyza crassiseta

Taxon classificationAnimaliaDipteraPhytomyzinae

Zetterstedt

Figs 134 , 766–770

Phytomyza crassiseta Zetterstedt, 1860: 6469. Melander 1913: 271; Hendel 1935: 387; Frick 1959: 427; Block 1969: 357; Spencer 1976: 408, 1990: 224; Spencer and Steyskal 1986b: 185; Glesener and Tilman 1978: 662; Černý 2018: 132; Scheffer and Lonsdale 2018: 88; Eiseman and Lonsdale 2018: 71; Papp and Černý 2020: 335; Černý et al. 2020: 216.
Phytomyza veronicae Brischke, 1881: 271 [preoccupied by Kaltenbach]. Hendel 1935 [synonymy].

###### Description

**(Fig. 134).** Wing length 2.1 mm (European ♂), 2.3 mm (WA ♂), 1.6–2.3 mm (♀). Vein dm-m absent. Eye height divided by gena height: 1.9–4.0. Arista laterally flattened, tapered on apical 1/2. First flagellomere slightly longer than high, broadly rounded apically, profile sometimes slightly subrectangular; hairs slightly longer than average, relatively dense. Fronto-orbital plate projecting anteriorly, and parafacial narrow but sometimes also prominent when viewed laterally. Ocellar triangle slightly larger than tubercle, sometimes subcircular. Cheek pronounced.

***Chaetotaxy***: One ori in female, two in male; two ors. Ocellar and postocellar setae fine and sometimes longer than fronto-orbitals. Four dorsocentrals, decreasing in length anteriorly. Two sparse rows of acrostichal setulae anteriorly. Intra-alar setulae strongly reduced to one or a few setulae anteriorly.

***Colouration***: Body with faint greyish pruinosity evident on pigmented regions, which is denser on thorax, especially on dorsum. Head light yellow with frons sometimes slightly darker; clypeus, palpus and first flagellomere dark brown; scape, pedicel, ocellar triangle, back of head, posteroventral margin of gena and posterolateral corner of frons lateral to base of inner vertical seta brown; fronto-orbital plate with faint, fine whitish pruinosity that appears grey on an angle (as in some *Phytoliriomyza*); fronto-orbital plate with minute to large spots around bases of fronto-orbitals that are sometimes connected to faint brownish grey line along eye margin that uncommonly extends from dark posterolateral corner of frons. Thorax dark brown with grey pruinosity. Halter white. Calypter entirely yellowish white. Legs mostly dark brown, with apex of femora yellow for length greater than width of femur apex, and fore coxa variably yellow, but always with at least apex yellow and base dark. Abdomen brown; side of tergites yellow in female.

***Genitalia***: (Figs 766–770) Epandrium rounded, fused to small, setose surstylus. Cercus small, outer-dorsal margin ill-defined. Hypandrium broadly rounded with wide apical apodeme; inner lobe discrete, with two setae, connected via weak lateral sclerotisation and oblique distal band. Postgonite well-developed with strong apical arch. Basiphallus with two ill-defined, dextrally twisted bands that are each weakly defined and dorsally fused, forming a broad transverse apical plate. Hypophallus membranous, small, concave. Paraphalli lateral, asymmetrical, right sclerite largely desclerotised except at base, and left sclerite large, weakly sclerotised and clavate with basal stem abruptly narrowed; with one pair of darker inner accessory sclerites that flank mesophallus and are connected to distiphallus ventrally by membrane. Mesophallus (interpreted as thicker basal section of distiphallus connected to narrower ejaculatory duct) not readily differentiated from distiphallus, tubular, clear. Distiphallus tubular, mostly clear, slightly flared at opening, with band-like medial sclerotisation with ventral suture and narrow basomedial stem.

###### Hosts.

Plantaginaceae – *Hebe* (Spencer and Steyskal 1986b), *Veronica* (Benavent-Corai et al. 2005).

###### Distribution.

**Canada.** ON*, QC (leaf mine). **USA**: CA, ID, IN*, MA, MD, ME (leaf mines), NC, NJ*, NY, PA, VA, WA, WV. Argentina, Chile, Europe, Japan, Turkey, Russia (Papp and Černý 2020).

###### Type material.

***Holotype* [*crassiseta*]: Sweden.** Skane: Kingsmarken, Lake Ringsjon (HT ♀, ZIL). [Not examined]

***Holotype* [*veronicae*]: Poland.** Gdansk [as “Danzig”] (type data unknown). [Not examined]

###### Material examined.

**Canada. ON**: Wellington Co., Smith Property Trail nr. Arkell, 43°33'N, 80°11'W, 23.vi.2015, O. Lonsdale, CNC441143 (1♀, CNC), Halton, Norval, 5.vi.2008, on *Veronicaspicata*, D. Cheung (1♀, photo voucher – Fig. 134). **Chile.** Estero la Jaula Curico, 1600 m, Nothofagus, i.1964, L. Pena, CNC480097 (1♀, CNC), Piscicultura Aconcagua, 1600 m, 11.xi.1963, L. Pena, CNC480096 (1♀, CNC). **England.** Chippenham Fen, Cambs., 20.ix.1958, [K.A. Spencer], CNC480093 (1♂, CNC), [illegible] Head, S Devon, 7.ix.1954, [K.A. Spencer], Veronica em. 29.ix.1954, CNC480094 (1♀, CNC). **Germany.** Saxony-Anhalt: Tilleda, Sud Kyffhausse, x.1962, I. Michel, mine an Veronica ”Z No. 1962 Hering: Z, CNC480095 (2♀, CNC). **USA. ID**: Collins, A.L. Melander (1♀, USNM), **IN**: Lafayette, J.M. Aldrich, 10.vi.1915 (1♀, USNM), 6.vii.1915 (1♀, USNM), Michigan City, 29.vi.1915, J.M. Aldrich (1♀, USNM), **MA**: Franklin Co., Sunderland, Falls Rd., 13.vii.2012, em. 23.vii.2012, C.S. Eiseman, ex *Veronicaofficinalis* (1♀[with puparium], CNC), Hampshire Co., Pelham, 88 Arnold St., 25.vi.2014, C.S. Eiseman, ex. *Veronicachamaedrys* em. 2–16.vii.2014, #CSE1148, CNC384850–384862 (13♀, CNC), **MD**: Glen Echo, 29.v.1919, J.M. Aldrich (1♀, USNM), Montgomery Co., Colesville, 14.vi.1976, Malaise trap, W.W. Wirth (2♀, USNM), Bethseda, G.C. Steyskal, 3.vi.1972 (1♀, USNM), 16.vii.1967 (1♀, USNM), 4mi SW of Ashton, 25.iv.1987, G.F. and J.F. Hevel (1♀, USNM), Forest Glen, 25.vi.1967, W.W. Wirth (1♀, USNM), P.G. Co., Camp Springs, G.F. Hevel, Malaise trap, 9.vii.1979 (1♀, USNM), 8.vii.1979 (2♀, USNM), 16.vii.1979 (1♀, USNM), Carroll Co., Eldersburg, 2.vi.1985, W.E. Steiner and J.E. Lowry (1♀, USNM), **NC**: Chatham Co., Haywood, 5.vi.1986, G.C. Steyskal (3♀, USNM), Macon Co., Highlands, Lake Ravenel, Malaise trap, W.W. Wirth, 14.vi.1986 (1♀, USNM), 19.vi.1986 (1♀, USNM), Durham Co., Durham, Pelham Rd., 20.iv.2016, T.S. Feldman, *Veronicaperegrine*, em. 14–16.v.2016, #CSE2461, CNC634800–634803 (4♀, CNC), Scotland Co., Laurinburg, St. Andrews University, 2.v.2016, T.S. Feldman, *Veronicaarvensis*, em. 17.v.2016, #CSE2468, CNC634782 (1♀, CNC), Durham Co., Durham, Duke University, 23.v.2016, T.S. Feldman, *Veronicapersica*, em. 29.v-3.vi.2016, #CSE2517, CNC654295–654302 (8♀, CNC), **NJ**: Morristown, 9.iv.1922, A.H. Sturtevant (1♀, USNM), **NY**: Bear Mt., 8.vi.1918, A.H. Sturtevant (1♀, USNM), **PA**: Dubois, 3.ix.1927, A.L. Melander (1♀, USNM), **VA**: Glencarlyn, 30.v.1925, J.R. Malloch (1♀, USNM), Arlington, 18.v.1982, F.C. and B.J. Thompson (1♀, USNM), Alexandria, 11.vi.1952, W.W. Wirth (1♀, USNM), Pulaski, 7.v.1979, G. Steyskal (2♀, USNM), Fairfax Co., Great Falls Park, quarry, 38°59.1'N, 77°14.8'W, Malaise trap, 10–17.v.2007, D.R. Smith (1♀, USNM), **WA**: Mt. Constitution, 22.vii.1909 (1♀, USNM), Chehalis, 25.viii.1911, A.L. Melander (1♀, USNM).

###### Comments.

The laterally flattened arista and unusual genitalia of *Phytomyzacrassiseta* readily differentiate it from other Delmarva species, but diagnosis elsewhere is more difficult where similar characters occur in a number of related species that require further study to refine diagnostic characters. These include the Quebec species *P.pedicularicaulis* Spencer (on *Pedicularis*, Orobanchaceae) (Spencer 1969: figs 477, 478) and the British Columbian species *P.superba* Spencer (host unknown) (Spencer 1969: figs 515, 516). Other related species, including the Palearctic *Phytomyzaaffinis* Fallén, the Albertan *P.banffensis* Spencer, and a number of more southern Nearctic species (Spencer and Steyskal 1986), are superficially quite similar but have a filamentous arista.

Collection records show males of *Phytomyzacrassiseta* to be very rarely encountered in the New World (an English male is illustrated here), where the species has likely been introduced, with females suspected to reproduce parthenogenetically (Spencer and Steyskal 1986b). In Europe, males are also uncommon towards the north, but are found in numbers equal to those of females in the Mediterranean region (Glesener and Tilman 1978). The cytology of this species was examined by Block (1969), who noted that the only other known parthenogenetic species in the family was *P.plantaginis*.

##### Phytomyza davisii

Taxon classificationAnimaliaDipteraPhytomyzinae

(Walton)

Figs 771–777

Agromyza davisii Walton, 1912: 463. Melander 1913: 253; Malloch 1913: 284.
Napomyza davisii . Frick, 1952a: 420, 1959: 419.
Phytomyza davisii . Spencer, 1969: 238, 1990: 43; Spencer and Steyskal 1986b: 175; Eiseman and Lonsdale 2019: 18.

###### Description.

Wing length 3.2 mm (♂), 3.4 mm (♀). Length of ultimate section of vein M4 divided by penultimate section 8.5–13.9. Eye height divided by gena height: 3.3–3.7. Cheek narrow but distinct. First flagellomere rounded, slightly longer than high, narrowing apically. Ocellar triangle barely larger than tubercle.

***Chaetotaxy***: Anterior ori thinner, slightly less or more than 1/2 length of posterior ori; posterior ors slightly shorter and thinner than anterior ors. Four to five rows of scattered acrostichal setulae. Four dorsocentrals, barely decreasing in size anteriorly. Presutural supra-alar small.

***Colouration***: Head light yellow with antenna, back of head, ocellar tubercle, posterolateral corner of frons, gena, occiput and palpus dark brown; face faintly to more distinctly brown; clypeus brown; brownish grey stripe extending along fronto-orbital plate from posterior margin of frons to level of posterior ors; fronto-orbital plate with faint, fine greyish white pruinosity. Thorax dark brown with thick grey pruinosity that is lighter on pleuron and slightly evident on remainder of body. Halter white. Calypter entirely yellowish white. Legs dark brown with apices of femora narrowly yellow. Abdomen dark brown with lateral margin of tergites fading to yellow.

***Genitalia***: (Figs 771–777) Apex of surstylus, which is entirely fused to epandrium, well-defined, but posteriorly with thin, ill-defined carina extending along margin of epandrium to pointed, inwardly directed process ventral to apex of cercus; setae apically on surstylus and along ridge following thin process. Cercus narrow, curved, slightly compressed laterally. Hypandrium large with stout basal arms and two setae on strong, transverse inner lobe; with rounded apical apodeme. Postgonite large and well-sclerotised with three sockets on upcurved apex; one seta basal to shallow, dark inner lobe; basal section dark and divided longitudinally into two bands. Phallophorus large and well-developed with dorsoapical plate partially interlocking and fused with base of right sclerite of basiphallus; flanked by one pair of long, narrow, apically pointed rods. Halves of basiphallus separate, slightly wider on distal 2/5 with dark, shallow ventrobasal process; left sclerite shorter with ventrobasal process wrapped along side of shaft. Hypophallus U-shaped, strongly curved in lateral view; basal margin thick, straight, and with dorsal sub-basal fossa; subapically with short, clear tubule. Paraphallus dark, rod-like, with slight medial constriction; apex weakly fused to mesophallus. Mesophallus with subspherical, dark apical chamber on distal 2/5 that is ventrally composed of two rounded plates; basal section of mesophallus (not interpreted as ejaculatory duct due to positioning past basiphallus) narrower, subcylindrical, with base slightly narrower and only sclerotised ventrally. Distiphallus divided into one pair of long sinuate arms that are strongly arched medially and dorsally angled apically. Ejaculatory apodeme large with short, narrow stem and base, and with large, broad blade; sperm pump with transverse sclerite that produces dark lateral processes and is fused to base of ejaculatory apodeme; base of duct sclerotised, continuous with sclerite on sperm pump.

###### Hosts.

Ranunculaceae – *Ranunculus*.

###### Distribution.

**Canada**: ON, QC. **USA**: IN, MA, MI, MN, MO, NY, TX*, WI.

###### Type material.

***Holotype*: USA. IN**: Lafayette, bred from leaf miners on *Ranunculus*, 9.vi.1912, J.J. Davis (1♀, USNM; type No. 15563).

###### Additional material examined.

**Canada. ON**: Fenelon Falls, 27.v.1928, F.P. Ide, CNC480098 (1♂, CNC), Go Home Bay, 8mi W of Bala, 28.v.1959, J.G. Chillcott, CNC480099 (1♂, CNC). **USA. IN**: Lafayette, bred from leaf miners on *Ranunculusabortivus*, 9.vi.1912 (1? [body mostly destroyed], USNM), **MO**: reared from leaf-miner on *Ranunculusabortivus*, 14.vi.1913 (1♂, USNM), Kirkwood (1♀ [with puparium], USNM), **TX**: Bexar Co., Ebony Hill, Res. Stn., ex. larva, R.O. and C.A. Kendall, 27.iv.1977 (1♂ 1♀ [with puparia], USNM), 28.iv.1977 (1♀ [with puparium], USNM).

###### Comments.

*Phytomyzadavisii* is the only known Delmarva *Phytomyza* with vein dm-m present. The distiphallus, mesophallus and hypophallus are also distinct.

##### Phytomyza ditmani

Taxon classificationAnimaliaDipteraPhytomyzinae

Kulp

Figs 778–780

Phytomyza ditmani Kulp, 1968: 11. Spencer and Steyskal 1986b: 215; Griffiths and Piercey-Normore 1995: 23; Scheffer and Wiegmann 2000: 249; Lonsdale and Scheffer 2011: 1187; Scheffer et al. 2021: 62.

###### Description.

As described for *P.ilicicola* except as follows:

Wing length 1.7–2.0 mm (♂), 1.9–2.0 mm (♀).Wing sometimes with additional transverse veins in first and second radial cells apically. Eye height divided by gena height: 3.6–5.0.

***Chaetotaxy***: Posterior ori shorter than ors, anterior ori minute. Four to six rows of scattered acrostichal setulae.

***Colouration***: Lunule sometimes paler than frons. Face yellowish medially, brown to dark grey laterally. Gena sometimes distinctly whiter than frons. Posterolateral margin of frons dark, with spot encompassing base of vertical setae, sometimes with dark stripe extending along fronto-orbital plate to level of anterior ors. Gena and occiput sometimes dark along posterior and posteroventral margins of eye. Legs evenly brown.

***Genitalia***: (Figs 778–780) Surstylus relatively short, straight and broadly rounded. Epandrial process (bump above anus) short and rounded. Sclerite of hypophallus 1/2-length of mesophallus, apex broadly rounded, base narrow and upcurved (i.e., comma-shaped); broader apical section sometimes subquadrate. Length of mesophallus 4 × width at midpoint. Arms of distiphallus separate and approximately as long as mesophallus.

###### Hosts.

Aquifoliaceae – *Ilexdecidua*, *I.montana*, *I.verticillata* (Scheffer et al. 2021).

###### Distribution.

**USA**: DC, MD, ME, MI, NC, PA.

###### Type material.

***Holotype*: USA. DC**: Washington, 30.x.1964, L.A. Kulp, Type No. 67775 (1♂, USNM).

###### Additional material examined.

See Lonsdale and Scheffer (2011).

##### Phytomyza glabricola

Taxon classificationAnimaliaDipteraPhytomyzinae

Kulp

Figs 781–783

Phytomyza weidhausii . Nomen nudum. Hamilton 1957: 94 [attributed to E. C. Crafts].
Phytomyza ditmani Kulp, 1968: 14. Spencer and Steyskal 1986b: 214; Griffiths and Piercey-Normore 1995: 23; Scheffer and Wiegmann 2000: 249; Scheffer and Hawthorne 2007: 2627; Lonsdale and Scheffer 2011: 1188; Scheffer and Lonsdale 2018: 88; Scheffer et al. 2021: 62.

###### Description.

As described for *P.ilicicola* except as follows:

Wing length 2.2–2.4 mm (♂), 2.4–2.6 mm (♀). Eye height divided by gena height: 3.4–4.4.

***Chaetotaxy***: Sometimes very small additional ori present in front of anterior ori, which is 1/2 length of posterior ori. Five scattered rows of acrostichal setulae.

***Colouration***: Body darker brown; gena greyish to dirty white; dark posterolateral spot on frons surrounding vertical setae sometimes with extension along margin of eye to surround base of ors and (less frequently) base of posterior ori; eye with dark margin along gena and postgena. Legs dark with base of tibiae and apices of femora narrowly to indistinctly yellow; tarsi dirty white, becoming browner apically.

***Genitalia***: (Figs 781–783) Surstylus short and rounded. Epandrial process short and broad. Sclerite of hypophallus slightly longer than mesophallus, narrow and strongly arched; base fused to apex of basiphallus. Length of mesophallus ~ 2.5 × width, shorter than distiphallus and slightly constricted at midpoint. Arms of distiphallus connected at base.

###### Hosts.

Aquifoliaceae – *Ilexglabra*, *I.coriacea*; possibly *I.cassine* (Scheffer et al. 2021).

###### Distribution.

**USA**: AL, CT, DC, FL, GE, MA, MD, MS, NC, NJ, NY, OH, SC.

###### Type material.

***Holotype*: USA. DC**: Washington, 19.viii.1964, L.A. Kulp, Type No. 67427 (1♂, USNM).

###### Additional material examined.

**USA. FL**: Highlands Co., Venus-Archibold Biological Station, 30.iii.2013, C.S. Eiseman, ex *Ilexglabra*, em. 8–28.iv.2013, #CSE255, CNC384726, CNC384727 (1♂,1♀, CNC). Also see Lonsdale and Scheffer (2011).

##### Phytomyza ilicicola

Taxon classificationAnimaliaDipteraPhytomyzinae

Loew

Figs 784–786

Phytomyza Ilicis Loew, 1863: 54 [preoccupied by Curtis 1846].
Phytomyza ilicicola Loew, 1872: 291 [new name for ilicis Loew]. Hendel 1920: 168 [as possible synonym of ilicis Curtis], Frick 1952a: 426, 1957: 205 [lectotype designation], 1959: 429; Kulp 1968: 16; Spencer 1969: 246; Spencer and Steyskal 1986: 213; Scheffer and Wiegmann 2000: 249; Lonsdale and Scheffer 2011: 1189; Scheffer and Lonsdale 2018: 88; Scheffer et al. 2021: 62.
Phytomyza obscurella var. ilicicola . Melander, 1913: 270.
Phytomyza ilicis . Misidentification. Frost, 1924: 76.

###### Description.

Wing length: 1.7–2.1 mm (♂), 1.9–2.1 mm (♀). Vein dm-m absent. Eye height divided by gena height: 3.5–6.5. Vein dm-m absent; veins R_2+3_ and R_4+5_ crowded relatively close to anterior margin of wing. Ocellar tubercle wider than long (often subrectangular), with space between posterior ocelli wider than 3 × width of ocellus; ocellar triangle indistinct, barely larger than tubercle. Fronto-orbital plate narrow with inner margin smooth and slightly convex. Cheek narrow, but distinct.

***Chaetotaxy***: Two ori, anterior 1/2 length of posterior; two ors. Ocellar and postocellar setae at least as long as fronto-orbitals, but thinner. Four to six scattered rows of acrostichal setulae. Four dorsocentral setae, one presutural, decreasing in length anteriorly.

***Colouration***: Body covered with greyish pruinosity that is denser on thorax, especially dorsally; pruinosity coppery postsuturally, bluish presuturally and on anterodorsal corner of anepisternum (colour differences often difficult to see in poorly preserved material). Head mostly grey with brown to yellowish tint; back of head, clypeus, palpus, ocellar triangle and scape dark brown; pedicel and first flagellomere dark brown to black; lunule sometimes paler than frons; frons with thin reflective pruinosity appearing white to dark grey depending on individual and angle of view; posterolateral corner of frons dark to base of inner vertical seta, often with narrow band extending to base of posterior ors; fronto-orbital plate sometimes with spot posterior to base of anterior ors and posterior ori, or with most of plate dark grey excluding anterior and anterolateral margins; face yellowish medially, whitish to dark grey or brownish laterally and on parafacial; gena usually very pale, strongly contrasting remainder of head, sometimes with yellowish tint, less commonly greyish. Thorax dark brown with pruinosity as mentioned above. Halter white. Calypter white with margin and hairs grey to brownish. Legs brown with yellowish tint, with femora darker and fore and mid tibiae and tarsi paler, at least medially; if predominantly pale, then apex of fore femur and faint supra-alar spot on scutum also pale. Abdomen dark brown.

***Genitalia***: (Figs 784–786) Surstylus short, rounded and relatively narrow; fused to epandrium but with suture evident along anterior margin. Epandrium broadly rounded and setose and with small dorsomedial protuberance (“epandrial process”) above anus. Cercus small, setose, and narrow. Subepandrial sclerite divided medially, each side with dark dorsal arm that is contiguous ventrally with flatter, pointed ventral process. Hypophallus membranous and sac-like with one pair of floating lateral sclerites that are subrectangular, curved and ~ 4 × longer than wide with margin irregular. Mesophallus nearly 6 × longer than wide at midpoint and slightly longer than distiphallus. Paraphallus with dark basal section and very flat, pale, nearly indistinct distal plate approaching mediobasal surface of mesophallus. Arms of distiphallus separate, not strongly diverging at base and curved dorsally. Ejaculatory apodeme with base bulging, stem very short, and blade well-developed with marked gradation in pigment distally; sperm pump with transverse sclerite with ends thick, dark, and produced.

###### Hosts.

Aquifoliaceae – *Ilexaquifolium*, *I.opaca*; possibly *I.vomitoria* (Scheffer et al. 2021).

###### Distribution.

**Canada**: ON. **USA**: DC, DE, FL, GA, KY, MA, MD, NC, NY, OH, PA, SC; leaf mines only: CT, TN, VA.

###### Type material.

***Lectotype*: USA. DC**: “Loew Coll.”, [Osten-Sacken], Type No. 13431 (1♀, MCZ).

###### Additional material examined.

**USA. USA. NC**: Scotland Co., Laurinburg, St. Andrews University, 25.ii-4.iii.2016, T.S. Feldman, *Ilexopaca*, em. 18–25.iii.2016, #CSE2387, CNC653938–653941 (1♂ 3♀, CNC), **VA**: Falls Church, 5.v.1969, [illegible], mines *Ilexopaca* em. 5–11.v.1969, CNC480100 (1♂ 1♀, CNC). Also see Lonsdale and Scheffer (2011).

###### Comments.

*Phytomyzailicicola* is collected with relative frequency compared to other holly-mining agromyzids in the Delmarva states, all of which belong to the *P.ilicis* species group. This group was recently revised in North America by Lonsdale and Scheffer (2011), who included discussions on life history and host use. This followed molecular treatments of the group by Scheffer and Wiegmann (2000) and Scheffer and Hawthorne (2007), and the description of a related species feeding on *Gelsemium* (Gelsemiaceae) in North Carolina, *P.omlandi* Scheffer and Lonsdale (Scheffer and Lonsdale 2011). Scheffer et al. (2021) provided a thorough analysis of the host plant use and diversification in the group. Of the 12 Nearctic species in this group, *P.ilicis* was introduced into the western Nearctic from Europe and eleven native species occur in the eastern United States. An additional undescribed species from Florida and North Carolina mentioned in Scheffer et al. (2021) on *Ilexamelanchier* is not considered here. Nine of the eleven described native species occur in the Delmarva states: *P.ditmani*, *P.glabricola*, *P.ilicicola*, *P.leslieae*, *P.lineata*, *P.nemopanthi*, *P.opacae*, *P.verticillatae*, and *P.wiggii*.

The species group is tentatively treated as monophyletic on the basis of *Ilex*-feeding, widely separated posterior ocelli, and overall genitalic morphology. The eleven native Nearctic species are further allied on the basis of a subrectangular ocellar tubercle and a bump above the anus on the epandrium (Lonsdale and Scheffer 2011).

##### Phytomyza lactuca

Taxon classificationAnimaliaDipteraPhytomyzinae

Frost

Figs 802–804

Phytomyza lactuca Frost, 1924: 85. Frost 1928: 77; Spencer 1969: 249; Winkler et al. 2009: 284; Scheffer and Lonsdale 2018: 88.
Chromatomyia lactuca . Griffiths, 1974: 37, 1977: 336; Spencer 1981: 442; Spencer and Steyskal 1986b: 191; Scheffer et al. 2007: 772.

###### Description.

Wing length 2.2–2.6 mm (♂), 2.1–2.6 mm (♀). Vein dm-m absent. Eye height divided by gena height: 2.3–2.9. First flagellomere large with distal margin rounded; shape variable; larger in female, sometimes circular but usually slightly clavate with anterodorsal region swollen; smaller in male, sometimes with dorsal and ventral margins nearly parallel; hairs very conspicuously elongate, length at least 3 × width of base of arista, becoming longer anterodorsally. Fronto-orbital plate shallowly produced dorsally, smaller than narrow “cheek”.

***Chaetotaxy***: Two ori with anterior seta either absent or very thin and not more than 1/2 length posterior ori; two ors. Single row of orbital setulae straight to irregular. Ocellar and postocellar setae at least as long as fronto-orbitals. Eye with very sparse short hairs. Two strong rows of acrostichal setulae to midpoint of scutum. Intra-alar setulae relatively few, at least with one well-developed setula in front and behind suture. Four dorsocentrals, decreasing in length anteriorly. Small additional medial presutural supra-alar present.

***Colouration***: Head light yellow with ocellar tubercle and posterolateral margin of frons dark brown to region between vertical setae or to base or inner vertical; pedicel, first flagellomere, palpus and back of head dark brown; clypeus brown. Thorax dark brown with dense greyish pruinosity that is thicker dorsally and also faintly seen elsewhere on body. Halter white. Calypter margin and hairs brown. Legs dark brown with base of tibiae narrowly yellow and apex of femora yellow for length equal to width of femur at spot. Abdomen brown with posterior margin of tergites 2–6 sometimes yellow.

***Genitalia***: (Figs 802–804) Hypandrium very short and stout; inner lobe with several anterior setae and broad membranous extension that is broadly fused to postgonite. Phallophorus small, shallow; lateral paired sclerites short, dark, slightly pointed apically. Postgonite broad, flat and with several outer setae; apex rounded with dorsal subapical point. Halves of basiphallus crossing at base; left sclerite with ventrobasal process wrapping around shaft, right sclerite with broad laterobasal lobe-like extension. Paraphallus absent. Hypophallus narrow and U-shaped with arms darker and parallel, and distal arch broadly and more weakly sclerotised. Mesophallus not evident. Distiphallus dark, tubular, narrow, and angled ventrally but slightly upcurved, nearly as long as hypophallus. Ejaculatory apodeme small, finger-like; sperm pump small.

###### Host.

Asteraceae – *Crepis*, *Lactuca*, *Sonchus*, *Taraxacum* (Spencer and Steyskal 1986b).

###### Distribution.

**Canada**: AB, NL*, ON, PE*, SK*. **USA**: CA, MD, MI, NC, NY, PA, WV*.

###### Type material.

***Holotype*: USA. PA**: Arendtville, reared ex. Lactucascarolavar.integrata (1♂, USNM). [Lost]

###### Material examined.

**Canada. NL**: Pistolet Bay, 10.viii.1982, G. Cuff, Lot 83–69, 64, CNC480102 (1♂, CNC), **ON**: Ottawa, 695 Malibu Tr., 45°22'N, 75°43'W, leaf mine *Sonchusoleraceus*, 3.vii.2015, O. Lonsdale, em. 7.vii.2015, CNC454633–454635 (1♂ 2♀, CNC), Ottawa, 2.vii.2015, O. Lonsdale, leaf mine unknown host, em. 7.vii.2015, CNC454639 (1♂, CNC), Ottawa, 4.ix.1956, G.G. Lewis, serpentine leaf mine in *Lactucacanadensis*, CNC480104–480106 (1♀, 1♂1♀[same pin], 2 empty puparia [gel capsule], CNC), **PE**: O’Leary, v.1975, L.S. Thompson, 75–332, CNC480101 (1♀, CNC), **SK**: Regina, 4.x.1973, M.G. Maw, hand collected, ex *Sonchusoleraceus* 73–216-Sask, So-R-10, CNC480103 (1♂, CNC). **USA. MD**: Bethseda, 28.vi.1980, G.C. Steyskal, ex. *Lactuca* sp. (3♂ 2♀ [with puparia], USNM), **MI**: Battle Creek, 25.iv.1936, C.W. Sabrosky (1♂, USNM), **WV**: Parkersburg, 21.vi.1970, G. Steyskal (1♂, USNM).

###### Comments.

The enlarged first flagellomere and very elongate hairs on the first flagellomere are highly diagnostic of this species. As noted in Griffiths (1977), the male genitalia are similar to those in the *Phytomyzasyngenesiae* group, including the pestiferous *P.horticola*, but the distiphallus of this species is proclinate, and the hypophallus and distiphallus are darker and stouter.

##### Phytomyza leslieae

Taxon classificationAnimaliaDipteraPhytomyzinae

Lonsdale & Scheffer

Figs 787–789

Phytomyza leslieae Lonsdale & Scheffer, 2011: 1191. Scheffer et al. 2021: 62.

###### Description.

As described for *P.ilicicola* except as follows:

Wing length 1.4–2.1 mm (♂), 1.4–1.8 mm (♀). Eye height divided by gena height: 3.5–5.2.

***Chaetotaxy***: Anterior ori 1/2 length of posterior ori.

***Colouration***: Gena usually very pale, strongly contrasting remainder of head, sometimes with yellowish tint. Pedicel relatively pale, never as dark as first flagellomere. Face yellowish centrally, with side and parafacial whitish, never dark grey. Fronto-orbital plate sometimes with dark stripe extending from lateral margin to base of one or both ors. Legs more uniformly brown with tibiae and tarsi variably whitish, at least in part on fore and mid legs; apex of femora sometimes narrowly yellowish.

***Genitalia***: (Figs 787–789) Surstylus short, broader apically. Epandrial process rounded with slight ventral curve. Sclerite of hypophallus directed posteriorly (not ventrally), subrectangular in general outline, with length 2 × width, and with irregular margins; posterior margin sometimes strongly convex. Mesophallus bulging on distal 1/2 and with length ~ 3 × width at midpoint. Paraphallus broad. Arms of distiphallus separate and bases not strongly diverging.

###### Hosts.

Aquifoliaceae – *Ilexcassine*, *I.myrtifolia*; possibly also *I.opaca* (Scheffer et al. 2021).

###### Distribution.

**USA**: AL, FL, MD, NC, SC.

###### Type material.

***Holotype*: USA. AL**: Covington Co., Conecuh Natl. For., “Salt Pond” area, 10.i.1998, coll. S.J. Scheffer, 98-13, ex *Ilexmyrtifolia* (1♂, USNM).

###### Additional material examined.

See Lonsdale and Scheffer (2011).

##### Phytomyza lineata

Taxon classificationAnimaliaDipteraPhytomyzinae

Lonsdale & Scheffer

Figs 790–792

Phytomyza lineata Lonsdale & Scheffer, 2011: 1194. Scheffer et al. 2021.

###### Description.

As described for *P.ilicicola* except as follows:

Wing length 1.8 mm (♂). Female unknown. Eye height divided by gena height: 4.0–4.8.

***Chaetotaxy***: Anterior ori very small; posterior ors duplicated on left side in one male. Six to seven scattered rows of acrostichal setulae.

***Colouration***: Scutum sometimes with postpronotum slightly paler/reddish and with posterior margin of notopleuron yellowish. Face brown with lateral margin and parafacial dark grey. Posterolateral corner of frons dark with narrow yellowish line between base of vertical setae. Posterolateral margin of frons with narrow dark line. Legs brown.

***Genitalia***: (Figs 790–792) Surstylus short and tapering. Epandrial process narrow with distinct ventral curve. Sclerite of hypophallus narrow, length 5 × width. Length of mesophallus slightly > 5 × width at midpoint. Paraphallus relatively narrow and weakly defined. Arms of distiphallus separate, slightly longer than mesophallus and subparallel on basal 1/2.

###### Host.

Aquifoliaceae – *Ilexverticillata*.

###### Distribution.

**USA**: MD, MI, PA, VA.

###### Type material.

***Holotype*: USA. VA**: Nelson Co. not far from Crabtree Falls, 12.ix.1999, coll. S.J. Scheffer, ex *Ilexverticillata* (1♂, USNM).

###### Additional material examined.

See Lonsdale and Scheffer (2011).

##### Phytomyza loewii

Taxon classificationAnimaliaDipteraPhytomyzinae

Hendel

Figs 805–807

Phytomyza Clematidis Loew, 1863: 55 [preoccupied by Kaltenbach (1859)].
Phytomyza nitida . Misidentification, in part. Melander 1913: 271.
Phytomyza loewii Hendel, 1923: 1923 [replacement name for Clematidis]. Frick 1957: 205 [type data, lectotype designation]; Spencer 1969: 251; Spencer and Steyskal 1986b: 217; Eiseman and Lonsdale 2018: 77.
Phytomyza ranunculoides Spencer in Spencer and Steyskal 1986b: 320. Syn. nov.

###### Description.

Wing length 1.6–1.9 mm (♂), 2.0–2.1 mm (♀). Vein dm-m absent. Eye height divided by gena height: 2.7–5.0. Fronto-orbital plate and scutum very shiny, strongly contrasting remainder of frons; fronto-orbital plate relatively broad medially, 1/4 to ~ 1/3 width of frons. Ocellar triangle rounded, slightly larger than tubercle.

***Chaetotaxy***: One ori (sometimes with additional minute ori anteriorly); one ors (sometimes two on one side). Ocellar and postocellar setae subequal to fronto-orbitals, ocellar slightly thinner and shorter. Four dorsocentrals, one presutural, decreasing in size anteriorly. Four to six irregular rows of acrostichal setulae.

***Colouration***: Body dark brown with halter and labellum yellow; usually with tarsi (distal two tarsomeres becoming darker apically), narrow apices of femora, and base and apex of fore tibia variably light brown to yellowish; sometimes mid and hind tarsi similarly yellowish, and/or most of fore tibia, distal 1/3 of mid tibia and apex of hind tibia also paler. Calypter margin and hairs brown.

***Genitalia***: (Figs 805–807) Hypandrium very short and broad with haired membrane arising from inner lobe. Postgonite broadened medially and distally with apical margin scalloped; with one seta. Halves of basiphallus relatively long, flat and parallel with bases overlapping. Hypophallus with one pair of lateral rod-like sclerites sometimes partially connected apically by marginal sclerotisations along membrane. Paraphalli elongate, fused and dark with apex long, curved, pointed and often curved to right. Distiphallus short, Y-shaped, directed basally and with large membranous fringe.

###### Hosts.

Ranunculaceae – *Clematis*, *Ranunculushispidus* (corrected identity of MI host listed below, C.S. Eiseman, pers. comm.).

###### Distribution.

**Canada**: ON, QC. **USA**: CA, DC, GA, ID, IN, LA, MA, MD*, MI, VA*, WA.

###### Type material.

***Lectotype* [*loewii*]: USA. DC**: “D.C.”, “85”, “Loew coll.”, Clematidis [illegible], Type No. 13429 (1♀, MCZ).

***Holotype* [*ranunculoides*]: USA. MN**: Minneapolis, Minnehaha Creek, em. 20.viii.1980, ex. leaf mine coll. 29.vi.1980, K.A. Spencer (1♂, USNM).

###### Additional material examined.

**Canada. ON**: Toronto, “2-5-96”, J.M. Aldrich (1♂ 1♀, USNM), Bell’s Cor., wild Clematis, 4.vi.1952, J.F. McAlpine, sweeping, CNC480109–480119 (2♂, 9♀, CNC), Renfrew Co., Jacks Lakes, 45°41'16.14"N, 77°30'4.77"W, 7.vii.2018, C.S. Eiseman, *Clematisvirginiana*, em. 22.vii.2018, #CSE4822, CNC1643631 (1♂, CNC), **QC**: Perkin’s Mills, caught on Clem[atis]. vert[icillaris]., 18.vii.1967, [K.A. Spencer], CNC480107 (1♂, CNC). **USA. DC**: 26.viii.1904 (1♂, USNM), on young terminals of *Clematis* (1♀, USNM), **GA**: Rabun Co., Pine Mountain, 426 m, 4.v.1957, J.R. Vockeroth, CNC480108 (1♂, CNC), **ID**: Moscow, J.M. Aldrich (1♀, USNM), **IN**: Lafayette, 16.v.1917, J.M. Aldrich (1♀, USNM), **MA**: Franklin Co., Northfield, 276 Old Wendell Rd., 29.vi-2.vii.2016, *Clematisvirginiana* em. 18.vii-2.viii.2016, C.S. Eiseman (23♂ 26♀), Franklin Co., Northfield, 42°38'45.47"N, 72°25'39.65"W, adult on *Clematisvirginiana*, 29.vi.2016, C.S. Eiseman, #CSE2646, CNC654108 (1♂, CNC), **MD**: Montgomery Co., Clarksburg, Little Bennett Reg. Park, 21.ix.1990, W.E. Steiner, M.J. and R. Molineaux (5♂, USNM), **MI**: Ingham Co., Meridian, Harris Nature Center, 42°41'50.45"N, 84°22'38.90"W, 28.vi.2018, C.S. Eiseman, *Anemonevirgiana*, em. 12.vii.2018, #CSE4745, CNC1643653 (1♀, CNC), **VA**: Fairfax Co., Dyke Marsh, 18.v.1977, W.N. Mathis (1♀, USNM).

###### Comments.

*Phytomyzaloewii*, as discussed in Spencer and Steyskal (1986b), apparently differs from *P.ranunculoides* in having paler tarsi, a longer costal ratio and a less shiny fronto-orbital plate, but this does not appear to be supported with the material available, including the types of both species. Furthermore, the series of specimens from Bell’s Corners, Ontario, reveal a full range of colour types, suggesting that colour is not useful in differentiating the two species. The glossiness of the fronto-orbital plates is also quite uniform across all specimens, and while there is slight variation is venation, differences appear to be negligible. Regardless, the lengths of costal sectors appear to vary much within agromyzid species and do not appear to be useful is species diagnosis. Genitalic morphology, a much more accurate indicator of specific boundaries in the genus, also appears to be quite uniform for all available males.

The lectotype of *Phytomyzaloewii* is unfortunately a female missing its head, and therefore of little practical use, but its wing length and colour otherwise fit well within the spectrum of variation listed above; the type appears to be slightly paler overall (particularly on the calypter), but this may be an artifact of preservation.

##### Phytomyza nemopanthi

Taxon classificationAnimaliaDipteraPhytomyzinae

Griffths & Piercey-Normore

Phytomyza nemopanthi Griffths & Piercey-Normore, 1995: 24. Scheffer and Wiegmann 2000: 249; Lonsdale and Scheffer 2011: 1196; Scheffer et al. 2021: 62.

###### Description.

As described for *P.ilicicola* except as follows (from Lonsdale and Scheffer 2011):

Wing length 1.9 mm (♂), 2.2 mm (♀). Eye height divided by gena height: 3.1–3.6. Thorax slightly less pruinose and without bluish tint.

***Chaetotaxy***: Anterior ori 1/2 length of posterior ori or absent. Acrostichal setulae in five to six scattered rows.

***Colouration***: Unlike most (but not all) *P.ilicicola*, gena never bright white and face and posterior margin of postgena never white, and legs more uniformly pale. Head with brownish to grey tint, without strong colour contrast on gena; face yellowish centrally, and side of face and parafacial dark grey or brownish; gena and occiput dark along posterior and posterolateral margins of eye. Posterolateral corner of frons dark, with spot encompassing base of vertical bristles and often with thin band extending to base of posterior ors; orbital plate variable in coloration, sometimes with light spot posterior to base of anterior ors and posterior ori, or with most of plate dark grey, excluding anterior and anterolateral margins. Legs brown with tibiae and tarsi paler; fore femur sometimes becoming gradually paler to apex.

***Genitalia***: (Lonsdale and Scheffer 2011: figs 59, 60) Lateral sclerite of hypophallus usually shorter and less strongly arched, mesophallus thinner before midpoint, and arms of distiphallus more strongly diverging.

###### Hosts.

Aquifoliaceae – *Ilexambigua*, *I.collina*, *I.montana*, *I.verticillata* (Scheffer et al. 2021), *I.mucronata*.

###### Distribution.

**Canada**: NL. **USA**: FL, NC, NY, WV, WI.

###### Type material.

***Holotype*: Canada. NL**: Avalon Peninsula, Cochrane Pond Road, 27.ix-10.x.1991, M. D. Piercey-Normore, in leaf of *Nemopanthusmucronata* (L.) (1♂, CNC).

##### Phytomyza nervosa

Taxon classificationAnimaliaDipteraPhytomyzinae

Loew

Figs 133 , 808–810

Phytomyza nervosa Loew, 1869: 52. Frick 1959: 432; Spencer and Steyskal 1986b: 181.

###### Description

**(Fig. 133).** Wing length 2.2–2.5 mm (♂), 2.7–2.8 mm (♀). Vein dm-m absent. Eye height divided by gena height: 2.1–2.2. Buccal cavity relatively broad with epistoma produced. Ring around eye prominent. Palpus short and broadly ovate.

***Chaetotaxy***: Two ori; two ors. Acrostichal setulae absent. Four dorsocentrals, decreasing in length anteriorly.

***Colouration***: Head dark yellow with posterolateral margin of frons lateral to base of inner vertical, large round spot around tubercle and back of head above foramen (excluding margin) brown; palpus, clypeus and first flagellomere black. Notum dark brown with grey pruinosity, and postpronotum, notopleuron and supra-alar spot yellow. Pleuron dark yellow with brown/grey mottling (paler posterodorsally on katepisternum and medially on anepisternum). Halter white. Calypter dusky with hairs brown to golden. Coxae and femora yellow with light dorsal mottling on fore femur (sometimes restricted to base) and with brownish dorsal spot near base of mid femur and near apices of mid and hind femora; tibiae brownish; tarsi brown. Abdomen brown with male sternite 8 and posterior margin of tergites 5 and 6 yellow.

***Genitalia***: (Figs 808–810) Hypandrium subtriangular with apex rounded, and with long, separate basomedial plate; inner lobe transverse, arched and with several anterior setae. Postgonite long and flat with apex broad and emarginate apically. Halves of basiphallus narrow, diverging apically. Hypophallus broad and U-shaped, above one pair of converging narrow bars that nearly meet to form a triangle. Paraphalli small, weakly sclerotised, converging apically, but not meeting. Mesophallus length 2 × width, narrowing apically, rounded basally; duct adjoining mesophallus pigmented. Distiphallus divided into one pair of very narrow, slender, ventrally pointing rods slightly shorter than mesophallus. Ejaculatory apodeme short and finger-like with swollen base.

###### Host.

Unknown.

###### Distribution.

**USA**: DC, IA, IL*, IN, KS, MD*, MI, PA, TN*, VA*.

###### Type material.

***Holotype*: USA.** District of Columbia (1♀, MCZ; type No. 13432). [Not examined]

###### Material examined.

**USA. IL**: Chicago, Thatcher Road, 29.v.1949, A.L. Melander (1♀, USNM), **MD**: Cabin John Bridge, 28.iv.1912, Malloch (1♂, USNM), nr. Plummers Id., 25.iv.1915, R.C. Shannon (2♀, USNM), Montgomery Co., Plummers Island, 38°58'N, 77°10'W, Malaise trap, 24.iv-7.v.2006, D.R. Smith and J.W. Brown (1♀, USNM), 25.iv.1987 (1♂ 1♀, USNM), 4mi SW of Ashton, G.F. and J.F. Hevel, 29.iv.1984 (2♀, USNM), **TN**: Cocke Co., Ramsey’s Cascade, 8.5mi E Gatlinburg, Great Smokey Mountain National Park, 35°42.2'N, 83°21.5'W, 1900', 28.v.1999, S.D. Gaimari (1♀, USNM), **VA**: Chain Bridge, 20.iv.1924, J.R. Malloch (1♂, USNM).

###### Comments.

The broadly ovate palpus is characteristic of this species, as is the bright head, prominent parafacial, lack of acrostichal setulae and large body size.

##### Phytomyza opacae

Taxon classificationAnimaliaDipteraPhytomyzinae

Kulp

Figs 793–795

Phytomyza opacae Kulp, 1968: 21. Spencer and Steyskal 1986b: 215; Lonsdale and Scheffer 2011: 1198; Scheffer and Lonsdale 2018: 88; Scheffer et al. 2021.

###### Description.

As described for *P.ilicicola* except as follows:

Wing length 1.5–1.9 mm (♂), 1.8–2.0 mm (♀). Eye height divided by gena height: 4.2–5.2.

***Colouration***: Fronto-orbital plate pale, usually with narrow dark line extending to base of posterior fronto-orbital, but rarely reaching base of posterior ori.

***Genitalia***: (Figs 793–795) Surstylus broadly rounded. Epandrial process broad with slight ventral curve. Sclerite of hypophallus semi-circular with anterior margin slightly concave. Paraphalli tapered along outer-distal margin and slightly bowed, not parallel. Mesophallus nearly 7 × longer than wide. Arms of distiphallus slightly shorter than mesophallus, separate and not strongly diverging.

###### Hosts.

Aquifoliaceae – *Ilexopaca*, *I.cassine*, *I.aquifolium*, *I.amelenchier* (Scheffer et al. 2021).

###### Distribution.

**USA**: DC, DE, FL, MD, NC, NJ (leaf mines only), NY, SC.

###### Type material.

***Holotype*: USA. MD**: Prince George Co., College Park, 21.v.1964, em. 29.v.1964, C.W. McComb, Type No. 67776 (1♂, USNM).

###### Additional material examined.

See Lonsdale and Scheffer (2011).

##### Phytomyza osmorhizae

Taxon classificationAnimaliaDipteraPhytomyzinae

Spencer

Figs 757–759

Phytomyza osmorhizae Spencer, 1969: 261. Spencer and Steyskal 1986b: 220.

###### Description.

Wing length 2.1–2.4 mm (♂), 2.5–2.6 mm (♀). Vein dm-m absent. Eye height divided by gena height: 2.6–3.3. Relatively stout-bodied. Fronto-orbital plate subshiny. First flagellomere narrow, not much higher than pedicel. Cheek narrow.

***Chaetotaxy***: One or two ori (anterior seta no more than 1/2 length); one or two ors (posterior seta less than 2/3 length). Orbital setulae sparse. Four dorsocentrals, one presutural, decreasing in height anteriorly. Four irregular rows of acrostichal setulae.

***Colouration***: Head light brown with antenna, ocellar tubercle, back of head, posterior margin of frons and posterolateral corner of frons around vertical setae dark brown; fronto-orbital plate with irregular dark brown pigment medially and posteriorly. Remainder of body mostly dark brown with light brownish pruinosity; fore knee yellowish, halter white and notopleuron and postpronotum slightly paler. Calypter margin and hairs brown.

***Genitalia***: (Figs 757–759) Hypandrium short, broadly rounded, basal arm gracile, with thinner narrow apical process, haired inner membrane, and small setose inner lobe. Basiphallus long and narrow with bases overlapping and partially fused; base of left sclerite with ventral arm. Hypophallus membranous with narrow rod-like lateral sclerites that terminate in a small, apically sclerotised and serrated plate that is ill-defined basally. Paraphallus band-like, extending from apex of basiphallus to ventromedial surface of mesophallus; mostly clear with irregular pigmentation. Mesophallus indistinguishable from distiphallus, clear and bulbous with ventromedial sclerotised patch. Distiphallus membranous, split into two relatively thick tubules that widen on distal 1/2; subapically with ill-defined pigmented band. Ejaculatory apodeme small and narrow with blade only slightly expanded; sperm pump with faint sclerotised patch.

###### Distribution.

**Canada**: ON. **USA**: VA, WI.

###### Hosts.

Apiaceae – *Osmorhizaclaytonia*, *O.longistylis* (Spencer and Steyskal 1986b).

###### Type material.

***Holotype*: USA. VA**: Rosslyn, em. ex. *Osmorhizalongistylis* in lab, xii.1922-i.1923 from larva, leg. 17.xi.1922, M.T. VanHorn (1♂, USNM).

###### Additional material examined.

**USA. VA**: Chain Bridge, 23.iv.1922, J.R. Malloch (2♂, USNM), Rosslyn, 17.xi.1923, larvae and pupae, ex. *Osmorhizalongistylis*, iss. in laboratory xii.1922-i.1923 (1♂ 4♀, USNM), M.T. VanHorn (1♀, USNM), Plummers Isl., R.C. Shannon, 23.iv.1914 (1♂, USNM), 8.iv.1914 (1♀, USNM), Fairfax Co., Dead Run, R.C. Shannon, 26.iii.1925 (1♂, USNM), 18.iv.1916 (1♂, USNM), 24.iii.1925 (1♂, USNM).

###### Comments.

*Phytomyzaosmorhizae*, like many other *Phytomyza*, is a small dark species that is unremarkable externally, although it has light brownish (not grey) pruinosity on the notum and a relatively dark frons. The phallus is most diagnostic, however, and should be examined for accurate identification.

##### Phytomyza persicae

Taxon classificationAnimaliaDipteraPhytomyzinae

Frick

Figs 811–813

Phytomyza obscurella var. nigritella Zetterstedt. Misidentification. Frost 1924: 81.
Phytomyza persicae Frick, 1954: 369. Spencer 1969: 266; Spencer and Steyskal 1986b: 211; Scheffer and Lonsdale 2018: 88; Eiseman and Lonsdale 2018: 81.

###### Description.

Wing length 1.7–2.0 mm (♂), 1.8–2.2 (♀). Vein dm-m absent. Eye height divided by gena height: 3.5–5.9. First flagellomere rounded, narrow, slightly shorter than high, not much higher than pedicel. Posterior ocelli separated by at least 3 × their width. Ocellar triangle indistinct. Cheek narrow.

***Chaetotaxy***: One or two ori (anterior seta usually 1/2-length or smaller if present, but uncommonly up to 3/4 length); two ors. Ocellar and postocellar setae at least as long as fronto-orbitals. Four dorsocentrals, one presutural, decreasing in height anteriorly; only posterior seta large, with second from rear not more than 3/5 length. Three or four scattered rows of acrostichal setulae ending at level of posterior dorsocentral.

***Colouration***: Setae brown to dark brown with pale shine (not black). Head light brown to greyish with antenna (sometimes only first flagellomere), fronto-orbital plate, palpus, clypeus and ventral margin of gena dark brown; gena and postgena (at least below cheek) sometimes slightly yellowish; dark brown spot on ocellar tubercle sometimes not much darker than surrounding frons, but sometimes clearly darker and sometimes much enlarged with posterior margin extending to base of vertical setae; posterolateral corner of frons sometimes noticeably darker to base of outer or inner vertical. Thorax with faint greyish pruinosity that is denser dorsally and also less clearly present on remainder of body; pruinosity sometimes appearing coppery postsuturally. Halter white. Calypter margin and hairs brown. Legs mostly dark brown, apices of femora sometimes narrowly to more widely yellowish, tibiae sometimes slightly paler, at least apically on fore leg, and tarsi yellowish with apical segments darker. Abdomen brown.

***Genitalia***: (Figs 811–813) Inner lobe of hypandrium narrow and closely surrounding postgonite. Halves of basiphallus overlapping at base, but otherwise flat, parallel. Sclerites of hypophallus band-like, mostly parallel with apices slightly incurved. Paraphallus small, dark, band-like, directed dorsally to fuse to ventromedial margin of mesophallus. Mesophallus dark, subcylindrical and with slight medial constriction. Distiphallus entirely split, forming one pair of narrow and very elongate, looped arms; very dark, but apex paler with small, clear apical cup. Ejaculatory apodeme with dark, narrow stalk and broad fan-shaped blade.

###### Hosts.

Rosaceae – *Prunus*.

###### Distribution.

**Canada**: ON, NS*. **USA**: CT, MA, NY, OH, VA.

###### Type material.

***Holotype*: USA. OH**: Erie Co., 15.viii.1952, peach leaves, A.C. Dowdy (1♂[only head, one leg and puparium remaining], USNM).

###### Paratypes examined.

**Canada. ON**: Fonthill, 18.viii.1950, W.L. Putnam, peach leaf miner, CNC480131 (1♀, CNC), Vineland Sta., 20.vi.1951, W.L. Putnam, peach, CNC480122 (1♂, CNC), 21.vi.1951, CNC480124, CNC480129 (1♂,1♀, CNC), 23.vi.1951, CNC480125–480128 (4♀, CNC), 25.vi.1951, CNC480130 (1♀, CNC), 26.vi.1951, CNC480120, CNC480121, CNC480123 (3♂, CNC). **USA. OH**: Berlin Heights, v.1952 (10♂ 1♀ 1?, USNM).

###### Additional material examined.

**Canada. NS**: CBHNt. Pk., Mackenzie Mtn., 400 m, birch and fir, 29.v.1984, B.E. Cooper, PG639848, CNC480132 (1♂, CNC), 7.vi.1984, CNC480134 (1♂, CNC), CBHNt. Pk., North Mt., 400 m, 9.vi.1984, B.E. Cooper, PG767865, CNC480133 (1♂, CNC). **USA. CT**: New haven, “1717”, W.E. Britton, larva coll., 6.viii.1917, in peach leaf, emerged 1.vii.1918 (1?, USNM), **MA**: Hampshire Co., Southampton, 37 Middle Rd., 18.x.2013, C.S. Eiseman, ex. *Prunuspersica* em. 21.iii.2014, #CSE1015, CNC384791 (1♂, CNC), **OH**: Erie Co., 15.viii.1952, peach leaves, A.C. Dowdy (1♀, USNM), Wayne Co., 1944, Weaver, peach leaf miner, “6-5”, “6-16” (1♂ 1♀, USNM), **VA**: Winchester, 29.vii.1915, bred from peach foliage, quaintance No. 1481, E.B. Blakeslee (2♂ 1♀, USNM).

###### Comments.

The long, dark, looped arm of the distiphallus is an unmistakable characteristic of *Phytomyzapersicae*, and should be examined to confidently differentiate it from similar species such as the *Ilex* leaf miners, which are nearly identical externally. In Europe, the very similar P. *heringiana* Hendel occurs on apple (*Malusdomestica*) (Papp and Černý 2020: fig. 128).

##### Phytomyza plantaginis

Taxon classificationAnimaliaDipteraPhytomyzinae

Robineau-Desvoidy

Figs 814–818

Phytomyza Robinaldi Goureau, 1851: 142 [“Priority should… be accorded to R.-D. but a submission to the I.C.Z.N. will be required under Article 79(c) (1) of the 1985 code” (Spencer and Martinez 1987)].
Phytomyza plantaginis Robineau-Desvoidy, 1851: 404. Frost 1924: 82; Hendel 1935: 455; Frick 1959: 434; Spencer and Steyskal 1986b: 190; Černý 2018: 132; Scheffer and Lonsdale 2018: 88; Eiseman et al. 2019: 316; Papp and Černý 2020: 515; Černý et al. 2020: 218.
Phytomyza genualis Loew, 1869: 52. Frick 1957 [synonymy].
Phytomyza crassiseta (Zetterstedt). Misidentification, in part. Melander 1913: 271.
Phytomyza nannodes Hendel, 1935: 59. Spencer 1963a [synonymy].
Phytomyza biseriata Hering, 1936: 77. Spencer 1963a [synonymy].
Phytomyza plantaginicaulis Hering, 1944: 118. Spencer 1963a [synonymy].

###### Description.

Wing length 1.6–1.8 mm (♂), 1.9–2.3 mm (♀). Vein dm-m absent. Eye height divided by gena height: 1.5–2.9. Parafacial relatively pronounced when seen laterally; fronto-orbital plate slightly evident laterally, more so anteriorly; cheek evident, nearly 1/2 height of gena, continuing as ring around eye. First flagellomere well-developed, slightly longer than high with dorsal and ventral margins usually subparallel and anterodorsal margin more produced.

***Chaetotaxy***: One ori; two ors. Acrostichal setulae absent or several setulae present in two rows anteriorly. Four dorsocentrals, decreasing in length anteriorly. Posterior notopleural seta and additional medial presutural supra-alar absent.

***Colouration***: Head mostly whitish yellow with frons between fronto-orbital plates yellower; small brown spot surrounding ocellar tubercle; first flagellomere black, pedicel and sometimes scape brownish towards base; posterolateral corner of frons brown to base of inner vertical seta, but not extending far laterally along margin of eye, leaving most of border around eye yellow; back of head dark brown, often with ventral margin yellowish and one pair of yellow spots dorsally lateral to tubercle; clypeus light brown to yellowish or brown; palpus dark yellow to light brown. Thorax dark brown with dense grey pruinosity, pruinosity more faintly present on rest of body, but only evident on pigmented regions. Halter white. Calypter entirely white. Legs brown with fore coxa (excluding base) and apex of femora yellow; sometimes base of tibiae narrowly yellow, usually on fore leg. Abdomen dark brown.

***Genitalia***: (Figs 814–818) Hypandrium short, rounded and stout with shallow apical process and narrow inner lobe with one pair of medial setae. Postgonite broad with curved ventral subapical process and one medial seta. Phallophorus flanked by one pair of narrow membranous plates that are only sclerotised at pointed apex. Basiphallus divided into several sclerites, including one pair of long diverging bars, several smaller, irregular, thinner interlocking dorsomedial plates, and several minute medial spinulae. Paraphallus C-shaped, distal 1/2 parallel to distiphallus. Long apical section of duct pigmented. Mesophallus indistinct. Distiphallus short, tubular, angled ventrally, length 2 × width. Ejaculatory apodeme very short with base produced to one side.

###### Hosts.

Plantaginaceae – *Plantago* spp.

###### Distribution.

**Canada**: BC*, ON, QC. **USA**: Widespread, including HI (Sasakawa 1964). Algeria (Černý 2018). Bermuda*. Europe, Cyprus, Turkey, Uzbekistan, Russia, Israel, Japan, Kyrgyzstan, Canary Islands, Azores, Egypt, Tunisia, South Africa, Australia, New Zealand, Taiwan, Thailand (Papp and Černý 2020; Černý and Merz 2006). Iran (Hazini et al. 2013).

###### Type material.

***Syntypes* [*biseriata*]: Germany.** Near Berlin (♂♀, ZMHU). [Not examined]

***Holotype* [*genualis*]: USA. DC**: “Loew coll., *genualis* m.” (1♀, MCZ; type No. 13430). [Not examined]

***Syntypes* [*nannodes***]: “Aus dem Jakutsk-Gebiet, Ost-Sibirien, aus Dyn-nan-yn, N.-Alashan, China und aus Japan, Misaki, Kiu-Shiu” (type data unknown, NMW). [Not examined]

***Holotype* [*plantaginicaulis*]: France.** “La Baule, Loire inf.” (♂♀, ZMHU). [Not examined]

***Syntypes* [*plantaginis*]: France** [not given] ([type information not given], UMO?). [Not examined]

***Holotype* [*Robinaldi*]: France.** [not given] ([type information not given], UMO?). [Not examined]

###### Material examined.

**Australia.** NSW: Bowral, 30.i.1961, [K.A. Spencer], CNC480142, CNC480145 (2♀, CNC). **Bermuda.** St. Georges, 28.i.1936, A.L. Melander (11♀, USNM). **Canada. BC**: Aiken, 12.vi.1957, J.R. Vockeroth, CNC480160 (1♀, CNC). **England.** Busk[?], Slough, “14/6 em. 27/6/39”, ex. *Plantagolanceolata*, det. and coll. O.W. Richards (1♂, USNM), Berry Head, Brixham, 1.ix.1960, J.R. Vockeroth, CNC480173 (1♀, CNC), Devon, Newton Abbot, 22.vi.1960, J.R. Vockeroth, CNC480172 (1♀, CNC), Dor., Lyme Regis, 14.vi.1958, [K.A. Spencer], CNC480143 (1♀, CNC), Hampstead, 20.vi.1971, [K.A. Spencer], mine *Plantagomajor* em. 25.vi.1971, CNC480146 (1♂, CNC), S Devon, Hope, 17.ix.1954, [K.A. Spencer], *Plantagomajor* em. 11.x.1954, CNC480144 (1♀, CNC), Torquay, 17.vi.1960, J.R. Vockeroth, CNC480166, CNC480168, CNC480169 (1♂ 2♀, CNC), 25.vi.1960, CNC480170 (1♀, CNC), 30.vi.1960, CNC480167, CNC480171 (1♂ 1♀, CNC). **Germany.** Junsternheida, M. Hering, 30.vi.1922, *Plantagomajor* (1♂ 3♀, USNM), Berlin, 24.vi.1932, Hering, *Plantagomajor* (1?, USNM). **New Zealand.** Bay of Plenty: Coromandel, 26.xii.1974, [K.A. Spencer], CNC480140, CNC480141 (2♀, CNC), Rotoura, 13.xii.1974, [K.A. Spencer], mine *Plantago*, em. 16–20.xii.1974, CNC480137–480139 (3♀, CNC), Southland: Bluff, 24.i.1975, [K.A. Spencer], mine *Plantago*, em. 1.ii.1975, CNC480136 (1♀, CNC). **Spain.** Canary Islands: Tenerife, San Andreas, 14.ii.1963, [K.A. Spencer], CNC480135 (1♀, CNC), Extremadura: 23.1 km SW Talarrubias, Dense Patch of Flowers in Short Grassland, 38°52'N, 5°23'W, 430 m, 4.vi.2008, J. and R. Skevington, CNC311345, CNC311349 (2♂, CNC), Extremadura: 4 km NW Guadelupe, Along Creek in Oak Forest, 39°28'N, 5°22'W, 1027 m, 5.vi.2008, J. and R. Skevington, CNC287132, CNC287136, CNC287139, CNC287150, CNC287152, CNC287154 (3♂ 3♀, CNC), Extremadura: 5.7 km WSW Navalvillar de Ibor, Wet Meadow in Oak Forest, 39°35'N, 5°21'W, 1026 m, 5.vi.2008, J.andR. Skevington, CNC286150, CNC286195, CNC286202, CNC286247, CNC286254, CNC286289, CNC286299–286301, CNC286303 (5♂ 5♀, CNC). **USA. AR**: Hot Springs, 17.v.1979, K.A. Spencer (3♀, USNM), **CA**: Ukiah, 7.ix.1941, R.M. Bohart, ex. lawn grass (1♀, USNM), **DC**: Potomac Pk., 18.iv.1913, R.C. Shannon (1♀, USNM), **DE**: Newark, 22.vi.1955 (1♀, UDCC), **IN**: Lafayette, 26.vii.1916, J.M. Aldrich (1♀, USNM), Michigan City, 29.vi.1915 (4♀, USNM), **GA**: Pine Mt., 1mi N, 12.vii.1957, W.R. Richards, CNC480149 (1♀, CNC), **KS**: Lawrence, 23.v.1934, M.W. Sanderson (2♀, USNM), **MA**: Concord, 27.vii.1961, marshy pond, W.W. Wirth (1♀, USNM), **MD**: Cabin john, 20.vi.1916, R.C. Shannon (1♂, USNM), Cabin John Bridge, “vi-6”, J.M. Aldrich (9♀, USNM), Beltsville, 3.vii.1926, W.L. McAtee (1♀, USNM), Glen Echo, J.M. Aldrich [no date] (14♀, USNM), 28.v.1919 (4♀, USNM), 29.v.1919 (25♀, USNM), Montgomery Co., Chevy Chase, "Woodend”, 7.vi.1975, G.F. Hevel (1♀, USNM), P.G. Co., Temple Hills 17.vii.1978, G.F. Hevel (1♀, USNM), Baltimore Co., Soldiers Delight, 3 km N Deer Park, 29.x.1986, W.E. Steiner, J.M. Swearington and J.M. Hill (1♀, USNM), **MI**: Isle Royale, 3–7.viii.1936, C. Sabrosky (1♂, USNM), E Lansing, 29.v.1937, C. Sabrosky (1♀, USNM), **NC**: Franklin, 609 m, 10.vi.1957, J.R. Vockeroth, CNC480152–480154 (3♀, CNC), 8.v.1957, J.R. Vockeroth, CNC480155–480159 (5♀, CNC), Highlands, 14.vii.1957, W.R. Richards, CNC480148 (1♀, CNC), Highlands, 1158 m, 17.viii.1957, J.G. Chillcott, CNC480147 (1♂, CNC), Toxaway, 30.viii.1957, W.R. Richards, CNC480150, CNC480151 (2♀, CNC), **NY**: Pocantico Hills, 22.vi.1935, C.W. Sabrosky (1♀, USNM), Oswego Co., Pulaski, Hinman Rd., 30.viii.1997, sweep net, old field, 30.viii.1997 (1♀, UDCC). **UT**: Salt Lake, on *Plantagolanceolata*, P.H. Timberlake, [various dates].vi.1914 (8♀, USNM), **VA**: Shenandoah, Big Meadows, 5.vii.1939, A.L. Melander (1♀, USNM), Blacksburg, 640 m, 28.v.1962, J.G. Chillcott, CNC480161, CNC480163, CNC480164 (3♀, CNC), 29.v.1962, J.G. Chillcott, CNC480162 (1♀, CNC), Giles Co., Mountain Lake, 975 m, 28.v.1962, J.G. Chillcott, CNC480165 (1♀, CNC), **WA**: Kamiac Butte, 1.vi.1912, J.M. Aldrich (1♀, USNM). **No locality data**: *Phyt.plantaginis* ♂ det. Hendel (1♂, USNM).

###### Comments.

The missing hind notopleural seta, not previously noted for *Phytomyzaplantaginis*, is highly diagnostic, and only otherwise found in North America in the Californian species *P.minutissima* Spencer. The latter is slightly smaller with only one ors, the head is anterodorsally projecting, and the phallus is small and membranous.

##### Phytomyza sehgali

Taxon classificationAnimaliaDipteraPhytomyzinae

Spencer

Figs 819–821

Phytomyza sehgali Spencer, 1969: 274. Sehgal 1971: 376.

###### Description.

Wing length 2.2 mm (♂), 2.2–2.4 mm (♀). Vein dm-m absent. Eye height divided by gena height: 3.0–3.8. First flagellomere rounded, distal hairs slightly longer, especially dorsally. Cheek narrow.

***Chaetotaxy***: Two ori, anterior seta slightly shorter to 2/3 length; two ors, posterior seta slightly shorter. Ocellar seta slightly longer than fronto-orbitals, postocellar more so. Four dorsocentrals, one presutural, decreasing in height anteriorly. Acrostichal setulae in six rows.

***Colouration***: Body with slight brownish pruinosity that is slightly thicker dorsally on thorax. Head light brown with antenna, clypeus, palpus, back of head, ocellar tubercle, posterior margin of frons and fronto-orbital plate dark brown; face and gena paler, yellowish. Remainder of body dark brown, with apices of femora (more so on fore leg) yellowish, and notopleuron and postpronotum sometimes with slightly paler tint. Halter white. Calypter margin and hairs light brown. Abdomen dark brown.

***Genitalia***: (Figs 819–821) Hypandrium well-developed, rounded apically; inner lobe bent at midpoint and with two apical setae. Postgonite strongly narrow in profile, flattened medially, with one seta. Basiphallus relatively short, dark, and well-sclerotised along dorsum, weaker with irregular margin ventrally; left sclerite with ventrobasal arm. Hypophallus with well-defined and sclerotised lateral margin, sometimes fused to pair of medial plates that are somewhat H-shaped and joined ventromedially. Base of mesophallus flanked by converging rod-like paraphalli. Mesophallus constricted and angled upwards at midpoint, narrower basally, apically widening to distiphallus, with ventral suture. Distiphallus divided into one pair of narrow, shallowly sinuate tubules. Ejaculatory apodeme with short, ill-defined stem and narrow blade with clear margin; sperm pump clear with transverse sclerotised band.

###### Host.

Unknown – reared from “thimbleweed” (possibly *Anemone* sp., Ranunculaceae) and “thimbleberry” (possibly *Rubusparviflorus* Nutt., Rosaceae).

###### Distribution.

**Canada**: AB. **USA**: MD*.

###### Type material.

***Holotype*: Canada. AB**: Edmonton, Whitemud Ck., 23.vi.1966, K.A. Spencer (1♂, CNC).

###### Additional material examined.

**USA. MD**: Elkton, 86-23, DFB, em. 1.v.1987, x Thimbleweed (1♂, UDCC), x Thimbleberry (2♀, UDCC).

###### Comments.

The Maryland material examined here greatly extends he known distribution of this species, which is known for the first time in the United States.

##### Phytomyza pulchelloides

Taxon classificationAnimaliaDipteraPhytomyzinae

Henshaw & Howse

Figs 822–824

Phytomyza pulchelloides Henshaw & Howse, 1989: 84 [new name for P.pulchella].
Phytomyza pulchella Spencer in Spencer and Steyskal 1986b: 320 [junior homonym of P.pulchellaSpencer 1977: 228].

###### Description.

Wing length 3.2–3.7 mm (♂), 3.4–4.1 mm (♀). Vein dm-m absent. Eye height divided by gena height: 2.4–5.1. First flagellomere well-developed, slightly longer than high, apically rounded and with dorsal and ventral margins parallel in part. Ocelli forming equilateral triangle, ocellar triangle indistinct. Epistoma present, not exceptionally large with width 1/4–1/2 length. Parafacial and fronto-orbital plate produced towards anterodorsal margin of eye. Cheek narrow, elongate, not continuing along eye posteriorly.

***Chaetotaxy***: Three or four ori; one ors. Ocellar and postocellar setae longer than fronto-orbitals. Four dorsocentrals, decreasing in length anteriorly. Four rows of acrostichal setulae.

***Colouration***: Head dark brown with first flagellomere darker, centre of frons and gena (excluding ventral margin) paler, but dark ocellar spot usually extending outside tubercle, labellum contrastingly yellow. Thorax dark brown with small paler spot medially on notopleuron and at anterior and posterior corner of postpronotum. Wing veins yellow basally to brownish apically. Halter white. Calypter entirely yellow. Legs dark brown with apices of femora yellow for distance equal to width of femur apex; base and apex of tibiae gradually light brown to yellowish. Abdomen brown to dark brown with posterior (and sometimes posterolateral) margin of tergites and laterobasal margin of epandrium yellowish.

***Genitalia***: (Figs 822–824) Hypandrium subtriangular to evenly rounded apically; inner lobe narrow and strongly bent medially with one seta at elbow; adjoining membrane haired. Postgonite narrow when viewed laterally, strongly flattened medially. Phallophorus flanked by one pair of long bars that are darker and pointed apically. Halves of basiphallus narrow with apical 1/3 expanded, plate-like; crossing basally, left sclerite with ventrobasal arm. Hypophallus with one pair of apically converging bars that are elongate, apically pointed, and flanking dark, upcurved medial V-shaped sclerite. Paraphallus absent. Duct pigmented apically. Mesophallus dark and cylindrical with rounded base, length 3 × width, fused via narrow band to distiphallus. Distiphallus divided into one pair of narrow dark tubules that are slightly outcurved apically, but are strongly curved dorsally on basal 2/3. Ejaculatory apodeme with short stalk and very broad, rounded apical blade; sperm pump with strong transverse sclerite.

###### Distribution.

**USA**: NY*, VA.

###### Host.

Likely Ranunculaceae (Spencer and Steyskal 1986b).

###### Type material.

***Holotype*: USA. VA**: Smyth Co., Mt. Rogers, 4700–5800 ft, 1.vi.1962, J.R. Vockeroth (1♂, CNC).

###### Paratypes examined.

**USA. VA**: Same collection as holotype, CNC480185–480187 (3♂, CNC).

###### Additional material examined.

**USA. NY**: Ithaca, 17.v.1936, H.K. Townes, A.L. Melander (1♀, USNM), Bear Mtn., 31.v.1937, A.L. Melander (1♂ 1♀, USNM), **VA**: Fairfax Co., Turkey Run Park, 0.3 km W mouth Turkey Run, 38°58'N, 77°09.6'W, Malaise trap, river, 14–17.v.2006, D.R. Smith (1♂, USNM).

##### Phytomyza verticillatae

Taxon classificationAnimaliaDipteraPhytomyzinae

Kulp

Figs 796–799

Phytomyza verticillatae Kulp, 1968: 25. Spencer and Stegmaier 1973: 123; Spencer and Steyskal 1986b: 214; Griffiths and Piercey-Normore 1995: 23; Scheffer and Wiegmann 2000: 249; Lonsdale and Scheffer 2011: 1201; Scheffer et al. 2021: 62.

###### Description.

As described for *P.ilicicola* except as follows:

Wing length 1.5–1.9 mm (♂), 1.7–2.0 mm (♀). Eye height divided by gena height: 4.3–6.0.

***Chaetotaxy***: Anterior ori minute. Four to five scattered rows of acrostichal setulae.

***Colouration***: Parafacial and side of face dark grey, centre of face yellowish. Posterolateral margin of frons dark to base of inner vertical seta; dark spot sometimes extending along eye margin to level of posterior ori and occasionally extending from margin to surround base of all fronto-orbitals. Legs slightly whitish with mid tibia sometimes paler.

***Genitalia***: (Figs 796–799) Surstylus short and tapered. Epandrial process relatively broad. Sclerite of hypophallus ill-defined, darkest near leading margin, but entire lateral and ventral surface of hypophallus lightly sclerotised to form an irregular cup. Paraphalli narrow, curved, diverging. Mesophallus ~ 11 × longer than wide at midpoint, apex broader, clearer, and U-shaped. Arms of distiphallus broadly separate, weakly diverging, and not much more than 1/2 length of mesophallus.

###### Hosts.

Aquifoliaceae – *Ilexverticillata*, *I.mucronata*.

###### Distribution.

**USA**: AL, DC, FL, MA, MD, ME, MI, MN, NY, PA, TN, TX, VA, WV.

###### Type material.

***Holotype*: USA. DC**: Washington, 6.vii.1964, L.A. Kulp, Type No. 67428 (1♂, USNM).

###### Additional material examined.

See Lonsdale and Scheffer (2011).

##### Phytomyza vockerothi

Taxon classificationAnimaliaDipteraPhytomyzinae

Winkler

Figs 825–827

Chromatomyia nigrella Spencer in Spencer and Steyskal 1986b: 328.
Phytomyza vockerothi Winkler in Winkler et al. 2009: 289 [replacement name for nigrella Spencer – secondary homonym of nigrella Hendel].

###### Description.

Wing length 1.7–2.0 mm (♂). Female unknown. Vein dm-m absent. Eye height divided by gena height: 2.8–3.1. First flagellomere small and rounded, not much higher than scape; hairs along anterodorsal margin slightly longer. Gena strongly narrowing anteriorly; cheek narrow, weakly continuing along posterior margin of eye.

***Chaetotaxy***: One or two ori; one or two ors (posterior ors no more than 4/5 length of anterior ors, but both ors subequal on one side of frons in holotype); two ori and two ors only seen together in holotype, where anterior ori 1/2-length on one side and absent on other. Ocellar and postocellar setae subequal to fronto-orbitals. Four dorsocentrals, one presutural, decreasing in height anteriorly. Acrostichal setulae in four scattered rows anteriorly; Spencer and Steyskal (1986b) notes 2 rows, possibly in unexamined paratype. Intra-alar setulae reduced in number.

***Colouration***: Body mostly dark brown; frons, sometimes including fronto-orbital plate, paler; palpus, ventral margin of gena, first flagellomere and ocellar tubercle darker; halter white; postpronotum and notopleuron with faint yellow tint or mottling; apices of femora yellow, fore tibia sometimes paler with base faintly yellow. Notum subshiny. Calypter hairs light brown, margin paler.

***Genitalia***: (Figs 825–827) Hypandrium subtriangular in outline, inner lobe sometimes with setae. Postgonite well-developed with one seta. Phallophorus flanked by one pair of small bands. Halves of basiphallus relatively broad, flat, situated roof-like dorsally; left sclerite with ventrobasal process. Hypophallus small, directed apically, composed of one pair of small, pale, converging finger-like sclerites; holotype with sclerites more parallel and with small, faint medial sclerotised line. Paraphallus similar to sclerite of hypophallus but thicker, converging below distiphallus. Mesophallus not evident. Distiphallus ill-defined, linear with two lightly sclerotised to membranous strips. Ejaculatory apodeme small and dark with apex barely widened.

###### Host.

Unknown.

###### Distribution.

**Canada**: NS*. **USA**: MD*, NC, VA*, WV*.

###### Type material.

***Holotype*: USA. NC**: Macon Co., Highlands, 3800ft, 8.v.1957, J.R. Vockeroth (1♂, CNC).

###### Additional material examined.

**Canada. NS**: Mount Uniacke, 5.viii.1958, J.R. Vockeroth, CNC480188 (1♂, CNC). **USA. MD**: Colesville, 4.vi.1977, W.W. Wirth (1♂, USNM), **VA**: Shenandoah, Big Meadows, 2.vii.1939, A.L. Melander (1♂, USNM), Giles Co., Mtn. lake, 9.ix.1970, G. Steyskal (2♂, USNM), **WV**: Parkersburg, 21.vi.1970, G. Steyskal (2♂, USNM), Ritchie Co., North Bend St. Pk., 23.vi.1970, G. Steyskal (2♂, USNM).

###### Comments.

*Phytomyzavockerothi* is relatively difficult to diagnose externally, being similar to many other small, dark congeners, but the phallus is unmistakable. There is one pair of narrow sclerites on the hypophallus and one pair of similarly narrow paraphalli converging in front of a very pale, linear distiphallus. It is here recorded in Canada for the first time.

##### Phytomyza wiggii

Taxon classificationAnimaliaDipteraPhytomyzinae

Lonsdale & Scheffer

Figs 800 , 801

Phytomyza wiggii Lonsdale & Scheffer, 2011: 1204. Scheffer et al. 2021: 62.

###### Description.

As described for *P.ilicicola* except as follows:

Wing length 1.8–2.5 mm (♂), 2.1–2.6 mm (♀). Eye height divided by gena height: 5.3–6.0.

***Chaetotaxy***: Anterior ori 1/2 length of posterior ori. Four to five scattered rows of acrostichal setulae.

***Colouration***: Face yellowish centrally and dark grey laterally. Posterolateral corner of frons and fronto-orbital plate dark, excluding paler anterior and inner-anterior margins. Notum sometimes with faint to distinct yellowish spots on anterior and posterior margins of anepisternum. Legs brown with tibiae and tarsi paler or with yellowish to white mottling, particularly on fore and mid legs.

***Genitalia***: (Figs 799–801) Surstylus broadly rounded, short. Epandrial process short, slightly downturned. Lateral sclerite of hypophallus narrow on basal 1/2, broader distal 1/2. Paraphallus small, lobate, and curved laterally. Mesophallus ~ 6 × longer than width at midpoint. Arms of distiphallus separate, narrow, and subparallel on basal 1/2.

###### Hosts.

Aquifoliaceae – *Ilexverticillata*, *I.mucronata*, *I.longipes* (Scheffer et al. 2021).

###### Distribution.

**Canada.** ON*. **USA**: GA, MA, MD, ME, NC, NY, PA.

###### Type material.

***Holotype***: **USA. PA**: Dauphin Co., behind rest area on southbound I-81, 11.x1999, coll. S.J. Scheffer, ex *Ilexverticillata* (1♂, USNM).

###### Additional material examined.

**Canada. ON**: [Ottawa], Constance Bay, Freeman and Lewis, *Ilexverticillata*, see also leaf mines colln., em, 28.ii.1957, 56–205, CNC480189–480195 (2♂ 4♀ 5 puparia [gel capsule], CNC). **USA. MA**: Berkshire Co., Savoy, 1.vi.2013, C.S. Eiseman, ex *Ilexverticillata*, em. 14–24.vi.2013, #CSE561, CNC392711, CNC392712 (1♂ 1 ex, CNC), Franklin Co., Northfield, Crag Mountain, 16.x.2013, C.S. Eiseman, ex. *Ilexmucronata*, em. 11.iii.2014, #CSE998, CNC384759–384777 (10♂ 9♀, CNC), Hampshire Co., Cummington, 18.v.2013, C.S. Eiseman, ex *Ilexverticillata*, em. 1.vi.2013, #CSE549, CNC392689–392700 (6♂ 6♀, CNC), Nantucket Co., State Forest, 10.vi.2013, C.S. Eiseman, ex. *Ilexverticillata*, em. 16.vi.2013, #CSE565, CNC384810 (1♂, CNC). Also see Lonsdale and Scheffer (2011).

###### Comments.

*Phytomyzawiggii* is here recorded for the first time in Canada.

##### Phytomyza winkleri sp. nov.

Taxon classificationAnimaliaDipteraPhytomyzinae

http://zoobank.org/684853B2-EB34-48F6-AA37-F8BF3930CBE7

Figs 828–832

###### Description.

Wing length 1.8 mm (♂). Female unknown. Vein dm-m absent. Eye height divided by gena height: 3.7. First flagellomere relatively large, 1/3 height of eye, slightly longer than high, hairs along distal margin slightly longer. Ocellar triangle not much larger than tubercle and rounded. Parafacial projecting, slightly narrower than cheek; fronto-orbital plate slightly projecting medially, becoming more pronounced anteriorly.

***Chaetotaxy***: One ori; two ors; all subequal. Ocellar seta slightly longer than fronto-orbitals, postocellar more so. Few orbital setulae. Four dorsocentrals, one presutural, slightly decreasing in height anteriorly. Acrostichal setulae irregular and few, variably appearing in two to four rows around and slightly posterior to suture. Few intra-alar setulae around suture.

***Colouration***: Head partially yellow, with antenna black, back of head, ocellar triangle (barely larger than tubercle) and ventral margin of gena dark brown; palpus and clypeus brown; posterolateral corner of frons dark brown to base of inner vertical seta, dark spot continuing anteriorly along fronto-orbital plate to disappear between bases of ors. Thorax dark brown with grey pruinosity that is denser dorsally; pruinosity also seen more thinly across remainder of body, especially along dark lateral regions of frons. Halter white. Calypter margin and hairs brownish. Legs mostly dark brown; coxae brownish, fore coxa dark towards base; apex of femora and bases of tibiae yellow. Abdomen dark brown.

***Genitalia***: Epandrium shallow, long setose posteriorly, completely fused to small surstylus. Postgonite broad and flat with large distoventral lobe and fused to hypandrium. Dorsal margin of phallophorus fused to single apically split basiphallus. Paraphallus originating slightly beyond apices of basiphallus, converging and becoming more plate-like apically; broken medially. Mesophallus not evident. Distiphallus pale and cylindrical, narrower at base, dark apically and weakly pigmented dorsally. Ejaculatory apodeme with small pale blade and broad base.

###### Host.

Unknown.

###### Distribution.

**USA**: MD.

###### Etymology.

The specific epithet recognises the collector of the holotype, I. Winkler, who provided the specimen to the author after recognising it to be an undescribed species.

###### Type material.

***Holotype*: USA. MD**: Montgomery Co., Pautuxent State Park, below Brighton dam, W. of Columbia, 39°11.39'N, 77°0.29'W, 8.v.2005 IS Winkler (1♂, USNM).

###### Comments.

The phallus of *Phytomyzawinkleri* superficially suggests a close association to species of the subgenusNapomyza, but unlike many members of this subgenus, vein dm-m is absent, making it more similar in appearance to other *Phytomyza* s. s. While its position within the genus is still unclear, a relationship to *P.avicursa* is suggested in the structure of the hypandrial complex, mostly in the dorsal position of the split basiphallus and the long perpendicular paraphallus. Both are further characterised by a dark, grey pruinose notum, dark legs with yellowish knees, a black antenna with the first flagellomere relatively large and one ori (two present on one side in the *P.avicursa* type).

**Figures 78–83.**
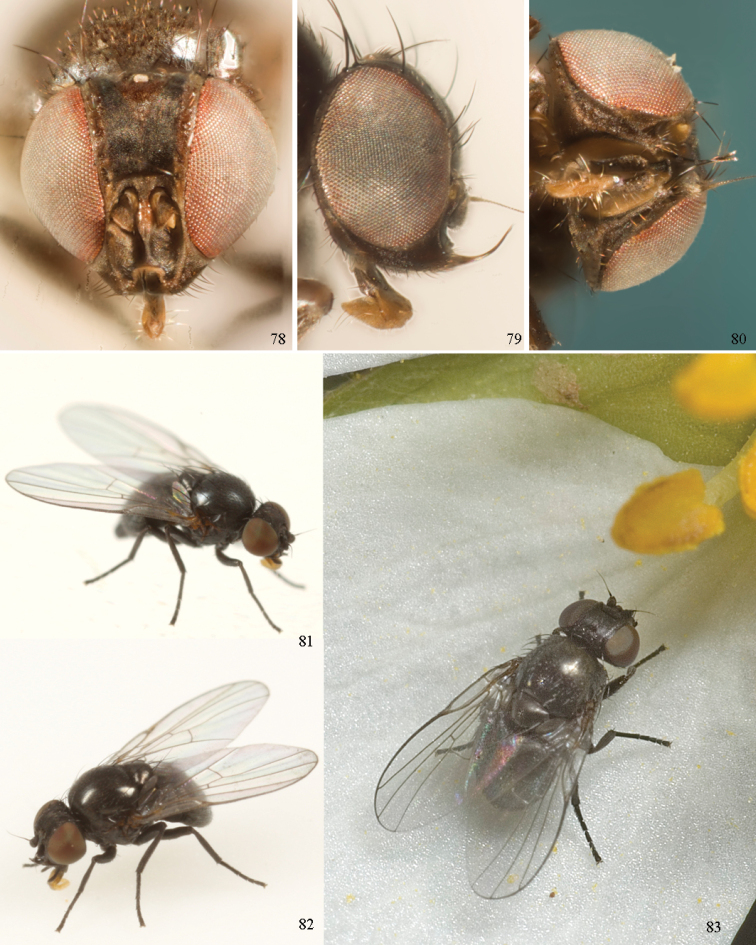
*Ophiomyialabiatarum* Hering, head **78** anterior **79** lateral **80** ventral **81***Ophiomyiacarolinensis* Spencer, female (C. Eiseman – Massachusetts) **82***Ophiomyiaconiceps* (Malloch), male (C. Eiseman – Massachusetts) **83***Ophiomyia* sp. (S.A. Marshall – Virginia, Butt Mtn.).

**Figures 84–91.**
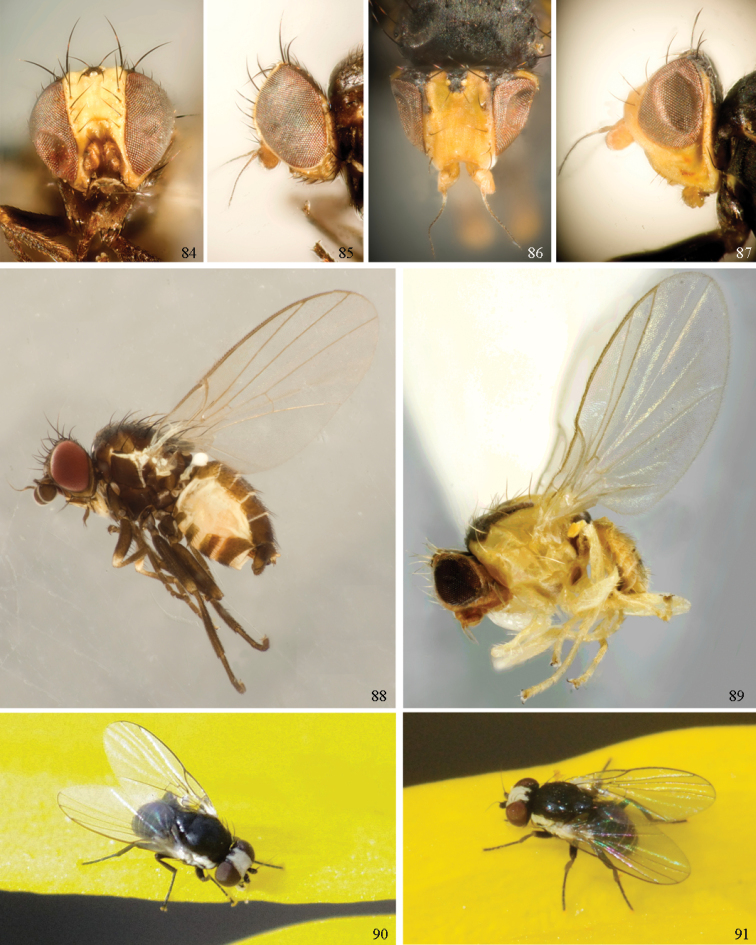
*Amauromyzaflavifrons* (Meigen), head **84** anterior **85** lateral **86, 87***Amauromyzakarli* (Hendel), head **86** dorsal **87** lateral **88***Aulagromyzanitida* (Malloch), male, lateral **89***Aulagromyzatridentata* (Loew), male, lateral **90, 91***Calycomyzaenceliae* Spencer, male, on bush sunflower (R. Hemberger – California, Irvine).

**Figures 92–98.**
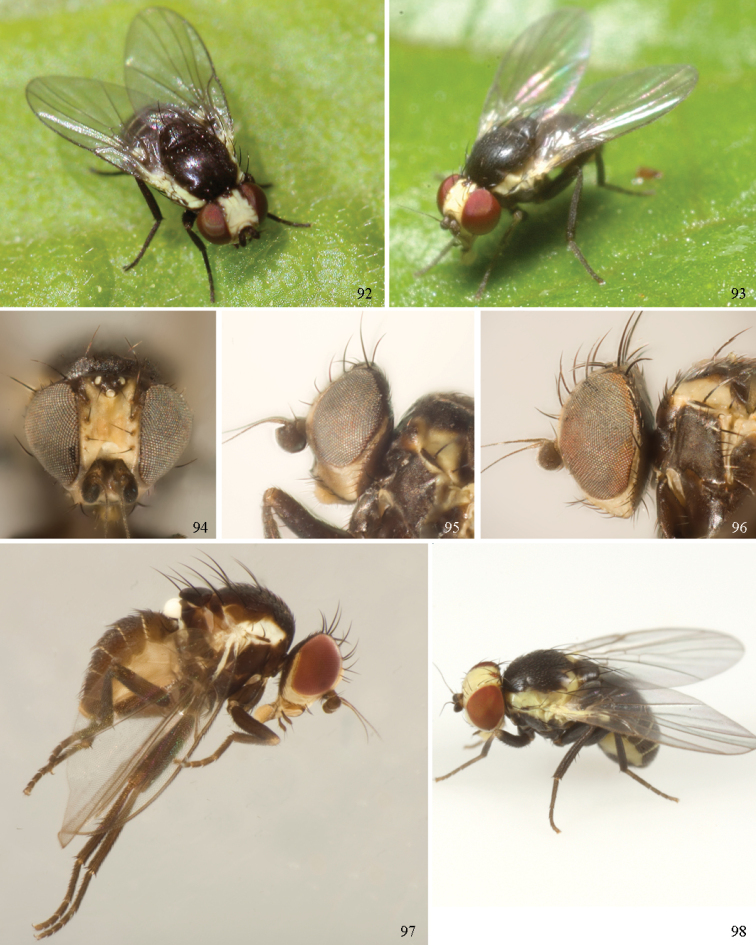
*Calycomyza* sp. (T. Bentley – Illinois, Reed-Turner Woodland Nature Preserve) **93***Calycomyza* sp. (S.A. Marshall – Cuba, Santiago) **94, 95***Calycomyzahumeralis* (Roser), head **94** anterior **95** lateral **96***Calycomyzacynoglossi* (Frick), head, lateral **97***Calycomyzamalvae* (Burgess), male, lateral **98***Calycomyzaflavinotum* (Frick) (C.S. Eiseman).

**Figures 99–106.**
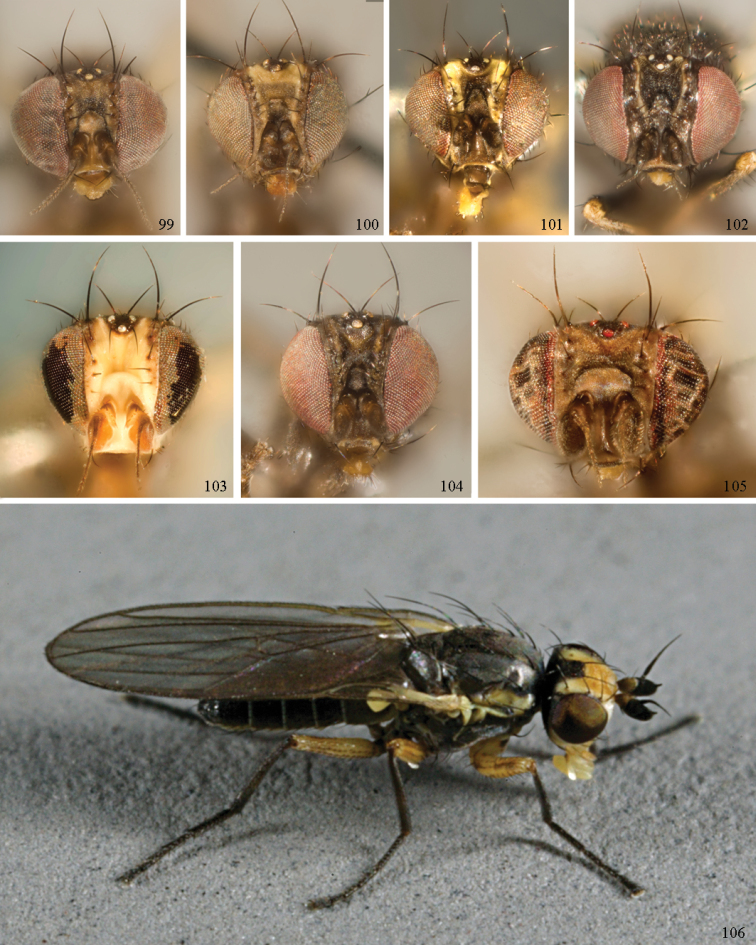
**99**Cerodontha (Butomomyza) angulata (Loew), head, anterior **100**Cerodontha (Phytagromyza) frankensis Spencer, head, anterior **101**Cerodontha (Poemyza) muscina (Meigen), head, anterior **102**Cerodontha (Poemyza) incisa (Meigen), head, anterior **103**Cerodontha (Icteromyza) longipennis (Loew), head, anterior **104**Cerodontha (Xenophytomyza) illinoensis (Malloch), head, anterior **105**Cerodontha (Dizygomuza) morosa (Meigen), head, anterior **106**Cerodontha (Cerodontha) dorsalis (Loew), (K. Matz – Utah, Salt Lake City).

**Figures 107–115.**
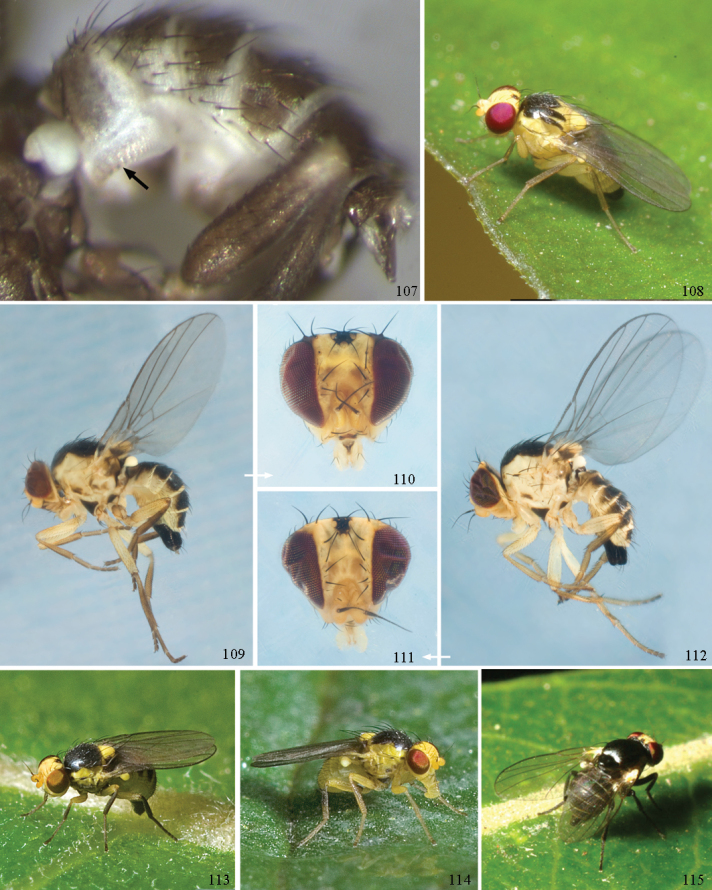
**107***Liriomyza* sp., male, with arrow indicating stridulatory file **108***Liriomyza* sp., female (S.A. Marshall – Cuba, Santiago) **109, 110***Liriomyzasativae* (Blanchard), female **109** lateral **110** head, anterior **111, 112***Liriomyzatrifolii* (Burgess), female **111** head, anterior **112** lateral **113***Liriomyzaasclepiadis* Spencer, female (S.A. Marshall – Virginia, Craig County) **114***Liriomyza* sp., female (S.A. Marshall – Ontario, Orangeville) **115***Liriomyzanigriscutellata* Spencer, female (P. Bryant – California, Newport Beach).

**Figures 116–123.**
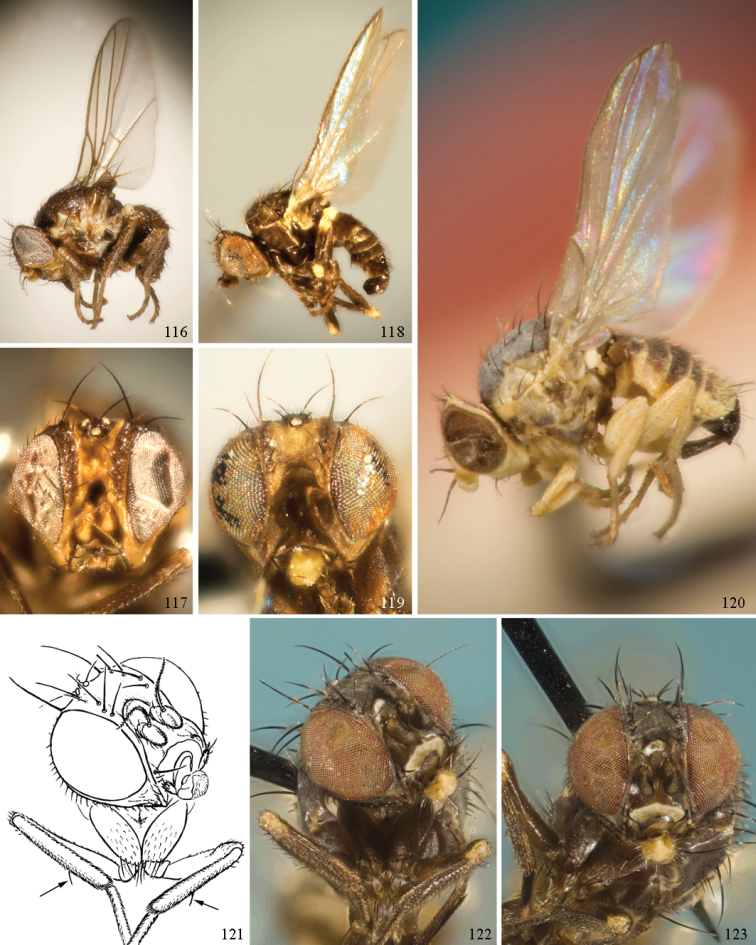
*Liriomyzaviolivora* (Spencer), male **116** lateral **117** head, anterior **118, 119***Metopomyzainterfrontalis* (Melander), male **118** lateral **119** head, anterior **120***Haplopeodesminutus* (Frost), female, lateral **121–123***Nemorimyzaposticata* (Meigen), anterior, with illustration showing lateral tibial bristles.

**Figures 124–130.**
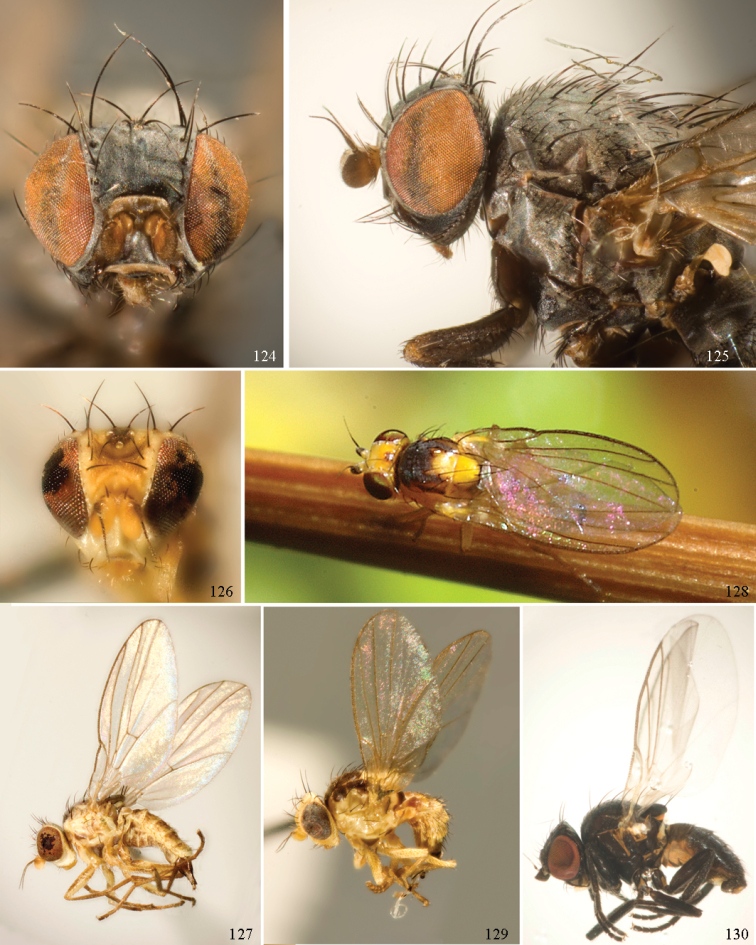
*Phytobiasetosa* (Loew) **124** head, anterior **125** lateral **126, 127***Phytoliriomyzaarctica*, female **126** head, anterior **127** lateral **128***Phytoliriomyzapacifica* (Melander) **129***Phytoliriomyzamelampyga* (Loew), male, lateral **130***Pseudonapomyza* sp., lateral.

**Figures 131–138.**
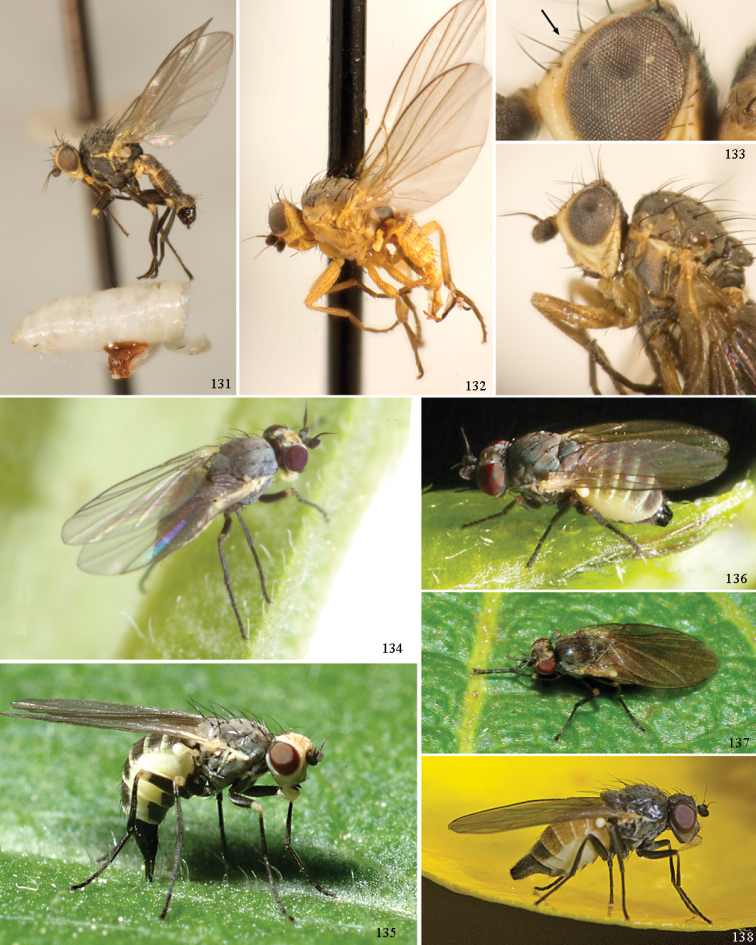
**131**Phytomyza (Napomyza) lateralis Fallén, female and puparium **132***Phytomyzacompta* (Spencer), male holotype, lateral **133***Phytomyzanervosa* Loew, lateral, with detail of proclinate orbital setulae (arrow) **134***Phytomyzacrassiseta* Zetterstedt, female (D. Cheung – Ontario) **135***Phytomyza* sp., female (S.A. Marshall – Hornings Mills) **136***Phytomyza* sp., female (B. Dupree – Georgia, Atlanta) **137***Phytomyza* sp., female (K. Hall – Washington, Lacey) **138***Phytomyzabicolor* Coquillett, female on *Calthapalustris* (S.A. Marshall – Ontario, Spring Creek).

**Figures 139–142.**
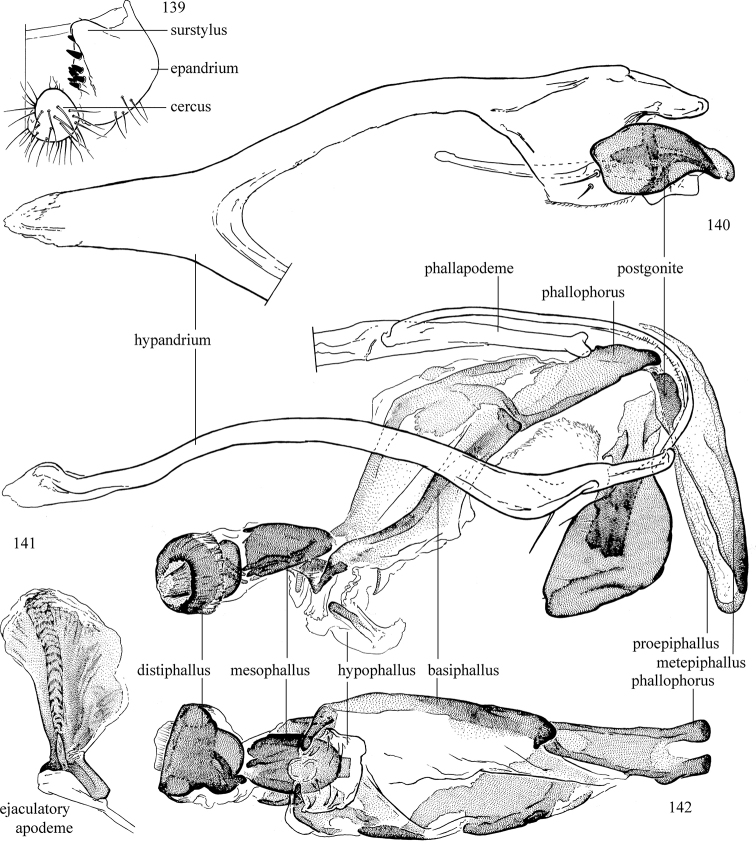
*Agromyzaabiens* Zetterstedt, male genitalia **139** external genitalia, ventral **140** hypandrium and postgonite, ventral **141** hypandrial complex, left lateral **142** phallus, ventral.

**Figures 143–146.**
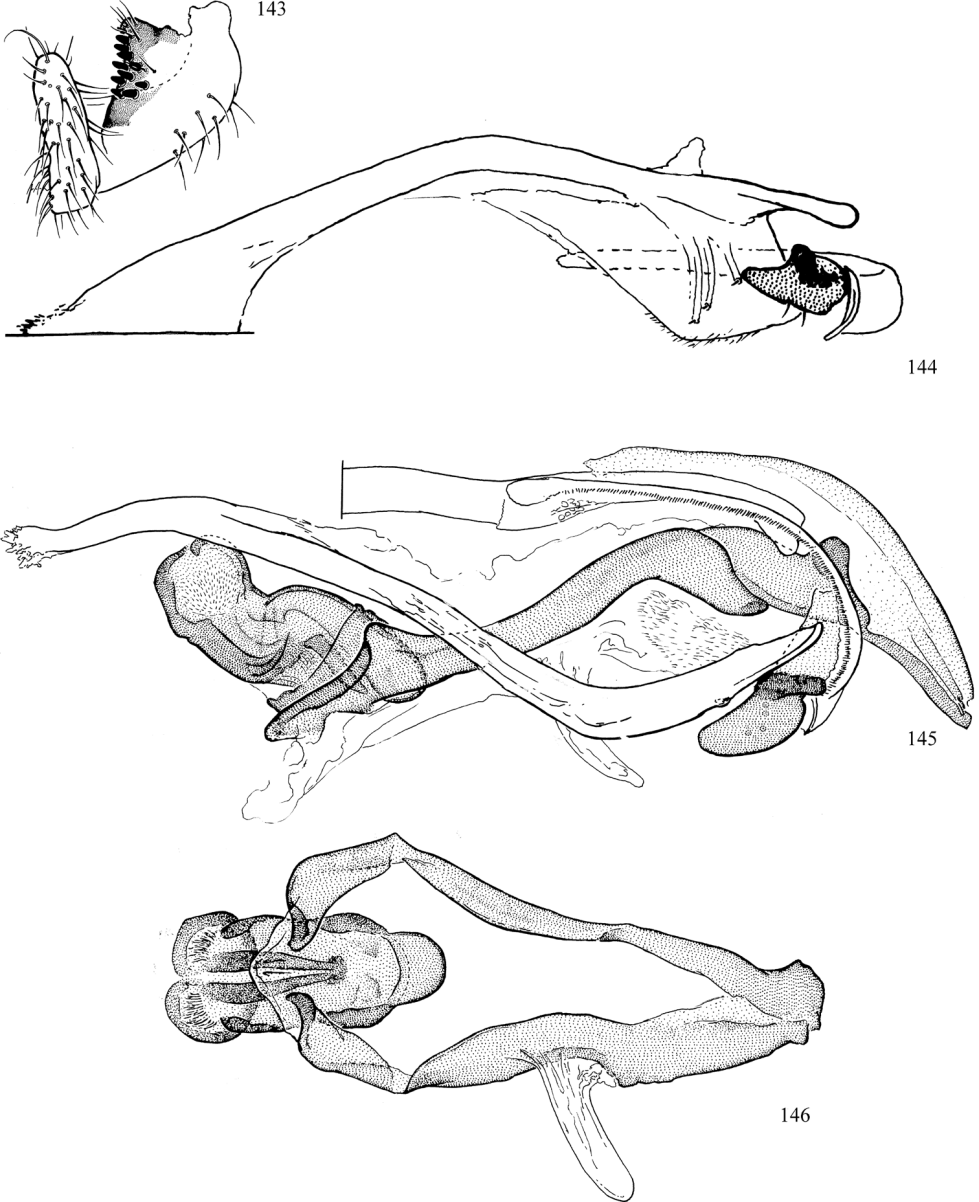
*Agromyzaalbipennis* Meigen, male genitalia **143** external genitalia, ventral **144** hypandrium and postgonite, ventral **145** hypandrial complex, left lateral **146** phallus, ventral.

**Figures 147–151.**
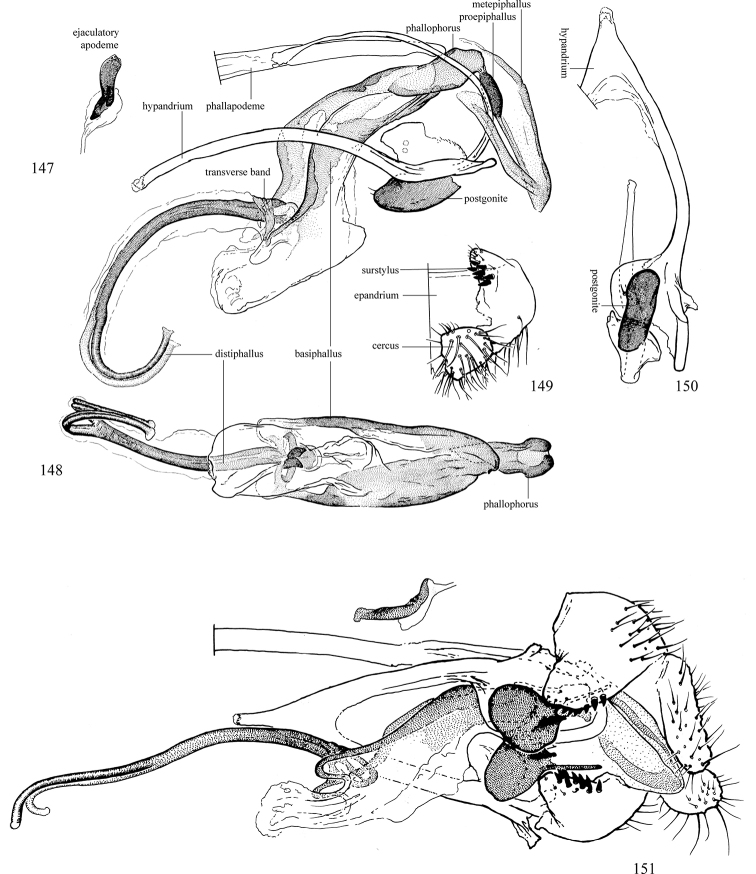
*Agromyzaambrosivora* Spencer, male genitalia **147** hypandrial complex, left lateral **148** phallus, ventral **149** external genitalia, ventral **150** hypandrium and postgonite, ventral **151***A.virginiensis* Spencer, male holotype genitalia.

**Figures 152–155.**
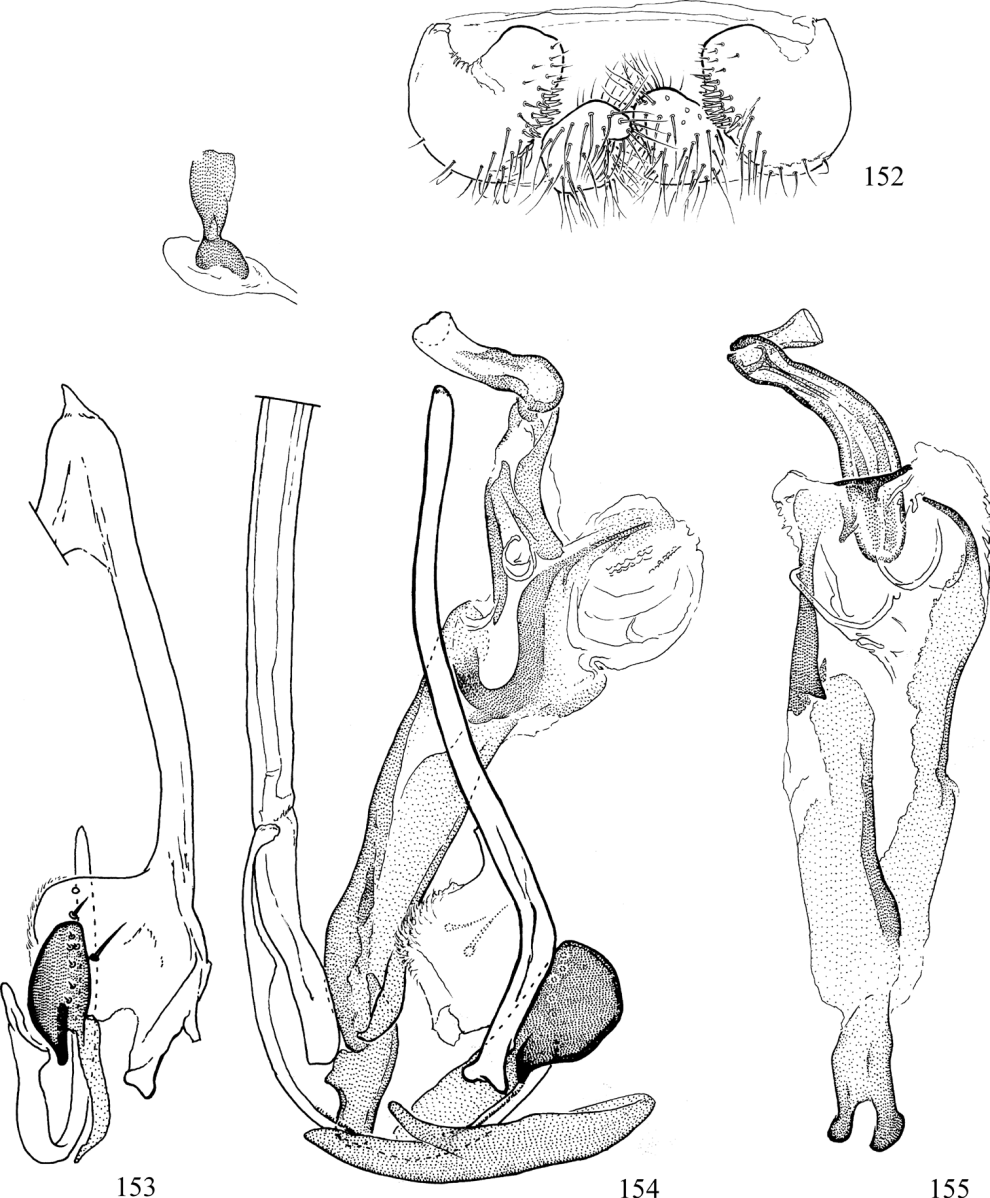
*Agromyzaapfelbecki* Strobl, male genitalia **152** external components, ventral **153** hypandrium and postgonite, ventral **154** hypandrial complex, right lateral **155** phallus, ventral.

**Figures 156–160.**
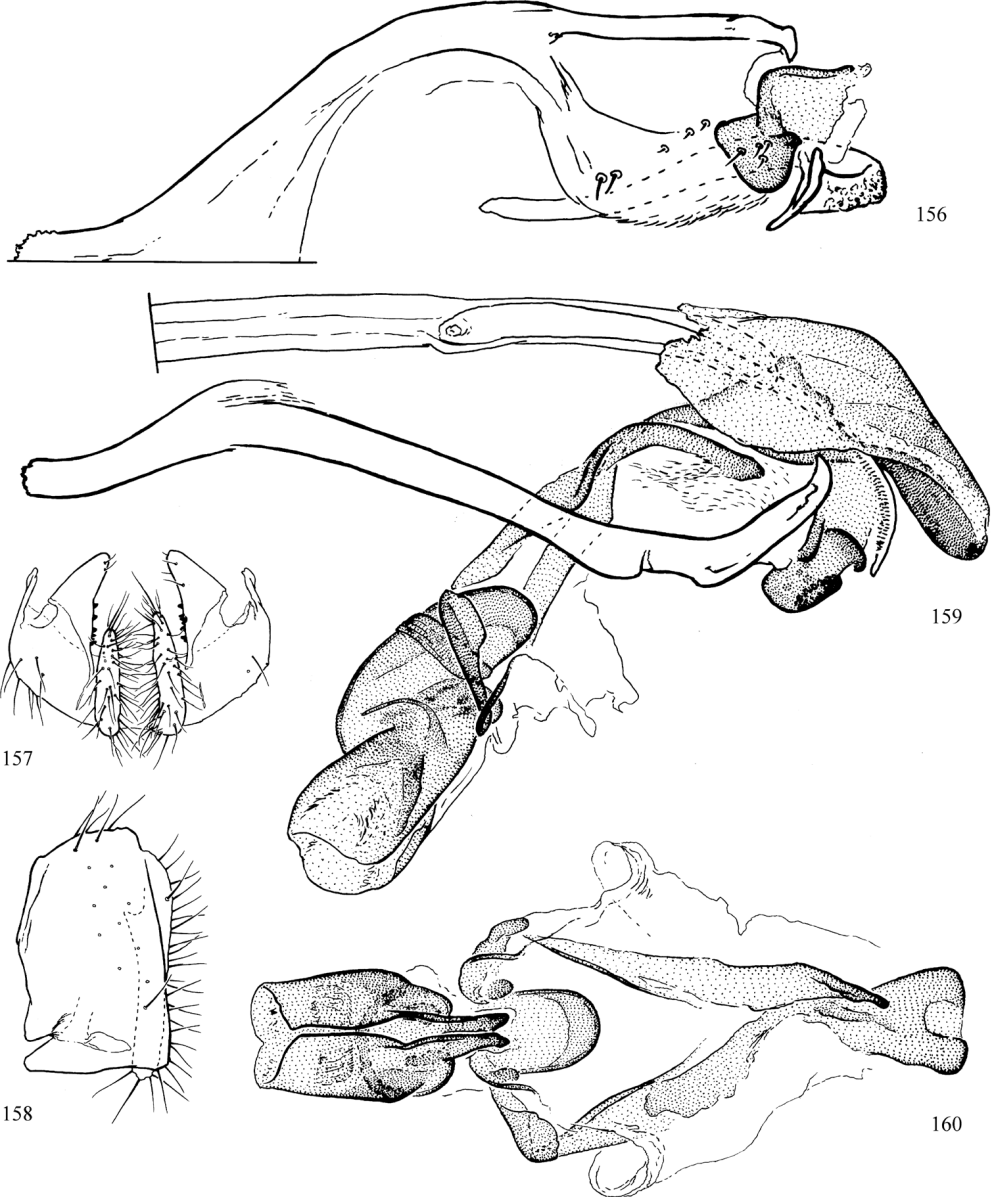
*Agromyzaaprilina* Malloch, male genitalia **156** hypandrium and postgonite, ventral **157** external genitalia, ventral **158** external genitalia, left lateral **159** hypandrial complex, left lateral **160** phallus, ventral.

**Figures 161–166.**
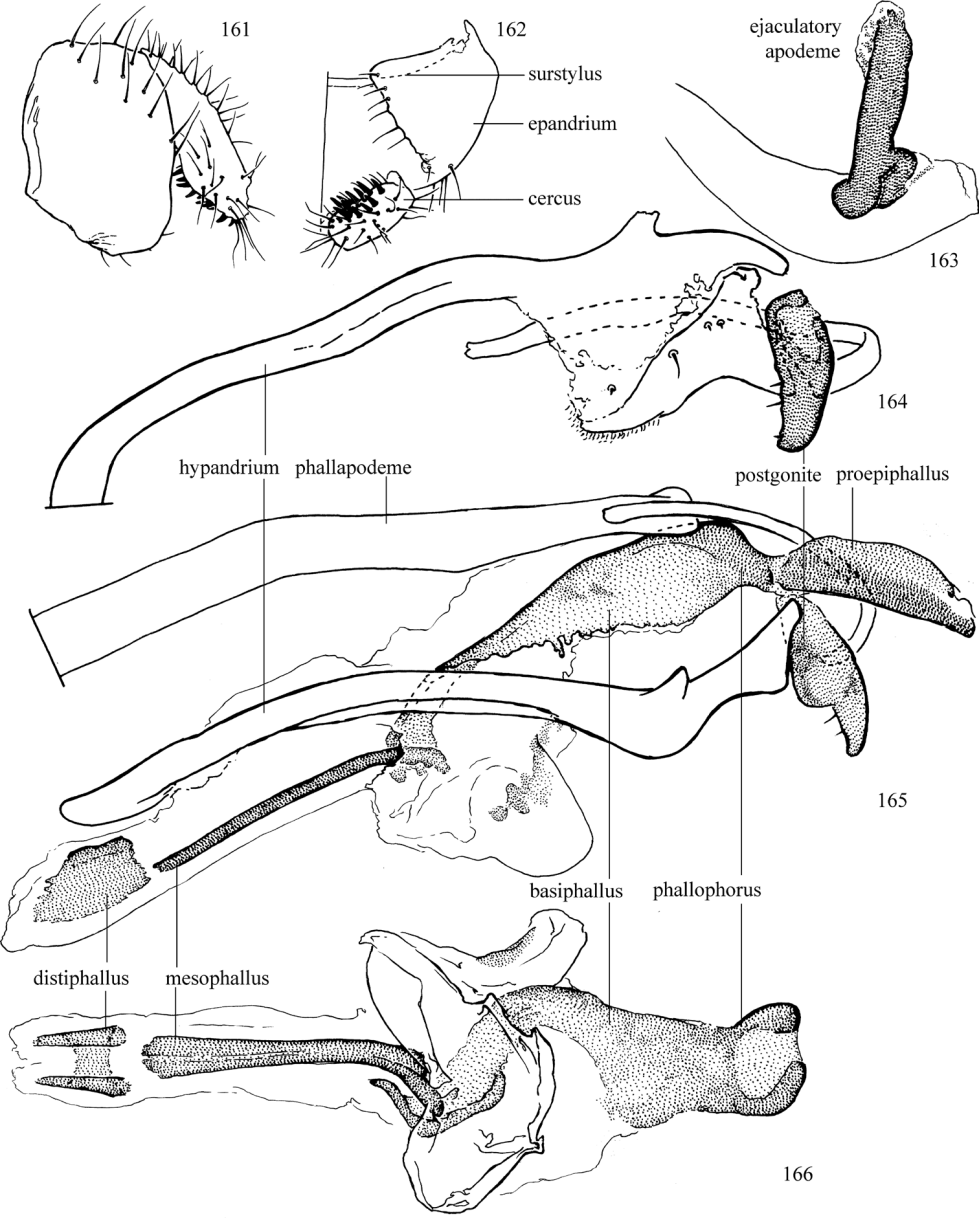
*Agromyzaaristata* Malloch, male genitalia **161** external genitalia, left lateral **162** external genitalia, ventral **163** ejaculatory apodeme **164** hypandrium and postgonite, ventral **165** hypandrial complex, left lateral **166** phallus, ventral.

**Figures 167–174.**
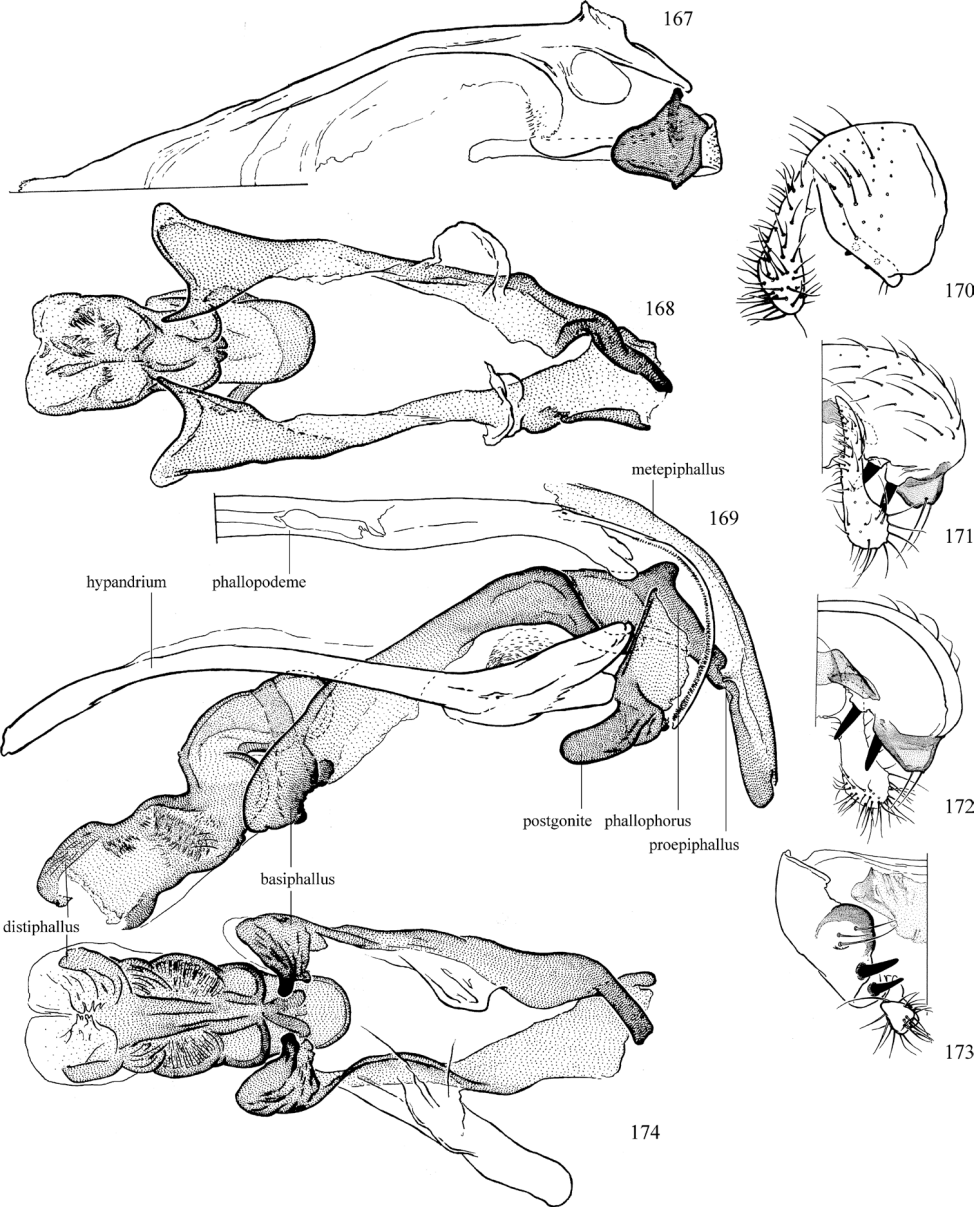
*Agromyzabispinata* Spencer, “typical” male genitalia **167** hypandrium and postgonite, ventral **168** phallus, ventral **169** hypandrial complex, left lateral **170** external genitalia, left lateral **171** external genitalia, posterior **172** external genitalia, anterior **173** external genitalia, ventral **174***A.bispinata*, longer, multi-chambered “atypical” male (Maryland specimen), phallus, ventral.

**Figures 175–178.**
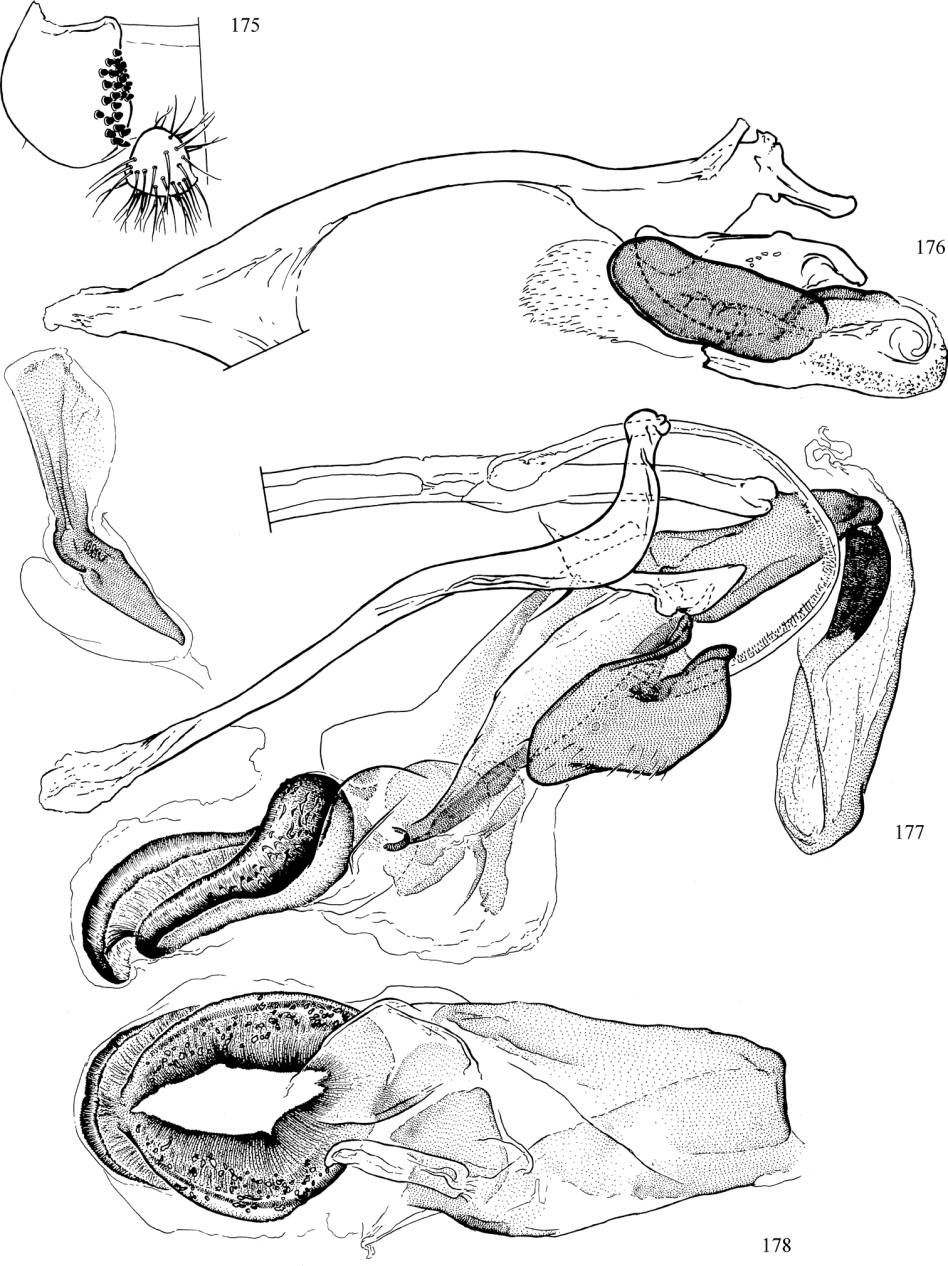
*Agromyzacanadensis* Malloch, male genitalia **175** external genitalia, ventral **176** hypandrium and postgonite, ventral **177** hypandrial complex, left lateral **178** phallus, ventral.

**Figures 179–182.**
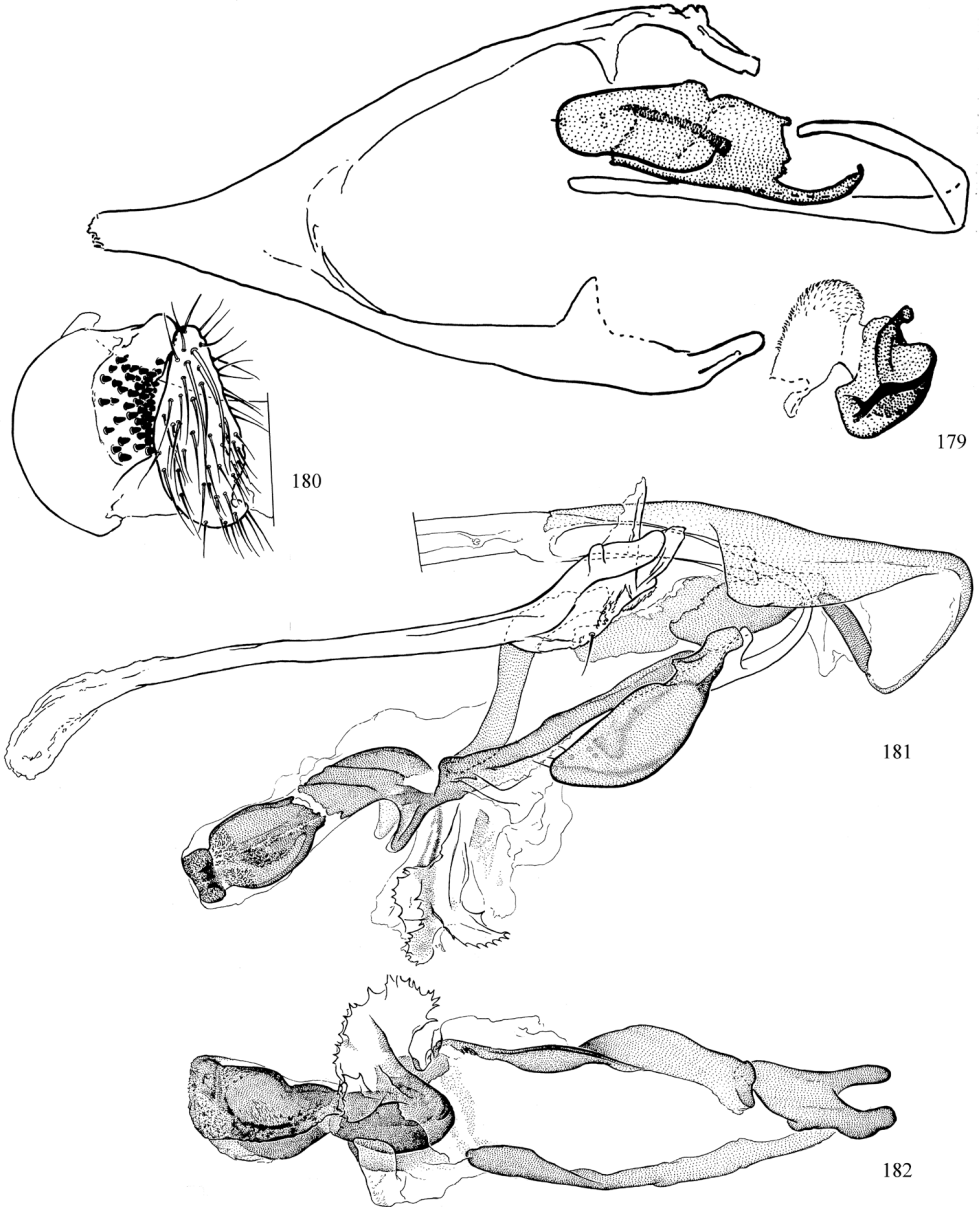
*Agromyzadiversa* Johnson, male genitalia **179** hypandrium and postgonite, ventral **180** external genitalia, ventral **181** hypandrial complex, left lateral **182** phallus, ventral.

**Figures 183–187.**
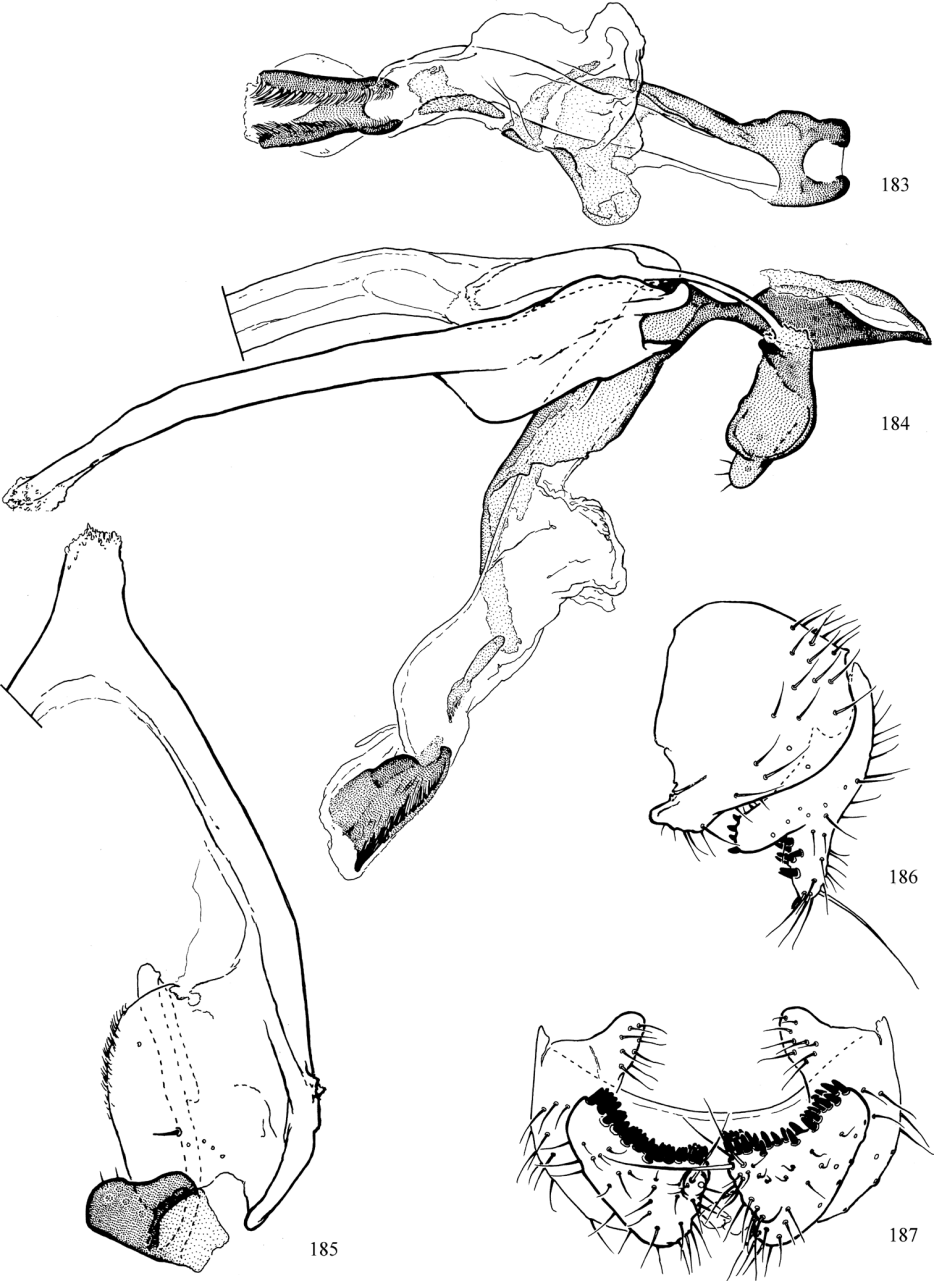
*Agromyzafission* Eiseman & Lonsdale, male genitalia **183** phallus, ventral **184** hypandrial complex, left lateral **185** hypandrium and postgonite, ventral **186** external genitalia, left lateral **187** external genitalia, ventral. Originally published in Eiseman and Lonsdale (2018).

**Figures 188–192.**
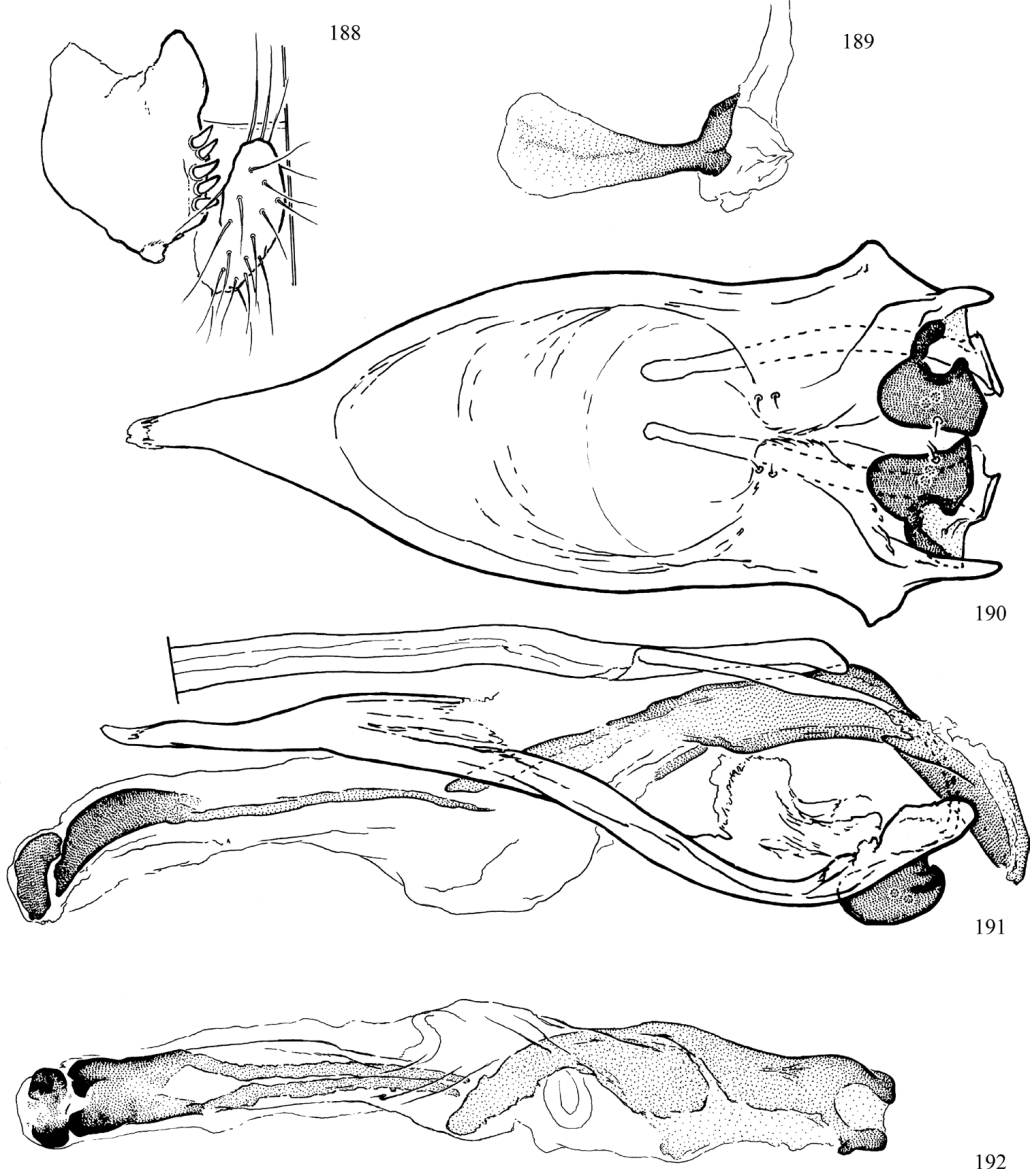
*Agromyzaisolata* Malloch, male genitalia **188** external genitalia, left lateral **189** ejaculatory apodeme **190** hypandrium and postgonite, ventral **191** hypandrial complex, left lateral **192** phallus, ventral.

**Figures 193–197.**
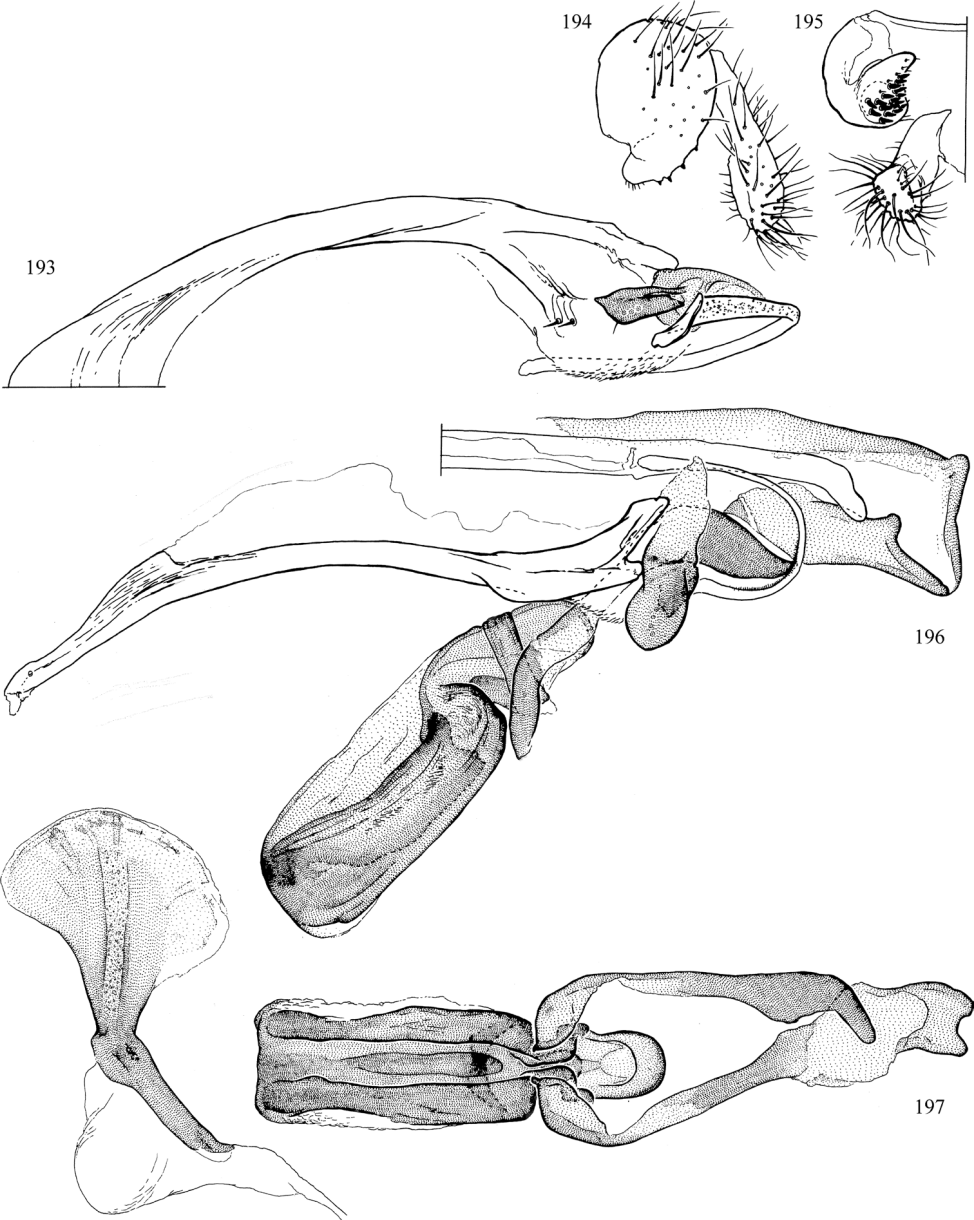
*Agromyzakincaidi* Malloch, male genitalia **193** hypandrium and postgonite, ventral **194** external genitalia, left lateral **195** external genitalia, ventral **196** hypandrial complex, left lateral **197** phallus, ventral.

**Figures 198–204.**
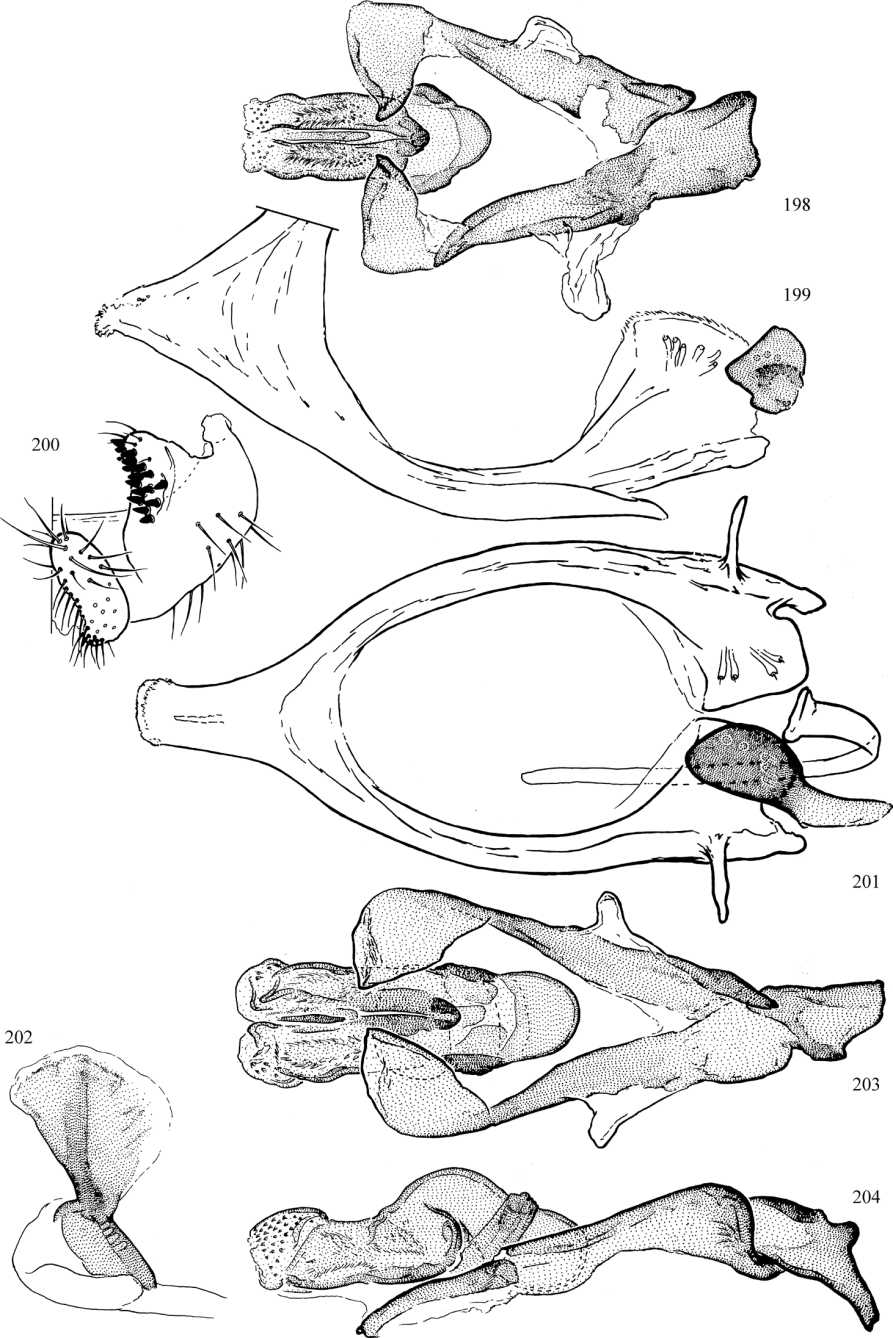
*Agromyzaparca* Spencer, genitalia of atypical males with white calypter **198** distiphallus, ventral **199** hypandrium and postgonite, ventral **200** external genitalia **201–204***A.parca*, genitalia of typical males with margin of calypter brown **201** hypandrium and postgonite, ventral **202** ejaculatory apodeme **203** phallus, ventral **204** phallus, left lateral.

**Figures 205–209.**
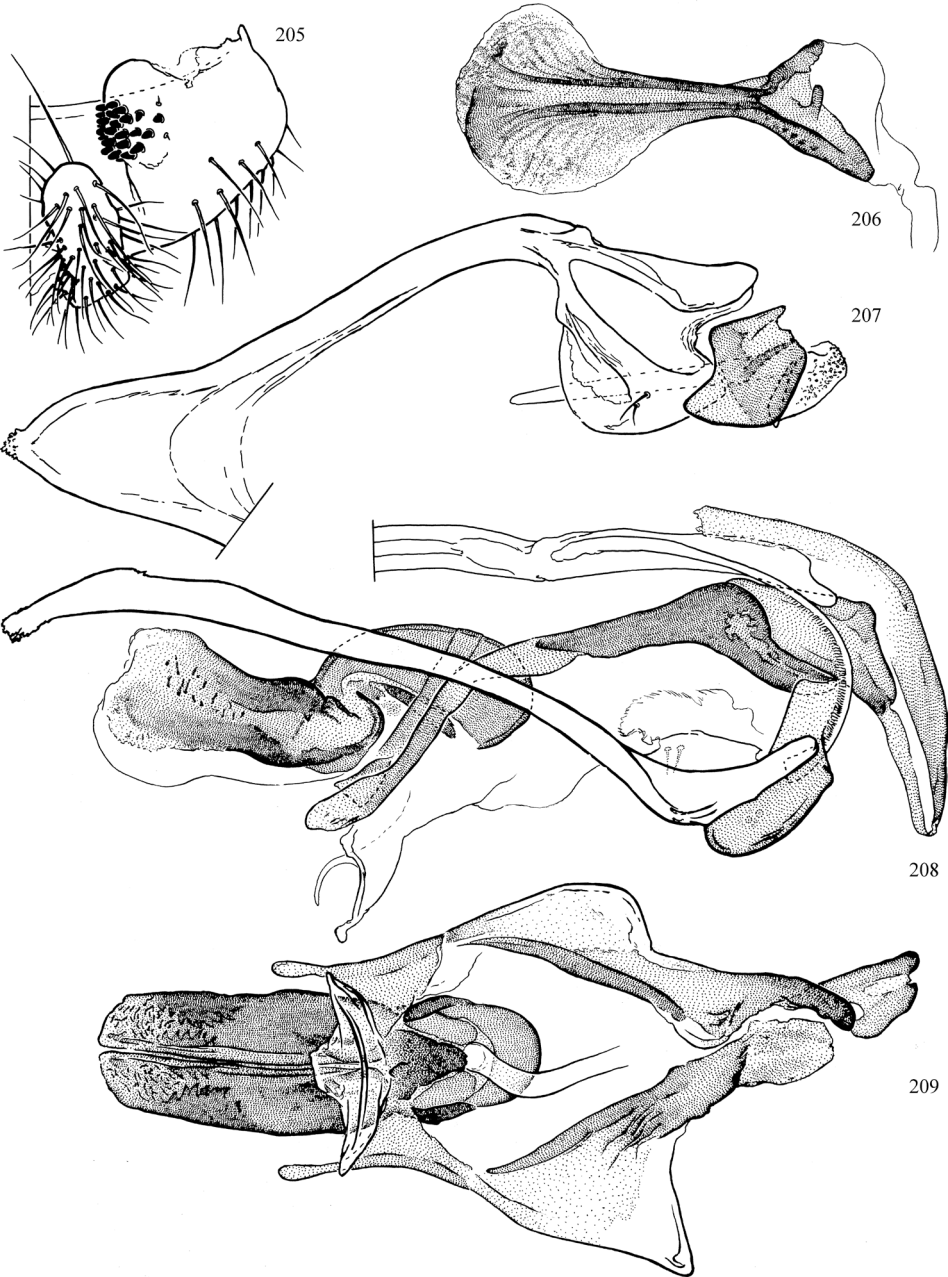
*Agromyzaparilis* Spencer, male genitalia **205** external genitalia, ventral **206** ejaculatory apodeme **207** hypandrium and postgonite, ventral **208** hypandrial complex, left lateral **209** phallus, ventral.

**Figures 210–215.**
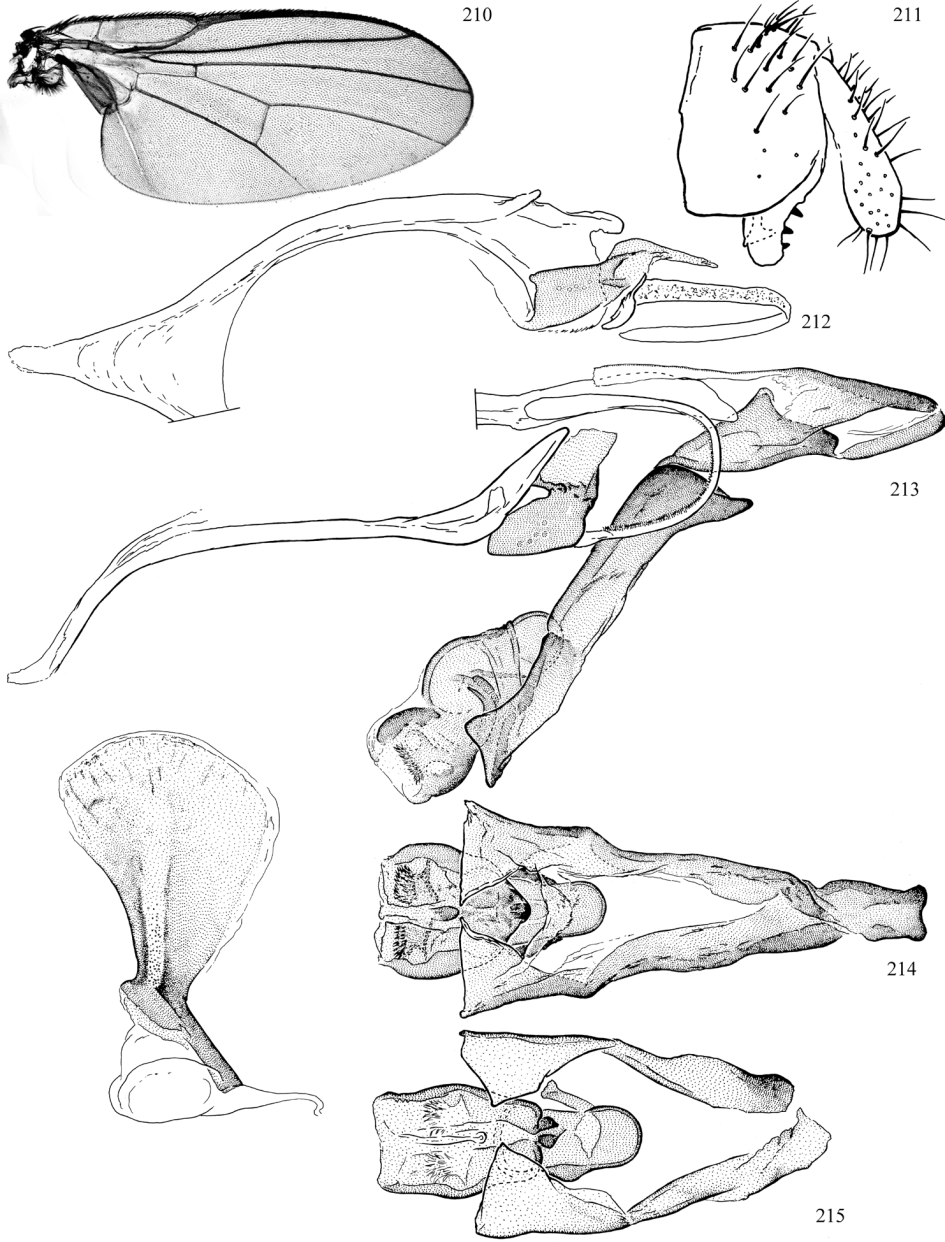
*Agromyzaparvicornis* Loew **210** wing **211** external male genitalia, left lateral **212** hypandrium and postgonite, ventral **213** hypandrial complex, left lateral **214** phallus, ventral **215***Agromyzaproxima* Spencer, male holotype phallus, ventral.

**Figures 216–220.**
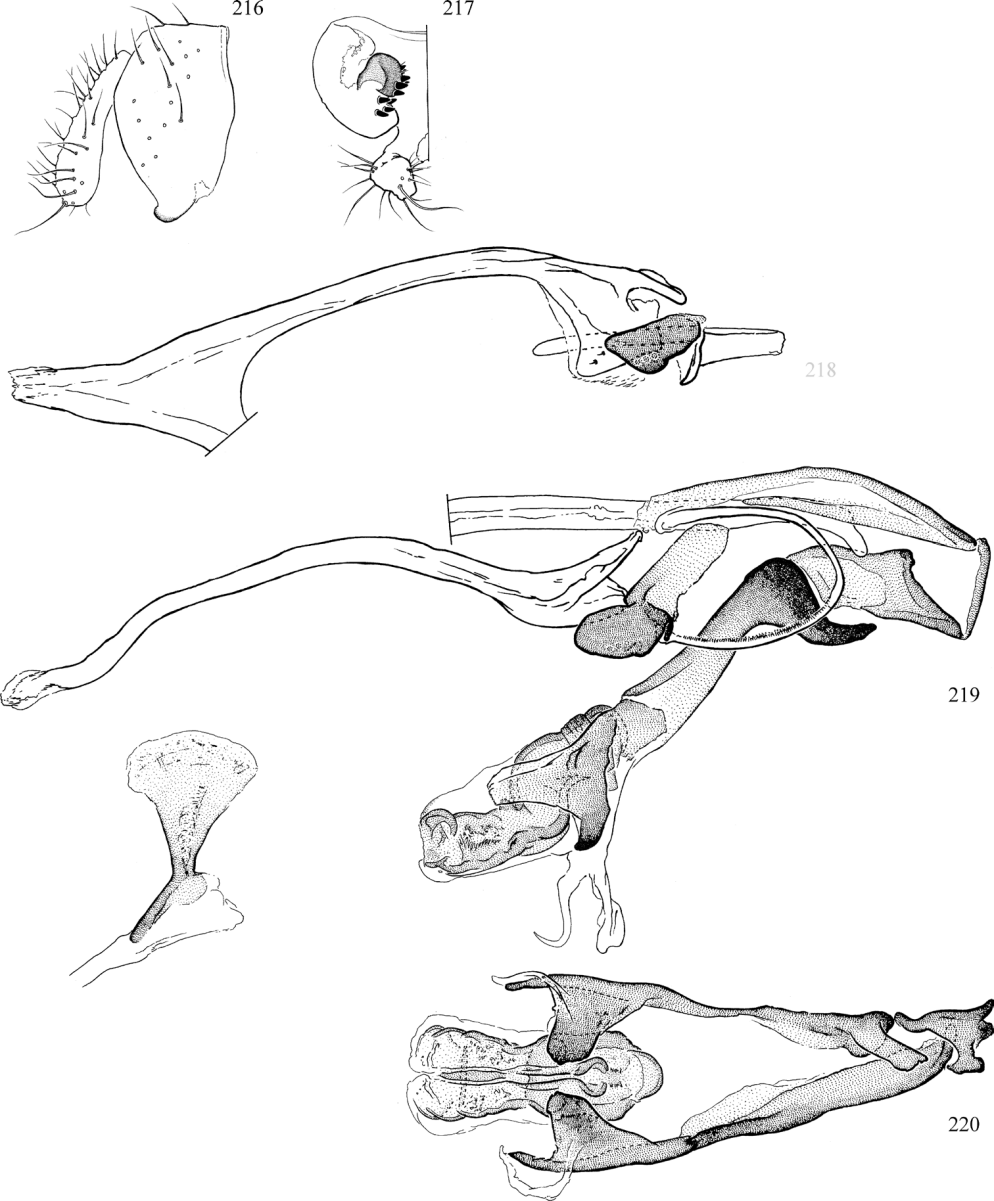
*Agromyzapudica* Spencer, male genitalia **216** external genitalia, left lateral **217** external genitalia, ventral **218** hypandrium and postgonite, ventral **219** hypandrial complex, left lateral **220** phallus, ventral.

**Figures 221–224.**
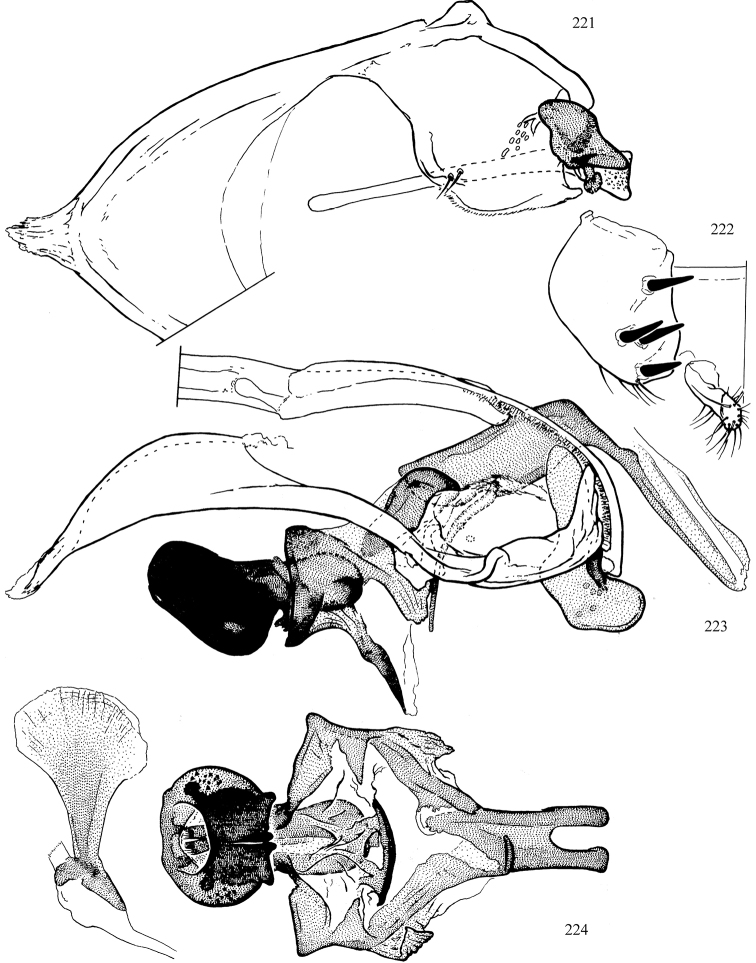
*Agromyzasoka* Eiseman & Lonsdale **221** hypandrium and postgonite, ventral **222** external genitalia, left lateral **223** hypandrial complex, left lateral **224** phallus, ventral. Originally published in Eiseman and Lonsdale (2018).

**Figures 225–235.**
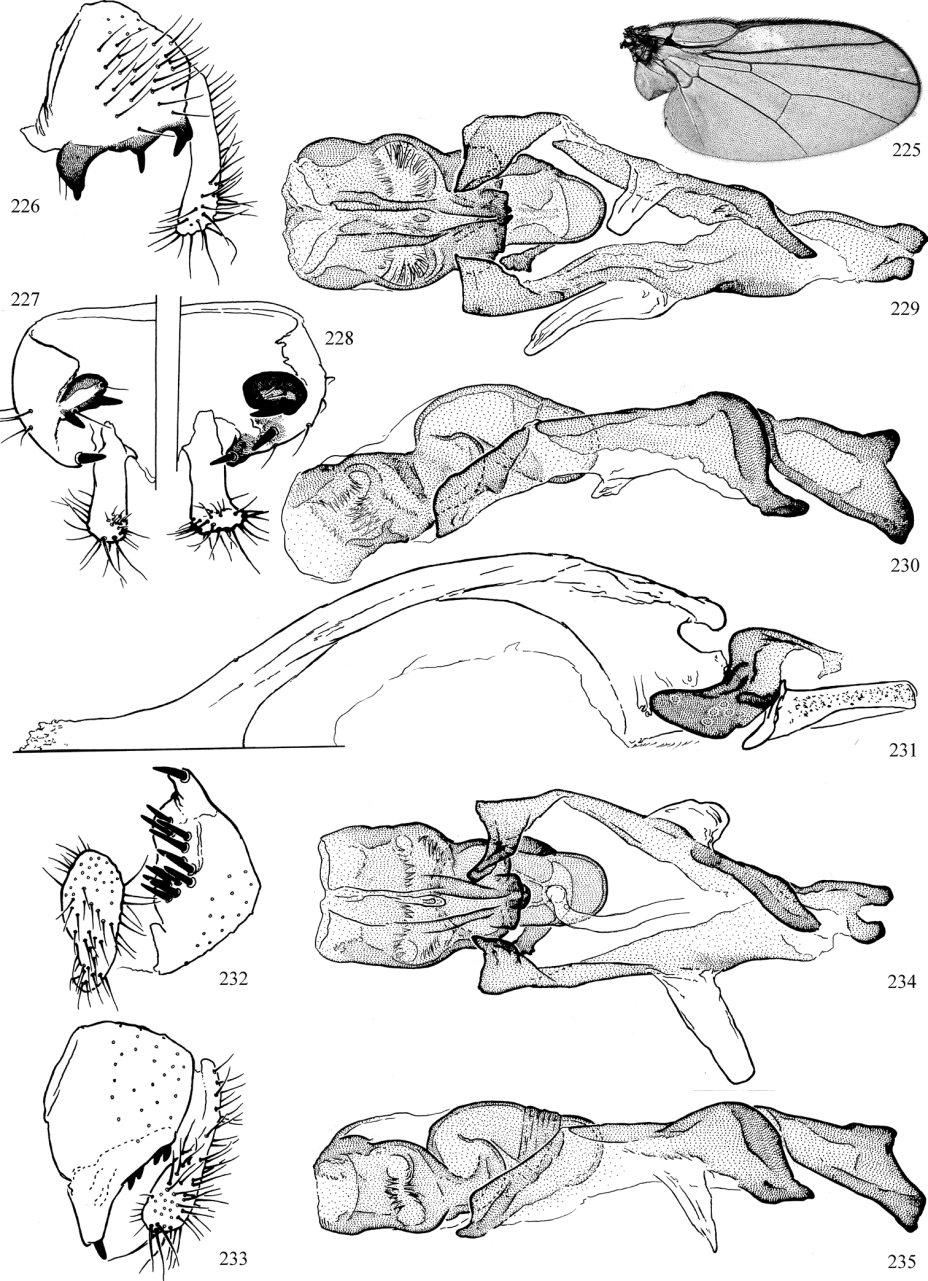
**225***Agromyzatacita* Spencer, wing **226, 227***A.tacita*, external genitalia (New Hampshire male) **226** left lateral **227** ventral **228–231***A.tacita*, genitalia (Plummers Island male) **228** external genitalia, ventral **229** phallus, ventral **230** phallus, left lateral **231** hypandrium and postgonite, ventral **232–235***A.echinalis* Lonsdale, sp. nov., male holotype genitalia **232** external genitalia, ventral **233** external genitalia, left lateral **234** phallus, ventral **235** phallus, left lateral.

**Figures 236–242.**
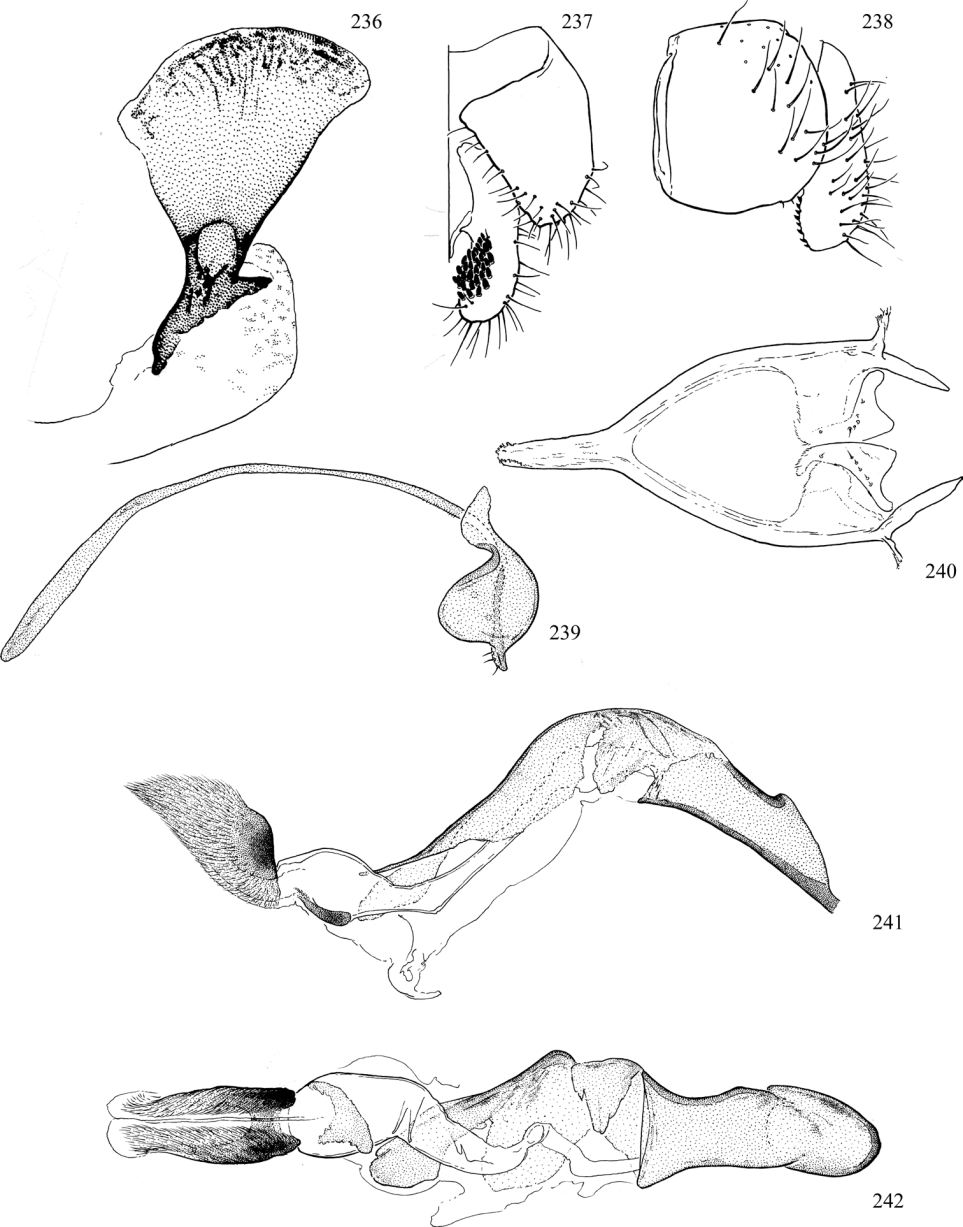
*Agromyzavarifrons* Coquillett, male genitalia **236** ejaculatory apodeme **237** external genitalia, ventral **238** external genitalia, left lateral **239** postgonite, lateral (1/2 size) **240** hypandrium, ventral (1/2 size) **241** phallus, left lateral **242** phallus, ventral.

**Figures. 243–249.**
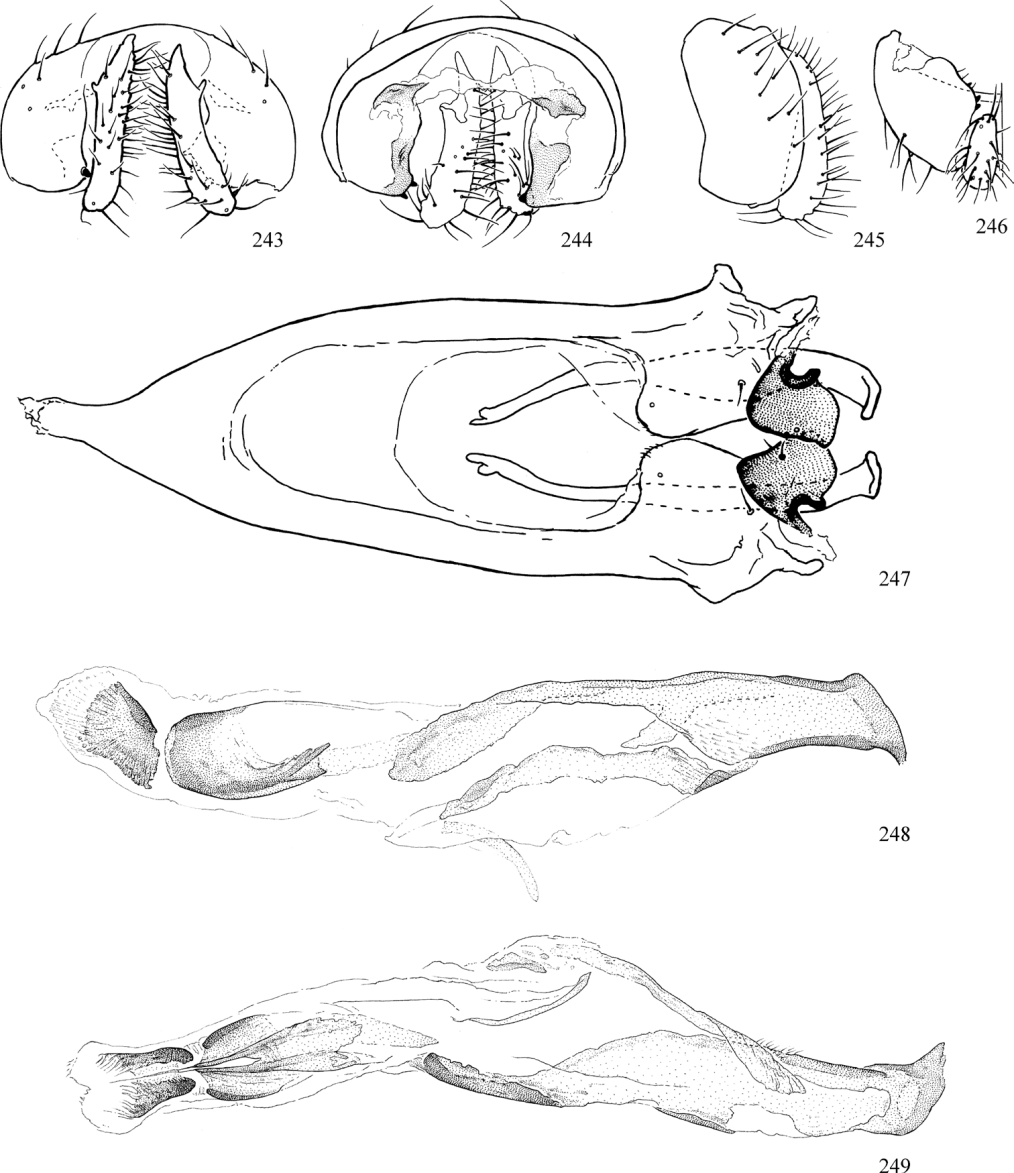
*Agromyzavockerothi* Spencer, male genitalia **243** external genitalia, posterior **244** external genitalia, anterior **245** external genitalia, left lateral **246** external genitalia, ventral **247** hypandrium and postgonite, ventral **248** phallus, left lateral **249** phallus, ventral.

**Figure 250.**
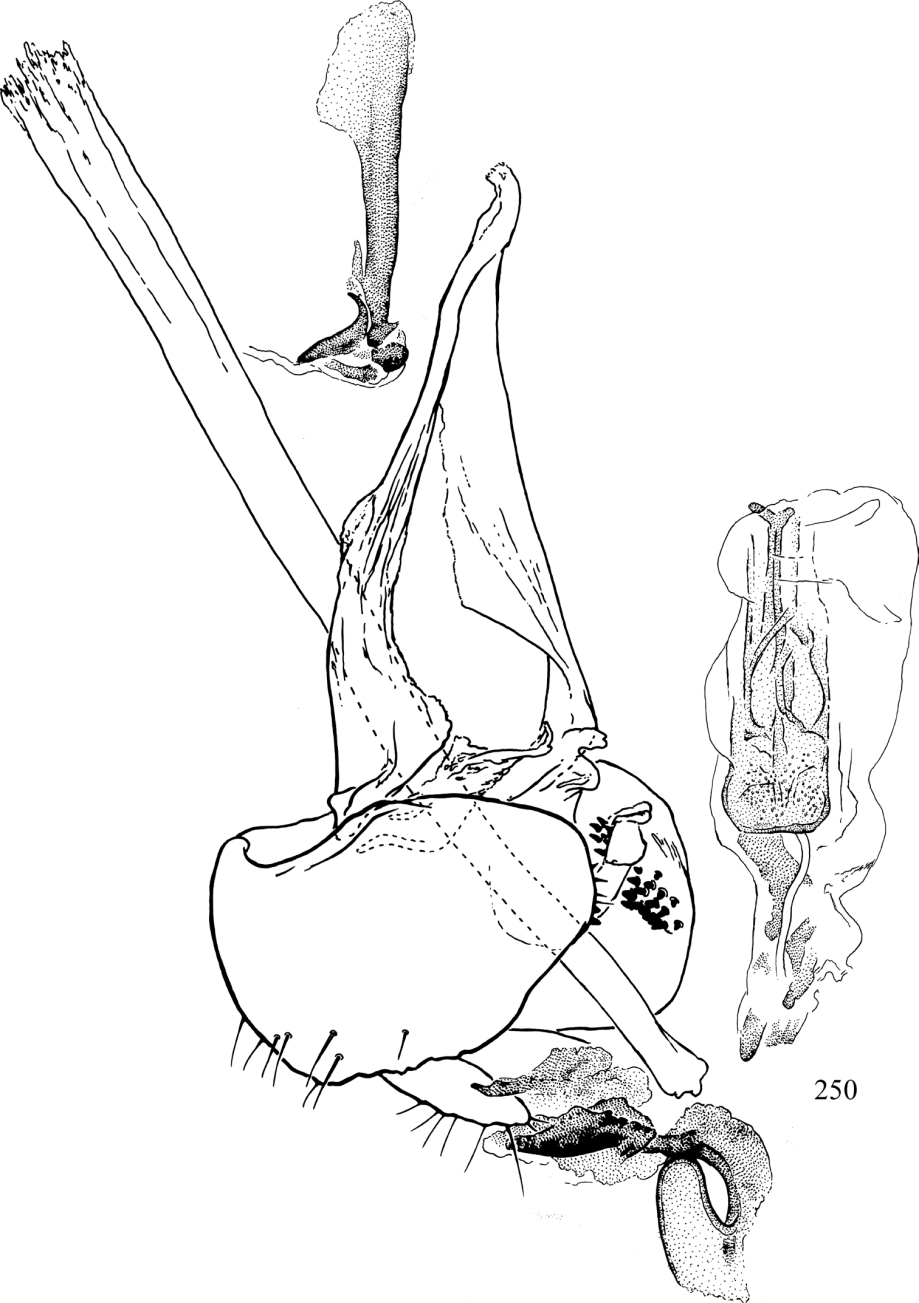
*Euhexomyzaalbicula* (Spencer), male holotype genitalia, with phallus (broken off) inset at right, ventral view.

**Figures 251–256.**
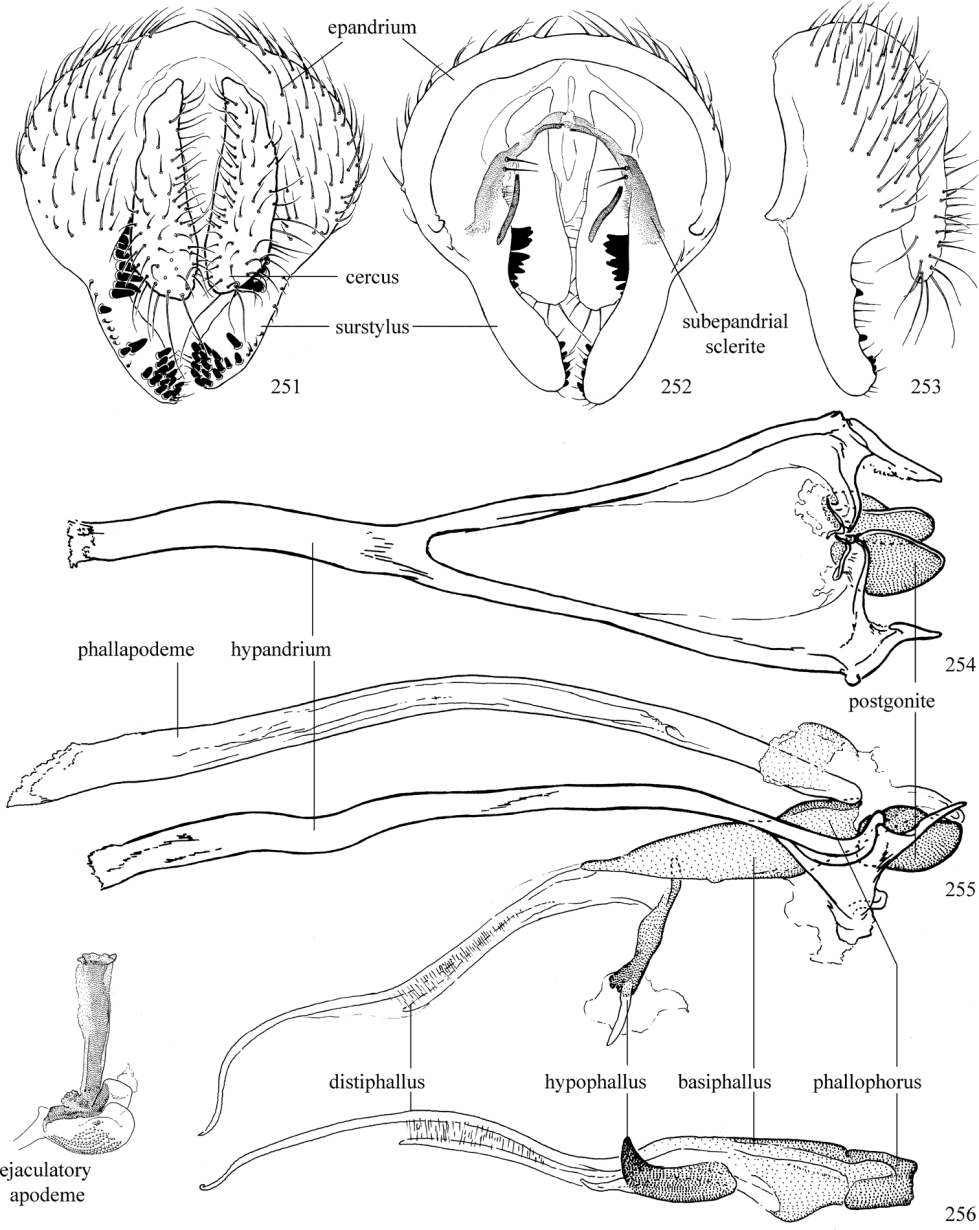
*Japanagromyzaviridula* (Coquillett), male genitalia **251** external genitalia, posterior **252** external genitalia, anterior **253** external genitalia, left lateral **254** hypandrium and postgonite, ventral **255** hypandrial complex, left lateral **256** phallus, ventral.

**Figures 257–260.**
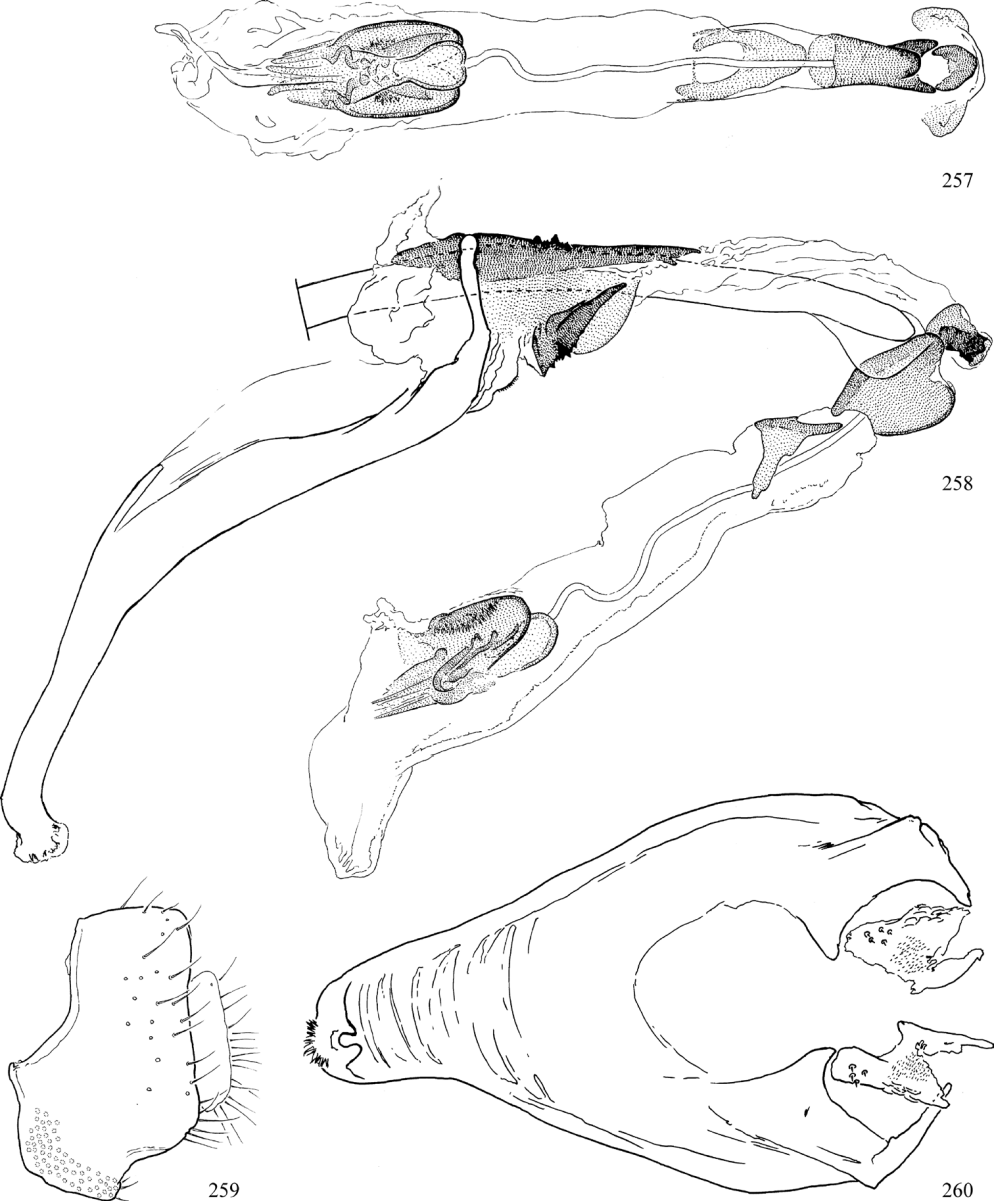
*Melanagromyzaangelicae* (Frost), male genitalia **257** phallus and proepiphallus, ventral **258** hypandrial complex, left lateral **259** external components, left lateral **260** hypandrium, ventral.

**Figures 261–263.**
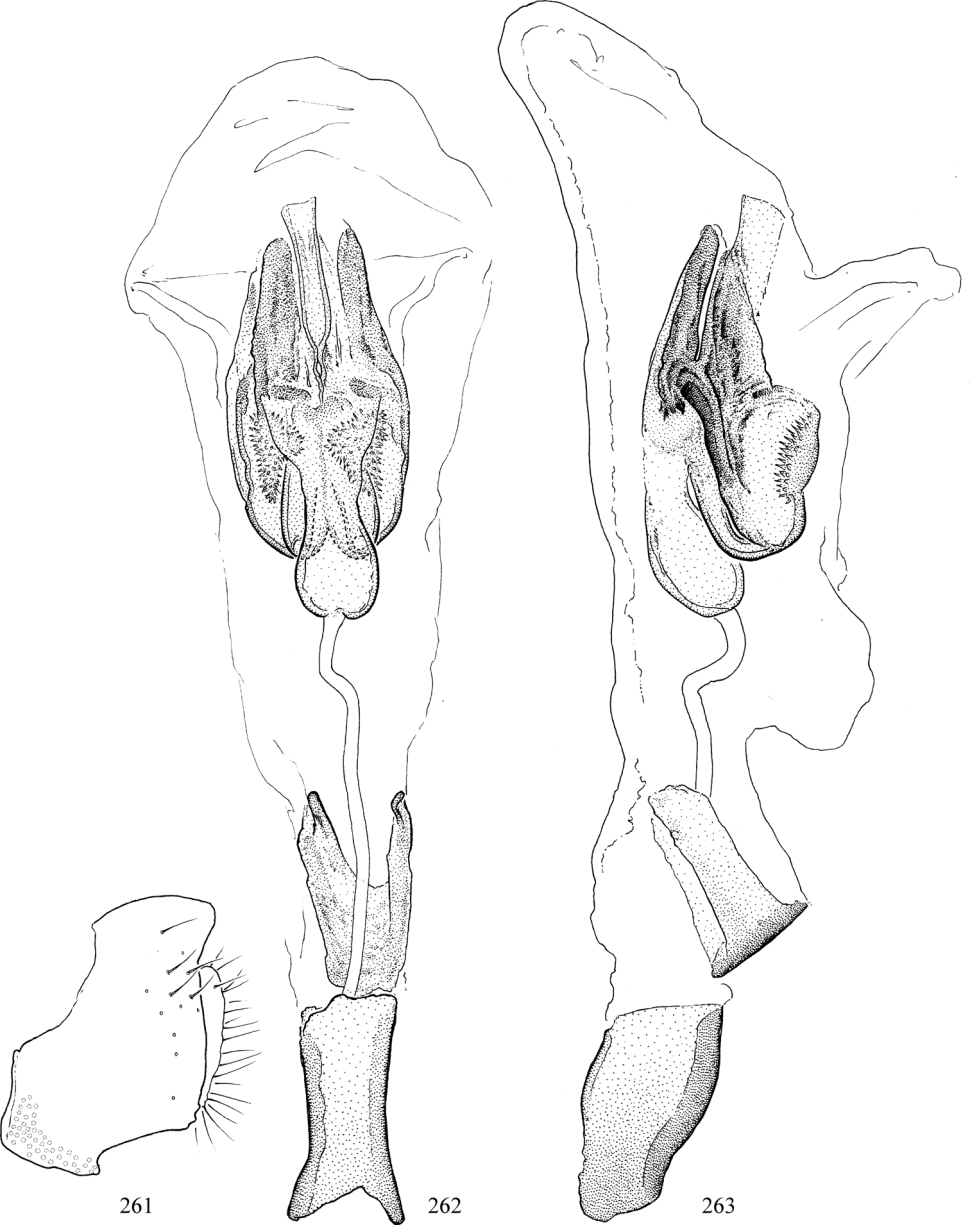
*Melanagromyzabrunkei* sp. nov., male genitalia **261** external components, left lateral **262** phallus, ventral **263** phallus, left lateral.

**Figures 264–268.**
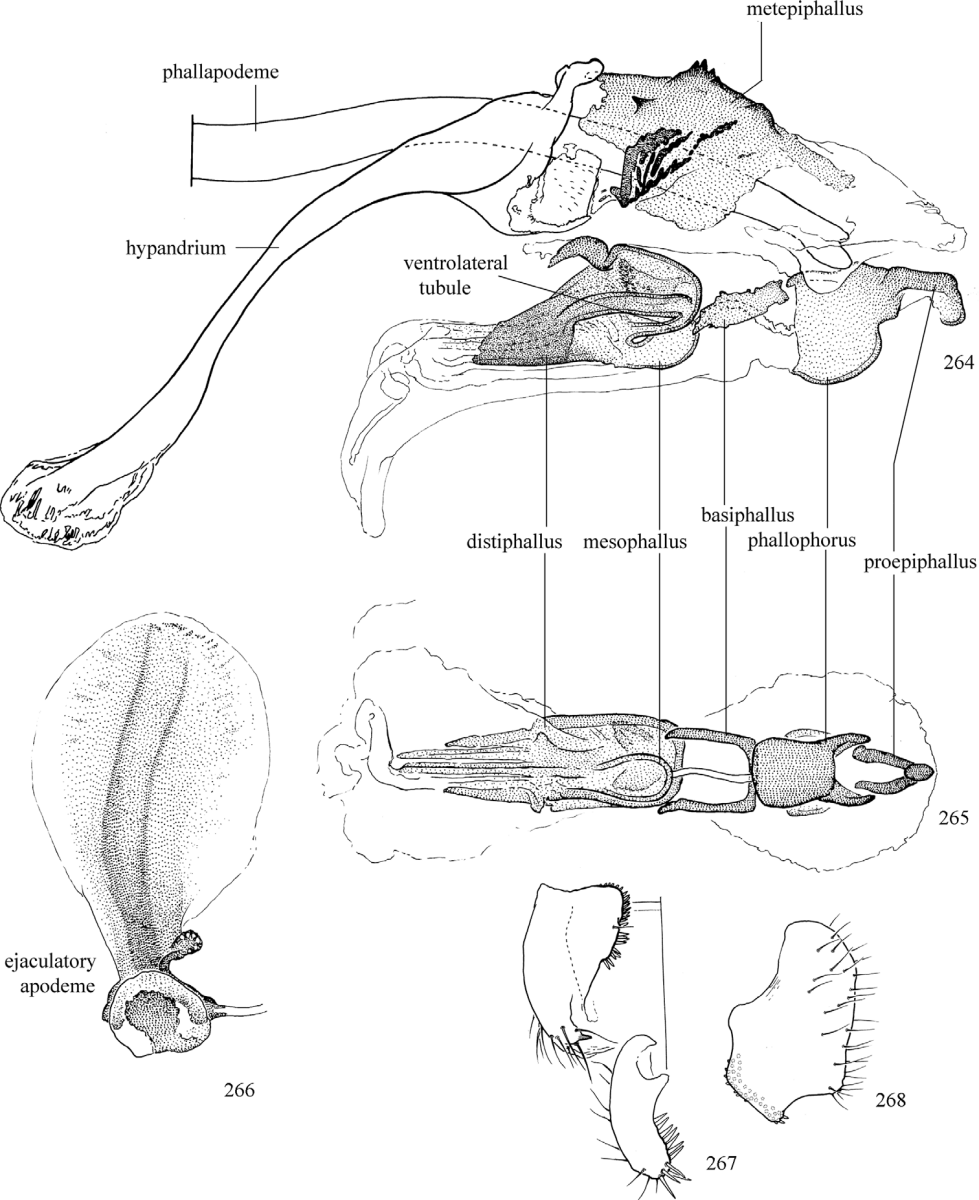
*Melanagromyzabuccalis* Spencer, male genitalia **264** hypandrial complex, left lateral **265** phallus and proepiphallus, ventral **266** ejaculatory apodeme **267** external components, ventral **268** external components, left lateral.

**Figures 269–271.**
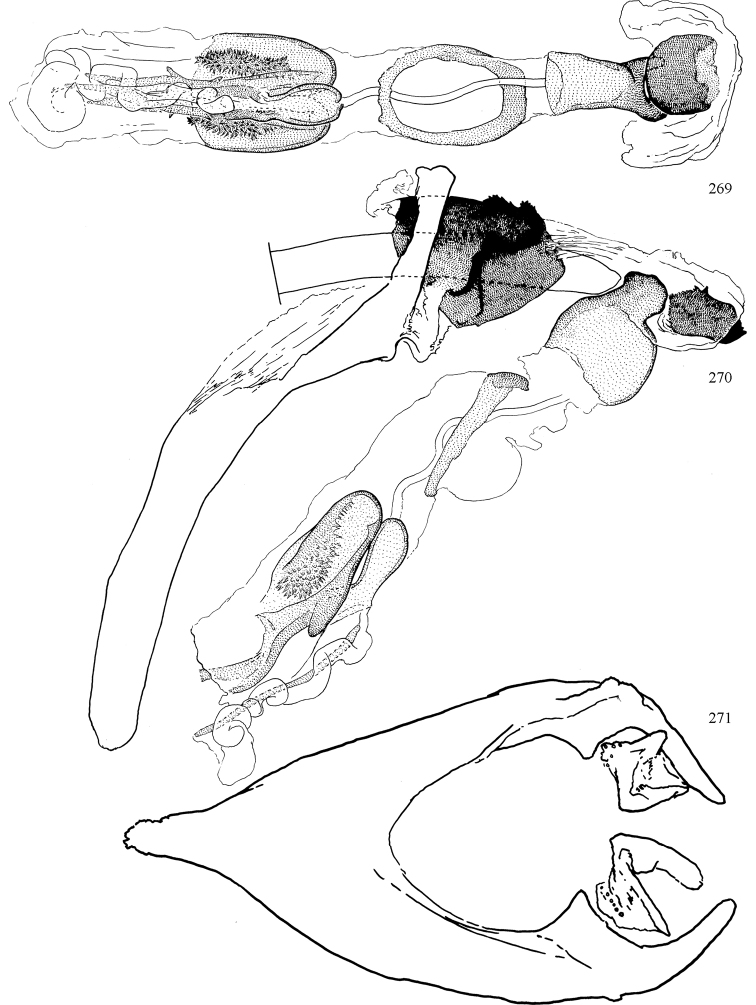
*Melanagromyzaburgessi* (Malloch), male genitalia **269** phallus and proepiphallus, ventral **270** hypandrial complex, left lateral **271** hypandrium, ventral.

**Figures 272–276.**
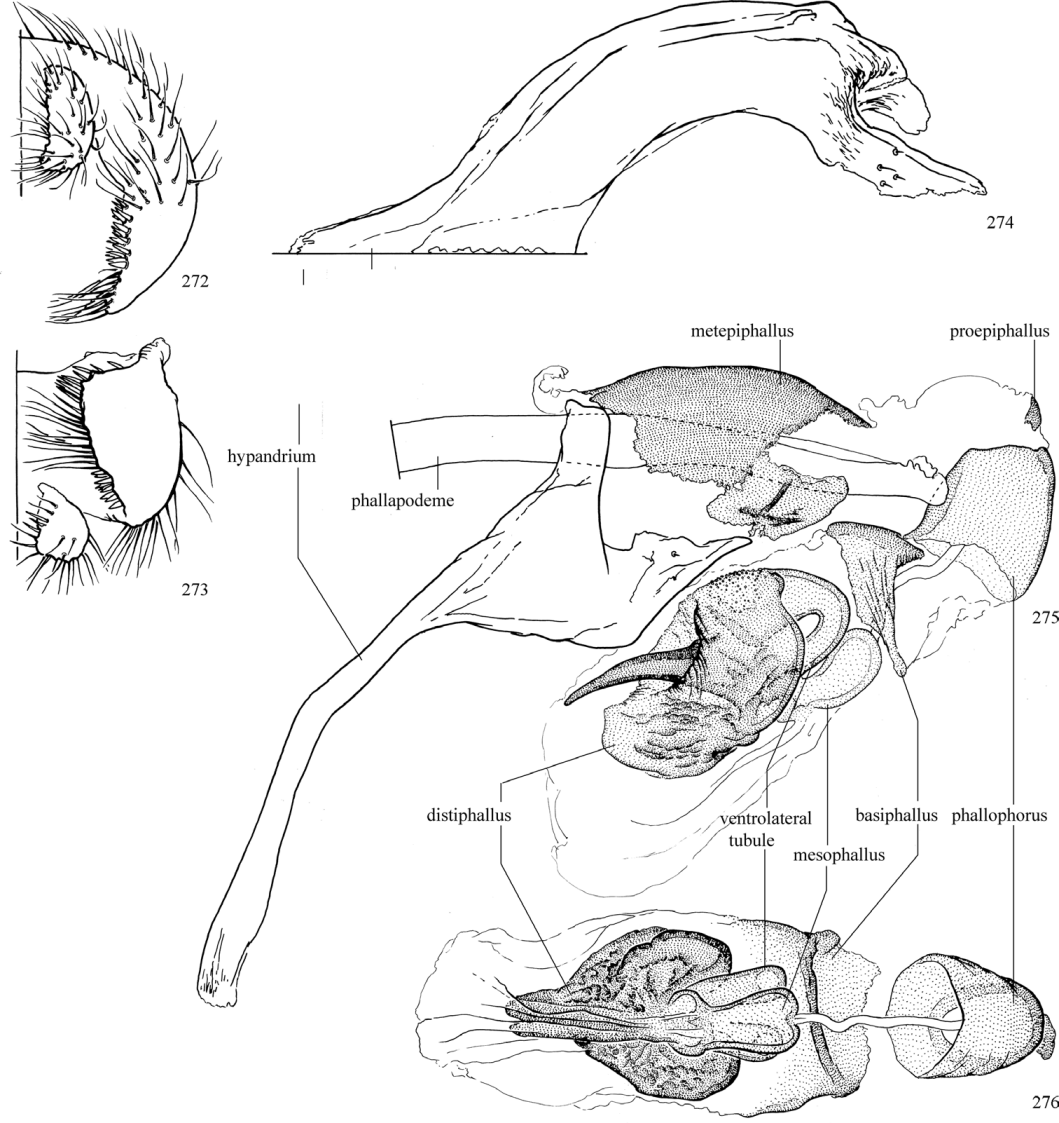
*Melanagromyzadianthereae* (Malloch), male genitalia **272** external components, posterior **273** external components, ventral **274** hypandrium, ventral **275** hypandrial complex, left lateral **276** phallus and proepiphallus, ventral.

**Figures 277–280.**
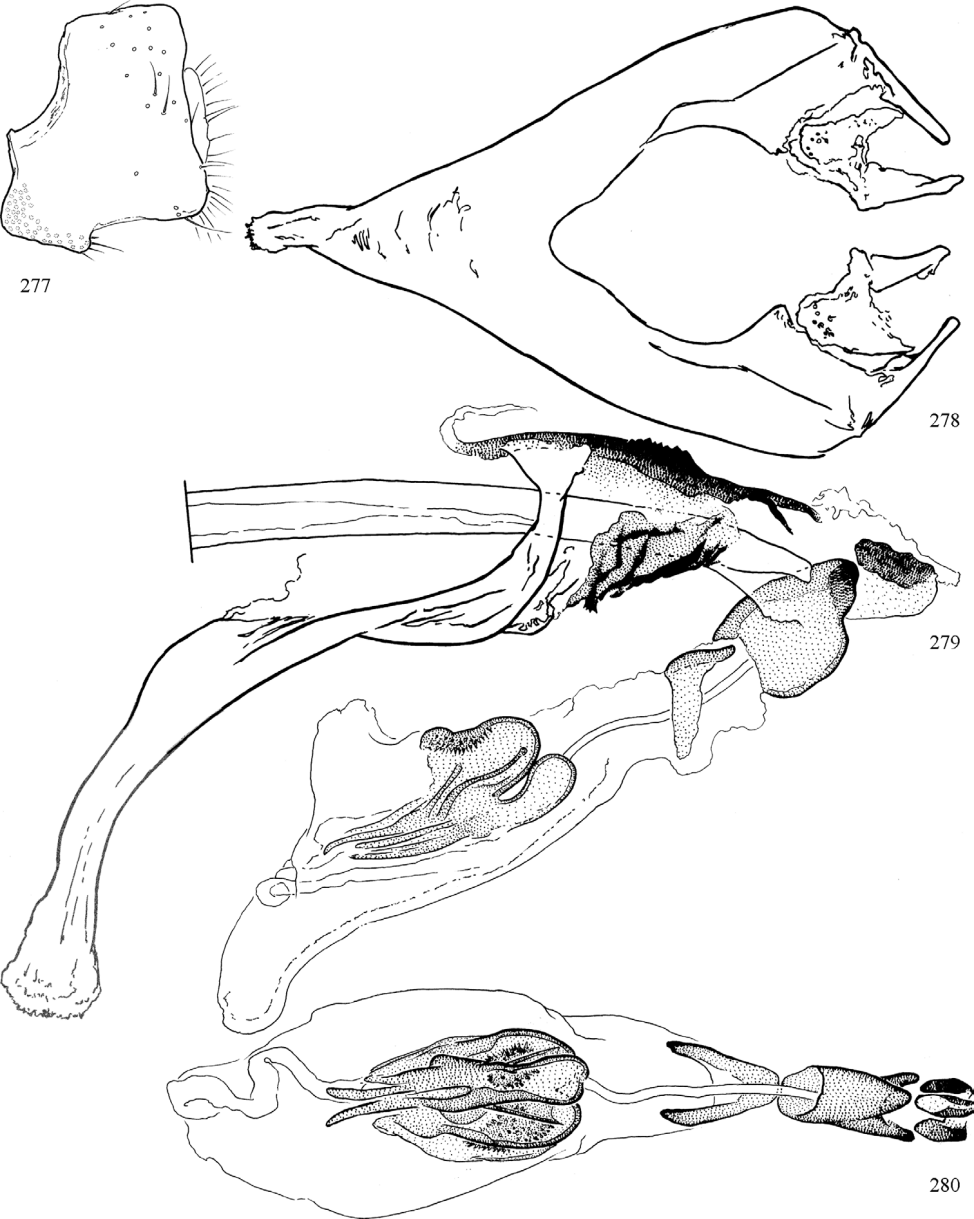
*Melanagromyzaeoflacensis* sp. nov., male genitalia **277** external components, left lateral **278** hypandrium, ventral **279** hypandrial complex, left lateral **280** phallus and proepiphallus, ventral.

**Figures 281–284.**
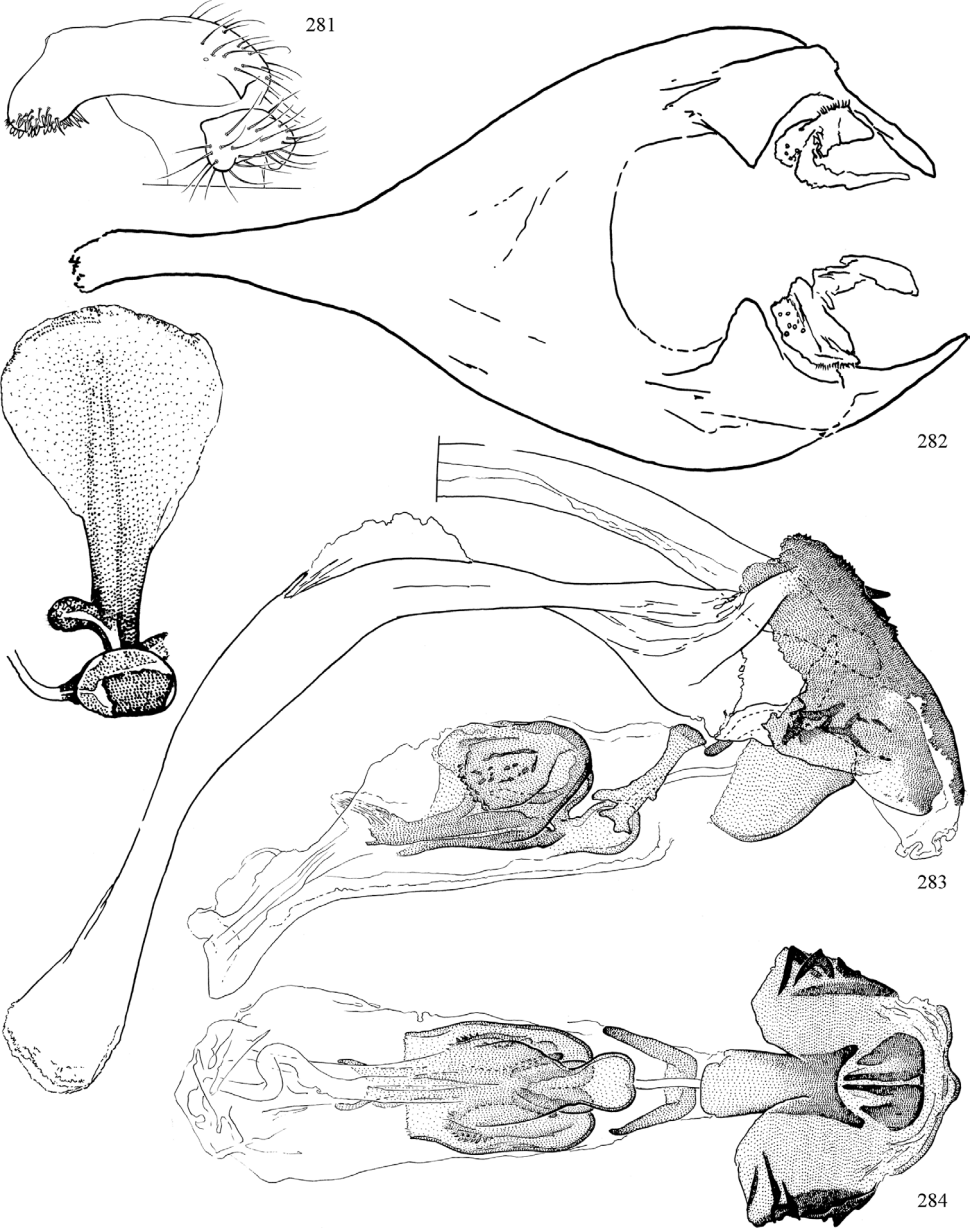
*Melanagromyzaglyptos* Lonsdale, sp. nov., male genitalia **281** external components, ventral **282** hypandrium, ventral **283** hypandrial complex, left lateral **284** phallus and epiphallus, ventral.

**Figures 285–288.**
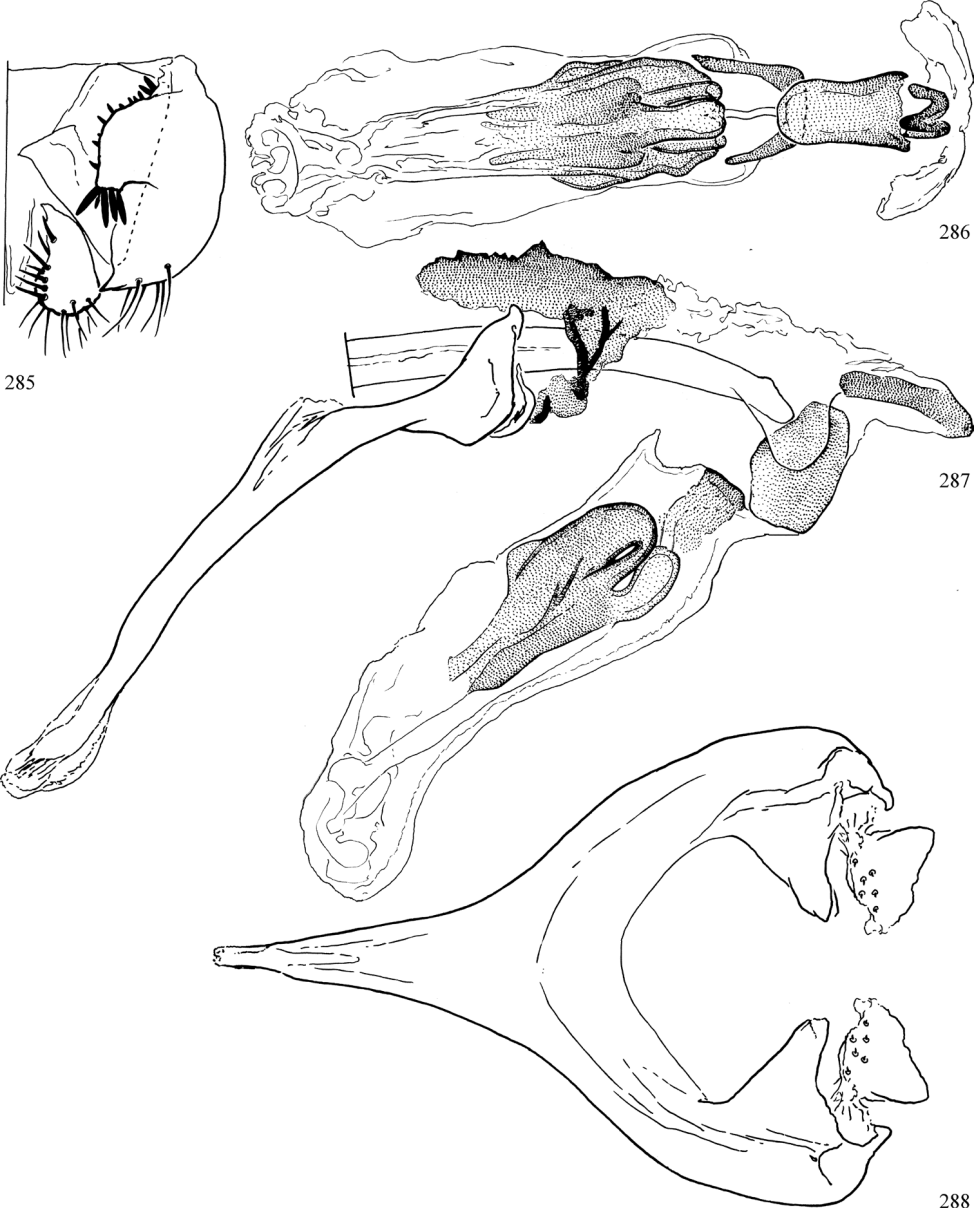
*Melanagromyzamatricariodes* Spencer, male genitalia **285** external genitalia (including subepandrial sclerite), ventral **286** phallus and proepiphallus, ventral **287** hypandrial complex, left lateral **288** hypandrium, ventral.

**Figures 289–292.**
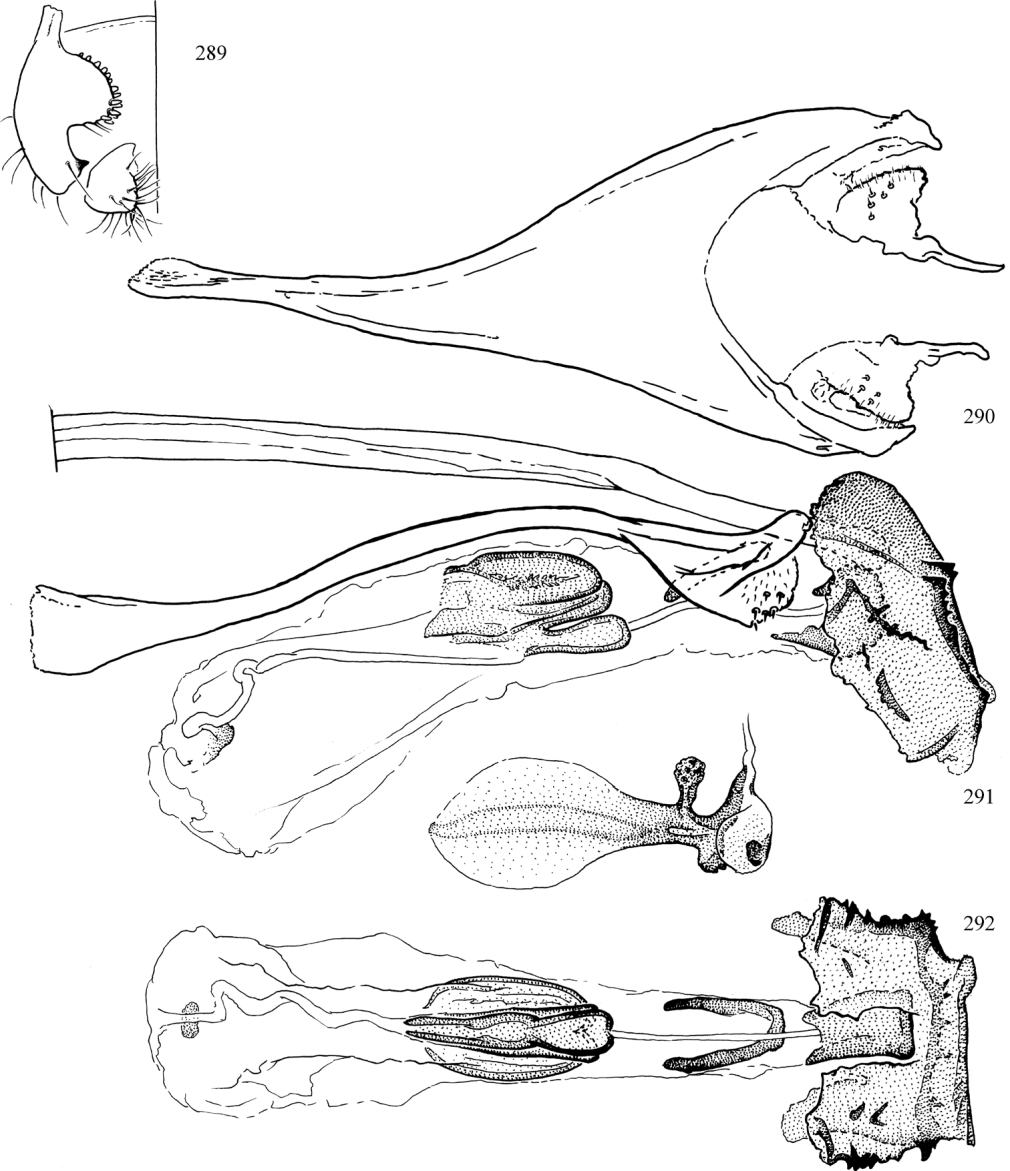
*Melanagromyzaminimoides* Spencer, male genitalia **289** external components, ventral **290** hypandrium, ventral **291** hypandrial complex, left lateral **292** phallus and epiphallus, ventral.

**Figures 293–296.**
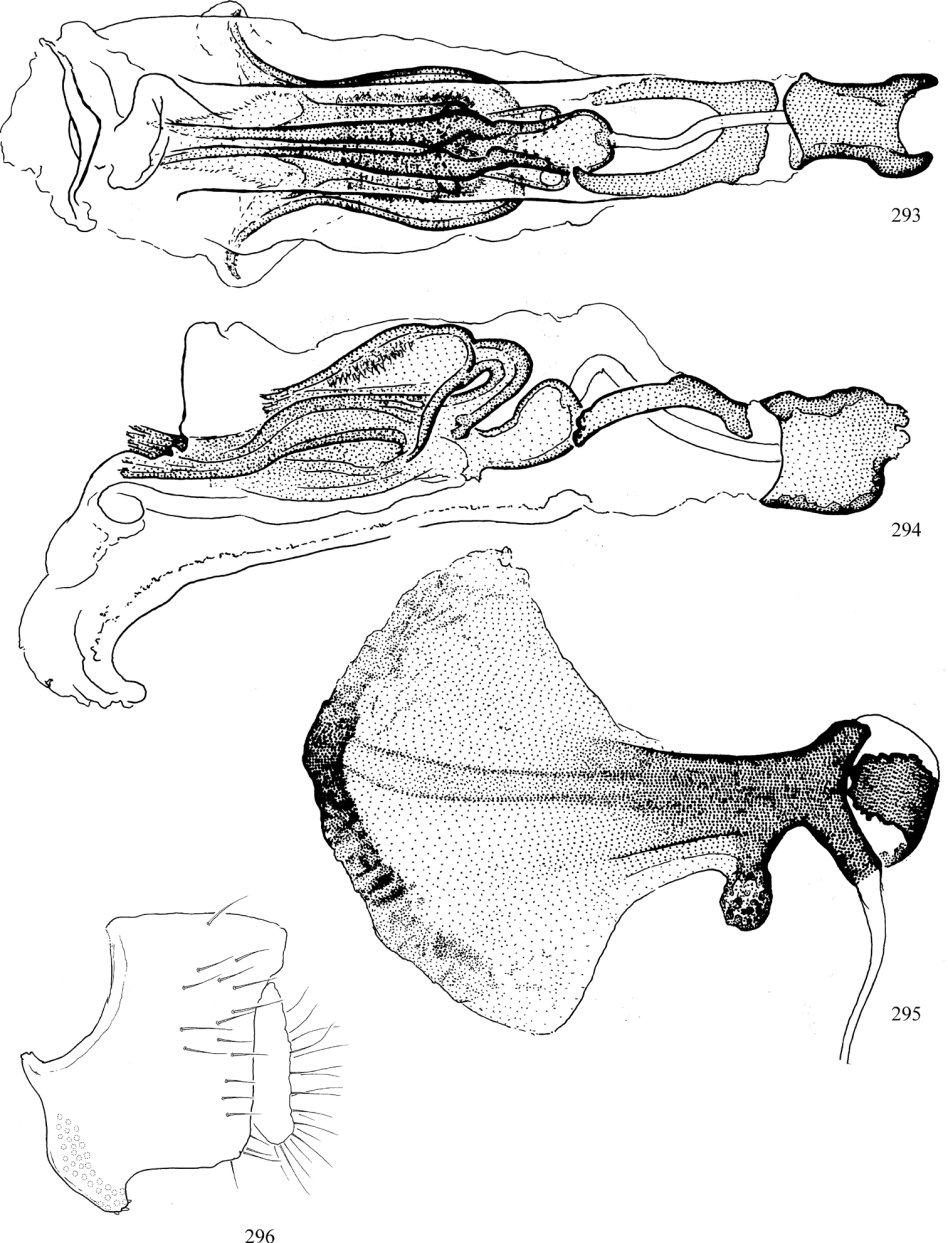
*Melanagromyzarutella* Lonsdale, sp. nov. **293** phallus, ventral **294** phallus, left lateral **295** ejaculatory apodeme **296** external components, left lateral.

**Figures 297–300.**
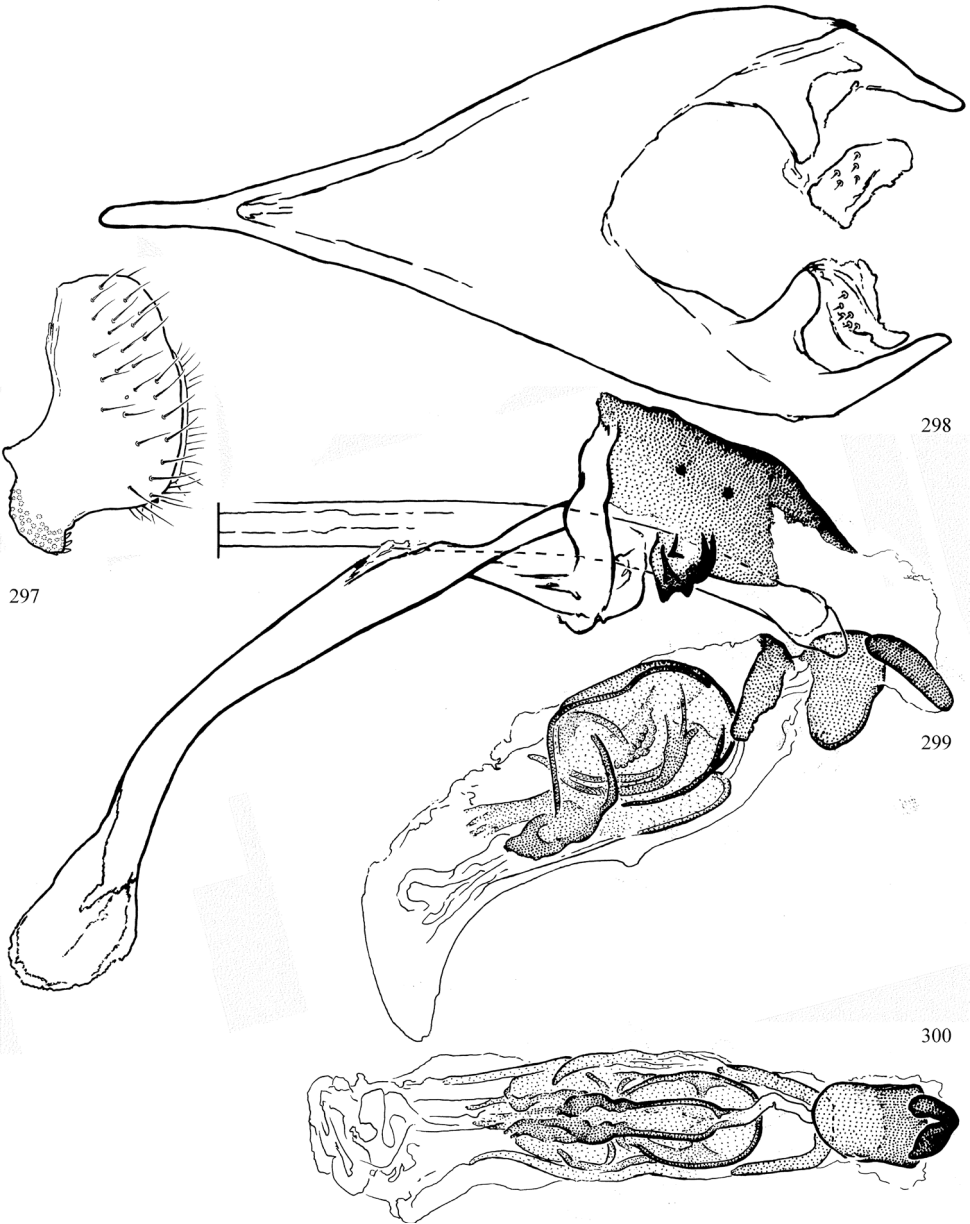
*Melanagromyzasplendida* Frick, male genitalia **297** external components, left lateral **298** hypandrium, ventral **299** hypandrial complex, left lateral **300** phallus and proepiphallus, ventral.

**Figures 301–304.**
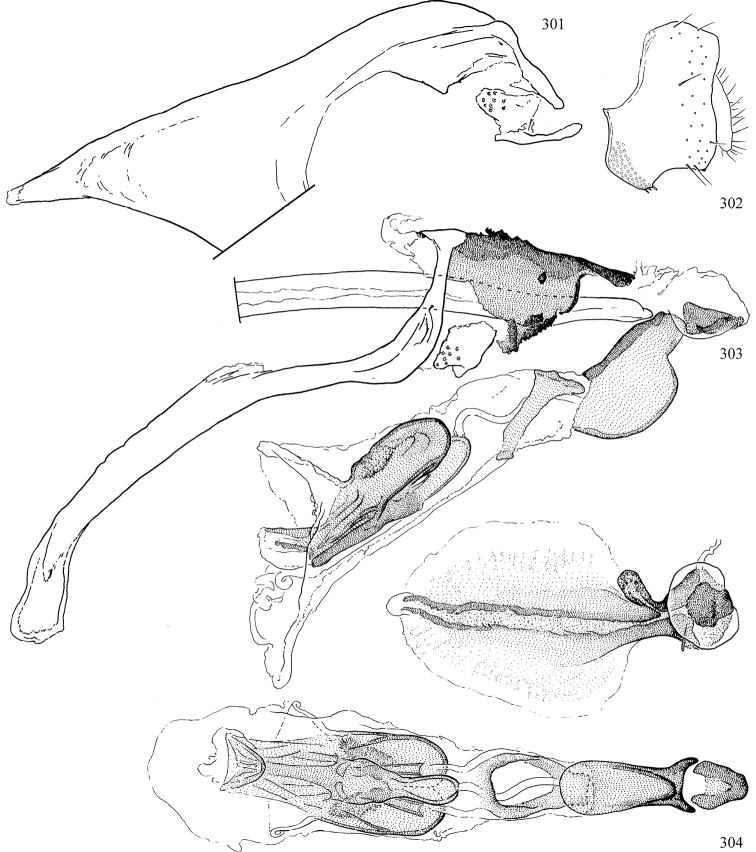
*Melanagromyzasubvirens* (Malloch), male genitalia **301** hypandrium, ventral **302** external components, left lateral **303** hypandrial complex, left lateral **304** phallus and proepiphallus, ventral.

**Figures 305–308.**
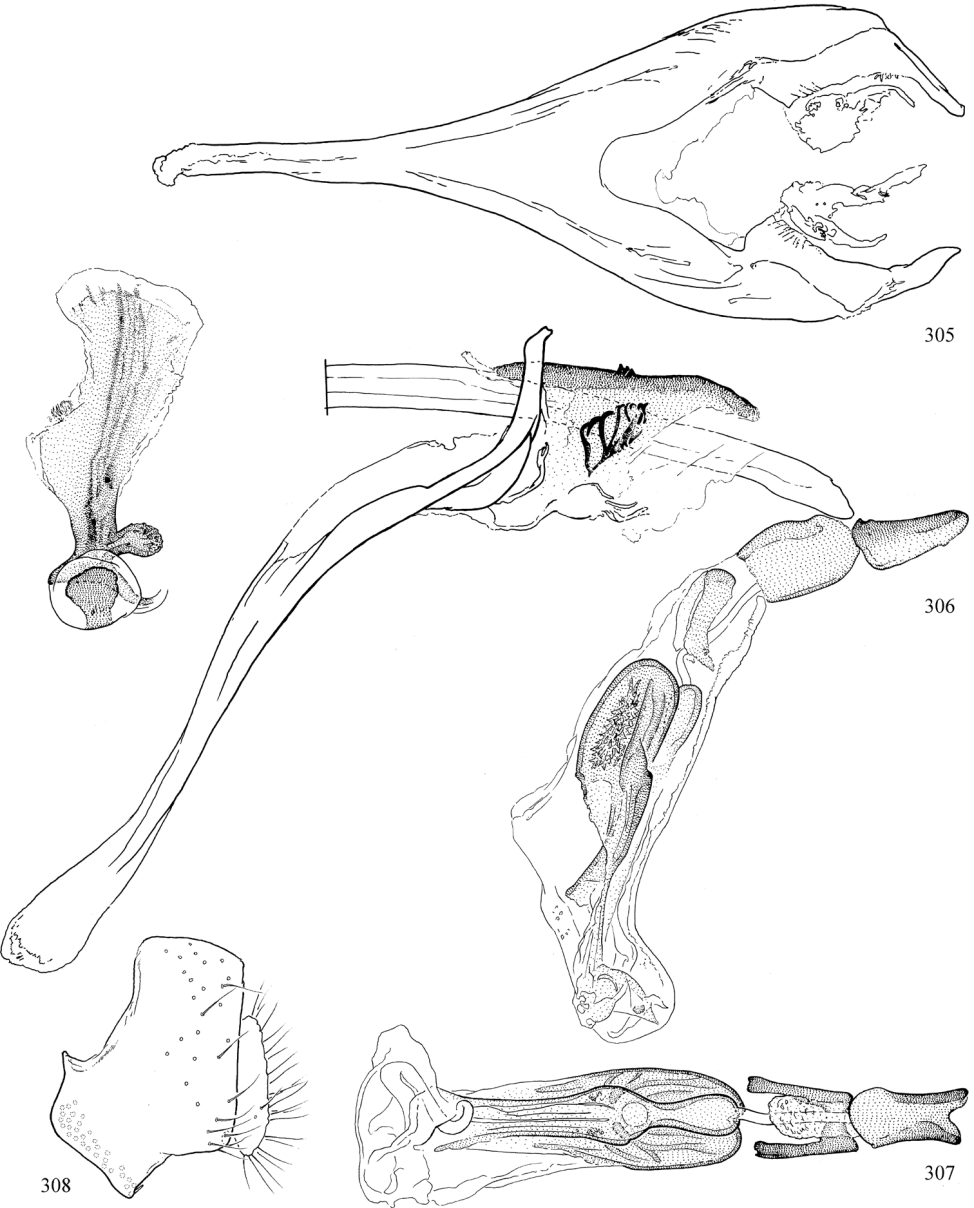
*Melanagromyzavernoniae* Steyskal, male genitalia **305** hypandrium, ventral **306** hypandrial complex, left lateral **307** phallus and proepiphallus, ventral **308** external components, left lateral.

**Figures 309–317.**
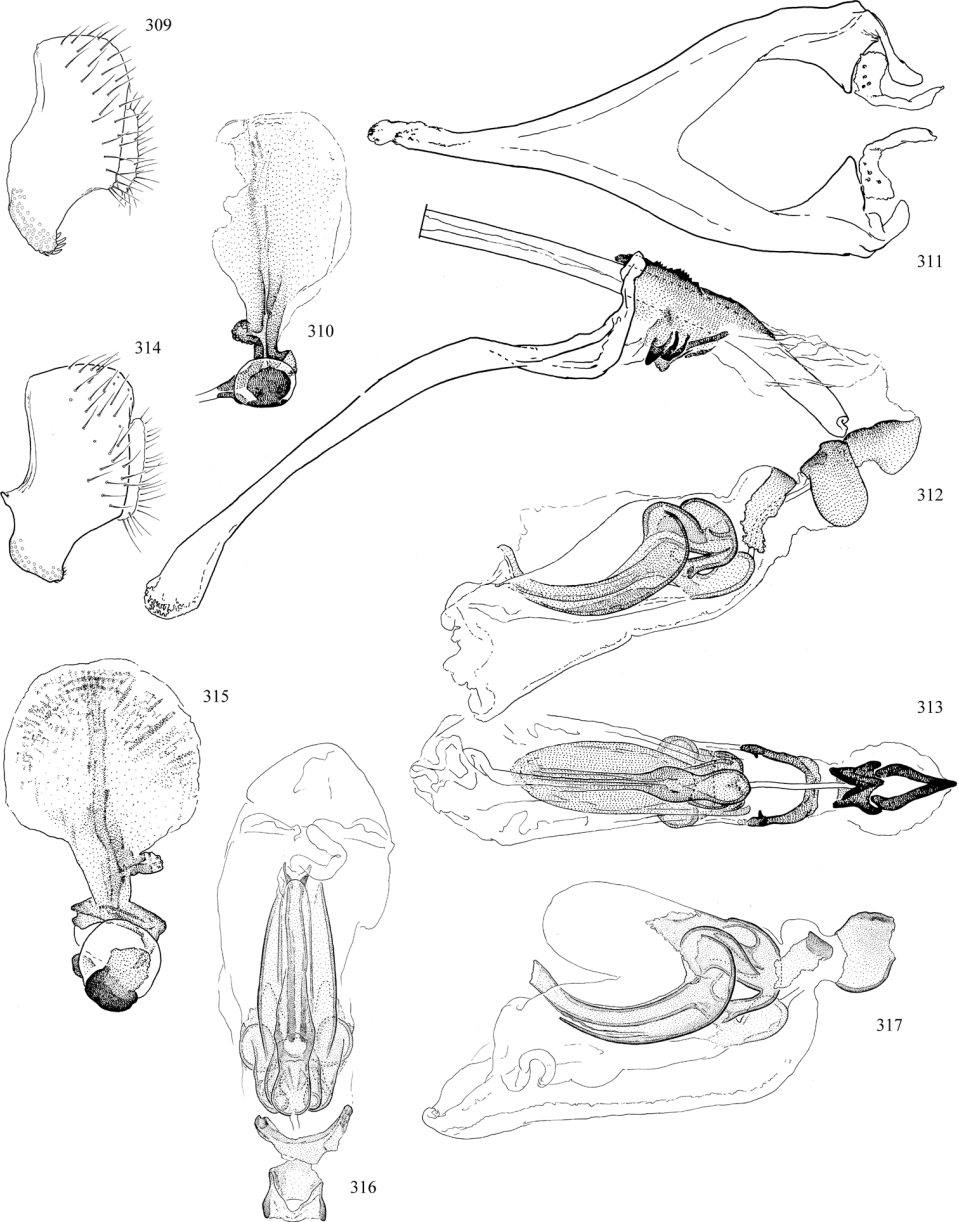
*Melanagromyzavernoniana* Steyskal, male genitalia **309** external components, left lateral (non-type with elongate, shallow surstylus as seen in type series) **310** ejaculatory apodeme, paratype **311** hypandrium, ventral, paratype **312** hypandrial complex, left lateral, paratype **313** phallus and proepiphallus, ventral, paratype **314–317***Melanagromyzavernoniana*, male genitalia, non-type ex “artichoke” **314** external components, left lateral (note shorter, truncated surstylus) **315** ejaculatory apodeme **316** phallus, ventral **317** phallus, left lateral.

**Figures 318–324.**
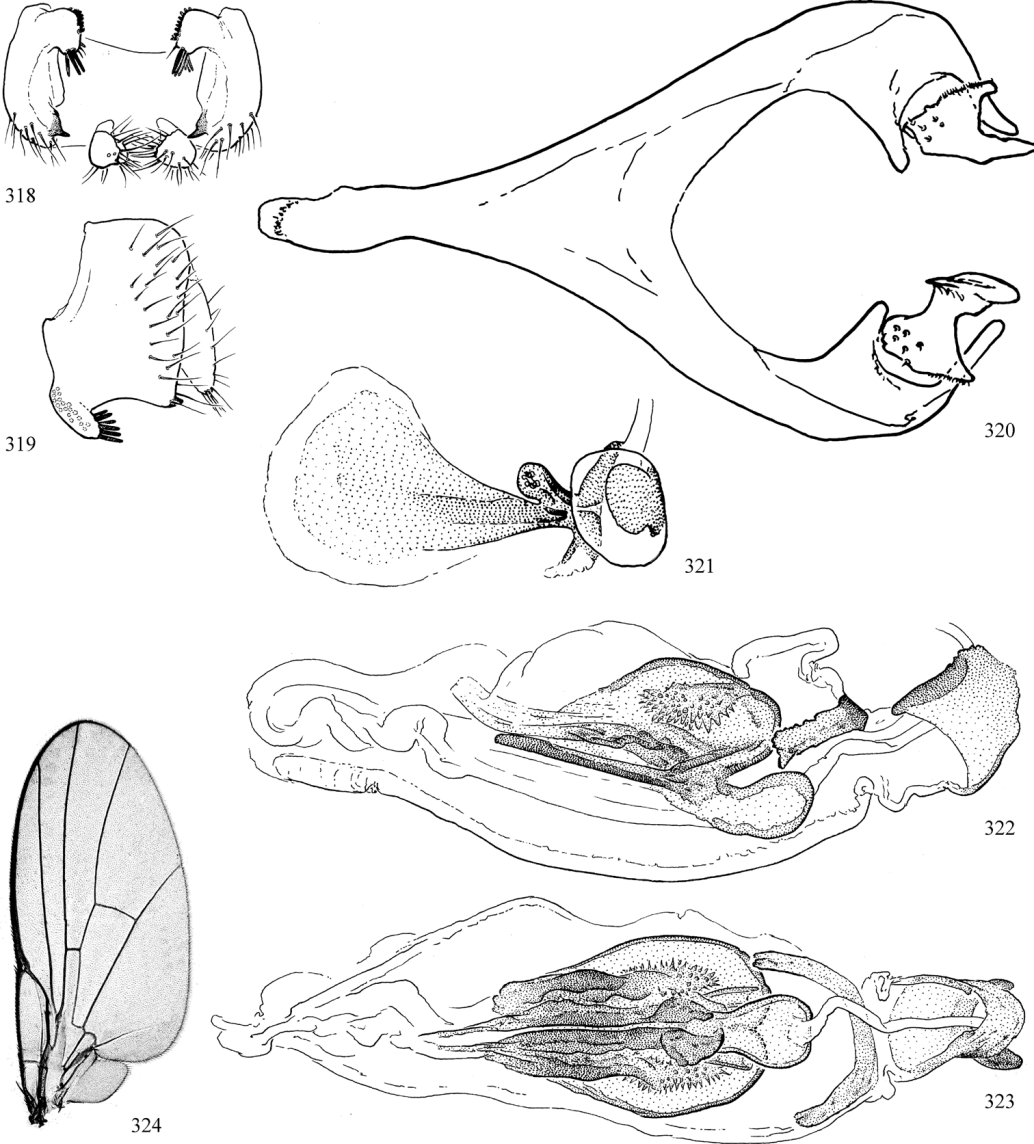
*Melanagromyzavirens* (Loew), male genitalia **318** external components, ventral **319** external components, left lateral **320** hypandrium, ventral **321** ejaculatory apodeme **322** phallus, left lateral **323** phallus, ventral **324***Melanagromyzavirens*, wing.

**Figures 325–328.**
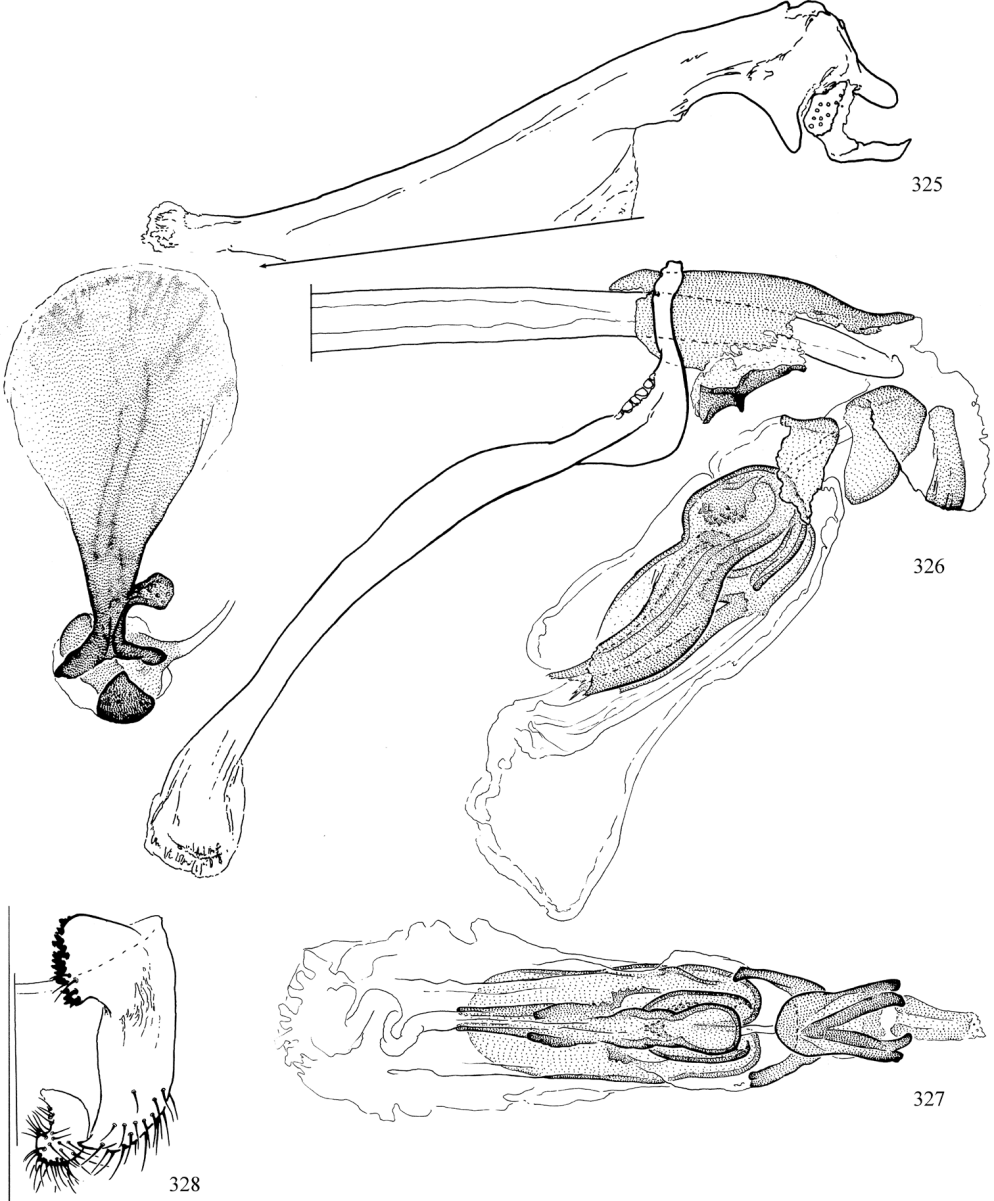
*Melanagromyzavirginiensis* Spencer, male genitalia **325** hypandrium, ventral **326** hypandrial complex, left lateral **327** phallus and epiphallus, ventral **328** external genitalia, ventral.

**Figures 329–331.**
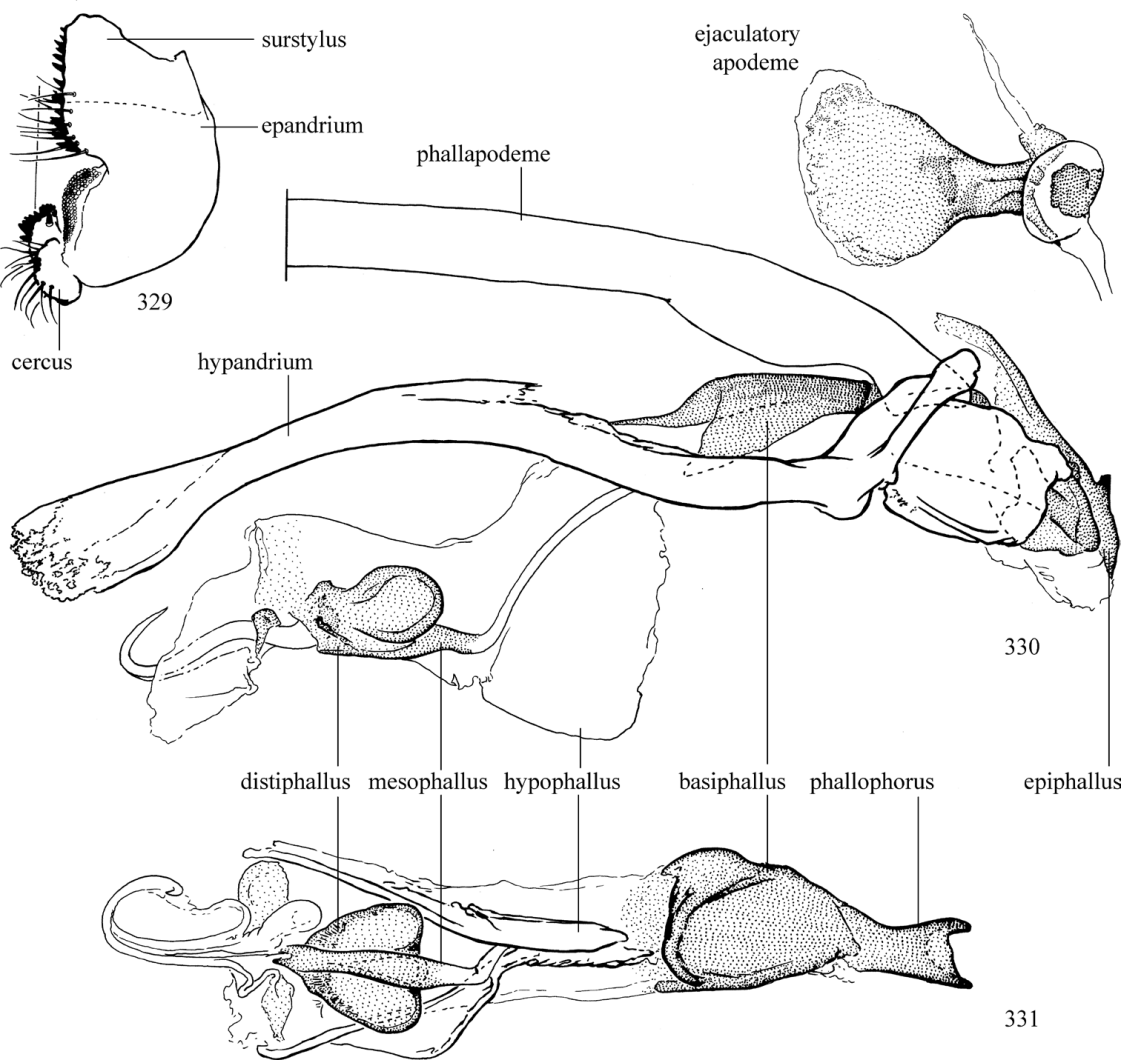
*Ophiomyiaabutilivora* Spencer, male genitalia **329** external genitalia, ventral **330** hypandrial complex, left lateral **331** phallus, ventral.

**Figures 332–336.**
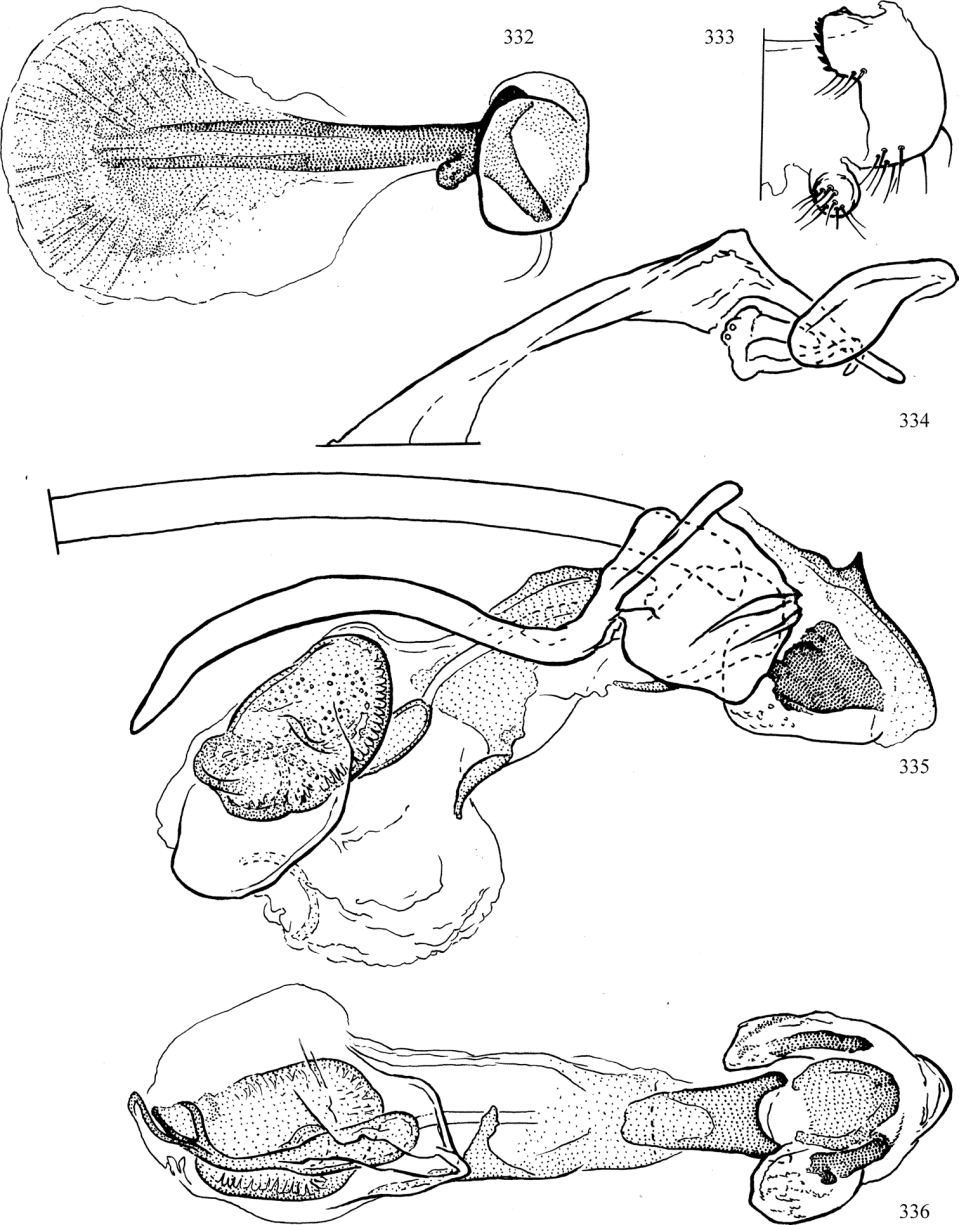
*Ophiomyiacapitolia* Lonsdale, sp. nov., male genitalia **332** ejaculatory apodeme **333** external genitalia, ventral **334** hypandrium, ventral **335** hypandrial complex, left lateral **336** phallus and proepiphallus, ventral.

**Figures 337–341.**
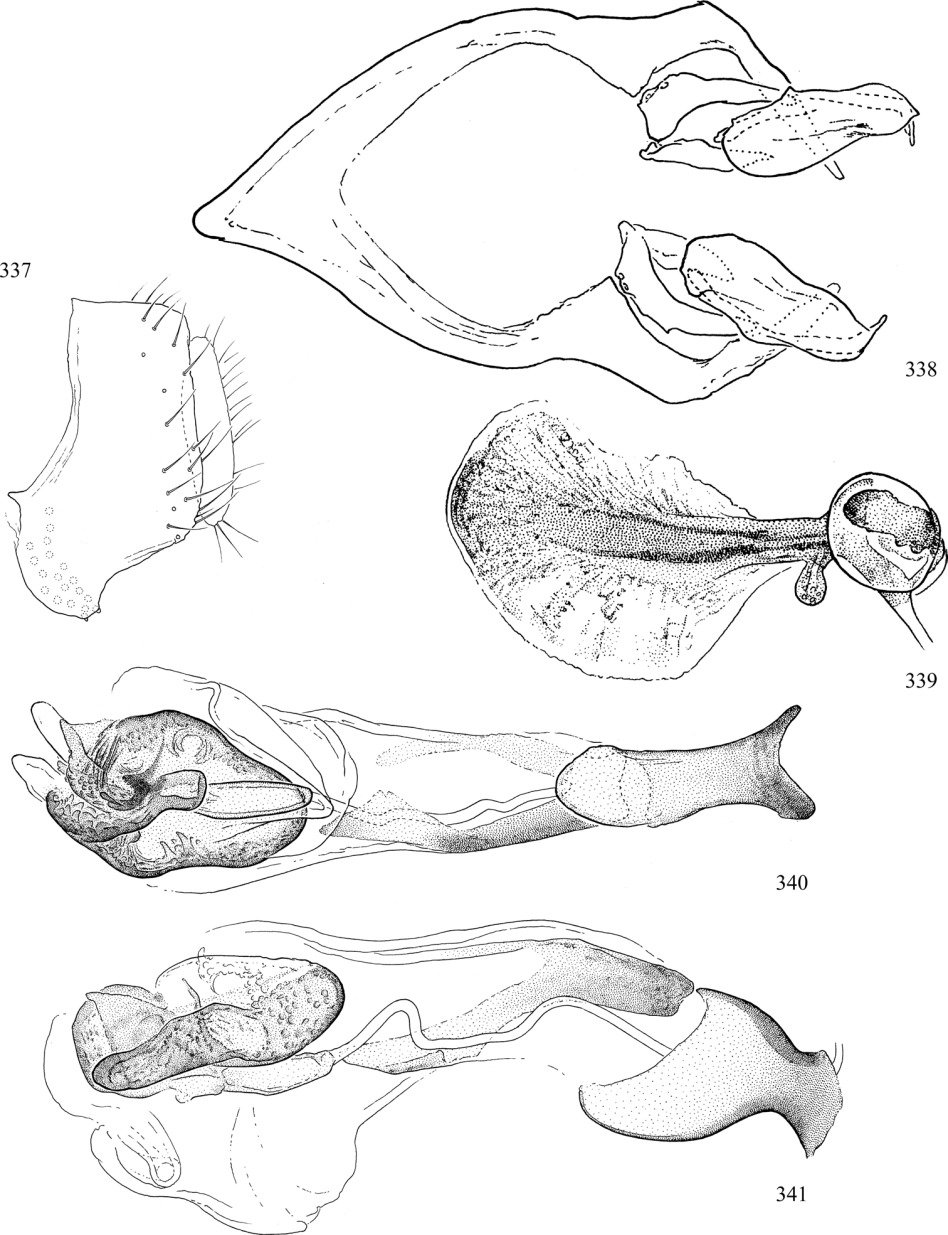
*Ophiomyiaconiceps* (Malloch), male genitalia **337** external genitalia, left lateral **338** hypandrium, ventral **339** ejaculatory apodeme **340** phallus, ventral **341** phallus, left lateral.

**Figures 342–345.**
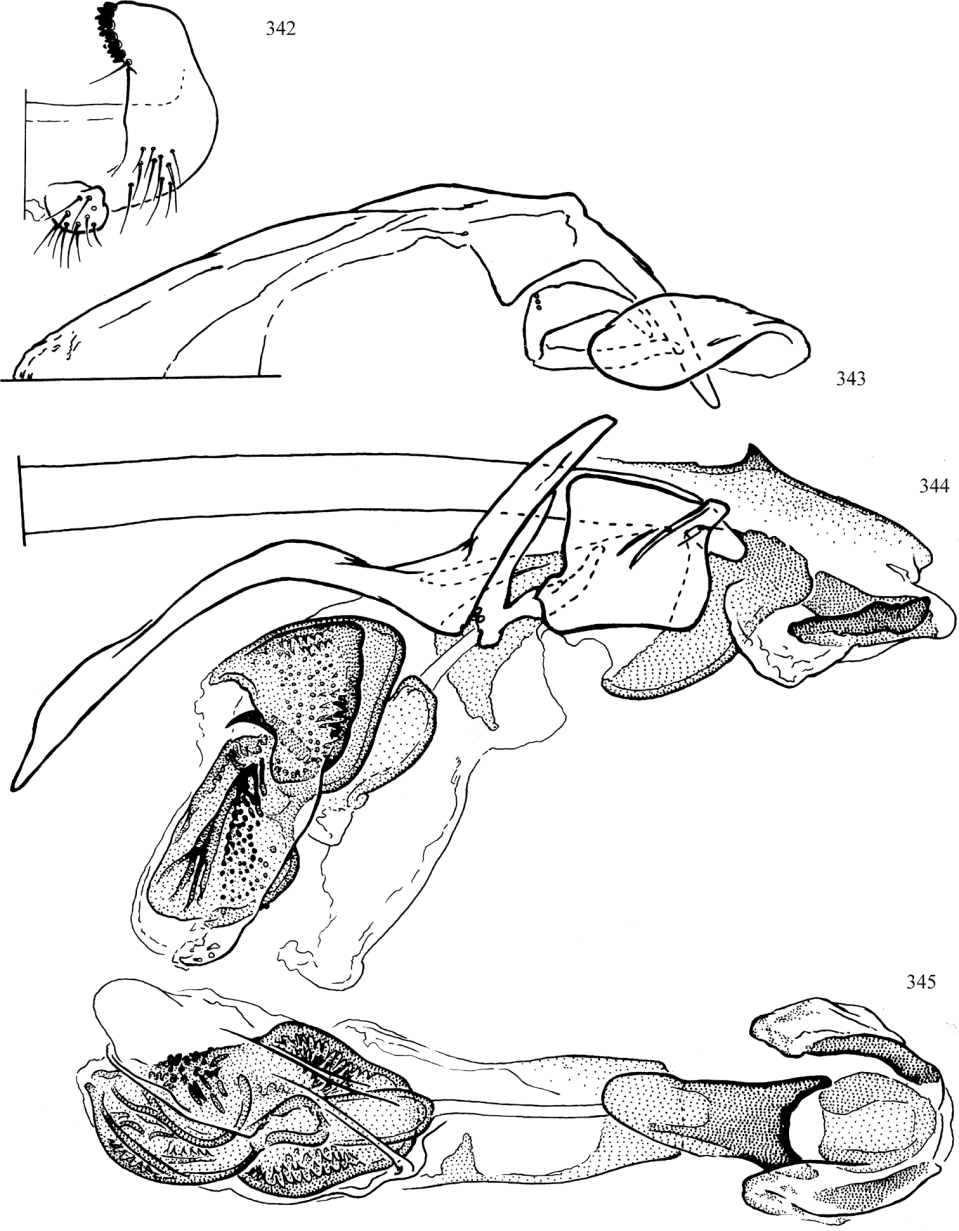
*Ophiomyiacuprea* Lonsdale, sp. nov., male genitalia **342** external genitalia, ventral **343** hypandrium, ventral **344** hypandrial complex, left lateral **345** phallus and proepiphallus, ventral.

**Figures 346–350.**
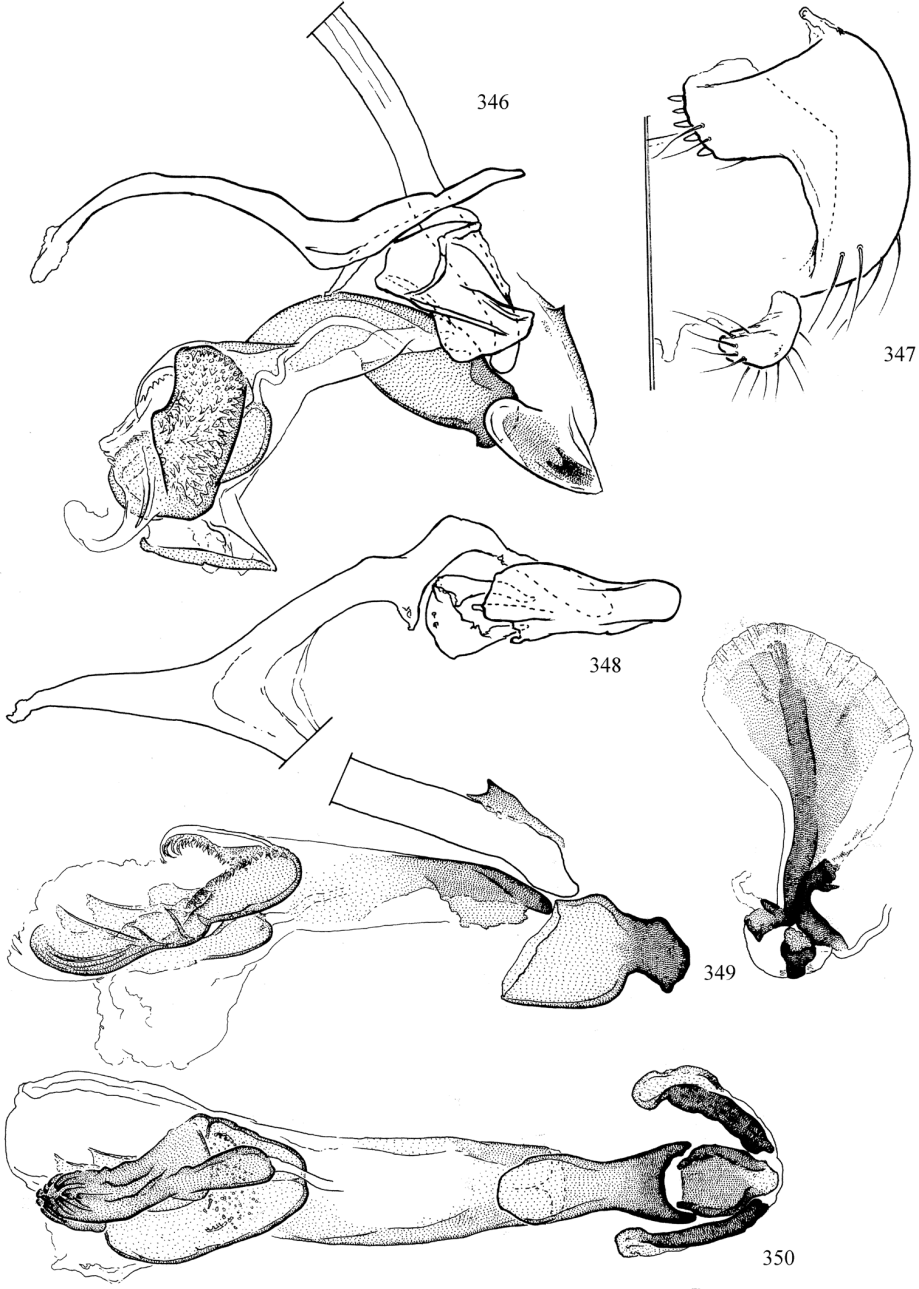
*Ophiomyiagaliodes* Lonsdale, sp. nov. **346** hypandrial complex, left lateral **347** external genitalia, ventral **348–350***O.heleios* Lonsdale, sp. nov. **348** hypandrium, ventral **349** hypandrial complex (excluding hypandrium and proepiphallus), left lateral **350** phallus and proepiphallus, ventral.

**Figures 351–355.**
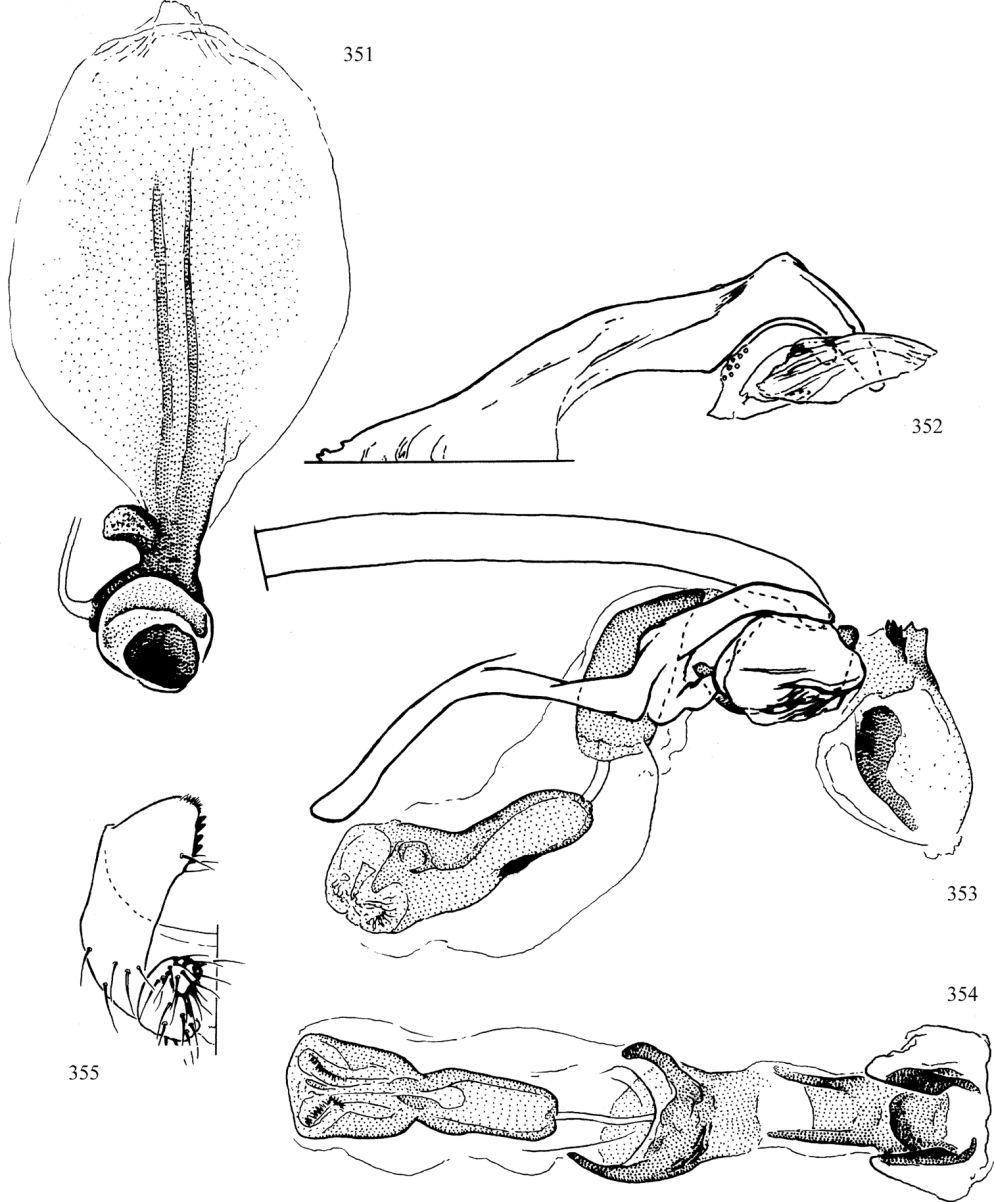
*Ophiomyiakalia* Lonsdale, sp. nov., male genitalia **351** ejaculatory apodeme **352** hypandrium, ventral **353** hypandrial complex, left lateral **354** phallus and proepiphallus, ventral **355** external genitalia, ventral.

**Figures 356–359.**
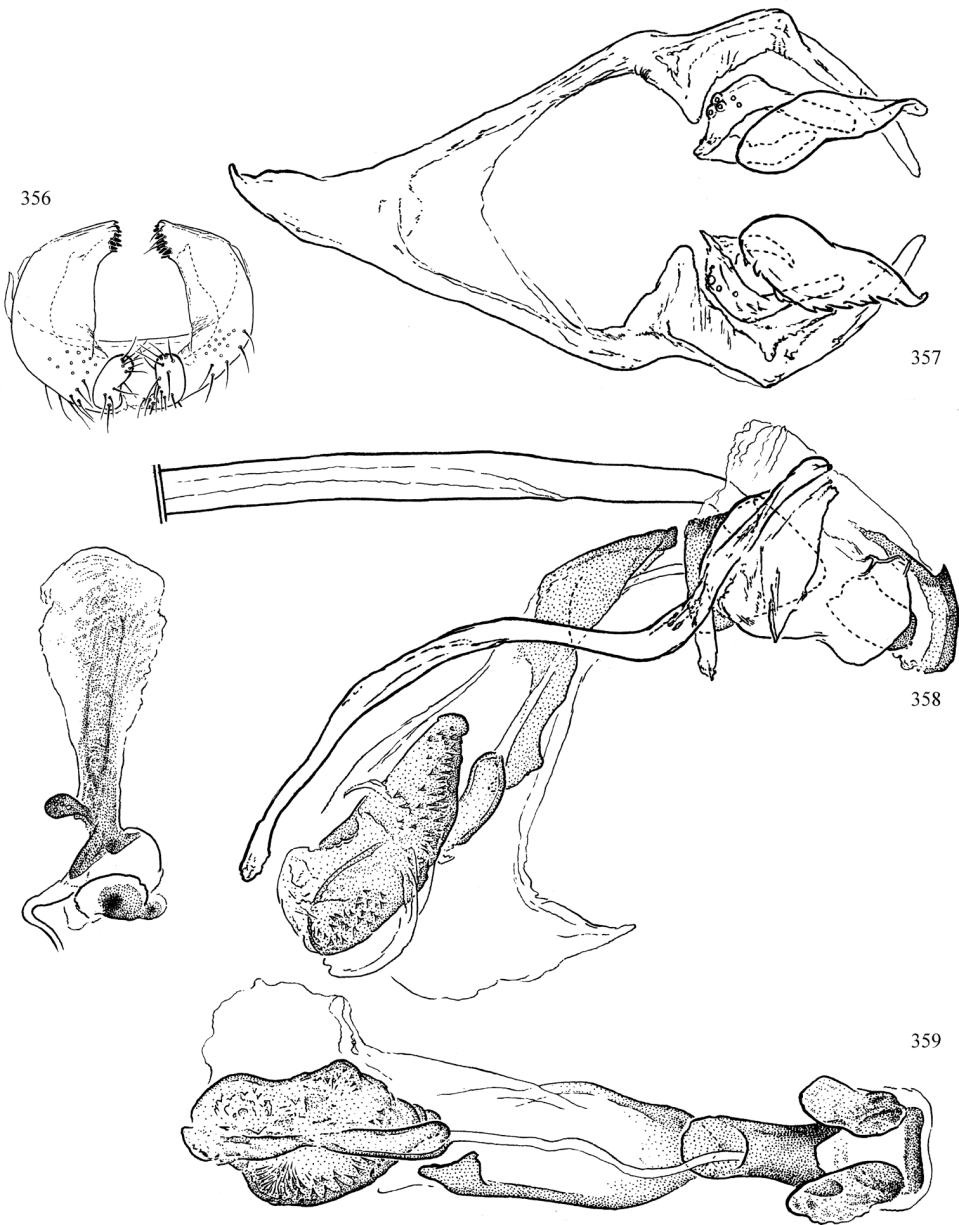
*Ophiomyiakwansonis* Sasakawa, male genitalia **356** external genitalia, ventral **357** hypandrium, ventral **358** hypandrial complex, left lateral **359** phallus and epiphallus, ventral.

**Figures 360–366.**
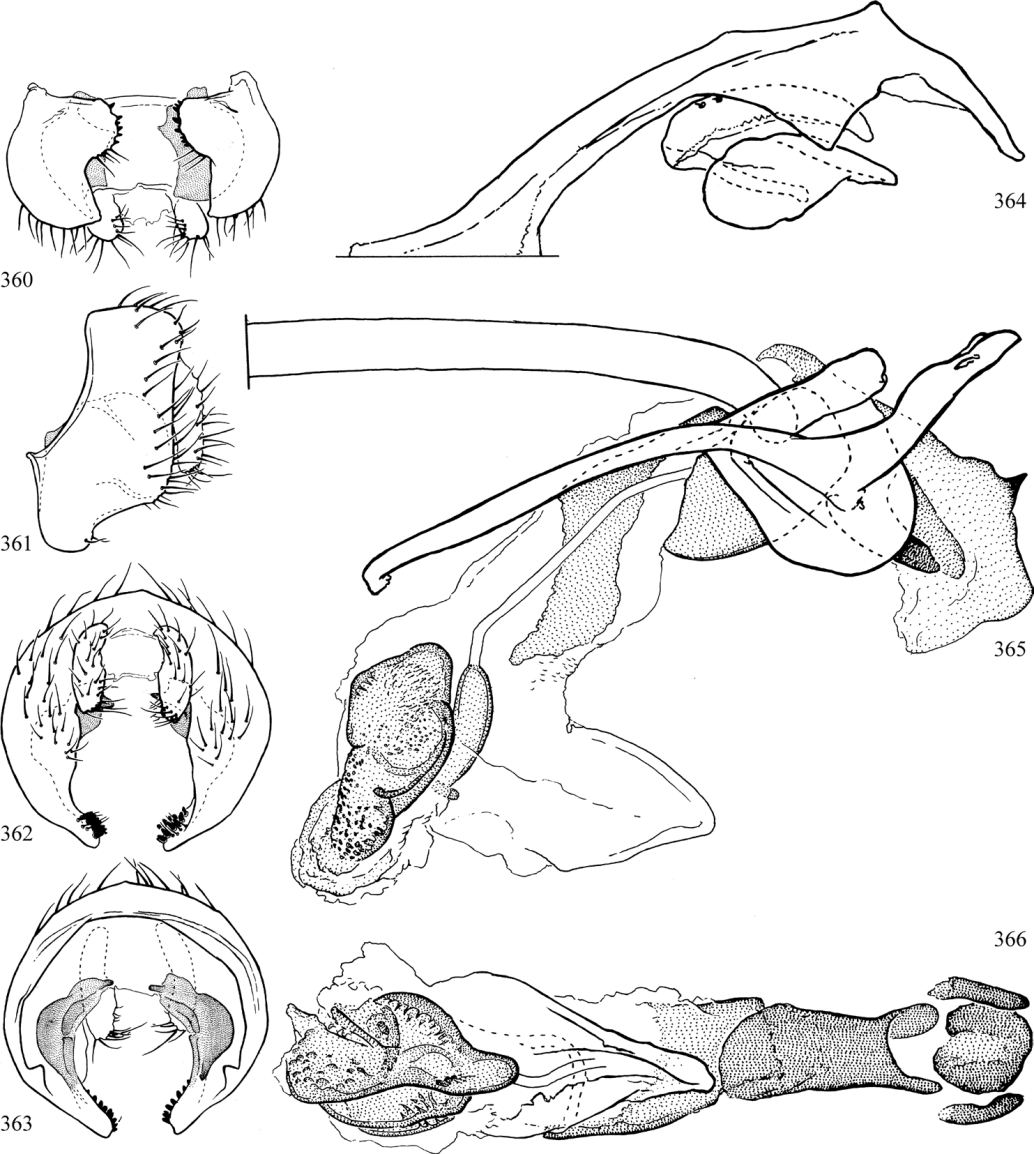
*Ophiomyialabiatarum* Hering, male genitalia **360** external genitalia, ventral (subepandrial sclerite shaded) **361** external genitalia, left lateral **362** external genitalia, posterior **363** external genitalia, anterior **364** hypandrium, ventral **365** hypandrial complex, left lateral **366** phallus and proepiphallus, ventral.

**Figures 367–371.**
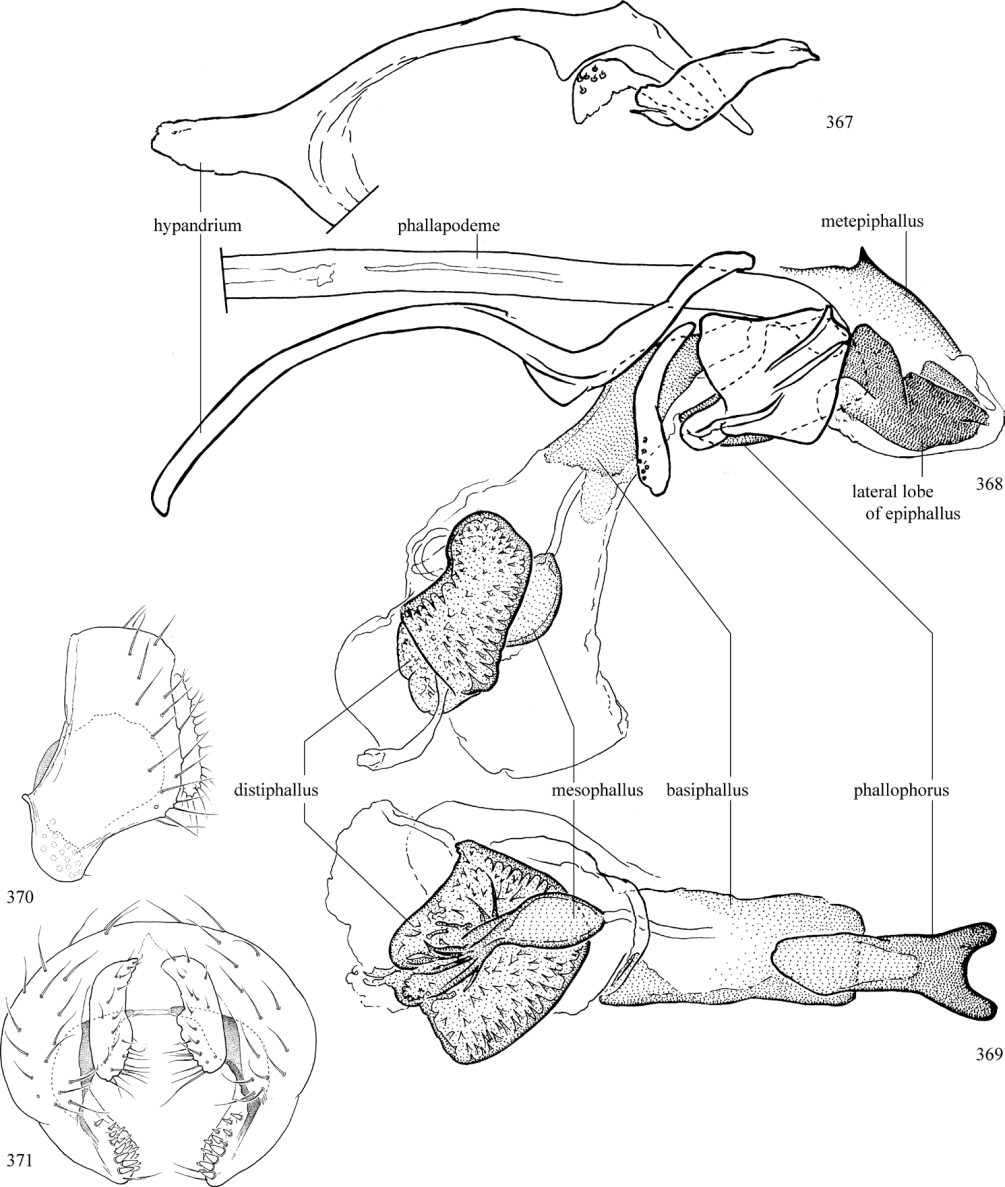
*Ophiomyialaticolis* sp. nov., male genitalia **367** hypandrium, ventral **368** hypandrial complex, left lateral **369** phallus, ventral **370** external genitalia, left lateral **371** external genitalia, posterior.

**Figures 372–376.**
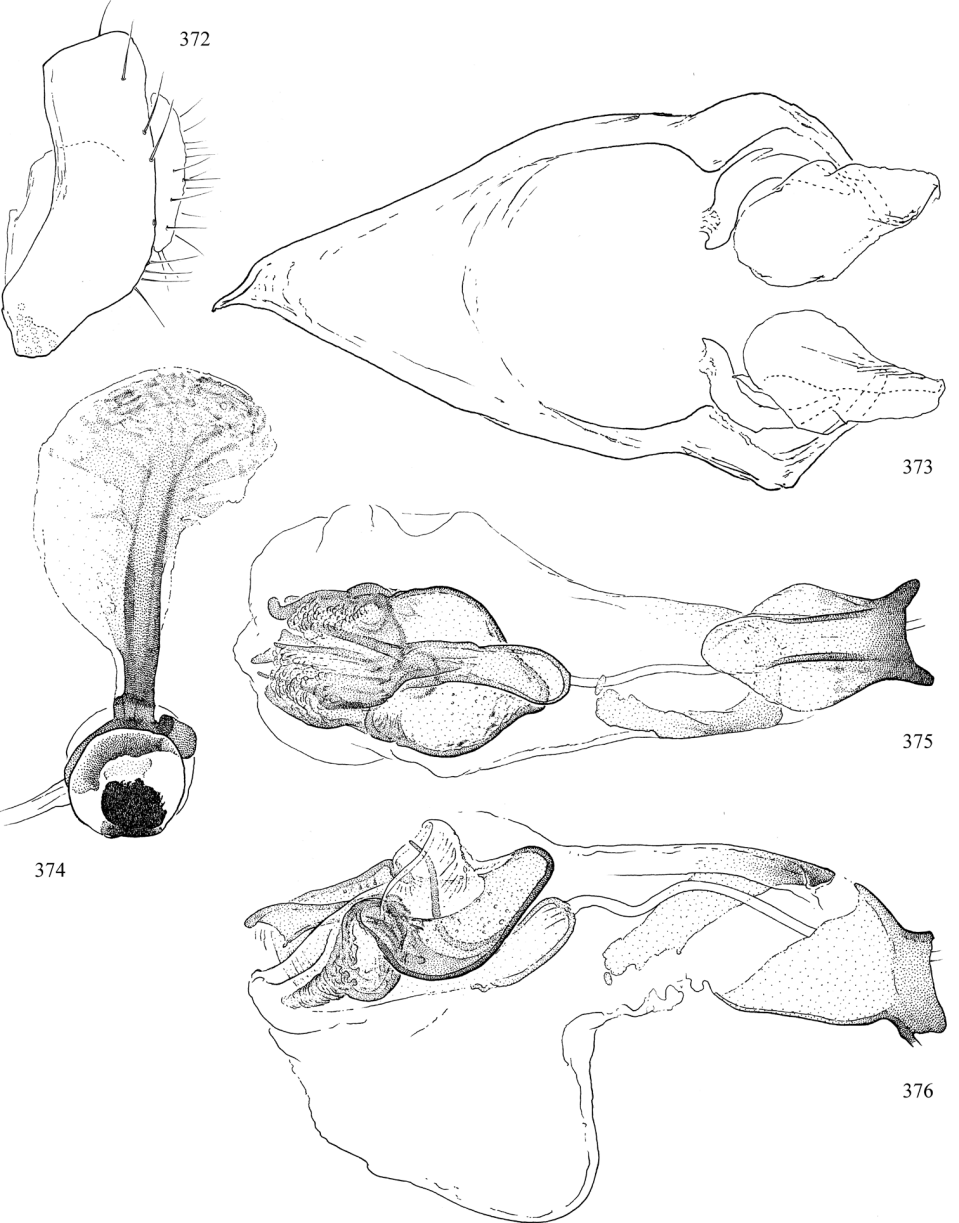
*Ophiomyiamaura* (Meigen), male genitalia **372** external genitalia, left lateral **373** hypandrium, ventral **374** ejaculatory apodeme **375** phallus, ventral **376** phallus, left lateral.

**Figures 377–382.**
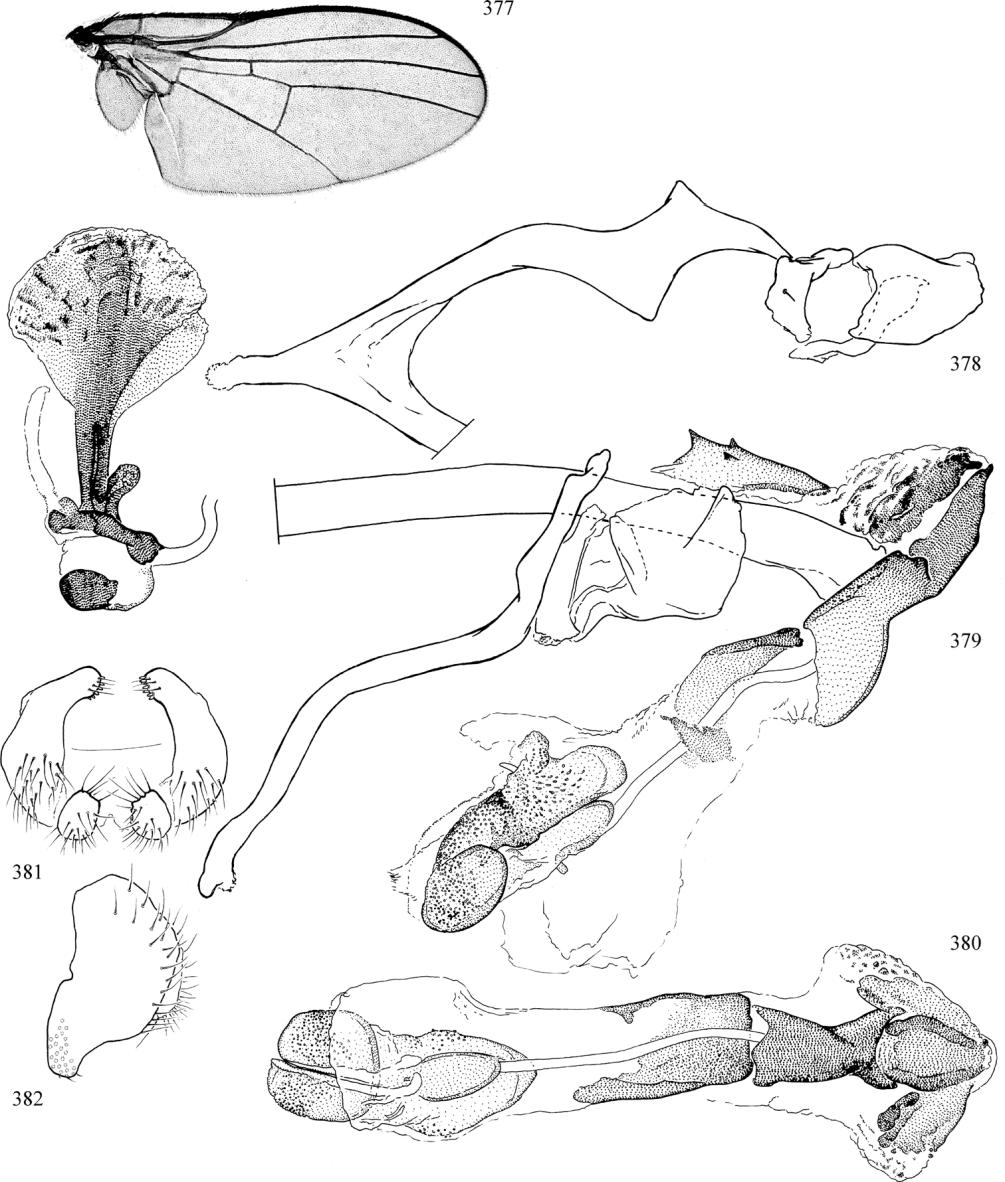
**377***Ophiomyianasuta* (Melander), wing **378–382***O.nasuta*, male genitalia **378** hypandrium, ventral **379** hypandrial complex, left lateral **380** phallus and proepiphallus, ventral **381** external genitalia, ventral **382** external genitalia, left lateral.

**Figures 383–387.**
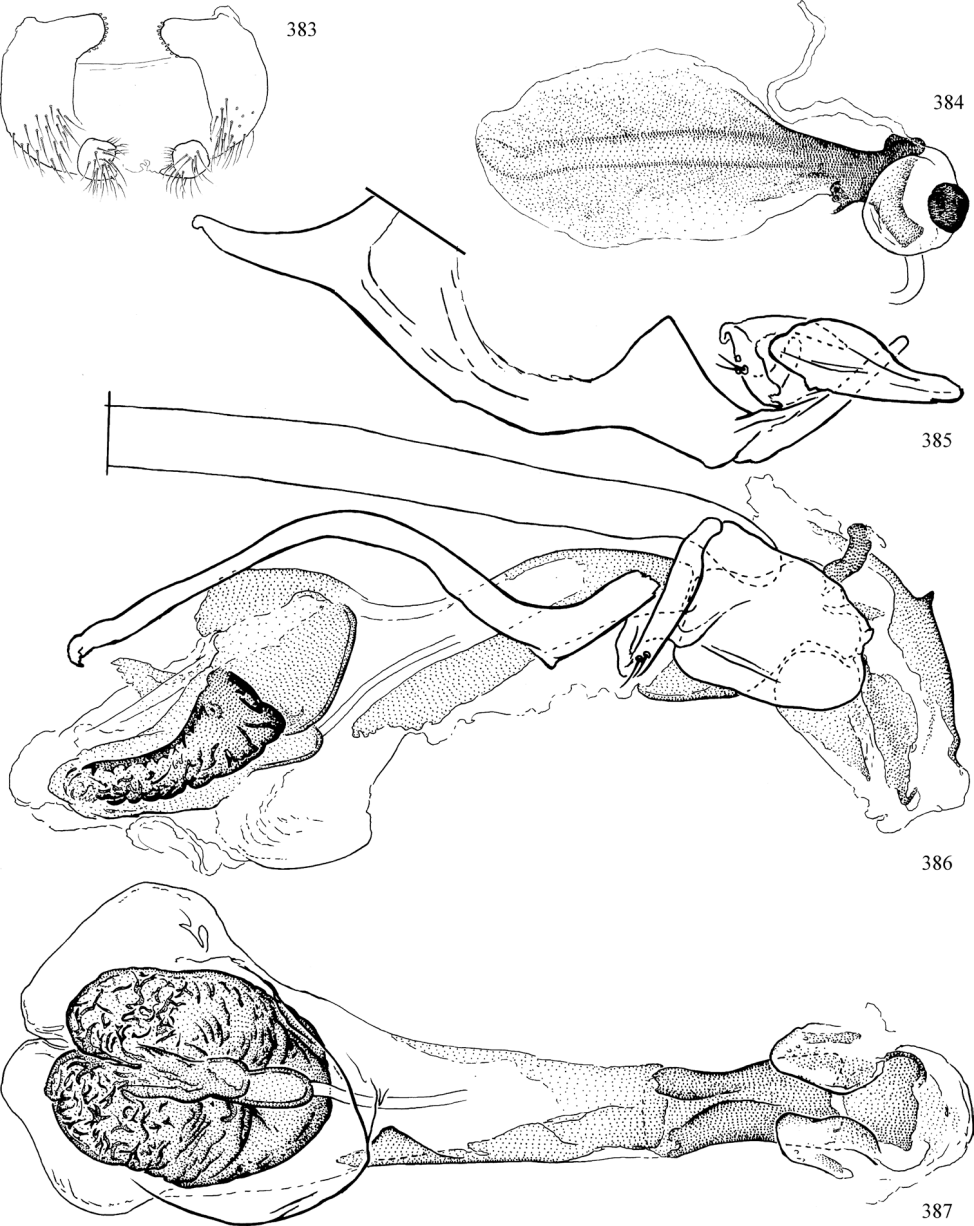
*Ophiomyiasexta* Spencer, male genitalia **383** external genitalia, ventral **384** ejaculatory apodeme **385** hypandrium, ventral **386** hypandrial complex, left lateral **387** phallus and proepiphallus, ventral.

**Figures 388–393.**
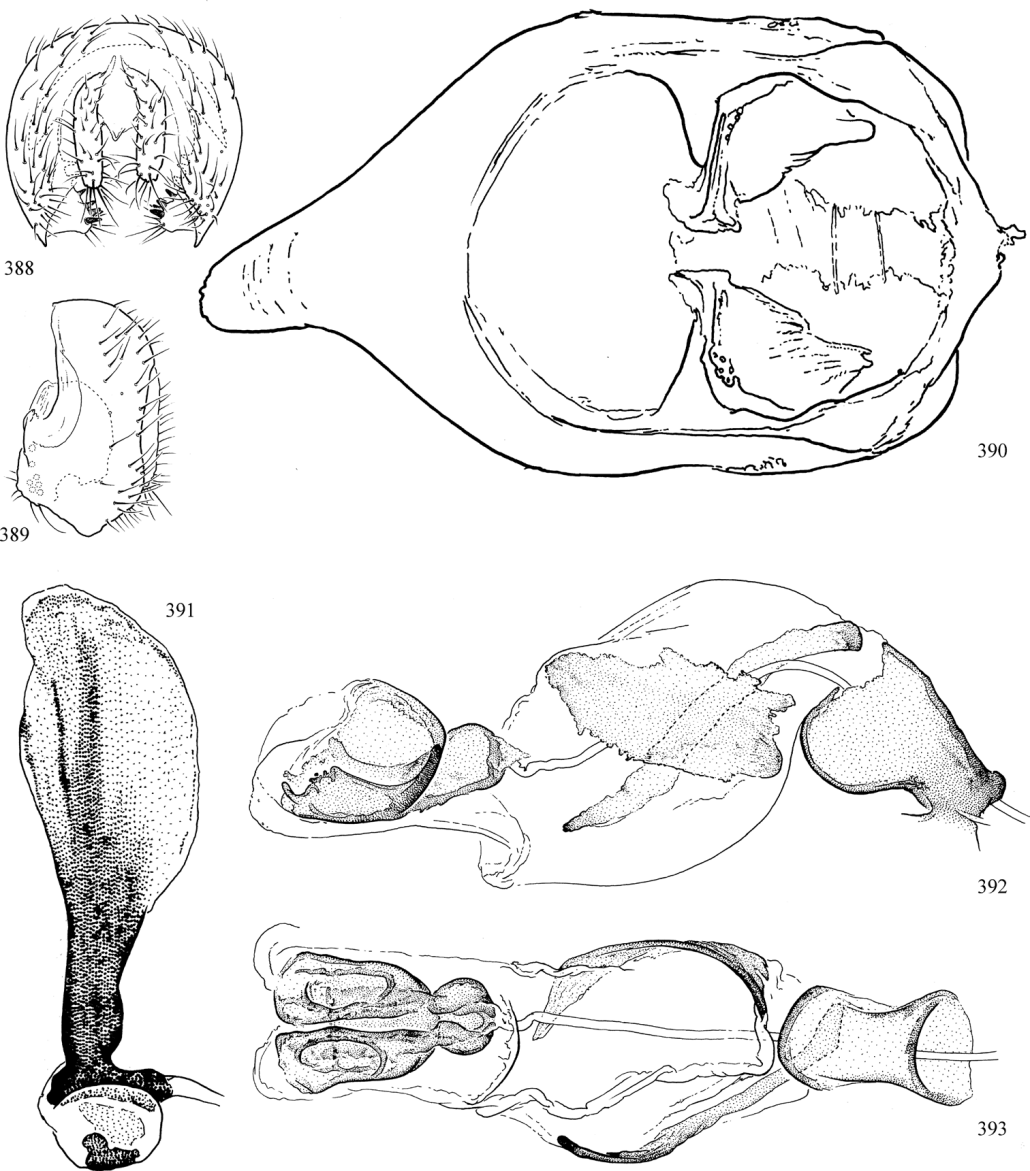
*Ophiomyiasimplex* (Loew), male genitalia **388** external components, posterior **389** external components, left lateral **390** hypandrium, ventral **391** ejaculatory apodeme **392** phallus, left lateral **393** phallus, ventral.

**Figures 394–398.**
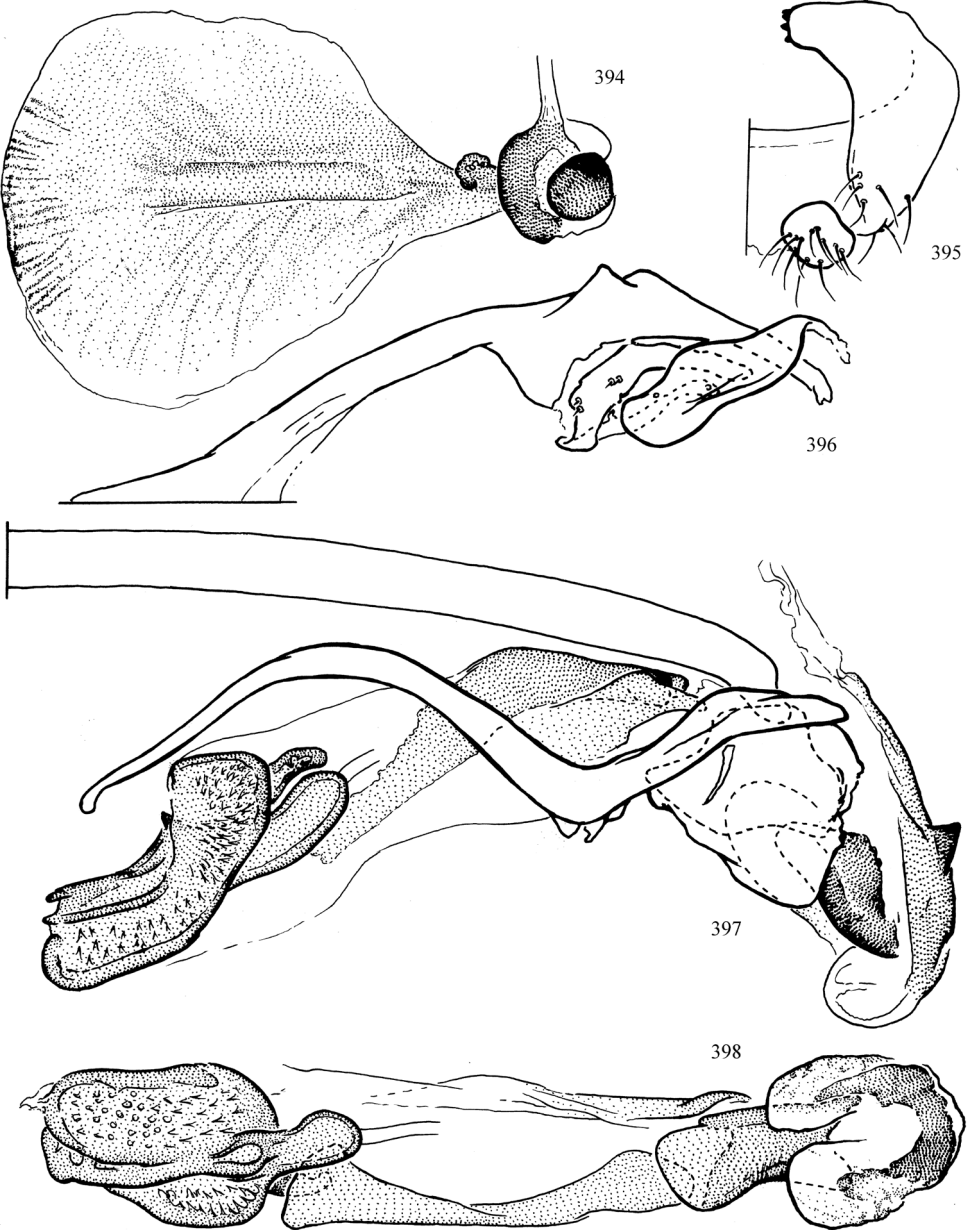
*Ophiomyiatexana* (Malloch), male genitalia **394** ejaculatory apodeme **395** external genitalia, ventral **396** hypandrium, ventral **397** hypandrial complex, left lateral **398** phallus and proepiphallus, ventral.

**Figures 399–404.**
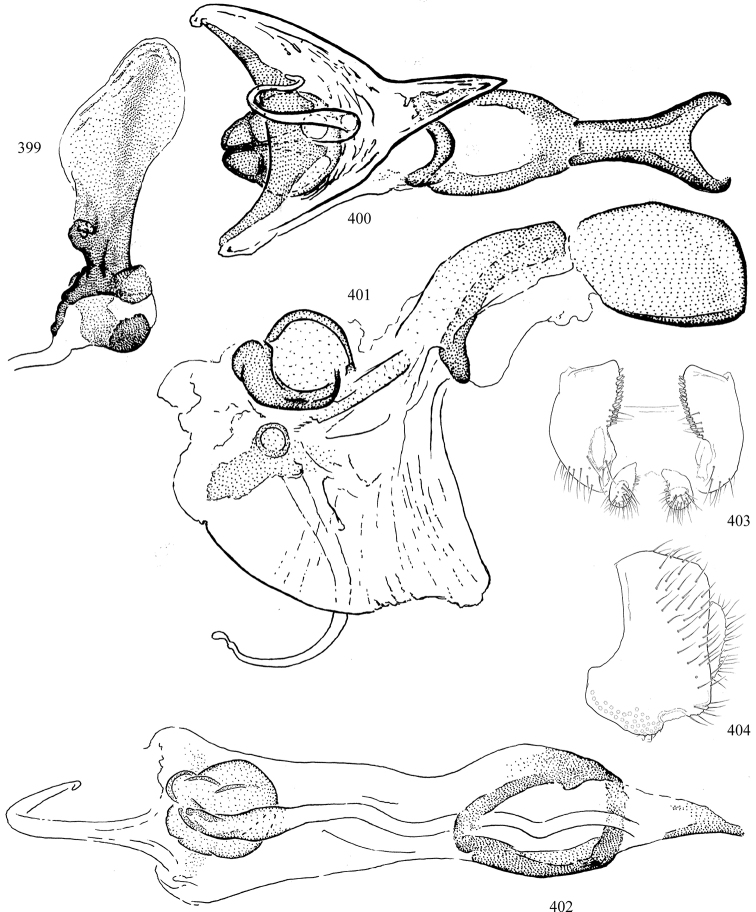
*Ophiomyiatiliae* (Couden), male genitalia **399** ejaculatory apodeme **400** phallus, ventral **401** phallus, left lateral **402** holotype phallus, ventral **403** external genitalia, ventral **404** external genitalia, left lateral.

**Figures 405–410.**
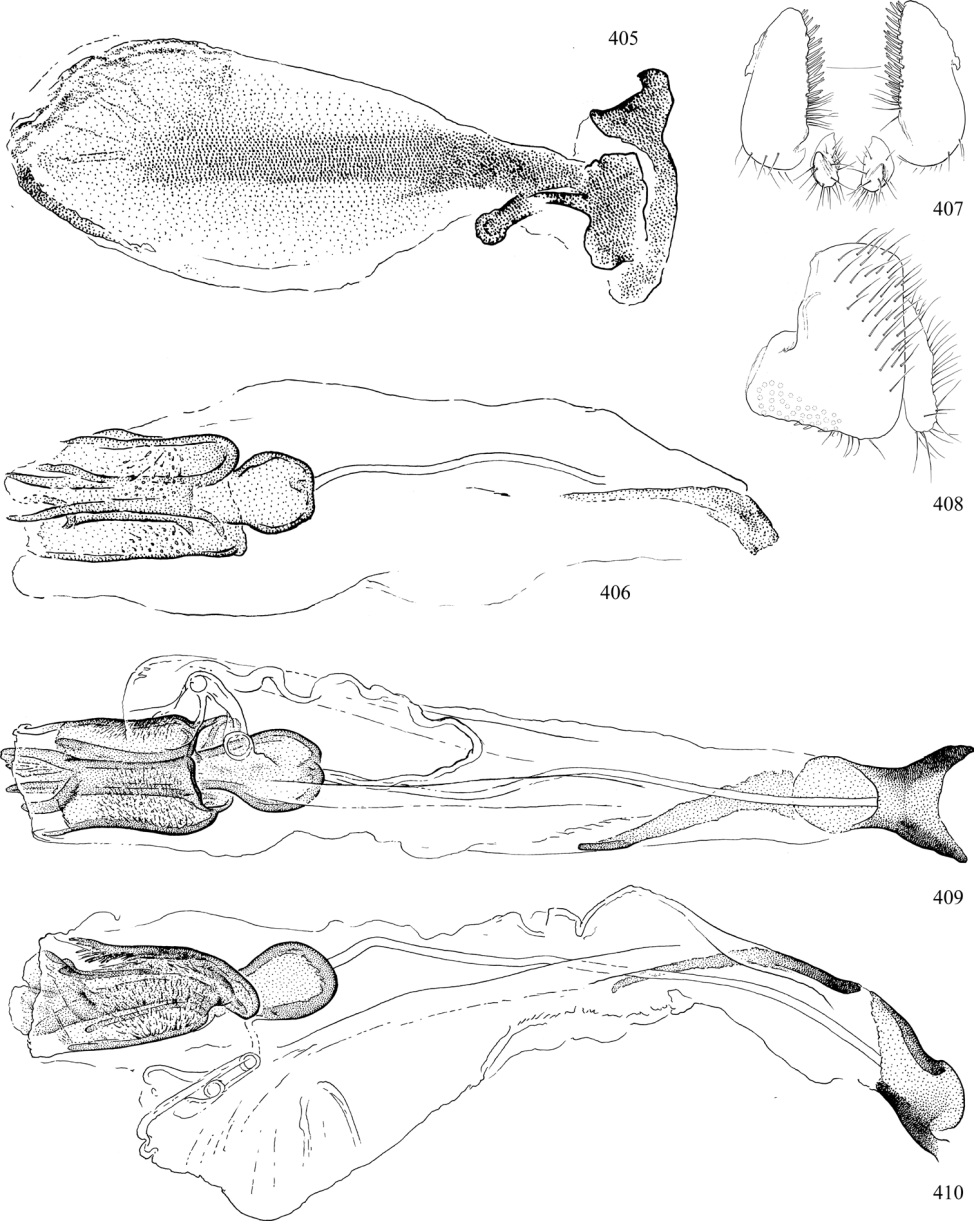
*Ophiomyiaultima* (Spencer), male holotype genitalia **405** ejaculatory apodeme **406** phallus, ventral **407–410***O.ultima*, Ontario non-type male **407** external genitalia, ventral **408** external genitalia, left lateral **409** phallus, ventral **410** phallus, left lateral.

**Figures 411, 412.**
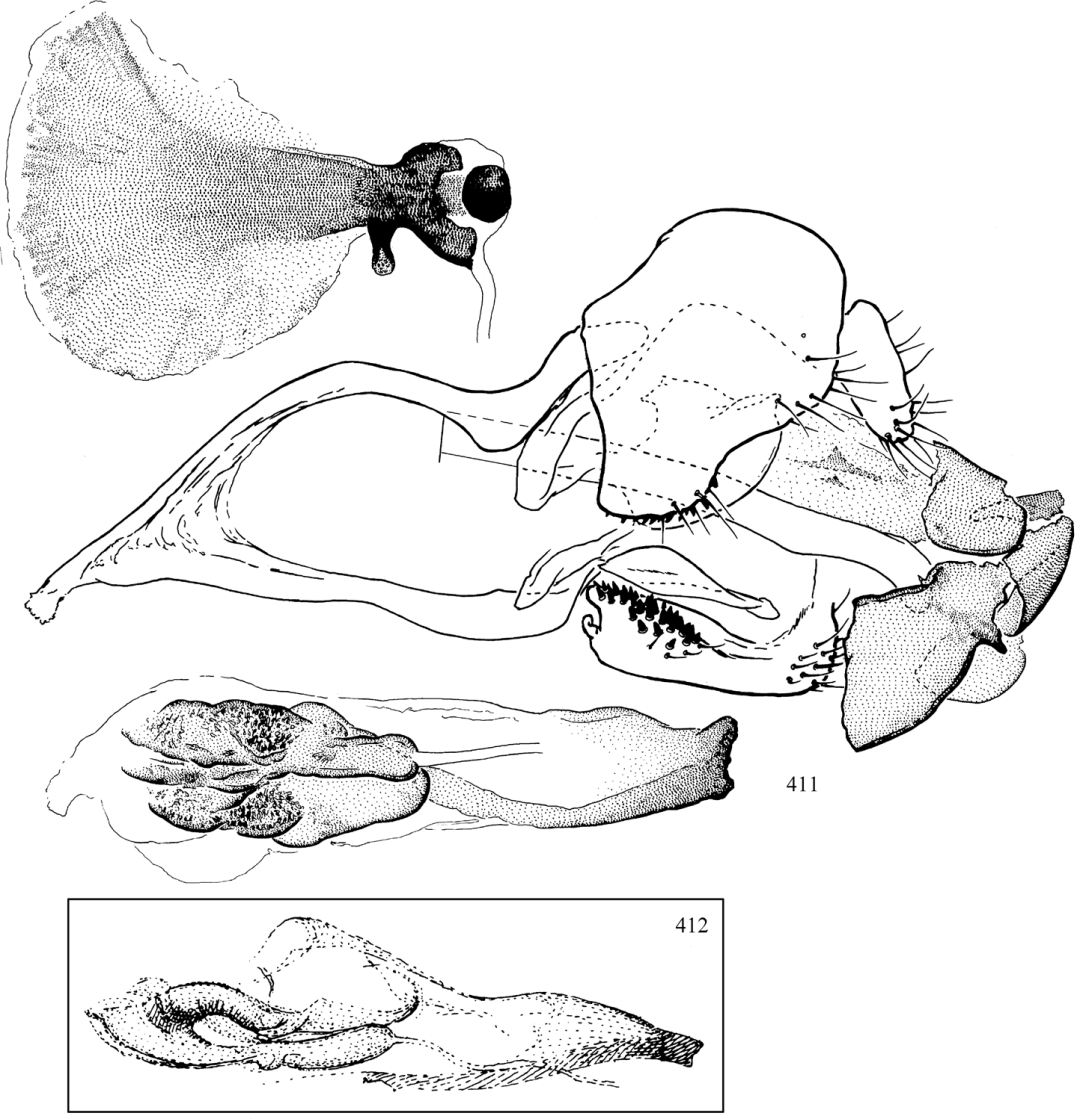
*Ophiomyiavirginiensis* Spencer, holotype **411** male genitalia **412** phallus, left lateral (from Spencer and Steyskal 1986b).

**Figures 413–417.**
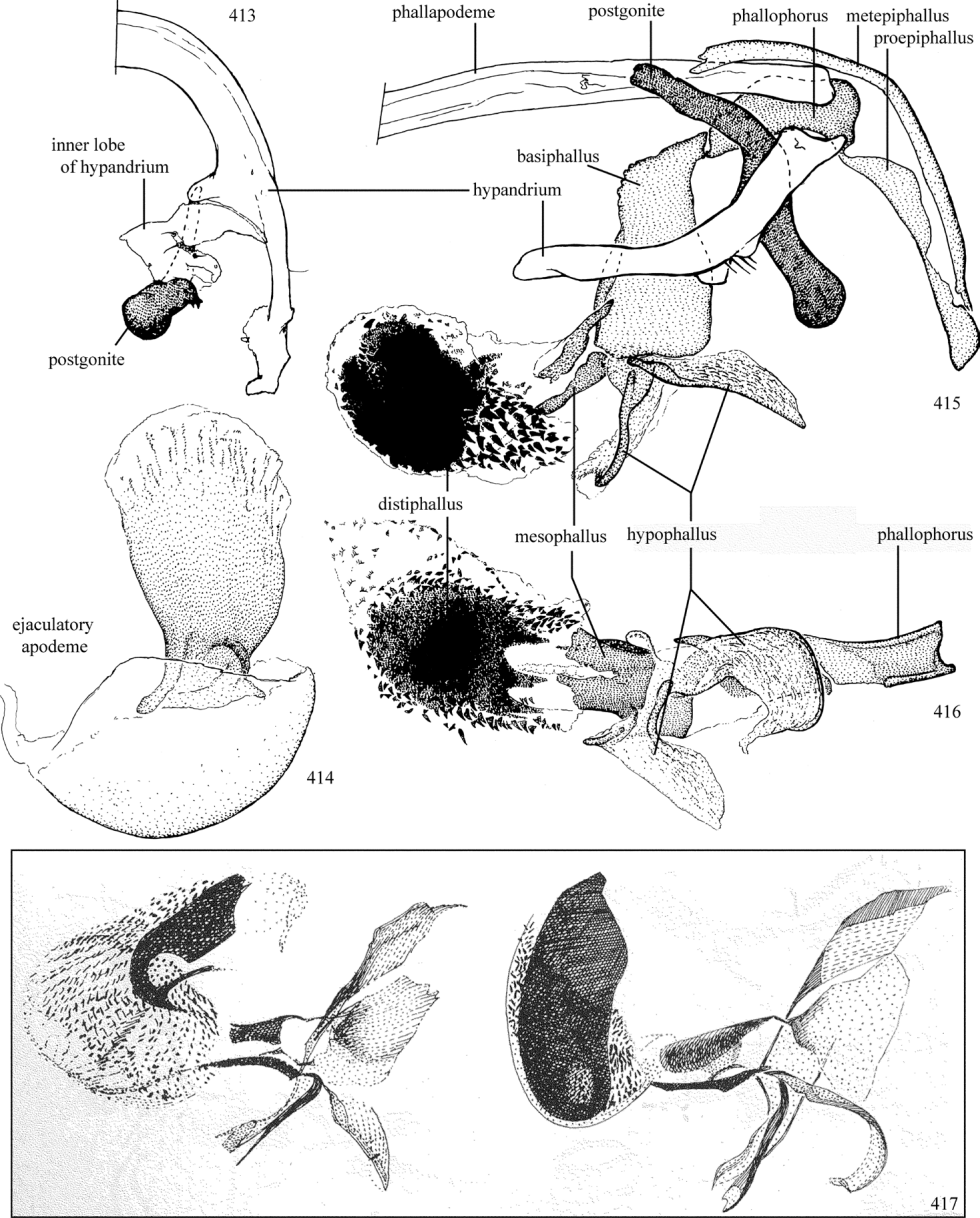
*Amauromyzaabnormalis* (Malloch), male genitalia **413** hypandrium and postgonite, ventral **414** ejaculatory apodeme **415** hypandrial complex, left lateral **416** phallus, ventral **417** phallus, left lateral, of males from California (left) and Quebec (right), from figures in Spencer and Steyskal (1986b).

**Figures 418–424.**
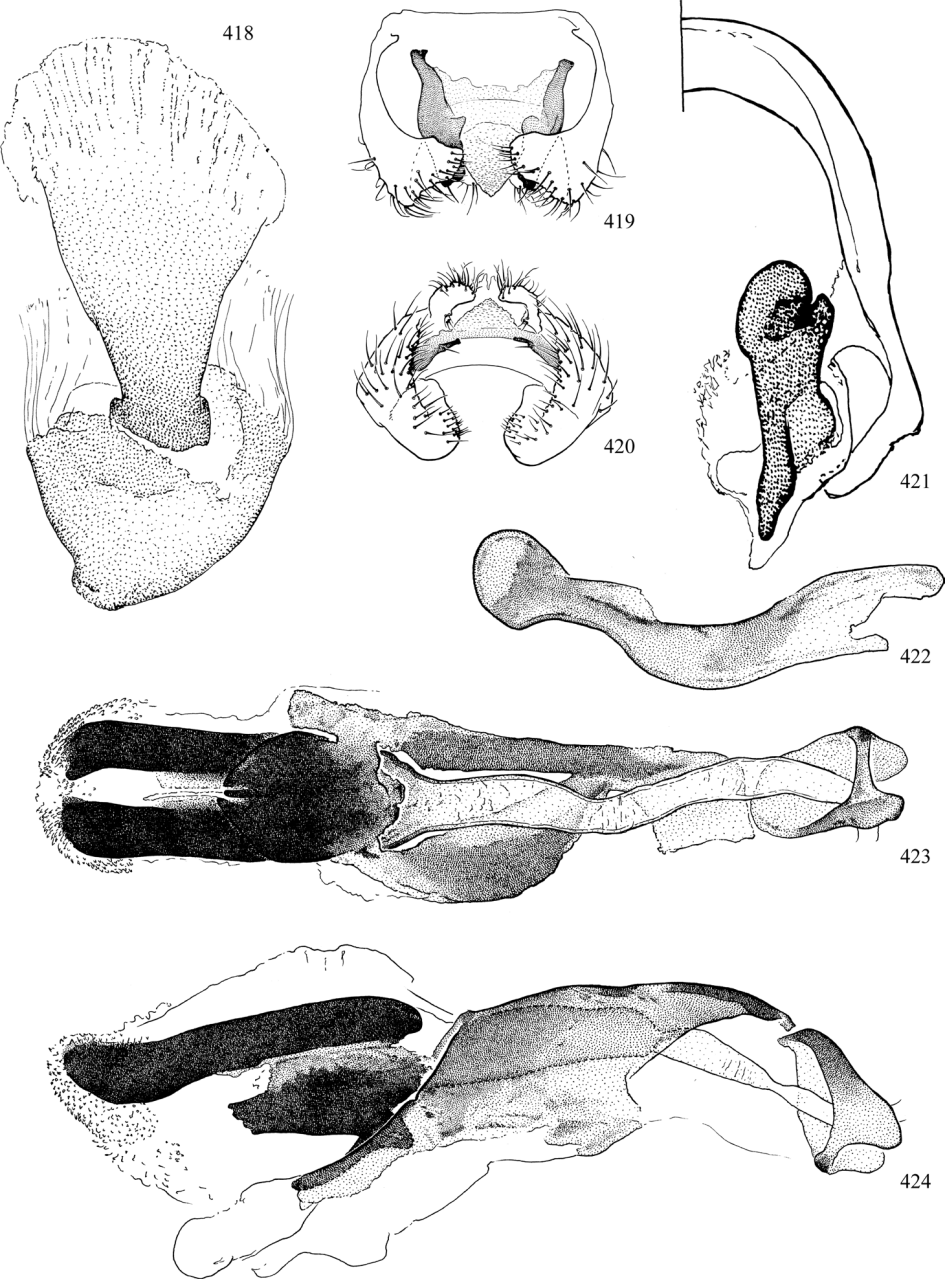
*Amauromyzaflavifrons* (Meigen), male genitalia **418** ejaculatory apodeme **419** external genitalia, anterior (subepandrial sclerite shaded) **420** external genitalia, ventral **421** hypandrium and postgonite, ventral **422** postgonite, lateral **423** phallus, ventral **424** phallus, left lateral.

**Figures 425–430.**
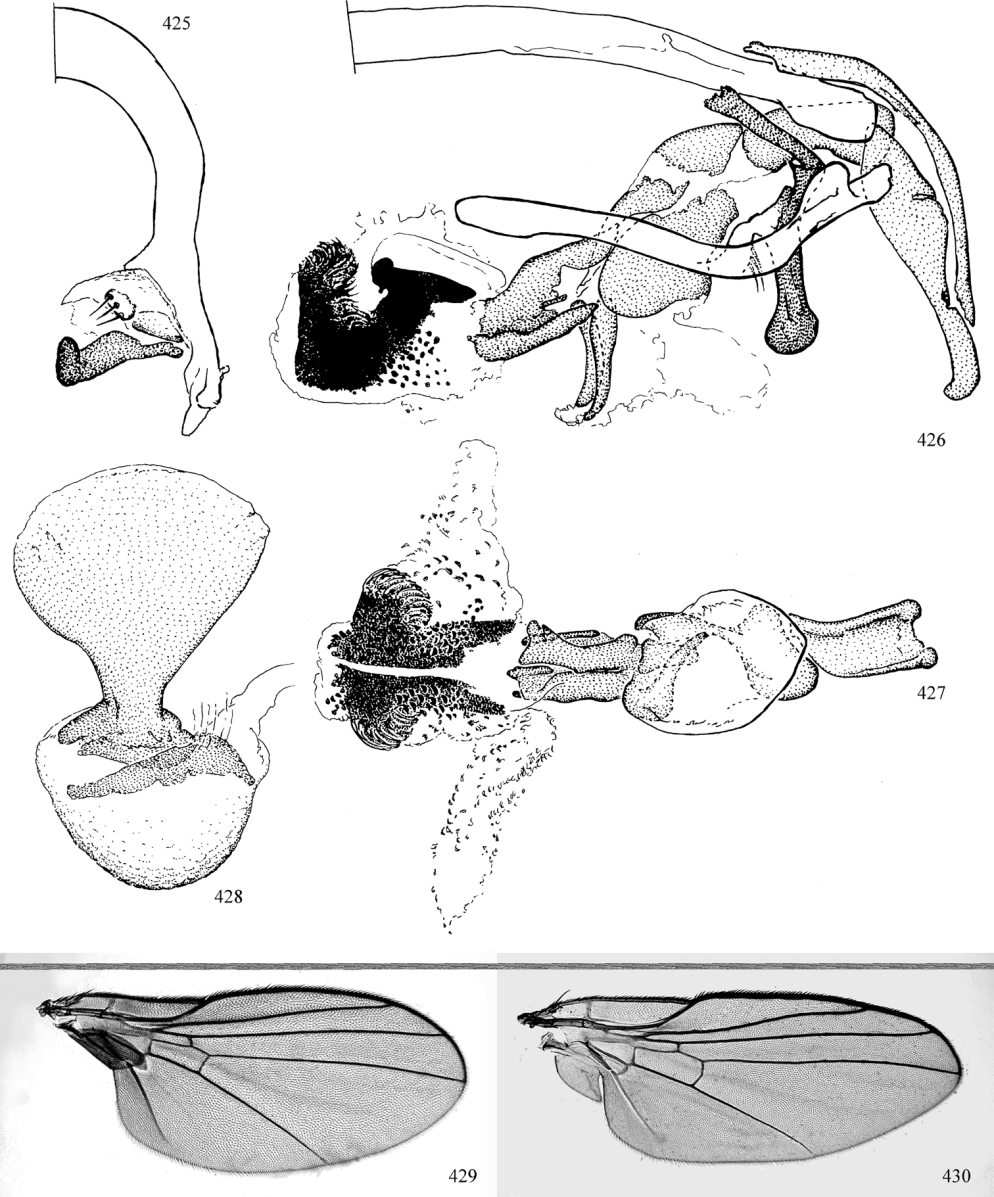
*Amauromyzakarli* (Hendel), male genitalia **425** hypandrium and postgonite, ventral **426** hypandrial complex, left lateral **427** phallus, ventral **428** ejaculatory apodeme **429, 430** wings **429***A.flavifrons* (Meigen) **430***A.karli*.

**Figures 431–434.**
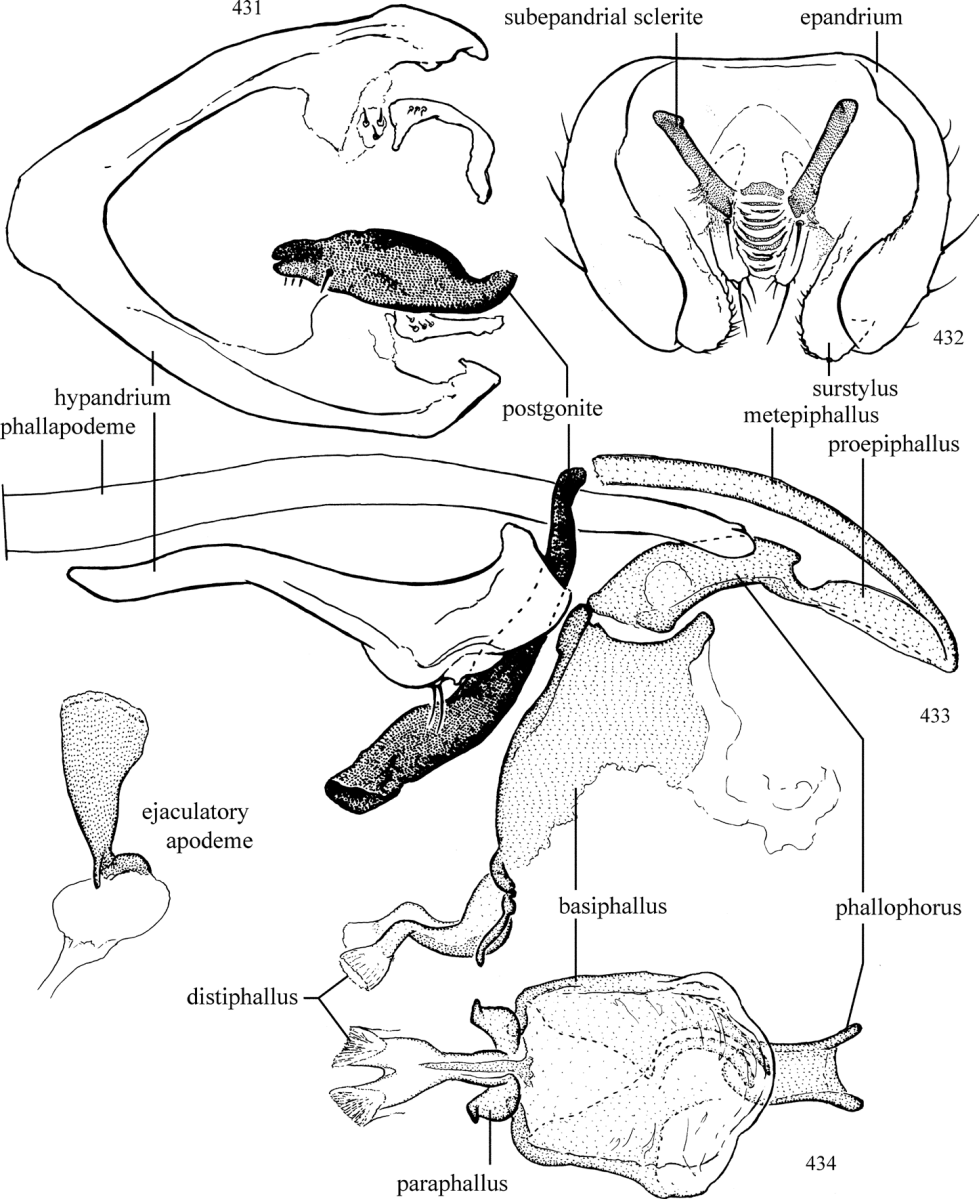
*Aulagromyzanitida* (Malloch), male genitalia **431** hypandrium and postgonite, ventral **432** external genitalia, anterior (subepandrial sclerite shaded) **433** hypandrial complex, left lateral **434** phallus, ventral.

**Figures 435–441.**
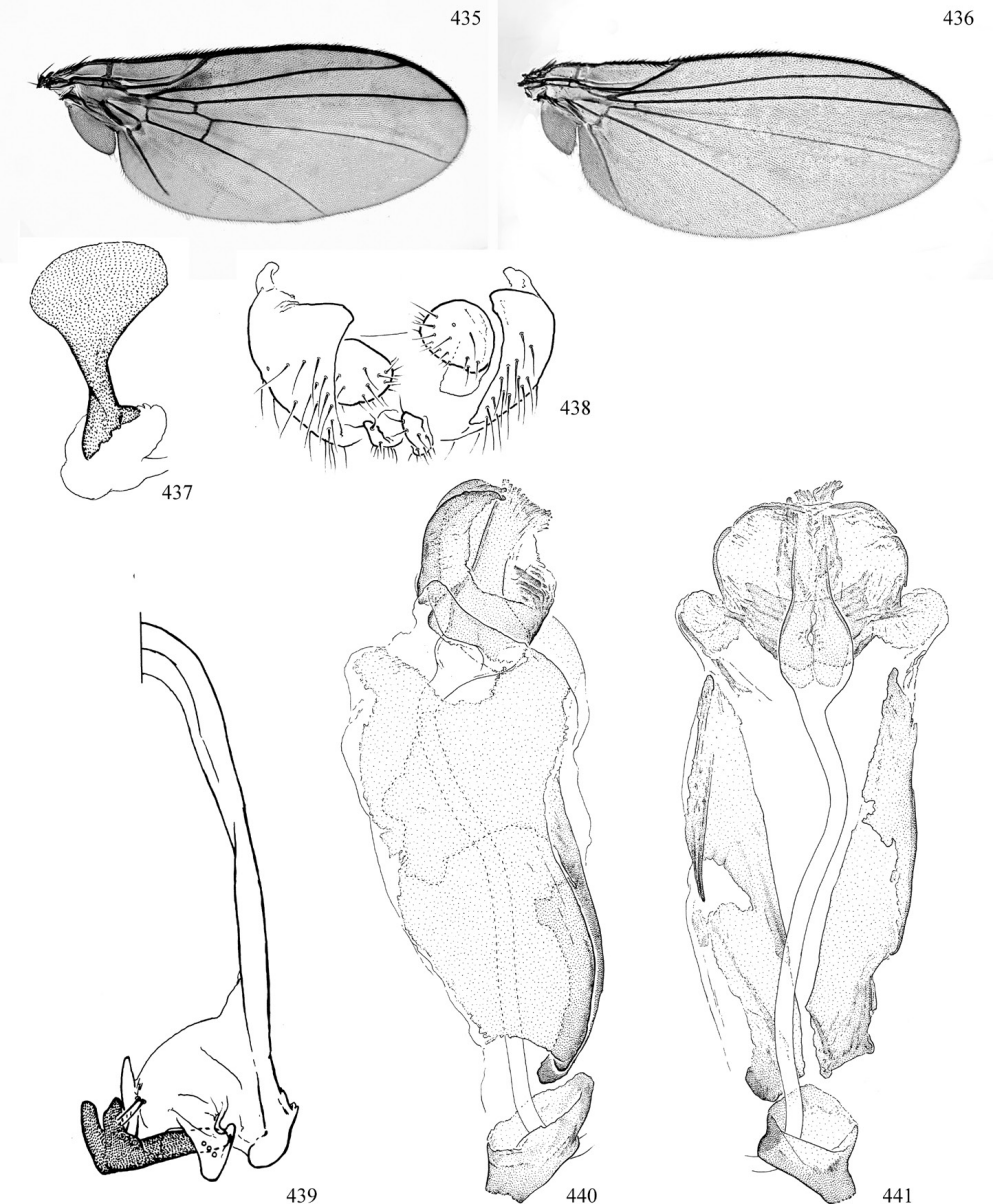
wings **435***Aulagromyzanitida* (Malloch) **436***A.orbitalis* (Melander) **437–441***A.orbitalis*, male genitalia **437** ejaculatory apodeme **438** external genitalia, ventral **439** hypandrium and postgonite, ventral **440** phallus, left lateral **441** phallus, ventral.

**Figures 442–445.**
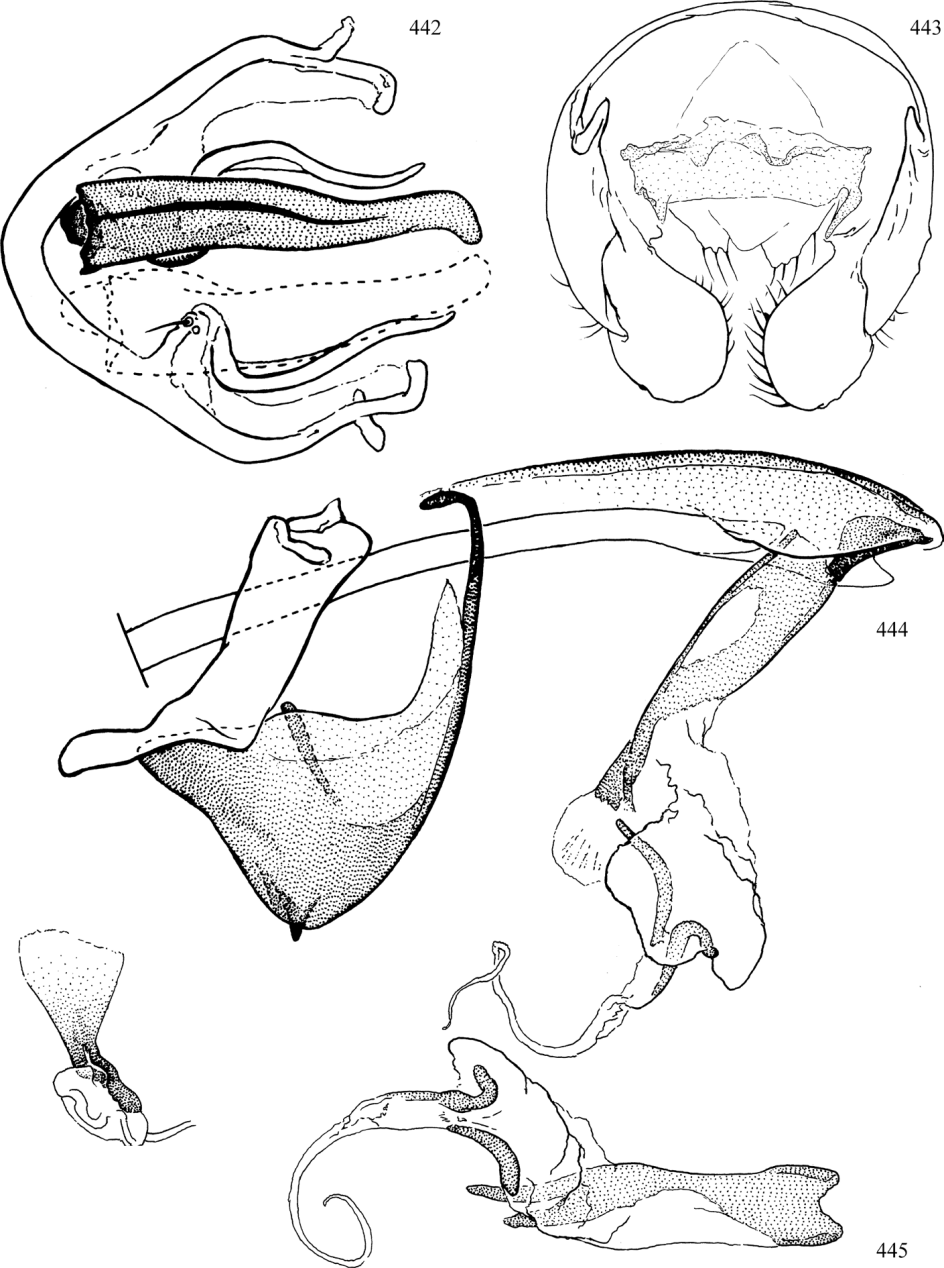
*Aulagromyzatridentata* (Loew), male genitalia **442** hypandrium and postgonite, ventral **443** external genitalia, anterior (subepandrial sclerite shaded) **444** hypandrial complex, left lateral **445** phallus, ventral.

**Figures 446–451.**
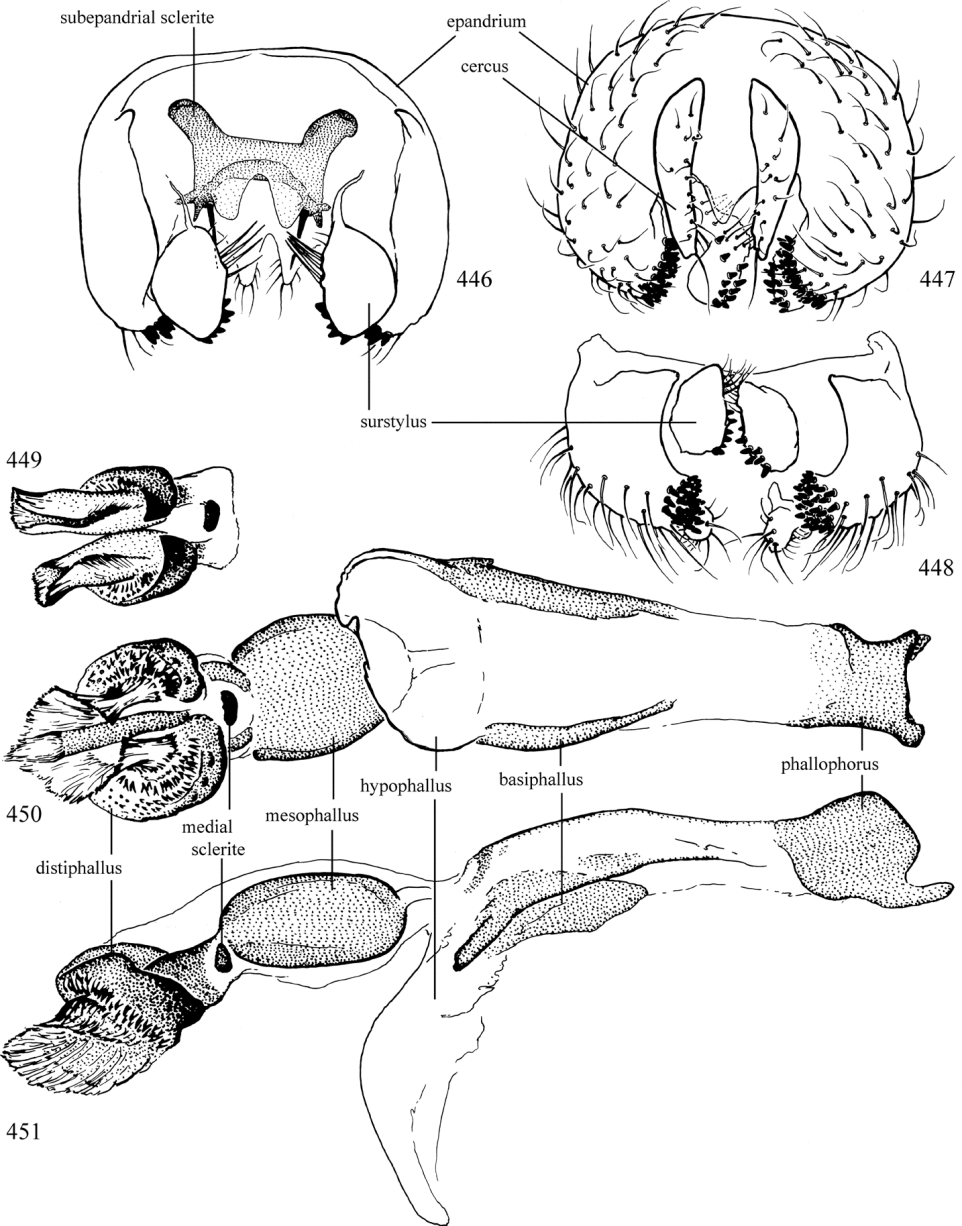
*Calycomyzaavira* Eiseman and Lonsdale, male genitalia, West Virginia male **446** external genitalia, anterior (subepandrial sclerite shaded) **447** external genitalia, posterior **448** external genitalia, ventral **449** distiphallus, ventral (Connecticut male) **450** phallus, ventral **451** phallus, left lateral. Originally published in Eiseman and Lonsdale (2018).

**Figures 452–459.**
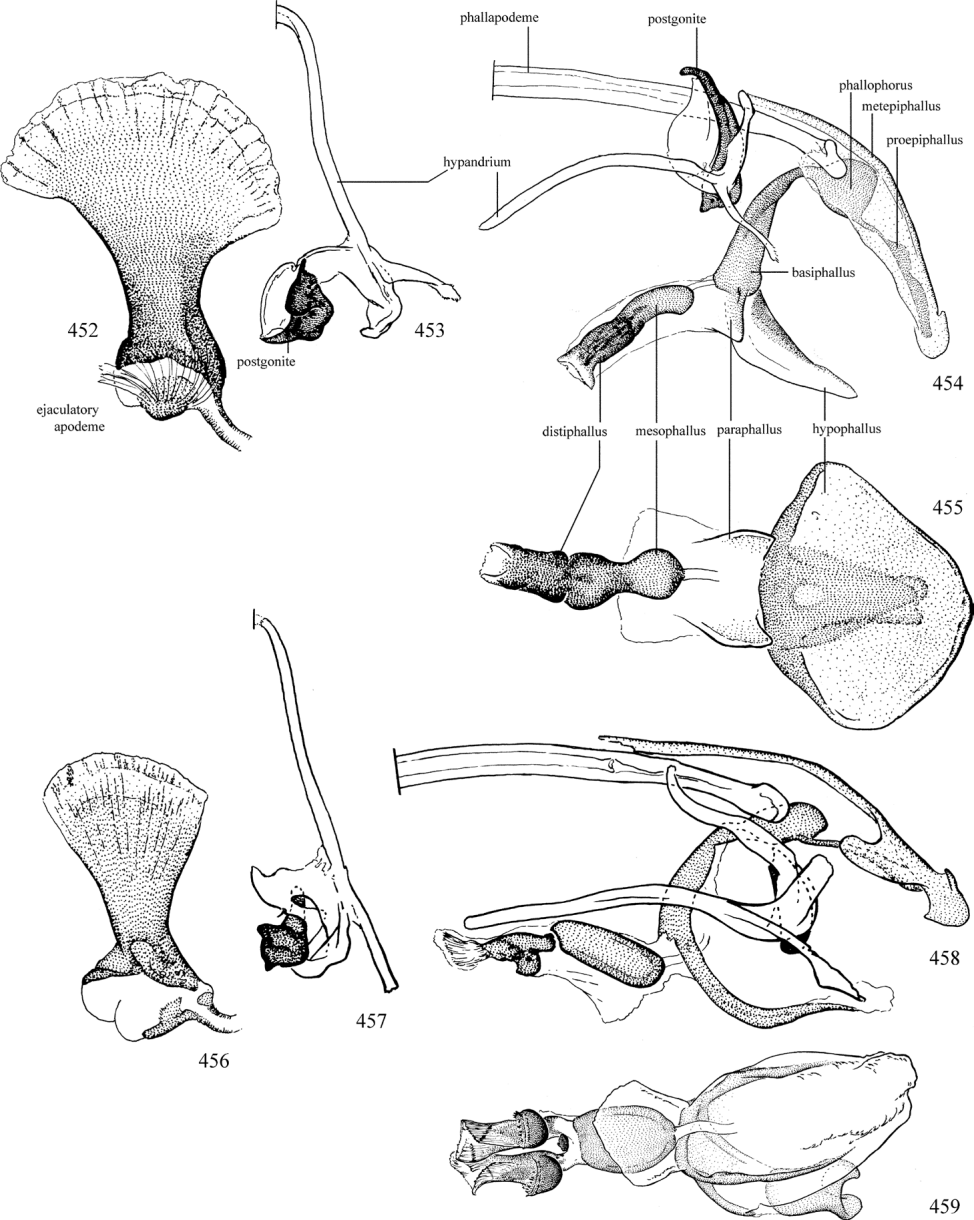
*Calycomyzabarbarensis* Spencer, male genitalia **452** ejaculatory apodeme **453** hypandrium and postgonite, ventral **454** hypandrial complex, left lateral **455** phallus, ventral **456–459***C.flavinotum* (Frick), male genitalia **456** ejaculatory apodeme **457** hypandrium and postgonite, ventral **458** hypandrial complex, left lateral **459** phallus, ventral.

**Figures 460–465.**
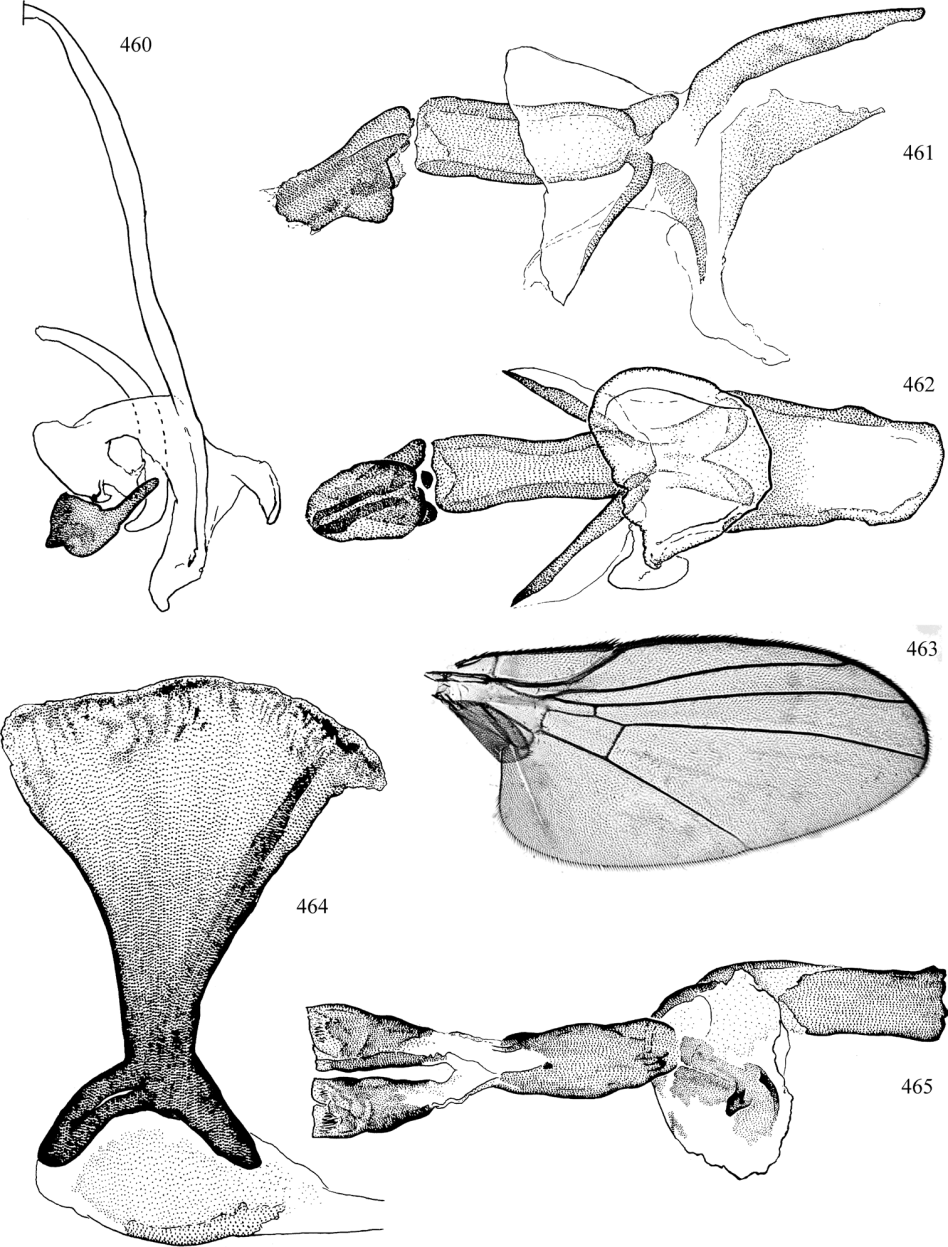
*Calycomyzafrickiana* Spencer, male genitalia **460** hypandrium and postgonite, ventral **461** phallus, left lateral **462** phallus, ventral **463***C.frickiana*, wing **464, 465***C.gigantea* (Frick), male holotype genitalia **464** ejaculatory apodeme **465** phallus, ventral.

**Figures 466–474.**
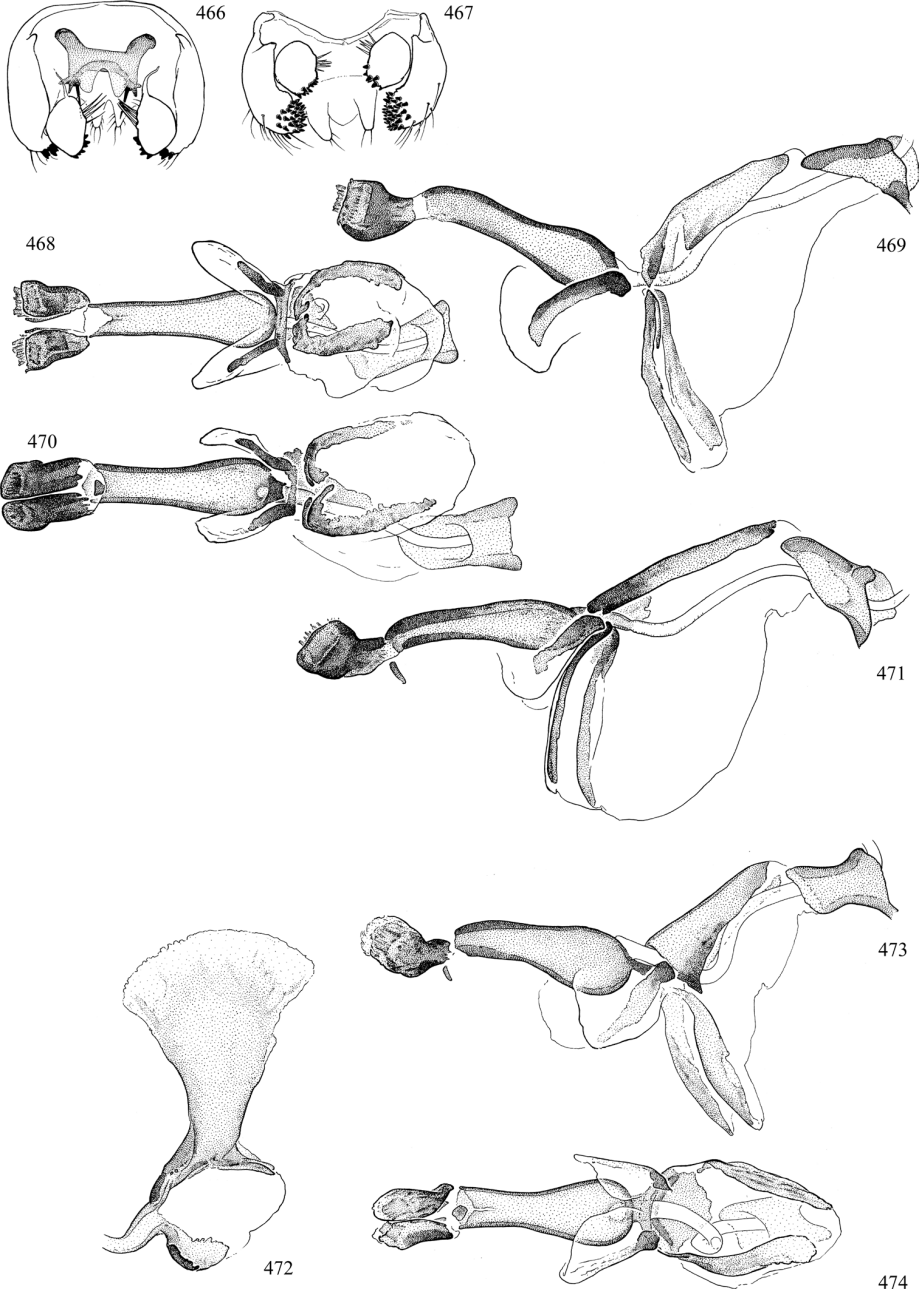
*Calycomyzahumeralis* (Roser), male genitalia **466** external genitalia, anterior **467** external genitalia, ventral **468** phallus, ventral **469** phallus, left lateral **470, 471***C.solidagensis* (Kaltenbach), male genitalia **470** phallus, ventral **471** phallus, left lateral **472–474***C.platyptera* (Thompson), male genitalia **472** ejaculatory apodeme **473** phallus, left lateral **474** phallus, ventral.

**Figures 475–477.**
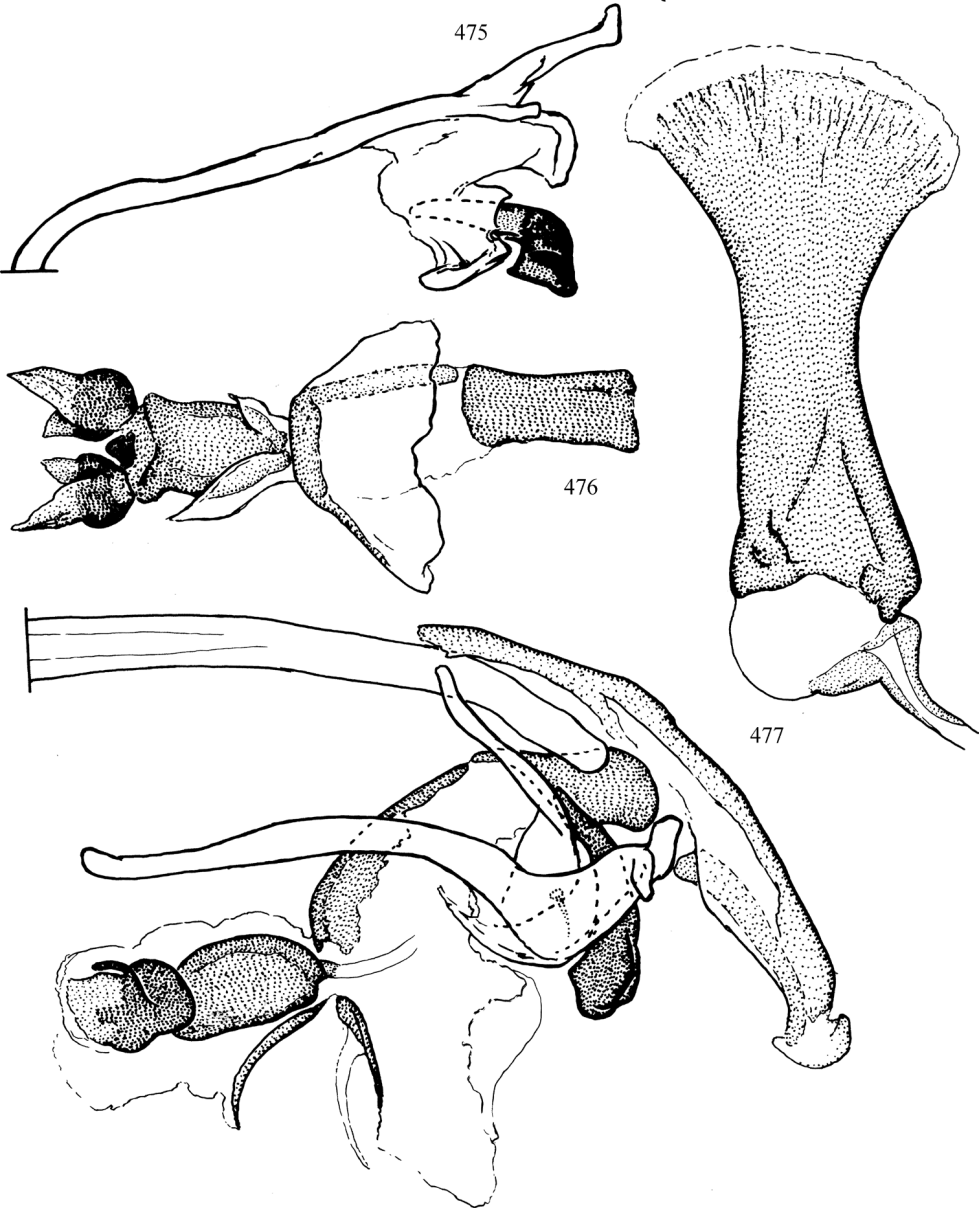
*Calycomyzamalvae* (Burgess), male genitalia **475** hypandrium and postgonite, ventral **476** phallus, ventral **477** hypandrial complex, left lateral.

**Figures 478, 479.**
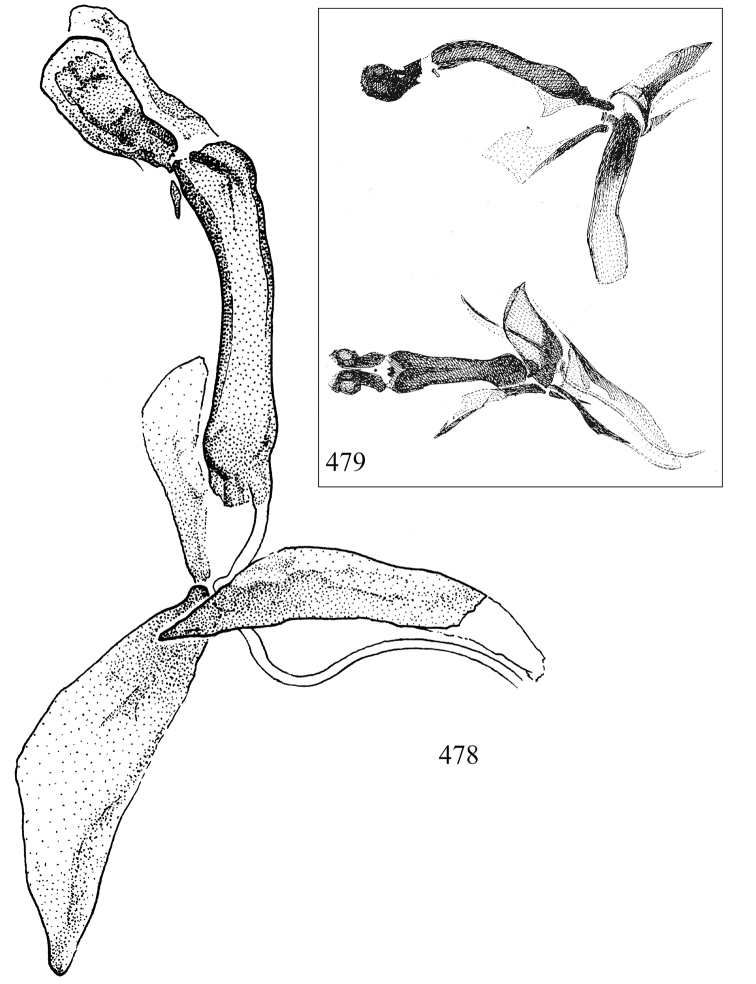
*Calycomyzaorientalis* Spencer, male genitalia **478** paratype, phallus, left lateral **479** holotype, phallus, left lateral (top) and left lateral (bottom) [Originally published in Spencer and Steyskal (1986b)].

**Figures 480–489.**
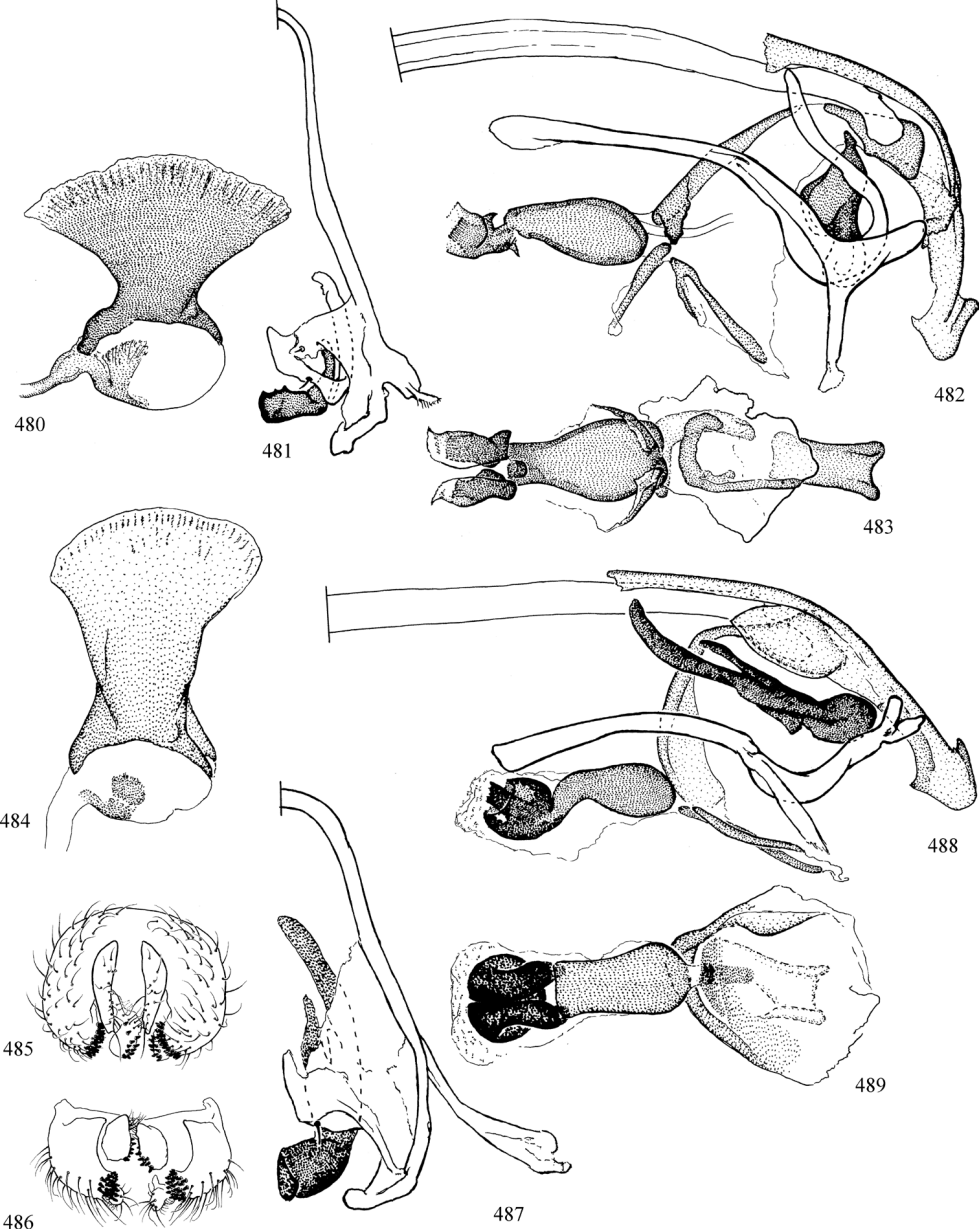
*Calycomyzapromissa* (Frick), male genitalia **480** ejaculatory apodeme **481** hypandrium and postgonite, ventral **482** hypandrial complex, left lateral **483** phallus, ventral **484–489***C.verbenae* (Hering), male genitalia **484** ejaculatory apodeme **485** external genitalia, posterior **486** external genitalia, ventral **487** hypandrium and postgonite, ventral **488** hypandrial complex, left lateral **489** phallus, ventral.

**Figures 490–494.**
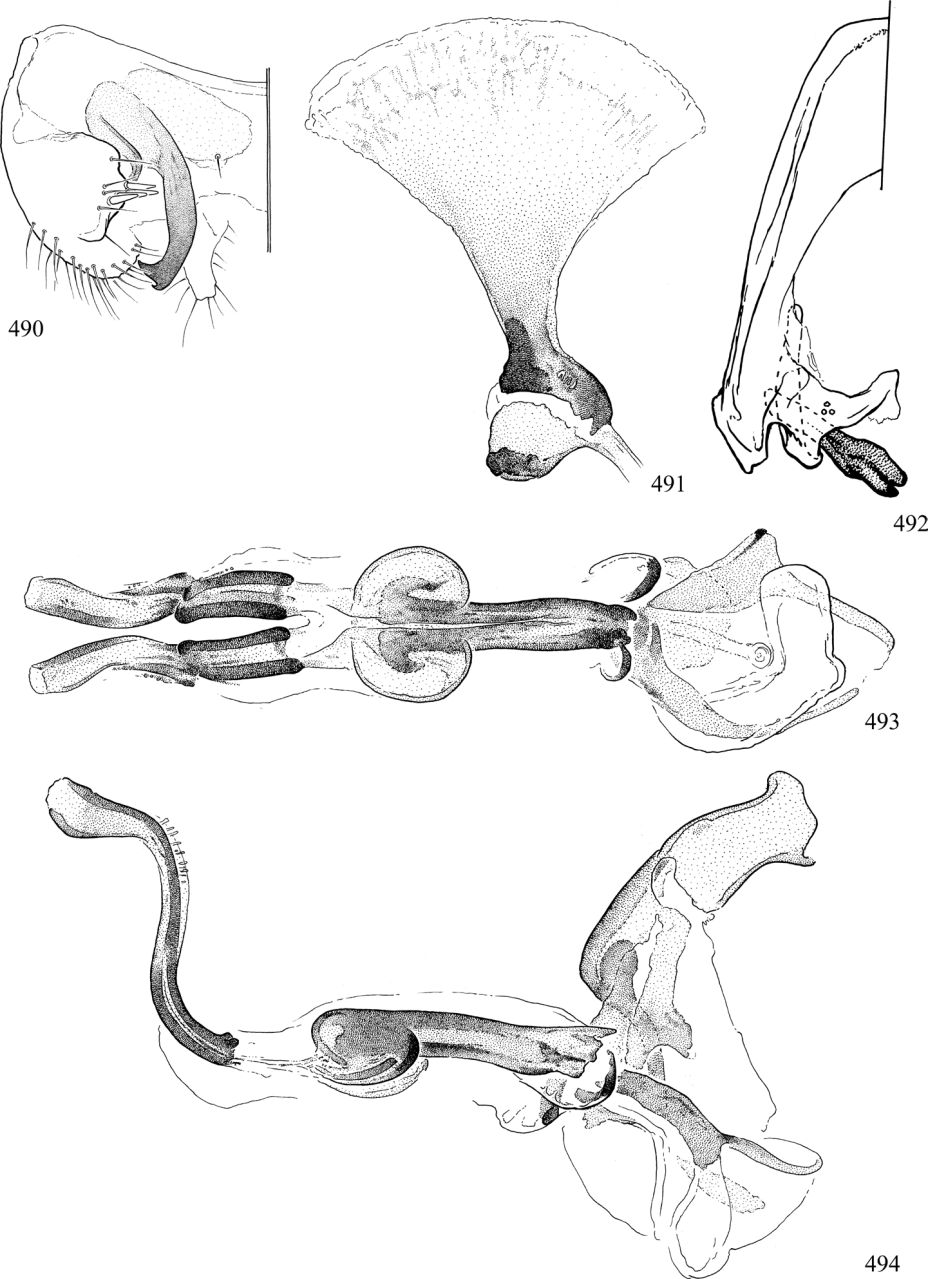
Cerodontha (Butomomyza) angulata (Loew), “typical” male genitalia **490** external genitalia, ventral (subepandrial sclerite shaded) **491** ejaculatory apodeme **492** hypandrium and postgonite, ventral **493** phallus, ventral **494** phallus, left lateral.

**Figures 495–502.**
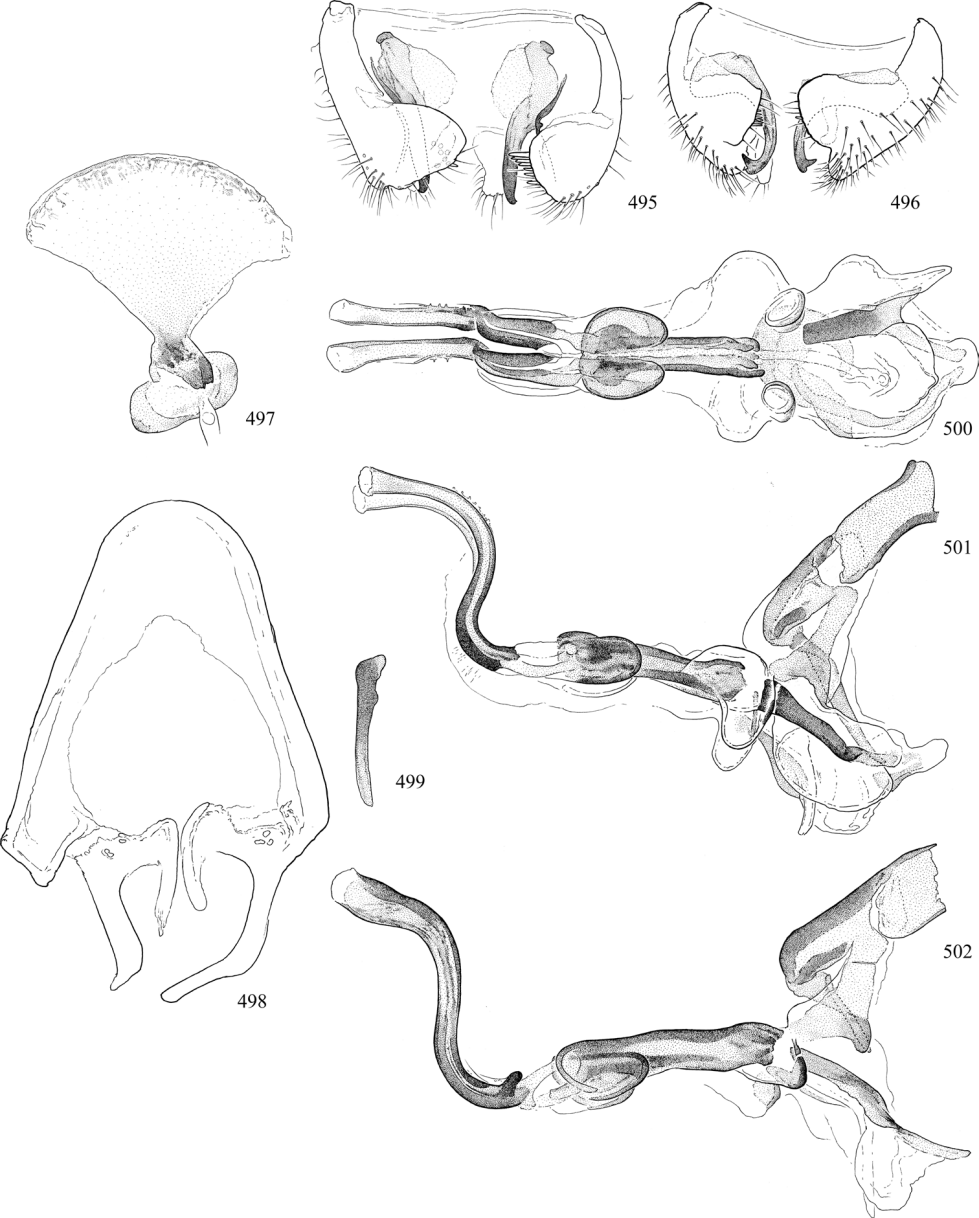
Cerodontha (Butomomyza) angulata (Loew), “atypical” male genitalia **495** external genitalia, ventral (Maryland) **496** external genitalia, ventral (Indian Gap, North Carolina) **497** ejaculatory apodeme (Maryland) **498** hypandrium, ventral (Maryland) **499** postgonite, lateral (Maryland) **500** phallus, ventral (Maryland) **501** phallus, left lateral (Maryland) **502** phallus, left lateral (Indian Gap).

**Figures 503–507.**
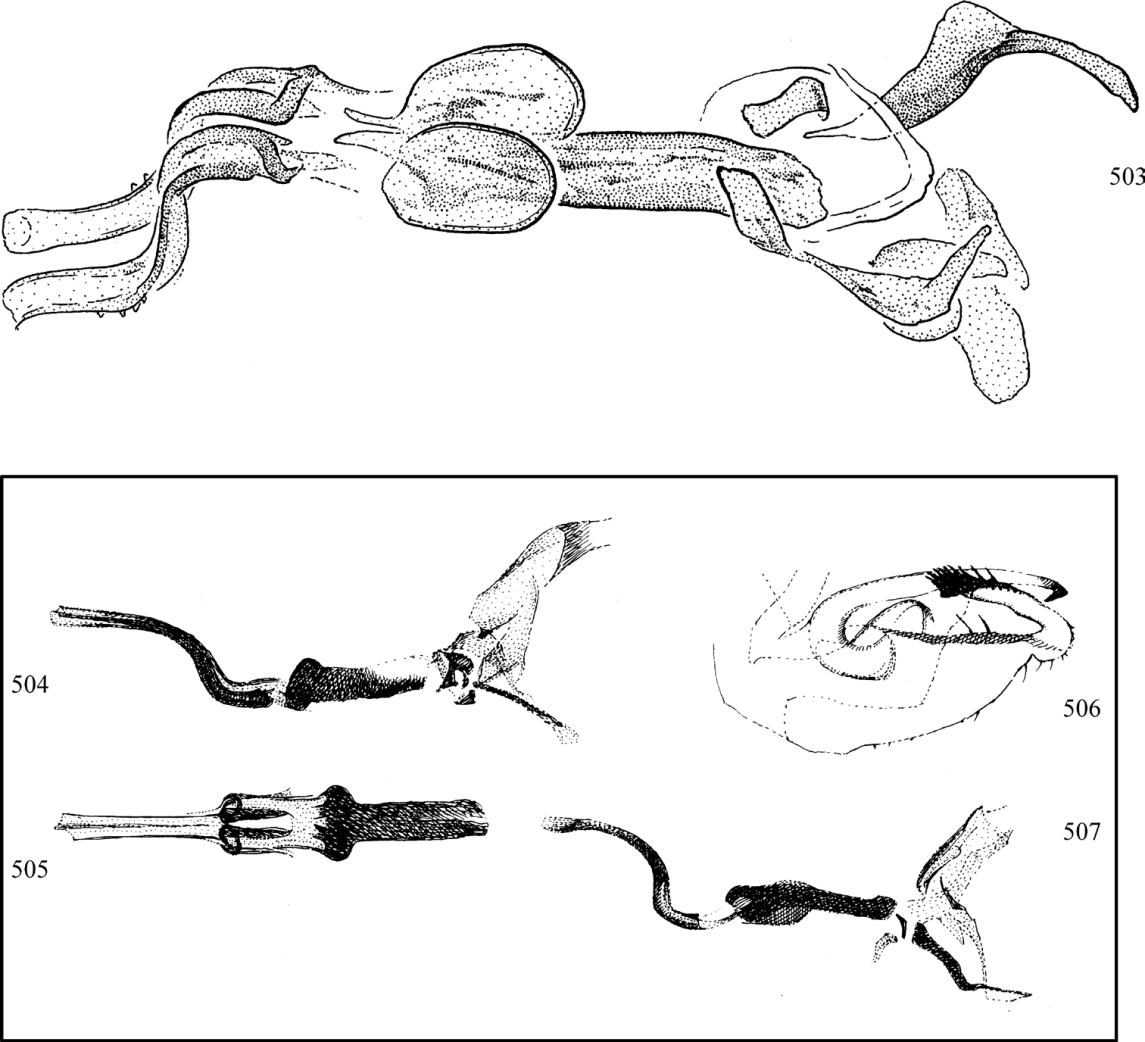
**503**Cerodontha (Butomomyza) subangulata (Malloch), phallus, ventral, non-type from North Carolina **504–507**C. (B.) subangulata, illustrations from Spencer and Steyskal (1986b)**504** holotype phallus, left lateral **505** holotype phallus, ventral **506** holotype external genitalia **507** male from Cabin John Creek (Maryland), phallus, left lateral.

**Figures 508–512.**
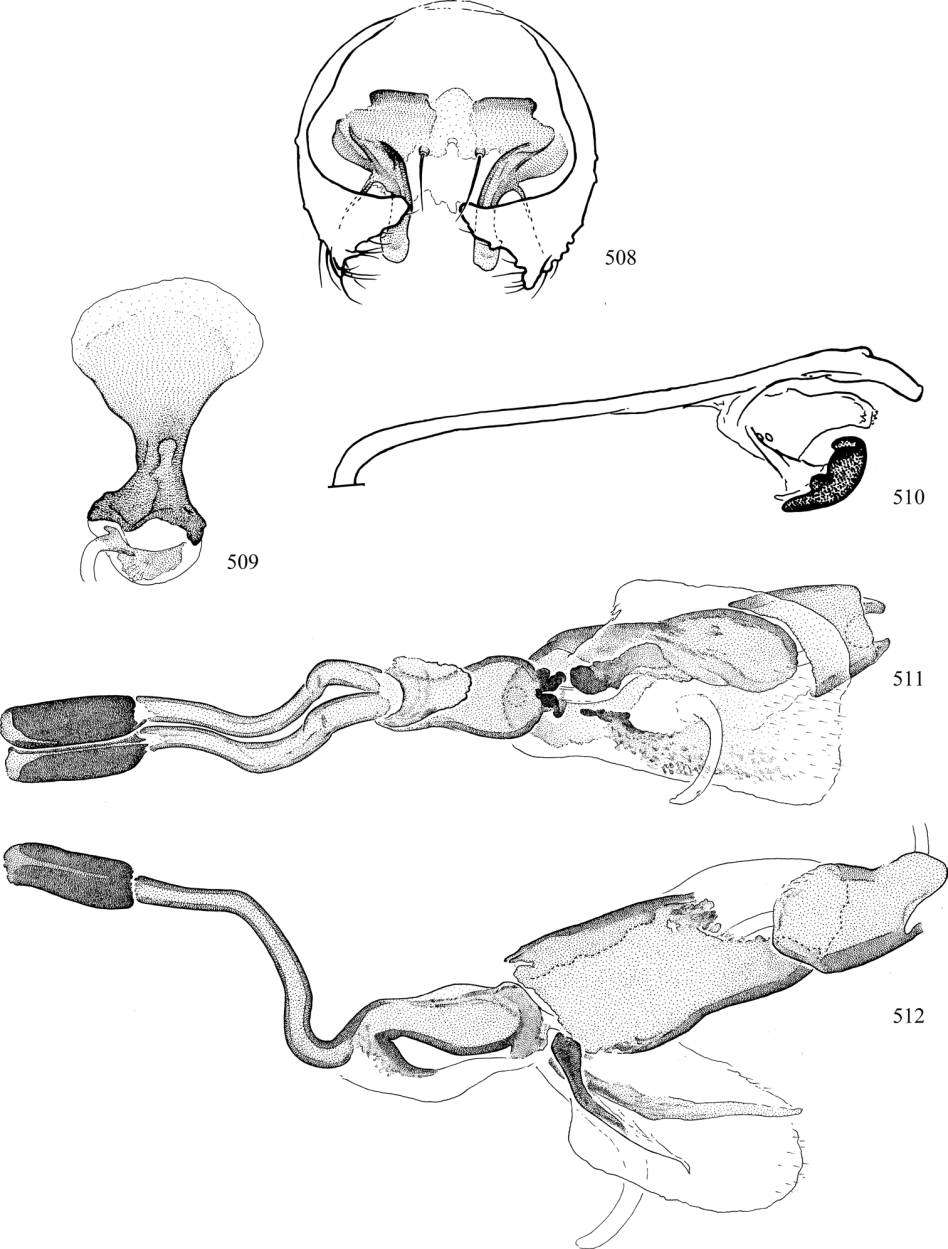
Cerodontha (Cerodontha) dorsalis (Loew), male genitalia **508** external genitalia, anterior **509** ejaculatory apodeme **510** hypandrium and postgonite, ventral **511** phallus, ventral **512** phallus, left lateral.

**Figures 513–518.**
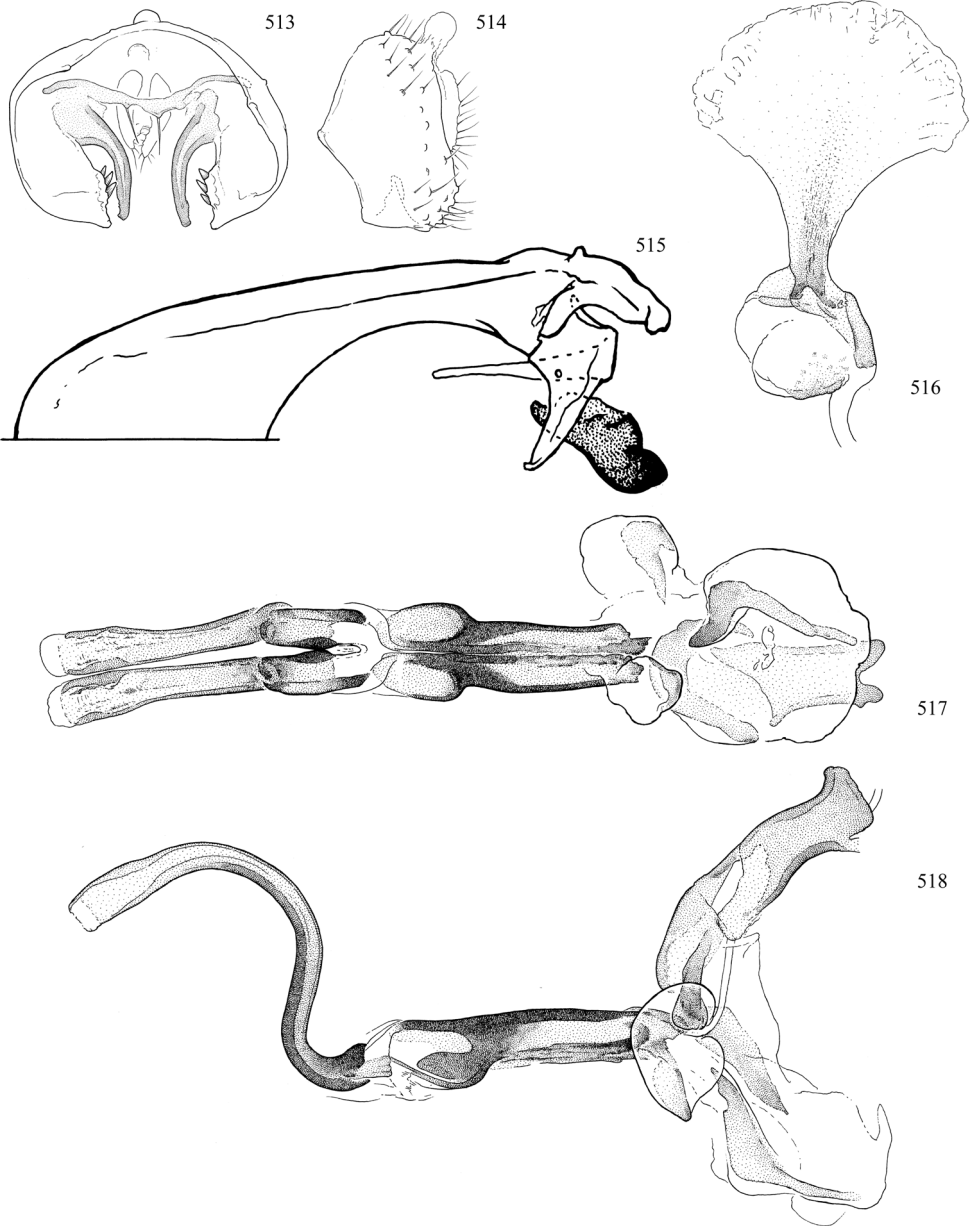
Cerodontha (Dizygomyza) fasciata (Strobl), male genitalia **513** external genitalia, anterior **514** external genitalia, left lateral **515** hypandrium and postgonite, ventral **516** ejaculatory apodeme **517** phallus, ventral **518** phallus, left lateral.

**Figures 519–524.**
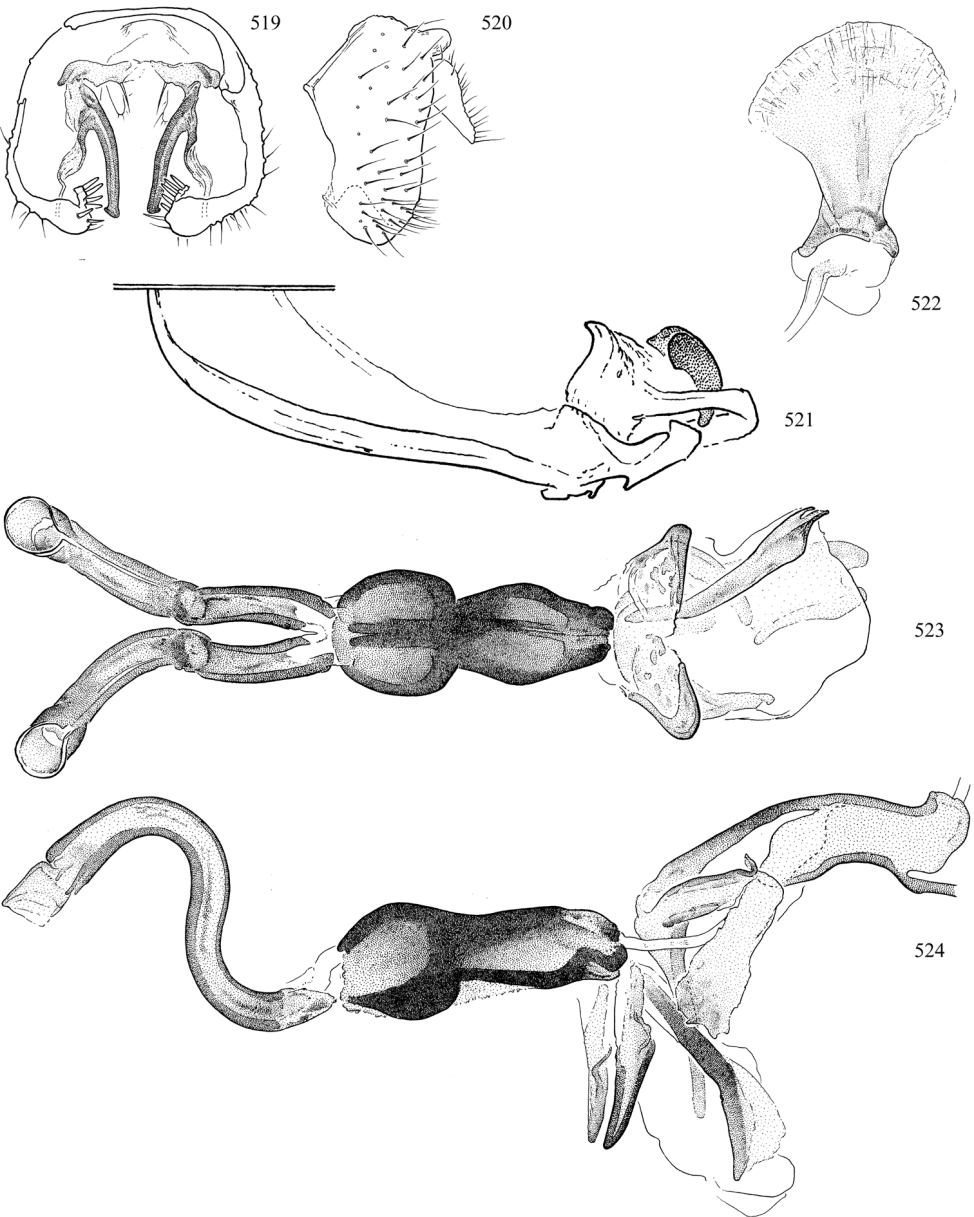
Cerodontha (Dizygomyza) maclayi Spencer, male genitalia **519** external genitalia, anterior **520** external genitalia, left lateral **521** hypandrium and postgonite, ventral **522** ejaculatory apodeme **523** phallus, ventral **524** phallus, left lateral.

**Figures 525–528.**
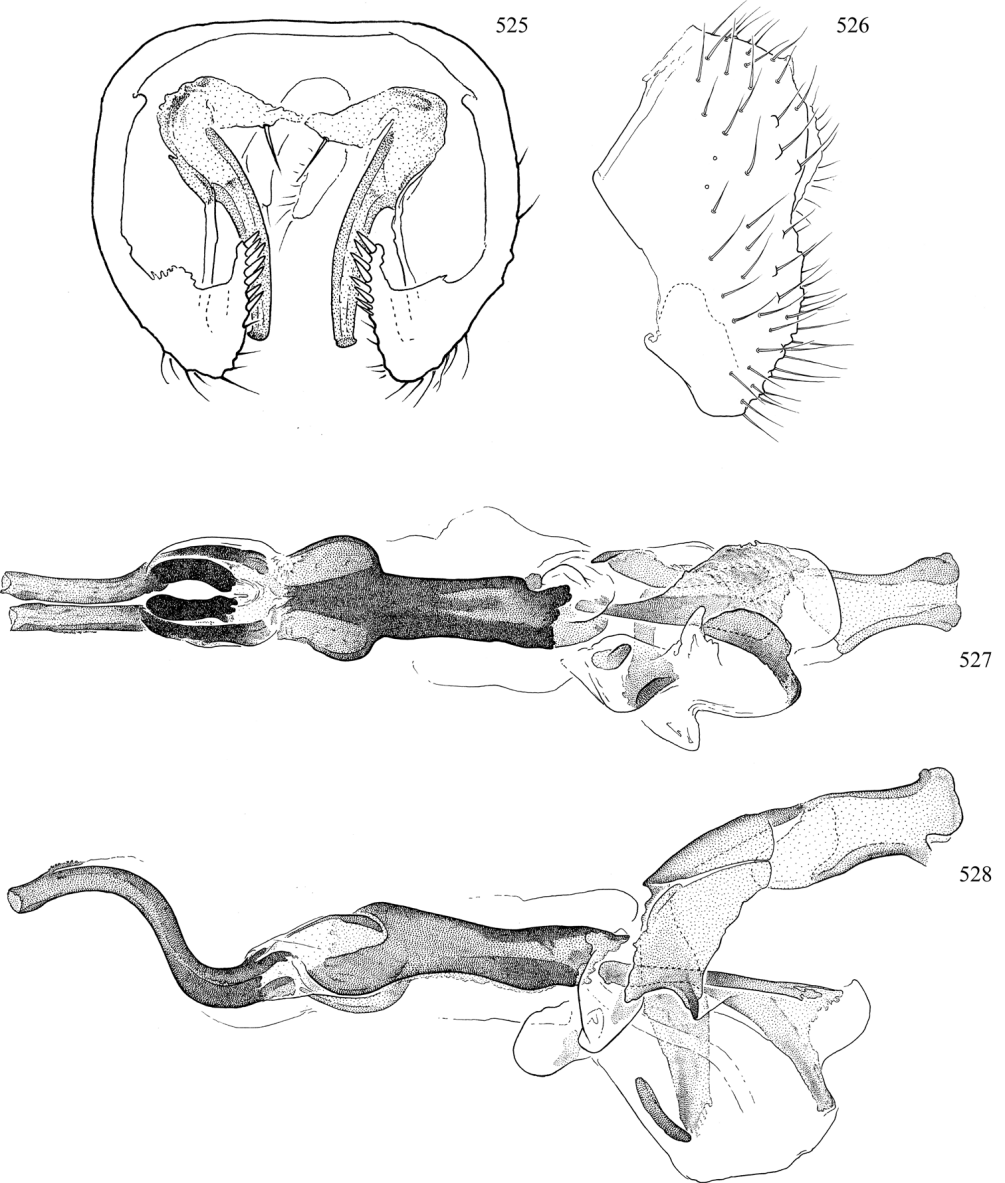
Cerodontha (Dizygomyza) magnicornis (Loew), male genitalia **525** external genitalia, anterior **526** external genitalia, left lateral **527** phallus, ventral **528** phallus, left lateral.

**Figures 529–534.**
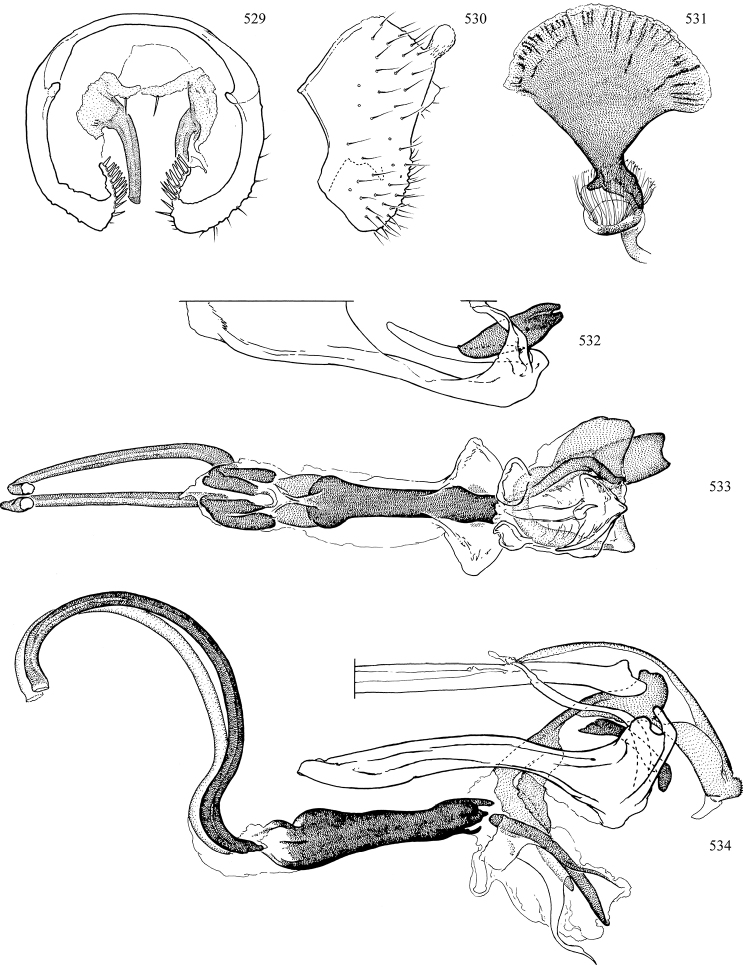
Cerodontha (Dizygomyza) morosa (Meigen), male genitalia **529** external genitalia, anterior **530** external genitalia, left lateral **531** ejaculatory apodeme **532** hypandrium and postgonite, ventral **533** phallus, ventral **534** phallus, left lateral.

**Figures 535–540.**
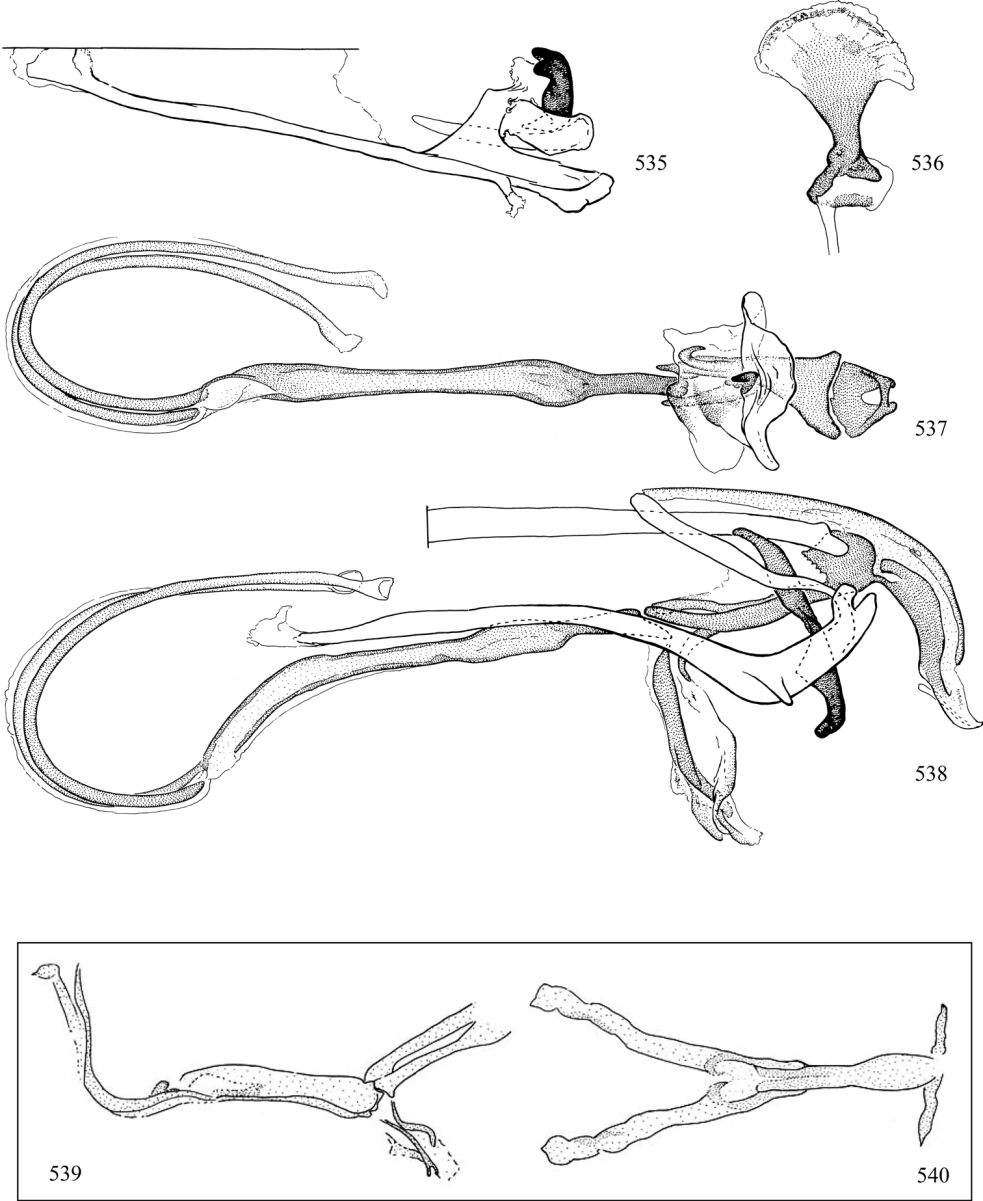
Cerodontha (Icteromyza) longipennis (Loew), male genitalia **535** hypandrium and postgonite, ventral **536** ejaculatory apodeme **537** phallus, ventral **538** phallus, left lateral **539, 540**C. (I.) vockerothi Boucher (illustrations from Boucher 2012) **539** apex of phallus, left lateral **540** same, ventral.

**Figures 541–544.**
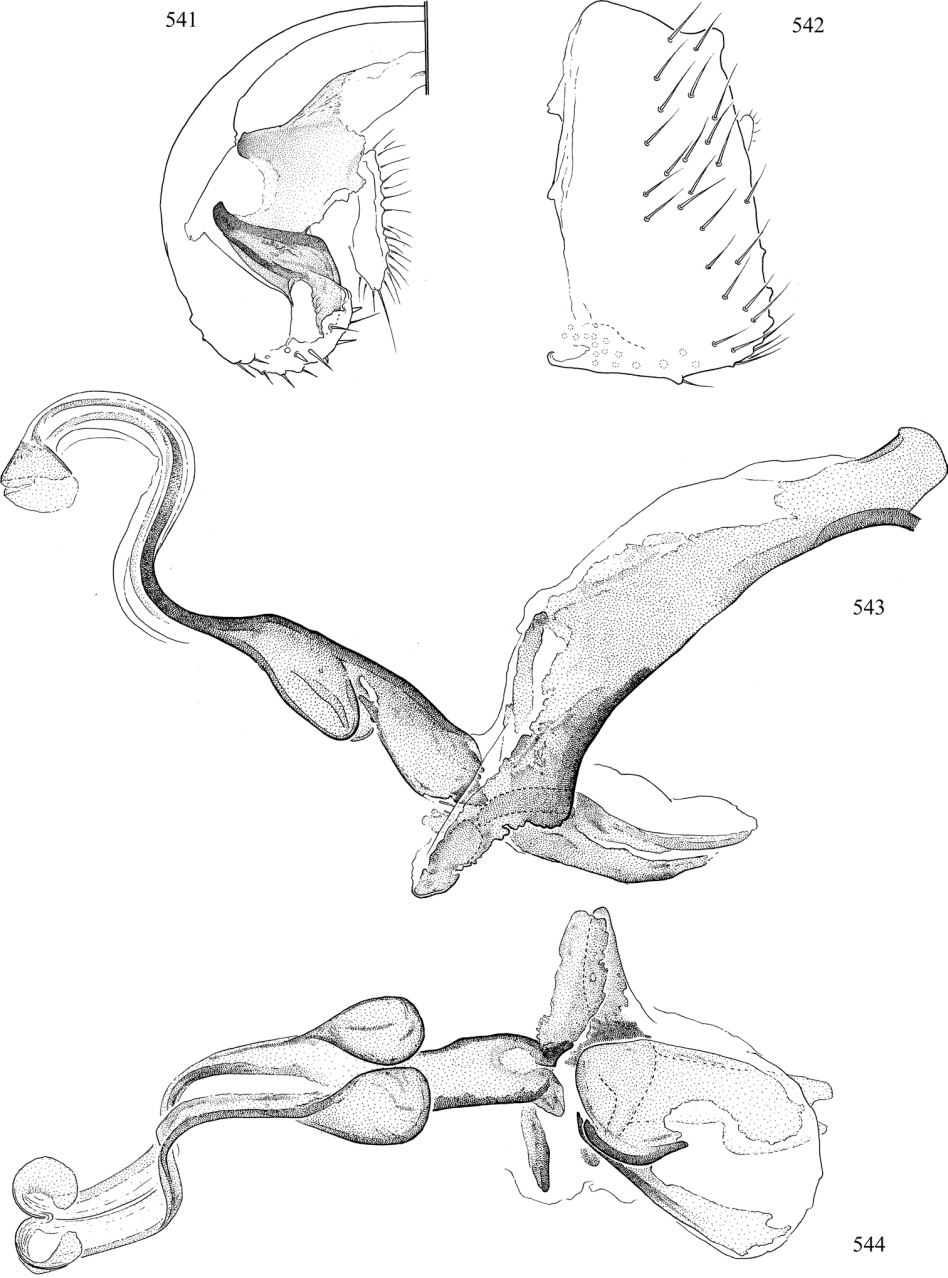
Cerodontha (Poemyza) incisa (Meigen), male genitalia **541** external genitalia, anterior **542** external genitalia, left lateral **543** phallus, left lateral **544** phallus, ventral.

**Figures 545–549.**
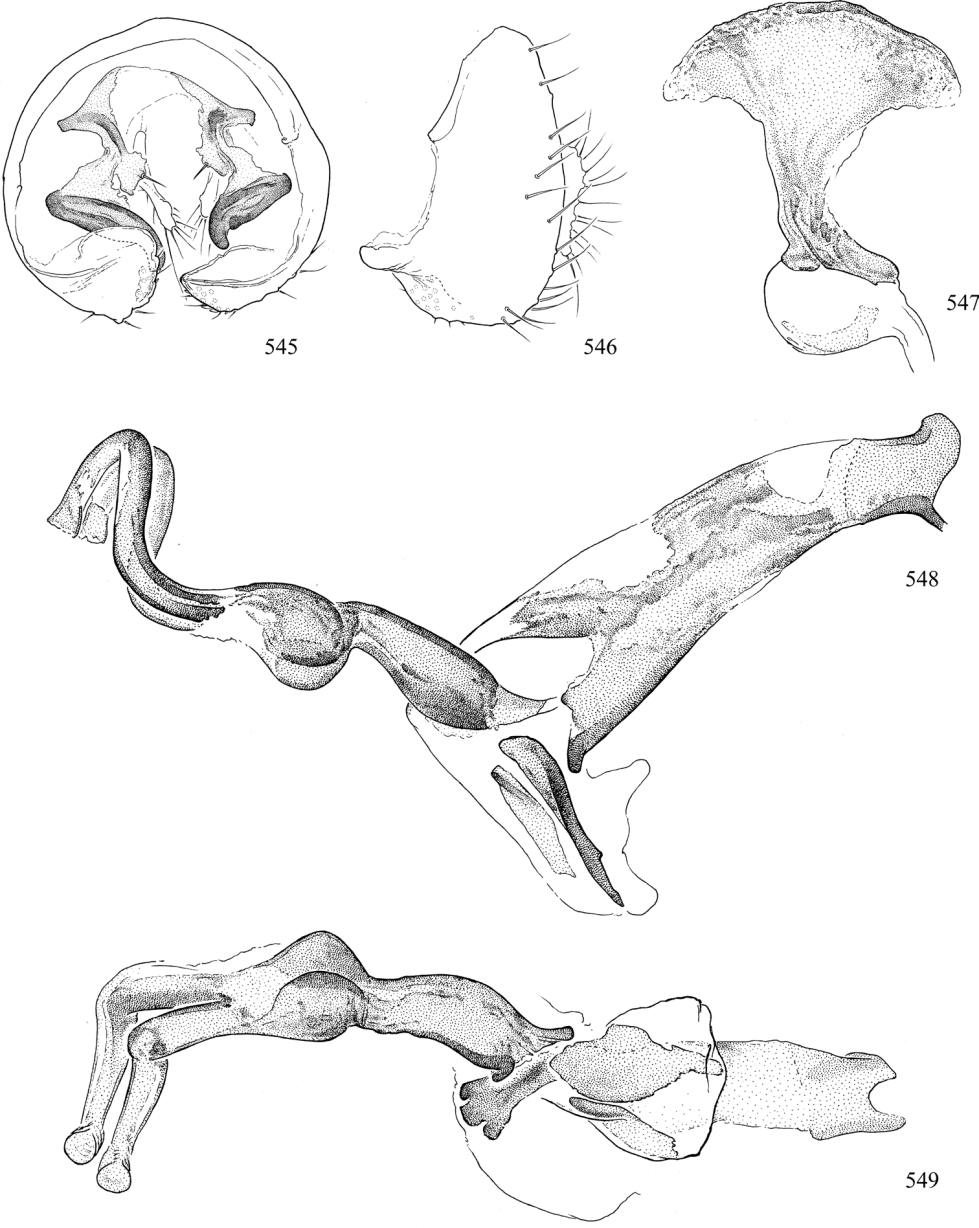
Cerodontha (Poemyza) muscina (Meigen), male genitalia **545** external genitalia, anterior **546** external genitalia, left lateral **547** ejaculatory apodeme **548** phallus, left lateral **549** phallus, ventral.

**Figures 550–554.**
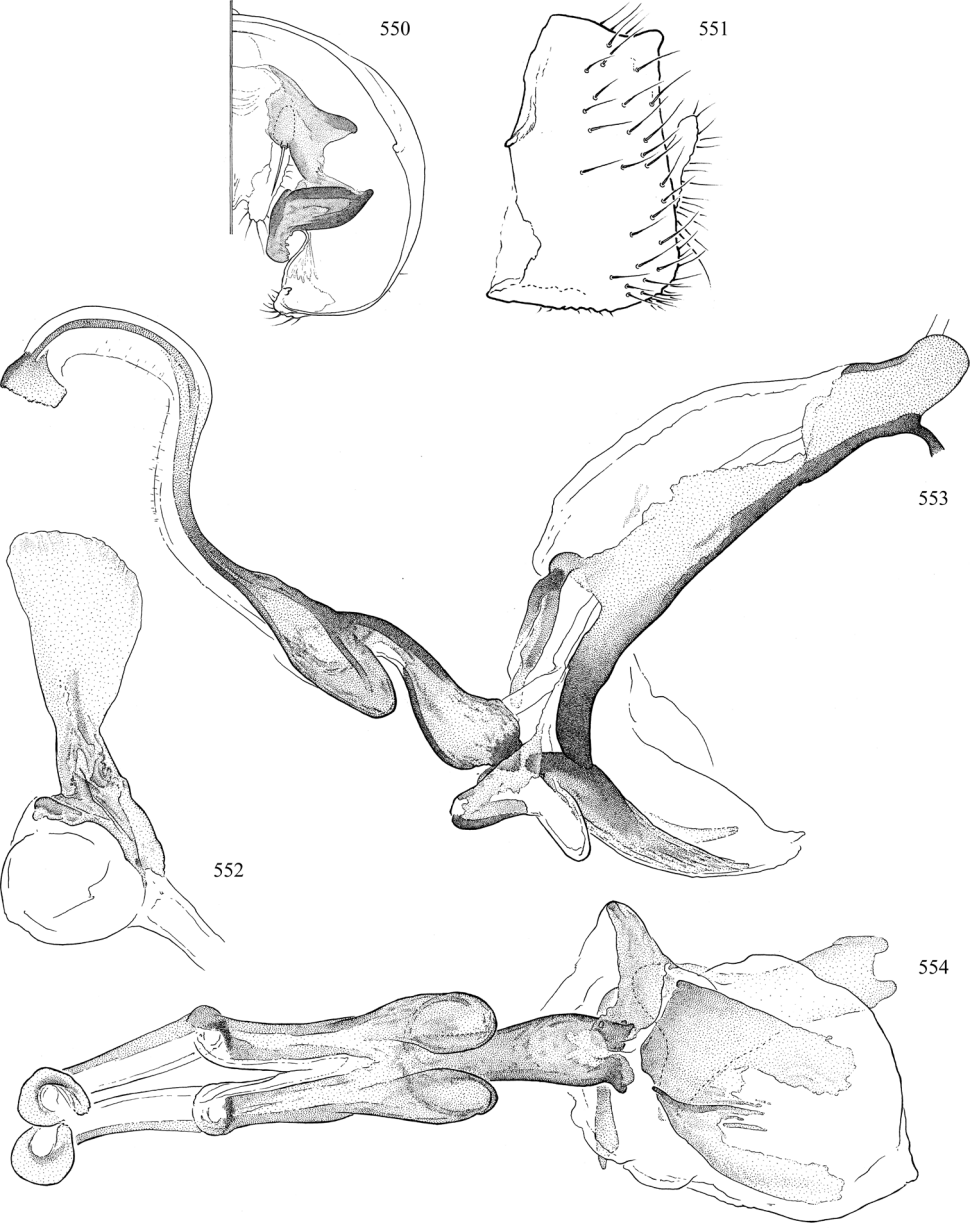
Cerodontha (Poemyza) pygmaea (Meigen), male genitalia **550** external genitalia, anterior **551** external genitalia, left lateral **552** ejaculatory apodeme **553** phallus, left lateral **554** phallus, ventral.

**Figures 555–558.**
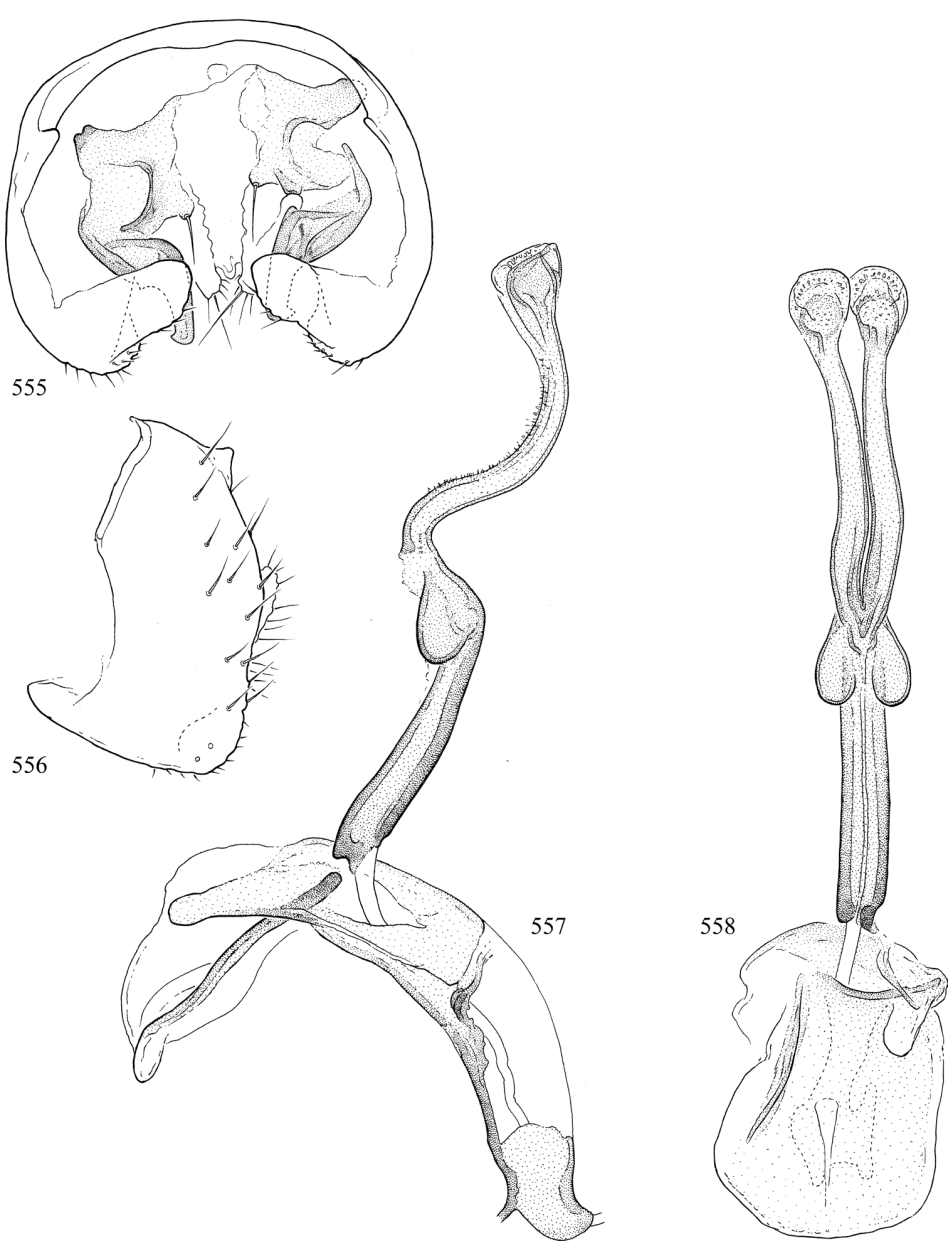
Cerodontha (Poemyza) pygminoides Spencer, male genitalia **555** external genitalia, anterior **556** external genitalia, left lateral **557** phallus, left lateral **558** phallus, ventral.

**Figures 559–563.**
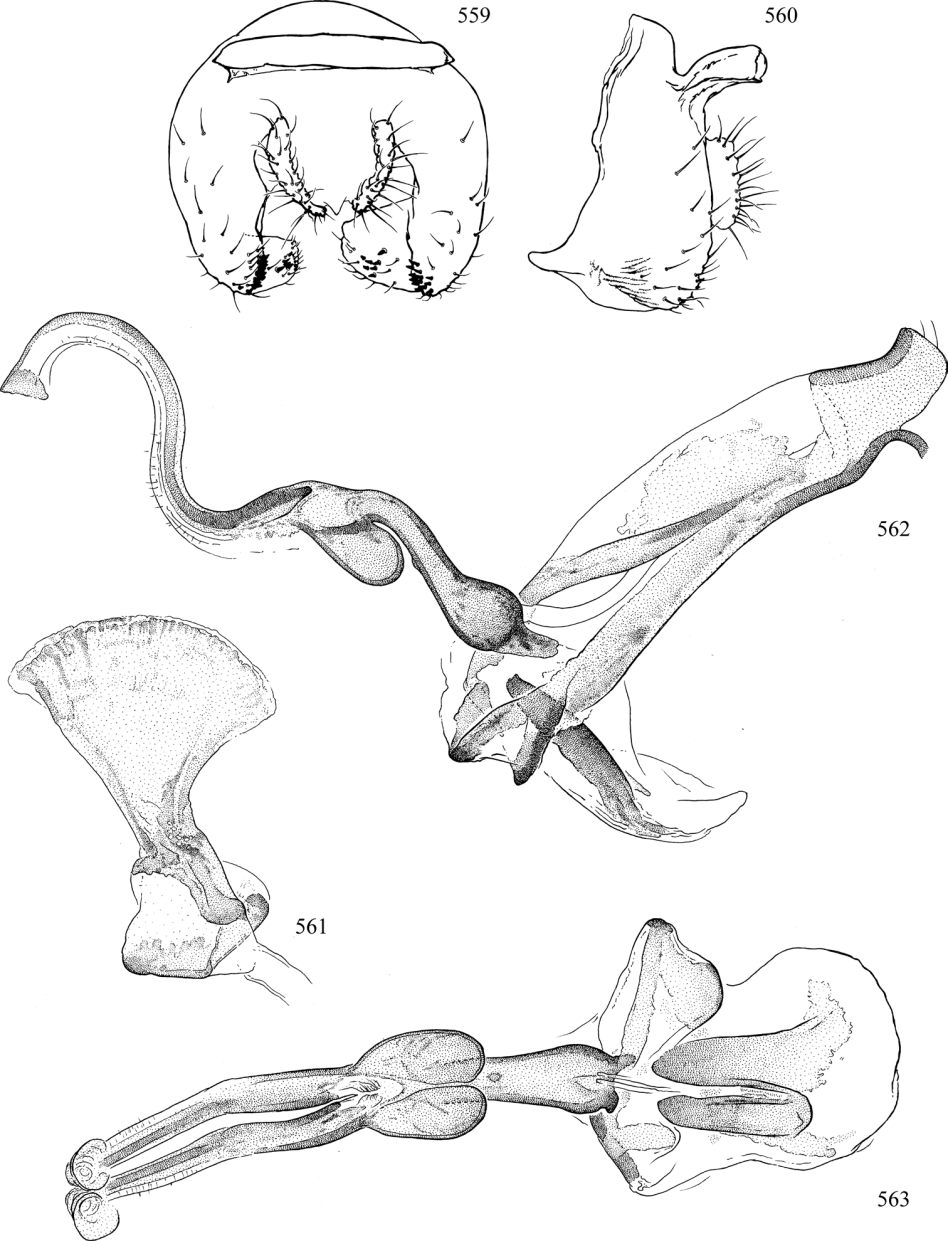
Cerodontha (Poemyza) superciliosa (Zetterstedt), male genitalia **559** external genitalia, posterior **560** external genitalia, left lateral **561** ejaculatory apodeme **562** phallus, left lateral **563** phallus, ventral.

**Figures 564–566.**
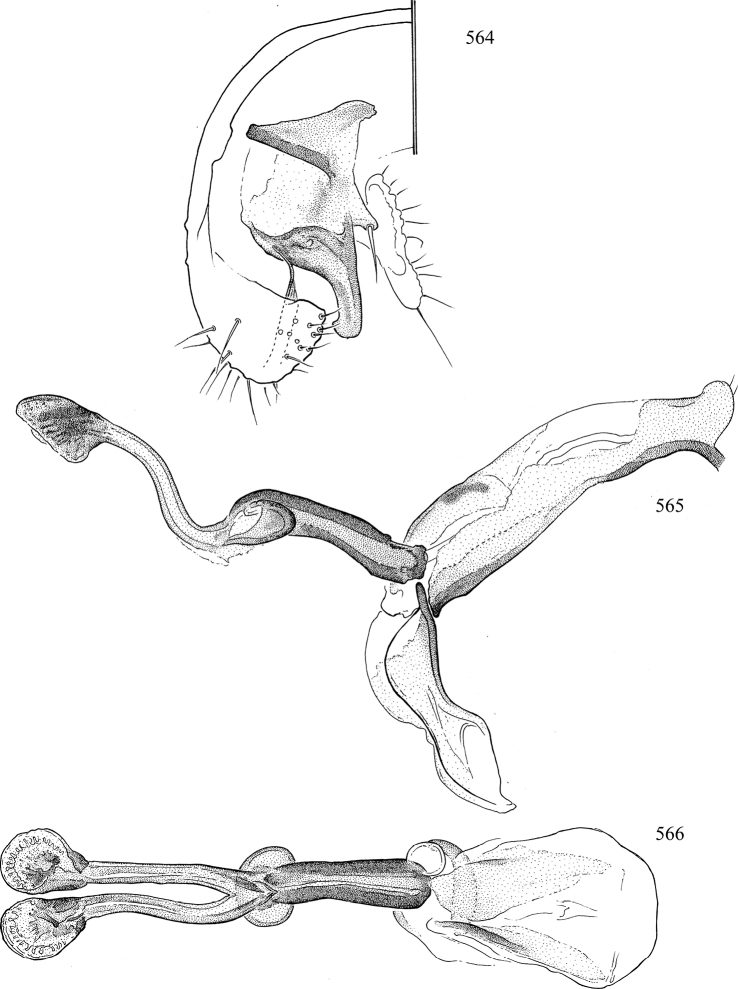
Cerodontha (Poemyza) ungula sp. nov., male genitalia **564** external genitalia, posterior **565** phallus, left lateral **566** phallus, ventral.

**Figures 567–573.**
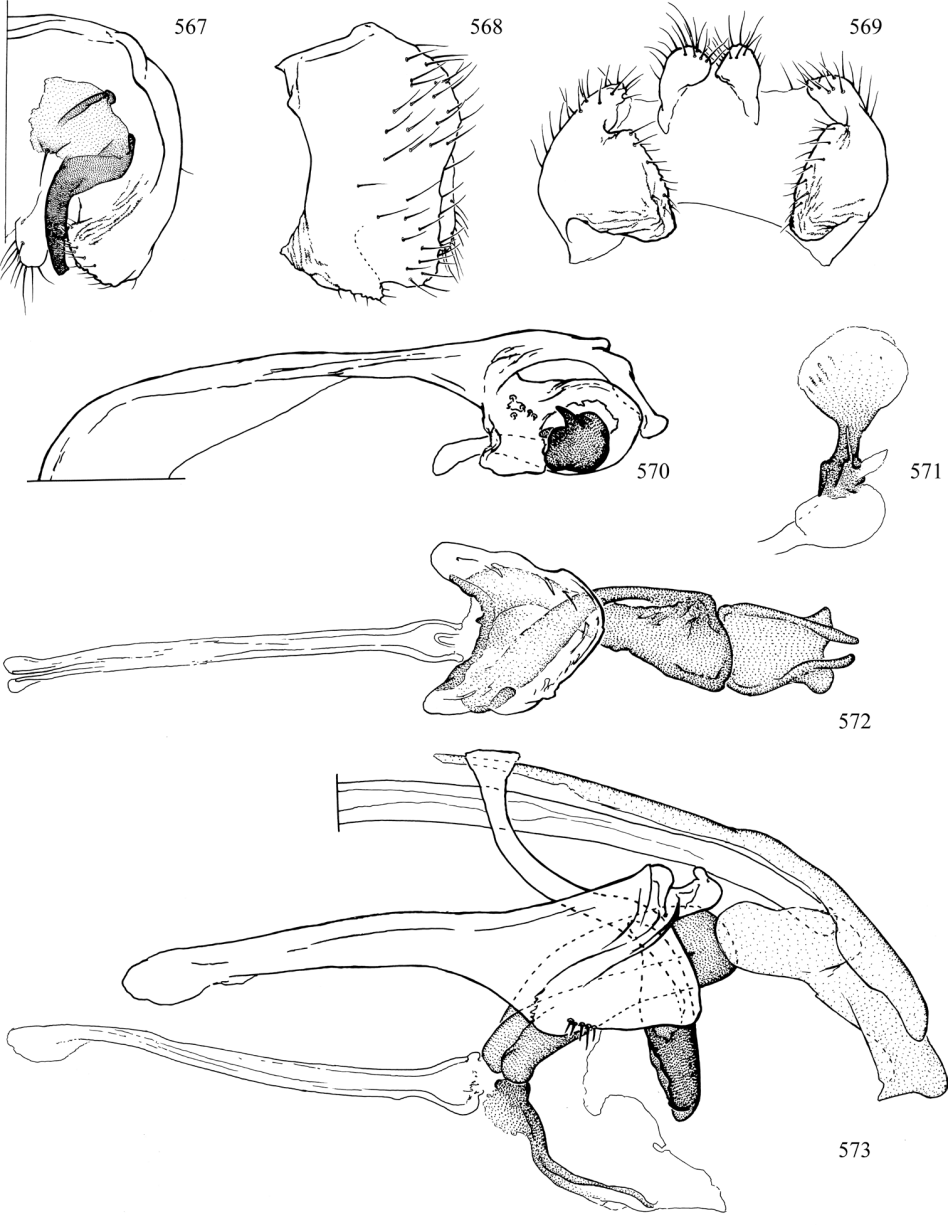
Cerodontha (Xenophytomyza) illinoensis (Malloch), male genitalia **567** external genitalia, anterior **568** external genitalia, left lateral **569** external genitalia, ventral **570** hypandrium and postgonite, ventral **571** ejaculatory apodeme **572** phallus, ventral **573** phallus, left lateral.

**Figures 574–581.**
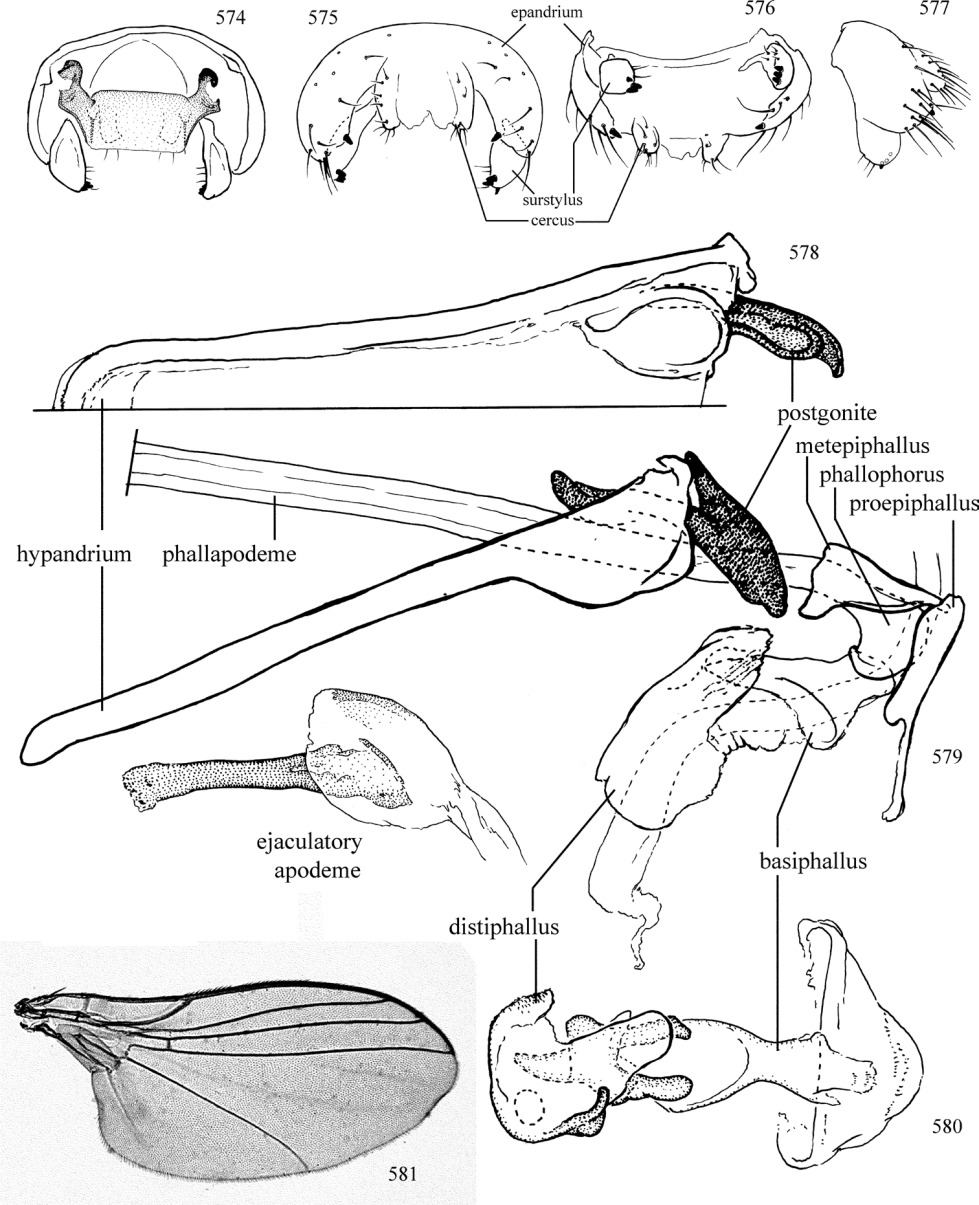
*Haplopeodesminutus* (Frost), male genitalia **574** external genitalia, anterior **575** external genitalia, posterior **576** external genitalia, ventral **577** external genitalia, left lateral **578** hypandrium and postgonite, ventral **579** hypandrial complex, left lateral **580** phallus, ventral **581***H.minutus*, wing.

**Figures 582–587.**
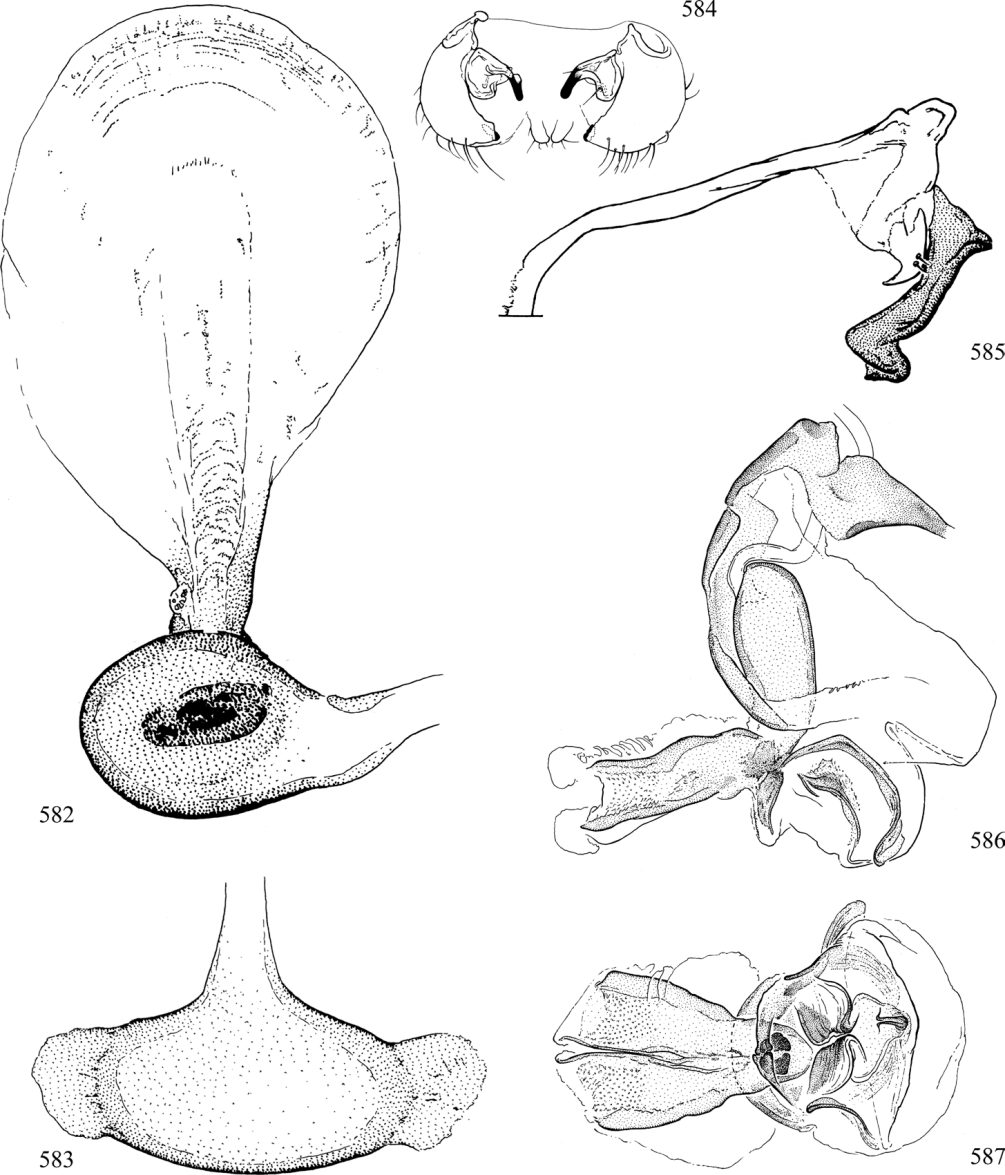
*Liriomyzablechi* Spencer, male genitalia **582** ejaculatory apodeme **583** detail of sperm pump, rotated 90° **584** external genitalia, ventral **585** hypandrium and postgonite, ventral **586** phallus, left lateral **587** phallus, ventral. Originally published in part in Lonsdale (2017).

**Figures 588–594.**
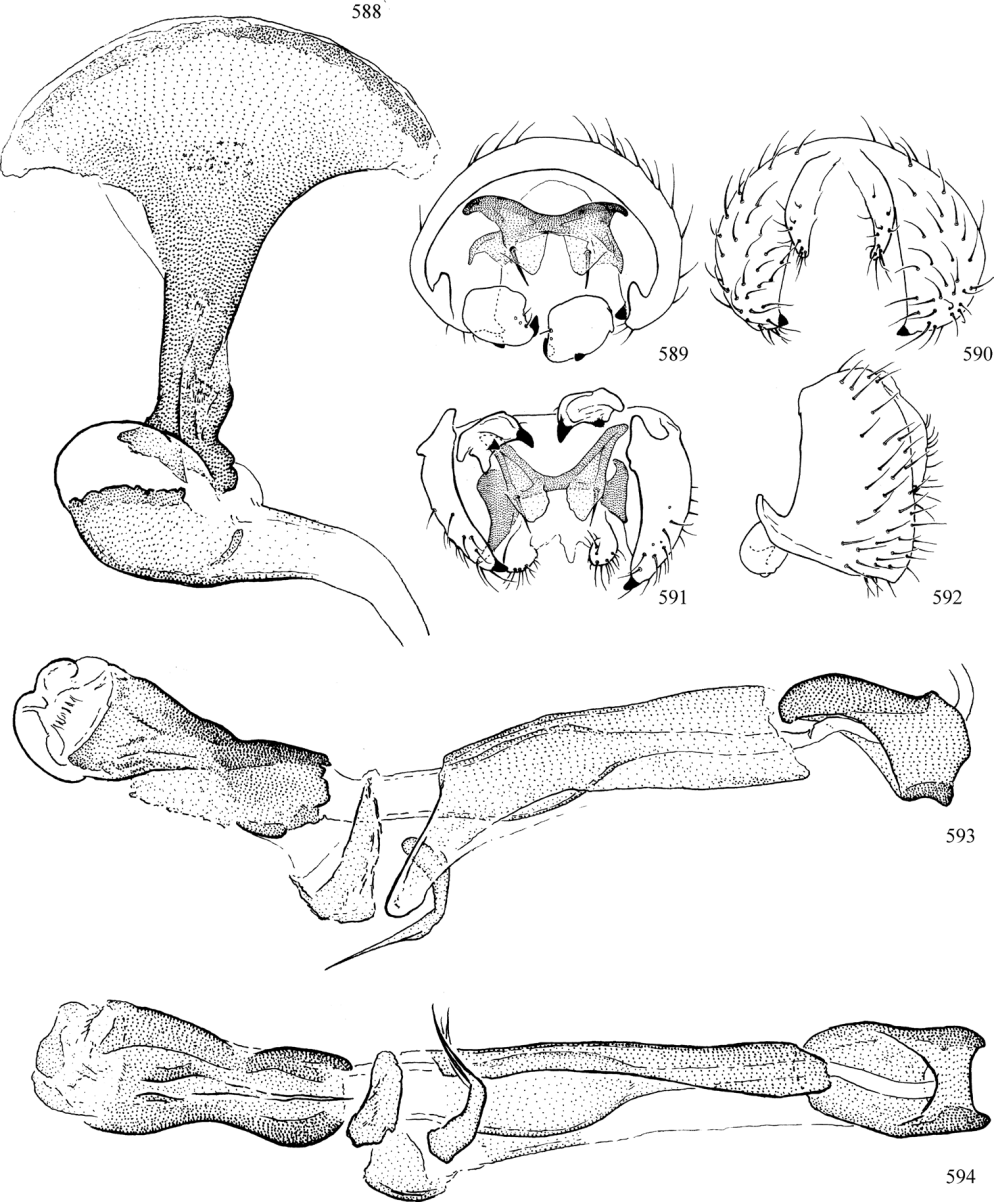
*Liriomyzabrassicae* (Riley), male genitalia **588** ejaculatory apodeme **589** external genitalia, anterior (subepandrial sclerite shaded) **590** external genitalia, posterior **591** external genitalia, ventral **592** external genitalia, left lateral **593** phallus, left lateral **594** phallus, ventral. Originally published in Lonsdale (2011).

**Figures 595–598.**
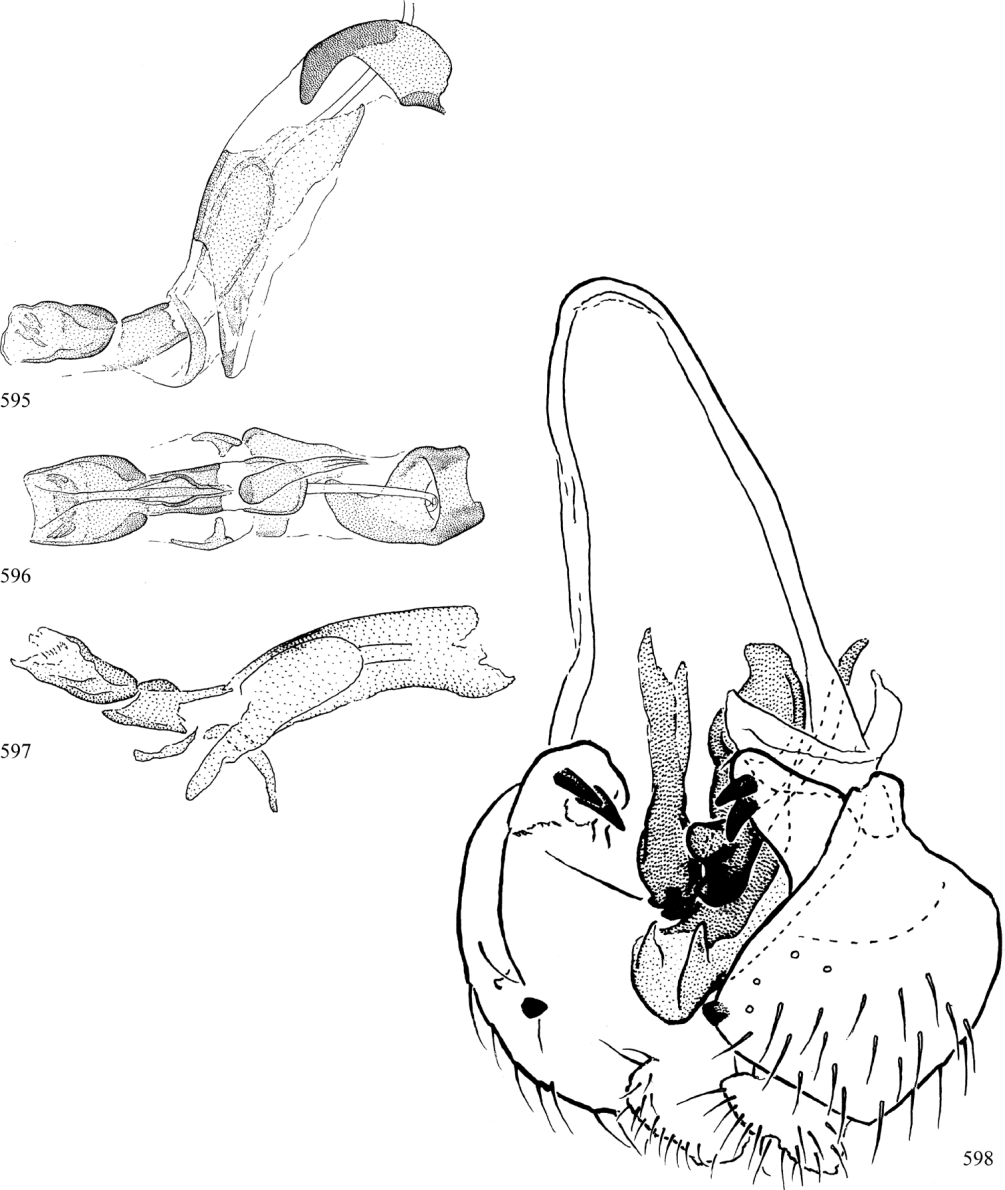
*Liriomyzaeupatoriella* Spencer **595** phallus, left lateral, Iowa male **596** same, ventral **597** phallus, left lateral, male holotype **598** same, remainder of genitalia.

**Figures 599–607.**
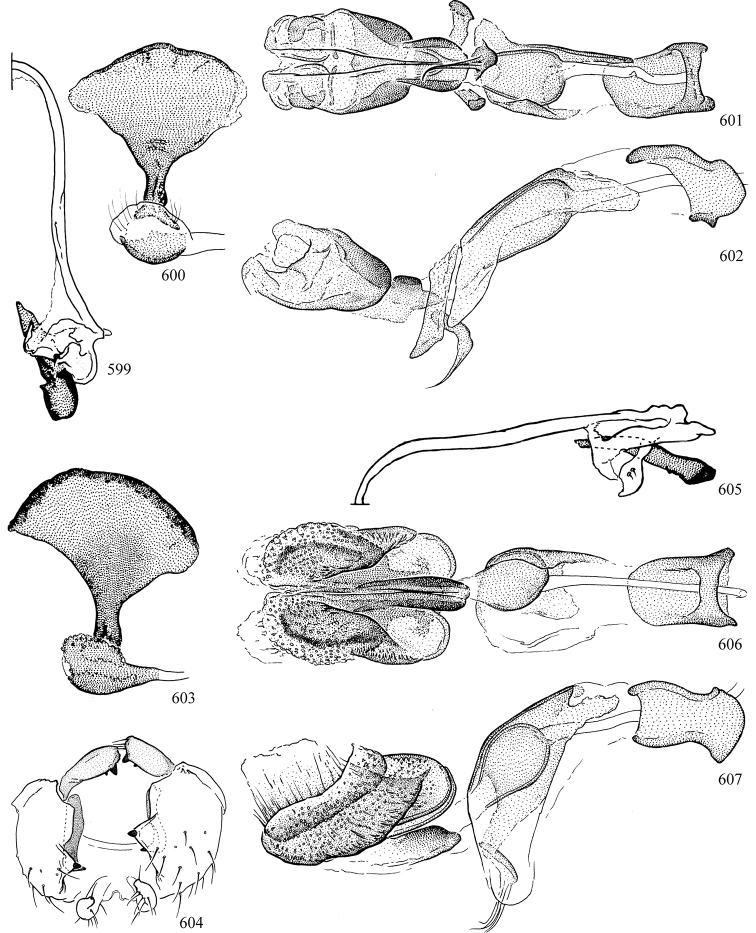
*Liriomyzaeupatorii* (Kaltenbach), male genitalia **599** hypandrium and postgonite, ventral **600** ejaculatory apodeme **601** phallus, ventral **602** phallus, left lateral. Originally published in part in Lonsdale (2011)**603–607***L.fricki* Spencer, male genitalia **603** ejaculatory apodeme **604** external genitalia, ventral **605** hypandrium and postgonite, ventral **606** phallus, ventral **607** phallus, left lateral. Originally published in part in Lonsdale (2017).

**Figures 608–611.**
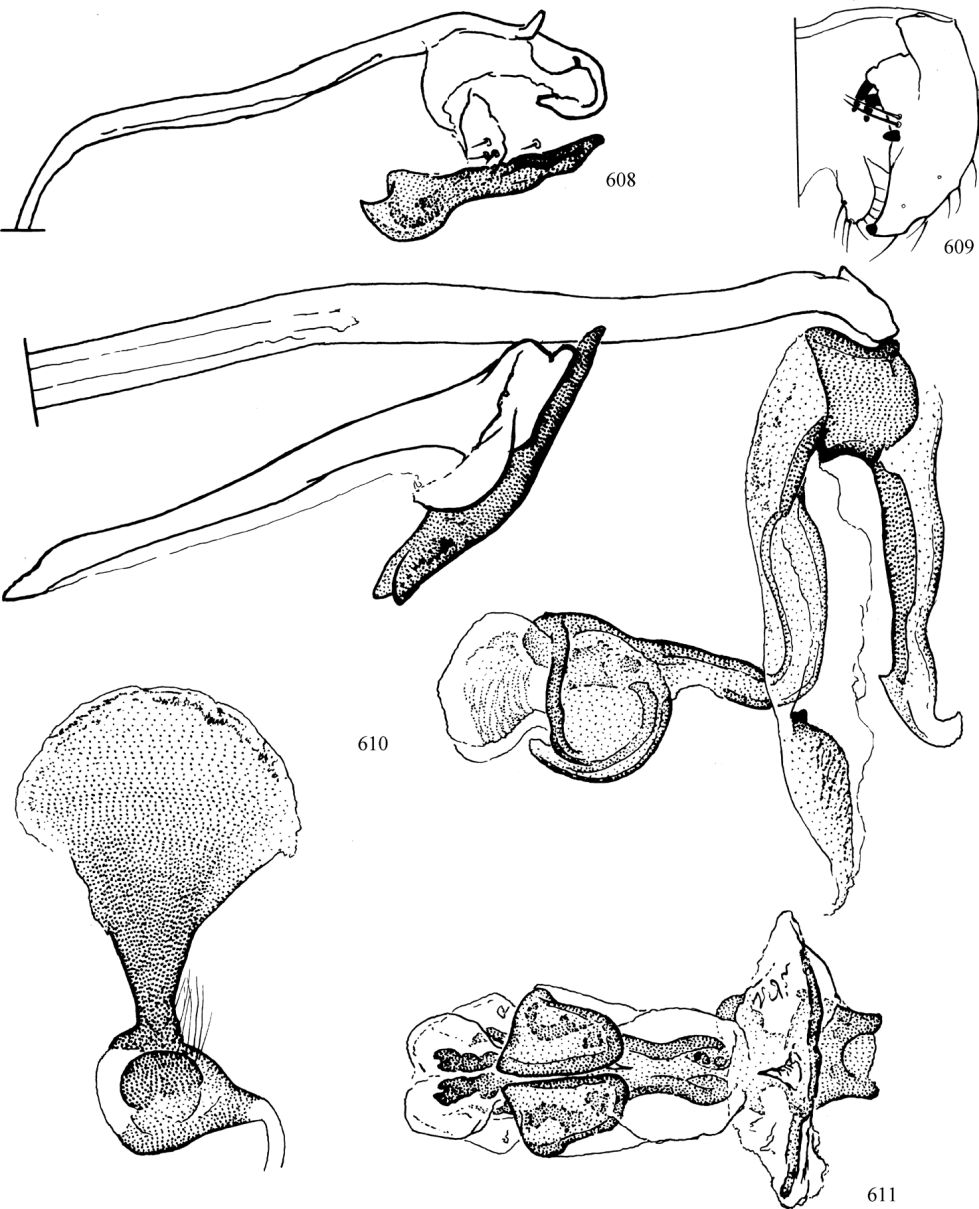
*Liriomyzagaliivora* (Spencer), male genitalia **608** hypandrium and postgonite, ventral **609** external genitalia, ventral **610** hypandrial complex, left lateral **611** phallus, ventral. Originally published in part in Lonsdale (2017).

**Figures 612–615.**
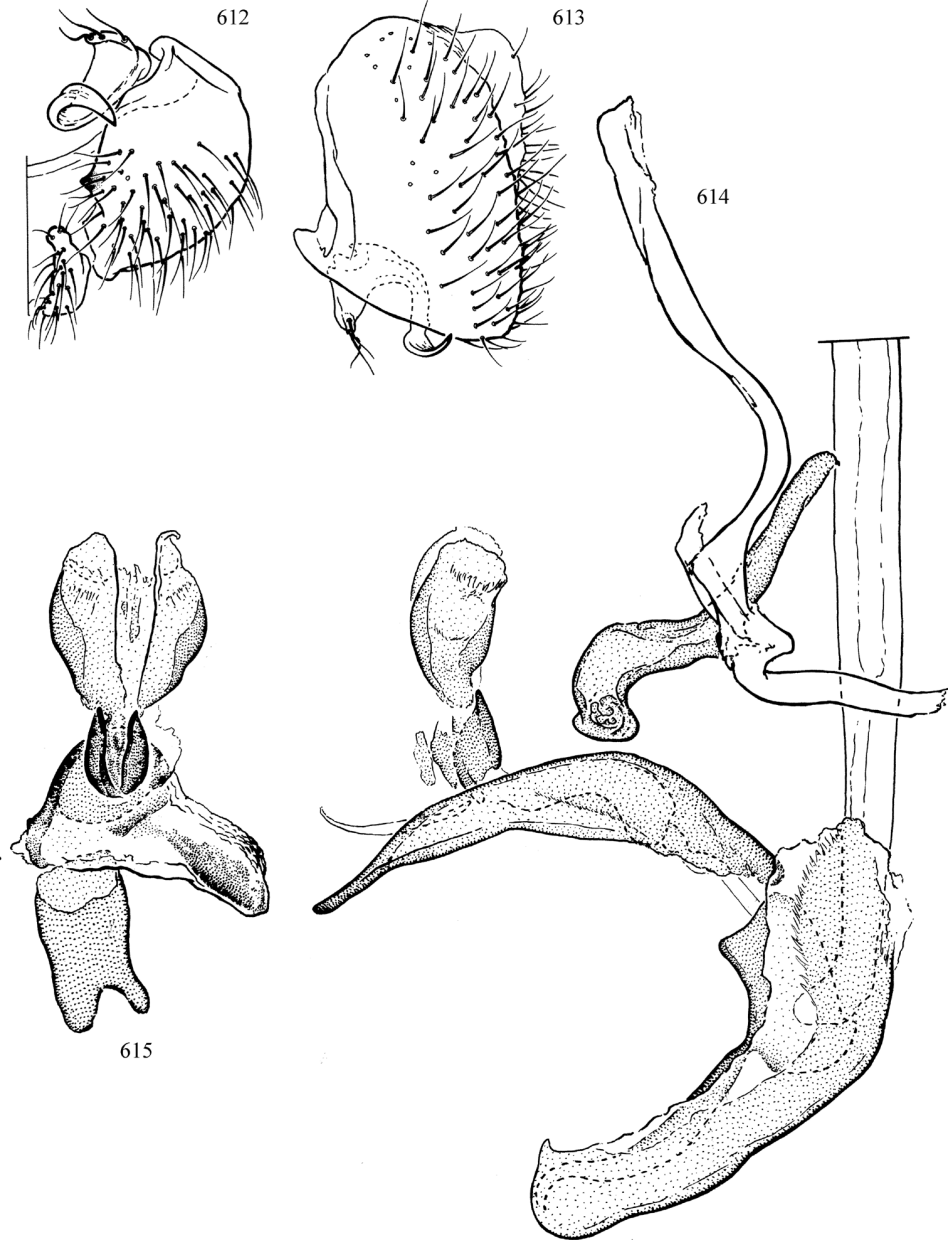
*Liriomyzaphiladelphivora* (Kaltenbach), male genitalia **612** external genitalia, ventral **613** external genitalia, left lateral **614** hypandrial complex, left lateral **615** phallus, ventral. Originally published in Lonsdale (2017).

**Figures 616–620.**
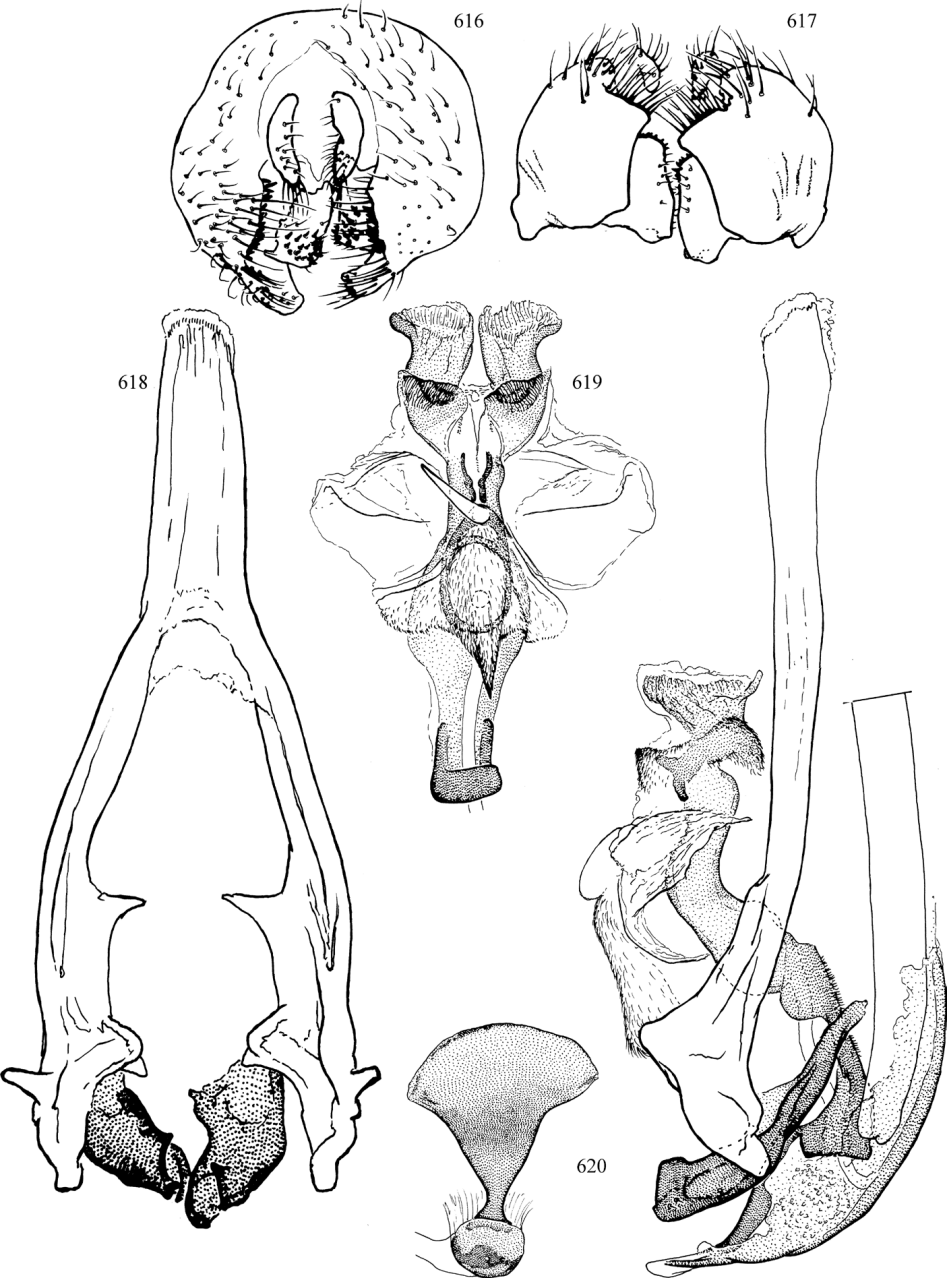
*Liriomyzaquadrisetosa* (Malloch), male genitalia **616** external genitalia, posterior **617** external genitalia, ventral **618** hypandrium and postgonite, ventral **619** phallus, ventral **620** hypandrial complex, left lateral. Originally published in Lonsdale (2011).

**Figures 621–625.**
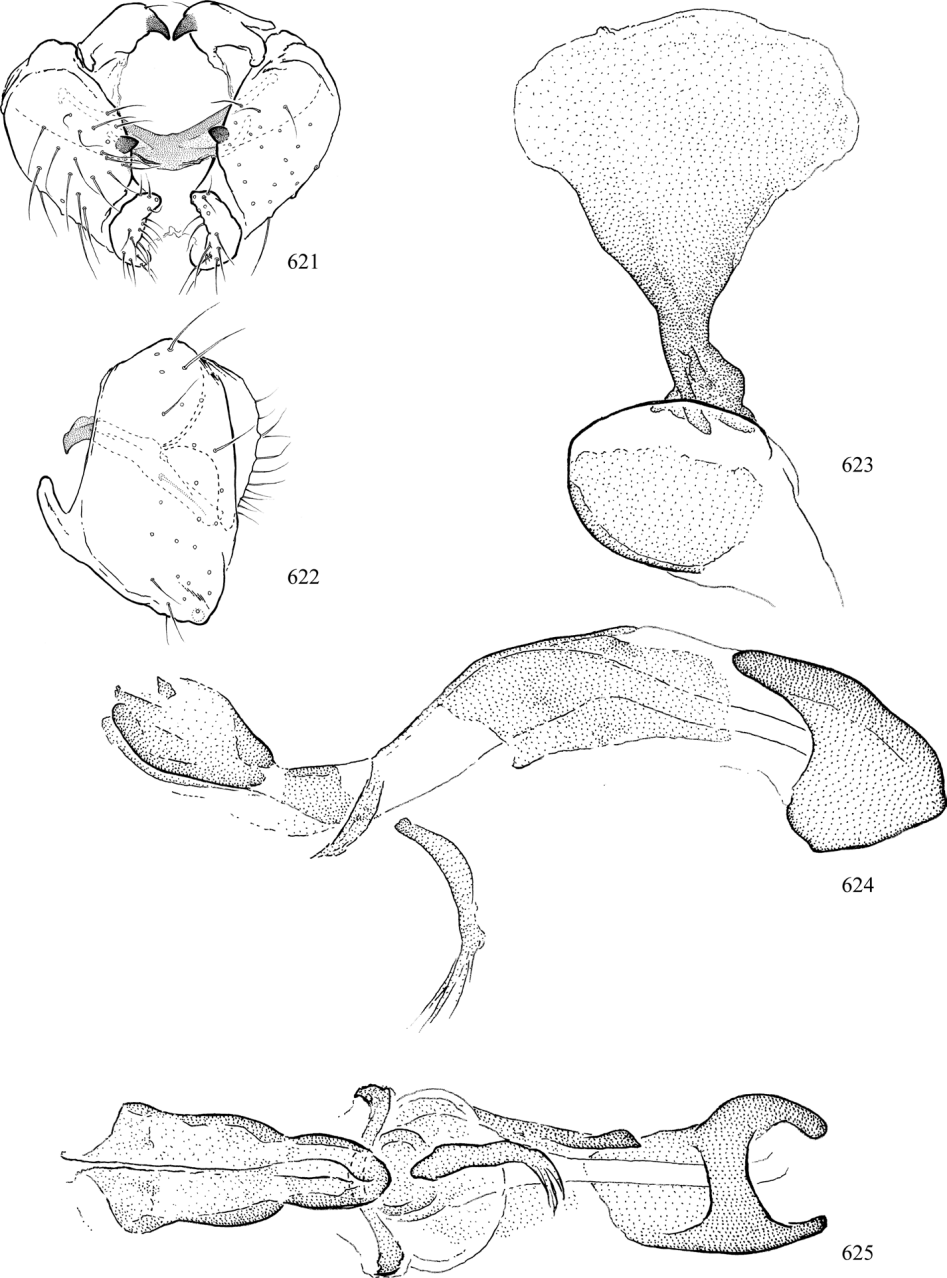
*Liriomyzasativae* Blanchard, male genitalia **621** external genitalia, ventral **622** external genitalia, left lateral **623** ejaculatory apodeme **624** phallus, left lateral **625** phallus, ventral. Originally published in Lonsdale (2017).

**Figures 626–635.**
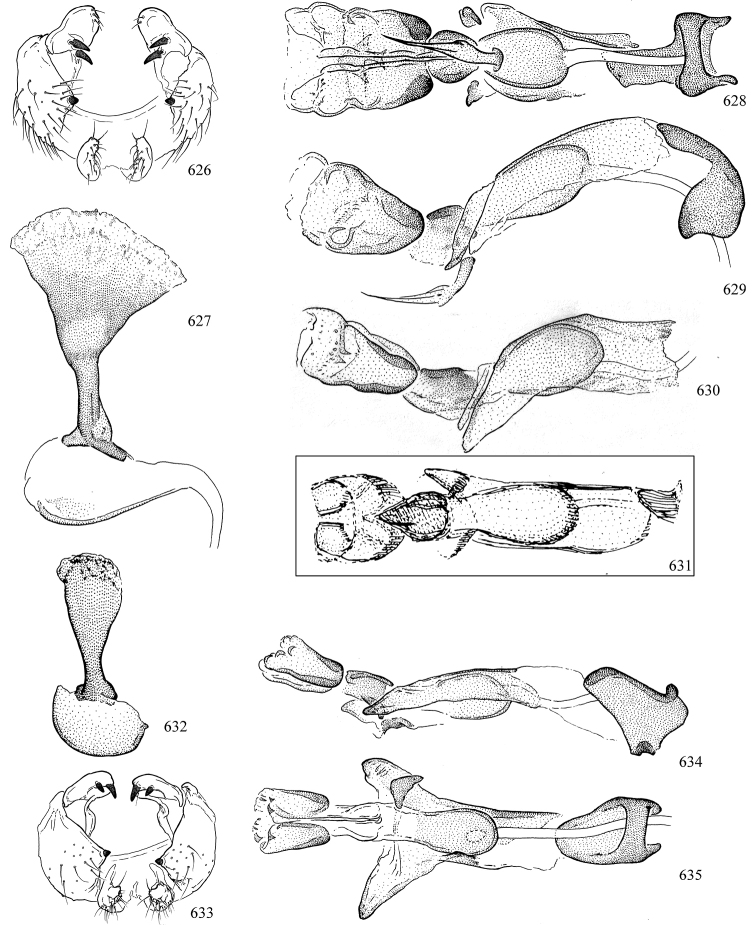
*Liriomyzatemperata* Spencer, male genitalia **626** external components, ventral **627** ejaculatory apodeme **628** phallus, ventral **629** phallus, left lateral **630** holotype phallus, left lateral **631** holotype phallus, ventral. Originally published in Lonsdale (2011), with holotype phallus venter reprinted from Spencer and Steyskal (1986) **632–635***L.trifoliearum* Spencer, male genitalia **632** ejaculatory apodeme **633** external genitalia, ventral **634** phallus, left lateral **635** phallus, ventral. Originally published in Lonsdale (2011).

**Figures 636–644.**
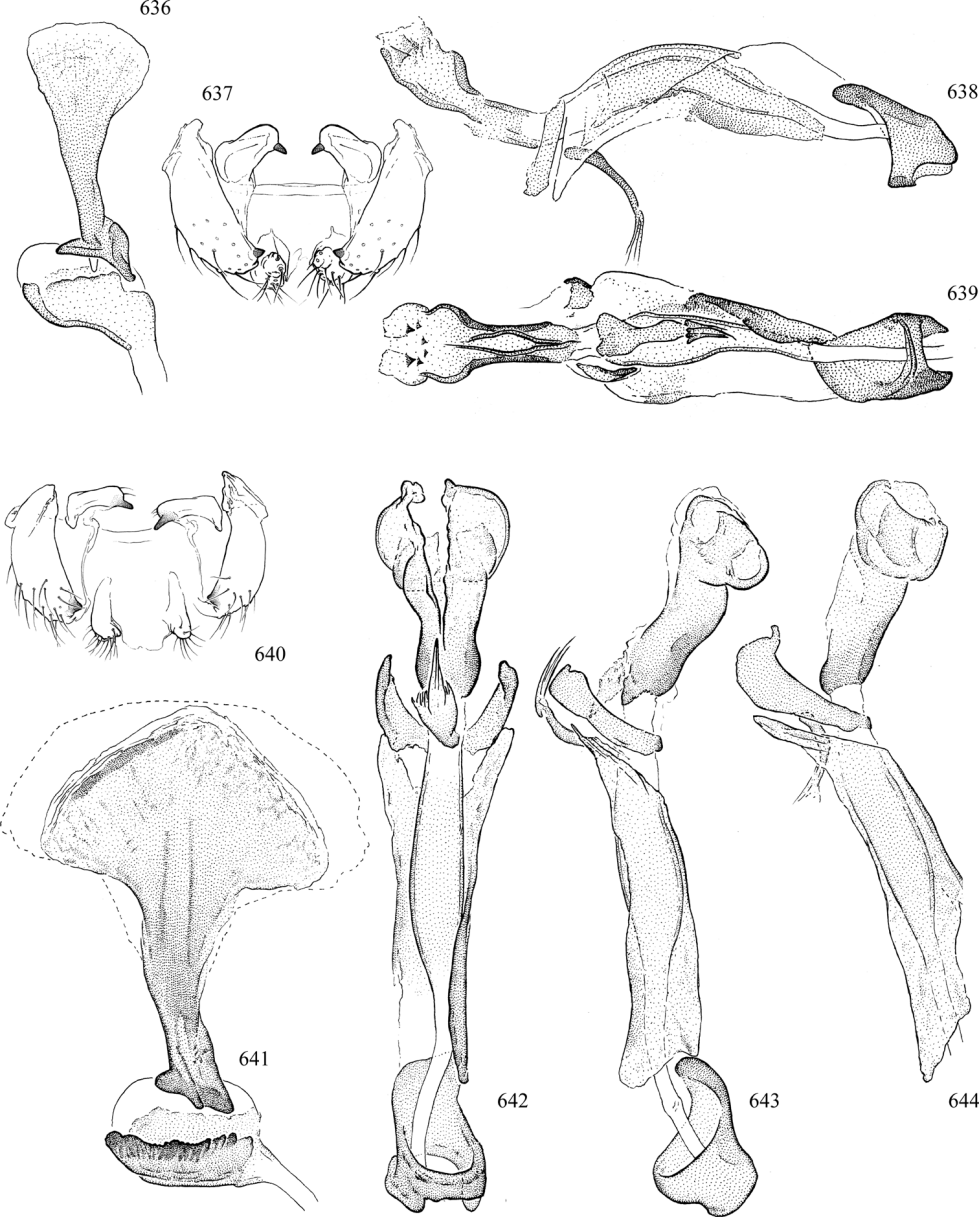
*Liriomyzatrifolii* (Burgess), male genitalia **636** ejaculatory apodeme **637** external genitalia, ventral **638** phallus, left lateral **639** phallus, ventral **640–644***L.helianthi* Spencer, male genitalia **640** external genitalia, ventral **641** ejaculatory apodeme **642** phallus, ventral **643** phallus, left lateral **644** holotype phallus, left lateral. Originally published in Lonsdale (2011).

**Figures 645–652.**
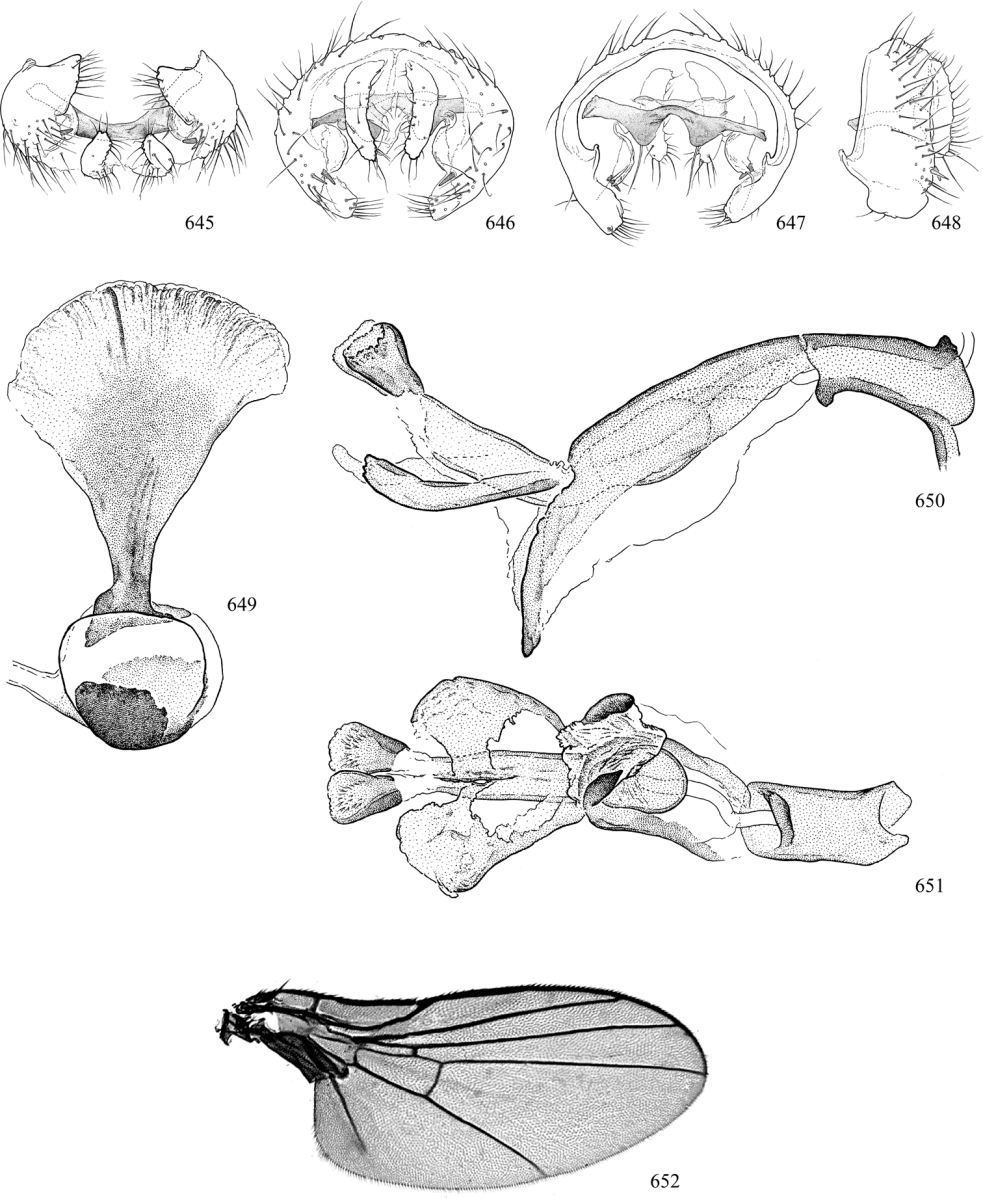
*Liriomyzaviolivora* Spencer, male genitalia **645** external genitalia, ventral (subepandrial sclerite shaded) **646** external genitalia, posterior **647** external genitalia, anterior **648** external genitalia, left lateral **649** ejaculatory apodeme **650** phallus, left lateral **651** phallus, ventral. Originally published in Lonsdale (2017)**652***L.violivora*, wing.

**Figures 653–659.**
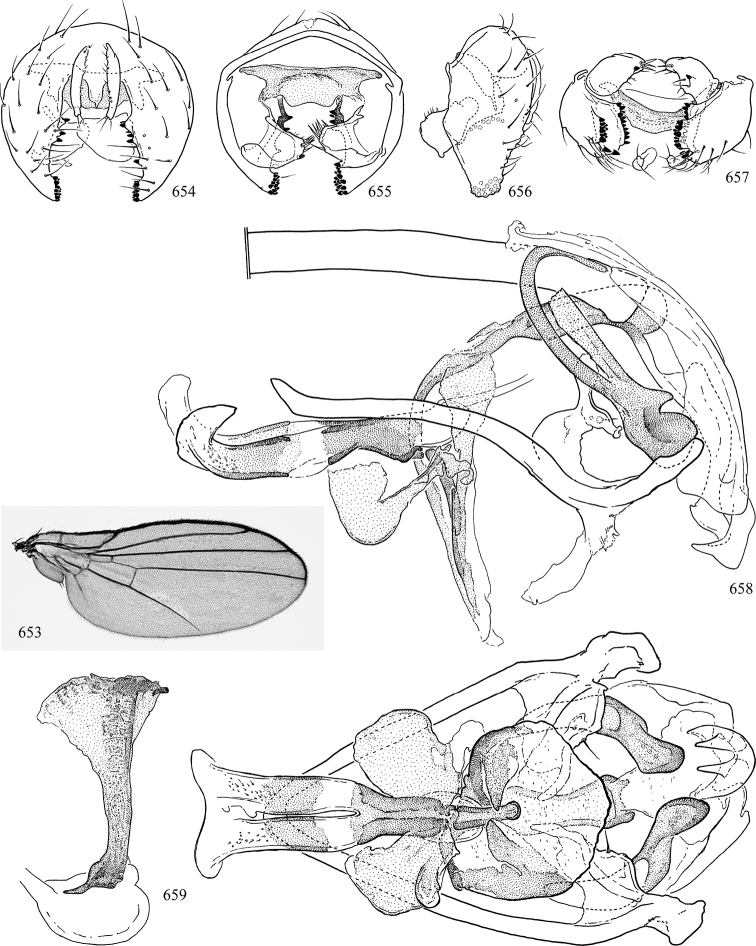
**653***Metopomyzainterfrontalis* (Melander), wing **654–659***M.interfrontalis*, male genitalia **654** external genitalia, posterior **655** external genitalia, anterior **656** external genitalia, left lateral **657** external genitalia, ventral **658** hypandrial complex, left lateral **659** hypandrial complex, ventral.

**Figures 660–666.**
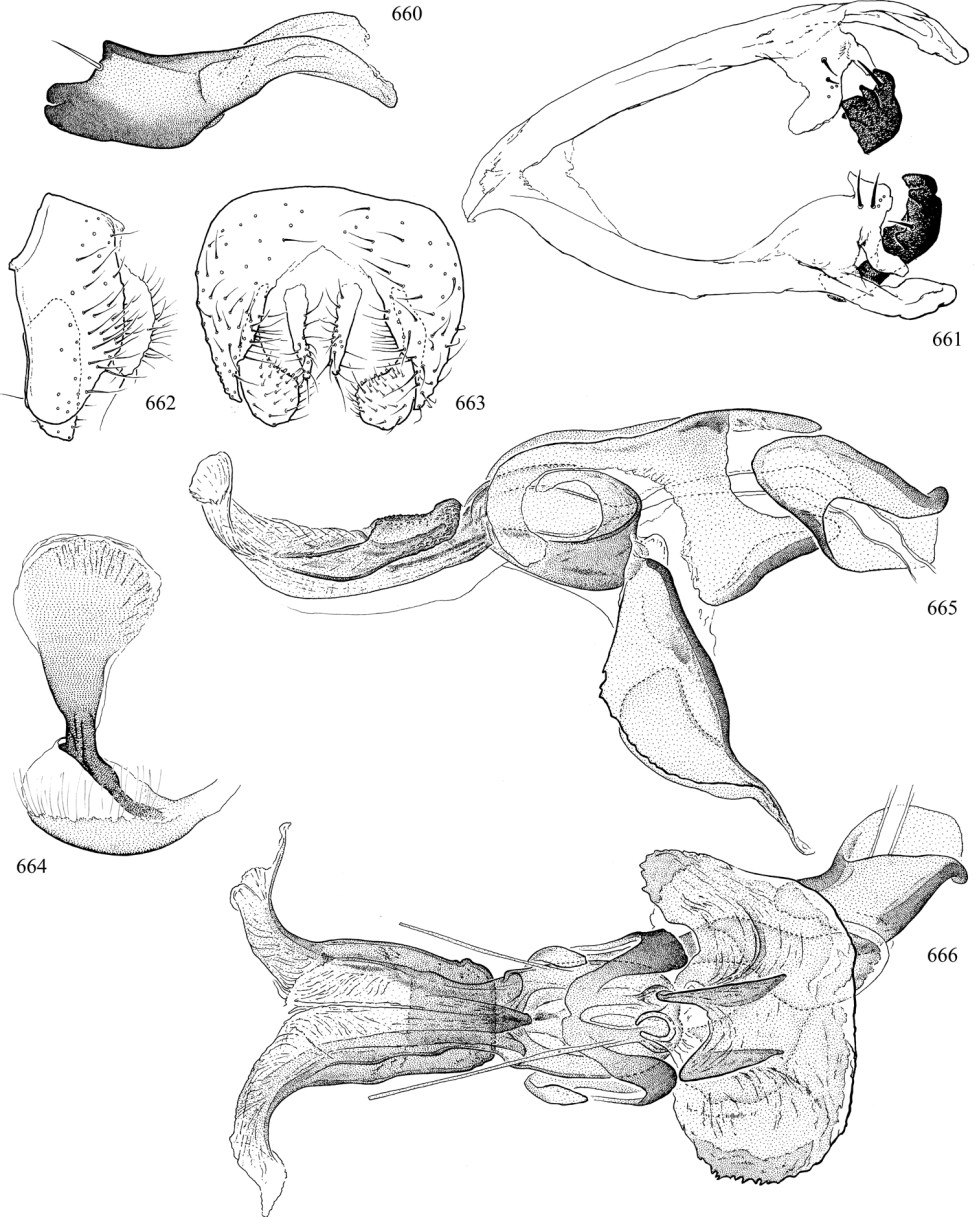
*Nemorimyzamaculosa* (Malloch), male genitalia **660** postgonite, lateral **661** hypandrium and postgonite, ventral **662** external genitalia, left lateral **663** external genitalia, posterior **664** ejaculatory apodeme **665** phallus, left lateral **666** phallus, ventral.

**Figures 667–674.**
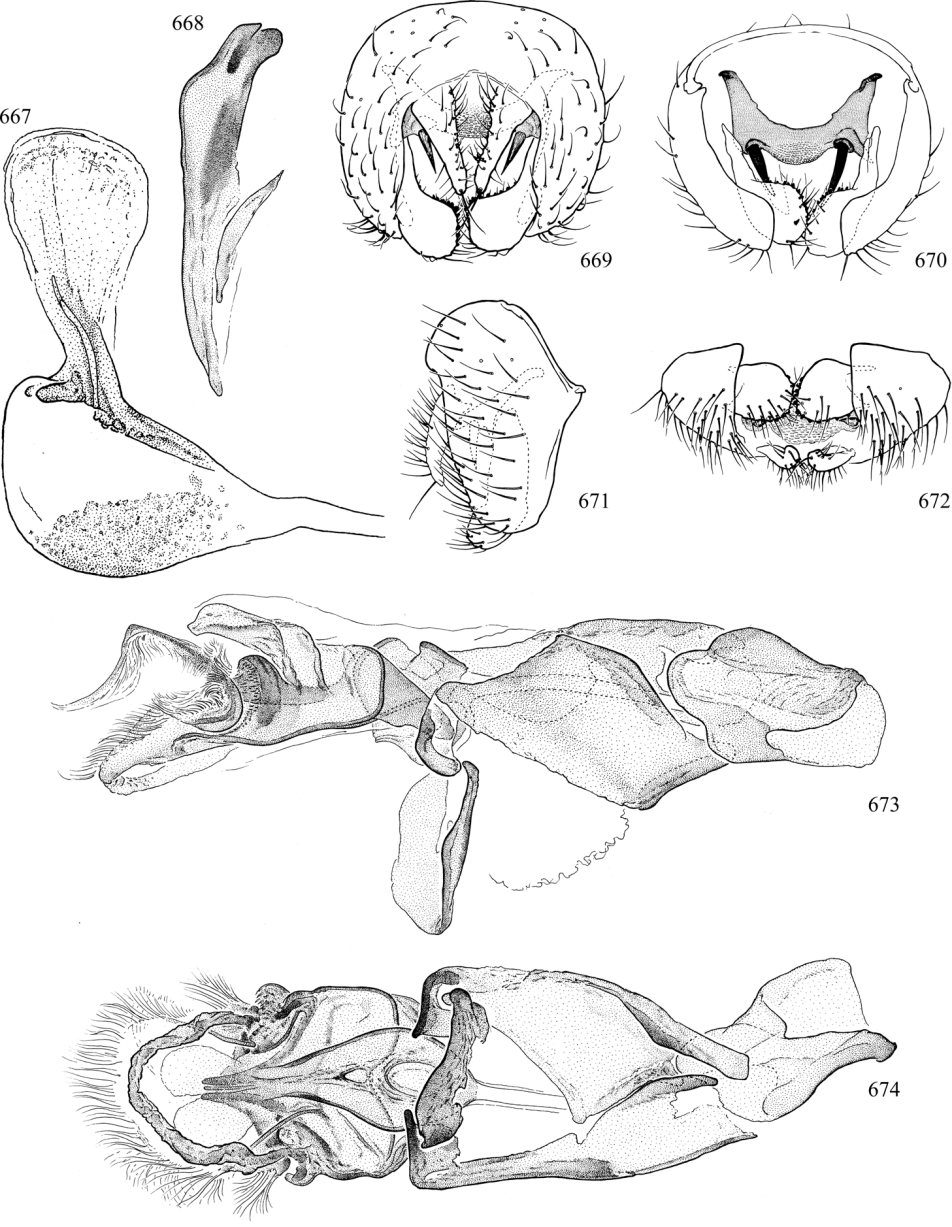
*Nemorimyzaposticata* (Meigen), male genitalia **667** ejaculatory apodeme **668** postgonite, lateral **669** external genitalia, posterior **670** external genitalia, anterior **671** external genitalia, right lateral **672** external genitalia, ventral **673** phallus, left lateral **674** phallus, ventral.

**Figures 675–678.**
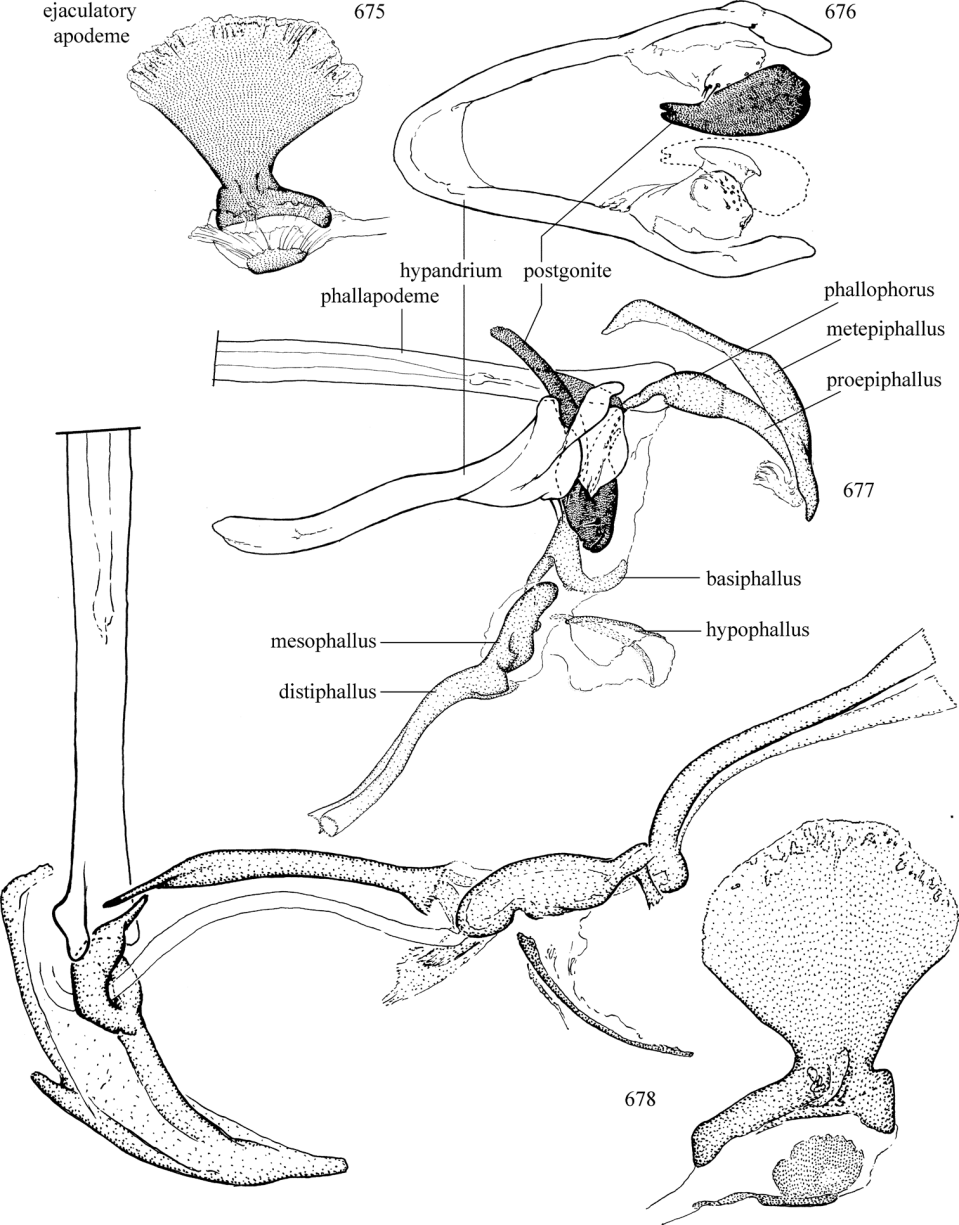
*Phytobiaamelanchieris* (Greene), male genitalia **675** ejaculatory apodeme **676** hypandrium and postgonite, ventral **677** hypandrial complex, left lateral **678***P.betulivora* Spencer, male, phallapodeme, epiphallus, phallus and ejaculatory apodeme, right lateral.

**Figures 679–682.**
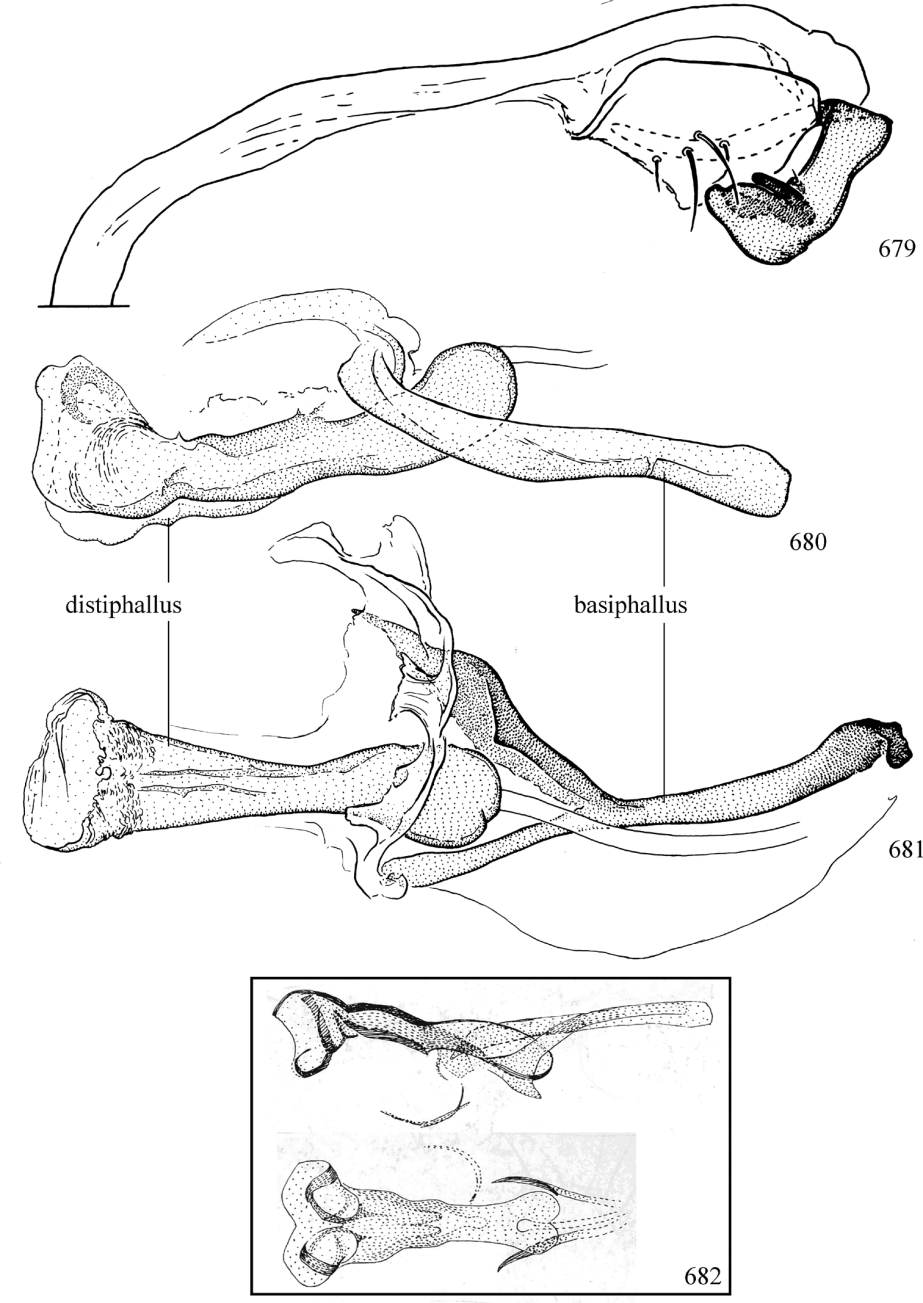
*Phytobiacalyptrata* (Hendel), male genitalia **679** hypandrium and postgonite, ventral **680** phallus of *P.septentrionalis* holotype, ventral **681** phallus, ventral **682** phallus of *A.calyptrata* holotype, left lateral and ventral (from Spencer and Steyskal 1986b).

**Figures 683–688.**
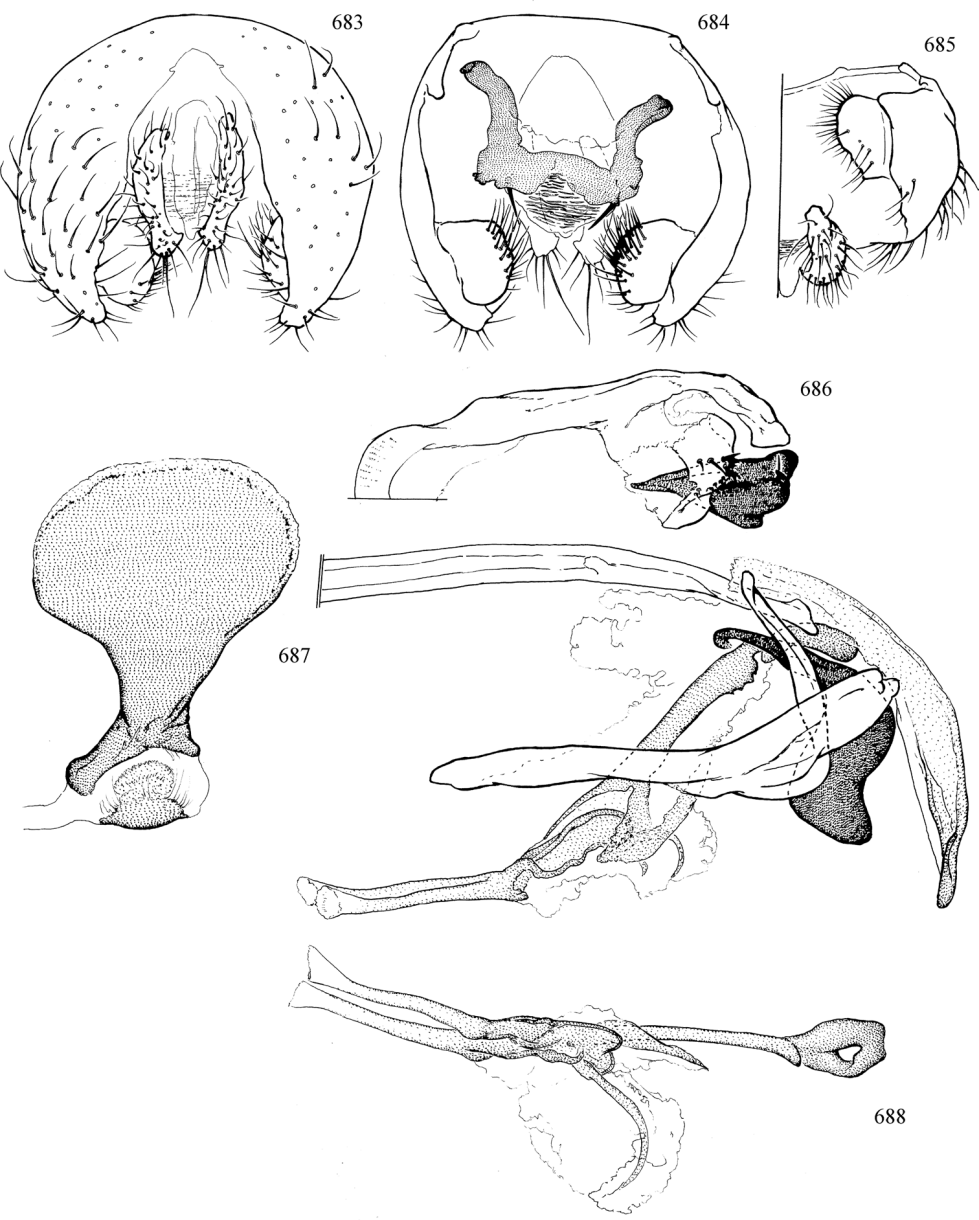
*Phytobiasetosa* (Loew), male genitalia **683** external genitalia, posterior **684** external genitalia, anterior (subepandrial sclerite shaded) **685** external genitalia, ventral **686** hypandrium and postgonite, ventral **687** hypandrial complex, left lateral **688** phallus, ventral.

**Figures 689–692.**
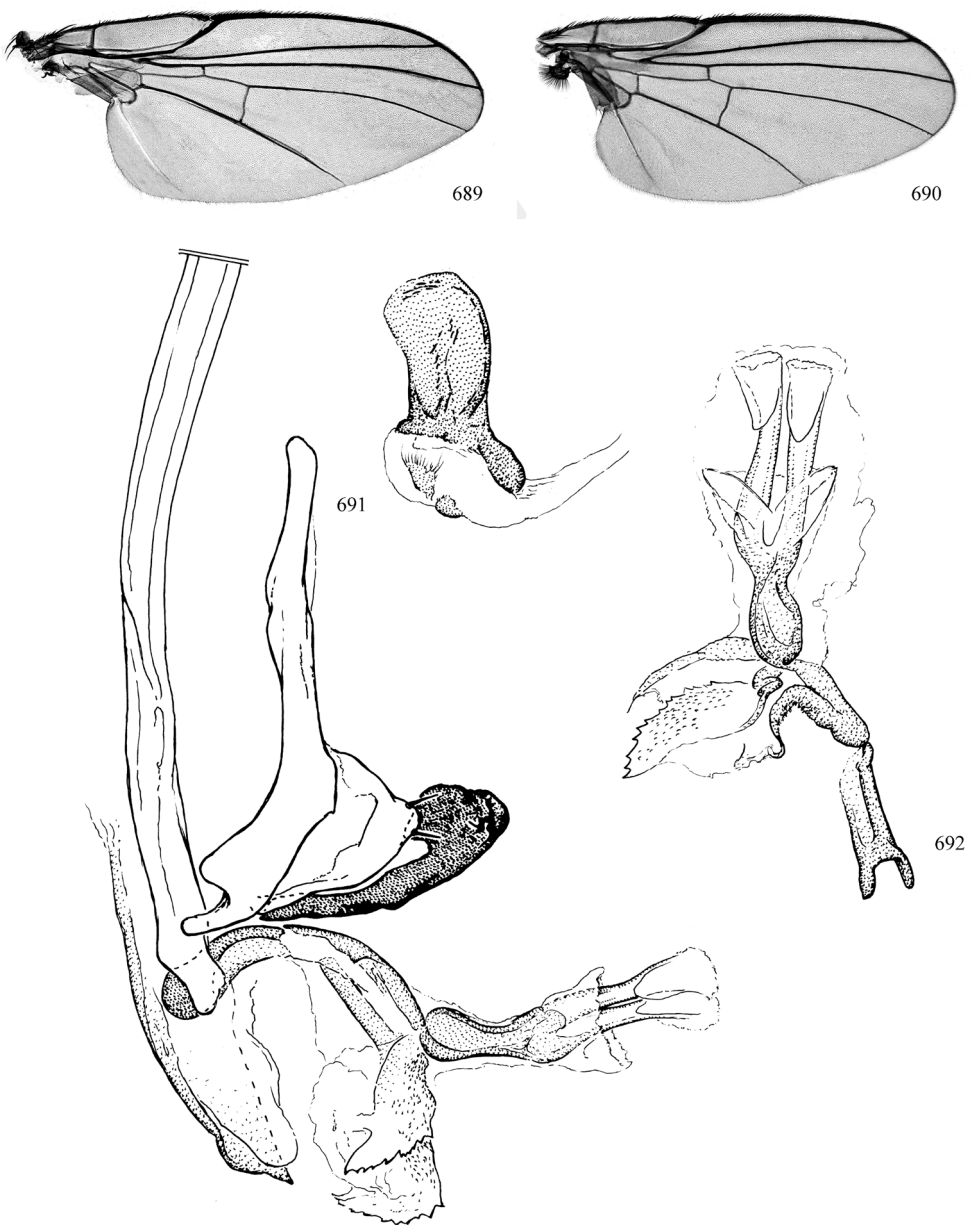
**689***Phytobiasetosa* (Loew), wing **690***P.waltoni* (Malloch), wing **691, 692***P.waltoni*, male genitalia **691** hypandrial complex, right lateral **692** phallus, ventral.

**Figures 693–698.**
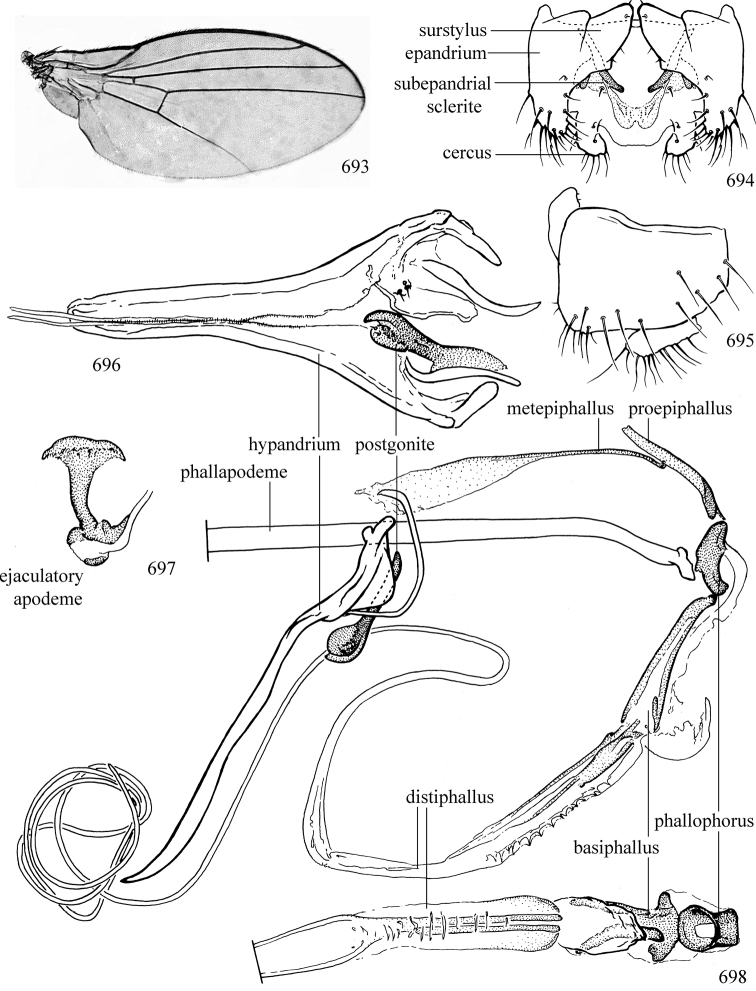
**693***Phytoliriomyzaarctica* (Lundbeck), wing **694–698***P.arctica*, male genitalia **694** external genitalia, ventral **695** external genitalia, left lateral **696** hypandrium and postgonite, ventral **697** hypandrial complex, left lateral **698** phallus, ventral.

**Figures 699–704.**
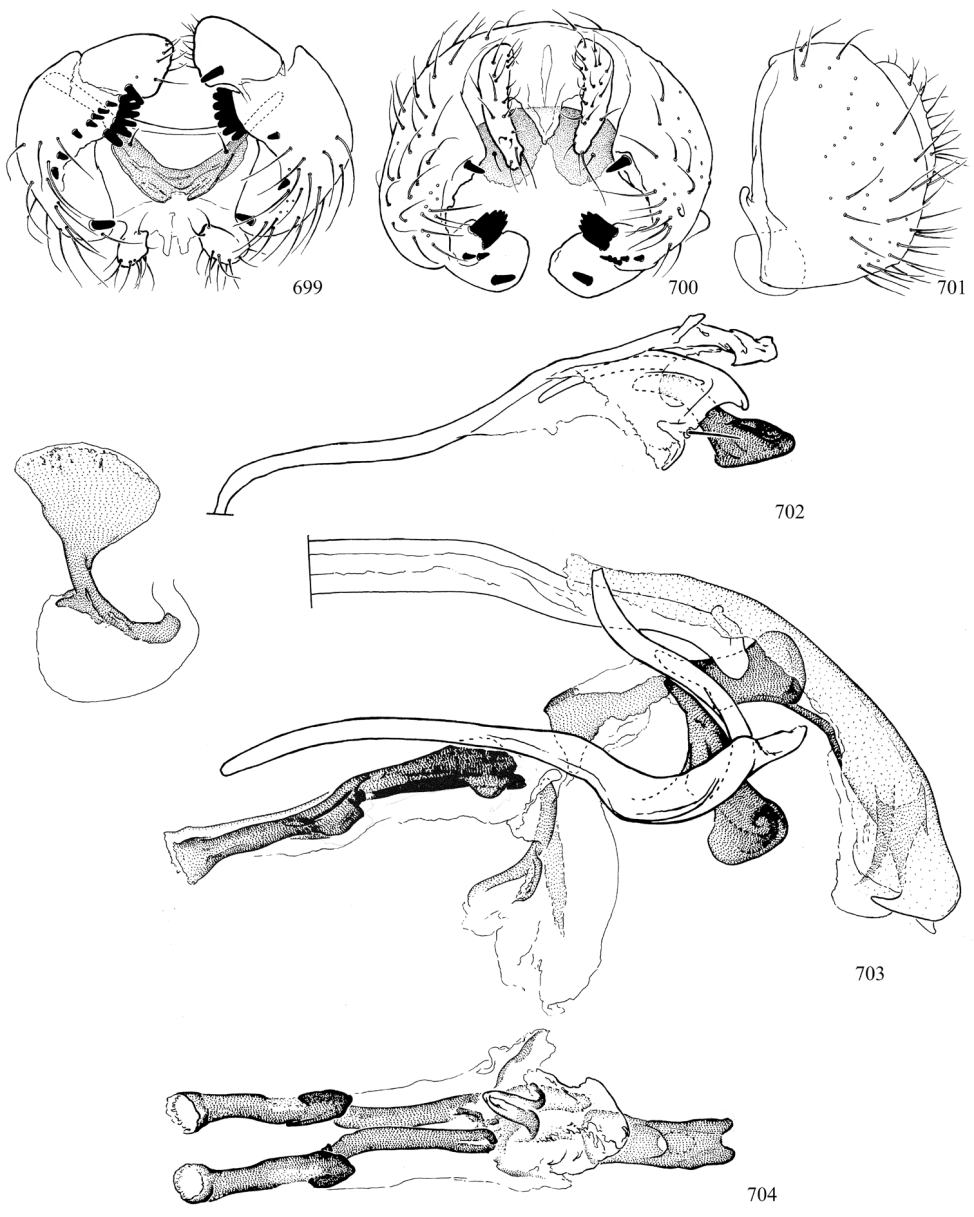
*Phytoliriomyzadorsata* (Siebke), male genitalia **699** external genitalia, ventral (subepandrial sclerite shaded) **700** external genitalia, posterior **701** external genitalia, left lateral **702** hypandrium and postgonite, ventral **703** hypandrial complex, left lateral **704** phallus, ventral.

**Figures 705–710.**
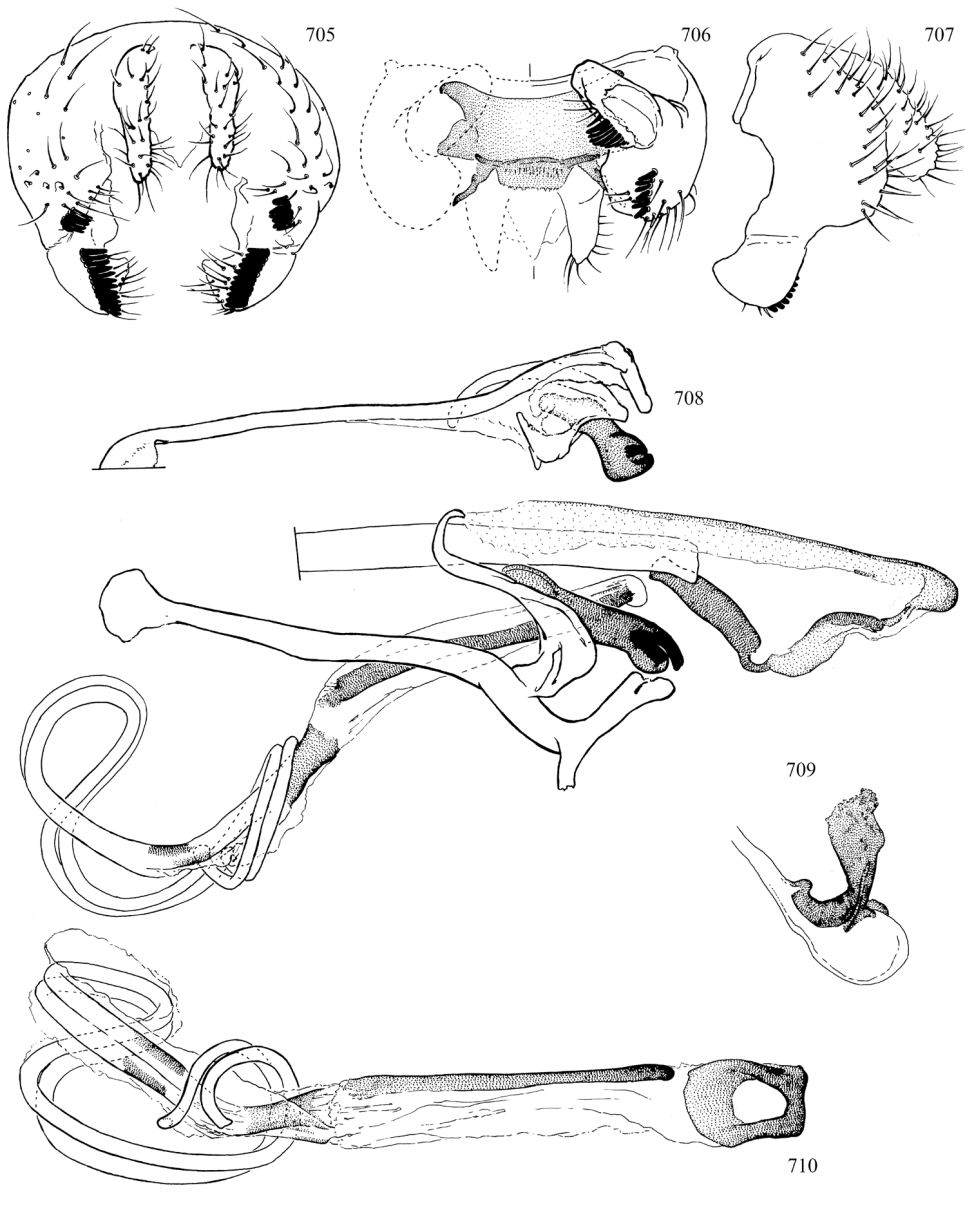
*Phytoliriomyzamelampyga* (Loew), male genitalia **705** external genitalia, posterior **706** external genitalia, ventral (subepandrial sclerite shaded) **707** external genitalia, left lateral **708** hypandrium and postgonite, ventral **709** hypandrial complex, left lateral **710** phallus, ventral.

**Figures 711–716.**
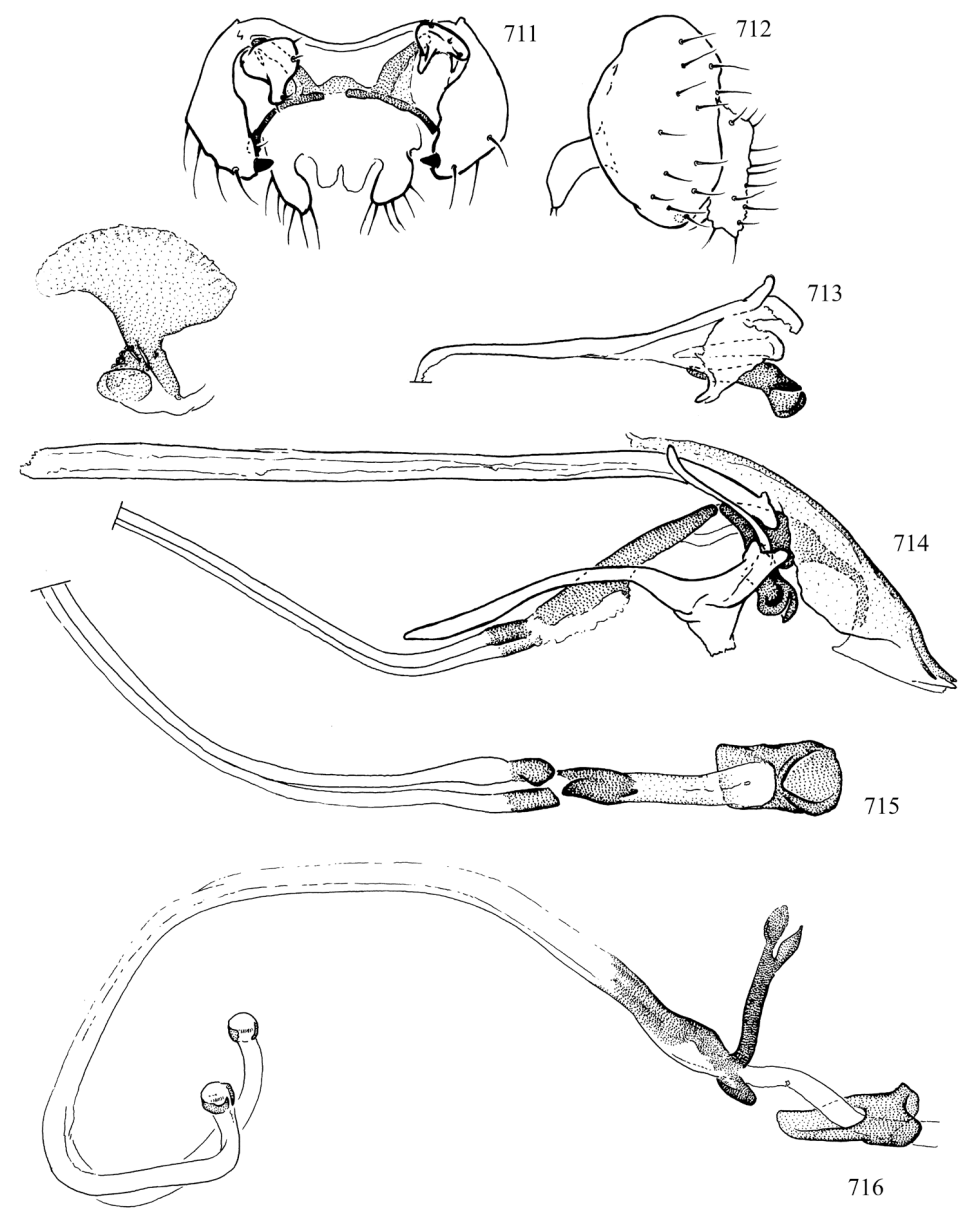
*Phytoliriomyzapilosella* Spencer, male genitalia **711** external genitalia, ventral (subepandrial sclerite shaded) **712** external genitalia, left lateral **713** hypandrium and postgonite, ventral **714** hypandrial complex, left lateral **715** phallus, ventral **716***P.clara* (Melander), phallus, ventral.

**Figures 717–726.**
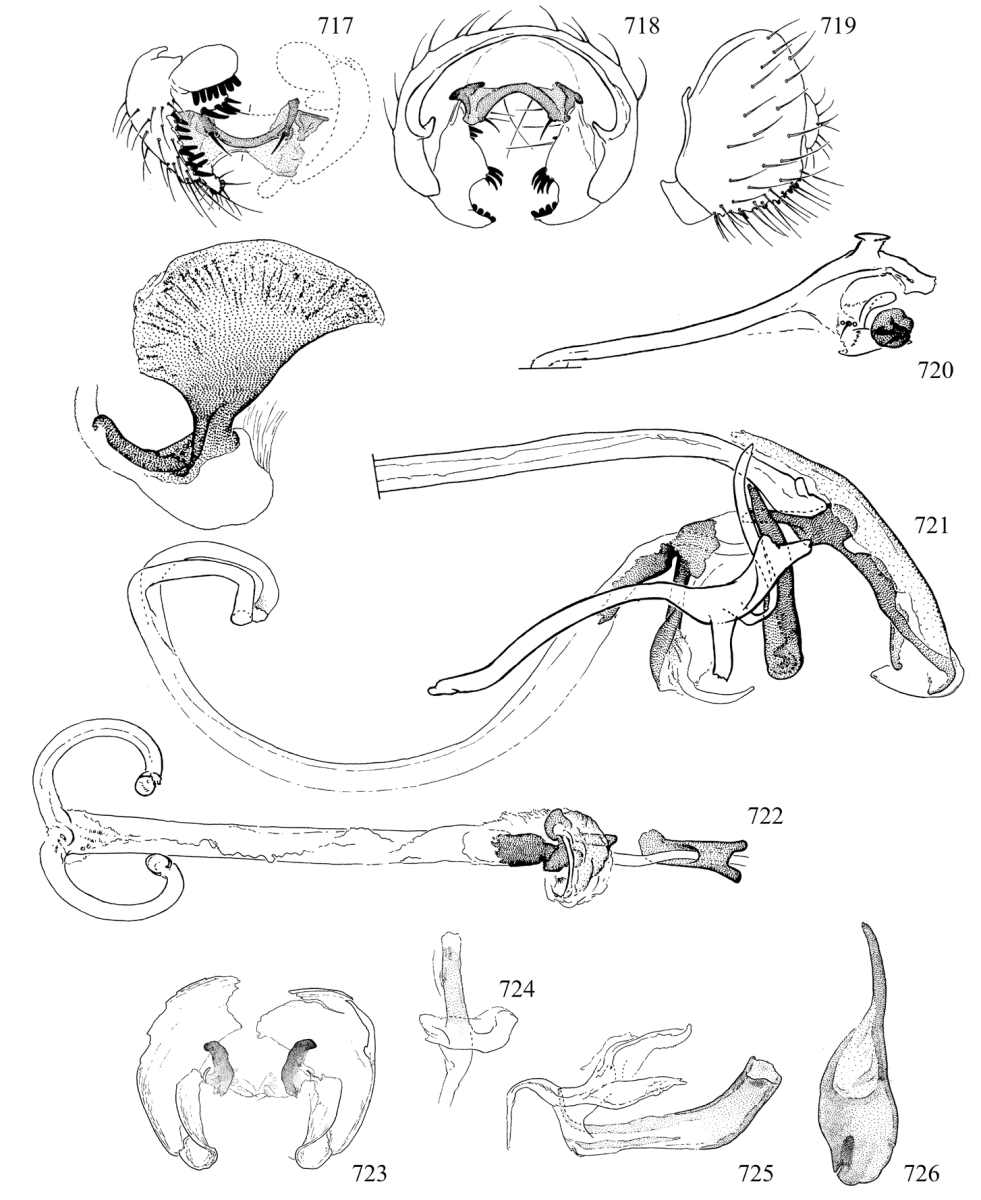
*Phytoliriomyzapulchella* Spencer, male genitalia **717** external genitalia, ventral (subepandrial sclerite shaded) **718** external genitalia, anterior **719** external genitalia, left lateral **720** hypandrium and postgonite, ventral **721** hypandrial complex, left lateral **722** phallus, ventral **723–726***P.robiniae* (Valley), male genitalia **723** external genitalia, anterior view (broken on slide mount; subepandrial sclerite shaded) **724** ejaculatory apodeme **725** phallus, left lateral **726** postgonite, lateral.

**Figures 727–731.**
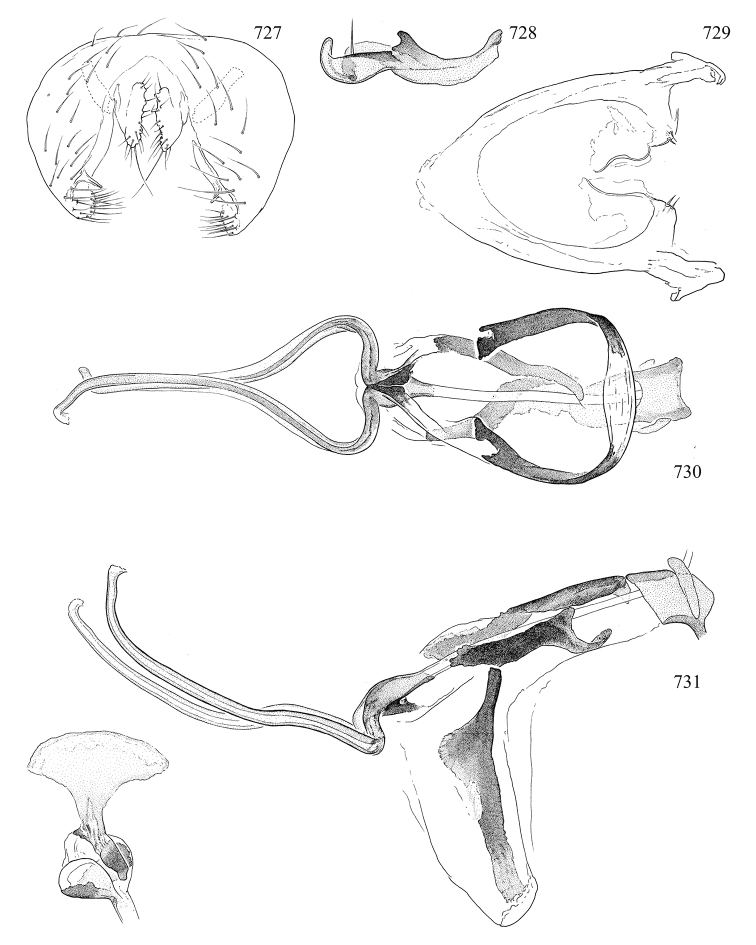
*Phytomyzaaesculi* Eiseman and Lonsdale, male genitalia **727** external components, posterior **728** postgonite, left lateral **729** hypandrium, ventral **730** phallus, ventral **731** phallus, left lateral, and ejaculatory apodeme. Originally published in Eiseman and Lonsdale (2018).

**Figures 732–734.**
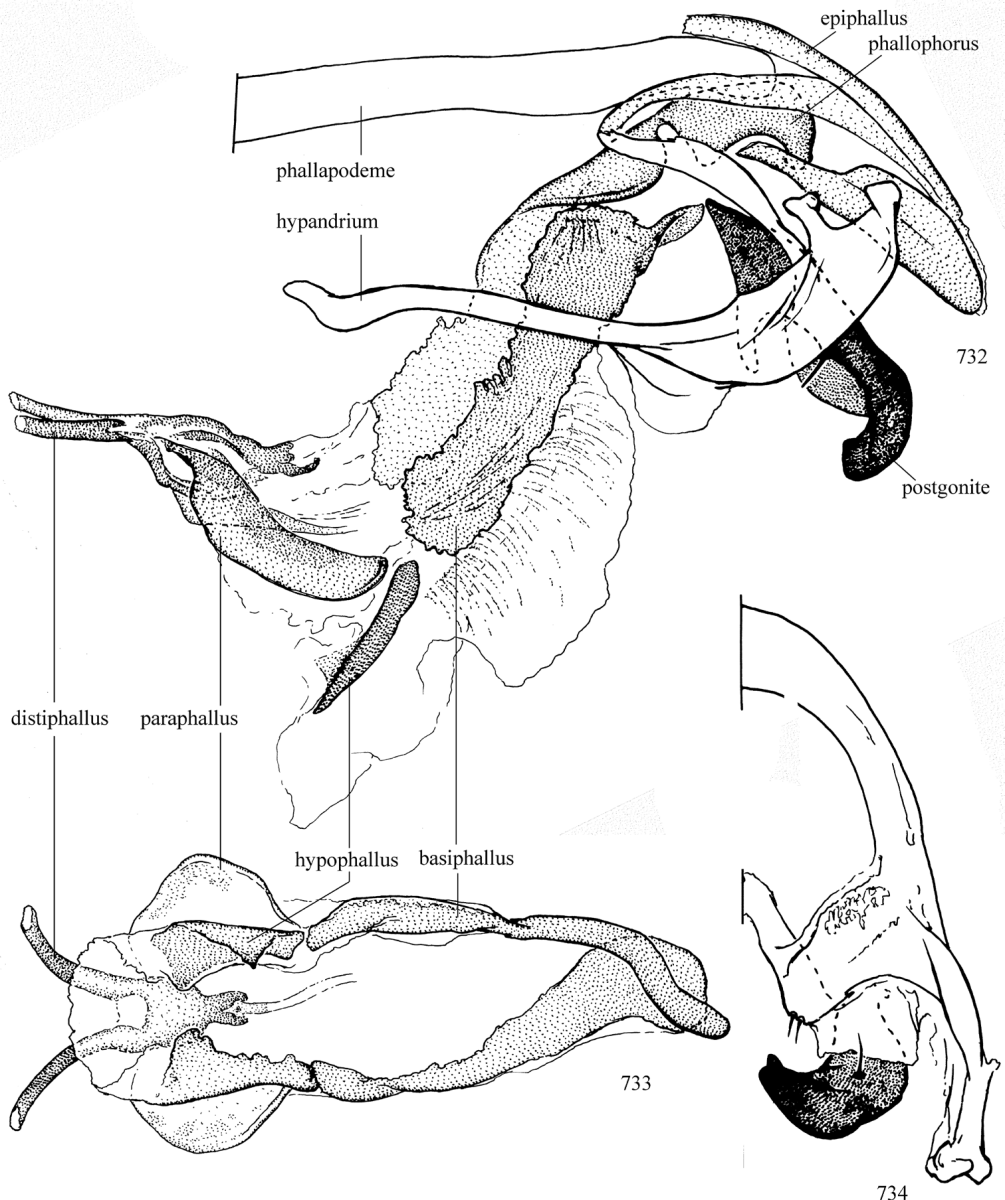
*Phytomyzaaldrichi* Spencer, male genitalia **732** hypandrial complex, left lateral **733** phallus, ventral **734** hypandrium and postgonite, ventral.

**Figures 735–740.**
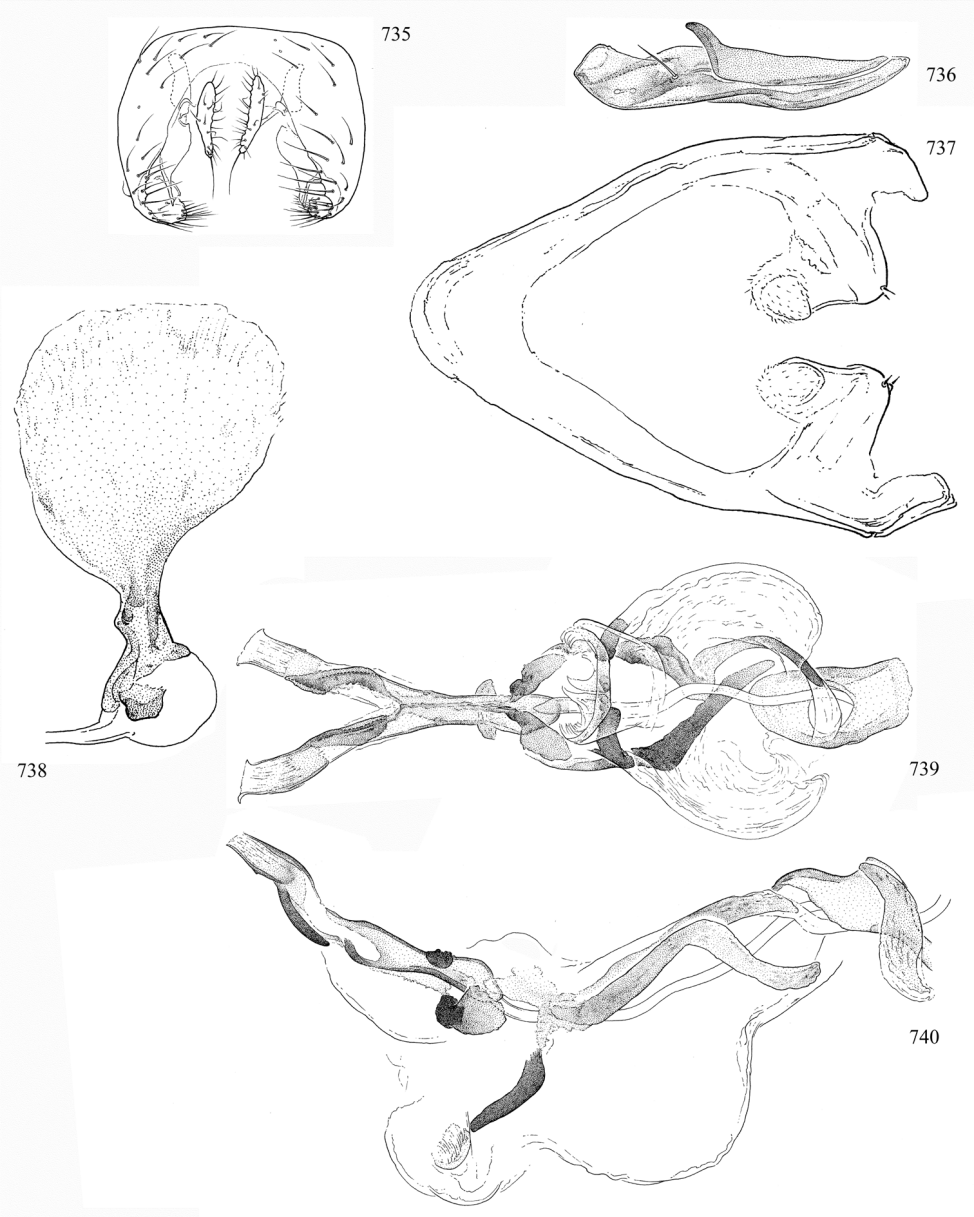
*Phytomyzaaquilegiana* Frost, male genitalia **735** external genitalia, posterior **736** postgonite, lateral **737** hypandrium, ventral **738** ejaculatory apodeme **739** phallus, ventral **740** phallus, left lateral.

**Figures 741–744.**
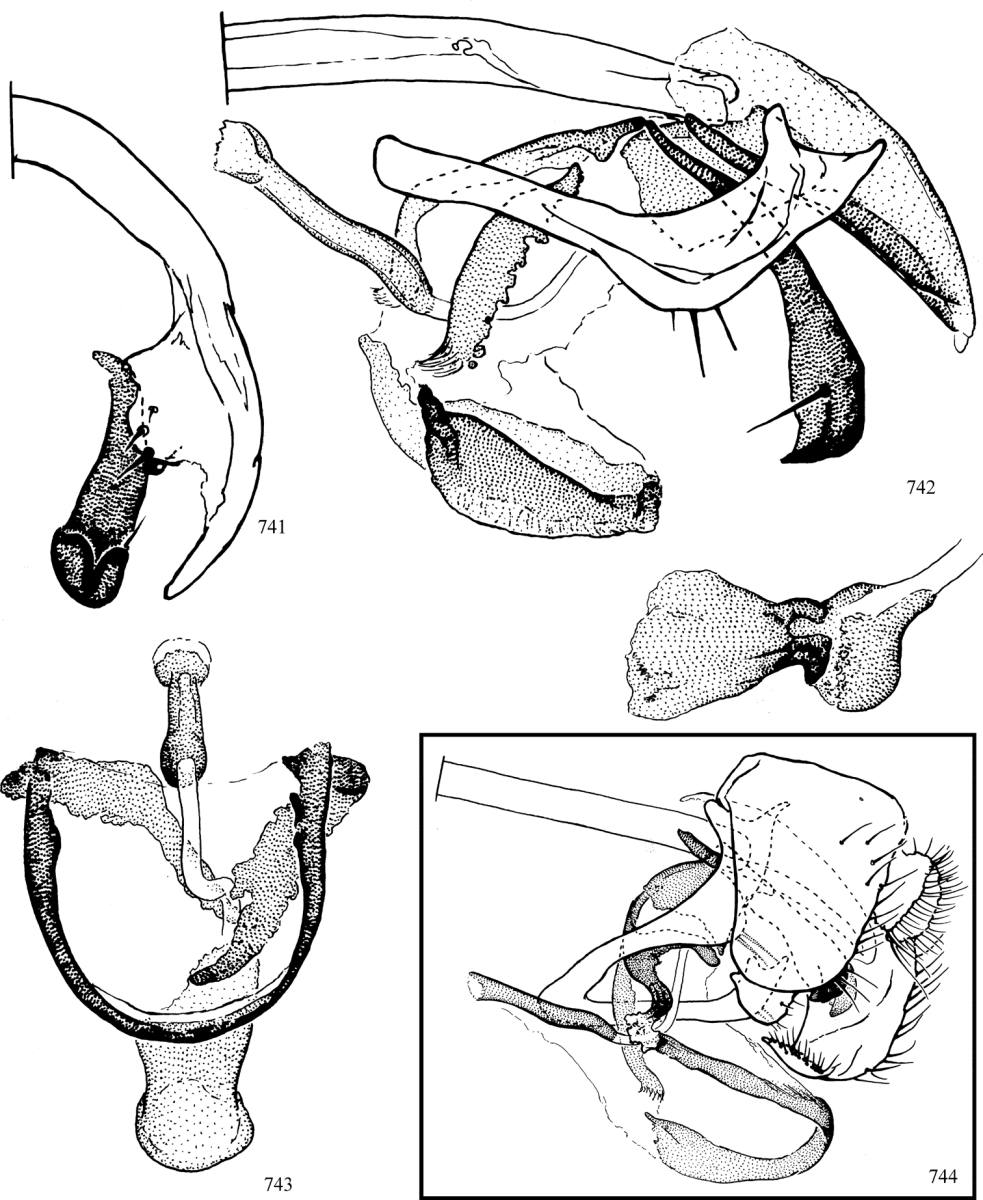
*Phytomyzaaquilegivora* Spencer, male genitalia **741** hypandrium and postgonite, ventral **742** hypandrial complex, left lateral **743** phallus, ventral **744** male genitalia associated, left ventrolateral.

**Figures 745–747.**
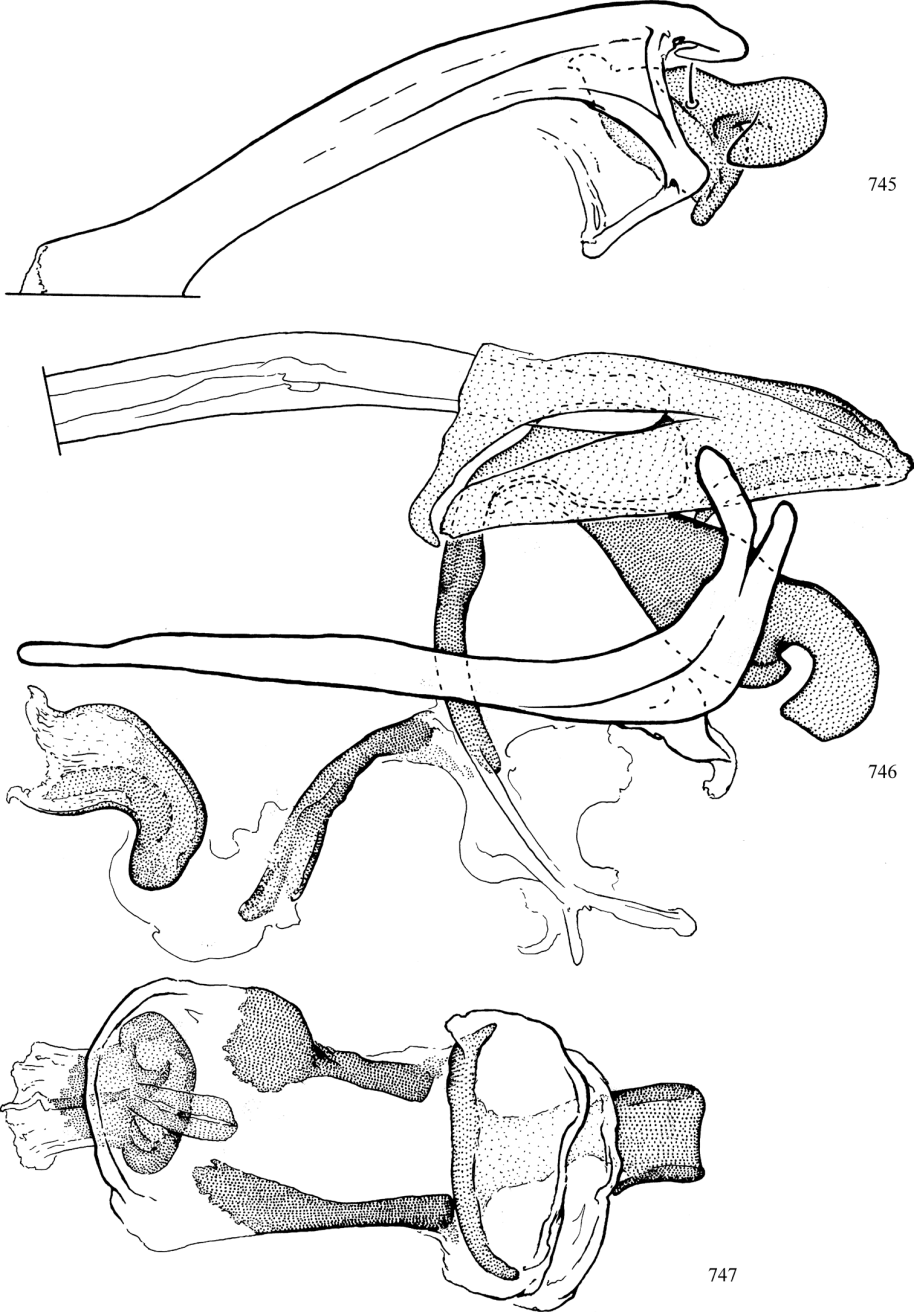
*Phytomyzaavicursa*, sp. nov., male genitalia **745** hypandrium and postgonite, ventral **746** hypandrial complex, left lateral **747** phallus, ventral.

**Figures 748–750.**
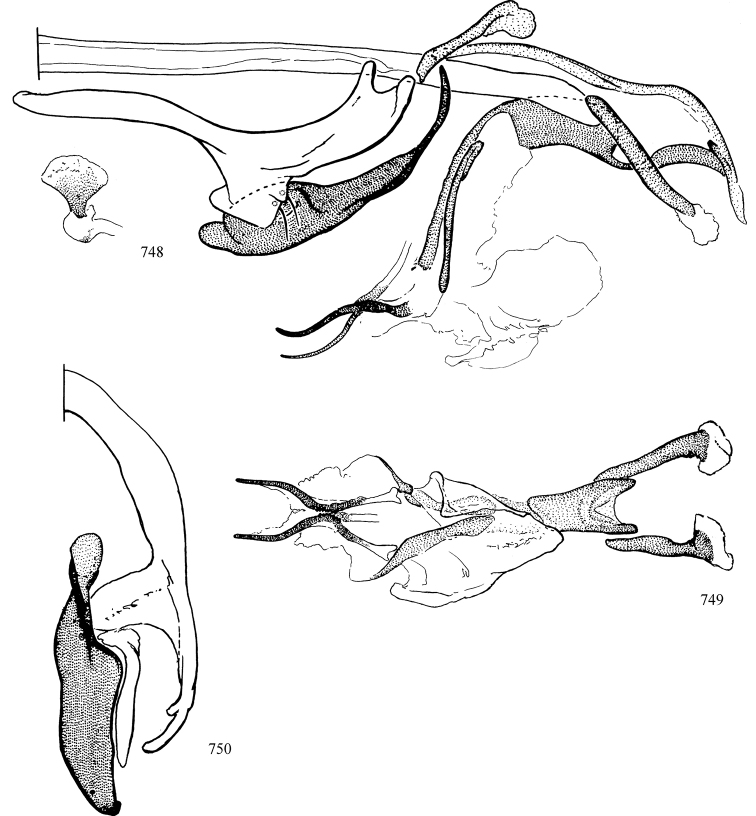
*Phytomyzabicolor* Coquillett, male genitalia **748** hypandrial complex, left lateral **749** phallus, ventral **750** hypandrium and postgonite, ventral.

**Figures 751–753.**
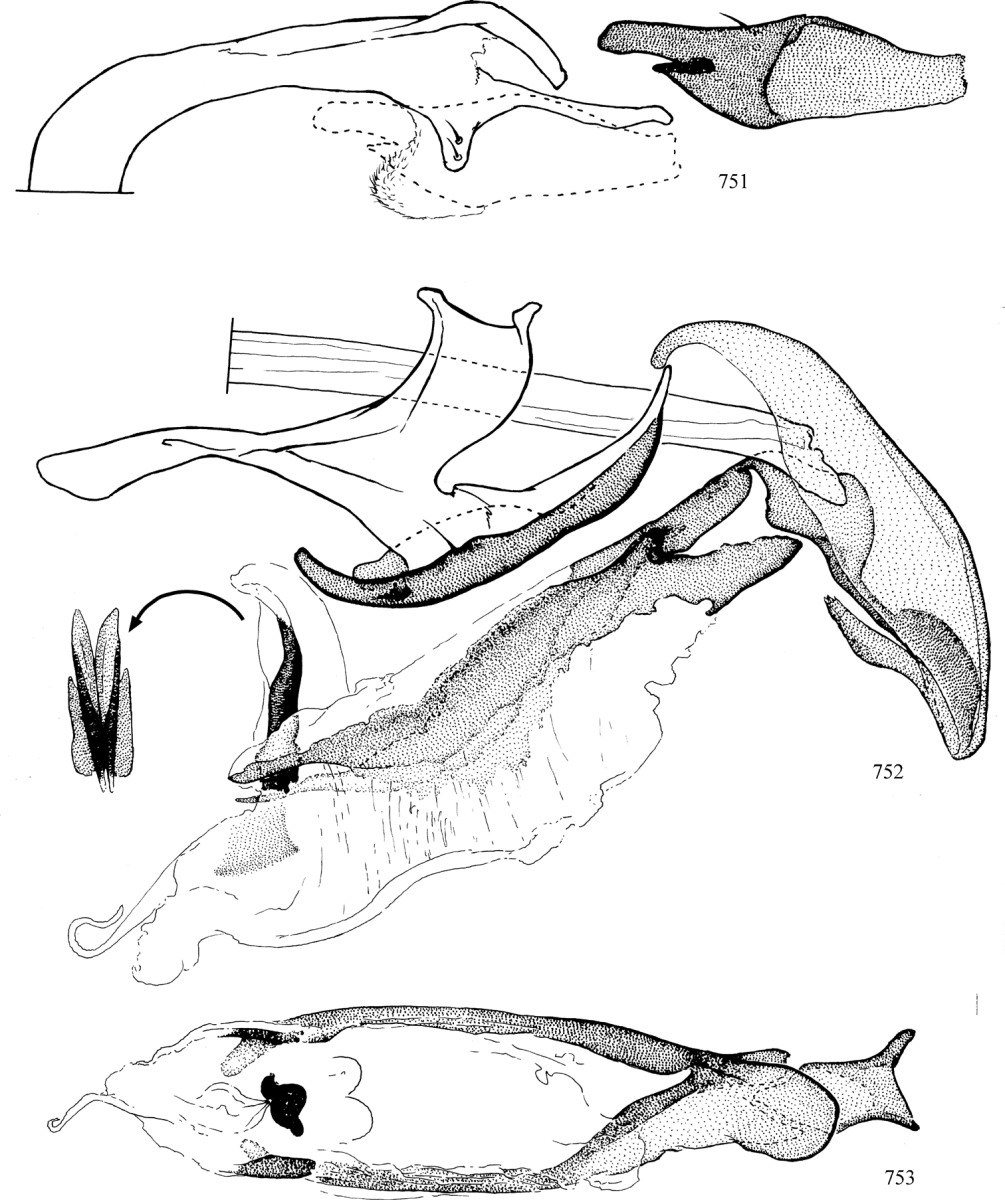
*Phytomyzacatenula*, sp. nov., male genitalia **751** hypandrium and postgonite, ventral **752** hypandrial complex, left lateral, with (functional) anterior view of distiphallus inset **753** phallus, ventral.

**Figures 754–759.**
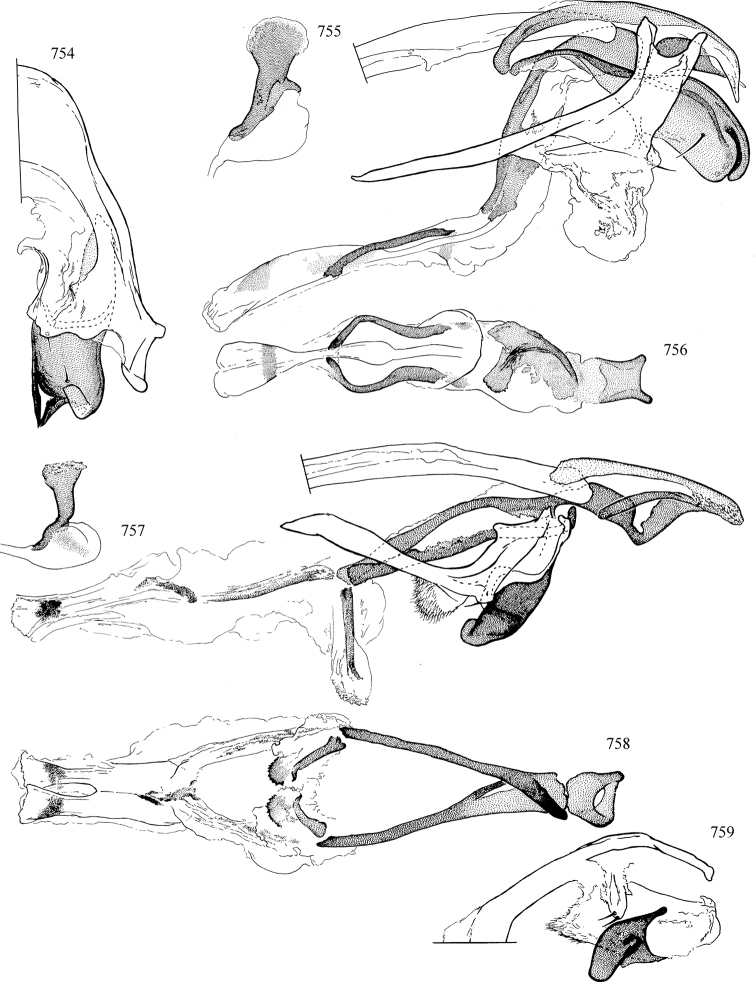
*Phytomyzachelonei* Spencer, male genitalia **754** hypandrium and postgonite, ventral **755** hypandrial complex, left lateral **756** phallus, ventral **757–759***P.osmorhizae* Spencer, male genitalia **757** hypandrial complex, left lateral **758** phallus, ventral **759** hypandrium and postgonite, ventral.

**Figures 760–765.**
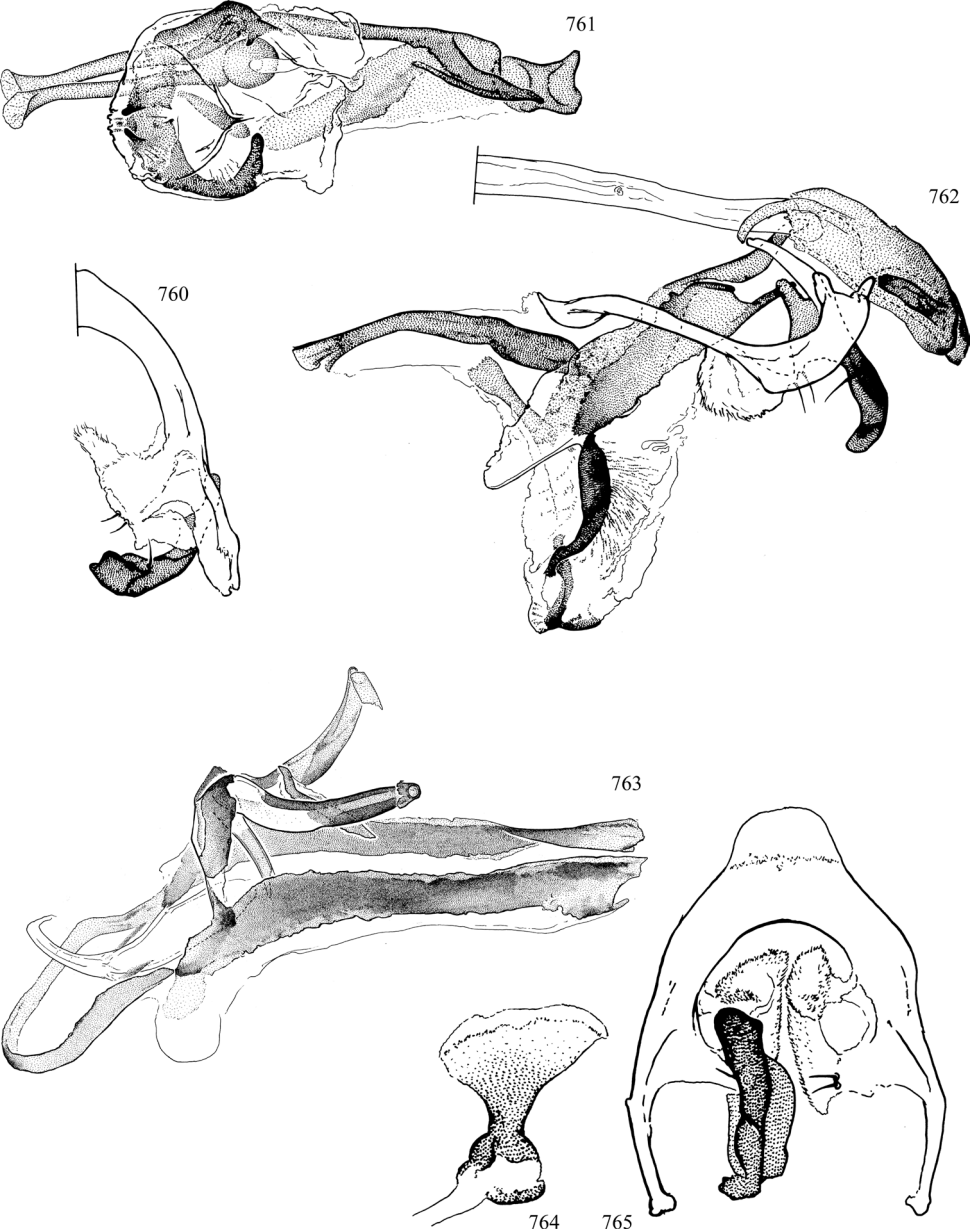
*Phytomyzaclematiphaga* Spencer, male genitalia **760** hypandrium and postgonite, ventral **761** hypandrial complex, left lateral **762** phallus, ventral **763–765***P.compta* (Spencer), male genitalia **763** phallus, left lateral **764** ejaculatory apodeme **765** hypandrium and postgonite, ventral.

**Figures 766–770.**
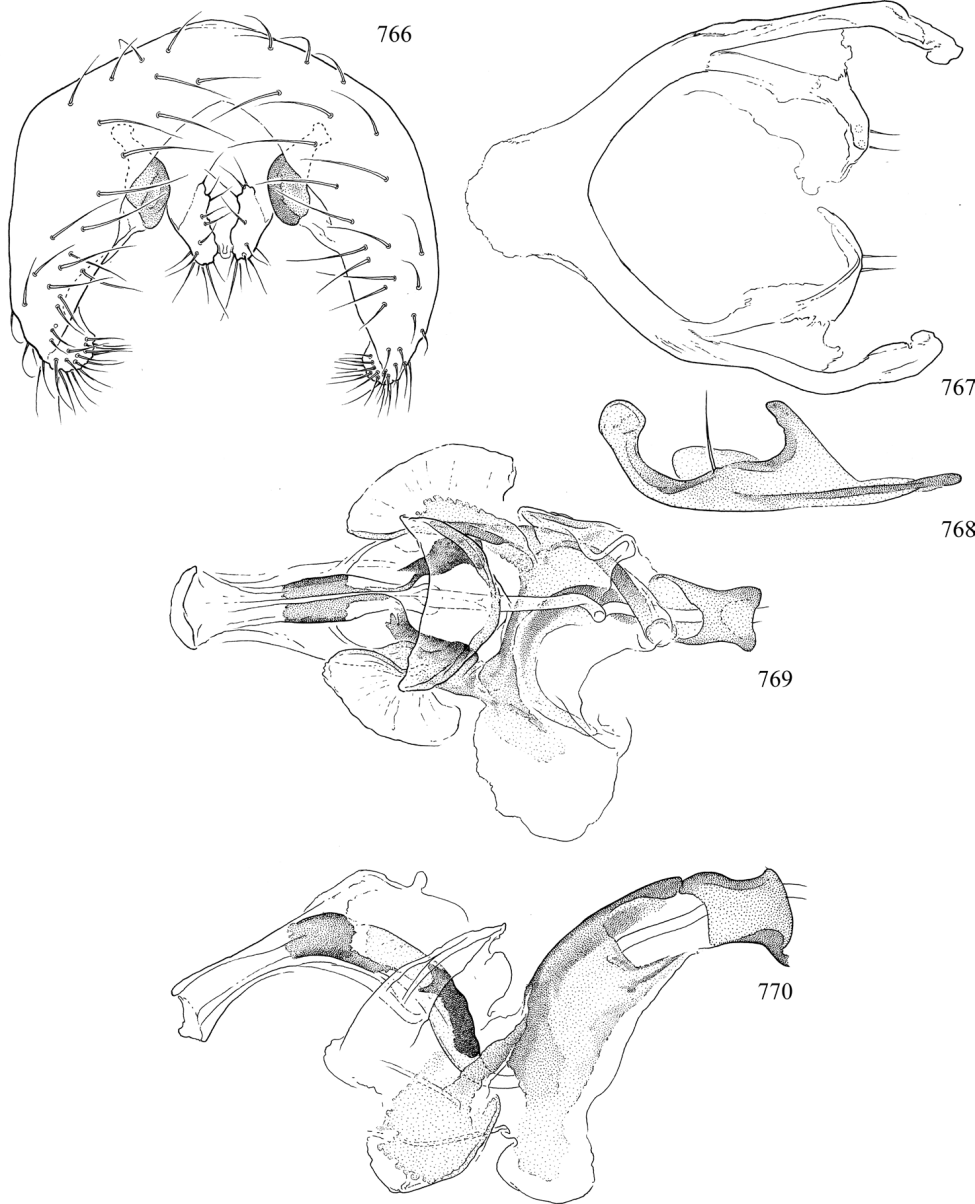
*Phytomyzacrassiseta* Zetterstedt, male genitalia **766** external genitalia, posterior **767** hypandrium, ventral **768** postgonite, lateral **769** phallus, ventral **770** phallus, left lateral.

**Figures 771–777.**
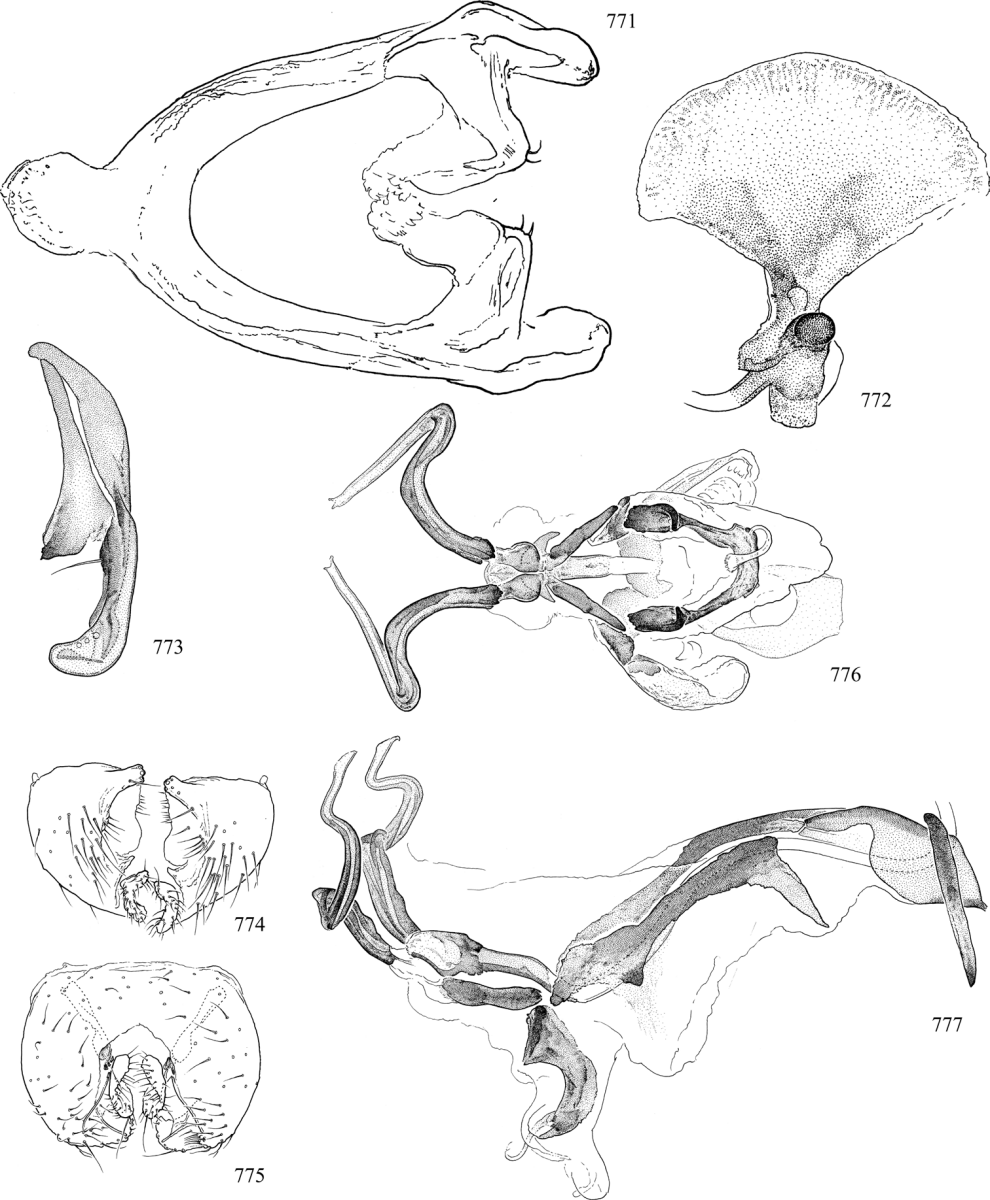
*Phytomyzadavisii* (Walton), male genitalia **771** hypandrium, ventral **772** ejaculatory apodeme **773** postgonite, lateral **774** external genitalia, ventral **775** external genitalia, posterior **776** phallus, ventral **777** phallus, left lateral.

**Figures 778–786.**
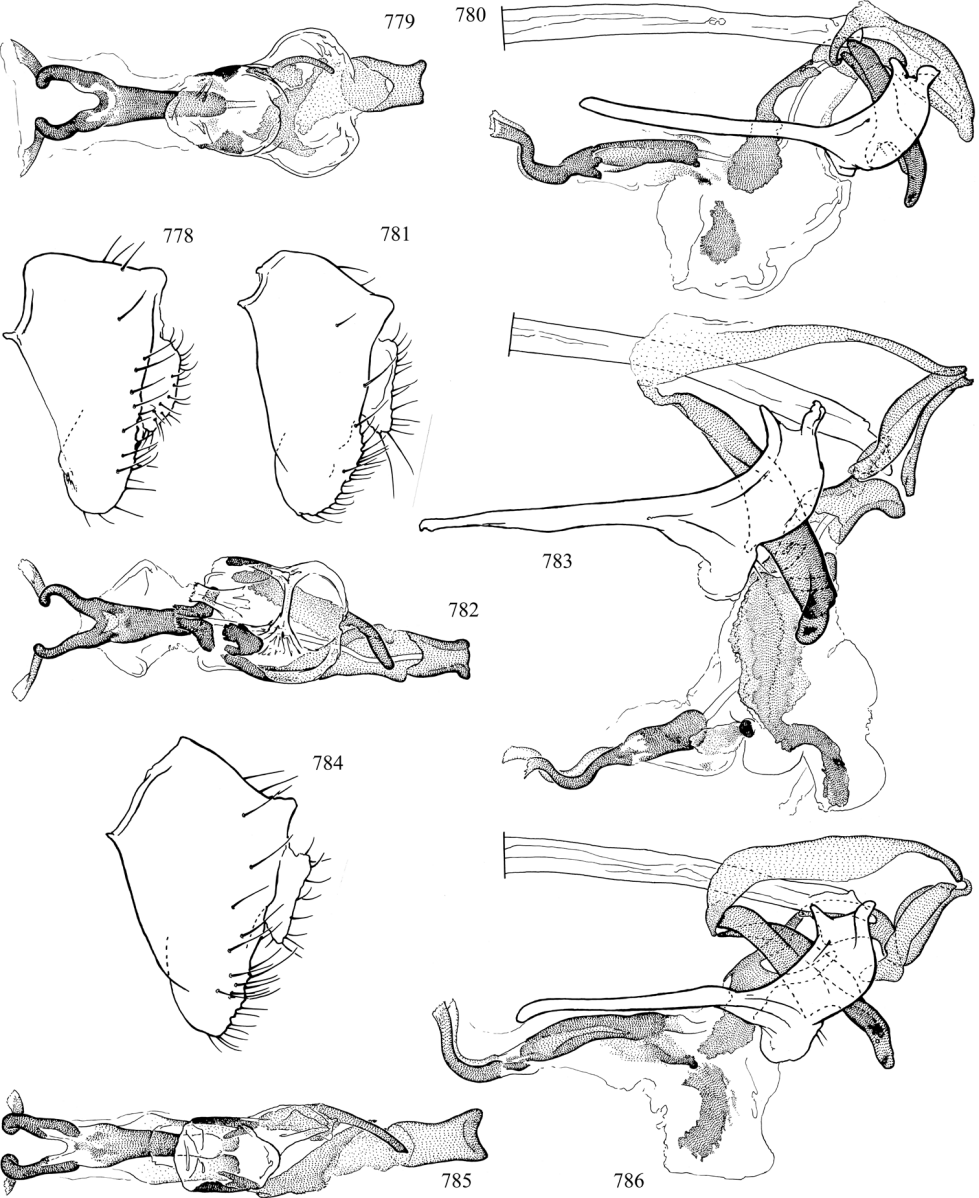
*Phytomyzaditmani* Kulp, male genitalia **778** external genitalia, left lateral **779** phallus, ventral **780** hypandrial complex, left lateral **781–783***P.glabricola* Kulp, male genitalia **781** external genitalia, left lateral **782** phallus, ventral **783** hypandrial complex, left lateral **784–786***P.ilicicola* Loew, male genitalia **784** external genitalia, left lateral **785** phallus, ventral **786** hypandrial complex, left lateral. Originally published in Lonsdale and Scheffer (2011).

**Figures 787–795.**
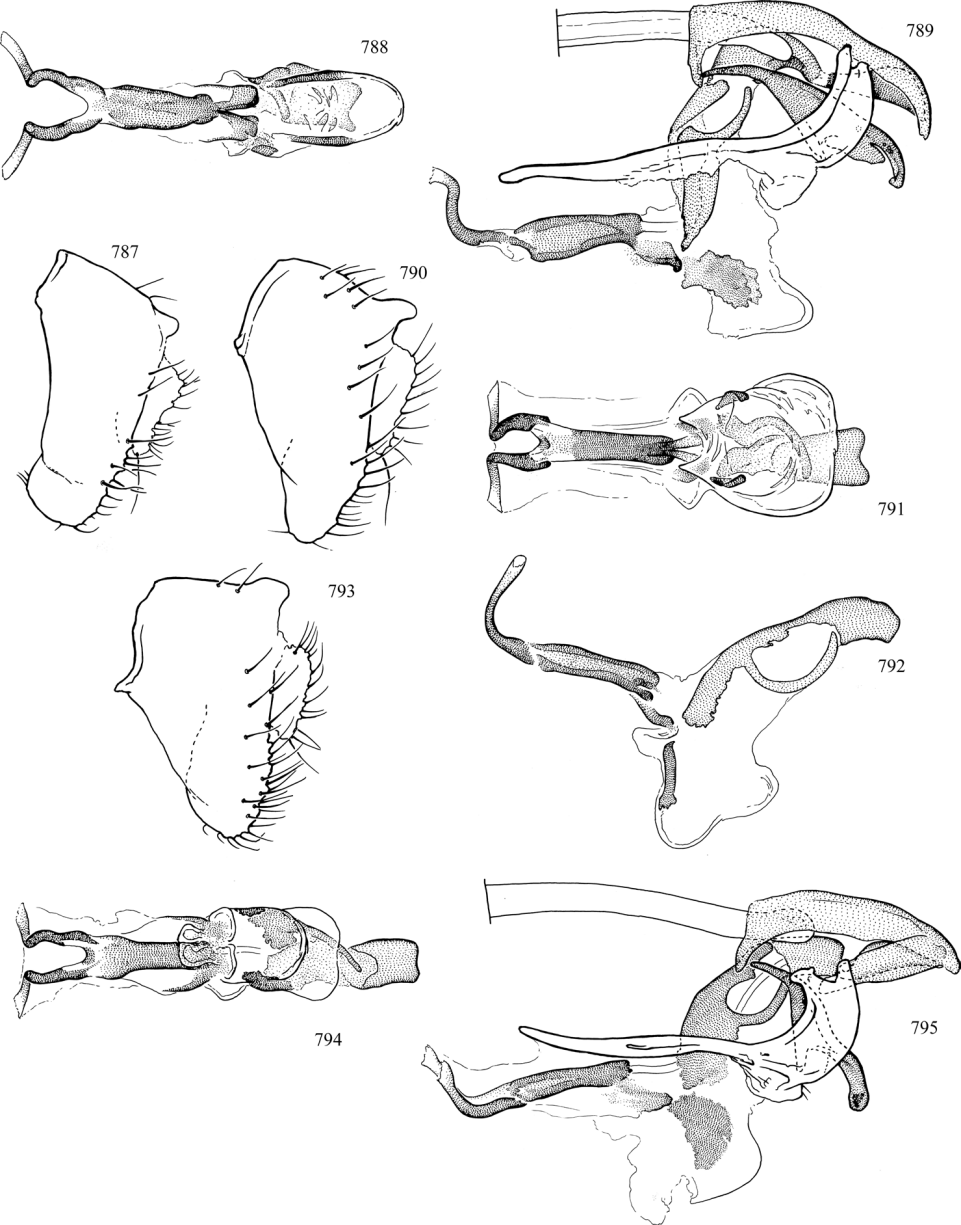
*Phytomyzaleslieae* Lonsdale and Scheffer, male genitalia **787** external genitalia, left lateral **788** phallus, ventral **789** hypandrial complex, left lateral **790–792***P.lineata* Lonsdale and Scheffer, male genitalia **790** external genitalia, left lateral **791** phallus, ventral **792** hypandrial complex, left lateral **793–795***P.opacae* Kulp, male genitalia **793** external genitalia, left lateral **794** phallus, ventral **795** hypandrial complex, left lateral. Originally published in Lonsdale and Scheffer (2011).

**Figures 796–801.**
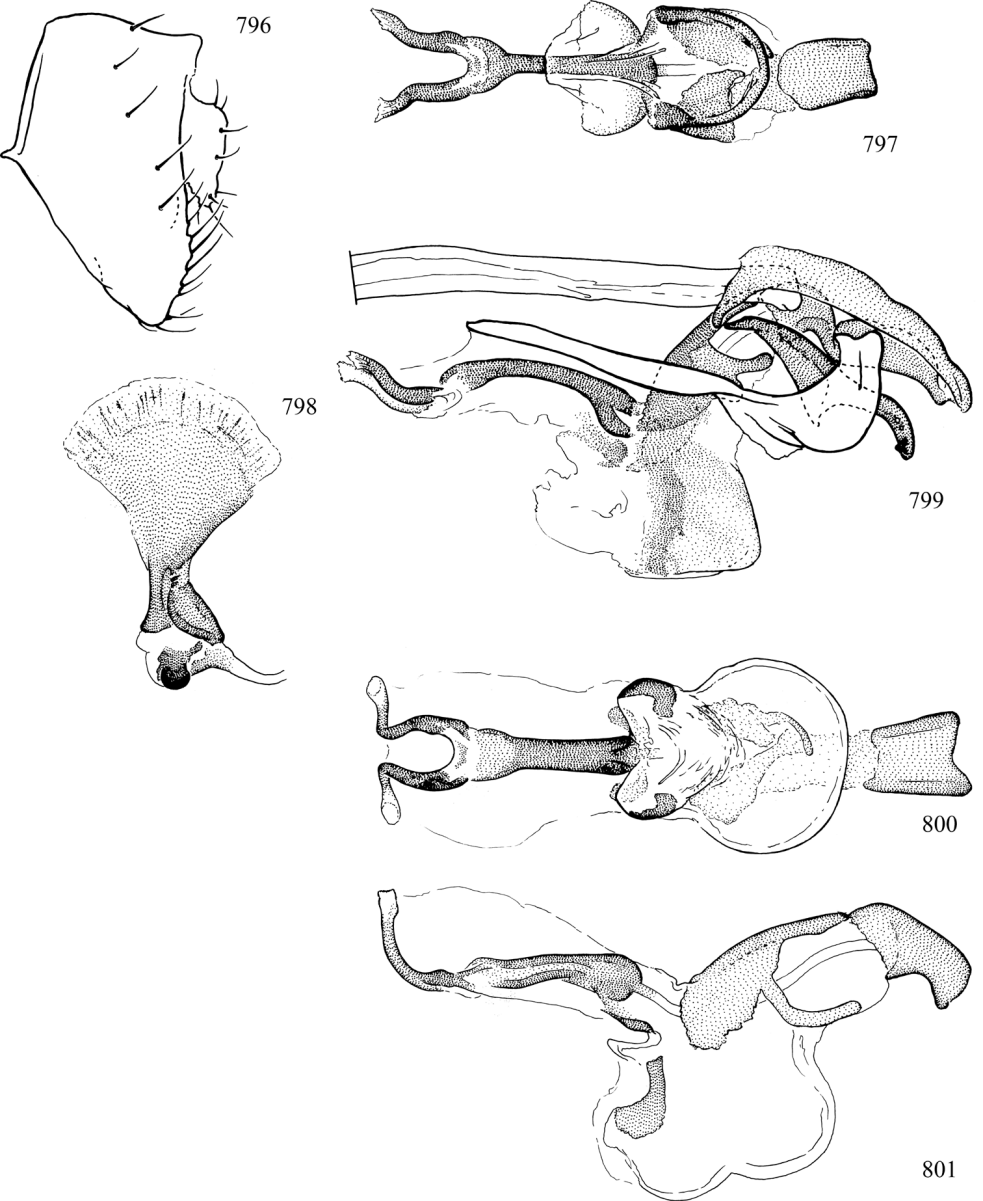
*Phytomyzaverticillatae* Kulp, male genitalia **796** external genitalia, left lateral **797** phallus, ventral **798** ejaculatory apodeme **799** hypandrial complex, left lateral **800, 801***P.wiggii* Lonsdale and Scheffer, male genitalia **800** phallus, ventral **801** hypandrial complex, left lateral. Originally published in Lonsdale and Scheffer (2011).

**Figures 802–804.**
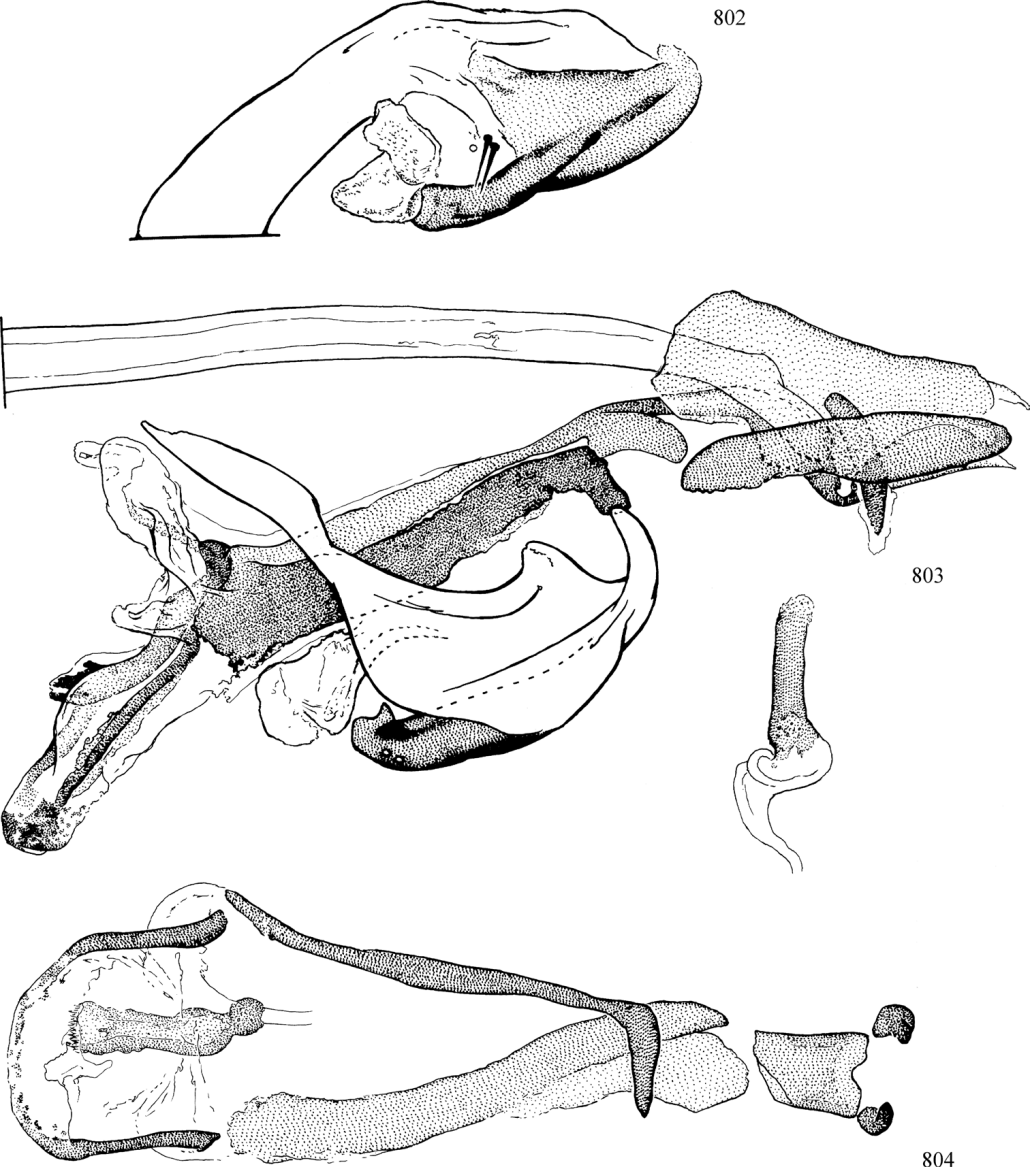
*Phytomyzalactuca* Frost, male genitalia **802** hypandrium and postgonite, ventral **803** hypandrial complex, left lateral **804** phallus, ventral.

**Figures 805–810.**
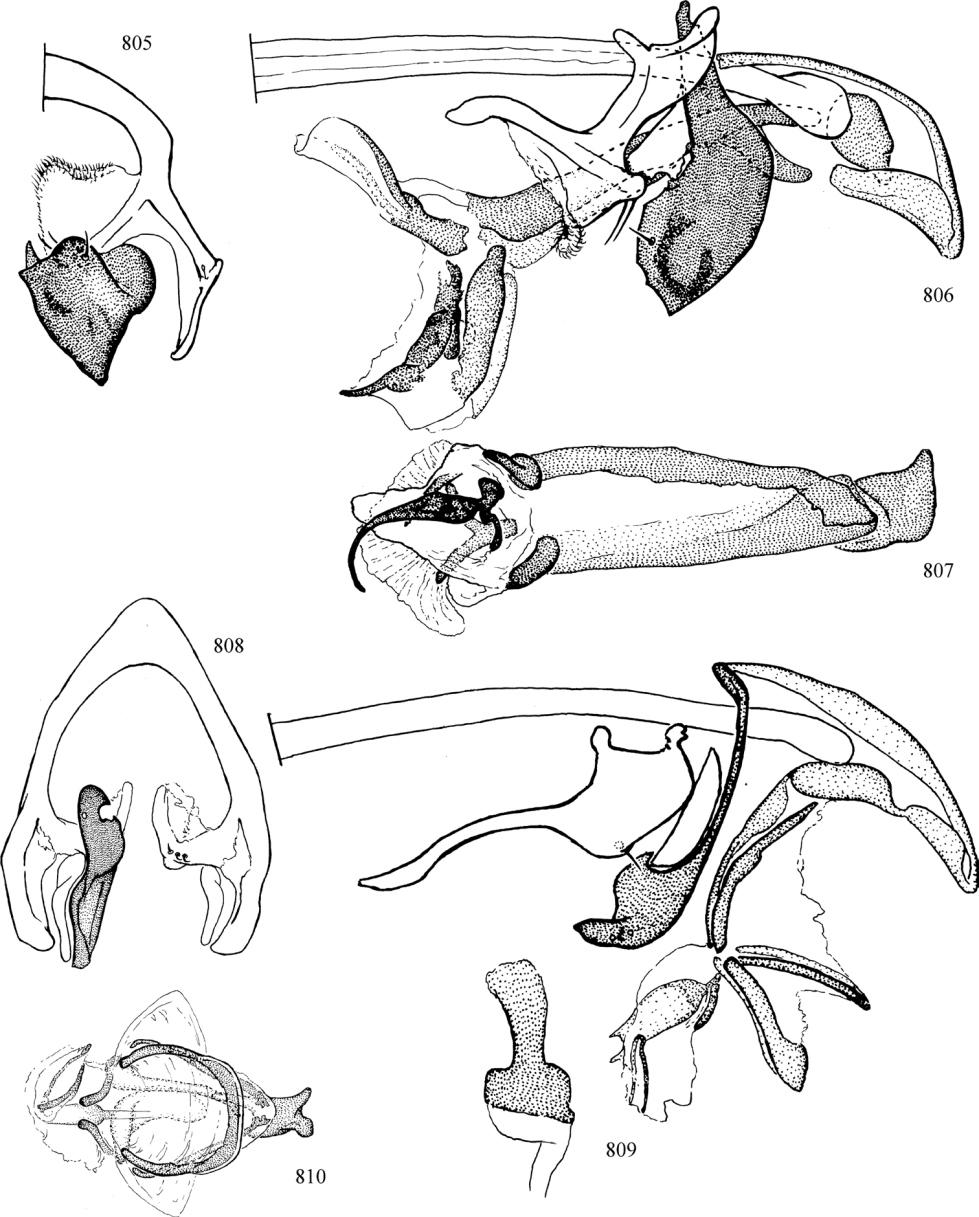
*Phytomyzaloewii* Hendel, male genitalia **805** hypandrium and postgonite, ventral **806** hypandrial complex, left lateral **807** phallus, ventral **808–810***P.nervosa* Loew, male genitalia **808** hypandrium and postgonite, ventral **809** hypandrial complex, left lateral **810** phallus, ventral.

**Figures 811–813.**
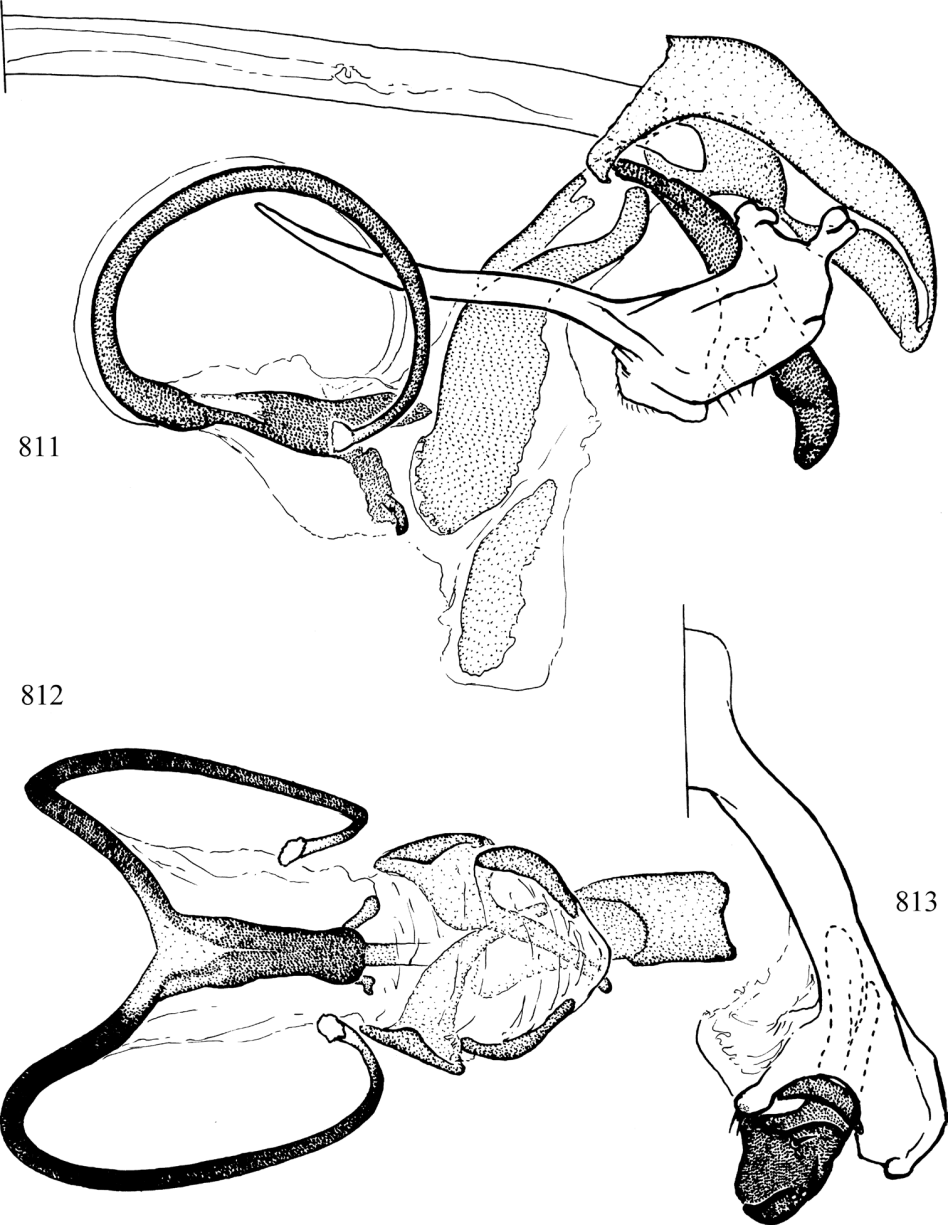
*Phytomyzapersicae* Frick, male genitalia **811** hypandrial complex, left lateral **812** phallus, ventral **813** hypandrium and postgonite, ventral.

**Figures 814–818.**
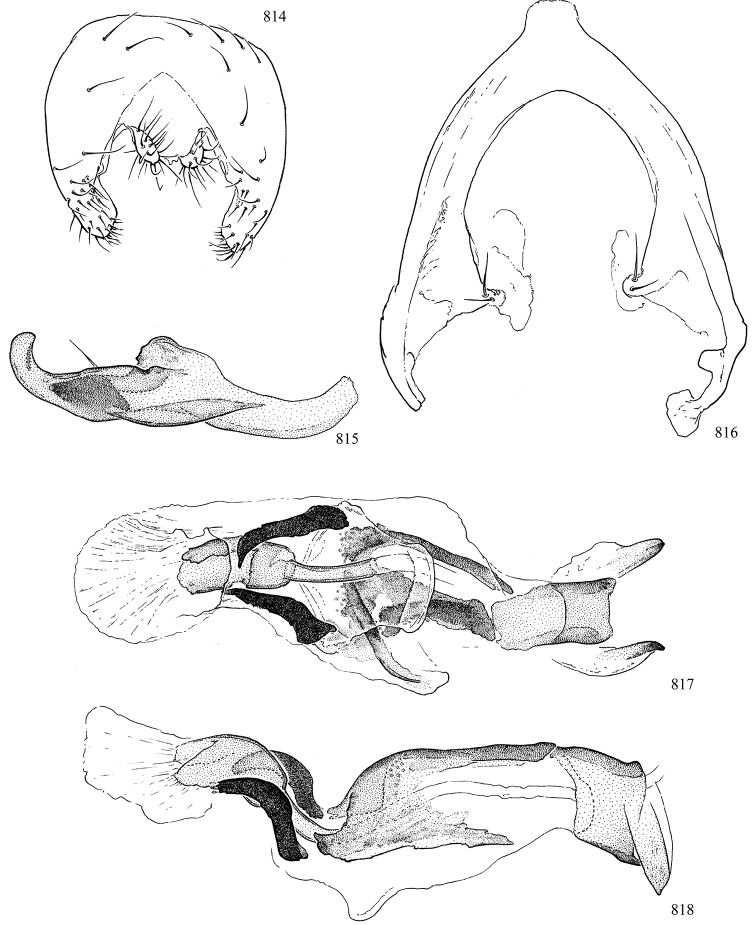
*Phytomyzaplantaginis* Robineau-Desvoidy, male genitalia **814** external genitalia, posterior **815** postgonite, lateral **816** hypandrium, ventral **817** phallus, ventral **818** phallus, left lateral.

**Figures 819–821.**
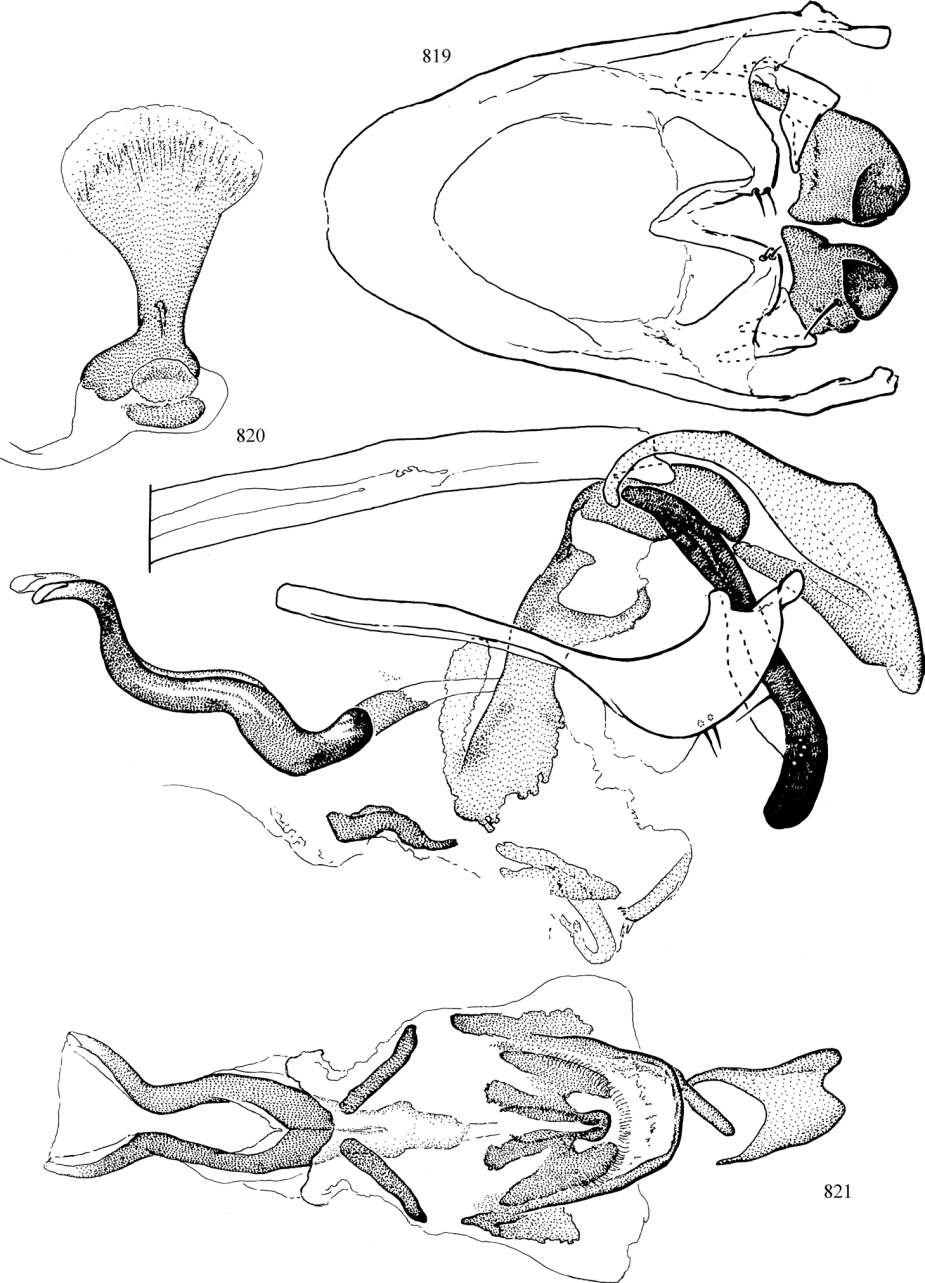
*Phytomyzasehgali* Spencer, male genitalia **819** hypandrium and postgonite, ventral **820** hypandrial complex, left lateral **821** phallus, ventral.

**Figures 822–824.**
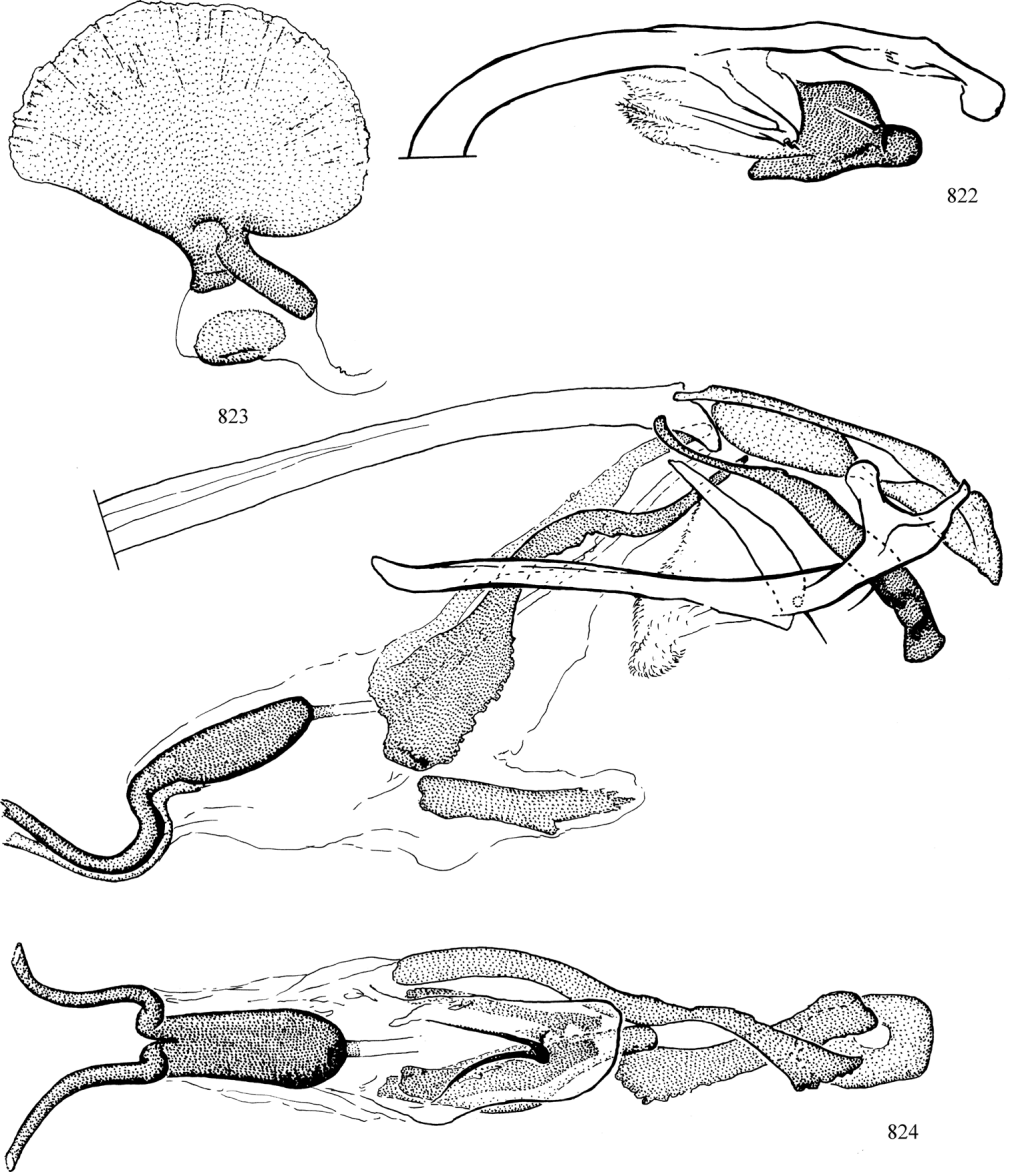
*Phytomyzapulchelloides* Henshaw and Howse, male genitalia **822** hypandrium and postgonite, ventral **823** hypandrial complex, left lateral **824** phallus, ventral.

**Figures 825–827.**
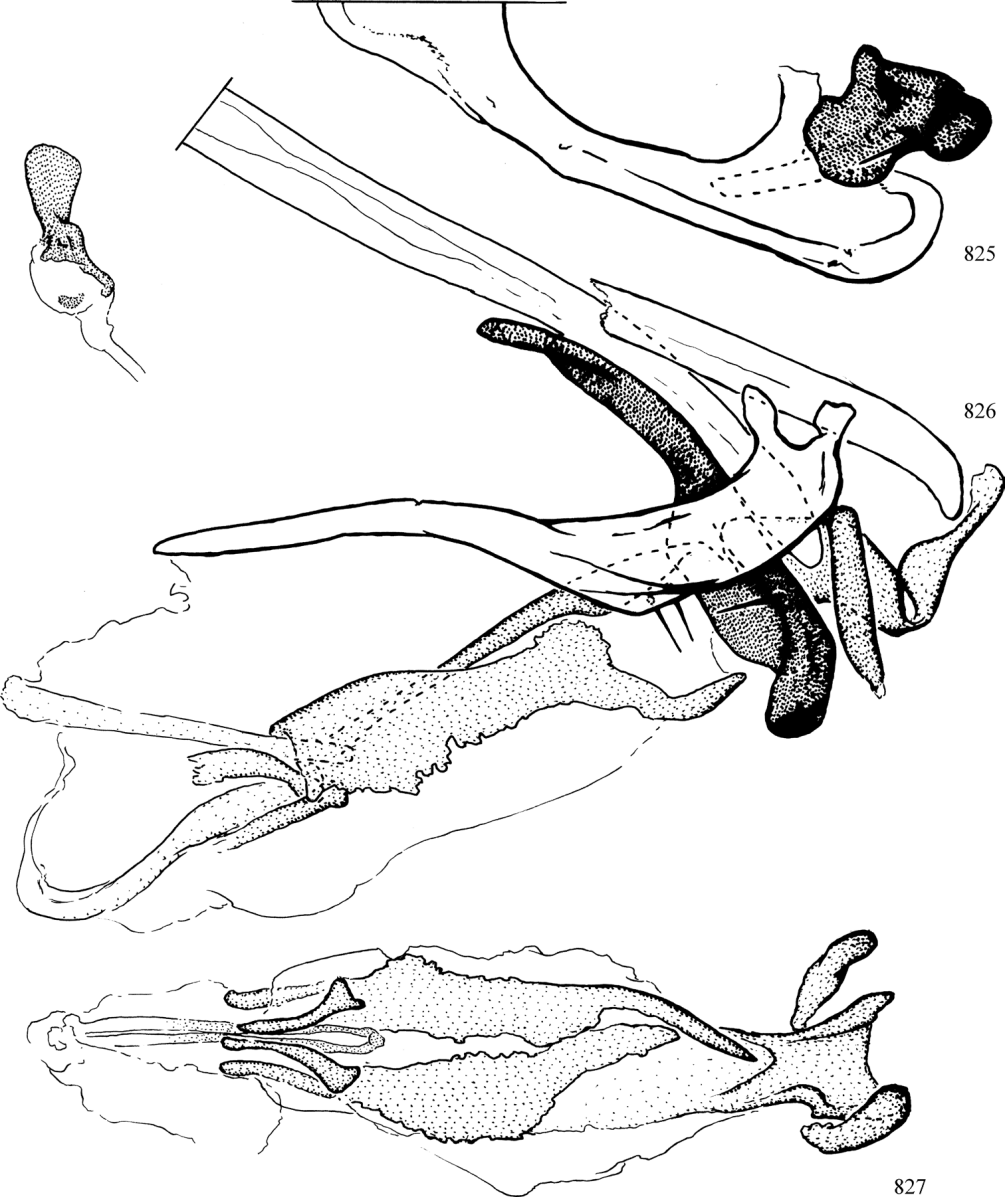
*Phytomyzavockerothi* Winkler, male genitalia **825** hypandrium and postgonite, ventral **826** hypandrial complex, left lateral **827** phallus, ventral.

**Figures 828–832.**
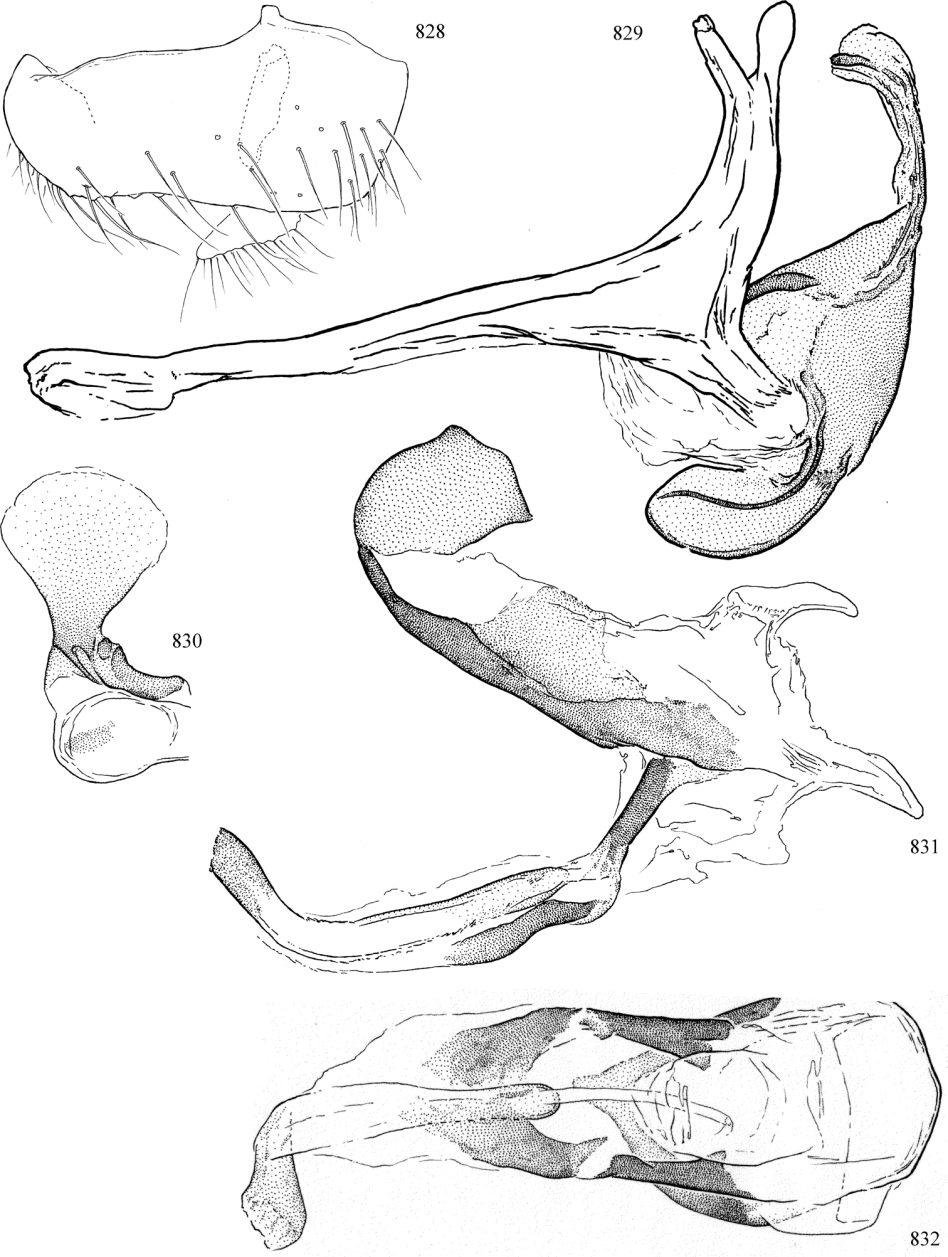
*Phytomyzawinkleri* sp. nov., male genitalia **828** external genitalia, left lateral **829** hypandrium and postgonite, left lateral **830** ejaculatory apodeme **831** phallus left lateral **832** phallus, ventral.

**Figures 833–839.**
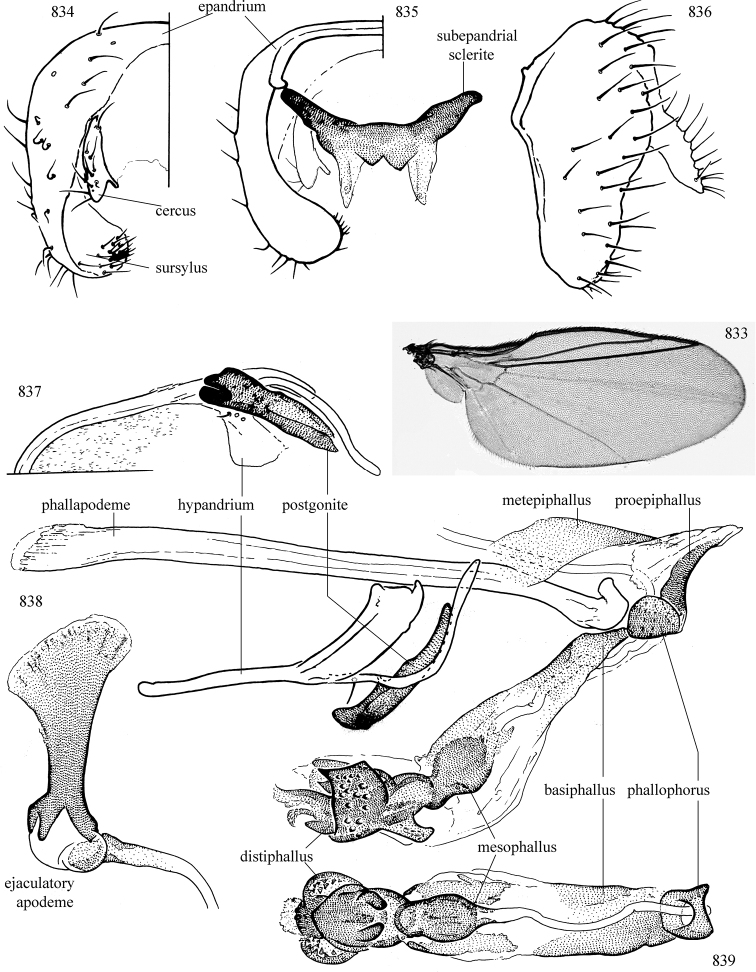
**833***Pseudonapomyzaatra* (Meigen), wing **834–839***P.atra*, male genitalia **834** external genitalia, posterior **835** external genitalia, anterior (subepandrial sclerite shaded) **836** external genitalia, left lateral **837** hypandrium and postgonite, ventral **838** hypandrial complex, left lateral **839** phallus, ventral.
